# Supplementary material for: Seminoma and Embryonal Carcinoma Footprints Identified by Analysis of Integrated Genome-Wide Epigenetic and Expression Profiles of Germ Cell Cancer Cell Lines
Source: PLoS One. 2014 Jun 2;9(6):e98330. doi: 10.1371/journal.pone.0098330 (PMC4041891; doi:10.1371/journal.pone.0098330)
Supplement: File S1 — ZIP file containing DMRforPairs output for significant regions. Please start from the html files. (ZIP) [file pone.0098330.s008.zip › relevant.html]

DMRforPairs output generated on Sun Oct 20 16:11:16 2013. Identified regions were set to contain at least 4 probes with a maximum distance of 200 bp between individual probes (n=30306). Regions in which median methylation levels (M-values) between the samples differed at least |1.4| (n=5139) (=relevant) were tested for statistical significance (significant: p<0.05; multiple testing adjusted, n=143). The folowing samples were studied: TCam2,NCCIT. Chromosomal positions are indicated in bp. N indicates the number of probes in a region. ID indicates the region ID. Values in the columns per sample indicate median beta values. dM indicates the largest median difference (absolute) between any of the sample pairs. p indicates uncorrected p-value from Mann-Whitney U test (n=2) or Kruskal Wallis test (n>2). p.adj denotes the multiple testing corrected p-value (method:bonferroni). Gene symbols of overlapping transcripts are listed for the exact region and within a margin of 10000 bp of the region.

| Thumbnail | Chr | Start | End | Links | Length | n | ID | Class | TCam2 | NCCIT | Gene.Symbol | dM | p | p.adj |
| --- | --- | --- | --- | --- | --- | --- | --- | --- | --- | --- | --- | --- | --- | --- |
|  | 6 | 27840105 | 27840257 | PDFSTATSENSEMBLUCSC | 153 | 6 | 34663 | tss | 0.01102 | 0.96427 | Not queried | 11.80 | 2.16e-03 | 1.00e+00 |
|  | 12 | 75785089 | 75785295 | PDFSTATSENSEMBLUCSC | 207 | 5 | 9197 | gene;island | 0.01220 | 0.96990 | Not queried | 11.39 | 7.94e-03 | 1.00e+00 |
|  | 15 | 89089914 | 89090195 | PDFSTATSENSEMBLUCSC | 282 | 4 | 13852 | tss | 0.98129 | 0.04995 | Not queried | 10.66 | 2.86e-02 | 1.00e+00 |
|  | 19 | 291239 | 291315 | PDFSTATSENSEMBLUCSC | 77 | 5 | 19587 | gene;tss;island | 0.02355 | 0.97214 | Not queried | 10.59 | 7.94e-03 | 1.00e+00 |
|  | 1 | 150947832 | 150948030 | PDFSTATSENSEMBLUCSC | 199 | 5 | 1912 | tss;island | 0.96070 | 0.02463 | Not queried | 10.50 | 7.94e-03 | 1.00e+00 |
|  | 1 | 161008811 | 161008977 | PDFSTATSENSEMBLUCSC | 167 | 4 | 2038 | tss | 0.02429 | 0.96836 | Not queried | 10.48 | 2.86e-02 | 1.00e+00 |
|  | 16 | 3414983 | 3415311 | PDFSTATSENSEMBLUCSC | 329 | 5 | 15616 | island | 0.00936 | 0.91281 | Not queried | 10.42 | 7.94e-03 | 1.00e+00 |
|  | X | 133941107 | 133941194 | PDFSTATSENSEMBLUCSC | 88 | 7 | 41533 | tss | 0.92463 | 0.01010 | Not queried | 10.39 | 5.83e-04 | 1.00e+00 |
|  | 2 | 63283806 | 63284132 | PDFSTATSENSEMBLUCSC | 327 | 5 | 22668 | gene;island | 0.97646 | 0.03421 | Not queried | 10.36 | 7.94e-03 | 1.00e+00 |
|  | 16 | 58548873 | 58549320 | PDFSTATSENSEMBLUCSC | 448 | 8 | 15244 | tss;island | 0.01536 | 0.94265 | Not queried | 10.10 | 1.55e-04 | 7.99e-01 |
|  | 4 | 144480206 | 144480561 | PDFSTATSENSEMBLUCSC | 356 | 11 | 30566 | tss | 0.00812 | 0.80744 | SMARCA5; GUSBP5 (margin: SMARCA5; GUSBP5; RP11-481K16.2) | 10.03 | 2.84e-06 | 1.46e-02 |
|  | 12 | 75784855 | 75784884 | PDFSTATSENSEMBLUCSC | 30 | 4 | 9856 | tss;island | 0.01659 | 0.95414 | Not queried | 9.95 | 2.86e-02 | 1.00e+00 |
|  | X | 133941107 | 133941225 | PDFSTATSENSEMBLUCSC | 119 | 8 | 41113 | gene | 0.91639 | 0.01550 | Not queried | 9.95 | 1.55e-04 | 7.99e-01 |
|  | 6 | 160182438 | 160182856 | PDFSTATSENSEMBLUCSC | 419 | 10 | 35257 | tss;island | 0.95376 | 0.02036 | Not queried | 9.85 | 1.08e-05 | 5.56e-02 |
|  | 4 | 144480206 | 144480740 | PDFSTATSENSEMBLUCSC | 535 | 12 | 31283 | island | 0.00838 | 0.85083 | SMARCA5; GUSBP5 (margin: SMARCA5; GUSBP5; RP11-481K16.2) | 9.83 | 7.40e-07 | 3.80e-03 |
|  | 7 | 154684327 | 154684562 | PDFSTATSENSEMBLUCSC | 236 | 4 | 37241 | gene;island | 0.02386 | 0.95080 | Not queried | 9.66 | 2.86e-02 | 1.00e+00 |
|  | 6 | 24358236 | 24358327 | PDFSTATSENSEMBLUCSC | 92 | 5 | 33526 | gene;island | 0.01419 | 0.93329 | Not queried | 9.63 | 7.94e-03 | 1.00e+00 |
|  | 4 | 103940711 | 103941300 | PDFSTATSENSEMBLUCSC | 590 | 11 | 31159 | island | 0.01663 | 0.89135 | SLC9B1; SLC9B2 (margin: SLC9B1; SLC9B2) | 9.62 | 2.84e-06 | 1.46e-02 |
|  | 4 | 103940876 | 103941300 | PDFSTATSENSEMBLUCSC | 425 | 9 | 30476 | tss | 0.01663 | 0.90335 | Not queried | 9.62 | 4.11e-05 | 2.11e-01 |
|  | 7 | 95025611 | 95026248 | PDFSTATSENSEMBLUCSC | 638 | 20 | 38614 | island | 0.02405 | 0.93699 | PON1; PON3 (margin: PON1; PON3; PON2) | 9.60 | 1.45e-11 | 7.46e-08 |
|  | 1 | 161008127 | 161008977 | PDFSTATSENSEMBLUCSC | 851 | 11 | 3884 | island | 0.03083 | 0.96123 | F11R; TSTD1; USF1 (margin: RP11-544M22.3; F11R; TSTD1; USF1; ARHGAP30) | 9.59 | 2.84e-06 | 1.46e-02 |
|  | 1 | 161008127 | 161008750 | PDFSTATSENSEMBLUCSC | 624 | 7 | 844 | gene | 0.03121 | 0.91634 | Not queried | 9.55 | 5.83e-04 | 1.00e+00 |
|  | 7 | 95025736 | 95026248 | PDFSTATSENSEMBLUCSC | 513 | 19 | 37661 | tss | 0.02143 | 0.93631 | PON1; PON3 (margin: PON1; PON3; PON2) | 9.50 | 5.66e-11 | 2.91e-07 |
|  | 16 | 29796620 | 29796798 | PDFSTATSENSEMBLUCSC | 179 | 4 | 15773 | island | 0.96569 | 0.04814 | Not queried | 9.43 | 2.86e-02 | 1.00e+00 |
|  | 5 | 172662315 | 172662463 | PDFSTATSENSEMBLUCSC | 149 | 4 | 32442 | tss | 0.02210 | 0.89061 | Not queried | 9.40 | 2.86e-02 | 1.00e+00 |
|  | 12 | 89748726 | 89749142 | PDFSTATSENSEMBLUCSC | 417 | 9 | 10639 | island | 0.89799 | 0.04113 | Not queried | 9.39 | 4.11e-05 | 2.11e-01 |
|  | 1 | 165737775 | 165738563 | PDFSTATSENSEMBLUCSC | 789 | 14 | 3921 | island | 0.89099 | 0.01537 | RP11-466F5.8; TMCO1 (margin: RP11-466F5.8; TMCO1) | 9.32 | 4.99e-08 | 2.56e-04 |
|  | 1 | 165737775 | 165738087 | PDFSTATSENSEMBLUCSC | 313 | 6 | 864 | gene | 0.77665 | 0.01158 | Not queried | 9.32 | 2.16e-03 | 1.00e+00 |
|  | 1 | 162337134 | 162337375 | PDFSTATSENSEMBLUCSC | 242 | 4 | 857 | gene;island | 0.03948 | 0.95308 | Not queried | 9.32 | 2.86e-02 | 1.00e+00 |
|  | 4 | 85414016 | 85414194 | PDFSTATSENSEMBLUCSC | 179 | 4 | 31106 | island | 0.03414 | 0.93475 | Not queried | 9.30 | 2.86e-02 | 1.00e+00 |
|  | 1 | 165738180 | 165738563 | PDFSTATSENSEMBLUCSC | 384 | 8 | 2065 | tss | 0.95631 | 0.02225 | Not queried | 9.30 | 1.55e-04 | 7.99e-01 |
|  | 7 | 149389669 | 149389941 | PDFSTATSENSEMBLUCSC | 273 | 4 | 38937 | island | 0.96657 | 0.04933 | Not queried | 9.29 | 2.86e-02 | 1.00e+00 |
|  | 18 | 21718735 | 21718979 | PDFSTATSENSEMBLUCSC | 245 | 10 | 19176 | tss;island | 0.03836 | 0.94397 | Not queried | 9.21 | 1.08e-05 | 5.56e-02 |
|  | X | 20134346 | 20134908 | PDFSTATSENSEMBLUCSC | 563 | 8 | 40946 | gene;island | 0.02038 | 0.92385 | Not queried | 9.17 | 1.55e-04 | 7.99e-01 |
|  | X | 20134778 | 20134908 | PDFSTATSENSEMBLUCSC | 131 | 4 | 41217 | tss | 0.01745 | 0.91853 | Not queried | 9.17 | 2.86e-02 | 1.00e+00 |
|  | 8 | 6692785 | 6693125 | PDFSTATSENSEMBLUCSC | 341 | 4 | 39128 | gene | 0.02536 | 0.87165 | Not queried | 9.14 | 2.86e-02 | 1.00e+00 |
|  | 6 | 27839751 | 27840257 | PDFSTATSENSEMBLUCSC | 507 | 10 | 35509 | island | 0.02719 | 0.94949 | Not queried | 9.09 | 1.08e-05 | 5.56e-02 |
|  | 14 | 65006688 | 65007512 | PDFSTATSENSEMBLUCSC | 825 | 18 | 12907 | island | 0.03705 | 0.90166 | HSPA2; RP11-973N13.4 (margin: ZBTB1; HSPA2; PPP1R36; RP11-973N13.4; RP11-973N13.5; RP11-973N13.3) | 9.07 | 2.20e-10 | 1.13e-06 |
|  | 19 | 21646329 | 21646781 | PDFSTATSENSEMBLUCSC | 453 | 5 | 21799 | island | 0.79716 | 0.01123 | Not queried | 9.04 | 7.94e-03 | 1.00e+00 |
|  | 19 | 50979561 | 50979637 | PDFSTATSENSEMBLUCSC | 77 | 4 | 20943 | tss | 0.02362 | 0.85525 | Not queried | 9.04 | 2.86e-02 | 1.00e+00 |
|  | 4 | 187985253 | 187985472 | PDFSTATSENSEMBLUCSC | 220 | 4 | 31435 | island | 0.03285 | 0.91228 | Not queried | 9.04 | 2.86e-02 | 1.00e+00 |
|  | 19 | 54290737 | 54291194 | PDFSTATSENSEMBLUCSC | 458 | 11 | 21003 | tss | 0.02519 | 0.93216 | MIR371A; MIR372; MIR373; AC008753.4; MIR371B (margin: AC008753.3; MIR371A; MIR372; MIR373; AC008753.4; MIR371B; NLRP12) | 8.99 | 2.84e-06 | 1.46e-02 |
|  | 3 | 39424697 | 39424992 | PDFSTATSENSEMBLUCSC | 296 | 6 | 28954 | island | 0.01773 | 0.95343 | Not queried | 8.99 | 2.16e-03 | 1.00e+00 |
|  | 2 | 139537745 | 139537845 | PDFSTATSENSEMBLUCSC | 101 | 4 | 24726 | island | 0.01400 | 0.87401 | Not queried | 8.98 | 2.86e-02 | 1.00e+00 |
|  | 14 | 65006688 | 65007171 | PDFSTATSENSEMBLUCSC | 484 | 15 | 12431 | tss | 0.04363 | 0.90142 | HSPA2; RP11-973N13.4 (margin: ZBTB1; HSPA2; PPP1R36; RP11-973N13.4; RP11-973N13.5) | 8.94 | 1.29e-08 | 6.63e-05 |
|  | 6 | 27832728 | 27833095 | PDFSTATSENSEMBLUCSC | 368 | 8 | 34661 | tss | 0.03343 | 0.95911 | Not queried | 8.94 | 1.55e-04 | 7.99e-01 |
|  | 3 | 39425775 | 39426101 | PDFSTATSENSEMBLUCSC | 327 | 4 | 27683 | gene;island | 0.05556 | 0.96319 | Not queried | 8.92 | 2.86e-02 | 1.00e+00 |
|  | 5 | 8457548 | 8458089 | PDFSTATSENSEMBLUCSC | 542 | 6 | 32622 | island | 0.02126 | 0.91076 | Not queried | 8.90 | 2.16e-03 | 1.00e+00 |
|  | 6 | 27832728 | 27833555 | PDFSTATSENSEMBLUCSC | 828 | 13 | 35507 | island | 0.02640 | 0.94173 | HIST1H2AL; HIST1H2BPS2; HIST1H1B (margin: HIST1H2BN; HIST1H2AL; HIST1H2BPS2; HIST1H1B; HIST1H3I; HIST1H4L) | 8.90 | 3.85e-07 | 1.98e-03 |
|  | 6 | 27833121 | 27833555 | PDFSTATSENSEMBLUCSC | 435 | 5 | 33558 | gene | 0.02246 | 0.91631 | Not queried | 8.90 | 7.94e-03 | 1.00e+00 |
|  | 8 | 37605552 | 37605978 | PDFSTATSENSEMBLUCSC | 427 | 5 | 39582 | tss | 0.03565 | 0.94799 | Not queried | 8.89 | 7.94e-03 | 1.00e+00 |
|  | 3 | 186857284 | 186857593 | PDFSTATSENSEMBLUCSC | 310 | 7 | 28735 | tss;island | 0.01799 | 0.90171 | Not queried | 8.89 | 5.83e-04 | 1.00e+00 |
|  | 10 | 50887472 | 50887632 | PDFSTATSENSEMBLUCSC | 161 | 7 | 5059 | tss | 0.01532 | 0.85417 | Not queried | 8.84 | 5.83e-04 | 1.00e+00 |
|  | 19 | 11530021 | 11530065 | PDFSTATSENSEMBLUCSC | 45 | 4 | 20435 | tss | 0.94156 | 0.04473 | Not queried | 8.82 | 2.86e-02 | 1.00e+00 |
|  | 8 | 80803196 | 80803526 | PDFSTATSENSEMBLUCSC | 331 | 5 | 40345 | island | 0.02697 | 0.91067 | Not queried | 8.78 | 7.94e-03 | 1.00e+00 |
|  | 15 | 78556834 | 78557094 | PDFSTATSENSEMBLUCSC | 261 | 6 | 13408 | gene;tss;island | 0.04285 | 0.93962 | Not queried | 8.77 | 2.16e-03 | 1.00e+00 |
|  | 12 | 7781004 | 7781431 | PDFSTATSENSEMBLUCSC | 428 | 5 | 10201 | island | 0.03621 | 0.93350 | Not queried | 8.73 | 1.51e-01 | 1.00e+00 |
|  | 15 | 40544855 | 40545511 | PDFSTATSENSEMBLUCSC | 657 | 11 | 13261 | gene;island | 0.01977 | 0.89700 | RP11-133K1.2; PAK6; C15orf56 (margin: RP11-133K1.2; PAK6; C15orf56) | 8.73 | 2.84e-06 | 1.46e-02 |
|  | 5 | 179740743 | 179741120 | PDFSTATSENSEMBLUCSC | 378 | 4 | 31883 | gene;island | 0.01214 | 0.83445 | Not queried | 8.72 | 2.86e-02 | 1.00e+00 |
|  | 15 | 91473059 | 91473569 | PDFSTATSENSEMBLUCSC | 511 | 9 | 14433 | island | 0.01579 | 0.88853 | Not queried | 8.72 | 4.11e-05 | 2.11e-01 |
|  | 7 | 27205159 | 27205658 | PDFSTATSENSEMBLUCSC | 500 | 10 | 37431 | tss | 0.01815 | 0.89898 | Not queried | 8.71 | 1.08e-05 | 5.56e-02 |
|  | 3 | 109035381 | 109035781 | PDFSTATSENSEMBLUCSC | 401 | 6 | 28450 | tss | 0.01786 | 0.86308 | Not queried | 8.69 | 2.16e-03 | 1.00e+00 |
|  | 19 | 50861774 | 50862121 | PDFSTATSENSEMBLUCSC | 348 | 5 | 20137 | gene;island | 0.03010 | 0.85631 | Not queried | 8.69 | 7.94e-03 | 1.00e+00 |
|  | 6 | 10390811 | 10391412 | PDFSTATSENSEMBLUCSC | 602 | 6 | 35360 | island | 0.93433 | 0.03855 | Not queried | 8.68 | 2.16e-03 | 1.00e+00 |
|  | 20 | 30134929 | 30135177 | PDFSTATSENSEMBLUCSC | 249 | 8 | 25623 | tss | 0.97013 | 0.08380 | Not queried | 8.68 | 1.55e-04 | 7.99e-01 |
|  | 10 | 134599783 | 134600998 | PDFSTATSENSEMBLUCSC | 1216 | 30 | 5432 | tss;island | 0.03311 | 0.93434 | RP11-288G11.3; NKX6-2 (margin: INPP5A; RP11-288G11.3; NKX6-2) | 8.67 | 1.69e-17 | 8.69e-14 |
|  | 11 | 15095017 | 15095178 | PDFSTATSENSEMBLUCSC | 162 | 6 | 8061 | island | 0.02096 | 0.86387 | Not queried | 8.62 | 2.16e-03 | 1.00e+00 |
|  | X | 113815930 | 113816304 | PDFSTATSENSEMBLUCSC | 375 | 5 | 41966 | island | 0.03224 | 0.92793 | Not queried | 8.61 | 7.94e-03 | 1.00e+00 |
|  | 1 | 179560700 | 179561111 | PDFSTATSENSEMBLUCSC | 412 | 6 | 3988 | island | 0.01445 | 0.86061 | Not queried | 8.58 | 2.16e-03 | 1.00e+00 |
|  | 18 | 21719263 | 21719433 | PDFSTATSENSEMBLUCSC | 171 | 5 | 19177 | tss | 0.06129 | 0.93350 | Not queried | 8.57 | 7.94e-03 | 1.00e+00 |
|  | 6 | 27835363 | 27835460 | PDFSTATSENSEMBLUCSC | 98 | 6 | 34662 | tss | 0.03731 | 0.92585 | Not queried | 8.55 | 2.16e-03 | 1.00e+00 |
|  | 6 | 24358304 | 24358327 | PDFSTATSENSEMBLUCSC | 24 | 4 | 34602 | tss | 0.02476 | 0.92632 | Not queried | 8.55 | 2.86e-02 | 1.00e+00 |
|  | 16 | 82044738 | 82045078 | PDFSTATSENSEMBLUCSC | 341 | 5 | 14826 | gene | 0.03669 | 0.91932 | Not queried | 8.54 | 7.94e-03 | 1.00e+00 |
|  | 7 | 45026259 | 45026583 | PDFSTATSENSEMBLUCSC | 325 | 8 | 37511 | tss;island | 0.93583 | 0.02678 | Not queried | 8.53 | 1.55e-04 | 7.99e-01 |
|  | 13 | 113420153 | 113420371 | PDFSTATSENSEMBLUCSC | 219 | 4 | 11222 | gene;island | 0.03990 | 0.94757 | Not queried | 8.53 | 2.86e-02 | 1.00e+00 |
|  | 2 | 234776807 | 234777085 | PDFSTATSENSEMBLUCSC | 279 | 5 | 25174 | island | 0.01852 | 0.87342 | Not queried | 8.51 | 7.94e-03 | 1.00e+00 |
|  | 20 | 30134929 | 30135362 | PDFSTATSENSEMBLUCSC | 434 | 10 | 25341 | gene;island | 0.97013 | 0.08380 | Not queried | 8.51 | 1.08e-05 | 5.56e-02 |
|  | 1 | 234367145 | 234367586 | PDFSTATSENSEMBLUCSC | 442 | 5 | 1082 | gene;island | 0.01665 | 0.92147 | Not queried | 8.48 | 7.94e-03 | 1.00e+00 |
|  | 6 | 27835191 | 27835460 | PDFSTATSENSEMBLUCSC | 270 | 7 | 35508 | island | 0.03571 | 0.92826 | Not queried | 8.48 | 5.83e-04 | 1.00e+00 |
|  | 5 | 176559881 | 176560265 | PDFSTATSENSEMBLUCSC | 385 | 4 | 32462 | tss;island | 0.01656 | 0.84381 | Not queried | 8.43 | 2.86e-02 | 1.00e+00 |
|  | 1 | 150254280 | 150254546 | PDFSTATSENSEMBLUCSC | 267 | 5 | 1901 | tss;island | 0.04870 | 0.94628 | Not queried | 8.43 | 7.94e-03 | 1.00e+00 |
|  | 7 | 111368367 | 111368847 | PDFSTATSENSEMBLUCSC | 481 | 4 | 37125 | gene;island | 0.04517 | 0.91340 | Not queried | 8.40 | 2.86e-02 | 1.00e+00 |
|  | 15 | 99409194 | 99409506 | PDFSTATSENSEMBLUCSC | 313 | 4 | 13468 | gene | 0.02855 | 0.89758 | Not queried | 8.39 | 2.86e-02 | 1.00e+00 |
|  | 5 | 180258557 | 180258852 | PDFSTATSENSEMBLUCSC | 296 | 5 | 31889 | gene;island | 0.91704 | 0.03020 | Not queried | 8.36 | 7.94e-03 | 1.00e+00 |
|  | 13 | 100547192 | 100547367 | PDFSTATSENSEMBLUCSC | 176 | 5 | 11179 | gene;island | 0.04541 | 0.94878 | Not queried | 8.36 | 7.94e-03 | 1.00e+00 |
|  | 12 | 64784070 | 64784252 | PDFSTATSENSEMBLUCSC | 183 | 4 | 9178 | gene;island | 0.03331 | 0.89519 | Not queried | 8.32 | 2.86e-02 | 1.00e+00 |
|  | 1 | 209979111 | 209979470 | PDFSTATSENSEMBLUCSC | 360 | 5 | 985 | gene | 0.00816 | 0.82463 | Not queried | 8.31 | 7.94e-03 | 1.00e+00 |
|  | 6 | 27782484 | 27782658 | PDFSTATSENSEMBLUCSC | 175 | 5 | 34656 | tss;island | 0.00755 | 0.54945 | Not queried | 8.27 | 7.94e-03 | 1.00e+00 |
|  | 7 | 121651766 | 121652024 | PDFSTATSENSEMBLUCSC | 259 | 4 | 37141 | gene | 0.05188 | 0.93791 | Not queried | 8.27 | 2.86e-02 | 1.00e+00 |
|  | 7 | 23287222 | 23287560 | PDFSTATSENSEMBLUCSC | 339 | 4 | 36851 | gene;island | 0.01997 | 0.86601 | Not queried | 8.27 | 2.86e-02 | 1.00e+00 |
|  | 8 | 37605359 | 37605978 | PDFSTATSENSEMBLUCSC | 620 | 7 | 39216 | gene | 0.04379 | 0.94799 | Not queried | 8.26 | 5.83e-04 | 1.00e+00 |
|  | 17 | 36997420 | 36997627 | PDFSTATSENSEMBLUCSC | 208 | 4 | 16507 | gene | 0.04557 | 0.94266 | Not queried | 8.25 | 2.86e-02 | 1.00e+00 |
|  | 18 | 21719263 | 21719568 | PDFSTATSENSEMBLUCSC | 306 | 7 | 19044 | gene;island | 0.06207 | 0.91153 | Not queried | 8.24 | 5.83e-04 | 1.00e+00 |
|  | 1 | 179560700 | 179560983 | PDFSTATSENSEMBLUCSC | 284 | 5 | 2115 | tss | 0.01450 | 0.85319 | Not queried | 8.23 | 7.94e-03 | 1.00e+00 |
|  | 11 | 7110074 | 7110196 | PDFSTATSENSEMBLUCSC | 123 | 5 | 8004 | island | 0.02345 | 0.86500 | Not queried | 8.21 | 7.94e-03 | 1.00e+00 |
|  | 4 | 187422005 | 187422119 | PDFSTATSENSEMBLUCSC | 115 | 5 | 31427 | island | 0.04050 | 0.87308 | Not queried | 8.20 | 7.94e-03 | 1.00e+00 |
|  | 12 | 131303016 | 131303247 | PDFSTATSENSEMBLUCSC | 232 | 4 | 9392 | gene;island | 0.01976 | 0.85662 | Not queried | 8.18 | 2.86e-02 | 1.00e+00 |
|  | 6 | 26033394 | 26034145 | PDFSTATSENSEMBLUCSC | 752 | 13 | 34618 | tss;island | 0.01093 | 0.81336 | HIST1H3B; HIST1H2AB (margin: HIST1H4A; U91328.2; HIST1H3C; HIST1H4B; HIST1H3B; HIST1H2AB; HIST1H2BB) | 8.15 | 1.92e-07 | 9.88e-04 |
|  | 16 | 29296614 | 29296797 | PDFSTATSENSEMBLUCSC | 184 | 4 | 15769 | island | 0.01862 | 0.67507 | Not queried | 8.14 | 2.86e-02 | 1.00e+00 |
|  | 11 | 130029961 | 130030459 | PDFSTATSENSEMBLUCSC | 499 | 4 | 6956 | gene;island | 0.02321 | 0.82595 | Not queried | 8.14 | 2.86e-02 | 1.00e+00 |
|  | 4 | 48491941 | 48492344 | PDFSTATSENSEMBLUCSC | 404 | 8 | 30981 | island | 0.05676 | 0.90610 | Not queried | 8.13 | 1.55e-04 | 7.99e-01 |
|  | 2 | 166650805 | 166650965 | PDFSTATSENSEMBLUCSC | 161 | 6 | 23657 | tss | 0.02316 | 0.85930 | Not queried | 8.12 | 2.16e-03 | 1.00e+00 |
|  | 2 | 113342070 | 113342262 | PDFSTATSENSEMBLUCSC | 193 | 4 | 22793 | gene | 0.02042 | 0.86764 | Not queried | 8.12 | 2.86e-02 | 1.00e+00 |
|  | 1 | 59042745 | 59043070 | PDFSTATSENSEMBLUCSC | 326 | 4 | 556 | gene | 0.06667 | 0.90849 | Not queried | 8.11 | 2.86e-02 | 1.00e+00 |
|  | 2 | 187713554 | 187714279 | PDFSTATSENSEMBLUCSC | 726 | 12 | 24912 | island | 0.03341 | 0.91428 | ZSWIM2 (margin: ZSWIM2) | 8.10 | 7.40e-07 | 3.80e-03 |
|  | 6 | 24877462 | 24877729 | PDFSTATSENSEMBLUCSC | 268 | 4 | 33530 | gene | 0.03440 | 0.91979 | Not queried | 8.09 | 2.86e-02 | 1.00e+00 |
|  | 2 | 166650606 | 166650965 | PDFSTATSENSEMBLUCSC | 360 | 9 | 24794 | island | 0.03001 | 0.83333 | Not queried | 8.09 | 4.11e-05 | 2.11e-01 |
|  | 3 | 170746029 | 170746265 | PDFSTATSENSEMBLUCSC | 237 | 5 | 28674 | tss | 0.03112 | 0.88366 | Not queried | 8.08 | 7.94e-03 | 1.00e+00 |
|  | 18 | 7011217 | 7011463 | PDFSTATSENSEMBLUCSC | 247 | 4 | 19018 | gene;island | 0.09564 | 0.93804 | Not queried | 8.01 | 2.86e-02 | 1.00e+00 |
|  | 2 | 225266733 | 225266880 | PDFSTATSENSEMBLUCSC | 148 | 6 | 23887 | tss | 0.01542 | 0.81557 | Not queried | 8.00 | 2.16e-03 | 1.00e+00 |
|  | 4 | 48491941 | 48492304 | PDFSTATSENSEMBLUCSC | 364 | 7 | 30336 | tss | 0.07113 | 0.90283 | Not queried | 8.00 | 5.83e-04 | 1.00e+00 |
|  | 7 | 97602481 | 97602715 | PDFSTATSENSEMBLUCSC | 235 | 4 | 37676 | tss;island | 0.95573 | 0.07921 | Not queried | 7.99 | 2.86e-02 | 1.00e+00 |
|  | 17 | 17109570 | 17110353 | PDFSTATSENSEMBLUCSC | 784 | 14 | 18027 | island | 0.05499 | 0.94317 | MPRIP; PLD6; RP11-45M22.4 (margin: MPRIP; PLD6; RP11-45M22.4; FLCN) | 7.98 | 9.97e-08 | 5.12e-04 |
|  | 4 | 56023751 | 56024045 | PDFSTATSENSEMBLUCSC | 295 | 4 | 31006 | island | 0.05930 | 0.92843 | Not queried | 7.98 | 2.86e-02 | 1.00e+00 |
|  | 3 | 9404422 | 9404837 | PDFSTATSENSEMBLUCSC | 416 | 9 | 28812 | island | 0.87427 | 0.03068 | Not queried | 7.98 | 4.11e-05 | 2.11e-01 |
|  | 3 | 9404422 | 9404684 | PDFSTATSENSEMBLUCSC | 263 | 7 | 28082 | tss | 0.88224 | 0.03068 | Not queried | 7.98 | 5.83e-04 | 1.00e+00 |
|  | 19 | 1074727 | 1075104 | PDFSTATSENSEMBLUCSC | 378 | 4 | 19606 | gene;island | 0.02600 | 0.84611 | Not queried | 7.95 | 2.86e-02 | 1.00e+00 |
|  | 14 | 61748243 | 61748440 | PDFSTATSENSEMBLUCSC | 198 | 4 | 12077 | gene;island | 0.02814 | 0.85989 | Not queried | 7.95 | 2.86e-02 | 1.00e+00 |
|  | 15 | 91473059 | 91473386 | PDFSTATSENSEMBLUCSC | 328 | 6 | 13878 | tss | 0.03576 | 0.89739 | Not queried | 7.93 | 2.16e-03 | 1.00e+00 |
|  | 2 | 73429084 | 73429539 | PDFSTATSENSEMBLUCSC | 456 | 8 | 24374 | island | 0.95476 | 0.07225 | Not queried | 7.92 | 1.55e-04 | 7.99e-01 |
|  | 20 | 52199520 | 52199778 | PDFSTATSENSEMBLUCSC | 259 | 5 | 26223 | island | 0.08439 | 0.90248 | Not queried | 7.90 | 7.94e-03 | 1.00e+00 |
|  | 12 | 54332992 | 54333323 | PDFSTATSENSEMBLUCSC | 332 | 4 | 9129 | gene;island | 0.01084 | 0.67248 | Not queried | 7.88 | 2.86e-02 | 1.00e+00 |
|  | 1 | 59042745 | 59043370 | PDFSTATSENSEMBLUCSC | 626 | 10 | 3307 | island | 0.02713 | 0.88002 | Not queried | 7.87 | 1.08e-05 | 5.56e-02 |
|  | 7 | 48495072 | 48495333 | PDFSTATSENSEMBLUCSC | 262 | 4 | 36945 | gene;island | 0.04772 | 0.91243 | Not queried | 7.86 | 2.86e-02 | 1.00e+00 |
|  | 20 | 61051029 | 61051039 | PDFSTATSENSEMBLUCSC | 11 | 4 | 25832 | tss | 0.01815 | 0.80397 | Not queried | 7.86 | 2.86e-02 | 1.00e+00 |
|  | 6 | 34724739 | 34724845 | PDFSTATSENSEMBLUCSC | 107 | 5 | 34858 | tss;island | 0.02667 | 0.89123 | Not queried | 7.85 | 7.94e-03 | 1.00e+00 |
|  | 12 | 64784483 | 64784686 | PDFSTATSENSEMBLUCSC | 204 | 5 | 9816 | tss;island | 0.04889 | 0.90648 | Not queried | 7.84 | 7.94e-03 | 1.00e+00 |
|  | 5 | 179779115 | 179779237 | PDFSTATSENSEMBLUCSC | 123 | 4 | 31885 | gene;island | 0.86507 | 0.04894 | Not queried | 7.84 | 2.86e-02 | 1.00e+00 |
|  | 19 | 33210498 | 33210851 | PDFSTATSENSEMBLUCSC | 354 | 5 | 21834 | island | 0.03319 | 0.89451 | Not queried | 7.84 | 7.94e-03 | 1.00e+00 |
|  | 4 | 779480 | 779880 | PDFSTATSENSEMBLUCSC | 401 | 4 | 29793 | gene;island | 0.03548 | 0.89383 | Not queried | 7.84 | 2.86e-02 | 1.00e+00 |
|  | 3 | 170746029 | 170746291 | PDFSTATSENSEMBLUCSC | 263 | 6 | 29617 | island | 0.03472 | 0.88057 | Not queried | 7.83 | 2.16e-03 | 1.00e+00 |
|  | 15 | 40545143 | 40545511 | PDFSTATSENSEMBLUCSC | 369 | 9 | 13556 | tss | 0.01759 | 0.85795 | Not queried | 7.82 | 4.11e-05 | 2.11e-01 |
|  | 3 | 87138203 | 87138700 | PDFSTATSENSEMBLUCSC | 498 | 6 | 29257 | island | 0.03926 | 0.90923 | Not queried | 7.82 | 2.16e-03 | 1.00e+00 |
|  | 11 | 130029478 | 130029684 | PDFSTATSENSEMBLUCSC | 207 | 5 | 8933 | island | 0.04930 | 0.92126 | Not queried | 7.82 | 7.94e-03 | 1.00e+00 |
|  | 10 | 49812686 | 49813128 | PDFSTATSENSEMBLUCSC | 443 | 4 | 4567 | gene | 0.02834 | 0.83465 | Not queried | 7.82 | 2.86e-02 | 1.00e+00 |
|  | 1 | 209848845 | 209849185 | PDFSTATSENSEMBLUCSC | 341 | 5 | 983 | gene;island | 0.87628 | 0.03055 | Not queried | 7.81 | 7.94e-03 | 1.00e+00 |
|  | 13 | 36871646 | 36871962 | PDFSTATSENSEMBLUCSC | 317 | 6 | 11101 | gene | 0.96347 | 0.10961 | Not queried | 7.81 | 2.16e-03 | 1.00e+00 |
|  | 1 | 8013974 | 8014650 | PDFSTATSENSEMBLUCSC | 677 | 6 | 2625 | island | 0.04738 | 0.87225 | Not queried | 7.81 | 2.16e-03 | 1.00e+00 |
|  | X | 8751190 | 8751687 | PDFSTATSENSEMBLUCSC | 498 | 7 | 41624 | island | 0.02642 | 0.91226 | Not queried | 7.80 | 5.83e-04 | 1.00e+00 |
|  | 2 | 73429084 | 73429371 | PDFSTATSENSEMBLUCSC | 288 | 7 | 23399 | tss | 0.94896 | 0.05955 | Not queried | 7.80 | 5.83e-04 | 1.00e+00 |
|  | 7 | 48494362 | 48494734 | PDFSTATSENSEMBLUCSC | 373 | 5 | 36944 | gene;island | 0.02817 | 0.87326 | Not queried | 7.76 | 7.94e-03 | 1.00e+00 |
|  | 5 | 156696185 | 156696520 | PDFSTATSENSEMBLUCSC | 336 | 4 | 31816 | gene;island | 0.04131 | 0.83740 | Not queried | 7.75 | 2.86e-02 | 1.00e+00 |
|  | 13 | 36871646 | 36872346 | PDFSTATSENSEMBLUCSC | 701 | 14 | 11639 | island | 0.94549 | 0.09210 | SOHLH2; CCDC169-SOHLH2; CCDC169 (margin: SOHLH2; CCDC169-SOHLH2; CCDC169; SPG20) | 7.75 | 4.99e-08 | 2.56e-04 |
|  | 2 | 187713902 | 187714279 | PDFSTATSENSEMBLUCSC | 378 | 9 | 23737 | tss | 0.03151 | 0.82817 | Not queried | 7.75 | 4.11e-05 | 2.11e-01 |
|  | 11 | 130029478 | 130029615 | PDFSTATSENSEMBLUCSC | 138 | 4 | 7795 | tss | 0.05268 | 0.92436 | Not queried | 7.72 | 2.86e-02 | 1.00e+00 |
|  | X | 153284899 | 153285158 | PDFSTATSENSEMBLUCSC | 260 | 4 | 41154 | gene;island | 0.01487 | 0.80619 | Not queried | 7.72 | 2.86e-02 | 1.00e+00 |
|  | 6 | 84140564 | 84140997 | PDFSTATSENSEMBLUCSC | 434 | 8 | 36228 | island | 0.03057 | 0.91471 | Not queried | 7.72 | 1.55e-04 | 7.99e-01 |
|  | 2 | 45159894 | 45160554 | PDFSTATSENSEMBLUCSC | 661 | 8 | 24222 | island | 0.90392 | 0.06398 | Not queried | 7.71 | 1.55e-04 | 7.99e-01 |
|  | 19 | 12306136 | 12306497 | PDFSTATSENSEMBLUCSC | 362 | 4 | 21561 | island | 0.01954 | 0.87090 | Not queried | 7.70 | 2.86e-02 | 1.00e+00 |
|  | 4 | 188916496 | 188916943 | PDFSTATSENSEMBLUCSC | 448 | 11 | 31437 | island | 0.96312 | 0.11854 | ZFP42 (margin: ZFP42) | 7.69 | 2.84e-06 | 1.46e-02 |
|  | 19 | 12075601 | 12075848 | PDFSTATSENSEMBLUCSC | 248 | 5 | 20450 | tss | 0.01073 | 0.67328 | Not queried | 7.69 | 7.94e-03 | 1.00e+00 |
|  | X | 149716155 | 149716393 | PDFSTATSENSEMBLUCSC | 239 | 4 | 42095 | island | 0.07104 | 0.92328 | Not queried | 7.68 | 2.86e-02 | 1.00e+00 |
|  | 22 | 36903287 | 36903611 | PDFSTATSENSEMBLUCSC | 325 | 6 | 27067 | tss;island | 0.02539 | 0.88235 | Not queried | 7.68 | 1.52e-02 | 1.00e+00 |
|  | 6 | 26240307 | 26240579 | PDFSTATSENSEMBLUCSC | 273 | 5 | 34635 | tss;island | 0.01173 | 0.70687 | Not queried | 7.67 | 7.94e-03 | 1.00e+00 |
|  | 17 | 74533845 | 74534090 | PDFSTATSENSEMBLUCSC | 246 | 7 | 17648 | tss;island | 0.05128 | 0.87093 | Not queried | 7.65 | 5.83e-04 | 1.00e+00 |
|  | 22 | 50064736 | 50065058 | PDFSTATSENSEMBLUCSC | 323 | 4 | 27563 | island | 0.04304 | 0.87097 | Not queried | 7.65 | 2.86e-02 | 1.00e+00 |
|  | 12 | 7072696 | 7072891 | PDFSTATSENSEMBLUCSC | 196 | 4 | 9514 | tss | 0.07813 | 0.90443 | Not queried | 7.62 | 2.86e-02 | 1.00e+00 |
|  | 14 | 55907007 | 55907501 | PDFSTATSENSEMBLUCSC | 495 | 10 | 12841 | island | 0.04414 | 0.82780 | Not queried | 7.62 | 1.08e-05 | 5.56e-02 |
|  | 19 | 21657529 | 21658001 | PDFSTATSENSEMBLUCSC | 473 | 6 | 21800 | island | 0.89143 | 0.05281 | Not queried | 7.62 | 2.16e-03 | 1.00e+00 |
|  | 22 | 22901569 | 22901661 | PDFSTATSENSEMBLUCSC | 93 | 4 | 26767 | gene | 0.02123 | 0.83234 | Not queried | 7.61 | 2.86e-02 | 1.00e+00 |
|  | 10 | 50887472 | 50887934 | PDFSTATSENSEMBLUCSC | 463 | 10 | 5719 | island | 0.02436 | 0.84748 | Not queried | 7.61 | 1.08e-05 | 5.56e-02 |
|  | 2 | 113341711 | 113342262 | PDFSTATSENSEMBLUCSC | 552 | 12 | 24586 | island | 0.02745 | 0.85022 | CHCHD5 (margin: POLR1B; CHCHD5; AC012442.5; AC012442.6; Y\_RNA) | 7.61 | 7.40e-07 | 3.80e-03 |
|  | 2 | 113341711 | 113341977 | PDFSTATSENSEMBLUCSC | 267 | 8 | 23541 | tss | 0.03128 | 0.85022 | Not queried | 7.60 | 1.55e-04 | 7.99e-01 |
|  | 8 | 58055876 | 58056175 | PDFSTATSENSEMBLUCSC | 300 | 4 | 40254 | island | 0.09566 | 0.92403 | Not queried | 7.59 | 2.86e-02 | 1.00e+00 |
|  | 2 | 131046122 | 131046551 | PDFSTATSENSEMBLUCSC | 430 | 4 | 24680 | island | 0.03290 | 0.88335 | Not queried | 7.59 | 2.86e-02 | 1.00e+00 |
|  | X | 153285622 | 153285934 | PDFSTATSENSEMBLUCSC | 313 | 5 | 41590 | tss;island | 0.05775 | 0.86536 | Not queried | 7.58 | 7.94e-03 | 1.00e+00 |
|  | 19 | 58520847 | 58521273 | PDFSTATSENSEMBLUCSC | 427 | 6 | 22453 | island | 0.03253 | 0.88397 | Not queried | 7.58 | 2.16e-03 | 1.00e+00 |
|  | 13 | 36872048 | 36872346 | PDFSTATSENSEMBLUCSC | 299 | 8 | 11365 | tss | 0.94021 | 0.06589 | Not queried | 7.57 | 1.55e-04 | 7.99e-01 |
|  | 5 | 3188066 | 3188374 | PDFSTATSENSEMBLUCSC | 309 | 5 | 32601 | island | 0.05160 | 0.93482 | Not queried | 7.56 | 7.94e-03 | 1.00e+00 |
|  | 17 | 43246883 | 43247290 | PDFSTATSENSEMBLUCSC | 408 | 4 | 16597 | gene;island | 0.05336 | 0.89771 | Not queried | 7.56 | 2.86e-02 | 1.00e+00 |
|  | 6 | 27258460 | 27258767 | PDFSTATSENSEMBLUCSC | 308 | 6 | 35489 | island | 0.92281 | 0.03937 | Not queried | 7.54 | 2.16e-03 | 1.00e+00 |
|  | 14 | 104583632 | 104584279 | PDFSTATSENSEMBLUCSC | 648 | 6 | 13173 | island | 0.05562 | 0.87820 | Not queried | 7.53 | 2.16e-03 | 1.00e+00 |
|  | 1 | 236686564 | 236686804 | PDFSTATSENSEMBLUCSC | 241 | 5 | 1095 | gene;tss;island | 0.06307 | 0.86925 | Not queried | 7.52 | 7.94e-03 | 1.00e+00 |
|  | 2 | 3260576 | 3261155 | PDFSTATSENSEMBLUCSC | 580 | 5 | 22506 | gene | 0.13322 | 0.82791 | Not queried | 7.52 | 7.94e-03 | 1.00e+00 |
|  | 6 | 16306377 | 16306857 | PDFSTATSENSEMBLUCSC | 481 | 4 | 33522 | gene | 0.06106 | 0.90297 | Not queried | 7.51 | 2.86e-02 | 1.00e+00 |
|  | 6 | 26021916 | 26022273 | PDFSTATSENSEMBLUCSC | 358 | 5 | 33532 | gene | 0.12071 | 0.94958 | Not queried | 7.51 | 7.94e-03 | 1.00e+00 |
|  | 6 | 10419835 | 10420079 | PDFSTATSENSEMBLUCSC | 245 | 7 | 34560 | tss | 0.03384 | 0.80483 | Not queried | 7.50 | 5.83e-04 | 1.00e+00 |
|  | 14 | 55907299 | 55907501 | PDFSTATSENSEMBLUCSC | 203 | 7 | 12386 | tss | 0.05232 | 0.82192 | Not queried | 7.50 | 5.83e-04 | 1.00e+00 |
|  | 14 | 54430382 | 54430831 | PDFSTATSENSEMBLUCSC | 450 | 4 | 12826 | island | 0.02445 | 0.87721 | Not queried | 7.50 | 2.86e-02 | 1.00e+00 |
|  | 15 | 23810163 | 23810378 | PDFSTATSENSEMBLUCSC | 216 | 5 | 13486 | tss | 0.07822 | 0.93121 | Not queried | 7.49 | 7.94e-03 | 1.00e+00 |
|  | 20 | 61050885 | 61051039 | PDFSTATSENSEMBLUCSC | 155 | 7 | 26280 | island | 0.02368 | 0.81008 | Not queried | 7.48 | 5.83e-04 | 1.00e+00 |
|  | 4 | 188916496 | 188916875 | PDFSTATSENSEMBLUCSC | 380 | 10 | 30662 | tss | 0.96089 | 0.11016 | Not queried | 7.47 | 1.08e-05 | 5.56e-02 |
|  | 7 | 27138712 | 27138974 | PDFSTATSENSEMBLUCSC | 263 | 4 | 38273 | island | 0.93916 | 0.08941 | Not queried | 7.46 | 2.86e-02 | 1.00e+00 |
|  | 6 | 10416337 | 10416531 | PDFSTATSENSEMBLUCSC | 195 | 4 | 33498 | gene;tss;island | 0.84675 | 0.03795 | Not queried | 7.46 | 2.86e-02 | 1.00e+00 |
|  | 7 | 27204663 | 27205658 | PDFSTATSENSEMBLUCSC | 996 | 17 | 38292 | island | 0.02568 | 0.77553 | HOXA9; RP1-170O19.20 (margin: HOXA-AS3; RP1-170O19.21; HOXA-AS4; HOXA7; HOXA9; RP1-170O19.20; MIR196B; HOXA10) | 7.46 | 8.57e-10 | 4.40e-06 |
|  | 20 | 61002595 | 61002866 | PDFSTATSENSEMBLUCSC | 272 | 6 | 25831 | tss | 0.02276 | 0.80987 | Not queried | 7.45 | 2.16e-03 | 1.00e+00 |
|  | 8 | 55533655 | 55534103 | PDFSTATSENSEMBLUCSC | 449 | 7 | 39243 | gene;island | 0.03814 | 0.84950 | Not queried | 7.43 | 5.83e-04 | 1.00e+00 |
|  | 8 | 24772137 | 24772435 | PDFSTATSENSEMBLUCSC | 299 | 7 | 39541 | tss | 0.05704 | 0.91136 | Not queried | 7.41 | 5.83e-04 | 1.00e+00 |
|  | 11 | 15095017 | 15095070 | PDFSTATSENSEMBLUCSC | 54 | 5 | 7148 | tss | 0.03234 | 0.85084 | Not queried | 7.40 | 7.94e-03 | 1.00e+00 |
|  | 7 | 65878734 | 65879115 | PDFSTATSENSEMBLUCSC | 382 | 4 | 38470 | island | 0.01518 | 0.66878 | Not queried | 7.40 | 2.86e-02 | 1.00e+00 |
|  | 6 | 160211355 | 160211577 | PDFSTATSENSEMBLUCSC | 223 | 6 | 35259 | tss;island | 0.76892 | 0.01876 | Not queried | 7.40 | 2.16e-03 | 1.00e+00 |
|  | 17 | 43221220 | 43221575 | PDFSTATSENSEMBLUCSC | 356 | 4 | 18391 | island | 0.02670 | 0.84002 | Not queried | 7.38 | 2.86e-02 | 1.00e+00 |
|  | 2 | 74357527 | 74357872 | PDFSTATSENSEMBLUCSC | 346 | 4 | 24387 | island | 0.02233 | 0.81291 | Not queried | 7.38 | 2.86e-02 | 1.00e+00 |
|  | 6 | 10419684 | 10420079 | PDFSTATSENSEMBLUCSC | 396 | 8 | 35368 | island | 0.04219 | 0.79468 | Not queried | 7.36 | 1.55e-04 | 7.99e-01 |
|  | 4 | 52917271 | 52917567 | PDFSTATSENSEMBLUCSC | 297 | 9 | 30343 | tss | 0.90564 | 0.07837 | Not queried | 7.34 | 4.11e-05 | 2.11e-01 |
|  | 8 | 143781297 | 143781406 | PDFSTATSENSEMBLUCSC | 110 | 4 | 39869 | tss;island | 0.02975 | 0.77073 | Not queried | 7.33 | 2.86e-02 | 1.00e+00 |
|  | 10 | 21797112 | 21797191 | PDFSTATSENSEMBLUCSC | 80 | 4 | 5575 | island | 0.78928 | 0.02256 | Not queried | 7.33 | 2.86e-02 | 1.00e+00 |
|  | 7 | 1113014 | 1113237 | PDFSTATSENSEMBLUCSC | 224 | 4 | 36683 | gene | 0.04396 | 0.90399 | Not queried | 7.32 | 2.86e-02 | 1.00e+00 |
|  | 1 | 59043173 | 59043370 | PDFSTATSENSEMBLUCSC | 198 | 6 | 1651 | tss | 0.02555 | 0.78057 | Not queried | 7.32 | 2.16e-03 | 1.00e+00 |
|  | 13 | 100547728 | 100548078 | PDFSTATSENSEMBLUCSC | 351 | 5 | 11180 | gene;island | 0.06000 | 0.93890 | Not queried | 7.31 | 7.94e-03 | 1.00e+00 |
|  | X | 83757470 | 83757693 | PDFSTATSENSEMBLUCSC | 224 | 8 | 41408 | tss | 0.02390 | 0.71093 | Not queried | 7.30 | 1.55e-04 | 7.99e-01 |
|  | 6 | 26033394 | 26033786 | PDFSTATSENSEMBLUCSC | 393 | 6 | 33534 | gene | 0.01307 | 0.69523 | Not queried | 7.30 | 2.16e-03 | 1.00e+00 |
|  | X | 119133700 | 119134082 | PDFSTATSENSEMBLUCSC | 383 | 5 | 41994 | island | 0.91256 | 0.06222 | Not queried | 7.30 | 7.94e-03 | 1.00e+00 |
|  | 12 | 52626814 | 52627047 | PDFSTATSENSEMBLUCSC | 234 | 6 | 10411 | island | 0.03118 | 0.83634 | Not queried | 7.28 | 2.16e-03 | 1.00e+00 |
|  | 1 | 91192451 | 91192803 | PDFSTATSENSEMBLUCSC | 353 | 5 | 3457 | island | 0.82731 | 0.05999 | Not queried | 7.28 | 7.94e-03 | 1.00e+00 |
|  | 1 | 2980380 | 2980598 | PDFSTATSENSEMBLUCSC | 219 | 4 | 94 | gene;island | 0.03522 | 0.83688 | Not queried | 7.28 | 2.86e-02 | 1.00e+00 |
|  | 22 | 21386885 | 21387058 | PDFSTATSENSEMBLUCSC | 174 | 7 | 26950 | tss | 0.02559 | 0.74364 | Not queried | 7.28 | 5.83e-04 | 1.00e+00 |
|  | 19 | 2289982 | 2290107 | PDFSTATSENSEMBLUCSC | 126 | 4 | 19650 | gene;island | 0.04571 | 0.87808 | Not queried | 7.28 | 2.86e-02 | 1.00e+00 |
|  | 1 | 63249197 | 63249213 | PDFSTATSENSEMBLUCSC | 17 | 4 | 1663 | tss | 0.02830 | 0.84162 | Not queried | 7.27 | 2.86e-02 | 1.00e+00 |
|  | 11 | 117069780 | 117069966 | PDFSTATSENSEMBLUCSC | 187 | 7 | 7695 | tss | 0.02835 | 0.84932 | Not queried | 7.27 | 5.83e-04 | 1.00e+00 |
|  | 22 | 21386798 | 21387058 | PDFSTATSENSEMBLUCSC | 261 | 8 | 27288 | island | 0.02648 | 0.75974 | Not queried | 7.26 | 1.55e-04 | 7.99e-01 |
|  | X | 134429653 | 134429972 | PDFSTATSENSEMBLUCSC | 320 | 4 | 41114 | gene;island | 0.04585 | 0.88283 | Not queried | 7.25 | 2.86e-02 | 1.00e+00 |
|  | 22 | 37212787 | 37213130 | PDFSTATSENSEMBLUCSC | 344 | 4 | 26819 | gene;island | 0.91572 | 0.03503 | Not queried | 7.25 | 2.86e-02 | 1.00e+00 |
|  | 2 | 106886503 | 106886729 | PDFSTATSENSEMBLUCSC | 227 | 4 | 24553 | island | 0.01299 | 0.50599 | Not queried | 7.24 | 2.86e-02 | 1.00e+00 |
|  | 6 | 26021768 | 26022273 | PDFSTATSENSEMBLUCSC | 506 | 9 | 35443 | island | 0.12071 | 0.94958 | Not queried | 7.23 | 4.11e-05 | 2.11e-01 |
|  | 11 | 34460107 | 34460557 | PDFSTATSENSEMBLUCSC | 451 | 10 | 8182 | island | 0.02558 | 0.81088 | Not queried | 7.23 | 1.08e-05 | 5.56e-02 |
|  | 8 | 52722023 | 52722237 | PDFSTATSENSEMBLUCSC | 215 | 5 | 39631 | tss | 0.03736 | 0.83653 | Not queried | 7.23 | 7.94e-03 | 1.00e+00 |
|  | 14 | 104583048 | 104583245 | PDFSTATSENSEMBLUCSC | 198 | 4 | 12629 | tss;island | 0.03992 | 0.82290 | Not queried | 7.22 | 2.86e-02 | 1.00e+00 |
|  | 6 | 164392967 | 164393860 | PDFSTATSENSEMBLUCSC | 894 | 8 | 36557 | island | 0.07239 | 0.85147 | Not queried | 7.22 | 1.55e-04 | 7.99e-01 |
|  | 5 | 140530028 | 140530606 | PDFSTATSENSEMBLUCSC | 579 | 5 | 31752 | gene;island | 0.10467 | 0.95127 | Not queried | 7.19 | 7.94e-03 | 1.00e+00 |
|  | 11 | 34460107 | 34460386 | PDFSTATSENSEMBLUCSC | 280 | 8 | 7221 | tss | 0.02742 | 0.81088 | Not queried | 7.17 | 1.55e-04 | 7.99e-01 |
|  | 6 | 76203530 | 76203675 | PDFSTATSENSEMBLUCSC | 146 | 6 | 35013 | tss | 0.04415 | 0.87764 | Not queried | 7.16 | 2.16e-03 | 1.00e+00 |
|  | 7 | 914964 | 915203 | PDFSTATSENSEMBLUCSC | 240 | 4 | 37325 | tss;island | 0.07134 | 0.92817 | Not queried | 7.16 | 2.86e-02 | 1.00e+00 |
|  | 22 | 24181212 | 24181270 | PDFSTATSENSEMBLUCSC | 59 | 4 | 26971 | tss | 0.67259 | 0.01448 | Not queried | 7.15 | 2.86e-02 | 1.00e+00 |
|  | 22 | 24180995 | 24181270 | PDFSTATSENSEMBLUCSC | 276 | 6 | 27312 | island | 0.64213 | 0.01448 | Not queried | 7.15 | 2.16e-03 | 1.00e+00 |
|  | 13 | 41768782 | 41768978 | PDFSTATSENSEMBLUCSC | 197 | 5 | 11382 | tss | 0.02159 | 0.73505 | Not queried | 7.15 | 7.94e-03 | 1.00e+00 |
|  | 14 | 75593812 | 75594058 | PDFSTATSENSEMBLUCSC | 247 | 9 | 12488 | tss | 0.76159 | 0.02069 | Not queried | 7.15 | 4.11e-05 | 2.11e-01 |
|  | 4 | 89618324 | 89619573 | PDFSTATSENSEMBLUCSC | 1250 | 20 | 30009 | gene | 0.04982 | 0.91616 | HERC3; NAP1L5 (margin: HERC3; FAM13A-AS1; NAP1L5) | 7.14 | 2.32e-08 | 1.19e-04 |
|  | 5 | 1594676 | 1595048 | PDFSTATSENSEMBLUCSC | 373 | 8 | 31912 | tss | 0.96342 | 0.14518 | Not queried | 7.14 | 1.55e-04 | 7.99e-01 |
|  | 12 | 6649733 | 6649994 | PDFSTATSENSEMBLUCSC | 262 | 4 | 9004 | gene;island | 0.04367 | 0.85483 | Not queried | 7.12 | 2.86e-02 | 1.00e+00 |
|  | 22 | 29711916 | 29712139 | PDFSTATSENSEMBLUCSC | 224 | 6 | 27005 | tss;island | 0.93169 | 0.07224 | Not queried | 7.12 | 2.16e-03 | 1.00e+00 |
|  | 19 | 15343235 | 15343425 | PDFSTATSENSEMBLUCSC | 191 | 6 | 20521 | tss | 0.85072 | 0.04046 | Not queried | 7.12 | 2.16e-03 | 1.00e+00 |
|  | 12 | 52626814 | 52626904 | PDFSTATSENSEMBLUCSC | 91 | 5 | 9700 | tss | 0.03210 | 0.80271 | Not queried | 7.11 | 7.94e-03 | 1.00e+00 |
|  | 17 | 36997420 | 36997740 | PDFSTATSENSEMBLUCSC | 321 | 7 | 18228 | island | 0.06639 | 0.91160 | Not queried | 7.11 | 5.83e-04 | 1.00e+00 |
|  | 12 | 65562903 | 65563329 | PDFSTATSENSEMBLUCSC | 427 | 7 | 9819 | tss;island | 0.71037 | 0.01763 | Not queried | 7.09 | 4.08e-03 | 1.00e+00 |
|  | 11 | 75139390 | 75139736 | PDFSTATSENSEMBLUCSC | 347 | 4 | 6817 | gene;island | 0.01376 | 0.62436 | Not queried | 7.09 | 2.86e-02 | 1.00e+00 |
|  | X | 100353031 | 100353462 | PDFSTATSENSEMBLUCSC | 432 | 6 | 41907 | island | 0.02274 | 0.68991 | Not queried | 7.09 | 2.16e-03 | 1.00e+00 |
|  | 6 | 27259010 | 27259237 | PDFSTATSENSEMBLUCSC | 228 | 5 | 35490 | island | 0.88516 | 0.05714 | Not queried | 7.08 | 7.94e-03 | 1.00e+00 |
|  | 7 | 39649224 | 39649443 | PDFSTATSENSEMBLUCSC | 220 | 5 | 36911 | gene;island | 0.01812 | 0.77272 | Not queried | 7.08 | 7.94e-03 | 1.00e+00 |
|  | 5 | 1594579 | 1595048 | PDFSTATSENSEMBLUCSC | 470 | 9 | 32575 | island | 0.96122 | 0.14631 | Not queried | 7.07 | 4.11e-05 | 2.11e-01 |
|  | 17 | 17109651 | 17110353 | PDFSTATSENSEMBLUCSC | 703 | 12 | 17129 | tss | 0.11191 | 0.94317 | MPRIP; PLD6; RP11-45M22.4 (margin: MPRIP; PLD6; RP11-45M22.4; FLCN) | 7.07 | 1.48e-06 | 7.60e-03 |
|  | 1 | 236687053 | 236687277 | PDFSTATSENSEMBLUCSC | 225 | 4 | 1096 | gene;island | 0.04696 | 0.79982 | Not queried | 7.04 | 2.86e-02 | 1.00e+00 |
|  | 8 | 114389383 | 114389479 | PDFSTATSENSEMBLUCSC | 97 | 4 | 39812 | tss | 0.05779 | 0.84163 | Not queried | 7.03 | 2.86e-02 | 1.00e+00 |
|  | 8 | 24772137 | 24772513 | PDFSTATSENSEMBLUCSC | 377 | 8 | 39183 | gene;island | 0.06134 | 0.91791 | Not queried | 7.02 | 1.55e-04 | 7.99e-01 |
|  | 5 | 140207460 | 140207636 | PDFSTATSENSEMBLUCSC | 177 | 5 | 32293 | tss | 0.06297 | 0.86472 | Not queried | 7.01 | 7.94e-03 | 1.00e+00 |
|  | 22 | 36877697 | 36877737 | PDFSTATSENSEMBLUCSC | 41 | 4 | 27066 | tss | 0.01260 | 0.59779 | Not queried | 6.99 | 2.86e-02 | 1.00e+00 |
|  | 5 | 54826547 | 54826768 | PDFSTATSENSEMBLUCSC | 222 | 4 | 31562 | gene;island | 0.01582 | 0.63050 | Not queried | 6.99 | 5.71e-02 | 1.00e+00 |
|  | 10 | 118900324 | 118900492 | PDFSTATSENSEMBLUCSC | 169 | 4 | 6154 | island | 0.91452 | 0.09738 | Not queried | 6.98 | 2.86e-02 | 1.00e+00 |
|  | 12 | 66583029 | 66583431 | PDFSTATSENSEMBLUCSC | 403 | 5 | 9182 | gene;island | 0.03316 | 0.84151 | Not queried | 6.97 | 7.94e-03 | 1.00e+00 |
|  | 14 | 105714153 | 105714589 | PDFSTATSENSEMBLUCSC | 437 | 6 | 12230 | gene;tss;island | 0.10548 | 0.91835 | Not queried | 6.97 | 2.16e-03 | 1.00e+00 |
|  | 1 | 158119395 | 158119673 | PDFSTATSENSEMBLUCSC | 279 | 5 | 3865 | island | 0.04065 | 0.85340 | Not queried | 6.96 | 7.94e-03 | 1.00e+00 |
|  | 6 | 26021768 | 26021843 | PDFSTATSENSEMBLUCSC | 76 | 4 | 34615 | tss | 0.14909 | 0.95043 | Not queried | 6.95 | 2.86e-02 | 1.00e+00 |
|  | 14 | 55493573 | 55494452 | PDFSTATSENSEMBLUCSC | 880 | 10 | 12058 | gene;tss;island | 0.71947 | 0.02434 | Not queried | 6.95 | 1.08e-05 | 5.56e-02 |
|  | 6 | 30698584 | 30698987 | PDFSTATSENSEMBLUCSC | 404 | 11 | 33755 | gene | 0.04332 | 0.87591 | FLOT1 (margin: TUBB; XXbac-BPG252P9.10; XXbac-BPG252P9.9; FLOT1; Y\_RNA; IER3) | 6.94 | 2.84e-06 | 1.46e-02 |
|  | 2 | 128458240 | 128458556 | PDFSTATSENSEMBLUCSC | 317 | 6 | 23578 | tss | 0.91678 | 0.05747 | Not queried | 6.92 | 2.16e-03 | 1.00e+00 |
|  | 5 | 76010444 | 76010658 | PDFSTATSENSEMBLUCSC | 215 | 4 | 32075 | tss;island | 0.93070 | 0.08555 | Not queried | 6.91 | 2.86e-02 | 1.00e+00 |
|  | 7 | 50132541 | 50132764 | PDFSTATSENSEMBLUCSC | 224 | 4 | 36947 | gene | 0.04805 | 0.80515 | Not queried | 6.91 | 2.86e-02 | 1.00e+00 |
|  | 7 | 124404244 | 124404472 | PDFSTATSENSEMBLUCSC | 229 | 4 | 37144 | gene;island | 0.02319 | 0.65492 | Not queried | 6.91 | 2.86e-02 | 1.00e+00 |
|  | 19 | 52391078 | 52391789 | PDFSTATSENSEMBLUCSC | 712 | 13 | 22309 | island | 0.17681 | 0.95794 | CTC-429C10.2; ZNF577; ZNF649 (margin: CTC-429C10.2; ZNF577; ZNF649) | 6.90 | 1.92e-07 | 9.88e-04 |
|  | 7 | 138720786 | 138721065 | PDFSTATSENSEMBLUCSC | 280 | 9 | 37862 | tss;island | 0.04033 | 0.83668 | Not queried | 6.89 | 4.11e-05 | 2.11e-01 |
|  | 2 | 128458240 | 128458845 | PDFSTATSENSEMBLUCSC | 606 | 8 | 24662 | island | 0.89523 | 0.04643 | Not queried | 6.89 | 1.04e-02 | 1.00e+00 |
|  | 2 | 11051728 | 11052102 | PDFSTATSENSEMBLUCSC | 375 | 7 | 24036 | island | 0.02830 | 0.88806 | Not queried | 6.89 | 5.83e-04 | 1.00e+00 |
|  | 16 | 2902830 | 2903392 | PDFSTATSENSEMBLUCSC | 563 | 5 | 14586 | gene;island | 0.06340 | 0.75620 | Not queried | 6.88 | 7.94e-03 | 1.00e+00 |
|  | 3 | 169530268 | 169530621 | PDFSTATSENSEMBLUCSC | 354 | 6 | 29606 | island | 0.02610 | 0.80172 | Not queried | 6.87 | 2.16e-03 | 1.00e+00 |
|  | 7 | 12151221 | 12151638 | PDFSTATSENSEMBLUCSC | 418 | 4 | 38200 | island | 0.06686 | 0.83198 | Not queried | 6.86 | 2.86e-02 | 1.00e+00 |
|  | 17 | 21030030 | 21030370 | PDFSTATSENSEMBLUCSC | 341 | 8 | 18082 | island | 0.01579 | 0.66413 | Not queried | 6.86 | 1.55e-04 | 7.99e-01 |
|  | 5 | 140254793 | 140255069 | PDFSTATSENSEMBLUCSC | 277 | 4 | 31745 | gene;island | 0.06048 | 0.91864 | Not queried | 6.86 | 2.86e-02 | 1.00e+00 |
|  | 15 | 28016437 | 28016654 | PDFSTATSENSEMBLUCSC | 218 | 4 | 13234 | gene;island | 0.08077 | 0.81648 | Not queried | 6.85 | 2.86e-02 | 1.00e+00 |
|  | 6 | 44265644 | 44265696 | PDFSTATSENSEMBLUCSC | 53 | 4 | 34947 | tss;island | 0.18847 | 0.97050 | Not queried | 6.84 | 2.86e-02 | 1.00e+00 |
|  | 17 | 21030030 | 21030248 | PDFSTATSENSEMBLUCSC | 219 | 7 | 17167 | tss | 0.01598 | 0.65584 | Not queried | 6.84 | 5.83e-04 | 1.00e+00 |
|  | 6 | 79576851 | 79577177 | PDFSTATSENSEMBLUCSC | 327 | 8 | 35015 | tss | 0.03229 | 0.70641 | Not queried | 6.83 | 1.55e-04 | 7.99e-01 |
|  | 10 | 38692145 | 38692317 | PDFSTATSENSEMBLUCSC | 173 | 4 | 5021 | tss;island | 0.03423 | 0.58912 | Not queried | 6.83 | 5.71e-02 | 1.00e+00 |
|  | 17 | 79031376 | 79031712 | PDFSTATSENSEMBLUCSC | 337 | 5 | 16857 | gene | 0.03776 | 0.85479 | Not queried | 6.82 | 7.94e-03 | 1.00e+00 |
|  | 11 | 393938 | 394289 | PDFSTATSENSEMBLUCSC | 352 | 5 | 7825 | island | 0.04189 | 0.78778 | Not queried | 6.82 | 7.94e-03 | 1.00e+00 |
|  | 16 | 67906784 | 67906986 | PDFSTATSENSEMBLUCSC | 203 | 5 | 15283 | tss | 0.01766 | 0.65219 | Not queried | 6.81 | 7.94e-03 | 1.00e+00 |
|  | 4 | 75719957 | 75720078 | PDFSTATSENSEMBLUCSC | 122 | 5 | 30389 | tss;island | 0.02845 | 0.76156 | Not queried | 6.81 | 7.94e-03 | 1.00e+00 |
|  | 6 | 32977400 | 32977708 | PDFSTATSENSEMBLUCSC | 309 | 4 | 34822 | tss | 0.48113 | 0.01932 | Not queried | 6.81 | 1.14e-01 | 1.00e+00 |
|  | 8 | 82633568 | 82633764 | PDFSTATSENSEMBLUCSC | 197 | 7 | 39721 | tss | 0.01386 | 0.48673 | Not queried | 6.81 | 5.83e-04 | 1.00e+00 |
|  | X | 21776242 | 21776494 | PDFSTATSENSEMBLUCSC | 253 | 6 | 41221 | tss | 0.10051 | 0.90202 | Not queried | 6.79 | 2.16e-03 | 1.00e+00 |
|  | 2 | 208635549 | 208636363 | PDFSTATSENSEMBLUCSC | 815 | 10 | 25005 | island | 0.85574 | 0.04958 | Not queried | 6.79 | 4.87e-04 | 1.00e+00 |
|  | 10 | 38645346 | 38645450 | PDFSTATSENSEMBLUCSC | 105 | 4 | 4547 | gene;island | 0.03040 | 0.64559 | Not queried | 6.79 | 2.86e-02 | 1.00e+00 |
|  | 1 | 209848306 | 209848577 | PDFSTATSENSEMBLUCSC | 272 | 5 | 2227 | tss;island | 0.87335 | 0.05872 | Not queried | 6.79 | 7.94e-03 | 1.00e+00 |
|  | 6 | 88031886 | 88032455 | PDFSTATSENSEMBLUCSC | 570 | 9 | 34344 | gene;island | 0.03509 | 0.83561 | Not queried | 6.78 | 4.11e-05 | 2.11e-01 |
|  | 6 | 88031886 | 88032210 | PDFSTATSENSEMBLUCSC | 325 | 7 | 35044 | tss | 0.03456 | 0.83561 | Not queried | 6.78 | 5.83e-04 | 1.00e+00 |
|  | 11 | 67442067 | 67442408 | PDFSTATSENSEMBLUCSC | 342 | 6 | 6746 | gene | 0.02979 | 0.71849 | Not queried | 6.78 | 2.16e-03 | 1.00e+00 |
|  | 3 | 169530417 | 169530621 | PDFSTATSENSEMBLUCSC | 205 | 5 | 28666 | tss | 0.02835 | 0.77760 | Not queried | 6.78 | 7.94e-03 | 1.00e+00 |
|  | 6 | 79577539 | 79577801 | PDFSTATSENSEMBLUCSC | 263 | 4 | 34332 | gene;island | 0.01672 | 0.62111 | Not queried | 6.77 | 5.71e-02 | 1.00e+00 |
|  | X | 119134323 | 119134684 | PDFSTATSENSEMBLUCSC | 362 | 4 | 41995 | island | 0.95402 | 0.16257 | Not queried | 6.76 | 2.86e-02 | 1.00e+00 |
|  | 19 | 15342749 | 15343425 | PDFSTATSENSEMBLUCSC | 677 | 11 | 19867 | gene;island | 0.75907 | 0.04659 | EPHX3 (margin: AC004257.1; EPHX3; AC004257.3; BRD4) | 6.76 | 2.84e-06 | 1.46e-02 |
|  | 1 | 74663583 | 74663827 | PDFSTATSENSEMBLUCSC | 245 | 5 | 582 | gene | 0.01411 | 0.53298 | Not queried | 6.75 | 7.94e-03 | 1.00e+00 |
|  | 20 | 22559305 | 22559456 | PDFSTATSENSEMBLUCSC | 152 | 4 | 25593 | tss;island | 0.02419 | 0.69319 | Not queried | 6.74 | 2.86e-02 | 1.00e+00 |
|  | 15 | 31372742 | 31373178 | PDFSTATSENSEMBLUCSC | 437 | 4 | 13243 | gene | 0.87098 | 0.07111 | Not queried | 6.74 | 5.71e-02 | 1.00e+00 |
|  | 1 | 68512539 | 68513063 | PDFSTATSENSEMBLUCSC | 525 | 8 | 572 | gene;island | 0.05520 | 0.88016 | Not queried | 6.74 | 1.55e-04 | 7.99e-01 |
|  | X | 154299274 | 154299395 | PDFSTATSENSEMBLUCSC | 122 | 4 | 41166 | gene;tss;island | 0.01371 | 0.60990 | Not queried | 6.73 | 2.86e-02 | 1.00e+00 |
|  | 19 | 52900882 | 52901307 | PDFSTATSENSEMBLUCSC | 426 | 11 | 22322 | island | 0.02292 | 0.83850 | ZNF528; CTD-3018O17.3 (margin: ZNF880; ZNF528; CTD-3018O17.3) | 6.73 | 2.84e-06 | 1.46e-02 |
|  | 19 | 1132256 | 1132537 | PDFSTATSENSEMBLUCSC | 282 | 8 | 19609 | gene | 0.02886 | 0.80334 | Not queried | 6.73 | 1.55e-04 | 7.99e-01 |
|  | 12 | 3862221 | 3862597 | PDFSTATSENSEMBLUCSC | 377 | 12 | 10140 | island | 0.04487 | 0.81025 | EFCAB4B (margin: EFCAB4B) | 6.73 | 7.40e-07 | 3.80e-03 |
|  | 2 | 176981328 | 176981469 | PDFSTATSENSEMBLUCSC | 142 | 4 | 23697 | tss | 0.01257 | 0.56786 | Not queried | 6.72 | 2.86e-02 | 1.00e+00 |
|  | 10 | 93392870 | 93393509 | PDFSTATSENSEMBLUCSC | 640 | 10 | 5206 | tss | 0.03171 | 0.72871 | Not queried | 6.71 | 1.08e-05 | 5.56e-02 |
|  | 7 | 157809235 | 157809596 | PDFSTATSENSEMBLUCSC | 362 | 6 | 37293 | gene | 0.77168 | 0.04734 | Not queried | 6.70 | 2.16e-03 | 1.00e+00 |
|  | 19 | 37742739 | 37742956 | PDFSTATSENSEMBLUCSC | 218 | 4 | 21916 | island | 0.76963 | 0.03306 | Not queried | 6.70 | 2.86e-02 | 1.00e+00 |
|  | 6 | 79576851 | 79577260 | PDFSTATSENSEMBLUCSC | 410 | 11 | 36209 | island | 0.04090 | 0.71060 | IRAK1BP1 (margin: IRAK1BP1) | 6.70 | 2.84e-06 | 1.46e-02 |
|  | 17 | 48172598 | 48172668 | PDFSTATSENSEMBLUCSC | 71 | 4 | 17459 | tss;island | 0.02749 | 0.69301 | Not queried | 6.70 | 2.86e-02 | 1.00e+00 |
|  | 12 | 7858592 | 7858848 | PDFSTATSENSEMBLUCSC | 257 | 6 | 10202 | island | 0.09536 | 0.90491 | Not queried | 6.70 | 2.16e-03 | 1.00e+00 |
|  | X | 134555540 | 134555839 | PDFSTATSENSEMBLUCSC | 300 | 9 | 41542 | tss;island | 0.01719 | 0.60411 | Not queried | 6.69 | 4.11e-05 | 2.11e-01 |
|  | Y | 2655740 | 2655977 | PDFSTATSENSEMBLUCSC | 238 | 5 | 42175 | island | 0.01549 | 0.30053 | Not queried | 6.68 | 4.21e-01 | 1.00e+00 |
|  | X | 134478461 | 134479169 | PDFSTATSENSEMBLUCSC | 709 | 10 | 41541 | tss;island | 0.01536 | 0.60682 | Not queried | 6.68 | 1.08e-05 | 5.56e-02 |
|  | 15 | 25018132 | 25018449 | PDFSTATSENSEMBLUCSC | 318 | 4 | 13923 | island | 0.13870 | 0.93237 | Not queried | 6.67 | 2.86e-02 | 1.00e+00 |
|  | 2 | 66667039 | 66667101 | PDFSTATSENSEMBLUCSC | 63 | 5 | 22674 | gene | 0.64999 | 0.02538 | Not queried | 6.67 | 7.94e-03 | 1.00e+00 |
|  | 7 | 27204663 | 27205114 | PDFSTATSENSEMBLUCSC | 452 | 7 | 36875 | gene | 0.04069 | 0.74520 | Not queried | 6.67 | 5.83e-04 | 1.00e+00 |
|  | X | 153059760 | 153059963 | PDFSTATSENSEMBLUCSC | 204 | 4 | 41147 | gene | 0.00796 | 0.44839 | Not queried | 6.65 | 2.86e-02 | 1.00e+00 |
|  | 8 | 18541446 | 18541627 | PDFSTATSENSEMBLUCSC | 182 | 5 | 39156 | gene | 0.04223 | 0.81606 | Not queried | 6.65 | 7.94e-03 | 1.00e+00 |
|  | 5 | 140207460 | 140208201 | PDFSTATSENSEMBLUCSC | 742 | 9 | 31740 | gene;island | 0.08208 | 0.85401 | Not queried | 6.65 | 4.11e-05 | 2.11e-01 |
|  | X | 92928508 | 92928730 | PDFSTATSENSEMBLUCSC | 223 | 8 | 41413 | tss;island | 0.01513 | 0.56524 | Not queried | 6.65 | 1.55e-04 | 7.99e-01 |
|  | 13 | 103426286 | 103426481 | PDFSTATSENSEMBLUCSC | 196 | 7 | 11495 | tss;island | 0.01398 | 0.58667 | Not queried | 6.64 | 1.17e-03 | 1.00e+00 |
|  | 15 | 39871808 | 39872186 | PDFSTATSENSEMBLUCSC | 379 | 7 | 13547 | tss;island | 0.04241 | 0.85604 | Not queried | 6.63 | 1.17e-03 | 1.00e+00 |
|  | 5 | 140213783 | 140213924 | PDFSTATSENSEMBLUCSC | 142 | 5 | 31741 | gene;tss;island | 0.02658 | 0.91968 | Not queried | 6.63 | 7.94e-03 | 1.00e+00 |
|  | 19 | 12075601 | 12076348 | PDFSTATSENSEMBLUCSC | 748 | 11 | 21552 | island | 0.03051 | 0.67328 | ZNF763 (margin: ZNF763; CTD-2006C1.12) | 6.63 | 2.84e-06 | 1.46e-02 |
|  | X | 119005413 | 119006122 | PDFSTATSENSEMBLUCSC | 710 | 14 | 41095 | gene;tss;island | 0.01651 | 0.62465 | NDUFA1; RNF113A (margin: NDUFA1; RNF113A) | 6.62 | 4.99e-08 | 2.56e-04 |
|  | 2 | 176981328 | 176981654 | PDFSTATSENSEMBLUCSC | 327 | 5 | 24860 | island | 0.01516 | 0.60302 | Not queried | 6.62 | 9.52e-02 | 1.00e+00 |
|  | 7 | 56515510 | 56515846 | PDFSTATSENSEMBLUCSC | 337 | 5 | 36959 | gene;island | 0.02224 | 0.69929 | Not queried | 6.62 | 7.94e-03 | 1.00e+00 |
|  | 19 | 15344046 | 15344364 | PDFSTATSENSEMBLUCSC | 319 | 4 | 20522 | tss;island | 0.75708 | 0.04558 | Not queried | 6.61 | 2.86e-02 | 1.00e+00 |
|  | 17 | 75315486 | 75315868 | PDFSTATSENSEMBLUCSC | 383 | 8 | 16768 | gene | 0.03709 | 0.79106 | Not queried | 6.61 | 1.55e-04 | 7.99e-01 |
|  | 12 | 29302016 | 29302279 | PDFSTATSENSEMBLUCSC | 264 | 5 | 10278 | island | 0.01646 | 0.59787 | Not queried | 6.61 | 7.94e-03 | 1.00e+00 |
|  | 11 | 117800233 | 117800571 | PDFSTATSENSEMBLUCSC | 339 | 5 | 7702 | tss | 0.05404 | 0.86424 | Not queried | 6.60 | 7.94e-03 | 1.00e+00 |
|  | 19 | 50194120 | 50194252 | PDFSTATSENSEMBLUCSC | 133 | 5 | 20925 | tss;island | 0.82439 | 0.05214 | Not queried | 6.59 | 7.94e-03 | 1.00e+00 |
|  | 1 | 91185028 | 91185441 | PDFSTATSENSEMBLUCSC | 414 | 6 | 3453 | island | 0.80804 | 0.04225 | Not queried | 6.59 | 2.16e-03 | 1.00e+00 |
|  | X | 135579980 | 135580181 | PDFSTATSENSEMBLUCSC | 202 | 4 | 41120 | gene;island | 0.01592 | 0.57619 | Not queried | 6.58 | 2.86e-02 | 1.00e+00 |
|  | 17 | 75315486 | 75315567 | PDFSTATSENSEMBLUCSC | 82 | 5 | 17663 | tss | 0.03891 | 0.78937 | Not queried | 6.56 | 7.94e-03 | 1.00e+00 |
|  | 14 | 105714833 | 105715025 | PDFSTATSENSEMBLUCSC | 193 | 4 | 12231 | gene;tss;island | 0.07014 | 0.84429 | Not queried | 6.56 | 2.86e-02 | 1.00e+00 |
|  | 19 | 1132294 | 1132537 | PDFSTATSENSEMBLUCSC | 244 | 7 | 20256 | tss | 0.02918 | 0.79081 | Not queried | 6.55 | 5.83e-04 | 1.00e+00 |
|  | 14 | 75593334 | 75594058 | PDFSTATSENSEMBLUCSC | 725 | 14 | 12984 | island | 0.69509 | 0.02418 | RP11-950C14.7; NEK9 (margin: RP11-950C14.7; NEK9; HIF1AP1; TMED10) | 6.55 | 4.99e-08 | 2.56e-04 |
|  | 13 | 41768693 | 41768978 | PDFSTATSENSEMBLUCSC | 286 | 6 | 11663 | island | 0.05191 | 0.72537 | Not queried | 6.55 | 2.16e-03 | 1.00e+00 |
|  | 12 | 51236582 | 51236768 | PDFSTATSENSEMBLUCSC | 187 | 6 | 10392 | island | 0.09338 | 0.91108 | Not queried | 6.55 | 2.16e-03 | 1.00e+00 |
|  | 17 | 19437691 | 19437929 | PDFSTATSENSEMBLUCSC | 239 | 4 | 16445 | gene;island | 0.77999 | 0.08404 | Not queried | 6.54 | 2.86e-02 | 1.00e+00 |
|  | 8 | 114389168 | 114389479 | PDFSTATSENSEMBLUCSC | 312 | 6 | 39338 | gene | 0.08705 | 0.87580 | Not queried | 6.54 | 2.16e-03 | 1.00e+00 |
|  | 1 | 165567312 | 165567529 | PDFSTATSENSEMBLUCSC | 218 | 4 | 3917 | island | 0.02010 | 0.58561 | Not queried | 6.53 | 2.86e-02 | 1.00e+00 |
|  | 1 | 29138919 | 29139278 | PDFSTATSENSEMBLUCSC | 360 | 5 | 370 | gene;island | 0.01203 | 0.51739 | Not queried | 6.53 | 7.94e-03 | 1.00e+00 |
|  | X | 134477802 | 134478126 | PDFSTATSENSEMBLUCSC | 325 | 5 | 41540 | tss;island | 0.01845 | 0.71486 | Not queried | 6.53 | 7.94e-03 | 1.00e+00 |
|  | 10 | 115860606 | 115860959 | PDFSTATSENSEMBLUCSC | 354 | 4 | 6130 | island | 0.01864 | 0.69297 | Not queried | 6.53 | 2.86e-02 | 1.00e+00 |
|  | 6 | 32977138 | 32977708 | PDFSTATSENSEMBLUCSC | 571 | 9 | 35893 | island | 0.73315 | 0.05162 | Not queried | 6.51 | 1.85e-03 | 1.00e+00 |
|  | 2 | 61371880 | 61372226 | PDFSTATSENSEMBLUCSC | 347 | 7 | 23339 | tss | 0.07409 | 0.86546 | Not queried | 6.51 | 5.83e-04 | 1.00e+00 |
|  | 16 | 67906784 | 67907373 | PDFSTATSENSEMBLUCSC | 590 | 9 | 15999 | island | 0.01766 | 0.57928 | Not queried | 6.51 | 4.11e-05 | 2.11e-01 |
|  | 3 | 49236800 | 49236946 | PDFSTATSENSEMBLUCSC | 147 | 6 | 27736 | gene;island | 0.11060 | 0.89288 | Not queried | 6.50 | 2.16e-03 | 1.00e+00 |
|  | 13 | 48895173 | 48895478 | PDFSTATSENSEMBLUCSC | 306 | 4 | 11133 | gene;island | 0.04537 | 0.81251 | Not queried | 6.50 | 2.86e-02 | 1.00e+00 |
|  | 3 | 147142102 | 147142415 | PDFSTATSENSEMBLUCSC | 314 | 5 | 29533 | island | 0.82789 | 0.05037 | Not queried | 6.50 | 7.94e-03 | 1.00e+00 |
|  | 4 | 52917271 | 52917660 | PDFSTATSENSEMBLUCSC | 390 | 10 | 30988 | island | 0.91120 | 0.11379 | Not queried | 6.50 | 1.08e-05 | 5.56e-02 |
|  | 1 | 209979111 | 209979779 | PDFSTATSENSEMBLUCSC | 669 | 13 | 4154 | island | 0.02320 | 0.66796 | IRF6 (margin: IRF6) | 6.49 | 1.92e-07 | 9.88e-04 |
|  | 7 | 99517136 | 99517509 | PDFSTATSENSEMBLUCSC | 374 | 12 | 38677 | island | 0.80869 | 0.03291 | TRIM4 (margin: RP4-604G5.1; TRIM4; GJC3) | 6.49 | 7.40e-07 | 3.80e-03 |
|  | 7 | 99517192 | 99517509 | PDFSTATSENSEMBLUCSC | 318 | 10 | 37697 | tss | 0.82922 | 0.04160 | Not queried | 6.49 | 1.08e-05 | 5.56e-02 |
|  | 2 | 186603398 | 186603639 | PDFSTATSENSEMBLUCSC | 242 | 4 | 24908 | island | 0.00422 | 0.26337 | Not queried | 6.49 | 2.86e-02 | 1.00e+00 |
|  | 2 | 11051728 | 11051991 | PDFSTATSENSEMBLUCSC | 264 | 5 | 23148 | tss | 0.06255 | 0.88806 | Not queried | 6.49 | 7.94e-03 | 1.00e+00 |
|  | 1 | 11752109 | 11752494 | PDFSTATSENSEMBLUCSC | 386 | 4 | 225 | gene;tss;island | 0.69754 | 0.01792 | Not queried | 6.48 | 2.86e-02 | 1.00e+00 |
|  | 19 | 52900882 | 52900988 | PDFSTATSENSEMBLUCSC | 107 | 8 | 20977 | tss | 0.04823 | 0.84052 | Not queried | 6.47 | 1.55e-04 | 7.99e-01 |
|  | 14 | 23937965 | 23938207 | PDFSTATSENSEMBLUCSC | 243 | 4 | 12276 | tss | 0.12323 | 0.89202 | Not queried | 6.47 | 2.86e-02 | 1.00e+00 |
|  | 17 | 72352688 | 72352968 | PDFSTATSENSEMBLUCSC | 281 | 4 | 16717 | gene;island | 0.07471 | 0.76928 | Not queried | 6.46 | 2.86e-02 | 1.00e+00 |
|  | X | 50024233 | 50024550 | PDFSTATSENSEMBLUCSC | 318 | 4 | 41795 | island | 0.32943 | 0.95726 | Not queried | 6.46 | 2.86e-02 | 1.00e+00 |
|  | 16 | 4311628 | 4311941 | PDFSTATSENSEMBLUCSC | 314 | 5 | 14606 | gene;island | 0.06305 | 0.74432 | Not queried | 6.46 | 7.94e-03 | 1.00e+00 |
|  | 19 | 44324856 | 44325008 | PDFSTATSENSEMBLUCSC | 153 | 8 | 20799 | tss | 0.01834 | 0.65284 | Not queried | 6.45 | 1.55e-04 | 7.99e-01 |
|  | 20 | 61446962 | 61448325 | PDFSTATSENSEMBLUCSC | 1364 | 42 | 25843 | tss;island | 0.04147 | 0.74284 | OGFR; COL9A3 (margin: OGFR; COL9A3; OGFR-AS1) | 6.45 | 1.19e-24 | 6.12e-21 |
|  | X | 79590789 | 79590966 | PDFSTATSENSEMBLUCSC | 178 | 7 | 41404 | tss | 0.01492 | 0.52131 | Not queried | 6.45 | 5.83e-04 | 1.00e+00 |
|  | 21 | 38593636 | 38593754 | PDFSTATSENSEMBLUCSC | 119 | 4 | 26637 | island | 0.01831 | 0.61807 | Not queried | 6.44 | 2.86e-02 | 1.00e+00 |
|  | 17 | 46682390 | 46682413 | PDFSTATSENSEMBLUCSC | 24 | 4 | 17432 | tss | 0.06695 | 0.87101 | Not queried | 6.44 | 2.86e-02 | 1.00e+00 |
|  | 14 | 103674159 | 103674342 | PDFSTATSENSEMBLUCSC | 184 | 4 | 13155 | island | 0.83611 | 0.10448 | Not queried | 6.43 | 2.86e-02 | 1.00e+00 |
|  | 12 | 98850691 | 98851021 | PDFSTATSENSEMBLUCSC | 331 | 5 | 10676 | island | 0.16316 | 0.88762 | Not queried | 6.43 | 7.94e-03 | 1.00e+00 |
|  | 1 | 86174060 | 86174240 | PDFSTATSENSEMBLUCSC | 181 | 7 | 3421 | island | 0.01027 | 0.53554 | Not queried | 6.42 | 5.83e-04 | 1.00e+00 |
|  | 13 | 46742691 | 46742802 | PDFSTATSENSEMBLUCSC | 112 | 4 | 11128 | gene | 0.01522 | 0.56788 | Not queried | 6.42 | 2.86e-02 | 1.00e+00 |
|  | 6 | 24357720 | 24357985 | PDFSTATSENSEMBLUCSC | 266 | 4 | 33525 | gene;island | 0.05582 | 0.86875 | Not queried | 6.41 | 2.86e-02 | 1.00e+00 |
|  | 10 | 93392644 | 93393509 | PDFSTATSENSEMBLUCSC | 866 | 14 | 5913 | island | 0.03365 | 0.75510 | PPP1R3C (margin: PPP1R3C) | 6.41 | 4.99e-08 | 2.56e-04 |
|  | 10 | 49812686 | 49813434 | PDFSTATSENSEMBLUCSC | 749 | 13 | 5708 | island | 0.03878 | 0.81259 | ARHGAP22 (margin: ARHGAP22) | 6.41 | 1.92e-07 | 9.88e-04 |
|  | 9 | 89763370 | 89763570 | PDFSTATSENSEMBLUCSC | 201 | 4 | 40836 | island | 0.09329 | 0.91583 | Not queried | 6.41 | 2.86e-02 | 1.00e+00 |
|  | 19 | 44324747 | 44325008 | PDFSTATSENSEMBLUCSC | 262 | 9 | 22061 | island | 0.01949 | 0.66345 | Not queried | 6.40 | 4.11e-05 | 2.11e-01 |
|  | 6 | 27647713 | 27648057 | PDFSTATSENSEMBLUCSC | 345 | 5 | 35497 | island | 0.00566 | 0.29604 | Not queried | 6.40 | 7.94e-03 | 1.00e+00 |
|  | 11 | 67442126 | 67442408 | PDFSTATSENSEMBLUCSC | 283 | 4 | 7480 | tss | 0.02947 | 0.69940 | Not queried | 6.39 | 2.86e-02 | 1.00e+00 |
|  | 10 | 94825547 | 94825848 | PDFSTATSENSEMBLUCSC | 302 | 4 | 4665 | gene;island | 0.10428 | 0.88325 | Not queried | 6.39 | 2.86e-02 | 1.00e+00 |
|  | X | 154299647 | 154299853 | PDFSTATSENSEMBLUCSC | 207 | 5 | 41612 | tss;island | 0.01874 | 0.57887 | Not queried | 6.39 | 7.94e-03 | 1.00e+00 |
|  | 15 | 91427821 | 91428056 | PDFSTATSENSEMBLUCSC | 236 | 5 | 13449 | gene;tss;island | 0.06320 | 0.85316 | Not queried | 6.39 | 7.94e-03 | 1.00e+00 |
|  | 8 | 82633454 | 82633764 | PDFSTATSENSEMBLUCSC | 311 | 8 | 40356 | island | 0.01710 | 0.50073 | Not queried | 6.38 | 1.55e-04 | 7.99e-01 |
|  | 2 | 219866424 | 219866628 | PDFSTATSENSEMBLUCSC | 205 | 6 | 25064 | island | 0.01417 | 0.52500 | Not queried | 6.38 | 2.16e-03 | 1.00e+00 |
|  | 2 | 172952883 | 172953630 | PDFSTATSENSEMBLUCSC | 748 | 8 | 22886 | gene;island | 0.91054 | 0.19184 | Not queried | 6.37 | 1.55e-04 | 7.99e-01 |
|  | 22 | 19929066 | 19929254 | PDFSTATSENSEMBLUCSC | 189 | 5 | 26926 | tss | 0.01684 | 0.57003 | Not queried | 6.37 | 7.94e-03 | 1.00e+00 |
|  | 8 | 1649758 | 1650309 | PDFSTATSENSEMBLUCSC | 552 | 5 | 39110 | gene;island | 0.08054 | 0.93941 | Not queried | 6.36 | 7.94e-03 | 1.00e+00 |
|  | 1 | 43751270 | 43751563 | PDFSTATSENSEMBLUCSC | 294 | 8 | 1555 | tss | 0.02392 | 0.56091 | Not queried | 6.36 | 1.55e-04 | 7.99e-01 |
|  | X | 134478751 | 134479169 | PDFSTATSENSEMBLUCSC | 419 | 5 | 41115 | gene | 0.01346 | 0.52729 | Not queried | 6.35 | 7.94e-03 | 1.00e+00 |
|  | 2 | 43903470 | 43904011 | PDFSTATSENSEMBLUCSC | 542 | 8 | 22623 | gene;tss | 0.09554 | 0.88369 | Not queried | 6.35 | 3.11e-04 | 1.00e+00 |
|  | 20 | 29611652 | 29611924 | PDFSTATSENSEMBLUCSC | 273 | 8 | 26029 | island | 0.02003 | 0.61983 | Not queried | 6.34 | 1.55e-04 | 7.99e-01 |
|  | 1 | 86174125 | 86174240 | PDFSTATSENSEMBLUCSC | 116 | 6 | 1720 | tss | 0.01142 | 0.51742 | Not queried | 6.34 | 2.16e-03 | 1.00e+00 |
|  | X | 77151065 | 77151316 | PDFSTATSENSEMBLUCSC | 252 | 8 | 41400 | tss | 0.02757 | 0.64422 | Not queried | 6.34 | 1.55e-04 | 7.99e-01 |
|  | X | 13835198 | 13835295 | PDFSTATSENSEMBLUCSC | 98 | 4 | 40941 | gene | 0.75524 | 0.03341 | Not queried | 6.33 | 2.86e-02 | 1.00e+00 |
|  | 16 | 57836242 | 57836706 | PDFSTATSENSEMBLUCSC | 465 | 6 | 15936 | island | 0.03709 | 0.68130 | Not queried | 6.33 | 2.16e-03 | 1.00e+00 |
|  | 16 | 20360206 | 20360497 | PDFSTATSENSEMBLUCSC | 292 | 4 | 14653 | gene;island | 0.03436 | 0.67096 | Not queried | 6.33 | 2.86e-02 | 1.00e+00 |
|  | 4 | 85422998 | 85423302 | PDFSTATSENSEMBLUCSC | 305 | 4 | 31109 | island | 0.11847 | 0.87313 | Not queried | 6.33 | 2.86e-02 | 1.00e+00 |
|  | 2 | 214148856 | 214149035 | PDFSTATSENSEMBLUCSC | 180 | 7 | 23821 | tss | 0.01209 | 0.47248 | Not queried | 6.33 | 5.83e-04 | 1.00e+00 |
|  | 10 | 2357224 | 2357587 | PDFSTATSENSEMBLUCSC | 364 | 5 | 5483 | island | 0.06932 | 0.89598 | Not queried | 6.33 | 7.94e-03 | 1.00e+00 |
|  | 2 | 3642710 | 3642967 | PDFSTATSENSEMBLUCSC | 258 | 5 | 22516 | gene | 0.04520 | 0.79070 | Not queried | 6.32 | 7.94e-03 | 1.00e+00 |
|  | 12 | 123215151 | 123215684 | PDFSTATSENSEMBLUCSC | 534 | 9 | 10060 | tss | 0.03539 | 0.73595 | Not queried | 6.32 | 4.11e-05 | 2.11e-01 |
|  | 3 | 57541583 | 57542027 | PDFSTATSENSEMBLUCSC | 445 | 11 | 29184 | island | 0.01697 | 0.56060 | PDE12 (margin: RNU6-483P; PDE12; DNAH12) | 6.32 | 2.84e-06 | 1.46e-02 |
|  | 3 | 57541583 | 57541949 | PDFSTATSENSEMBLUCSC | 367 | 9 | 28366 | tss | 0.01697 | 0.56060 | Not queried | 6.32 | 4.11e-05 | 2.11e-01 |
|  | 3 | 38035463 | 38036060 | PDFSTATSENSEMBLUCSC | 598 | 7 | 27676 | gene;island | 0.06738 | 0.76059 | Not queried | 6.32 | 5.83e-04 | 1.00e+00 |
|  | 18 | 43608309 | 43608548 | PDFSTATSENSEMBLUCSC | 240 | 4 | 19065 | gene;island | 0.90787 | 0.05268 | Not queried | 6.31 | 2.86e-02 | 1.00e+00 |
|  | 7 | 128470217 | 128470614 | PDFSTATSENSEMBLUCSC | 398 | 5 | 38835 | island | 0.05841 | 0.88401 | Not queried | 6.31 | 7.94e-03 | 1.00e+00 |
|  | X | 114524264 | 114524286 | PDFSTATSENSEMBLUCSC | 23 | 4 | 41477 | tss | 0.10952 | 0.92458 | Not queried | 6.30 | 2.86e-02 | 1.00e+00 |
|  | 4 | 85402870 | 85403409 | PDFSTATSENSEMBLUCSC | 540 | 5 | 31105 | island | 0.12134 | 0.93920 | Not queried | 6.29 | 7.94e-03 | 1.00e+00 |
|  | 21 | 39493025 | 39493629 | PDFSTATSENSEMBLUCSC | 605 | 11 | 26504 | tss | 0.03988 | 0.79839 | DSCR8; DSCR4 (margin: DSCR8; DSCR4) | 6.29 | 2.84e-06 | 1.46e-02 |
|  | 6 | 74104388 | 74104600 | PDFSTATSENSEMBLUCSC | 213 | 4 | 34323 | gene;island | 0.02474 | 0.62052 | Not queried | 6.29 | 2.86e-02 | 1.00e+00 |
|  | 6 | 84140785 | 84140997 | PDFSTATSENSEMBLUCSC | 213 | 6 | 35031 | tss | 0.05223 | 0.88075 | Not queried | 6.28 | 2.16e-03 | 1.00e+00 |
|  | X | 153657001 | 153657411 | PDFSTATSENSEMBLUCSC | 411 | 5 | 41159 | gene | 0.02108 | 0.62538 | Not queried | 6.28 | 7.94e-03 | 1.00e+00 |
|  | X | 79590789 | 79591129 | PDFSTATSENSEMBLUCSC | 341 | 10 | 41889 | island | 0.02028 | 0.57700 | Not queried | 6.28 | 1.08e-05 | 5.56e-02 |
|  | 5 | 140753415 | 140753924 | PDFSTATSENSEMBLUCSC | 510 | 10 | 31768 | gene;island | 0.04916 | 0.84635 | Not queried | 6.27 | 2.17e-05 | 1.11e-01 |
|  | 22 | 19929066 | 19929557 | PDFSTATSENSEMBLUCSC | 492 | 9 | 26751 | gene;island | 0.01904 | 0.59052 | Not queried | 6.27 | 4.11e-05 | 2.11e-01 |
|  | 13 | 47012103 | 47012543 | PDFSTATSENSEMBLUCSC | 441 | 4 | 11686 | island | 0.11147 | 0.88343 | Not queried | 6.27 | 2.86e-02 | 1.00e+00 |
|  | 6 | 125284203 | 125284359 | PDFSTATSENSEMBLUCSC | 157 | 6 | 35144 | tss;island | 0.67986 | 0.02681 | Not queried | 6.27 | 2.16e-03 | 1.00e+00 |
|  | 12 | 25102311 | 25102521 | PDFSTATSENSEMBLUCSC | 211 | 4 | 9583 | tss | 0.86091 | 0.06660 | Not queried | 6.26 | 2.86e-02 | 1.00e+00 |
|  | X | 154299737 | 154299853 | PDFSTATSENSEMBLUCSC | 117 | 4 | 41167 | gene | 0.01901 | 0.56749 | Not queried | 6.26 | 2.86e-02 | 1.00e+00 |
|  | 19 | 38755103 | 38755330 | PDFSTATSENSEMBLUCSC | 228 | 5 | 19978 | gene | 0.01491 | 0.56187 | Not queried | 6.26 | 7.94e-03 | 1.00e+00 |
|  | 21 | 39493025 | 39493391 | PDFSTATSENSEMBLUCSC | 367 | 5 | 26384 | gene | 0.03988 | 0.79839 | Not queried | 6.26 | 7.94e-03 | 1.00e+00 |
|  | 21 | 45789122 | 45789379 | PDFSTATSENSEMBLUCSC | 258 | 4 | 26414 | gene;island | 0.09428 | 0.82133 | Not queried | 6.25 | 2.86e-02 | 1.00e+00 |
|  | 20 | 29611652 | 29611810 | PDFSTATSENSEMBLUCSC | 159 | 5 | 25617 | tss | 0.01882 | 0.61314 | Not queried | 6.25 | 7.94e-03 | 1.00e+00 |
|  | 5 | 137610127 | 137610645 | PDFSTATSENSEMBLUCSC | 519 | 9 | 33092 | island | 0.01411 | 0.49104 | Not queried | 6.25 | 8.23e-05 | 4.23e-01 |
|  | 22 | 28010359 | 28010598 | PDFSTATSENSEMBLUCSC | 240 | 4 | 27337 | island | 0.08407 | 0.87020 | Not queried | 6.24 | 2.86e-02 | 1.00e+00 |
|  | 19 | 50979561 | 50979755 | PDFSTATSENSEMBLUCSC | 195 | 6 | 20140 | gene;island | 0.03604 | 0.81659 | Not queried | 6.24 | 2.16e-03 | 1.00e+00 |
|  | 6 | 31096861 | 31097078 | PDFSTATSENSEMBLUCSC | 218 | 5 | 33790 | gene | 0.04149 | 0.76612 | Not queried | 6.24 | 3.17e-02 | 1.00e+00 |
|  | 2 | 27531163 | 27531535 | PDFSTATSENSEMBLUCSC | 373 | 8 | 23225 | tss | 0.05425 | 0.75659 | Not queried | 6.24 | 1.55e-04 | 7.99e-01 |
|  | 1 | 207818395 | 207818449 | PDFSTATSENSEMBLUCSC | 55 | 4 | 2220 | tss | 0.01361 | 0.47717 | Not queried | 6.24 | 2.86e-02 | 1.00e+00 |
|  | 19 | 50194120 | 50194228 | PDFSTATSENSEMBLUCSC | 109 | 4 | 20125 | gene | 0.78840 | 0.05997 | Not queried | 6.23 | 2.86e-02 | 1.00e+00 |
|  | X | 134186228 | 134186640 | PDFSTATSENSEMBLUCSC | 413 | 10 | 41538 | tss | 0.01861 | 0.54315 | Not queried | 6.23 | 1.08e-05 | 5.56e-02 |
|  | 9 | 841340 | 841636 | PDFSTATSENSEMBLUCSC | 297 | 5 | 40736 | tss;island | 0.02696 | 0.70565 | Not queried | 6.23 | 7.94e-03 | 1.00e+00 |
|  | 2 | 219866437 | 219866628 | PDFSTATSENSEMBLUCSC | 192 | 5 | 23850 | tss | 0.01611 | 0.54949 | Not queried | 6.23 | 7.94e-03 | 1.00e+00 |
|  | 4 | 79472605 | 79472939 | PDFSTATSENSEMBLUCSC | 335 | 5 | 31075 | island | 0.82870 | 0.04403 | Not queried | 6.22 | 7.94e-03 | 1.00e+00 |
|  | 3 | 44770851 | 44771113 | PDFSTATSENSEMBLUCSC | 263 | 9 | 28219 | tss | 0.58714 | 0.01584 | Not queried | 6.22 | 4.11e-05 | 2.11e-01 |
|  | 6 | 29599012 | 29599390 | PDFSTATSENSEMBLUCSC | 379 | 10 | 33618 | gene;island | 0.63939 | 0.03038 | Not queried | 6.22 | 1.08e-05 | 5.56e-02 |
|  | 1 | 12655992 | 12656315 | PDFSTATSENSEMBLUCSC | 324 | 4 | 233 | gene;island | 0.86837 | 0.06318 | Not queried | 6.22 | 2.86e-02 | 1.00e+00 |
|  | 4 | 15704599 | 15704844 | PDFSTATSENSEMBLUCSC | 246 | 4 | 29914 | gene | 0.11117 | 0.90815 | Not queried | 6.22 | 2.86e-02 | 1.00e+00 |
|  | 10 | 128077307 | 128077376 | PDFSTATSENSEMBLUCSC | 70 | 6 | 5415 | tss;island | 0.83355 | 0.08434 | Not queried | 6.22 | 2.16e-03 | 1.00e+00 |
|  | 14 | 104551867 | 104552034 | PDFSTATSENSEMBLUCSC | 168 | 4 | 12628 | tss | 0.17507 | 0.93144 | Not queried | 6.21 | 2.86e-02 | 1.00e+00 |
|  | 19 | 49713645 | 49714044 | PDFSTATSENSEMBLUCSC | 400 | 4 | 20111 | gene;island | 0.10181 | 0.86379 | Not queried | 6.21 | 2.86e-02 | 1.00e+00 |
|  | 19 | 23653837 | 23654274 | PDFSTATSENSEMBLUCSC | 438 | 5 | 21812 | island | 0.10525 | 0.89238 | Not queried | 6.20 | 7.94e-03 | 1.00e+00 |
|  | 1 | 38412519 | 38412711 | PDFSTATSENSEMBLUCSC | 193 | 4 | 438 | gene | 0.67862 | 0.02911 | Not queried | 6.20 | 2.86e-02 | 1.00e+00 |
|  | 3 | 129693370 | 129693791 | PDFSTATSENSEMBLUCSC | 422 | 6 | 27902 | gene;island | 0.87825 | 0.10174 | Not queried | 6.19 | 2.16e-03 | 1.00e+00 |
|  | X | 25033767 | 25034037 | PDFSTATSENSEMBLUCSC | 271 | 4 | 40953 | gene | 0.75015 | 0.03966 | Not queried | 6.19 | 2.86e-02 | 1.00e+00 |
|  | 19 | 1467921 | 1467979 | PDFSTATSENSEMBLUCSC | 59 | 4 | 19621 | gene;island | 0.03197 | 0.74112 | Not queried | 6.18 | 2.86e-02 | 1.00e+00 |
|  | 16 | 75150744 | 75150880 | PDFSTATSENSEMBLUCSC | 137 | 6 | 15328 | tss | 0.78758 | 0.06221 | Not queried | 6.18 | 2.16e-03 | 1.00e+00 |
|  | 5 | 178986131 | 178986412 | PDFSTATSENSEMBLUCSC | 282 | 4 | 31873 | gene;tss;island | 0.49461 | 0.01871 | Not queried | 6.18 | 2.86e-02 | 1.00e+00 |
|  | 5 | 94982161 | 94982715 | PDFSTATSENSEMBLUCSC | 555 | 11 | 32907 | island | 0.02558 | 0.66273 | RFESD (margin: RFESD; SPATA9) | 6.18 | 2.84e-06 | 1.46e-02 |
|  | 5 | 94982161 | 94982558 | PDFSTATSENSEMBLUCSC | 398 | 9 | 32126 | tss | 0.02727 | 0.66651 | Not queried | 6.18 | 4.11e-05 | 2.11e-01 |
|  | 13 | 110521956 | 110522297 | PDFSTATSENSEMBLUCSC | 342 | 5 | 11844 | island | 0.02726 | 0.80883 | Not queried | 6.17 | 7.94e-03 | 1.00e+00 |
|  | 10 | 49813142 | 49813434 | PDFSTATSENSEMBLUCSC | 293 | 9 | 5050 | tss | 0.05145 | 0.79903 | Not queried | 6.17 | 4.11e-05 | 2.11e-01 |
|  | 4 | 74847758 | 74848016 | PDFSTATSENSEMBLUCSC | 259 | 6 | 30384 | tss | 0.08150 | 0.84832 | Not queried | 6.17 | 2.16e-03 | 1.00e+00 |
|  | 11 | 78673073 | 78673290 | PDFSTATSENSEMBLUCSC | 218 | 4 | 6828 | gene;island | 0.75302 | 0.03105 | Not queried | 6.17 | 2.86e-02 | 1.00e+00 |
|  | 13 | 50707427 | 50708043 | PDFSTATSENSEMBLUCSC | 617 | 5 | 11715 | island | 0.74653 | 0.03943 | Not queried | 6.17 | 7.94e-03 | 1.00e+00 |
|  | 10 | 94451351 | 94451736 | PDFSTATSENSEMBLUCSC | 386 | 4 | 4662 | gene;island | 0.77371 | 0.04152 | Not queried | 6.17 | 2.86e-02 | 1.00e+00 |
|  | 12 | 95942761 | 95943114 | PDFSTATSENSEMBLUCSC | 354 | 5 | 9228 | gene;tss;island | 0.67512 | 0.06442 | Not queried | 6.17 | 7.94e-03 | 1.00e+00 |
|  | X | 73438299 | 73438541 | PDFSTATSENSEMBLUCSC | 243 | 4 | 41036 | gene;tss | 0.24541 | 0.91871 | Not queried | 6.16 | 2.86e-02 | 1.00e+00 |
|  | X | 114546190 | 114546624 | PDFSTATSENSEMBLUCSC | 435 | 4 | 41971 | island | 0.81043 | 0.09166 | Not queried | 6.16 | 2.86e-02 | 1.00e+00 |
|  | X | 77394971 | 77395543 | PDFSTATSENSEMBLUCSC | 573 | 8 | 41888 | island | 0.01955 | 0.56855 | Not queried | 6.16 | 3.11e-04 | 1.00e+00 |
|  | 2 | 27531124 | 27531535 | PDFSTATSENSEMBLUCSC | 412 | 9 | 24138 | island | 0.05322 | 0.80085 | Not queried | 6.16 | 4.11e-05 | 2.11e-01 |
|  | 19 | 52391234 | 52391789 | PDFSTATSENSEMBLUCSC | 556 | 10 | 20963 | tss | 0.22893 | 0.95652 | Not queried | 6.16 | 1.08e-05 | 5.56e-02 |
|  | 22 | 45608428 | 45608713 | PDFSTATSENSEMBLUCSC | 286 | 9 | 27177 | tss | 0.10123 | 0.88663 | Not queried | 6.15 | 4.11e-05 | 2.11e-01 |
|  | 6 | 5085404 | 5085713 | PDFSTATSENSEMBLUCSC | 310 | 5 | 34543 | tss;island | 0.02101 | 0.60385 | Not queried | 6.15 | 7.94e-03 | 1.00e+00 |
|  | X | 134186198 | 134186640 | PDFSTATSENSEMBLUCSC | 443 | 11 | 42048 | island | 0.01875 | 0.54118 | FAM127B (margin: FAM127B) | 6.15 | 2.84e-06 | 1.46e-02 |
|  | 11 | 118781633 | 118781978 | PDFSTATSENSEMBLUCSC | 346 | 7 | 7719 | tss | 0.04448 | 0.81207 | Not queried | 6.15 | 5.83e-04 | 1.00e+00 |
|  | 17 | 4802692 | 4803053 | PDFSTATSENSEMBLUCSC | 362 | 8 | 16323 | gene;island | 0.01948 | 0.60940 | Not queried | 6.14 | 1.55e-04 | 7.99e-01 |
|  | 19 | 48216390 | 48216502 | PDFSTATSENSEMBLUCSC | 113 | 5 | 20872 | tss | 0.72825 | 0.02960 | Not queried | 6.14 | 7.94e-03 | 1.00e+00 |
|  | 5 | 140777344 | 140777611 | PDFSTATSENSEMBLUCSC | 268 | 6 | 31772 | gene;tss;island | 0.02786 | 0.63231 | Not queried | 6.13 | 2.16e-03 | 1.00e+00 |
|  | 3 | 184971652 | 184971844 | PDFSTATSENSEMBLUCSC | 193 | 4 | 28025 | gene;island | 0.02075 | 0.58099 | Not queried | 6.13 | 2.86e-02 | 1.00e+00 |
|  | 8 | 143859669 | 143859990 | PDFSTATSENSEMBLUCSC | 322 | 7 | 39874 | tss;island | 0.96274 | 0.26979 | Not queried | 6.13 | 5.83e-04 | 1.00e+00 |
|  | 13 | 26760422 | 26760793 | PDFSTATSENSEMBLUCSC | 372 | 4 | 11588 | island | 0.01844 | 0.45006 | Not queried | 6.12 | 2.86e-02 | 1.00e+00 |
|  | 2 | 63285365 | 63286049 | PDFSTATSENSEMBLUCSC | 685 | 8 | 24304 | island | 0.90755 | 0.09797 | Not queried | 6.12 | 1.55e-04 | 7.99e-01 |
|  | 11 | 504784 | 504937 | PDFSTATSENSEMBLUCSC | 154 | 5 | 6358 | gene;island | 0.17197 | 0.94748 | Not queried | 6.12 | 7.94e-03 | 1.00e+00 |
|  | 21 | 42792127 | 42792516 | PDFSTATSENSEMBLUCSC | 390 | 4 | 26516 | tss | 0.00895 | 0.32052 | Not queried | 6.12 | 1.14e-01 | 1.00e+00 |
|  | 17 | 19883326 | 19883716 | PDFSTATSENSEMBLUCSC | 391 | 4 | 18075 | island | 0.09622 | 0.85840 | Not queried | 6.11 | 2.86e-02 | 1.00e+00 |
|  | 2 | 145147129 | 145147558 | PDFSTATSENSEMBLUCSC | 430 | 5 | 22852 | gene;island | 0.20321 | 0.92247 | Not queried | 6.11 | 7.94e-03 | 1.00e+00 |
|  | 3 | 139257713 | 139257907 | PDFSTATSENSEMBLUCSC | 195 | 5 | 27927 | gene;island | 0.90253 | 0.11829 | Not queried | 6.11 | 7.94e-03 | 1.00e+00 |
|  | 5 | 115297690 | 115298079 | PDFSTATSENSEMBLUCSC | 390 | 5 | 32173 | tss;island | 0.06065 | 0.89840 | Not queried | 6.11 | 7.94e-03 | 1.00e+00 |
|  | 11 | 66824138 | 66824219 | PDFSTATSENSEMBLUCSC | 82 | 5 | 7462 | tss;island | 0.03881 | 0.87586 | Not queried | 6.11 | 7.94e-03 | 1.00e+00 |
|  | 16 | 70285806 | 70285978 | PDFSTATSENSEMBLUCSC | 173 | 5 | 16032 | island | 0.01688 | 0.55139 | Not queried | 6.10 | 7.94e-03 | 1.00e+00 |
|  | 6 | 32116538 | 32117725 | PDFSTATSENSEMBLUCSC | 1188 | 27 | 33995 | gene;island | 0.04216 | 0.83493 | PRRT1 (margin: PPT2; PPT2-EGFL8; PRRT1) | 6.10 | 7.75e-10 | 3.98e-06 |
|  | X | 154444356 | 154444619 | PDFSTATSENSEMBLUCSC | 264 | 7 | 41613 | tss | 0.01828 | 0.58242 | Not queried | 6.09 | 5.83e-04 | 1.00e+00 |
|  | 1 | 28286348 | 28286411 | PDFSTATSENSEMBLUCSC | 64 | 4 | 1419 | tss;island | 0.58884 | 0.02321 | Not queried | 6.09 | 2.86e-02 | 1.00e+00 |
|  | 16 | 57831745 | 57832309 | PDFSTATSENSEMBLUCSC | 565 | 6 | 14738 | gene | 0.03555 | 0.71958 | Not queried | 6.09 | 2.16e-03 | 1.00e+00 |
|  | Y | 21239219 | 21239607 | PDFSTATSENSEMBLUCSC | 389 | 5 | 42182 | island | 0.96934 | 0.45050 | Not queried | 6.08 | 7.94e-03 | 1.00e+00 |
|  | 10 | 38299242 | 38299574 | PDFSTATSENSEMBLUCSC | 333 | 9 | 5018 | tss | 0.01959 | 0.60107 | Not queried | 6.07 | 4.11e-05 | 2.11e-01 |
|  | 3 | 130745442 | 130745959 | PDFSTATSENSEMBLUCSC | 518 | 13 | 28549 | tss;island | 0.02093 | 0.59228 | NEK11; ASTE1 (margin: ATP2C1; NEK11; ASTE1) | 6.07 | 1.92e-07 | 9.88e-04 |
|  | 16 | 57832005 | 57832309 | PDFSTATSENSEMBLUCSC | 305 | 4 | 15236 | tss;island | 0.03555 | 0.71958 | Not queried | 6.07 | 2.86e-02 | 1.00e+00 |
|  | 10 | 38299242 | 38299653 | PDFSTATSENSEMBLUCSC | 412 | 10 | 5654 | island | 0.02191 | 0.54101 | Not queried | 6.06 | 1.08e-05 | 5.56e-02 |
|  | 4 | 54959419 | 54960259 | PDFSTATSENSEMBLUCSC | 841 | 8 | 30998 | island | 0.78149 | 0.05652 | Not queried | 6.06 | 1.55e-04 | 7.99e-01 |
|  | X | 77395258 | 77395543 | PDFSTATSENSEMBLUCSC | 286 | 6 | 41403 | tss | 0.01814 | 0.56593 | Not queried | 6.06 | 4.33e-03 | 1.00e+00 |
|  | 1 | 63785867 | 63786306 | PDFSTATSENSEMBLUCSC | 440 | 5 | 3334 | island | 0.80044 | 0.05076 | Not queried | 6.06 | 7.94e-03 | 1.00e+00 |
|  | 3 | 138658243 | 138659021 | PDFSTATSENSEMBLUCSC | 779 | 8 | 29486 | island | 0.81503 | 0.06970 | Not queried | 6.05 | 1.55e-04 | 7.99e-01 |
|  | 14 | 67878973 | 67879166 | PDFSTATSENSEMBLUCSC | 194 | 4 | 12442 | tss;island | 0.12088 | 0.87448 | Not queried | 6.05 | 2.86e-02 | 1.00e+00 |
|  | X | 134232821 | 134233109 | PDFSTATSENSEMBLUCSC | 289 | 5 | 41539 | tss;island | 0.02416 | 0.65074 | Not queried | 6.05 | 7.94e-03 | 1.00e+00 |
|  | 20 | 61051317 | 61052060 | PDFSTATSENSEMBLUCSC | 744 | 18 | 25833 | tss;island | 0.07097 | 0.83112 | RP13-379O24.3; GATA5 (margin: RP13-379O24.3; GATA5) | 6.04 | 2.20e-10 | 1.13e-06 |
|  | 5 | 140614369 | 140614765 | PDFSTATSENSEMBLUCSC | 397 | 4 | 31758 | gene;island | 0.18048 | 0.93208 | Not queried | 6.04 | 2.86e-02 | 1.00e+00 |
|  | 22 | 18923787 | 18923986 | PDFSTATSENSEMBLUCSC | 200 | 5 | 27245 | island | 0.60396 | 0.02260 | Not queried | 6.04 | 7.94e-03 | 1.00e+00 |
|  | 16 | 28634766 | 28635053 | PDFSTATSENSEMBLUCSC | 288 | 4 | 15757 | island | 0.02539 | 0.66314 | Not queried | 6.04 | 2.86e-02 | 1.00e+00 |
|  | 3 | 130745442 | 130745618 | PDFSTATSENSEMBLUCSC | 177 | 7 | 27907 | gene | 0.02231 | 0.59228 | Not queried | 6.04 | 5.83e-04 | 1.00e+00 |
|  | 13 | 111267667 | 111268031 | PDFSTATSENSEMBLUCSC | 365 | 11 | 11851 | island | 0.68442 | 0.03776 | CARKD (margin: CARKD) | 6.04 | 2.84e-06 | 1.46e-02 |
|  | 20 | 32307885 | 32308119 | PDFSTATSENSEMBLUCSC | 235 | 4 | 25356 | gene | 0.02686 | 0.62879 | Not queried | 6.04 | 2.86e-02 | 1.00e+00 |
|  | X | 77041722 | 77042040 | PDFSTATSENSEMBLUCSC | 319 | 7 | 41399 | tss | 0.01537 | 0.49969 | Not queried | 6.03 | 1.11e-02 | 1.00e+00 |
|  | X | 77041452 | 77042040 | PDFSTATSENSEMBLUCSC | 589 | 9 | 41883 | island | 0.01537 | 0.49969 | Not queried | 6.03 | 1.85e-03 | 1.00e+00 |
|  | 3 | 44753865 | 44754131 | PDFSTATSENSEMBLUCSC | 267 | 8 | 28218 | tss | 0.57126 | 0.02730 | Not queried | 6.03 | 1.55e-04 | 7.99e-01 |
|  | 1 | 209957965 | 209958343 | PDFSTATSENSEMBLUCSC | 379 | 8 | 2228 | tss;island | 0.02006 | 0.58789 | Not queried | 6.03 | 1.55e-04 | 7.99e-01 |
|  | 19 | 3801294 | 3801727 | PDFSTATSENSEMBLUCSC | 434 | 4 | 19688 | gene | 0.04971 | 0.80067 | Not queried | 6.02 | 2.86e-02 | 1.00e+00 |
|  | 4 | 111540884 | 111541339 | PDFSTATSENSEMBLUCSC | 456 | 4 | 30047 | gene;island | 0.91897 | 0.17917 | Not queried | 6.02 | 2.86e-02 | 1.00e+00 |
|  | 16 | 67907176 | 67907373 | PDFSTATSENSEMBLUCSC | 198 | 4 | 14775 | gene | 0.02196 | 0.57672 | Not queried | 6.02 | 2.86e-02 | 1.00e+00 |
|  | 12 | 25102274 | 25102521 | PDFSTATSENSEMBLUCSC | 248 | 5 | 10264 | island | 0.83844 | 0.02830 | Not queried | 6.01 | 7.94e-03 | 1.00e+00 |
|  | 18 | 22006316 | 22006735 | PDFSTATSENSEMBLUCSC | 420 | 11 | 19403 | island | 0.02611 | 0.60460 | IMPACT (margin: IMPACT; Y\_RNA; RP11-178F10.1) | 6.01 | 2.84e-06 | 1.46e-02 |
|  | 3 | 44770851 | 44771316 | PDFSTATSENSEMBLUCSC | 466 | 11 | 28998 | island | 0.54117 | 0.01985 | ZNF501 (margin: ZNF502; ZNF501; KIAA1143) | 6.00 | 2.84e-06 | 1.46e-02 |
|  | X | 77150966 | 77151316 | PDFSTATSENSEMBLUCSC | 351 | 11 | 41884 | island | 0.02388 | 0.58420 | MAGT1 (margin: RN7SL460P; COX7B; MAGT1) | 5.99 | 2.84e-06 | 1.46e-02 |
|  | X | 47518552 | 47518849 | PDFSTATSENSEMBLUCSC | 298 | 9 | 41285 | tss | 0.02006 | 0.56523 | Not queried | 5.99 | 4.11e-05 | 2.11e-01 |
|  | 19 | 48216390 | 48216613 | PDFSTATSENSEMBLUCSC | 224 | 6 | 22165 | island | 0.67313 | 0.02855 | Not queried | 5.99 | 2.16e-03 | 1.00e+00 |
|  | 19 | 12146404 | 12147014 | PDFSTATSENSEMBLUCSC | 611 | 13 | 21553 | island | 0.02165 | 0.52029 | CTD-2006C1.2; ZNF433; CTD-2006C1.10 (margin: CTD-2006C1.2; ZNF433; CTD-2006C1.10; RNA5SP465; ZNF878) | 5.98 | 1.92e-07 | 9.88e-04 |
|  | 6 | 18122938 | 18123232 | PDFSTATSENSEMBLUCSC | 295 | 11 | 34594 | tss;island | 0.03106 | 0.66955 | NHLRC1 (margin: NHLRC1; TPMT) | 5.98 | 2.84e-06 | 1.46e-02 |
|  | 1 | 68516463 | 68516713 | PDFSTATSENSEMBLUCSC | 251 | 6 | 1683 | tss | 0.05951 | 0.73903 | Not queried | 5.97 | 2.16e-03 | 1.00e+00 |
|  | 5 | 2755820 | 2756113 | PDFSTATSENSEMBLUCSC | 294 | 4 | 32598 | island | 0.64216 | 0.02669 | Not queried | 5.97 | 2.86e-02 | 1.00e+00 |
|  | 22 | 22901569 | 22902237 | PDFSTATSENSEMBLUCSC | 669 | 11 | 26963 | tss;island | 0.03729 | 0.77074 | LL22NC03-63E9.3; PRAME (margin: LL22NC03-63E9.3; PRAME) | 5.97 | 2.84e-06 | 1.46e-02 |
|  | 12 | 54331892 | 54332695 | PDFSTATSENSEMBLUCSC | 804 | 12 | 10451 | island | 0.01790 | 0.54688 | HOXC13; HOXC-AS5 (margin: HOXC13; HOXC-AS5) | 5.96 | 1.48e-06 | 7.60e-03 |
|  | 12 | 54331892 | 54332530 | PDFSTATSENSEMBLUCSC | 639 | 10 | 9723 | tss | 0.02034 | 0.54688 | Not queried | 5.96 | 2.17e-05 | 1.11e-01 |
|  | 1 | 244624521 | 244624883 | PDFSTATSENSEMBLUCSC | 363 | 6 | 4381 | island | 0.04035 | 0.66690 | Not queried | 5.96 | 2.16e-03 | 1.00e+00 |
|  | 10 | 27541298 | 27541418 | PDFSTATSENSEMBLUCSC | 121 | 4 | 4984 | tss | 0.06844 | 0.82123 | Not queried | 5.96 | 2.86e-02 | 1.00e+00 |
|  | X | 149861526 | 149861779 | PDFSTATSENSEMBLUCSC | 254 | 7 | 41566 | tss | 0.01672 | 0.54082 | Not queried | 5.95 | 5.83e-04 | 1.00e+00 |
|  | 21 | 36041334 | 36041699 | PDFSTATSENSEMBLUCSC | 366 | 8 | 26621 | island | 0.64654 | 0.02389 | Not queried | 5.95 | 1.55e-04 | 7.99e-01 |
|  | 22 | 42394590 | 42394853 | PDFSTATSENSEMBLUCSC | 264 | 4 | 27495 | island | 0.14866 | 0.85934 | Not queried | 5.94 | 2.86e-02 | 1.00e+00 |
|  | 11 | 65554042 | 65554410 | PDFSTATSENSEMBLUCSC | 369 | 8 | 7420 | tss;island | 0.03046 | 0.62839 | Not queried | 5.94 | 1.55e-04 | 7.99e-01 |
|  | 1 | 247802703 | 247803166 | PDFSTATSENSEMBLUCSC | 464 | 7 | 4407 | island | 0.02625 | 0.59201 | Not queried | 5.93 | 5.83e-04 | 1.00e+00 |
|  | 11 | 64739253 | 64739484 | PDFSTATSENSEMBLUCSC | 232 | 6 | 8409 | island | 0.05017 | 0.78044 | Not queried | 5.93 | 2.16e-03 | 1.00e+00 |
|  | 6 | 169653905 | 169654035 | PDFSTATSENSEMBLUCSC | 131 | 7 | 34502 | gene | 0.89263 | 0.03694 | Not queried | 5.93 | 5.83e-04 | 1.00e+00 |
|  | 19 | 12075882 | 12076348 | PDFSTATSENSEMBLUCSC | 467 | 6 | 19815 | gene | 0.03524 | 0.70038 | Not queried | 5.93 | 2.16e-03 | 1.00e+00 |
|  | 1 | 2985218 | 2985720 | PDFSTATSENSEMBLUCSC | 503 | 8 | 1176 | tss;island | 0.03953 | 0.64337 | Not queried | 5.92 | 1.55e-04 | 7.99e-01 |
|  | 17 | 31528 | 31552 | PDFSTATSENSEMBLUCSC | 25 | 5 | 16944 | tss;island | 0.03373 | 0.66559 | Not queried | 5.92 | 7.94e-03 | 1.00e+00 |
|  | X | 153059760 | 153060546 | PDFSTATSENSEMBLUCSC | 787 | 11 | 41587 | tss;island | 0.01792 | 0.55873 | SSR4; IDH3G (margin: SRPK3; SSR4; IDH3G; PDZD4) | 5.92 | 2.84e-06 | 1.46e-02 |
|  | X | 153626255 | 153626527 | PDFSTATSENSEMBLUCSC | 273 | 7 | 41596 | tss | 0.01956 | 0.57489 | Not queried | 5.92 | 5.83e-04 | 1.00e+00 |
|  | X | 25033767 | 25034241 | PDFSTATSENSEMBLUCSC | 475 | 9 | 41694 | island | 0.62523 | 0.01895 | Not queried | 5.91 | 4.11e-05 | 2.11e-01 |
|  | X | 25034093 | 25034241 | PDFSTATSENSEMBLUCSC | 149 | 5 | 41235 | tss | 0.55025 | 0.01895 | Not queried | 5.91 | 7.94e-03 | 1.00e+00 |
|  | 1 | 207818395 | 207818493 | PDFSTATSENSEMBLUCSC | 99 | 5 | 4141 | island | 0.01508 | 0.47950 | Not queried | 5.91 | 7.94e-03 | 1.00e+00 |
|  | 19 | 4831543 | 4832024 | PDFSTATSENSEMBLUCSC | 482 | 8 | 21354 | island | 0.06593 | 0.80716 | Not queried | 5.91 | 1.55e-04 | 7.99e-01 |
|  | 3 | 49236800 | 49236918 | PDFSTATSENSEMBLUCSC | 119 | 5 | 28282 | tss | 0.11693 | 0.88644 | Not queried | 5.90 | 7.94e-03 | 1.00e+00 |
|  | 22 | 45608345 | 45608713 | PDFSTATSENSEMBLUCSC | 369 | 10 | 26860 | gene | 0.12166 | 0.88573 | Not queried | 5.90 | 1.08e-05 | 5.56e-02 |
|  | 11 | 118781408 | 118781978 | PDFSTATSENSEMBLUCSC | 571 | 10 | 8838 | island | 0.04933 | 0.74742 | Not queried | 5.90 | 1.08e-05 | 5.56e-02 |
|  | 1 | 146543992 | 146544267 | PDFSTATSENSEMBLUCSC | 276 | 4 | 3679 | island | 0.60622 | 0.02707 | Not queried | 5.89 | 2.86e-02 | 1.00e+00 |
|  | 19 | 12146565 | 12147014 | PDFSTATSENSEMBLUCSC | 450 | 10 | 20451 | tss | 0.02184 | 0.51254 | Not queried | 5.89 | 1.08e-05 | 5.56e-02 |
|  | 16 | 80574553 | 80575018 | PDFSTATSENSEMBLUCSC | 466 | 6 | 16083 | island | 0.00881 | 0.42182 | Not queried | 5.89 | 2.16e-03 | 1.00e+00 |
|  | 7 | 94023308 | 94023869 | PDFSTATSENSEMBLUCSC | 562 | 9 | 37653 | tss | 0.03720 | 0.73981 | Not queried | 5.89 | 2.76e-03 | 1.00e+00 |
|  | 4 | 41882564 | 41883172 | PDFSTATSENSEMBLUCSC | 609 | 9 | 30958 | island | 0.94065 | 0.28028 | Not queried | 5.89 | 1.65e-04 | 8.46e-01 |
|  | 7 | 158789723 | 158790115 | PDFSTATSENSEMBLUCSC | 393 | 4 | 39095 | island | 0.07696 | 0.84161 | Not queried | 5.88 | 2.86e-02 | 1.00e+00 |
|  | 1 | 171220575 | 171220819 | PDFSTATSENSEMBLUCSC | 245 | 4 | 882 | gene | 0.71638 | 0.06310 | Not queried | 5.88 | 2.86e-02 | 1.00e+00 |
|  | X | 153060150 | 153060546 | PDFSTATSENSEMBLUCSC | 397 | 5 | 41148 | gene | 0.02271 | 0.57701 | Not queried | 5.88 | 7.94e-03 | 1.00e+00 |
|  | 1 | 15930094 | 15930386 | PDFSTATSENSEMBLUCSC | 293 | 4 | 2717 | island | 0.01698 | 0.44524 | Not queried | 5.87 | 2.86e-02 | 1.00e+00 |
|  | 5 | 140552347 | 140552600 | PDFSTATSENSEMBLUCSC | 254 | 4 | 31754 | gene | 0.23944 | 0.91761 | Not queried | 5.87 | 2.86e-02 | 1.00e+00 |
|  | 11 | 34535357 | 34535579 | PDFSTATSENSEMBLUCSC | 223 | 7 | 7222 | tss | 0.11876 | 0.93865 | Not queried | 5.86 | 5.83e-04 | 1.00e+00 |
|  | 11 | 125757328 | 125757753 | PDFSTATSENSEMBLUCSC | 426 | 7 | 6940 | gene;island | 0.02372 | 0.55294 | Not queried | 5.86 | 5.83e-04 | 1.00e+00 |
|  | 5 | 43396802 | 43397288 | PDFSTATSENSEMBLUCSC | 487 | 7 | 31547 | gene;island | 0.01005 | 0.49202 | Not queried | 5.86 | 5.83e-04 | 1.00e+00 |
|  | X | 150565243 | 150565466 | PDFSTATSENSEMBLUCSC | 224 | 4 | 41569 | tss;island | 0.02493 | 0.55674 | Not queried | 5.86 | 2.86e-02 | 1.00e+00 |
|  | 15 | 93652578 | 93653073 | PDFSTATSENSEMBLUCSC | 496 | 6 | 14454 | island | 0.24191 | 0.84751 | Not queried | 5.85 | 4.33e-03 | 1.00e+00 |
|  | 8 | 105479058 | 105479420 | PDFSTATSENSEMBLUCSC | 363 | 7 | 40459 | island | 0.04915 | 0.88359 | Not queried | 5.85 | 5.83e-04 | 1.00e+00 |
|  | 4 | 15704393 | 15704844 | PDFSTATSENSEMBLUCSC | 452 | 8 | 30888 | island | 0.12819 | 0.87699 | Not queried | 5.85 | 1.55e-04 | 7.99e-01 |
|  | 3 | 194117018 | 194117403 | PDFSTATSENSEMBLUCSC | 386 | 4 | 28047 | gene;island | 0.03917 | 0.69287 | Not queried | 5.85 | 2.00e-01 | 1.00e+00 |
|  | 22 | 22901830 | 22902237 | PDFSTATSENSEMBLUCSC | 408 | 6 | 26768 | gene | 0.05465 | 0.76276 | Not queried | 5.84 | 2.16e-03 | 1.00e+00 |
|  | 19 | 4831885 | 4832024 | PDFSTATSENSEMBLUCSC | 140 | 5 | 20337 | tss | 0.06890 | 0.82429 | Not queried | 5.84 | 7.94e-03 | 1.00e+00 |
|  | 19 | 3801294 | 3801922 | PDFSTATSENSEMBLUCSC | 629 | 7 | 21312 | island | 0.06036 | 0.76262 | Not queried | 5.84 | 5.83e-04 | 1.00e+00 |
|  | 5 | 169659618 | 169659950 | PDFSTATSENSEMBLUCSC | 333 | 10 | 33313 | island | 0.14094 | 0.89681 | Not queried | 5.83 | 1.08e-05 | 5.56e-02 |
|  | 1 | 155579369 | 155579964 | PDFSTATSENSEMBLUCSC | 596 | 9 | 1997 | tss;island | 0.77826 | 0.04673 | Not queried | 5.83 | 2.76e-03 | 1.00e+00 |
|  | 8 | 33457483 | 33457822 | PDFSTATSENSEMBLUCSC | 340 | 6 | 39578 | tss | 0.04862 | 0.84913 | Not queried | 5.82 | 4.33e-03 | 1.00e+00 |
|  | 4 | 113970617 | 113970745 | PDFSTATSENSEMBLUCSC | 129 | 4 | 30507 | tss | 0.72974 | 0.06165 | Not queried | 5.82 | 2.86e-02 | 1.00e+00 |
|  | X | 144903565 | 144903748 | PDFSTATSENSEMBLUCSC | 184 | 4 | 41132 | gene;island | 0.08342 | 0.85653 | Not queried | 5.81 | 2.86e-02 | 1.00e+00 |
|  | X | 101854121 | 101854594 | PDFSTATSENSEMBLUCSC | 474 | 9 | 41055 | gene | 0.01498 | 0.54054 | Not queried | 5.81 | 4.11e-05 | 2.11e-01 |
|  | 1 | 228651708 | 228652067 | PDFSTATSENSEMBLUCSC | 360 | 4 | 4288 | island | 0.02608 | 0.56200 | Not queried | 5.81 | 2.86e-02 | 1.00e+00 |
|  | 1 | 152161237 | 152161683 | PDFSTATSENSEMBLUCSC | 447 | 4 | 3749 | island | 0.02853 | 0.69180 | Not queried | 5.81 | 1.14e-01 | 1.00e+00 |
|  | 2 | 214148856 | 214149200 | PDFSTATSENSEMBLUCSC | 345 | 9 | 25026 | island | 0.01541 | 0.47248 | Not queried | 5.81 | 4.11e-05 | 2.11e-01 |
|  | 8 | 41733475 | 41733931 | PDFSTATSENSEMBLUCSC | 457 | 5 | 39232 | gene;island | 0.09504 | 0.79989 | Not queried | 5.80 | 7.94e-03 | 1.00e+00 |
|  | X | 24691042 | 24691232 | PDFSTATSENSEMBLUCSC | 191 | 6 | 41233 | tss | 0.13248 | 0.90735 | Not queried | 5.80 | 4.33e-03 | 1.00e+00 |
|  | 16 | 82044738 | 82045222 | PDFSTATSENSEMBLUCSC | 485 | 9 | 16093 | island | 0.05212 | 0.88106 | Not queried | 5.80 | 4.11e-05 | 2.11e-01 |
|  | 19 | 10698020 | 10698098 | PDFSTATSENSEMBLUCSC | 79 | 4 | 20417 | tss | 0.02326 | 0.53073 | Not queried | 5.80 | 2.86e-02 | 1.00e+00 |
|  | X | 125300035 | 125300111 | PDFSTATSENSEMBLUCSC | 77 | 5 | 41506 | tss;island | 0.01976 | 0.51858 | Not queried | 5.80 | 7.94e-03 | 1.00e+00 |
|  | 11 | 62474725 | 62475078 | PDFSTATSENSEMBLUCSC | 354 | 8 | 6625 | gene;tss;island | 0.72171 | 0.04626 | Not queried | 5.80 | 1.55e-04 | 7.99e-01 |
|  | 15 | 45670865 | 45671347 | PDFSTATSENSEMBLUCSC | 483 | 13 | 13304 | gene;tss;island | 0.91080 | 0.06551 | GATM (margin: GATM) | 5.79 | 8.65e-06 | 4.45e-02 |
|  | 5 | 140800398 | 140800495 | PDFSTATSENSEMBLUCSC | 98 | 4 | 32323 | tss | 0.08682 | 0.88669 | Not queried | 5.79 | 2.86e-02 | 1.00e+00 |
|  | X | 144902897 | 144903125 | PDFSTATSENSEMBLUCSC | 229 | 4 | 41131 | gene;island | 0.05102 | 0.77755 | Not queried | 5.78 | 2.86e-02 | 1.00e+00 |
|  | 16 | 31146999 | 31147054 | PDFSTATSENSEMBLUCSC | 56 | 4 | 14702 | gene | 0.02117 | 0.57553 | Not queried | 5.78 | 2.86e-02 | 1.00e+00 |
|  | 10 | 49674251 | 49674530 | PDFSTATSENSEMBLUCSC | 280 | 4 | 4566 | gene;island | 0.22414 | 0.92817 | Not queried | 5.78 | 2.86e-02 | 1.00e+00 |
|  | 6 | 119669112 | 119669372 | PDFSTATSENSEMBLUCSC | 261 | 4 | 34394 | gene;island | 0.60723 | 0.02803 | Not queried | 5.78 | 2.86e-02 | 1.00e+00 |
|  | 19 | 38754529 | 38755330 | PDFSTATSENSEMBLUCSC | 802 | 10 | 21934 | island | 0.02182 | 0.55594 | Not queried | 5.78 | 1.08e-05 | 5.56e-02 |
|  | X | 153656771 | 153657411 | PDFSTATSENSEMBLUCSC | 641 | 9 | 42141 | island | 0.03345 | 0.61658 | Not queried | 5.77 | 4.11e-05 | 2.11e-01 |
|  | X | 153775262 | 153776107 | PDFSTATSENSEMBLUCSC | 846 | 16 | 41164 | gene;tss;island | 0.02333 | 0.59012 | IKBKG; G6PD (margin: IKBKG; G6PD) | 5.77 | 3.33e-09 | 1.71e-05 |
|  | 21 | 36041334 | 36041683 | PDFSTATSENSEMBLUCSC | 350 | 7 | 26490 | tss | 0.66612 | 0.03003 | Not queried | 5.76 | 5.83e-04 | 1.00e+00 |
|  | 2 | 24232603 | 24232974 | PDFSTATSENSEMBLUCSC | 372 | 11 | 24087 | island | 0.25470 | 0.94043 | MFSD2B (margin: UBXN2A; MFSD2B) | 5.75 | 2.84e-06 | 1.46e-02 |
|  | 2 | 131010423 | 131010871 | PDFSTATSENSEMBLUCSC | 449 | 5 | 24679 | island | 0.05326 | 0.70387 | Not queried | 5.75 | 7.94e-03 | 1.00e+00 |
|  | 14 | 75593334 | 75593738 | PDFSTATSENSEMBLUCSC | 405 | 5 | 12116 | gene | 0.60249 | 0.02742 | Not queried | 5.75 | 7.94e-03 | 1.00e+00 |
|  | 16 | 68481049 | 68481543 | PDFSTATSENSEMBLUCSC | 495 | 6 | 14785 | gene;island | 0.06177 | 0.75884 | Not queried | 5.74 | 2.16e-03 | 1.00e+00 |
|  | 12 | 30948636 | 30948794 | PDFSTATSENSEMBLUCSC | 159 | 5 | 10282 | island | 0.87502 | 0.11301 | Not queried | 5.74 | 7.94e-03 | 1.00e+00 |
|  | 12 | 51236582 | 51236693 | PDFSTATSENSEMBLUCSC | 112 | 4 | 9683 | tss | 0.11415 | 0.91108 | Not queried | 5.74 | 2.86e-02 | 1.00e+00 |
|  | X | 135579428 | 135579487 | PDFSTATSENSEMBLUCSC | 60 | 5 | 41119 | gene;tss;island | 0.01687 | 0.43278 | Not queried | 5.74 | 7.94e-03 | 1.00e+00 |
|  | X | 152939154 | 152939615 | PDFSTATSENSEMBLUCSC | 462 | 4 | 41143 | gene;tss;island | 0.15826 | 0.94389 | Not queried | 5.74 | 1.14e-01 | 1.00e+00 |
|  | X | 47518016 | 47518849 | PDFSTATSENSEMBLUCSC | 834 | 17 | 40982 | gene;island | 0.02432 | 0.52067 | RP1-212G6.7; UXT (margin: RP1-212G6.7; ELK1; UXT) | 5.74 | 3.43e-09 | 1.76e-05 |
|  | 6 | 33043762 | 33044012 | PDFSTATSENSEMBLUCSC | 251 | 6 | 34120 | gene | 0.72126 | 0.04786 | Not queried | 5.73 | 2.16e-03 | 1.00e+00 |
|  | 8 | 99959430 | 99959723 | PDFSTATSENSEMBLUCSC | 294 | 4 | 39311 | gene;island | 0.85236 | 0.04641 | Not queried | 5.72 | 2.86e-02 | 1.00e+00 |
|  | 5 | 68628240 | 68628738 | PDFSTATSENSEMBLUCSC | 499 | 5 | 32788 | island | 0.02457 | 0.60054 | Not queried | 5.72 | 7.94e-03 | 1.00e+00 |
|  | 5 | 169659618 | 169659863 | PDFSTATSENSEMBLUCSC | 246 | 9 | 32424 | tss | 0.21994 | 0.89731 | Not queried | 5.72 | 4.11e-05 | 2.11e-01 |
|  | 20 | 47896769 | 47897451 | PDFSTATSENSEMBLUCSC | 683 | 7 | 25409 | gene;island | 0.88857 | 0.15732 | Not queried | 5.72 | 5.83e-04 | 1.00e+00 |
|  | 20 | 47896769 | 47897207 | PDFSTATSENSEMBLUCSC | 439 | 5 | 25768 | tss | 0.88857 | 0.15732 | Not queried | 5.72 | 7.94e-03 | 1.00e+00 |
|  | 8 | 97171668 | 97172012 | PDFSTATSENSEMBLUCSC | 345 | 4 | 39304 | gene;island | 0.12110 | 0.76955 | Not queried | 5.71 | 2.86e-02 | 1.00e+00 |
|  | 20 | 32307885 | 32308529 | PDFSTATSENSEMBLUCSC | 645 | 11 | 26062 | island | 0.02402 | 0.61683 | PXMP4 (margin: ZNF341; PXMP4; RP4-553F4.2) | 5.71 | 2.84e-06 | 1.46e-02 |
|  | 20 | 32308170 | 32308529 | PDFSTATSENSEMBLUCSC | 360 | 7 | 25646 | tss | 0.02402 | 0.57988 | Not queried | 5.71 | 5.83e-04 | 1.00e+00 |
|  | 1 | 209979487 | 209979779 | PDFSTATSENSEMBLUCSC | 293 | 8 | 2229 | tss | 0.02740 | 0.59386 | Not queried | 5.71 | 1.55e-04 | 7.99e-01 |
|  | 5 | 140749735 | 140749834 | PDFSTATSENSEMBLUCSC | 100 | 4 | 31767 | gene;tss;island | 0.05757 | 0.70866 | Not queried | 5.70 | 2.86e-02 | 1.00e+00 |
|  | 1 | 68515788 | 68516713 | PDFSTATSENSEMBLUCSC | 926 | 17 | 3362 | island | 0.05192 | 0.72874 | GNG12-AS1; DIRAS3; ARL5AP3 (margin: GNG12-AS1; RP4-609E1.2; AL157407.1; DIRAS3; ARL5AP3) | 5.70 | 8.57e-10 | 4.40e-06 |
|  | 1 | 68515788 | 68516453 | PDFSTATSENSEMBLUCSC | 666 | 11 | 573 | gene | 0.05192 | 0.66770 | GNG12-AS1; DIRAS3; ARL5AP3 (margin: GNG12-AS1; RP4-609E1.2; AL157407.1; DIRAS3; ARL5AP3) | 5.70 | 2.84e-06 | 1.46e-02 |
|  | 4 | 26030463 | 26030691 | PDFSTATSENSEMBLUCSC | 229 | 4 | 30914 | island | 0.03189 | 0.60315 | Not queried | 5.70 | 2.86e-02 | 1.00e+00 |
|  | 1 | 46859671 | 46859845 | PDFSTATSENSEMBLUCSC | 175 | 6 | 1597 | tss;island | 0.93230 | 0.27915 | Not queried | 5.70 | 2.60e-02 | 1.00e+00 |
|  | 13 | 111267667 | 111267991 | PDFSTATSENSEMBLUCSC | 325 | 9 | 11506 | tss | 0.68442 | 0.04301 | Not queried | 5.70 | 4.11e-05 | 2.11e-01 |
|  | 6 | 157876915 | 157877218 | PDFSTATSENSEMBLUCSC | 304 | 5 | 34458 | gene | 0.04447 | 0.75577 | Not queried | 5.69 | 1.59e-02 | 1.00e+00 |
|  | 1 | 87617142 | 87617350 | PDFSTATSENSEMBLUCSC | 209 | 4 | 612 | gene;island | 0.77020 | 0.06188 | Not queried | 5.69 | 2.00e-01 | 1.00e+00 |
|  | 8 | 105479318 | 105479420 | PDFSTATSENSEMBLUCSC | 103 | 4 | 39797 | tss | 0.12715 | 0.89580 | Not queried | 5.69 | 2.86e-02 | 1.00e+00 |
|  | 11 | 65816463 | 65816646 | PDFSTATSENSEMBLUCSC | 184 | 4 | 6705 | gene | 0.75030 | 0.04526 | Not queried | 5.69 | 2.86e-02 | 1.00e+00 |
|  | 2 | 63284481 | 63284784 | PDFSTATSENSEMBLUCSC | 304 | 5 | 24303 | island | 0.95404 | 0.23909 | Not queried | 5.68 | 7.94e-03 | 1.00e+00 |
|  | 17 | 7287652 | 7288001 | PDFSTATSENSEMBLUCSC | 350 | 4 | 16357 | gene;island | 0.87216 | 0.13268 | Not queried | 5.68 | 2.86e-02 | 1.00e+00 |
|  | 5 | 524679 | 524941 | PDFSTATSENSEMBLUCSC | 263 | 4 | 31901 | tss | 0.03871 | 0.68803 | Not queried | 5.68 | 2.86e-02 | 1.00e+00 |
|  | 5 | 167956306 | 167956425 | PDFSTATSENSEMBLUCSC | 120 | 4 | 32419 | tss | 0.02433 | 0.51330 | Not queried | 5.68 | 2.86e-02 | 1.00e+00 |
|  | 22 | 18923876 | 18923986 | PDFSTATSENSEMBLUCSC | 111 | 4 | 26910 | tss | 0.62437 | 0.02772 | Not queried | 5.68 | 2.86e-02 | 1.00e+00 |
|  | 5 | 100239919 | 100240223 | PDFSTATSENSEMBLUCSC | 305 | 4 | 32138 | tss;island | 0.67800 | 0.03512 | Not queried | 5.67 | 2.86e-02 | 1.00e+00 |
|  | 18 | 22006316 | 22006514 | PDFSTATSENSEMBLUCSC | 199 | 8 | 19180 | tss | 0.02790 | 0.60540 | Not queried | 5.67 | 1.55e-04 | 7.99e-01 |
|  | 8 | 82192519 | 82192931 | PDFSTATSENSEMBLUCSC | 413 | 10 | 40352 | island | 0.08027 | 0.85785 | Not queried | 5.67 | 1.08e-05 | 5.56e-02 |
|  | 4 | 113739249 | 113739379 | PDFSTATSENSEMBLUCSC | 131 | 5 | 30055 | gene | 0.70384 | 0.02297 | Not queried | 5.67 | 7.94e-03 | 1.00e+00 |
|  | 19 | 50191342 | 50191683 | PDFSTATSENSEMBLUCSC | 342 | 5 | 20124 | gene | 0.12211 | 0.89265 | Not queried | 5.66 | 7.94e-03 | 1.00e+00 |
|  | 8 | 82543035 | 82543431 | PDFSTATSENSEMBLUCSC | 397 | 5 | 40354 | island | 0.10437 | 0.72090 | Not queried | 5.66 | 7.94e-03 | 1.00e+00 |
|  | 7 | 57484635 | 57484821 | PDFSTATSENSEMBLUCSC | 187 | 4 | 38455 | island | 0.05658 | 0.72984 | Not queried | 5.66 | 2.86e-02 | 1.00e+00 |
|  | 5 | 140536175 | 140536496 | PDFSTATSENSEMBLUCSC | 322 | 4 | 31753 | gene;island | 0.04401 | 0.77251 | Not queried | 5.66 | 2.86e-02 | 1.00e+00 |
|  | 6 | 32977138 | 32977349 | PDFSTATSENSEMBLUCSC | 212 | 5 | 34114 | gene | 0.73315 | 0.14884 | Not queried | 5.66 | 1.59e-02 | 1.00e+00 |
|  | 18 | 42643730 | 42643936 | PDFSTATSENSEMBLUCSC | 207 | 5 | 19064 | gene;island | 0.22393 | 0.91121 | Not queried | 5.66 | 7.94e-03 | 1.00e+00 |
|  | 1 | 208132388 | 208132787 | PDFSTATSENSEMBLUCSC | 400 | 4 | 4145 | island | 0.55861 | 0.03149 | Not queried | 5.65 | 2.86e-02 | 1.00e+00 |
|  | 7 | 1609793 | 1609908 | PDFSTATSENSEMBLUCSC | 116 | 7 | 36702 | gene;tss;island | 0.02857 | 0.63603 | Not queried | 5.65 | 5.83e-04 | 1.00e+00 |
|  | 18 | 32173084 | 32173227 | PDFSTATSENSEMBLUCSC | 144 | 4 | 19052 | gene;tss | 0.13720 | 0.88435 | Not queried | 5.65 | 2.86e-02 | 1.00e+00 |
|  | 6 | 27114864 | 27115249 | PDFSTATSENSEMBLUCSC | 386 | 5 | 34649 | tss;island | 0.02662 | 0.66394 | Not queried | 5.65 | 7.94e-03 | 1.00e+00 |
|  | X | 153626255 | 153626754 | PDFSTATSENSEMBLUCSC | 500 | 10 | 42138 | island | 0.02379 | 0.56661 | Not queried | 5.65 | 1.08e-05 | 5.56e-02 |
|  | 19 | 10697913 | 10698098 | PDFSTATSENSEMBLUCSC | 186 | 5 | 21504 | island | 0.02797 | 0.51433 | Not queried | 5.65 | 7.94e-03 | 1.00e+00 |
|  | 5 | 42995123 | 42995527 | PDFSTATSENSEMBLUCSC | 405 | 5 | 32701 | island | 0.69890 | 0.07248 | Not queried | 5.64 | 7.94e-03 | 1.00e+00 |
|  | X | 70752639 | 70752883 | PDFSTATSENSEMBLUCSC | 245 | 7 | 41378 | tss | 0.01412 | 0.40644 | Not queried | 5.64 | 5.83e-04 | 1.00e+00 |
|  | 5 | 140535215 | 140535810 | PDFSTATSENSEMBLUCSC | 596 | 6 | 33153 | island | 0.01953 | 0.43143 | Not queried | 5.64 | 2.16e-03 | 1.00e+00 |
|  | 17 | 4802692 | 4802906 | PDFSTATSENSEMBLUCSC | 215 | 6 | 17000 | tss | 0.02428 | 0.58387 | Not queried | 5.64 | 2.16e-03 | 1.00e+00 |
|  | 12 | 3862417 | 3862597 | PDFSTATSENSEMBLUCSC | 181 | 10 | 9470 | tss | 0.07107 | 0.80539 | Not queried | 5.63 | 1.08e-05 | 5.56e-02 |
|  | 5 | 140753415 | 140753570 | PDFSTATSENSEMBLUCSC | 156 | 7 | 32318 | tss | 0.03385 | 0.66696 | Not queried | 5.63 | 1.17e-03 | 1.00e+00 |
|  | 17 | 15686294 | 15686473 | PDFSTATSENSEMBLUCSC | 180 | 4 | 18010 | island | 0.26759 | 0.94356 | Not queried | 5.63 | 2.86e-02 | 1.00e+00 |
|  | 4 | 46995637 | 46995821 | PDFSTATSENSEMBLUCSC | 185 | 5 | 30328 | tss | 0.01761 | 0.47590 | Not queried | 5.62 | 7.94e-03 | 1.00e+00 |
|  | 19 | 48947560 | 48947631 | PDFSTATSENSEMBLUCSC | 72 | 4 | 20088 | gene;tss;island | 0.09967 | 0.78671 | Not queried | 5.62 | 2.86e-02 | 1.00e+00 |
|  | 8 | 65284438 | 65284519 | PDFSTATSENSEMBLUCSC | 82 | 4 | 40275 | island | 0.82342 | 0.08892 | Not queried | 5.62 | 2.86e-02 | 1.00e+00 |
|  | 5 | 524503 | 524941 | PDFSTATSENSEMBLUCSC | 439 | 5 | 32527 | island | 0.04405 | 0.68382 | Not queried | 5.61 | 7.94e-03 | 1.00e+00 |
|  | Y | 21239332 | 21239607 | PDFSTATSENSEMBLUCSC | 276 | 4 | 42170 | tss | 0.96958 | 0.46444 | Not queried | 5.61 | 2.86e-02 | 1.00e+00 |
|  | 6 | 111580510 | 111580939 | PDFSTATSENSEMBLUCSC | 430 | 6 | 34380 | gene | 0.40420 | 0.01636 | Not queried | 5.60 | 2.16e-03 | 1.00e+00 |
|  | X | 153672702 | 153672898 | PDFSTATSENSEMBLUCSC | 197 | 4 | 41161 | gene;island | 0.01841 | 0.50491 | Not queried | 5.60 | 2.86e-02 | 1.00e+00 |
|  | 17 | 16593426 | 16593613 | PDFSTATSENSEMBLUCSC | 188 | 6 | 17128 | tss;island | 0.06719 | 0.84711 | Not queried | 5.60 | 4.33e-03 | 1.00e+00 |
|  | 5 | 20305576 | 20305935 | PDFSTATSENSEMBLUCSC | 360 | 4 | 32648 | island | 0.10775 | 0.87741 | Not queried | 5.60 | 2.86e-02 | 1.00e+00 |
|  | 1 | 2986362 | 2986566 | PDFSTATSENSEMBLUCSC | 205 | 4 | 96 | gene;island | 0.03018 | 0.60900 | Not queried | 5.59 | 2.86e-02 | 1.00e+00 |
|  | 3 | 42306467 | 42306974 | PDFSTATSENSEMBLUCSC | 508 | 7 | 28200 | tss | 0.07905 | 0.75688 | Not queried | 5.58 | 5.83e-04 | 1.00e+00 |
|  | 9 | 137392877 | 137393288 | PDFSTATSENSEMBLUCSC | 412 | 5 | 40905 | island | 0.23018 | 0.90683 | Not queried | 5.58 | 7.94e-03 | 1.00e+00 |
|  | 6 | 49681299 | 49681391 | PDFSTATSENSEMBLUCSC | 93 | 5 | 34963 | tss | 0.01624 | 0.44944 | Not queried | 5.58 | 7.94e-03 | 1.00e+00 |
|  | 6 | 27114943 | 27115249 | PDFSTATSENSEMBLUCSC | 307 | 4 | 33554 | gene | 0.03402 | 0.62118 | Not queried | 5.58 | 2.86e-02 | 1.00e+00 |
|  | 6 | 168435914 | 168436099 | PDFSTATSENSEMBLUCSC | 186 | 4 | 34493 | gene;island | 0.10466 | 0.75112 | Not queried | 5.58 | 2.86e-02 | 1.00e+00 |
|  | 6 | 161100017 | 161100672 | PDFSTATSENSEMBLUCSC | 656 | 6 | 36552 | island | 0.11688 | 0.89567 | Not queried | 5.57 | 2.16e-03 | 1.00e+00 |
|  | 11 | 45825356 | 45825579 | PDFSTATSENSEMBLUCSC | 224 | 8 | 7245 | tss;island | 0.85411 | 0.08061 | Not queried | 5.57 | 1.55e-04 | 7.99e-01 |
|  | 12 | 103344336 | 103344685 | PDFSTATSENSEMBLUCSC | 350 | 5 | 10700 | island | 0.07534 | 0.73371 | Not queried | 5.57 | 7.94e-03 | 1.00e+00 |
|  | X | 125686801 | 125687207 | PDFSTATSENSEMBLUCSC | 407 | 8 | 42013 | island | 0.12927 | 0.91288 | Not queried | 5.56 | 3.11e-04 | 1.00e+00 |
|  | 2 | 2193511 | 2193665 | PDFSTATSENSEMBLUCSC | 155 | 4 | 22505 | gene;island | 0.10342 | 0.82006 | Not queried | 5.56 | 2.86e-02 | 1.00e+00 |
|  | 10 | 101874829 | 101875138 | PDFSTATSENSEMBLUCSC | 310 | 5 | 5999 | island | 0.27193 | 0.94399 | Not queried | 5.56 | 7.94e-03 | 1.00e+00 |
|  | 19 | 41106155 | 41106351 | PDFSTATSENSEMBLUCSC | 197 | 4 | 20011 | gene;tss;island | 0.03612 | 0.68406 | Not queried | 5.55 | 2.86e-02 | 1.00e+00 |
|  | 10 | 95361132 | 95361386 | PDFSTATSENSEMBLUCSC | 255 | 5 | 5222 | tss;island | 0.13955 | 0.82050 | Not queried | 5.55 | 7.94e-03 | 1.00e+00 |
|  | 2 | 154336045 | 154336401 | PDFSTATSENSEMBLUCSC | 357 | 5 | 23631 | tss;island | 0.50764 | 0.01701 | Not queried | 5.55 | 7.94e-03 | 1.00e+00 |
|  | 5 | 151066268 | 151066486 | PDFSTATSENSEMBLUCSC | 219 | 4 | 31809 | gene | 0.01575 | 0.42653 | Not queried | 5.55 | 2.86e-02 | 1.00e+00 |
|  | 1 | 240160972 | 240161447 | PDFSTATSENSEMBLUCSC | 476 | 5 | 4359 | island | 0.02548 | 0.54339 | Not queried | 5.55 | 7.94e-03 | 1.00e+00 |
|  | 19 | 4304933 | 4305239 | PDFSTATSENSEMBLUCSC | 307 | 4 | 19695 | gene;island | 0.92437 | 0.08067 | Not queried | 5.55 | 1.14e-01 | 1.00e+00 |
|  | 8 | 82192519 | 82192777 | PDFSTATSENSEMBLUCSC | 259 | 7 | 39720 | tss | 0.05601 | 0.83799 | Not queried | 5.55 | 5.83e-04 | 1.00e+00 |
|  | X | 17755642 | 17755839 | PDFSTATSENSEMBLUCSC | 198 | 4 | 40945 | gene | 0.31724 | 0.01331 | Not queried | 5.54 | 2.86e-02 | 1.00e+00 |
|  | 6 | 166876490 | 166877038 | PDFSTATSENSEMBLUCSC | 549 | 8 | 34480 | gene | 0.16334 | 0.84051 | Not queried | 5.54 | 1.55e-04 | 7.99e-01 |
|  | 8 | 22960461 | 22960619 | PDFSTATSENSEMBLUCSC | 159 | 4 | 39176 | gene | 0.02882 | 0.56932 | Not queried | 5.54 | 2.86e-02 | 1.00e+00 |
|  | 4 | 190862026 | 190862268 | PDFSTATSENSEMBLUCSC | 243 | 4 | 30173 | gene | 0.01871 | 0.41459 | Not queried | 5.54 | 2.86e-02 | 1.00e+00 |
|  | 10 | 2815004 | 2815547 | PDFSTATSENSEMBLUCSC | 544 | 6 | 5484 | island | 0.02801 | 0.56235 | Not queried | 5.54 | 2.16e-03 | 1.00e+00 |
|  | 15 | 26874098 | 26874515 | PDFSTATSENSEMBLUCSC | 418 | 4 | 13232 | gene;island | 0.23718 | 0.93045 | Not queried | 5.54 | 2.86e-02 | 1.00e+00 |
|  | 17 | 7791882 | 7792134 | PDFSTATSENSEMBLUCSC | 253 | 5 | 17069 | tss | 0.06522 | 0.87052 | Not queried | 5.54 | 7.94e-03 | 1.00e+00 |
|  | 4 | 8271196 | 8271414 | PDFSTATSENSEMBLUCSC | 219 | 6 | 30249 | tss | 0.04365 | 0.71309 | Not queried | 5.53 | 8.66e-03 | 1.00e+00 |
|  | 3 | 37033625 | 37034787 | PDFSTATSENSEMBLUCSC | 1163 | 22 | 27674 | gene | 0.39558 | 0.01455 | MLH1; EPM2AIP1 (margin: MLH1; RP11-129K12.4; RP11-129K12.3; EPM2AIP1) | 5.52 | 6.65e-12 | 3.42e-08 |
|  | 8 | 1764878 | 1765387 | PDFSTATSENSEMBLUCSC | 510 | 10 | 39472 | tss | 0.02434 | 0.52256 | Not queried | 5.52 | 1.08e-05 | 5.56e-02 |
|  | 1 | 53067637 | 53067965 | PDFSTATSENSEMBLUCSC | 329 | 5 | 1629 | tss;island | 0.06473 | 0.79736 | Not queried | 5.52 | 7.94e-03 | 1.00e+00 |
|  | 13 | 79181285 | 79181613 | PDFSTATSENSEMBLUCSC | 329 | 4 | 11766 | island | 0.74525 | 0.03678 | Not queried | 5.52 | 2.86e-02 | 1.00e+00 |
|  | 10 | 134838014 | 134838656 | PDFSTATSENSEMBLUCSC | 643 | 7 | 6307 | island | 0.15922 | 0.84655 | Not queried | 5.51 | 5.83e-04 | 1.00e+00 |
|  | 1 | 243646235 | 243646402 | PDFSTATSENSEMBLUCSC | 168 | 4 | 1109 | gene;island | 0.06440 | 0.80293 | Not queried | 5.51 | 2.86e-02 | 1.00e+00 |
|  | 10 | 134901199 | 134901305 | PDFSTATSENSEMBLUCSC | 107 | 7 | 5434 | tss | 0.02691 | 0.48235 | Not queried | 5.51 | 5.83e-04 | 1.00e+00 |
|  | 15 | 25841831 | 25842111 | PDFSTATSENSEMBLUCSC | 281 | 4 | 13928 | island | 0.18517 | 0.87033 | Not queried | 5.51 | 2.86e-02 | 1.00e+00 |
|  | 7 | 27195918 | 27196286 | PDFSTATSENSEMBLUCSC | 369 | 4 | 36874 | gene | 0.01727 | 0.40674 | Not queried | 5.50 | 2.86e-02 | 1.00e+00 |
|  | 8 | 10683044 | 10683266 | PDFSTATSENSEMBLUCSC | 223 | 5 | 39137 | gene;tss | 0.01389 | 0.35042 | Not queried | 5.50 | 9.52e-02 | 1.00e+00 |
|  | 2 | 119599459 | 119600002 | PDFSTATSENSEMBLUCSC | 544 | 6 | 24606 | island | 0.92159 | 0.15741 | Not queried | 5.50 | 2.16e-03 | 1.00e+00 |
|  | 19 | 37096010 | 37096588 | PDFSTATSENSEMBLUCSC | 579 | 7 | 19969 | gene;tss;island | 0.03953 | 0.62178 | Not queried | 5.50 | 5.83e-04 | 1.00e+00 |
|  | 16 | 89883988 | 89884347 | PDFSTATSENSEMBLUCSC | 360 | 5 | 15402 | tss;island | 0.93390 | 0.24414 | Not queried | 5.50 | 7.94e-03 | 1.00e+00 |
|  | 20 | 29535007 | 29535150 | PDFSTATSENSEMBLUCSC | 144 | 4 | 26028 | island | 0.02981 | 0.57516 | Not queried | 5.49 | 2.86e-02 | 1.00e+00 |
|  | 1 | 20669724 | 20669975 | PDFSTATSENSEMBLUCSC | 252 | 4 | 286 | gene;island | 0.03384 | 0.56648 | Not queried | 5.48 | 2.86e-02 | 1.00e+00 |
|  | 7 | 105221985 | 105222180 | PDFSTATSENSEMBLUCSC | 196 | 7 | 37754 | tss | 0.03022 | 0.55437 | Not queried | 5.48 | 5.83e-04 | 1.00e+00 |
|  | 17 | 45401148 | 45401312 | PDFSTATSENSEMBLUCSC | 165 | 4 | 17408 | tss | 0.02482 | 0.55740 | Not queried | 5.48 | 2.86e-02 | 1.00e+00 |
|  | 8 | 33457226 | 33457822 | PDFSTATSENSEMBLUCSC | 597 | 8 | 40150 | island | 0.04635 | 0.75722 | Not queried | 5.48 | 3.11e-04 | 1.00e+00 |
|  | 8 | 145016475 | 145016828 | PDFSTATSENSEMBLUCSC | 354 | 4 | 39435 | gene;island | 0.01771 | 0.38141 | Not queried | 5.47 | 2.86e-02 | 1.00e+00 |
|  | X | 153640221 | 153640674 | PDFSTATSENSEMBLUCSC | 454 | 7 | 41158 | gene;island | 0.03801 | 0.62181 | Not queried | 5.46 | 5.83e-04 | 1.00e+00 |
|  | X | 153640466 | 153640674 | PDFSTATSENSEMBLUCSC | 209 | 5 | 41598 | tss | 0.03801 | 0.62423 | Not queried | 5.46 | 7.94e-03 | 1.00e+00 |
|  | 5 | 64398797 | 64399099 | PDFSTATSENSEMBLUCSC | 303 | 4 | 32771 | island | 0.10179 | 0.83685 | Not queried | 5.46 | 2.86e-02 | 1.00e+00 |
|  | X | 70752639 | 70753023 | PDFSTATSENSEMBLUCSC | 385 | 10 | 41026 | gene;island | 0.01573 | 0.37933 | Not queried | 5.46 | 1.08e-05 | 5.56e-02 |
|  | 3 | 49449687 | 49449964 | PDFSTATSENSEMBLUCSC | 278 | 6 | 27742 | gene | 0.02184 | 0.52005 | Not queried | 5.46 | 2.16e-03 | 1.00e+00 |
|  | X | 125686936 | 125687207 | PDFSTATSENSEMBLUCSC | 272 | 7 | 41507 | tss | 0.13699 | 0.89008 | Not queried | 5.46 | 1.17e-03 | 1.00e+00 |
|  | 5 | 167956306 | 167956581 | PDFSTATSENSEMBLUCSC | 276 | 5 | 33308 | island | 0.02809 | 0.51901 | Not queried | 5.45 | 7.94e-03 | 1.00e+00 |
|  | 2 | 198364525 | 198365150 | PDFSTATSENSEMBLUCSC | 626 | 16 | 22931 | gene;tss;island | 0.02383 | 0.47477 | HSPE1; MOB4; HSPD1 (margin: HSPE1; MOB4; HSPD1) | 5.45 | 3.33e-09 | 1.71e-05 |
|  | X | 46306474 | 46306863 | PDFSTATSENSEMBLUCSC | 390 | 9 | 41737 | island | 0.03519 | 0.59478 | Not queried | 5.45 | 4.11e-05 | 2.11e-01 |
|  | X | 46306474 | 46306568 | PDFSTATSENSEMBLUCSC | 95 | 5 | 41270 | tss | 0.03519 | 0.59478 | Not queried | 5.45 | 7.94e-03 | 1.00e+00 |
|  | 22 | 17602561 | 17602681 | PDFSTATSENSEMBLUCSC | 121 | 4 | 26900 | tss;island | 0.53575 | 0.02949 | Not queried | 5.45 | 2.86e-02 | 1.00e+00 |
|  | 5 | 140718283 | 140718405 | PDFSTATSENSEMBLUCSC | 123 | 4 | 31759 | gene;island | 0.03517 | 0.57160 | Not queried | 5.44 | 1.14e-01 | 1.00e+00 |
|  | 17 | 5095195 | 5095455 | PDFSTATSENSEMBLUCSC | 261 | 8 | 17009 | tss | 0.02335 | 0.54302 | Not queried | 5.44 | 1.55e-04 | 7.99e-01 |
|  | 1 | 207224510 | 207224740 | PDFSTATSENSEMBLUCSC | 231 | 4 | 2213 | tss | 0.40079 | 0.00873 | Not queried | 5.44 | 5.71e-02 | 1.00e+00 |
|  | 5 | 178986620 | 178986906 | PDFSTATSENSEMBLUCSC | 287 | 5 | 31874 | gene;island | 0.48789 | 0.01809 | Not queried | 5.44 | 7.94e-03 | 1.00e+00 |
|  | 4 | 15704393 | 15704550 | PDFSTATSENSEMBLUCSC | 158 | 4 | 30264 | tss | 0.19572 | 0.84256 | Not queried | 5.44 | 2.86e-02 | 1.00e+00 |
|  | 7 | 155283738 | 155284062 | PDFSTATSENSEMBLUCSC | 325 | 4 | 39008 | island | 0.04801 | 0.69853 | Not queried | 5.43 | 2.86e-02 | 1.00e+00 |
|  | 3 | 49066699 | 49066825 | PDFSTATSENSEMBLUCSC | 127 | 4 | 27735 | gene | 0.02266 | 0.49902 | Not queried | 5.43 | 2.86e-02 | 1.00e+00 |
|  | 7 | 65970924 | 65971372 | PDFSTATSENSEMBLUCSC | 449 | 4 | 38472 | island | 0.07290 | 0.81089 | Not queried | 5.43 | 5.71e-02 | 1.00e+00 |
|  | 7 | 127744210 | 127744617 | PDFSTATSENSEMBLUCSC | 408 | 4 | 38826 | island | 0.03570 | 0.60708 | Not queried | 5.43 | 2.86e-02 | 1.00e+00 |
|  | 1 | 38022522 | 38022718 | PDFSTATSENSEMBLUCSC | 197 | 5 | 433 | gene | 0.76331 | 0.06046 | Not queried | 5.42 | 1.51e-01 | 1.00e+00 |
|  | 3 | 193922037 | 193922406 | PDFSTATSENSEMBLUCSC | 370 | 5 | 29740 | island | 0.01836 | 0.40112 | Not queried | 5.42 | 7.94e-03 | 1.00e+00 |
|  | 10 | 73847963 | 73848463 | PDFSTATSENSEMBLUCSC | 501 | 5 | 4612 | gene;island | 0.20133 | 0.90147 | Not queried | 5.42 | 7.94e-03 | 1.00e+00 |
|  | Y | 14774019 | 14774198 | PDFSTATSENSEMBLUCSC | 180 | 4 | 42167 | tss | 0.01982 | 0.47730 | Not queried | 5.41 | 2.86e-02 | 1.00e+00 |
|  | 19 | 50191342 | 50191882 | PDFSTATSENSEMBLUCSC | 541 | 6 | 20924 | tss;island | 0.15584 | 0.88427 | Not queried | 5.41 | 2.16e-03 | 1.00e+00 |
|  | 7 | 31288 | 31497 | PDFSTATSENSEMBLUCSC | 210 | 5 | 37962 | island | 0.04579 | 0.61482 | Not queried | 5.41 | 7.94e-03 | 1.00e+00 |
|  | 1 | 228652338 | 228652581 | PDFSTATSENSEMBLUCSC | 244 | 4 | 4289 | island | 0.04690 | 0.66470 | Not queried | 5.40 | 2.86e-02 | 1.00e+00 |
|  | 19 | 2525231 | 2525634 | PDFSTATSENSEMBLUCSC | 404 | 5 | 19655 | gene;island | 0.11105 | 0.80684 | Not queried | 5.40 | 7.94e-03 | 1.00e+00 |
|  | 20 | 57089953 | 57090317 | PDFSTATSENSEMBLUCSC | 365 | 9 | 25811 | tss | 0.04436 | 0.61360 | Not queried | 5.40 | 4.11e-05 | 2.11e-01 |
|  | 22 | 31364274 | 31364356 | PDFSTATSENSEMBLUCSC | 83 | 5 | 27029 | tss;island | 0.72786 | 0.05566 | Not queried | 5.40 | 7.94e-03 | 1.00e+00 |
|  | 7 | 156796967 | 156797410 | PDFSTATSENSEMBLUCSC | 444 | 6 | 39029 | island | 0.96932 | 0.32360 | Not queried | 5.40 | 2.16e-03 | 1.00e+00 |
|  | 22 | 17489112 | 17489367 | PDFSTATSENSEMBLUCSC | 256 | 7 | 26897 | tss;island | 0.10394 | 0.89829 | Not queried | 5.40 | 5.83e-04 | 1.00e+00 |
|  | 16 | 89440878 | 89441217 | PDFSTATSENSEMBLUCSC | 340 | 4 | 14911 | gene;island | 0.24255 | 0.88746 | Not queried | 5.39 | 1.14e-01 | 1.00e+00 |
|  | 17 | 6899085 | 6899380 | PDFSTATSENSEMBLUCSC | 296 | 9 | 17023 | tss | 0.03741 | 0.56382 | Not queried | 5.39 | 4.11e-05 | 2.11e-01 |
|  | 22 | 45680498 | 45680844 | PDFSTATSENSEMBLUCSC | 347 | 9 | 27178 | tss | 0.90286 | 0.16456 | Not queried | 5.39 | 4.11e-05 | 2.11e-01 |
|  | 4 | 53233 | 53548 | PDFSTATSENSEMBLUCSC | 316 | 5 | 29781 | gene | 0.02069 | 0.46989 | Not queried | 5.39 | 7.94e-03 | 1.00e+00 |
|  | 16 | 78601767 | 78601961 | PDFSTATSENSEMBLUCSC | 195 | 4 | 14819 | gene | 0.23665 | 0.93499 | Not queried | 5.39 | 2.86e-02 | 1.00e+00 |
|  | 11 | 7695165 | 7695528 | PDFSTATSENSEMBLUCSC | 364 | 7 | 7101 | tss;island | 0.03705 | 0.61656 | Not queried | 5.39 | 5.83e-04 | 1.00e+00 |
|  | 3 | 157261004 | 157261106 | PDFSTATSENSEMBLUCSC | 103 | 6 | 28638 | tss | 0.05086 | 0.71330 | Not queried | 5.38 | 2.16e-03 | 1.00e+00 |
|  | 5 | 139525919 | 139526226 | PDFSTATSENSEMBLUCSC | 308 | 4 | 33121 | island | 0.76434 | 0.11878 | Not queried | 5.37 | 2.86e-02 | 1.00e+00 |
|  | 11 | 57414401 | 57414626 | PDFSTATSENSEMBLUCSC | 226 | 4 | 6597 | gene;island | 0.05444 | 0.69055 | Not queried | 5.37 | 2.86e-02 | 1.00e+00 |
|  | 17 | 5094881 | 5095455 | PDFSTATSENSEMBLUCSC | 575 | 11 | 17858 | island | 0.02901 | 0.53716 | RP11-333E1.1; ZNF594 (margin: RP11-333E1.1; ZNF594) | 5.36 | 2.84e-06 | 1.46e-02 |
|  | 2 | 24232603 | 24232891 | PDFSTATSENSEMBLUCSC | 289 | 10 | 23182 | tss | 0.26393 | 0.94448 | Not queried | 5.35 | 1.08e-05 | 5.56e-02 |
|  | 5 | 114937535 | 114938459 | PDFSTATSENSEMBLUCSC | 925 | 11 | 31664 | gene;island | 0.12126 | 0.88478 | AC010226.4; TICAM2; TMED7-TICAM2 (margin: AC010226.4; TICAM2; TMED7-TICAM2; TMED7) | 5.35 | 2.84e-06 | 1.46e-02 |
|  | 17 | 46682308 | 46682413 | PDFSTATSENSEMBLUCSC | 106 | 6 | 16622 | gene;island | 0.05041 | 0.73489 | Not queried | 5.34 | 2.16e-03 | 1.00e+00 |
|  | 13 | 112725903 | 112726124 | PDFSTATSENSEMBLUCSC | 222 | 4 | 11883 | island | 0.83980 | 0.11115 | Not queried | 5.34 | 2.86e-02 | 1.00e+00 |
|  | 13 | 23489817 | 23490053 | PDFSTATSENSEMBLUCSC | 237 | 4 | 11566 | island | 0.02264 | 0.47176 | Not queried | 5.34 | 2.86e-02 | 1.00e+00 |
|  | 19 | 12267308 | 12267796 | PDFSTATSENSEMBLUCSC | 489 | 8 | 21559 | island | 0.03159 | 0.51812 | Not queried | 5.34 | 1.55e-04 | 7.99e-01 |
|  | 20 | 57089933 | 57090317 | PDFSTATSENSEMBLUCSC | 385 | 10 | 26251 | island | 0.03950 | 0.59734 | Not queried | 5.34 | 1.08e-05 | 5.56e-02 |
|  | 18 | 71815073 | 71815372 | PDFSTATSENSEMBLUCSC | 300 | 11 | 19285 | tss;island | 0.01393 | 0.37993 | TIMM21; FBXO15 (margin: TIMM21; FBXO15) | 5.34 | 2.84e-06 | 1.46e-02 |
|  | X | 46306719 | 46306863 | PDFSTATSENSEMBLUCSC | 145 | 4 | 40969 | gene | 0.03902 | 0.59825 | Not queried | 5.33 | 2.86e-02 | 1.00e+00 |
|  | 10 | 103043991 | 103044385 | PDFSTATSENSEMBLUCSC | 395 | 5 | 6047 | island | 0.86592 | 0.09068 | Not queried | 5.33 | 7.94e-03 | 1.00e+00 |
|  | 2 | 75185439 | 75185645 | PDFSTATSENSEMBLUCSC | 207 | 6 | 23429 | tss;island | 0.02718 | 0.48256 | Not queried | 5.33 | 2.16e-03 | 1.00e+00 |
|  | 19 | 20277919 | 20278059 | PDFSTATSENSEMBLUCSC | 141 | 6 | 20620 | tss | 0.73329 | 0.06211 | Not queried | 5.33 | 2.16e-03 | 1.00e+00 |
|  | 11 | 66360492 | 66360551 | PDFSTATSENSEMBLUCSC | 60 | 4 | 6715 | gene | 0.02222 | 0.45549 | Not queried | 5.33 | 2.86e-02 | 1.00e+00 |
|  | 21 | 37442104 | 37442476 | PDFSTATSENSEMBLUCSC | 373 | 4 | 26627 | island | 0.03289 | 0.56448 | Not queried | 5.32 | 2.86e-02 | 1.00e+00 |
|  | 7 | 156400711 | 156400990 | PDFSTATSENSEMBLUCSC | 280 | 4 | 39022 | island | 0.19116 | 0.91125 | Not queried | 5.32 | 2.86e-02 | 1.00e+00 |
|  | 7 | 105221826 | 105222180 | PDFSTATSENSEMBLUCSC | 355 | 8 | 38757 | island | 0.03006 | 0.53788 | Not queried | 5.32 | 1.55e-04 | 7.99e-01 |
|  | X | 70712215 | 70712810 | PDFSTATSENSEMBLUCSC | 596 | 8 | 41025 | gene;island | 0.96031 | 0.36695 | Not queried | 5.32 | 1.55e-04 | 7.99e-01 |
|  | 1 | 3634811 | 3635210 | PDFSTATSENSEMBLUCSC | 400 | 6 | 141 | gene;island | 0.02173 | 0.50013 | Not queried | 5.32 | 4.11e-02 | 1.00e+00 |
|  | 17 | 45401148 | 45401397 | PDFSTATSENSEMBLUCSC | 250 | 5 | 18420 | island | 0.02686 | 0.55826 | Not queried | 5.32 | 7.94e-03 | 1.00e+00 |
|  | 11 | 73358518 | 73358657 | PDFSTATSENSEMBLUCSC | 140 | 4 | 6804 | gene;tss | 0.41256 | 0.03387 | Not queried | 5.32 | 2.86e-02 | 1.00e+00 |
|  | 12 | 63025490 | 63025948 | PDFSTATSENSEMBLUCSC | 459 | 5 | 10558 | island | 0.62432 | 0.04018 | Not queried | 5.31 | 7.94e-03 | 1.00e+00 |
|  | X | 122866753 | 122867073 | PDFSTATSENSEMBLUCSC | 321 | 4 | 42005 | island | 0.02628 | 0.61043 | Not queried | 5.31 | 2.86e-02 | 1.00e+00 |
|  | 17 | 36719518 | 36719937 | PDFSTATSENSEMBLUCSC | 420 | 5 | 16504 | gene;island | 0.84569 | 0.14879 | Not queried | 5.31 | 7.94e-03 | 1.00e+00 |
|  | 1 | 207842833 | 207843084 | PDFSTATSENSEMBLUCSC | 252 | 4 | 976 | gene;island | 0.28445 | 0.94028 | Not queried | 5.31 | 2.86e-02 | 1.00e+00 |
|  | 3 | 44753865 | 44754587 | PDFSTATSENSEMBLUCSC | 723 | 12 | 28997 | island | 0.55584 | 0.03533 | ZNF502; SOCS5P3 (margin: ZNF502; SOCS5P3) | 5.30 | 7.40e-07 | 3.80e-03 |
|  | 4 | 8271196 | 8271507 | PDFSTATSENSEMBLUCSC | 312 | 7 | 30867 | island | 0.04791 | 0.71275 | Not queried | 5.30 | 4.08e-03 | 1.00e+00 |
|  | 5 | 140800398 | 140800983 | PDFSTATSENSEMBLUCSC | 586 | 9 | 31777 | gene;island | 0.09843 | 0.85198 | Not queried | 5.30 | 8.23e-05 | 4.23e-01 |
|  | 2 | 74153979 | 74154363 | PDFSTATSENSEMBLUCSC | 385 | 5 | 22698 | gene | 0.38850 | 0.01384 | Not queried | 5.30 | 7.94e-03 | 1.00e+00 |
|  | 5 | 140551968 | 140552600 | PDFSTATSENSEMBLUCSC | 633 | 8 | 33155 | island | 0.08111 | 0.88734 | Not queried | 5.30 | 2.07e-02 | 1.00e+00 |
|  | X | 70712619 | 70712810 | PDFSTATSENSEMBLUCSC | 192 | 5 | 41377 | tss | 0.95871 | 0.38382 | Not queried | 5.30 | 7.94e-03 | 1.00e+00 |
|  | 22 | 43253517 | 43253559 | PDFSTATSENSEMBLUCSC | 43 | 5 | 27156 | tss;island | 0.57571 | 0.01766 | Not queried | 5.30 | 7.94e-03 | 1.00e+00 |
|  | 12 | 56040025 | 56040336 | PDFSTATSENSEMBLUCSC | 312 | 5 | 10480 | island | 0.05326 | 0.65034 | Not queried | 5.29 | 7.94e-03 | 1.00e+00 |
|  | 6 | 117086850 | 117087238 | PDFSTATSENSEMBLUCSC | 389 | 8 | 36371 | island | 0.03472 | 0.62871 | Not queried | 5.29 | 1.55e-04 | 7.99e-01 |
|  | 15 | 98503714 | 98503952 | PDFSTATSENSEMBLUCSC | 239 | 11 | 14465 | island | 0.02180 | 0.46601 | ARRDC4 (margin: ARRDC4) | 5.29 | 2.84e-06 | 1.46e-02 |
|  | 16 | 5121709 | 5122251 | PDFSTATSENSEMBLUCSC | 543 | 8 | 15650 | island | 0.02279 | 0.52561 | Not queried | 5.29 | 1.55e-04 | 7.99e-01 |
|  | 16 | 5121855 | 5122251 | PDFSTATSENSEMBLUCSC | 397 | 6 | 14617 | gene | 0.02279 | 0.47849 | Not queried | 5.29 | 2.16e-03 | 1.00e+00 |
|  | 2 | 74153795 | 74154363 | PDFSTATSENSEMBLUCSC | 569 | 8 | 24384 | island | 0.40705 | 0.01616 | Not queried | 5.28 | 1.55e-04 | 7.99e-01 |
|  | 5 | 77146197 | 77146318 | PDFSTATSENSEMBLUCSC | 122 | 4 | 32843 | island | 0.92984 | 0.30043 | Not queried | 5.28 | 2.86e-02 | 1.00e+00 |
|  | 15 | 98503714 | 98503878 | PDFSTATSENSEMBLUCSC | 165 | 9 | 13893 | tss | 0.02180 | 0.46571 | Not queried | 5.28 | 4.11e-05 | 2.11e-01 |
|  | 15 | 25093244 | 25093521 | PDFSTATSENSEMBLUCSC | 278 | 4 | 13226 | gene;island | 0.89043 | 0.15319 | Not queried | 5.28 | 2.86e-02 | 1.00e+00 |
|  | 7 | 158750244 | 158750607 | PDFSTATSENSEMBLUCSC | 364 | 4 | 39094 | island | 0.09592 | 0.76919 | Not queried | 5.28 | 2.86e-02 | 1.00e+00 |
|  | 4 | 107956955 | 107957430 | PDFSTATSENSEMBLUCSC | 476 | 5 | 30035 | gene;island | 0.02886 | 0.53496 | Not queried | 5.27 | 7.94e-03 | 1.00e+00 |
|  | 17 | 7791882 | 7792191 | PDFSTATSENSEMBLUCSC | 310 | 6 | 16384 | gene;island | 0.12391 | 0.79373 | Not queried | 5.27 | 2.16e-03 | 1.00e+00 |
|  | 16 | 82045140 | 82045222 | PDFSTATSENSEMBLUCSC | 83 | 4 | 15353 | tss | 0.06416 | 0.71238 | Not queried | 5.26 | 2.86e-02 | 1.00e+00 |
|  | 10 | 134775542 | 134776050 | PDFSTATSENSEMBLUCSC | 509 | 5 | 6302 | island | 0.04993 | 0.67461 | Not queried | 5.26 | 7.94e-03 | 1.00e+00 |
|  | 5 | 176170138 | 176170795 | PDFSTATSENSEMBLUCSC | 658 | 7 | 33369 | island | 0.05230 | 0.52083 | Not queried | 5.25 | 5.83e-04 | 1.00e+00 |
|  | 1 | 74663583 | 74664160 | PDFSTATSENSEMBLUCSC | 578 | 11 | 1692 | tss;island | 0.02126 | 0.51291 | FPGT; FPGT-TNNI3K; TNNI3K; LRRIQ3 (margin: FPGT; FPGT-TNNI3K; TNNI3K; LRRIQ3) | 5.25 | 2.84e-06 | 1.46e-02 |
|  | 12 | 121688317 | 121688459 | PDFSTATSENSEMBLUCSC | 143 | 4 | 9337 | gene | 0.11272 | 0.87355 | Not queried | 5.25 | 2.86e-02 | 1.00e+00 |
|  | 13 | 20805380 | 20805679 | PDFSTATSENSEMBLUCSC | 300 | 8 | 11302 | tss | 0.63613 | 0.07719 | Not queried | 5.25 | 1.55e-04 | 7.99e-01 |
|  | 5 | 140180921 | 140181074 | PDFSTATSENSEMBLUCSC | 154 | 4 | 31737 | gene;island | 0.05731 | 0.63485 | Not queried | 5.24 | 2.86e-02 | 1.00e+00 |
|  | 2 | 200468445 | 200469026 | PDFSTATSENSEMBLUCSC | 582 | 5 | 24955 | island | 0.18162 | 0.87295 | Not queried | 5.24 | 7.94e-03 | 1.00e+00 |
|  | 19 | 12759034 | 12759156 | PDFSTATSENSEMBLUCSC | 123 | 4 | 19822 | gene;island | 0.06518 | 0.65823 | Not queried | 5.24 | 2.86e-02 | 1.00e+00 |
|  | 14 | 101175970 | 101176310 | PDFSTATSENSEMBLUCSC | 341 | 4 | 13112 | island | 0.07453 | 0.84869 | Not queried | 5.24 | 1.14e-01 | 1.00e+00 |
|  | 8 | 43132125 | 43132507 | PDFSTATSENSEMBLUCSC | 383 | 4 | 40203 | island | 0.08722 | 0.78831 | Not queried | 5.24 | 2.86e-02 | 1.00e+00 |
|  | 5 | 178487123 | 178487576 | PDFSTATSENSEMBLUCSC | 454 | 10 | 32488 | tss | 0.01117 | 0.33814 | Not queried | 5.23 | 1.08e-05 | 5.56e-02 |
|  | 16 | 89913618 | 89913972 | PDFSTATSENSEMBLUCSC | 355 | 4 | 14919 | gene;island | 0.24470 | 0.91369 | Not queried | 5.23 | 2.86e-02 | 1.00e+00 |
|  | 4 | 3873884 | 3874143 | PDFSTATSENSEMBLUCSC | 260 | 4 | 30807 | island | 0.26627 | 0.93261 | Not queried | 5.23 | 2.86e-02 | 1.00e+00 |
|  | 1 | 151103869 | 151104367 | PDFSTATSENSEMBLUCSC | 499 | 4 | 3728 | island | 0.04618 | 0.52680 | Not queried | 5.23 | 2.86e-02 | 1.00e+00 |
|  | 1 | 23521272 | 23521417 | PDFSTATSENSEMBLUCSC | 146 | 4 | 1332 | tss | 0.19284 | 0.89243 | Not queried | 5.22 | 2.86e-02 | 1.00e+00 |
|  | 11 | 123430575 | 123430949 | PDFSTATSENSEMBLUCSC | 375 | 4 | 6930 | gene | 0.05724 | 0.67401 | Not queried | 5.22 | 2.86e-02 | 1.00e+00 |
|  | 1 | 52498502 | 52498993 | PDFSTATSENSEMBLUCSC | 492 | 5 | 528 | gene;island | 0.58113 | 0.04148 | Not queried | 5.21 | 7.94e-03 | 1.00e+00 |
|  | 6 | 31164776 | 31164994 | PDFSTATSENSEMBLUCSC | 219 | 11 | 34739 | tss;island | 0.03173 | 0.57107 | Not queried | 5.21 | 1.13e-05 | 5.83e-02 |
|  | X | 11445498 | 11445818 | PDFSTATSENSEMBLUCSC | 321 | 4 | 40934 | gene;island | 0.09777 | 0.82598 | Not queried | 5.21 | 5.71e-02 | 1.00e+00 |
|  | 15 | 57998711 | 57998894 | PDFSTATSENSEMBLUCSC | 184 | 5 | 13668 | tss | 0.59031 | 0.03703 | Not queried | 5.21 | 4.21e-01 | 1.00e+00 |
|  | 6 | 117086931 | 117087238 | PDFSTATSENSEMBLUCSC | 308 | 7 | 35129 | tss | 0.03779 | 0.59565 | Not queried | 5.20 | 5.83e-04 | 1.00e+00 |
|  | 1 | 153508511 | 153508713 | PDFSTATSENSEMBLUCSC | 203 | 6 | 758 | gene | 0.31338 | 0.92518 | Not queried | 5.20 | 2.16e-03 | 1.00e+00 |
|  | 13 | 36728869 | 36729247 | PDFSTATSENSEMBLUCSC | 379 | 6 | 11637 | island | 0.02856 | 0.54651 | Not queried | 5.20 | 2.16e-03 | 1.00e+00 |
|  | 7 | 128578023 | 128578297 | PDFSTATSENSEMBLUCSC | 275 | 4 | 37155 | gene | 0.03490 | 0.59695 | Not queried | 5.20 | 2.86e-02 | 1.00e+00 |
|  | 5 | 172665707 | 172666228 | PDFSTATSENSEMBLUCSC | 522 | 4 | 33345 | island | 0.03855 | 0.65150 | Not queried | 5.19 | 2.86e-02 | 1.00e+00 |
|  | 3 | 44626492 | 44626860 | PDFSTATSENSEMBLUCSC | 369 | 4 | 27698 | gene | 0.41589 | 0.03080 | Not queried | 5.19 | 2.86e-02 | 1.00e+00 |
|  | 15 | 66947392 | 66947617 | PDFSTATSENSEMBLUCSC | 226 | 4 | 14217 | island | 0.05105 | 0.63832 | Not queried | 5.19 | 2.86e-02 | 1.00e+00 |
|  | 1 | 209957601 | 209957750 | PDFSTATSENSEMBLUCSC | 150 | 5 | 984 | gene;island | 0.03921 | 0.61169 | Not queried | 5.19 | 7.94e-03 | 1.00e+00 |
|  | 2 | 39005158 | 39005322 | PDFSTATSENSEMBLUCSC | 165 | 4 | 23276 | tss | 0.01092 | 0.31125 | Not queried | 5.18 | 2.86e-02 | 1.00e+00 |
|  | 16 | 75150456 | 75150880 | PDFSTATSENSEMBLUCSC | 425 | 8 | 16062 | island | 0.82506 | 0.06482 | Not queried | 5.18 | 6.22e-04 | 1.00e+00 |
|  | 17 | 3716214 | 3716558 | PDFSTATSENSEMBLUCSC | 345 | 6 | 16312 | gene | 0.32282 | 0.91927 | Not queried | 5.18 | 2.16e-03 | 1.00e+00 |
|  | 5 | 2753852 | 2754322 | PDFSTATSENSEMBLUCSC | 471 | 8 | 31494 | gene;island | 0.65068 | 0.05224 | Not queried | 5.18 | 1.09e-03 | 1.00e+00 |
|  | 5 | 54518469 | 54519307 | PDFSTATSENSEMBLUCSC | 839 | 7 | 32738 | island | 0.86138 | 0.07167 | Not queried | 5.18 | 5.83e-04 | 1.00e+00 |
|  | Y | 6778543 | 6778695 | PDFSTATSENSEMBLUCSC | 153 | 4 | 42166 | tss;island | 0.95581 | 0.32469 | Not queried | 5.17 | 2.86e-02 | 1.00e+00 |
|  | 19 | 35454992 | 35455389 | PDFSTATSENSEMBLUCSC | 398 | 8 | 20661 | tss | 0.54793 | 0.02517 | Not queried | 5.17 | 1.55e-04 | 7.99e-01 |
|  | 20 | 57581903 | 57582292 | PDFSTATSENSEMBLUCSC | 390 | 6 | 25440 | gene | 0.03119 | 0.56531 | Not queried | 5.17 | 2.16e-03 | 1.00e+00 |
|  | 5 | 178487123 | 178488100 | PDFSTATSENSEMBLUCSC | 978 | 14 | 33415 | island | 0.01466 | 0.37227 | Not queried | 5.17 | 4.21e-04 | 1.00e+00 |
|  | 1 | 1713944 | 1714271 | PDFSTATSENSEMBLUCSC | 328 | 5 | 2458 | island | 0.11169 | 0.82404 | Not queried | 5.17 | 7.94e-03 | 1.00e+00 |
|  | 8 | 1764878 | 1765477 | PDFSTATSENSEMBLUCSC | 600 | 12 | 39974 | island | 0.03227 | 0.53461 | MIR596 (margin: MIR596; ARHGEF10) | 5.16 | 7.40e-07 | 3.80e-03 |
|  | 15 | 76005189 | 76005446 | PDFSTATSENSEMBLUCSC | 258 | 4 | 13789 | tss | 0.04299 | 0.60868 | Not queried | 5.16 | 2.86e-02 | 1.00e+00 |
|  | 4 | 2765767 | 2766135 | PDFSTATSENSEMBLUCSC | 369 | 5 | 30771 | island | 0.14153 | 0.85446 | Not queried | 5.15 | 3.17e-02 | 1.00e+00 |
|  | 14 | 76451771 | 76452033 | PDFSTATSENSEMBLUCSC | 263 | 4 | 12495 | tss | 0.54446 | 0.04765 | Not queried | 5.15 | 1.14e-01 | 1.00e+00 |
|  | 11 | 5617703 | 5617926 | PDFSTATSENSEMBLUCSC | 224 | 5 | 6467 | gene | 0.02437 | 0.50760 | Not queried | 5.15 | 7.94e-03 | 1.00e+00 |
|  | 12 | 52627272 | 52627576 | PDFSTATSENSEMBLUCSC | 305 | 4 | 9110 | gene;island | 0.11993 | 0.81190 | Not queried | 5.15 | 2.86e-02 | 1.00e+00 |
|  | 15 | 36871420 | 36871800 | PDFSTATSENSEMBLUCSC | 381 | 5 | 13538 | tss | 0.01537 | 0.45608 | Not queried | 5.14 | 7.94e-03 | 1.00e+00 |
|  | 2 | 152118408 | 152118835 | PDFSTATSENSEMBLUCSC | 428 | 8 | 23620 | tss;island | 0.03048 | 0.52754 | Not queried | 5.13 | 4.99e-02 | 1.00e+00 |
|  | 2 | 162930175 | 162930454 | PDFSTATSENSEMBLUCSC | 280 | 4 | 22873 | gene;island | 0.02359 | 0.50376 | Not queried | 5.13 | 2.86e-02 | 1.00e+00 |
|  | 12 | 54343506 | 54343835 | PDFSTATSENSEMBLUCSC | 330 | 5 | 10453 | island | 0.02502 | 0.47936 | Not queried | 5.13 | 3.17e-02 | 1.00e+00 |
|  | 13 | 50706583 | 50707065 | PDFSTATSENSEMBLUCSC | 483 | 6 | 11714 | island | 0.47568 | 0.03176 | Not queried | 5.13 | 2.16e-03 | 1.00e+00 |
|  | 6 | 31111076 | 31111270 | PDFSTATSENSEMBLUCSC | 195 | 4 | 33792 | gene | 0.08609 | 0.74340 | Not queried | 5.13 | 2.86e-02 | 1.00e+00 |
|  | 4 | 74847646 | 74848016 | PDFSTATSENSEMBLUCSC | 371 | 8 | 31047 | island | 0.08150 | 0.84832 | Not queried | 5.12 | 1.55e-04 | 7.99e-01 |
|  | 13 | 113424938 | 113425298 | PDFSTATSENSEMBLUCSC | 361 | 6 | 11223 | gene;island | 0.08682 | 0.72233 | Not queried | 5.12 | 2.16e-03 | 1.00e+00 |
|  | 4 | 74864596 | 74864725 | PDFSTATSENSEMBLUCSC | 130 | 4 | 30385 | tss;island | 0.11110 | 0.72426 | Not queried | 5.12 | 2.86e-02 | 1.00e+00 |
|  | 16 | 31483137 | 31483534 | PDFSTATSENSEMBLUCSC | 398 | 5 | 15181 | tss;island | 0.36742 | 0.05707 | Not queried | 5.11 | 9.52e-02 | 1.00e+00 |
|  | 14 | 105173643 | 105173980 | PDFSTATSENSEMBLUCSC | 338 | 5 | 12220 | gene | 0.16342 | 0.89625 | Not queried | 5.11 | 7.94e-03 | 1.00e+00 |
|  | 12 | 130822603 | 130822818 | PDFSTATSENSEMBLUCSC | 216 | 4 | 10973 | island | 0.41364 | 0.94691 | Not queried | 5.11 | 2.86e-02 | 1.00e+00 |
|  | 16 | 51183988 | 51184583 | PDFSTATSENSEMBLUCSC | 596 | 8 | 14718 | gene;island | 0.61592 | 0.02418 | Not queried | 5.10 | 1.86e-03 | 1.00e+00 |
|  | 16 | 68270129 | 68270387 | PDFSTATSENSEMBLUCSC | 259 | 5 | 16008 | island | 0.02886 | 0.51379 | Not queried | 5.10 | 7.94e-03 | 1.00e+00 |
|  | 5 | 137610255 | 137610645 | PDFSTATSENSEMBLUCSC | 391 | 7 | 32258 | tss | 0.02524 | 0.49104 | Not queried | 5.10 | 1.17e-03 | 1.00e+00 |
|  | 2 | 31361593 | 31361818 | PDFSTATSENSEMBLUCSC | 226 | 8 | 23249 | tss;island | 0.66135 | 0.07143 | Not queried | 5.10 | 1.55e-04 | 7.99e-01 |
|  | 2 | 161127463 | 161127884 | PDFSTATSENSEMBLUCSC | 422 | 5 | 24777 | island | 0.16314 | 0.86991 | Not queried | 5.10 | 7.94e-03 | 1.00e+00 |
|  | 7 | 1121657 | 1121929 | PDFSTATSENSEMBLUCSC | 273 | 4 | 36685 | gene;island | 0.21229 | 0.89319 | Not queried | 5.10 | 2.86e-02 | 1.00e+00 |
|  | X | 148713660 | 148713830 | PDFSTATSENSEMBLUCSC | 171 | 7 | 41563 | tss;island | 0.03933 | 0.58303 | Not queried | 5.10 | 5.83e-04 | 1.00e+00 |
|  | 7 | 87848143 | 87848358 | PDFSTATSENSEMBLUCSC | 216 | 4 | 37013 | gene;island | 0.56612 | 0.04496 | Not queried | 5.10 | 2.86e-02 | 1.00e+00 |
|  | 2 | 1286216 | 1286641 | PDFSTATSENSEMBLUCSC | 426 | 5 | 22491 | gene;island | 0.07673 | 0.75063 | Not queried | 5.09 | 7.94e-03 | 1.00e+00 |
|  | 4 | 3748154 | 3748347 | PDFSTATSENSEMBLUCSC | 194 | 4 | 30804 | island | 0.11688 | 0.83903 | Not queried | 5.09 | 2.86e-02 | 1.00e+00 |
|  | 13 | 20805094 | 20805679 | PDFSTATSENSEMBLUCSC | 586 | 10 | 11068 | gene;island | 0.67702 | 0.12682 | Not queried | 5.09 | 1.08e-05 | 5.56e-02 |
|  | 6 | 2842146 | 2842550 | PDFSTATSENSEMBLUCSC | 405 | 7 | 34527 | tss | 0.08252 | 0.72869 | Not queried | 5.09 | 2.62e-02 | 1.00e+00 |
|  | 6 | 2841875 | 2842550 | PDFSTATSENSEMBLUCSC | 676 | 9 | 35313 | island | 0.08252 | 0.70478 | Not queried | 5.09 | 3.99e-03 | 1.00e+00 |
|  | 6 | 151560831 | 151561055 | PDFSTATSENSEMBLUCSC | 225 | 7 | 35234 | tss | 0.69047 | 0.12180 | Not queried | 5.09 | 5.83e-04 | 1.00e+00 |
|  | 16 | 85362963 | 85363209 | PDFSTATSENSEMBLUCSC | 247 | 4 | 16119 | island | 0.48562 | 0.03669 | Not queried | 5.08 | 2.86e-02 | 1.00e+00 |
|  | X | 73164102 | 73164145 | PDFSTATSENSEMBLUCSC | 44 | 4 | 41389 | tss | 0.02693 | 0.39853 | Not queried | 5.08 | 2.86e-02 | 1.00e+00 |
|  | 10 | 50886878 | 50887212 | PDFSTATSENSEMBLUCSC | 335 | 5 | 5058 | tss;island | 0.19102 | 0.78274 | Not queried | 5.08 | 7.94e-03 | 1.00e+00 |
|  | 15 | 89921672 | 89922071 | PDFSTATSENSEMBLUCSC | 400 | 4 | 13439 | gene;island | 0.19201 | 0.87603 | Not queried | 5.07 | 2.86e-02 | 1.00e+00 |
|  | 6 | 66497586 | 66498032 | PDFSTATSENSEMBLUCSC | 447 | 4 | 36169 | island | 0.08101 | 0.69084 | Not queried | 5.07 | 2.86e-02 | 1.00e+00 |
|  | 10 | 50506748 | 50507092 | PDFSTATSENSEMBLUCSC | 345 | 7 | 5054 | tss | 0.08385 | 0.80601 | Not queried | 5.07 | 2.33e-03 | 1.00e+00 |
|  | 21 | 47394354 | 47394659 | PDFSTATSENSEMBLUCSC | 306 | 4 | 26723 | island | 0.04576 | 0.59328 | Not queried | 5.07 | 2.86e-02 | 1.00e+00 |
|  | 8 | 27182871 | 27183342 | PDFSTATSENSEMBLUCSC | 472 | 8 | 39192 | gene;island | 0.03322 | 0.57475 | Not queried | 5.06 | 1.55e-04 | 7.99e-01 |
|  | 3 | 157261004 | 157261273 | PDFSTATSENSEMBLUCSC | 270 | 8 | 29571 | island | 0.06279 | 0.71330 | Not queried | 5.06 | 1.55e-04 | 7.99e-01 |
|  | 19 | 59074265 | 59074507 | PDFSTATSENSEMBLUCSC | 243 | 4 | 20228 | gene;island | 0.04060 | 0.58931 | Not queried | 5.06 | 2.86e-02 | 1.00e+00 |
|  | 10 | 27541213 | 27541418 | PDFSTATSENSEMBLUCSC | 206 | 5 | 5609 | island | 0.08830 | 0.90683 | Not queried | 5.06 | 7.94e-03 | 1.00e+00 |
|  | 6 | 111408745 | 111408765 | PDFSTATSENSEMBLUCSC | 21 | 5 | 35111 | tss | 0.53416 | 0.03326 | Not queried | 5.06 | 7.94e-03 | 1.00e+00 |
|  | 5 | 157098338 | 157098555 | PDFSTATSENSEMBLUCSC | 218 | 6 | 32398 | tss | 0.04443 | 0.59059 | Not queried | 5.06 | 2.16e-03 | 1.00e+00 |
|  | 2 | 198651303 | 198651576 | PDFSTATSENSEMBLUCSC | 274 | 4 | 23765 | tss;island | 0.03292 | 0.50989 | Not queried | 5.06 | 2.86e-02 | 1.00e+00 |
|  | 2 | 235406275 | 235406540 | PDFSTATSENSEMBLUCSC | 266 | 4 | 23923 | tss;island | 0.79686 | 0.10859 | Not queried | 5.06 | 5.71e-02 | 1.00e+00 |
|  | X | 73642618 | 73642885 | PDFSTATSENSEMBLUCSC | 268 | 4 | 41037 | gene;island | 0.60737 | 0.04548 | Not queried | 5.04 | 2.86e-02 | 1.00e+00 |
|  | 22 | 35937058 | 35937531 | PDFSTATSENSEMBLUCSC | 474 | 8 | 27405 | island | 0.63018 | 0.04151 | Not queried | 5.04 | 6.22e-04 | 1.00e+00 |
|  | 12 | 54410251 | 54410632 | PDFSTATSENSEMBLUCSC | 382 | 12 | 9729 | tss | 0.02116 | 0.35661 | HOXC5; HOXC6; HOXC4 (margin: HOXC5; HOXC6; HOXC8; HOXC4; AC012531.25) | 5.04 | 5.18e-06 | 2.66e-02 |
|  | 16 | 68270252 | 68270387 | PDFSTATSENSEMBLUCSC | 136 | 4 | 15288 | tss | 0.03348 | 0.53686 | Not queried | 5.03 | 2.86e-02 | 1.00e+00 |
|  | 14 | 104551867 | 104552397 | PDFSTATSENSEMBLUCSC | 531 | 6 | 13170 | island | 0.29586 | 0.93061 | Not queried | 5.03 | 2.16e-03 | 1.00e+00 |
|  | 5 | 38445522 | 38445916 | PDFSTATSENSEMBLUCSC | 395 | 7 | 31539 | gene | 0.04780 | 0.61240 | Not queried | 5.03 | 5.83e-04 | 1.00e+00 |
|  | 17 | 79393463 | 79393718 | PDFSTATSENSEMBLUCSC | 256 | 5 | 16875 | gene;island | 0.21180 | 0.78186 | Not queried | 5.03 | 7.94e-03 | 1.00e+00 |
|  | 16 | 14397576 | 14397766 | PDFSTATSENSEMBLUCSC | 191 | 5 | 15070 | tss | 0.43334 | 0.03145 | Not queried | 5.03 | 7.94e-03 | 1.00e+00 |
|  | 5 | 140723331 | 140723583 | PDFSTATSENSEMBLUCSC | 253 | 6 | 32314 | tss | 0.19234 | 0.84771 | Not queried | 5.02 | 2.16e-03 | 1.00e+00 |
|  | 6 | 27839751 | 27840068 | PDFSTATSENSEMBLUCSC | 318 | 4 | 33559 | gene | 0.22634 | 0.90049 | Not queried | 5.02 | 2.86e-02 | 1.00e+00 |
|  | 3 | 172166182 | 172166258 | PDFSTATSENSEMBLUCSC | 77 | 4 | 29622 | island | 0.12926 | 0.86002 | Not queried | 5.02 | 2.86e-02 | 1.00e+00 |
|  | 8 | 22960177 | 22960619 | PDFSTATSENSEMBLUCSC | 443 | 10 | 40081 | island | 0.04635 | 0.56908 | Not queried | 5.01 | 2.88e-03 | 1.00e+00 |
|  | 20 | 61148764 | 61149009 | PDFSTATSENSEMBLUCSC | 246 | 7 | 25835 | tss | 0.16365 | 0.87094 | Not queried | 5.00 | 5.83e-04 | 1.00e+00 |
|  | 5 | 38445522 | 38445614 | PDFSTATSENSEMBLUCSC | 93 | 4 | 31970 | tss | 0.09368 | 0.59260 | Not queried | 5.00 | 2.86e-02 | 1.00e+00 |
|  | 17 | 33775295 | 33775779 | PDFSTATSENSEMBLUCSC | 485 | 5 | 16485 | gene | 0.17234 | 0.86962 | Not queried | 5.00 | 7.94e-03 | 1.00e+00 |
|  | 2 | 162930666 | 162931175 | PDFSTATSENSEMBLUCSC | 510 | 5 | 24787 | island | 0.02912 | 0.45262 | Not queried | 5.00 | 7.94e-03 | 1.00e+00 |
|  | 5 | 76934327 | 76934696 | PDFSTATSENSEMBLUCSC | 370 | 6 | 32839 | island | 0.48674 | 0.02617 | Not queried | 5.00 | 4.33e-03 | 1.00e+00 |
|  | 3 | 55508379 | 55508686 | PDFSTATSENSEMBLUCSC | 308 | 4 | 27795 | gene;island | 0.12732 | 0.90904 | Not queried | 5.00 | 2.86e-02 | 1.00e+00 |
|  | 6 | 167508001 | 167508226 | PDFSTATSENSEMBLUCSC | 226 | 6 | 36580 | island | 0.31775 | 0.91141 | Not queried | 5.00 | 8.66e-03 | 1.00e+00 |
|  | 8 | 24771273 | 24771645 | PDFSTATSENSEMBLUCSC | 373 | 4 | 39182 | gene | 0.11955 | 0.71503 | Not queried | 5.00 | 2.86e-02 | 1.00e+00 |
|  | 3 | 42306385 | 42306974 | PDFSTATSENSEMBLUCSC | 590 | 8 | 28969 | island | 0.06999 | 0.75632 | Not queried | 5.00 | 1.55e-04 | 7.99e-01 |
|  | 7 | 27134109 | 27134369 | PDFSTATSENSEMBLUCSC | 261 | 4 | 36863 | gene;island | 0.84593 | 0.19557 | Not queried | 4.99 | 2.86e-02 | 1.00e+00 |
|  | 16 | 90113893 | 90114173 | PDFSTATSENSEMBLUCSC | 281 | 4 | 14925 | gene | 0.03378 | 0.52693 | Not queried | 4.99 | 2.86e-02 | 1.00e+00 |
|  | 1 | 156163755 | 156164205 | PDFSTATSENSEMBLUCSC | 451 | 8 | 814 | gene | 0.02517 | 0.38752 | Not queried | 4.99 | 1.55e-04 | 7.99e-01 |
|  | 12 | 131438523 | 131438788 | PDFSTATSENSEMBLUCSC | 266 | 6 | 9394 | gene | 0.10806 | 0.81799 | Not queried | 4.99 | 2.16e-03 | 1.00e+00 |
|  | 2 | 91777099 | 91777482 | PDFSTATSENSEMBLUCSC | 384 | 4 | 24470 | island | 0.15980 | 0.87096 | Not queried | 4.99 | 2.86e-02 | 1.00e+00 |
|  | 15 | 36871420 | 36871852 | PDFSTATSENSEMBLUCSC | 433 | 7 | 13979 | island | 0.02049 | 0.40110 | Not queried | 4.99 | 5.83e-04 | 1.00e+00 |
|  | 8 | 143695822 | 143696144 | PDFSTATSENSEMBLUCSC | 323 | 7 | 40573 | island | 0.06485 | 0.56400 | Not queried | 4.99 | 5.83e-04 | 1.00e+00 |
|  | 22 | 45680498 | 45681036 | PDFSTATSENSEMBLUCSC | 539 | 12 | 27531 | island | 0.87809 | 0.17172 | UPK3A (margin: CTA-268H5.9; UPK3A) | 4.98 | 7.40e-07 | 3.80e-03 |
|  | 12 | 54410251 | 54410727 | PDFSTATSENSEMBLUCSC | 477 | 17 | 10467 | island | 0.01517 | 0.30179 | HOXC5; HOXC6; HOXC4 (margin: HOXC5; HOXC6; HOXC8; HOXC4; AC012531.25) | 4.98 | 6.00e-09 | 3.08e-05 |
|  | 12 | 54410657 | 54410727 | PDFSTATSENSEMBLUCSC | 71 | 5 | 9133 | gene | 0.00703 | 0.21760 | Not queried | 4.98 | 7.94e-03 | 1.00e+00 |
|  | X | 153990478 | 153991165 | PDFSTATSENSEMBLUCSC | 688 | 10 | 42154 | island | 0.02604 | 0.54607 | Not queried | 4.98 | 1.05e-03 | 1.00e+00 |
|  | X | 23350313 | 23350651 | PDFSTATSENSEMBLUCSC | 339 | 4 | 41684 | island | 0.49805 | 0.02660 | Not queried | 4.98 | 2.86e-02 | 1.00e+00 |
|  | 4 | 113432595 | 113432911 | PDFSTATSENSEMBLUCSC | 317 | 4 | 31201 | island | 0.61177 | 0.04168 | Not queried | 4.98 | 2.86e-02 | 1.00e+00 |
|  | 2 | 9614471 | 9614744 | PDFSTATSENSEMBLUCSC | 274 | 7 | 24018 | island | 0.43700 | 0.90900 | Not queried | 4.98 | 5.83e-04 | 1.00e+00 |
|  | 17 | 46608277 | 46608570 | PDFSTATSENSEMBLUCSC | 294 | 9 | 17423 | tss | 0.83132 | 0.08350 | Not queried | 4.98 | 1.65e-04 | 8.46e-01 |
|  | 19 | 38754529 | 38754930 | PDFSTATSENSEMBLUCSC | 402 | 5 | 20712 | tss | 0.04250 | 0.55001 | Not queried | 4.97 | 7.94e-03 | 1.00e+00 |
|  | 1 | 111506233 | 111506560 | PDFSTATSENSEMBLUCSC | 328 | 6 | 677 | gene | 0.01721 | 0.33124 | Not queried | 4.97 | 2.16e-03 | 1.00e+00 |
|  | 20 | 57581903 | 57582371 | PDFSTATSENSEMBLUCSC | 469 | 7 | 26259 | island | 0.03205 | 0.56709 | Not queried | 4.97 | 5.83e-04 | 1.00e+00 |
|  | 10 | 38691787 | 38691801 | PDFSTATSENSEMBLUCSC | 15 | 4 | 5020 | tss;island | 0.02971 | 0.52951 | Not queried | 4.97 | 2.86e-02 | 1.00e+00 |
|  | 8 | 24771114 | 24771645 | PDFSTATSENSEMBLUCSC | 532 | 7 | 39540 | tss;island | 0.12077 | 0.71551 | Not queried | 4.97 | 5.83e-04 | 1.00e+00 |
|  | 16 | 80574553 | 80574757 | PDFSTATSENSEMBLUCSC | 205 | 4 | 15344 | tss | 0.01387 | 0.39083 | Not queried | 4.97 | 2.86e-02 | 1.00e+00 |
|  | 6 | 291687 | 291909 | PDFSTATSENSEMBLUCSC | 223 | 4 | 34520 | tss;island | 0.02223 | 0.47868 | Not queried | 4.96 | 2.86e-02 | 1.00e+00 |
|  | 8 | 11559039 | 11559488 | PDFSTATSENSEMBLUCSC | 450 | 4 | 40021 | island | 0.94472 | 0.44559 | Not queried | 4.96 | 2.00e-01 | 1.00e+00 |
|  | 11 | 47270041 | 47270669 | PDFSTATSENSEMBLUCSC | 629 | 14 | 6586 | gene;tss;island | 0.02928 | 0.45909 | NR1H3; ACP2 (margin: DDB2; NR1H3; ACP2) | 4.96 | 4.99e-08 | 2.56e-04 |
|  | 6 | 85482570 | 85483055 | PDFSTATSENSEMBLUCSC | 486 | 5 | 36236 | island | 0.01750 | 0.10203 | Not queried | 4.96 | 3.17e-02 | 1.00e+00 |
|  | X | 17755531 | 17755839 | PDFSTATSENSEMBLUCSC | 309 | 6 | 41669 | island | 0.25757 | 0.01468 | Not queried | 4.95 | 2.16e-03 | 1.00e+00 |
|  | 16 | 30198370 | 30198517 | PDFSTATSENSEMBLUCSC | 148 | 4 | 14688 | gene;island | 0.12803 | 0.70923 | Not queried | 4.95 | 5.71e-02 | 1.00e+00 |
|  | 12 | 54088760 | 54088972 | PDFSTATSENSEMBLUCSC | 213 | 4 | 10444 | island | 0.70431 | 0.09034 | Not queried | 4.95 | 2.86e-02 | 1.00e+00 |
|  | 2 | 162930666 | 162931003 | PDFSTATSENSEMBLUCSC | 338 | 4 | 22874 | gene | 0.03566 | 0.50837 | Not queried | 4.94 | 2.86e-02 | 1.00e+00 |
|  | 6 | 27064705 | 27065003 | PDFSTATSENSEMBLUCSC | 299 | 4 | 35482 | island | 0.02443 | 0.44102 | Not queried | 4.94 | 2.86e-02 | 1.00e+00 |
|  | 2 | 109647956 | 109648208 | PDFSTATSENSEMBLUCSC | 253 | 4 | 24566 | island | 0.49103 | 0.03005 | Not queried | 4.94 | 2.86e-02 | 1.00e+00 |
|  | 10 | 29698152 | 29698470 | PDFSTATSENSEMBLUCSC | 319 | 8 | 4991 | tss;island | 0.30536 | 0.94316 | Not queried | 4.94 | 1.04e-02 | 1.00e+00 |
|  | 16 | 1030383 | 1030619 | PDFSTATSENSEMBLUCSC | 237 | 8 | 14955 | tss;island | 0.63301 | 0.04742 | Not queried | 4.94 | 1.55e-04 | 7.99e-01 |
|  | 1 | 111506233 | 111507160 | PDFSTATSENSEMBLUCSC | 928 | 17 | 3576 | island | 0.01602 | 0.31724 | LRIF1 (margin: RP11-96K19.2; LRIF1) | 4.94 | 8.57e-10 | 4.40e-06 |
|  | 19 | 36822311 | 36822770 | PDFSTATSENSEMBLUCSC | 460 | 6 | 21902 | island | 0.31672 | 0.01191 | Not queried | 4.93 | 2.16e-03 | 1.00e+00 |
|  | 16 | 30572739 | 30573013 | PDFSTATSENSEMBLUCSC | 275 | 5 | 15804 | island | 0.34318 | 0.94746 | Not queried | 4.93 | 7.94e-03 | 1.00e+00 |
|  | X | 138773906 | 138774368 | PDFSTATSENSEMBLUCSC | 463 | 5 | 41128 | gene;island | 0.03831 | 0.61008 | Not queried | 4.93 | 7.94e-03 | 1.00e+00 |
|  | 7 | 74020711 | 74021054 | PDFSTATSENSEMBLUCSC | 344 | 5 | 38516 | island | 0.06315 | 0.67253 | Not queried | 4.93 | 7.94e-03 | 1.00e+00 |
|  | 12 | 54378736 | 54379056 | PDFSTATSENSEMBLUCSC | 321 | 8 | 10459 | island | 0.01768 | 0.34366 | Not queried | 4.93 | 6.22e-04 | 1.00e+00 |
|  | 16 | 33483244 | 33483591 | PDFSTATSENSEMBLUCSC | 348 | 5 | 15856 | island | 0.09168 | 0.72276 | Not queried | 4.93 | 7.94e-03 | 1.00e+00 |
|  | 2 | 228736089 | 228736459 | PDFSTATSENSEMBLUCSC | 371 | 9 | 25131 | island | 0.02907 | 0.46405 | Not queried | 4.93 | 4.11e-05 | 2.11e-01 |
|  | 3 | 112359488 | 112359652 | PDFSTATSENSEMBLUCSC | 165 | 6 | 27854 | gene | 0.12764 | 0.80963 | Not queried | 4.92 | 2.16e-03 | 1.00e+00 |
|  | 8 | 24813866 | 24814137 | PDFSTATSENSEMBLUCSC | 272 | 4 | 40097 | island | 0.02576 | 0.45845 | Not queried | 4.92 | 2.86e-02 | 1.00e+00 |
|  | 15 | 74658547 | 74658653 | PDFSTATSENSEMBLUCSC | 107 | 4 | 13393 | gene;island | 0.04869 | 0.49971 | Not queried | 4.92 | 2.86e-02 | 1.00e+00 |
|  | 19 | 34174665 | 34175554 | PDFSTATSENSEMBLUCSC | 890 | 21 | 19939 | gene;island | 0.08304 | 0.73978 | CHST8 (margin: CHST8) | 4.92 | 1.29e-06 | 6.63e-03 |
|  | 15 | 57668039 | 57668261 | PDFSTATSENSEMBLUCSC | 223 | 4 | 13666 | tss;island | 0.93002 | 0.19687 | Not queried | 4.91 | 1.14e-01 | 1.00e+00 |
|  | 12 | 49110781 | 49110861 | PDFSTATSENSEMBLUCSC | 81 | 6 | 9647 | tss | 0.36807 | 0.02198 | Not queried | 4.91 | 2.16e-03 | 1.00e+00 |
|  | X | 101853857 | 101854594 | PDFSTATSENSEMBLUCSC | 738 | 13 | 41917 | island | 0.02866 | 0.58891 | Not queried | 4.91 | 7.95e-04 | 1.00e+00 |
|  | 8 | 48172283 | 48172992 | PDFSTATSENSEMBLUCSC | 710 | 8 | 39624 | tss;island | 0.38817 | 0.02241 | Not queried | 4.91 | 1.55e-04 | 7.99e-01 |
|  | 5 | 157098338 | 157098619 | PDFSTATSENSEMBLUCSC | 282 | 7 | 33283 | island | 0.04908 | 0.58845 | Not queried | 4.91 | 5.83e-04 | 1.00e+00 |
|  | 1 | 166890293 | 166890436 | PDFSTATSENSEMBLUCSC | 144 | 4 | 869 | gene;island | 0.02190 | 0.43800 | Not queried | 4.91 | 2.86e-02 | 1.00e+00 |
|  | 7 | 38350921 | 38351226 | PDFSTATSENSEMBLUCSC | 306 | 5 | 38358 | island | 0.28084 | 0.90684 | Not queried | 4.91 | 7.94e-03 | 1.00e+00 |
|  | 22 | 22862802 | 22863293 | PDFSTATSENSEMBLUCSC | 492 | 5 | 26766 | gene;island | 0.56317 | 0.04384 | Not queried | 4.91 | 7.94e-03 | 1.00e+00 |
|  | 20 | 61148178 | 61149009 | PDFSTATSENSEMBLUCSC | 832 | 12 | 25449 | gene;island | 0.14751 | 0.82802 | C20orf166; C20orf166-AS1 (margin: C20orf166; MIR1-1; C20orf166-AS1) | 4.90 | 7.40e-07 | 3.80e-03 |
|  | 1 | 233086372 | 233086622 | PDFSTATSENSEMBLUCSC | 251 | 4 | 1079 | gene | 0.37201 | 0.02550 | Not queried | 4.90 | 2.86e-02 | 1.00e+00 |
|  | 4 | 4228571 | 4228650 | PDFSTATSENSEMBLUCSC | 80 | 4 | 30808 | island | 0.14744 | 0.72326 | Not queried | 4.90 | 2.86e-02 | 1.00e+00 |
|  | 5 | 80597143 | 80597828 | PDFSTATSENSEMBLUCSC | 686 | 13 | 31610 | gene;island | 0.44632 | 0.02171 | ZCCHC9; CTC-281B15.1 (margin: ZCCHC9; CTC-281B15.1) | 4.90 | 1.92e-07 | 9.88e-04 |
|  | 14 | 45722617 | 45722745 | PDFSTATSENSEMBLUCSC | 129 | 6 | 12346 | tss | 0.01784 | 0.39791 | Not queried | 4.90 | 2.16e-03 | 1.00e+00 |
|  | 12 | 2800055 | 2800500 | PDFSTATSENSEMBLUCSC | 446 | 8 | 8982 | gene;island | 0.49089 | 0.04000 | Not queried | 4.90 | 1.55e-04 | 7.99e-01 |
|  | 1 | 39546511 | 39546688 | PDFSTATSENSEMBLUCSC | 178 | 4 | 1517 | tss;island | 0.70300 | 0.06981 | Not queried | 4.90 | 2.86e-02 | 1.00e+00 |
|  | 19 | 36246395 | 36246408 | PDFSTATSENSEMBLUCSC | 14 | 4 | 19958 | gene;island | 0.18921 | 0.89072 | Not queried | 4.90 | 2.86e-02 | 1.00e+00 |
|  | X | 108868237 | 108868465 | PDFSTATSENSEMBLUCSC | 229 | 4 | 41956 | island | 0.19841 | 0.84814 | Not queried | 4.89 | 2.86e-02 | 1.00e+00 |
|  | 8 | 1796429 | 1796676 | PDFSTATSENSEMBLUCSC | 248 | 4 | 39116 | gene;island | 0.27243 | 0.91410 | Not queried | 4.89 | 2.86e-02 | 1.00e+00 |
|  | 7 | 27187372 | 27187691 | PDFSTATSENSEMBLUCSC | 320 | 8 | 37428 | tss | 0.03858 | 0.59822 | Not queried | 4.89 | 1.55e-04 | 7.99e-01 |
|  | 6 | 50818685 | 50819236 | PDFSTATSENSEMBLUCSC | 552 | 4 | 36138 | island | 0.89086 | 0.24117 | Not queried | 4.89 | 2.86e-02 | 1.00e+00 |
|  | 7 | 128577593 | 128578297 | PDFSTATSENSEMBLUCSC | 705 | 13 | 38839 | island | 0.04883 | 0.63166 | Not queried | 4.88 | 4.93e-04 | 1.00e+00 |
|  | 1 | 240656217 | 240656737 | PDFSTATSENSEMBLUCSC | 521 | 6 | 1103 | gene;island | 0.05184 | 0.55862 | Not queried | 4.88 | 2.16e-03 | 1.00e+00 |
|  | 17 | 17910235 | 17910425 | PDFSTATSENSEMBLUCSC | 191 | 4 | 16434 | gene;island | 0.05758 | 0.66351 | Not queried | 4.88 | 2.86e-02 | 1.00e+00 |
|  | 19 | 11529133 | 11530065 | PDFSTATSENSEMBLUCSC | 933 | 11 | 21532 | island | 0.77825 | 0.04542 | Not queried | 4.88 | 2.45e-03 | 1.00e+00 |
|  | 7 | 22539703 | 22539822 | PDFSTATSENSEMBLUCSC | 120 | 4 | 36849 | gene | 0.02233 | 0.24140 | Not queried | 4.88 | 2.86e-02 | 1.00e+00 |
|  | 16 | 2801668 | 2801952 | PDFSTATSENSEMBLUCSC | 285 | 5 | 14583 | gene;tss;island | 0.68681 | 0.05416 | Not queried | 4.88 | 7.94e-03 | 1.00e+00 |
|  | 12 | 11422897 | 11423120 | PDFSTATSENSEMBLUCSC | 224 | 4 | 9541 | tss | 0.05582 | 0.60120 | Not queried | 4.87 | 5.71e-02 | 1.00e+00 |
|  | 16 | 23765810 | 23766116 | PDFSTATSENSEMBLUCSC | 307 | 5 | 15737 | island | 0.06244 | 0.66120 | Not queried | 4.87 | 7.94e-03 | 1.00e+00 |
|  | 4 | 113970617 | 113970935 | PDFSTATSENSEMBLUCSC | 319 | 6 | 30056 | gene | 0.76709 | 0.09501 | Not queried | 4.87 | 2.16e-03 | 1.00e+00 |
|  | 19 | 11784246 | 11784774 | PDFSTATSENSEMBLUCSC | 529 | 9 | 20443 | tss | 0.02711 | 0.47910 | Not queried | 4.86 | 4.94e-04 | 1.00e+00 |
|  | 10 | 72141375 | 72141625 | PDFSTATSENSEMBLUCSC | 251 | 4 | 4604 | gene;island | 0.86035 | 0.14899 | Not queried | 4.85 | 2.86e-02 | 1.00e+00 |
|  | 12 | 49110427 | 49110861 | PDFSTATSENSEMBLUCSC | 435 | 10 | 10346 | island | 0.38376 | 0.02198 | Not queried | 4.85 | 1.08e-05 | 5.56e-02 |
|  | 8 | 11555178 | 11555548 | PDFSTATSENSEMBLUCSC | 371 | 4 | 40020 | island | 0.68799 | 0.09130 | Not queried | 4.85 | 2.86e-02 | 1.00e+00 |
|  | 8 | 144640306 | 144640403 | PDFSTATSENSEMBLUCSC | 98 | 6 | 39888 | tss | 0.18166 | 0.86626 | Not queried | 4.84 | 2.16e-03 | 1.00e+00 |
|  | X | 101853857 | 101854377 | PDFSTATSENSEMBLUCSC | 521 | 11 | 41431 | tss | 0.04539 | 0.58891 | Not queried | 4.84 | 5.24e-03 | 1.00e+00 |
|  | 2 | 45397776 | 45398036 | PDFSTATSENSEMBLUCSC | 261 | 4 | 24232 | island | 0.90480 | 0.18618 | Not queried | 4.84 | 2.86e-02 | 1.00e+00 |
|  | 19 | 35454891 | 35455389 | PDFSTATSENSEMBLUCSC | 499 | 10 | 21858 | island | 0.54793 | 0.03754 | Not queried | 4.84 | 1.08e-05 | 5.56e-02 |
|  | 5 | 172661803 | 172662463 | PDFSTATSENSEMBLUCSC | 661 | 8 | 33343 | island | 0.12725 | 0.87347 | Not queried | 4.83 | 1.55e-04 | 7.99e-01 |
|  | 3 | 124448994 | 124449432 | PDFSTATSENSEMBLUCSC | 439 | 7 | 29374 | island | 0.57160 | 0.04655 | Not queried | 4.83 | 5.83e-04 | 1.00e+00 |
|  | 4 | 1049609 | 1049922 | PDFSTATSENSEMBLUCSC | 314 | 4 | 30717 | island | 0.09937 | 0.82922 | Not queried | 4.83 | 5.71e-02 | 1.00e+00 |
|  | 16 | 88717374 | 88717989 | PDFSTATSENSEMBLUCSC | 616 | 13 | 16176 | island | 0.78964 | 0.09482 | CYBA; MVD (margin: IL17C; SNAI3-AS1; CYBA; MVD) | 4.83 | 3.85e-07 | 1.98e-03 |
|  | 10 | 88730324 | 88730599 | PDFSTATSENSEMBLUCSC | 276 | 4 | 4650 | gene;island | 0.42144 | 0.93714 | Not queried | 4.83 | 2.86e-02 | 1.00e+00 |
|  | 8 | 144640306 | 144640755 | PDFSTATSENSEMBLUCSC | 450 | 9 | 39420 | gene;island | 0.16314 | 0.82852 | Not queried | 4.82 | 4.11e-05 | 2.11e-01 |
|  | 8 | 27182871 | 27182965 | PDFSTATSENSEMBLUCSC | 95 | 5 | 39549 | tss | 0.03169 | 0.53656 | Not queried | 4.82 | 7.94e-03 | 1.00e+00 |
|  | 1 | 33592870 | 33593154 | PDFSTATSENSEMBLUCSC | 285 | 5 | 3002 | island | 0.13170 | 0.89095 | Not queried | 4.82 | 7.94e-03 | 1.00e+00 |
|  | 11 | 58912052 | 58912327 | PDFSTATSENSEMBLUCSC | 276 | 6 | 6601 | gene;island | 0.70944 | 0.11941 | Not queried | 4.82 | 2.16e-03 | 1.00e+00 |
|  | 19 | 12267592 | 12267796 | PDFSTATSENSEMBLUCSC | 205 | 6 | 20454 | tss | 0.03679 | 0.47268 | Not queried | 4.82 | 2.16e-03 | 1.00e+00 |
|  | 5 | 63257554 | 63257941 | PDFSTATSENSEMBLUCSC | 388 | 9 | 32028 | tss | 0.07176 | 0.59787 | Not queried | 4.82 | 4.11e-05 | 2.11e-01 |
|  | 14 | 80328091 | 80328595 | PDFSTATSENSEMBLUCSC | 505 | 5 | 12132 | gene;island | 0.32891 | 0.92483 | Not queried | 4.82 | 3.17e-02 | 1.00e+00 |
|  | 13 | 69558928 | 69559303 | PDFSTATSENSEMBLUCSC | 376 | 4 | 11741 | island | 0.08541 | 0.72925 | Not queried | 4.82 | 2.86e-02 | 1.00e+00 |
|  | 2 | 172378774 | 172379087 | PDFSTATSENSEMBLUCSC | 314 | 4 | 24816 | island | 0.47261 | 0.02671 | Not queried | 4.82 | 2.86e-02 | 1.00e+00 |
|  | 19 | 54676948 | 54677232 | PDFSTATSENSEMBLUCSC | 285 | 6 | 21019 | tss | 0.05519 | 0.51226 | Not queried | 4.81 | 1.52e-02 | 1.00e+00 |
|  | 7 | 27186993 | 27187691 | PDFSTATSENSEMBLUCSC | 699 | 11 | 38285 | island | 0.05266 | 0.60707 | Not queried | 4.81 | 8.51e-05 | 4.37e-01 |
|  | 19 | 12969204 | 12969611 | PDFSTATSENSEMBLUCSC | 408 | 5 | 19834 | gene;island | 0.27616 | 0.91550 | Not queried | 4.81 | 7.94e-03 | 1.00e+00 |
|  | 11 | 124823738 | 124823917 | PDFSTATSENSEMBLUCSC | 180 | 7 | 7766 | tss;island | 0.01361 | 0.27852 | Not queried | 4.81 | 5.83e-04 | 1.00e+00 |
|  | 16 | 745663 | 745765 | PDFSTATSENSEMBLUCSC | 103 | 4 | 14510 | gene;island | 0.05459 | 0.53827 | Not queried | 4.81 | 2.86e-02 | 1.00e+00 |
|  | 17 | 14207530 | 14207968 | PDFSTATSENSEMBLUCSC | 439 | 5 | 16415 | gene;island | 0.54779 | 0.07530 | Not queried | 4.81 | 5.56e-02 | 1.00e+00 |
|  | 11 | 2019436 | 2020560 | PDFSTATSENSEMBLUCSC | 1125 | 36 | 7035 | tss;island | 0.02256 | 0.46131 | H19 (margin: AC051649.6; MRPL23-AS1; H19) | 4.81 | 9.15e-13 | 4.70e-09 |
|  | 17 | 46688182 | 46688329 | PDFSTATSENSEMBLUCSC | 148 | 4 | 16624 | gene | 0.00602 | 0.09668 | Not queried | 4.80 | 2.86e-02 | 1.00e+00 |
|  | 17 | 37895771 | 37896175 | PDFSTATSENSEMBLUCSC | 405 | 5 | 16521 | gene | 0.02879 | 0.33431 | Not queried | 4.80 | 3.17e-02 | 1.00e+00 |
|  | 11 | 68924577 | 68924956 | PDFSTATSENSEMBLUCSC | 380 | 5 | 8564 | island | 0.12108 | 0.81873 | Not queried | 4.79 | 7.94e-03 | 1.00e+00 |
|  | 5 | 533963 | 534332 | PDFSTATSENSEMBLUCSC | 370 | 4 | 32528 | island | 0.13286 | 0.85909 | Not queried | 4.79 | 2.86e-02 | 1.00e+00 |
|  | 12 | 131303478 | 131303774 | PDFSTATSENSEMBLUCSC | 297 | 4 | 9393 | gene;island | 0.04748 | 0.24729 | Not queried | 4.79 | 2.86e-02 | 1.00e+00 |
|  | 11 | 2721207 | 2722713 | PDFSTATSENSEMBLUCSC | 1507 | 41 | 6448 | gene;island | 0.55738 | 0.04265 | KCNQ1; KCNQ1OT1 (margin: KCNQ1; KCNQ1OT1) | 4.79 | 4.09e-13 | 2.10e-09 |
|  | 7 | 45197251 | 45197346 | PDFSTATSENSEMBLUCSC | 96 | 4 | 37518 | tss | 0.06826 | 0.77055 | Not queried | 4.79 | 2.86e-02 | 1.00e+00 |
|  | 10 | 22624663 | 22625015 | PDFSTATSENSEMBLUCSC | 353 | 4 | 5583 | island | 0.93178 | 0.25957 | Not queried | 4.79 | 2.86e-02 | 1.00e+00 |
|  | 5 | 140810051 | 140810137 | PDFSTATSENSEMBLUCSC | 87 | 5 | 32324 | tss | 0.16812 | 0.88544 | Not queried | 4.78 | 7.94e-03 | 1.00e+00 |
|  | 5 | 137225191 | 137225509 | PDFSTATSENSEMBLUCSC | 319 | 5 | 31706 | gene;island | 0.01617 | 0.45407 | Not queried | 4.78 | 4.21e-01 | 1.00e+00 |
|  | 2 | 27805893 | 27806117 | PDFSTATSENSEMBLUCSC | 225 | 4 | 22594 | gene | 0.95876 | 0.39622 | Not queried | 4.78 | 2.86e-02 | 1.00e+00 |
|  | 5 | 162931471 | 162931829 | PDFSTATSENSEMBLUCSC | 359 | 4 | 31823 | gene;tss;island | 0.23648 | 0.01250 | Not queried | 4.78 | 2.86e-02 | 1.00e+00 |
|  | 19 | 51171640 | 51172144 | PDFSTATSENSEMBLUCSC | 505 | 5 | 20145 | gene;island | 0.07810 | 0.69250 | Not queried | 4.78 | 7.94e-03 | 1.00e+00 |
|  | 2 | 264120 | 264204 | PDFSTATSENSEMBLUCSC | 85 | 6 | 23115 | tss;island | 0.03705 | 0.45629 | Not queried | 4.77 | 2.16e-03 | 1.00e+00 |
|  | 5 | 145713607 | 145713882 | PDFSTATSENSEMBLUCSC | 276 | 6 | 33217 | island | 0.03759 | 0.54499 | Not queried | 4.77 | 2.16e-03 | 1.00e+00 |
|  | 2 | 162272107 | 162272687 | PDFSTATSENSEMBLUCSC | 581 | 7 | 24783 | island | 0.06224 | 0.71162 | Not queried | 4.77 | 5.83e-04 | 1.00e+00 |
|  | 6 | 111408745 | 111408840 | PDFSTATSENSEMBLUCSC | 96 | 6 | 36356 | island | 0.55064 | 0.03444 | Not queried | 4.77 | 2.16e-03 | 1.00e+00 |
|  | 11 | 119293601 | 119293869 | PDFSTATSENSEMBLUCSC | 269 | 5 | 6921 | gene;island | 0.72680 | 0.04512 | Not queried | 4.77 | 1.59e-02 | 1.00e+00 |
|  | 15 | 40268421 | 40268777 | PDFSTATSENSEMBLUCSC | 357 | 4 | 13257 | gene;island | 0.02310 | 0.37012 | Not queried | 4.77 | 1.14e-01 | 1.00e+00 |
|  | 7 | 98245716 | 98246006 | PDFSTATSENSEMBLUCSC | 291 | 4 | 37682 | tss;island | 0.05664 | 0.53646 | Not queried | 4.76 | 2.86e-02 | 1.00e+00 |
|  | 6 | 24494462 | 24494793 | PDFSTATSENSEMBLUCSC | 332 | 5 | 34604 | tss;island | 0.74351 | 0.09131 | Not queried | 4.76 | 7.94e-03 | 1.00e+00 |
|  | 3 | 124449238 | 124449432 | PDFSTATSENSEMBLUCSC | 195 | 4 | 27882 | gene | 0.55171 | 0.04977 | Not queried | 4.76 | 2.86e-02 | 1.00e+00 |
|  | 4 | 174459743 | 174460124 | PDFSTATSENSEMBLUCSC | 382 | 4 | 30135 | gene;island | 0.60329 | 0.05360 | Not queried | 4.76 | 2.86e-02 | 1.00e+00 |
|  | 17 | 46622454 | 46622899 | PDFSTATSENSEMBLUCSC | 446 | 8 | 17424 | tss;island | 0.75709 | 0.08148 | Not queried | 4.75 | 2.95e-03 | 1.00e+00 |
|  | 14 | 61789466 | 61789733 | PDFSTATSENSEMBLUCSC | 268 | 5 | 12079 | gene;island | 0.02199 | 0.28817 | Not queried | 4.75 | 7.94e-03 | 1.00e+00 |
|  | 7 | 27195918 | 27196555 | PDFSTATSENSEMBLUCSC | 638 | 11 | 38289 | island | 0.02090 | 0.34711 | HOXA-AS3; RP1-170O19.21; HOXA7 (margin: HOXA-AS3; RP1-170O19.21; HOXA-AS4; HOXA3; HOXA6; HOXA7; HOXA9; RP1-170O19.20) | 4.75 | 2.84e-06 | 1.46e-02 |
|  | X | 153084485 | 153085101 | PDFSTATSENSEMBLUCSC | 617 | 5 | 41150 | gene;island | 0.05975 | 0.56275 | Not queried | 4.74 | 5.56e-02 | 1.00e+00 |
|  | Y | 2655866 | 2655977 | PDFSTATSENSEMBLUCSC | 112 | 4 | 42163 | tss | 0.02194 | 0.15051 | Not queried | 4.74 | 6.86e-01 | 1.00e+00 |
|  | 1 | 108023249 | 108023482 | PDFSTATSENSEMBLUCSC | 234 | 5 | 659 | gene;island | 0.28696 | 0.91002 | Not queried | 4.74 | 7.94e-03 | 1.00e+00 |
|  | 5 | 140767197 | 140767412 | PDFSTATSENSEMBLUCSC | 216 | 7 | 31770 | gene;tss;island | 0.02196 | 0.44439 | Not queried | 4.74 | 1.17e-03 | 1.00e+00 |
|  | 6 | 5851644 | 5851994 | PDFSTATSENSEMBLUCSC | 351 | 5 | 35337 | island | 0.79716 | 0.09337 | Not queried | 4.74 | 7.94e-03 | 1.00e+00 |
|  | 22 | 44258572 | 44258712 | PDFSTATSENSEMBLUCSC | 141 | 5 | 27163 | tss;island | 0.03767 | 0.70854 | Not queried | 4.74 | 7.94e-03 | 1.00e+00 |
|  | 13 | 50070436 | 50070766 | PDFSTATSENSEMBLUCSC | 331 | 8 | 11138 | gene;island | 0.57020 | 0.06222 | Not queried | 4.74 | 1.86e-03 | 1.00e+00 |
|  | 17 | 22016686 | 22017365 | PDFSTATSENSEMBLUCSC | 680 | 6 | 18096 | island | 0.04587 | 0.48073 | Not queried | 4.74 | 2.16e-03 | 1.00e+00 |
|  | 4 | 85417312 | 85417755 | PDFSTATSENSEMBLUCSC | 444 | 4 | 30001 | gene;island | 0.64838 | 0.06623 | Not queried | 4.73 | 2.86e-02 | 1.00e+00 |
|  | 5 | 140723331 | 140723899 | PDFSTATSENSEMBLUCSC | 569 | 9 | 31761 | gene;island | 0.25800 | 0.85295 | Not queried | 4.73 | 8.23e-05 | 4.23e-01 |
|  | X | 74376241 | 74376313 | PDFSTATSENSEMBLUCSC | 73 | 4 | 41394 | tss | 0.04589 | 0.59846 | Not queried | 4.73 | 2.86e-02 | 1.00e+00 |
|  | 6 | 170578592 | 170578855 | PDFSTATSENSEMBLUCSC | 264 | 4 | 36622 | island | 0.16276 | 0.78834 | Not queried | 4.73 | 2.86e-02 | 1.00e+00 |
|  | X | 73164102 | 73164390 | PDFSTATSENSEMBLUCSC | 289 | 6 | 41873 | island | 0.02724 | 0.32240 | Not queried | 4.72 | 2.16e-03 | 1.00e+00 |
|  | 2 | 63281069 | 63281383 | PDFSTATSENSEMBLUCSC | 315 | 4 | 22667 | gene;island | 0.84976 | 0.16793 | Not queried | 4.72 | 2.86e-02 | 1.00e+00 |
|  | 7 | 128577593 | 128578176 | PDFSTATSENSEMBLUCSC | 584 | 12 | 37822 | tss | 0.06221 | 0.63632 | Not queried | 4.72 | 1.11e-03 | 1.00e+00 |
|  | 6 | 138866865 | 138867125 | PDFSTATSENSEMBLUCSC | 261 | 7 | 34424 | gene | 0.67034 | 0.09471 | Not queried | 4.72 | 4.08e-03 | 1.00e+00 |
|  | 14 | 37051693 | 37051949 | PDFSTATSENSEMBLUCSC | 257 | 7 | 12759 | island | 0.03948 | 0.57869 | Not queried | 4.72 | 5.83e-04 | 1.00e+00 |
|  | 1 | 6526049 | 6526583 | PDFSTATSENSEMBLUCSC | 535 | 12 | 173 | gene;island | 0.09128 | 0.73248 | Not queried | 4.71 | 3.64e-03 | 1.00e+00 |
|  | 16 | 14397576 | 14397892 | PDFSTATSENSEMBLUCSC | 317 | 6 | 15689 | island | 0.45233 | 0.03277 | Not queried | 4.71 | 2.16e-03 | 1.00e+00 |
|  | 14 | 45722289 | 45722745 | PDFSTATSENSEMBLUCSC | 457 | 9 | 12788 | island | 0.01834 | 0.36478 | Not queried | 4.71 | 4.11e-05 | 2.11e-01 |
|  | 11 | 2721243 | 2722713 | PDFSTATSENSEMBLUCSC | 1471 | 40 | 7057 | tss | 0.55688 | 0.04538 | KCNQ1; KCNQ1OT1 (margin: KCNQ1; KCNQ1OT1) | 4.71 | 1.16e-12 | 5.98e-09 |
|  | 15 | 68117903 | 68118127 | PDFSTATSENSEMBLUCSC | 225 | 4 | 14224 | island | 0.04174 | 0.48739 | Not queried | 4.71 | 2.86e-02 | 1.00e+00 |
|  | 13 | 111268673 | 111268714 | PDFSTATSENSEMBLUCSC | 42 | 4 | 11204 | gene;island | 0.60239 | 0.06773 | Not queried | 4.70 | 2.86e-02 | 1.00e+00 |
|  | 10 | 118386951 | 118387518 | PDFSTATSENSEMBLUCSC | 568 | 5 | 4761 | gene;island | 0.13292 | 0.79943 | Not queried | 4.70 | 7.94e-03 | 1.00e+00 |
|  | 12 | 49110427 | 49110646 | PDFSTATSENSEMBLUCSC | 220 | 4 | 9088 | gene | 0.38727 | 0.01959 | Not queried | 4.70 | 2.86e-02 | 1.00e+00 |
|  | 8 | 104153592 | 104153829 | PDFSTATSENSEMBLUCSC | 238 | 8 | 39326 | gene;tss;island | 0.55128 | 0.06016 | Not queried | 4.70 | 1.55e-04 | 7.99e-01 |
|  | 6 | 26020672 | 26020714 | PDFSTATSENSEMBLUCSC | 43 | 4 | 34614 | tss;island | 0.58043 | 0.96888 | Not queried | 4.69 | 2.86e-02 | 1.00e+00 |
|  | X | 134165954 | 134166347 | PDFSTATSENSEMBLUCSC | 394 | 9 | 42047 | island | 0.03946 | 0.52308 | Not queried | 4.69 | 4.11e-05 | 2.11e-01 |
|  | X | 134165954 | 134166287 | PDFSTATSENSEMBLUCSC | 334 | 7 | 41537 | tss | 0.03946 | 0.52308 | Not queried | 4.69 | 5.83e-04 | 1.00e+00 |
|  | X | 49022793 | 49023115 | PDFSTATSENSEMBLUCSC | 323 | 5 | 40993 | gene;island | 0.43453 | 0.04132 | Not queried | 4.69 | 7.94e-03 | 1.00e+00 |
|  | 10 | 93392644 | 93392839 | PDFSTATSENSEMBLUCSC | 196 | 4 | 4659 | gene | 0.14399 | 0.79183 | Not queried | 4.69 | 2.86e-02 | 1.00e+00 |
|  | 2 | 219773416 | 219774183 | PDFSTATSENSEMBLUCSC | 768 | 8 | 25057 | island | 0.02239 | 0.38885 | Not queried | 4.69 | 3.11e-04 | 1.00e+00 |
|  | 15 | 29033879 | 29033901 | PDFSTATSENSEMBLUCSC | 23 | 6 | 13943 | island | 0.35560 | 0.90957 | Not queried | 4.69 | 2.16e-03 | 1.00e+00 |
|  | 10 | 47151300 | 47151589 | PDFSTATSENSEMBLUCSC | 290 | 5 | 5702 | island | 0.02682 | 0.39541 | Not queried | 4.69 | 7.94e-03 | 1.00e+00 |
|  | X | 153656771 | 153656930 | PDFSTATSENSEMBLUCSC | 160 | 4 | 41599 | tss | 0.04837 | 0.57339 | Not queried | 4.68 | 2.86e-02 | 1.00e+00 |
|  | 1 | 156163644 | 156164205 | PDFSTATSENSEMBLUCSC | 562 | 10 | 3835 | island | 0.02918 | 0.37322 | Not queried | 4.68 | 1.08e-05 | 5.56e-02 |
|  | 12 | 54070891 | 54071194 | PDFSTATSENSEMBLUCSC | 304 | 7 | 9721 | tss;island | 0.01587 | 0.29912 | Not queried | 4.68 | 5.83e-04 | 1.00e+00 |
|  | 2 | 203103432 | 203103763 | PDFSTATSENSEMBLUCSC | 332 | 5 | 23786 | tss;island | 0.00950 | 0.17276 | Not queried | 4.68 | 1.51e-01 | 1.00e+00 |
|  | 5 | 63257499 | 63257941 | PDFSTATSENSEMBLUCSC | 443 | 10 | 32764 | island | 0.07590 | 0.61682 | Not queried | 4.67 | 1.08e-05 | 5.56e-02 |
|  | 2 | 131185333 | 131185700 | PDFSTATSENSEMBLUCSC | 368 | 6 | 24682 | island | 0.08931 | 0.65969 | Not queried | 4.67 | 2.16e-03 | 1.00e+00 |
|  | 17 | 46608099 | 46608570 | PDFSTATSENSEMBLUCSC | 472 | 11 | 18440 | island | 0.83132 | 0.20544 | Not queried | 4.67 | 1.13e-05 | 5.83e-02 |
|  | 3 | 50275394 | 50275694 | PDFSTATSENSEMBLUCSC | 301 | 5 | 27754 | gene;island | 0.32729 | 0.01950 | Not queried | 4.67 | 7.94e-03 | 1.00e+00 |
|  | 3 | 192444985 | 192445380 | PDFSTATSENSEMBLUCSC | 396 | 5 | 28044 | gene | 0.02891 | 0.46506 | Not queried | 4.67 | 3.17e-02 | 1.00e+00 |
|  | 20 | 44451667 | 44451973 | PDFSTATSENSEMBLUCSC | 307 | 4 | 26163 | island | 0.07554 | 0.70694 | Not queried | 4.66 | 2.86e-02 | 1.00e+00 |
|  | 1 | 55445834 | 55446387 | PDFSTATSENSEMBLUCSC | 554 | 7 | 1647 | tss | 0.07771 | 0.76319 | Not queried | 4.66 | 2.62e-02 | 1.00e+00 |
|  | 1 | 111506577 | 111507160 | PDFSTATSENSEMBLUCSC | 584 | 11 | 1817 | tss | 0.01602 | 0.28924 | LRIF1 (margin: RP11-96K19.2; LRIF1) | 4.66 | 2.84e-06 | 1.46e-02 |
|  | 12 | 133177383 | 133177827 | PDFSTATSENSEMBLUCSC | 445 | 4 | 11036 | island | 0.10846 | 0.71139 | Not queried | 4.66 | 2.86e-02 | 1.00e+00 |
|  | 19 | 56915403 | 56915658 | PDFSTATSENSEMBLUCSC | 256 | 6 | 21061 | tss | 0.53482 | 0.04028 | Not queried | 4.65 | 2.16e-03 | 1.00e+00 |
|  | 4 | 174427388 | 174428191 | PDFSTATSENSEMBLUCSC | 804 | 9 | 31370 | island | 0.14619 | 0.74207 | Not queried | 4.65 | 4.94e-04 | 1.00e+00 |
|  | 4 | 89619023 | 89619573 | PDFSTATSENSEMBLUCSC | 551 | 12 | 30448 | tss | 0.24005 | 0.92819 | Not queried | 4.65 | 1.03e-04 | 5.28e-01 |
|  | 7 | 157690609 | 157690769 | PDFSTATSENSEMBLUCSC | 161 | 4 | 37286 | gene;island | 0.32626 | 0.83345 | Not queried | 4.65 | 2.86e-02 | 1.00e+00 |
|  | 7 | 3033408 | 3033939 | PDFSTATSENSEMBLUCSC | 532 | 5 | 36760 | gene | 0.27220 | 0.90148 | Not queried | 4.65 | 7.94e-03 | 1.00e+00 |
|  | 5 | 134181484 | 134181607 | PDFSTATSENSEMBLUCSC | 124 | 6 | 32240 | tss | 0.01147 | 0.31932 | Not queried | 4.65 | 2.16e-03 | 1.00e+00 |
|  | 17 | 56738915 | 56739129 | PDFSTATSENSEMBLUCSC | 215 | 5 | 16676 | gene;island | 0.22515 | 0.94241 | Not queried | 4.64 | 7.94e-03 | 1.00e+00 |
|  | 3 | 8810980 | 8811279 | PDFSTATSENSEMBLUCSC | 300 | 4 | 27605 | gene | 0.02427 | 0.36286 | Not queried | 4.64 | 3.43e-01 | 1.00e+00 |
|  | 10 | 22726610 | 22726885 | PDFSTATSENSEMBLUCSC | 276 | 6 | 5587 | island | 0.54736 | 0.04777 | Not queried | 4.64 | 2.16e-03 | 1.00e+00 |
|  | 3 | 54157557 | 54157900 | PDFSTATSENSEMBLUCSC | 344 | 5 | 27793 | gene;island | 0.02001 | 0.31685 | Not queried | 4.63 | 7.94e-03 | 1.00e+00 |
|  | 5 | 151304770 | 151305143 | PDFSTATSENSEMBLUCSC | 374 | 6 | 32386 | tss;island | 0.04860 | 0.53672 | Not queried | 4.63 | 8.66e-03 | 1.00e+00 |
|  | 12 | 115124584 | 115125098 | PDFSTATSENSEMBLUCSC | 515 | 5 | 10820 | island | 0.73025 | 0.11167 | Not queried | 4.63 | 7.94e-03 | 1.00e+00 |
|  | 12 | 107348689 | 107349191 | PDFSTATSENSEMBLUCSC | 503 | 7 | 9946 | tss;island | 0.68126 | 0.02613 | Not queried | 4.63 | 2.59e-01 | 1.00e+00 |
|  | X | 153990478 | 153991009 | PDFSTATSENSEMBLUCSC | 532 | 9 | 41608 | tss | 0.02661 | 0.54119 | Not queried | 4.63 | 2.76e-03 | 1.00e+00 |
|  | 5 | 172661803 | 172662130 | PDFSTATSENSEMBLUCSC | 328 | 4 | 31845 | gene | 0.18195 | 0.85955 | Not queried | 4.63 | 2.86e-02 | 1.00e+00 |
|  | 6 | 34206400 | 34206683 | PDFSTATSENSEMBLUCSC | 284 | 4 | 34237 | gene;island | 0.51585 | 0.02690 | Not queried | 4.63 | 1.14e-01 | 1.00e+00 |
|  | 5 | 44388799 | 44389062 | PDFSTATSENSEMBLUCSC | 264 | 6 | 31993 | tss | 0.04644 | 0.52527 | Not queried | 4.63 | 2.16e-03 | 1.00e+00 |
|  | 1 | 233086060 | 233086622 | PDFSTATSENSEMBLUCSC | 563 | 12 | 4318 | island | 0.38720 | 0.02586 | NTPCR (margin: NTPCR) | 4.62 | 7.40e-07 | 3.80e-03 |
|  | 22 | 33195343 | 33195688 | PDFSTATSENSEMBLUCSC | 346 | 6 | 26808 | gene;tss;island | 0.53274 | 0.95858 | Not queried | 4.62 | 2.16e-03 | 1.00e+00 |
|  | 7 | 92672812 | 92673176 | PDFSTATSENSEMBLUCSC | 365 | 5 | 38601 | island | 0.51182 | 0.01859 | Not queried | 4.62 | 7.94e-03 | 1.00e+00 |
|  | 17 | 8230130 | 8230940 | PDFSTATSENSEMBLUCSC | 811 | 7 | 17968 | island | 0.22522 | 0.87869 | Not queried | 4.62 | 6.99e-03 | 1.00e+00 |
|  | 10 | 22634432 | 22634733 | PDFSTATSENSEMBLUCSC | 302 | 5 | 4517 | gene;island | 0.05389 | 0.58307 | Not queried | 4.62 | 7.94e-03 | 1.00e+00 |
|  | 6 | 99291343 | 99291677 | PDFSTATSENSEMBLUCSC | 335 | 4 | 36284 | island | 0.54792 | 0.03721 | Not queried | 4.61 | 2.86e-02 | 1.00e+00 |
|  | 11 | 35547074 | 35547311 | PDFSTATSENSEMBLUCSC | 238 | 4 | 8188 | island | 0.61139 | 0.07318 | Not queried | 4.61 | 2.86e-02 | 1.00e+00 |
|  | X | 68835489 | 68835895 | PDFSTATSENSEMBLUCSC | 407 | 7 | 41362 | tss | 0.09679 | 0.00772 | Not queried | 4.61 | 1.11e-02 | 1.00e+00 |
|  | 5 | 140098174 | 140098496 | PDFSTATSENSEMBLUCSC | 323 | 6 | 32291 | tss | 0.02740 | 0.29454 | Not queried | 4.61 | 2.16e-03 | 1.00e+00 |
|  | 2 | 162273011 | 162273648 | PDFSTATSENSEMBLUCSC | 638 | 7 | 22872 | gene;island | 0.13539 | 0.80642 | Not queried | 4.61 | 5.83e-04 | 1.00e+00 |
|  | 12 | 63195695 | 63195954 | PDFSTATSENSEMBLUCSC | 260 | 4 | 9175 | gene;island | 0.36942 | 0.92528 | Not queried | 4.60 | 2.86e-02 | 1.00e+00 |
|  | 13 | 20392406 | 20392706 | PDFSTATSENSEMBLUCSC | 301 | 5 | 11534 | island | 0.06805 | 0.66666 | Not queried | 4.60 | 7.94e-03 | 1.00e+00 |
|  | 12 | 6484521 | 6484729 | PDFSTATSENSEMBLUCSC | 209 | 4 | 9002 | gene;tss | 0.05482 | 0.55220 | Not queried | 4.60 | 2.86e-02 | 1.00e+00 |
|  | 2 | 33701120 | 33701319 | PDFSTATSENSEMBLUCSC | 200 | 4 | 22605 | gene;tss | 0.02913 | 0.41149 | Not queried | 4.60 | 2.86e-02 | 1.00e+00 |
|  | 16 | 88717464 | 88717989 | PDFSTATSENSEMBLUCSC | 526 | 11 | 15380 | tss | 0.67959 | 0.10529 | CYBA; MVD (margin: IL17C; SNAI3-AS1; CYBA; MVD) | 4.60 | 5.67e-06 | 2.91e-02 |
|  | 10 | 118429481 | 118429952 | PDFSTATSENSEMBLUCSC | 472 | 7 | 5369 | tss | 0.05003 | 0.57135 | Not queried | 4.59 | 5.83e-04 | 1.00e+00 |
|  | 17 | 36105117 | 36105517 | PDFSTATSENSEMBLUCSC | 401 | 6 | 17258 | tss | 0.66036 | 0.08689 | Not queried | 4.59 | 2.16e-03 | 1.00e+00 |
|  | 8 | 145027948 | 145028505 | PDFSTATSENSEMBLUCSC | 558 | 10 | 39441 | gene;island | 0.56490 | 0.03744 | Not queried | 4.59 | 3.89e-03 | 1.00e+00 |
|  | 19 | 58570419 | 58570491 | PDFSTATSENSEMBLUCSC | 73 | 6 | 21095 | tss;island | 0.02544 | 0.38647 | Not queried | 4.59 | 2.16e-03 | 1.00e+00 |
|  | 3 | 35720994 | 35721284 | PDFSTATSENSEMBLUCSC | 291 | 5 | 27670 | gene;tss | 0.04314 | 0.50425 | Not queried | 4.59 | 7.94e-03 | 1.00e+00 |
|  | 11 | 66360492 | 66360803 | PDFSTATSENSEMBLUCSC | 312 | 10 | 7454 | tss;island | 0.02479 | 0.39124 | Not queried | 4.59 | 2.17e-05 | 1.11e-01 |
|  | 11 | 66360740 | 66360803 | PDFSTATSENSEMBLUCSC | 64 | 4 | 6716 | gene | 0.01831 | 0.27685 | Not queried | 4.59 | 2.86e-02 | 1.00e+00 |
|  | 21 | 26934197 | 26934682 | PDFSTATSENSEMBLUCSC | 486 | 6 | 26574 | island | 0.04629 | 0.60903 | Not queried | 4.59 | 2.16e-03 | 1.00e+00 |
|  | 21 | 42792127 | 42792703 | PDFSTATSENSEMBLUCSC | 577 | 6 | 26648 | island | 0.03277 | 0.35871 | Not queried | 4.59 | 8.66e-03 | 1.00e+00 |
|  | 13 | 51417686 | 51418221 | PDFSTATSENSEMBLUCSC | 536 | 10 | 11716 | island | 0.06150 | 0.54352 | Not queried | 4.59 | 1.50e-03 | 1.00e+00 |
|  | 8 | 56433594 | 56433717 | PDFSTATSENSEMBLUCSC | 124 | 4 | 39246 | gene | 0.02747 | 0.27553 | Not queried | 4.59 | 2.86e-02 | 1.00e+00 |
|  | 5 | 3590820 | 3591171 | PDFSTATSENSEMBLUCSC | 352 | 4 | 32604 | island | 0.05826 | 0.63142 | Not queried | 4.58 | 2.86e-02 | 1.00e+00 |
|  | 22 | 17850431 | 17850690 | PDFSTATSENSEMBLUCSC | 260 | 4 | 27231 | island | 0.39155 | 0.04515 | Not queried | 4.58 | 2.86e-02 | 1.00e+00 |
|  | 7 | 143105826 | 143105970 | PDFSTATSENSEMBLUCSC | 145 | 4 | 37192 | gene | 0.04484 | 0.48612 | Not queried | 4.58 | 2.86e-02 | 1.00e+00 |
|  | 2 | 241395181 | 241395351 | PDFSTATSENSEMBLUCSC | 171 | 4 | 23084 | gene;tss;island | 0.08182 | 0.71385 | Not queried | 4.58 | 2.86e-02 | 1.00e+00 |
|  | 1 | 156182812 | 156183160 | PDFSTATSENSEMBLUCSC | 349 | 4 | 815 | gene | 0.44794 | 0.03324 | Not queried | 4.58 | 2.86e-02 | 1.00e+00 |
|  | 19 | 9649241 | 9649542 | PDFSTATSENSEMBLUCSC | 302 | 9 | 21464 | island | 0.37765 | 0.02568 | Not queried | 4.58 | 4.11e-05 | 2.11e-01 |
|  | 19 | 9649310 | 9649542 | PDFSTATSENSEMBLUCSC | 233 | 7 | 20390 | tss | 0.40138 | 0.02568 | Not queried | 4.58 | 5.83e-04 | 1.00e+00 |
|  | 6 | 99292170 | 99292438 | PDFSTATSENSEMBLUCSC | 269 | 4 | 36285 | island | 0.37899 | 0.04969 | Not queried | 4.57 | 2.86e-02 | 1.00e+00 |
|  | 5 | 126409007 | 126409573 | PDFSTATSENSEMBLUCSC | 567 | 12 | 33003 | island | 0.45541 | 0.93733 | Not queried | 4.57 | 1.44e-04 | 7.41e-01 |
|  | 5 | 1633830 | 1634218 | PDFSTATSENSEMBLUCSC | 389 | 5 | 32576 | island | 0.51457 | 0.06570 | Not queried | 4.57 | 7.94e-03 | 1.00e+00 |
|  | 1 | 74663927 | 74664160 | PDFSTATSENSEMBLUCSC | 234 | 4 | 583 | gene | 0.03001 | 0.41188 | Not queried | 4.56 | 2.86e-02 | 1.00e+00 |
|  | 3 | 44754201 | 44754587 | PDFSTATSENSEMBLUCSC | 387 | 4 | 27700 | gene | 0.51965 | 0.04417 | Not queried | 4.55 | 2.86e-02 | 1.00e+00 |
|  | 20 | 33999839 | 33999889 | PDFSTATSENSEMBLUCSC | 51 | 6 | 25666 | tss | 0.32733 | 0.02852 | Not queried | 4.55 | 2.16e-03 | 1.00e+00 |
|  | 6 | 27648317 | 27648605 | PDFSTATSENSEMBLUCSC | 289 | 5 | 35498 | island | 0.03380 | 0.48728 | Not queried | 4.55 | 7.94e-03 | 1.00e+00 |
|  | 6 | 32407689 | 32407838 | PDFSTATSENSEMBLUCSC | 150 | 4 | 34050 | gene | 0.55115 | 0.06893 | Not queried | 4.55 | 2.00e-01 | 1.00e+00 |
|  | X | 53254643 | 53254818 | PDFSTATSENSEMBLUCSC | 176 | 7 | 41328 | tss;island | 0.03297 | 0.26112 | Not queried | 4.55 | 5.83e-04 | 1.00e+00 |
|  | X | 21676344 | 21677134 | PDFSTATSENSEMBLUCSC | 791 | 10 | 41681 | island | 0.31919 | 0.91372 | Not queried | 4.55 | 1.08e-05 | 5.56e-02 |
|  | 3 | 49066699 | 49067151 | PDFSTATSENSEMBLUCSC | 453 | 11 | 29063 | island | 0.02423 | 0.33064 | RP13-131K19.6; IMPDH2; QRICH1 (margin: NDUFAF3; RP13-131K19.6; DALRD3; MIR425; MIR191; IMPDH2; QRICH1) | 4.54 | 2.84e-06 | 1.46e-02 |
|  | 1 | 154474344 | 154474530 | PDFSTATSENSEMBLUCSC | 187 | 4 | 776 | gene;tss;island | 0.03175 | 0.38735 | Not queried | 4.54 | 2.86e-02 | 1.00e+00 |
|  | 1 | 172113756 | 172114068 | PDFSTATSENSEMBLUCSC | 313 | 4 | 883 | gene | 0.31775 | 0.91485 | Not queried | 4.54 | 2.86e-02 | 1.00e+00 |
|  | 12 | 114838567 | 114839007 | PDFSTATSENSEMBLUCSC | 441 | 5 | 9298 | gene;island | 0.91183 | 0.30763 | Not queried | 4.54 | 7.94e-03 | 1.00e+00 |
|  | X | 21676481 | 21677134 | PDFSTATSENSEMBLUCSC | 654 | 9 | 41220 | tss | 0.24764 | 0.90738 | Not queried | 4.54 | 4.11e-05 | 2.11e-01 |
|  | 20 | 44657637 | 44657948 | PDFSTATSENSEMBLUCSC | 312 | 7 | 25400 | gene;island | 0.22158 | 0.82960 | Not queried | 4.54 | 5.83e-04 | 1.00e+00 |
|  | Y | 22737896 | 22738029 | PDFSTATSENSEMBLUCSC | 134 | 4 | 42162 | gene;island | 0.05432 | 0.43069 | Not queried | 4.54 | 2.86e-02 | 1.00e+00 |
|  | 6 | 36355488 | 36355825 | PDFSTATSENSEMBLUCSC | 338 | 6 | 34872 | tss | 0.08580 | 0.75344 | Not queried | 4.54 | 2.16e-03 | 1.00e+00 |
|  | 5 | 140220686 | 140220822 | PDFSTATSENSEMBLUCSC | 137 | 4 | 32295 | tss | 0.34159 | 0.87197 | Not queried | 4.54 | 2.86e-02 | 1.00e+00 |
|  | 12 | 6665108 | 6665447 | PDFSTATSENSEMBLUCSC | 340 | 7 | 10173 | island | 0.66848 | 0.07365 | Not queried | 4.53 | 5.83e-04 | 1.00e+00 |
|  | 7 | 28319343 | 28319676 | PDFSTATSENSEMBLUCSC | 334 | 6 | 37439 | tss | 0.38014 | 0.93616 | Not queried | 4.53 | 1.32e-01 | 1.00e+00 |
|  | 1 | 233086060 | 233086346 | PDFSTATSENSEMBLUCSC | 287 | 8 | 2321 | tss | 0.39822 | 0.02603 | Not queried | 4.53 | 1.55e-04 | 7.99e-01 |
|  | 14 | 77737495 | 77737800 | PDFSTATSENSEMBLUCSC | 306 | 6 | 13003 | island | 0.23837 | 0.88984 | Not queried | 4.52 | 2.16e-03 | 1.00e+00 |
|  | 5 | 2754753 | 2755158 | PDFSTATSENSEMBLUCSC | 406 | 4 | 31495 | gene;island | 0.05119 | 0.01891 | Not queried | 4.52 | 2.86e-02 | 1.00e+00 |
|  | 12 | 6665288 | 6665447 | PDFSTATSENSEMBLUCSC | 160 | 6 | 9491 | tss | 0.66558 | 0.07841 | Not queried | 4.52 | 2.16e-03 | 1.00e+00 |
|  | 2 | 162272107 | 162272604 | PDFSTATSENSEMBLUCSC | 498 | 6 | 23649 | tss | 0.07254 | 0.64519 | Not queried | 4.52 | 2.16e-03 | 1.00e+00 |
|  | 6 | 122931154 | 122931308 | PDFSTATSENSEMBLUCSC | 155 | 8 | 35142 | tss | 0.05218 | 0.57989 | Not queried | 4.52 | 1.55e-04 | 7.99e-01 |
|  | 10 | 118429452 | 118429952 | PDFSTATSENSEMBLUCSC | 501 | 8 | 6147 | island | 0.05070 | 0.60738 | Not queried | 4.52 | 6.99e-03 | 1.00e+00 |
|  | 17 | 46688182 | 46688845 | PDFSTATSENSEMBLUCSC | 664 | 13 | 18455 | island | 0.01769 | 0.24952 | HOXB7; HOXB8 (margin: HOXB-AS3; HOXB3; HOXB6; HOXB7; HOXB8; HOXB9) | 4.51 | 1.92e-07 | 9.88e-04 |
|  | 17 | 46688405 | 46688845 | PDFSTATSENSEMBLUCSC | 441 | 9 | 17433 | tss | 0.02149 | 0.33391 | Not queried | 4.51 | 4.11e-05 | 2.11e-01 |
|  | 5 | 10307544 | 10307831 | PDFSTATSENSEMBLUCSC | 288 | 4 | 31504 | gene;island | 0.02424 | 0.33822 | Not queried | 4.51 | 2.86e-02 | 1.00e+00 |
|  | 1 | 178994243 | 178994355 | PDFSTATSENSEMBLUCSC | 113 | 4 | 2110 | tss;island | 0.54357 | 0.04792 | Not queried | 4.50 | 2.86e-02 | 1.00e+00 |
|  | 19 | 7553986 | 7554201 | PDFSTATSENSEMBLUCSC | 216 | 6 | 20362 | tss | 0.03361 | 0.46488 | Not queried | 4.50 | 2.16e-03 | 1.00e+00 |
|  | 18 | 48085671 | 48085844 | PDFSTATSENSEMBLUCSC | 174 | 4 | 19242 | tss;island | 0.53860 | 0.04163 | Not queried | 4.50 | 2.86e-02 | 1.00e+00 |
|  | 16 | 90114063 | 90114307 | PDFSTATSENSEMBLUCSC | 245 | 6 | 15407 | tss | 0.03378 | 0.47087 | Not queried | 4.50 | 4.33e-03 | 1.00e+00 |
|  | 3 | 49314595 | 49314920 | PDFSTATSENSEMBLUCSC | 326 | 8 | 27737 | gene | 0.51334 | 0.96028 | Not queried | 4.50 | 1.55e-04 | 7.99e-01 |
|  | 13 | 99630462 | 99630583 | PDFSTATSENSEMBLUCSC | 122 | 4 | 11175 | gene;tss | 0.03092 | 0.48906 | Not queried | 4.49 | 2.86e-02 | 1.00e+00 |
|  | 11 | 70917159 | 70917533 | PDFSTATSENSEMBLUCSC | 375 | 4 | 6782 | gene | 0.31167 | 0.94607 | Not queried | 4.49 | 5.71e-02 | 1.00e+00 |
|  | 15 | 93633145 | 93633408 | PDFSTATSENSEMBLUCSC | 264 | 5 | 13888 | tss;island | 0.74837 | 0.04680 | Not queried | 4.49 | 7.94e-03 | 1.00e+00 |
|  | 18 | 32398024 | 32398287 | PDFSTATSENSEMBLUCSC | 264 | 4 | 19053 | gene;tss | 0.27133 | 0.92034 | Not queried | 4.49 | 5.71e-02 | 1.00e+00 |
|  | 5 | 140235435 | 140236081 | PDFSTATSENSEMBLUCSC | 647 | 9 | 31743 | gene;island | 0.08028 | 0.62538 | Not queried | 4.49 | 4.11e-05 | 2.11e-01 |
|  | 17 | 46805785 | 46806056 | PDFSTATSENSEMBLUCSC | 272 | 4 | 16631 | gene | 0.03378 | 0.32355 | Not queried | 4.49 | 2.86e-02 | 1.00e+00 |
|  | 10 | 63809073 | 63809170 | PDFSTATSENSEMBLUCSC | 98 | 6 | 4586 | gene | 0.02540 | 0.37797 | Not queried | 4.48 | 2.16e-03 | 1.00e+00 |
|  | 6 | 4282075 | 4282346 | PDFSTATSENSEMBLUCSC | 272 | 4 | 35330 | island | 0.19794 | 0.79521 | Not queried | 4.48 | 2.86e-02 | 1.00e+00 |
|  | X | 47518016 | 47518238 | PDFSTATSENSEMBLUCSC | 223 | 6 | 41284 | tss | 0.02701 | 0.45464 | Not queried | 4.48 | 2.16e-03 | 1.00e+00 |
|  | 17 | 6899085 | 6899888 | PDFSTATSENSEMBLUCSC | 804 | 14 | 17880 | island | 0.05150 | 0.56600 | Not queried | 4.47 | 3.52e-03 | 1.00e+00 |
|  | 2 | 136743411 | 136743698 | PDFSTATSENSEMBLUCSC | 288 | 5 | 23610 | tss;island | 0.02016 | 0.47865 | Not queried | 4.47 | 7.94e-03 | 1.00e+00 |
|  | 6 | 33401480 | 33401542 | PDFSTATSENSEMBLUCSC | 63 | 5 | 34227 | gene | 0.80676 | 0.16131 | Not queried | 4.47 | 7.94e-03 | 1.00e+00 |
|  | 10 | 14050305 | 14050521 | PDFSTATSENSEMBLUCSC | 217 | 4 | 4494 | gene | 0.25064 | 0.86800 | Not queried | 4.47 | 2.86e-02 | 1.00e+00 |
|  | 6 | 111580120 | 111580939 | PDFSTATSENSEMBLUCSC | 820 | 15 | 36357 | island | 0.42121 | 0.02368 | KIAA1919; RP11-428F8.2 (margin: KIAA1919; RP11-428F8.2) | 4.47 | 6.55e-06 | 3.37e-02 |
|  | 3 | 49395138 | 49395303 | PDFSTATSENSEMBLUCSC | 166 | 4 | 27738 | gene;island | 0.89328 | 0.30875 | Not queried | 4.47 | 2.86e-02 | 1.00e+00 |
|  | X | 100446220 | 100446736 | PDFSTATSENSEMBLUCSC | 517 | 5 | 41908 | island | 0.05323 | 0.55734 | Not queried | 4.47 | 7.94e-03 | 1.00e+00 |
|  | 10 | 131843517 | 131843798 | PDFSTATSENSEMBLUCSC | 282 | 4 | 6251 | island | 0.23413 | 0.80118 | Not queried | 4.46 | 2.86e-02 | 1.00e+00 |
|  | 14 | 103294363 | 103294740 | PDFSTATSENSEMBLUCSC | 378 | 5 | 12205 | gene | 0.11337 | 0.85966 | Not queried | 4.46 | 7.94e-03 | 1.00e+00 |
|  | 15 | 75918608 | 75918757 | PDFSTATSENSEMBLUCSC | 150 | 4 | 13787 | tss;island | 0.42084 | 0.03430 | Not queried | 4.46 | 2.86e-02 | 1.00e+00 |
|  | 19 | 49655176 | 49655517 | PDFSTATSENSEMBLUCSC | 342 | 4 | 20108 | gene;island | 0.02909 | 0.38853 | Not queried | 4.46 | 2.86e-02 | 1.00e+00 |
|  | 2 | 228736089 | 228736324 | PDFSTATSENSEMBLUCSC | 236 | 6 | 23894 | tss | 0.04927 | 0.46116 | Not queried | 4.46 | 2.16e-03 | 1.00e+00 |
|  | 2 | 229046325 | 229046785 | PDFSTATSENSEMBLUCSC | 461 | 5 | 25132 | island | 0.67879 | 0.07967 | Not queried | 4.46 | 9.52e-02 | 1.00e+00 |
|  | 11 | 57364841 | 57365088 | PDFSTATSENSEMBLUCSC | 248 | 6 | 7283 | tss | 0.76968 | 0.08072 | Not queried | 4.46 | 2.16e-03 | 1.00e+00 |
|  | 16 | 89573312 | 89573631 | PDFSTATSENSEMBLUCSC | 320 | 5 | 15394 | tss;island | 0.09915 | 0.76786 | Not queried | 4.45 | 7.94e-03 | 1.00e+00 |
|  | 6 | 31237013 | 31237199 | PDFSTATSENSEMBLUCSC | 187 | 4 | 33801 | gene;island | 0.10794 | 0.68423 | Not queried | 4.45 | 5.71e-02 | 1.00e+00 |
|  | 5 | 140772182 | 140772859 | PDFSTATSENSEMBLUCSC | 678 | 7 | 31771 | gene;island | 0.43160 | 0.92864 | Not queried | 4.45 | 5.83e-04 | 1.00e+00 |
|  | 2 | 115419537 | 115419846 | PDFSTATSENSEMBLUCSC | 310 | 4 | 22801 | gene;island | 0.05122 | 0.53643 | Not queried | 4.45 | 2.86e-02 | 1.00e+00 |
|  | 10 | 88391658 | 88392088 | PDFSTATSENSEMBLUCSC | 431 | 4 | 5888 | island | 0.06956 | 0.58392 | Not queried | 4.45 | 2.00e-01 | 1.00e+00 |
|  | 7 | 5085252 | 5085485 | PDFSTATSENSEMBLUCSC | 234 | 7 | 37354 | tss | 0.37357 | 0.02759 | Not queried | 4.45 | 5.83e-04 | 1.00e+00 |
|  | 19 | 39894559 | 39894767 | PDFSTATSENSEMBLUCSC | 209 | 4 | 21959 | island | 0.51853 | 0.05908 | Not queried | 4.45 | 2.86e-02 | 1.00e+00 |
|  | 10 | 22633916 | 22634226 | PDFSTATSENSEMBLUCSC | 311 | 8 | 4968 | tss;island | 0.04690 | 0.48472 | Not queried | 4.45 | 3.11e-04 | 1.00e+00 |
|  | 7 | 140103545 | 140103783 | PDFSTATSENSEMBLUCSC | 239 | 7 | 37873 | tss | 0.31510 | 0.85610 | Not queried | 4.45 | 5.83e-04 | 1.00e+00 |
|  | 20 | 57425979 | 57428032 | PDFSTATSENSEMBLUCSC | 2054 | 65 | 25433 | gene;tss;island | 0.49326 | 0.03771 | GNAS; GNAS-AS1 (margin: GNAS; GNAS-AS1; RP1-309F20.3) | 4.44 | 6.13e-17 | 3.15e-13 |
|  | 16 | 31159558 | 31159920 | PDFSTATSENSEMBLUCSC | 363 | 5 | 14703 | gene;island | 0.03265 | 0.41291 | Not queried | 4.44 | 7.94e-03 | 1.00e+00 |
|  | 7 | 158059704 | 158060003 | PDFSTATSENSEMBLUCSC | 300 | 5 | 37299 | gene;island | 0.22085 | 0.86715 | Not queried | 4.44 | 9.52e-02 | 1.00e+00 |
|  | 7 | 88425077 | 88425148 | PDFSTATSENSEMBLUCSC | 72 | 5 | 37628 | tss | 0.19341 | 0.68169 | Not queried | 4.44 | 7.94e-03 | 1.00e+00 |
|  | 11 | 86383182 | 86383585 | PDFSTATSENSEMBLUCSC | 404 | 12 | 6835 | gene | 0.05540 | 0.56346 | ME3 (margin: ME3) | 4.44 | 7.40e-07 | 3.80e-03 |
|  | 3 | 49314539 | 49314920 | PDFSTATSENSEMBLUCSC | 382 | 9 | 28283 | tss | 0.53903 | 0.95567 | Not queried | 4.44 | 2.76e-03 | 1.00e+00 |
|  | 3 | 49314370 | 49314920 | PDFSTATSENSEMBLUCSC | 551 | 11 | 29070 | island | 0.48764 | 0.94217 | Not queried | 4.44 | 3.94e-04 | 1.00e+00 |
|  | 16 | 67876973 | 67877413 | PDFSTATSENSEMBLUCSC | 441 | 4 | 14773 | gene;island | 0.45727 | 0.04507 | Not queried | 4.43 | 1.14e-01 | 1.00e+00 |
|  | 17 | 36105064 | 36105517 | PDFSTATSENSEMBLUCSC | 454 | 7 | 18217 | island | 0.65114 | 0.08575 | Not queried | 4.43 | 5.83e-04 | 1.00e+00 |
|  | 2 | 165812079 | 165812297 | PDFSTATSENSEMBLUCSC | 219 | 4 | 23656 | tss | 0.04314 | 0.57353 | Not queried | 4.43 | 5.71e-02 | 1.00e+00 |
|  | 2 | 583115 | 583384 | PDFSTATSENSEMBLUCSC | 270 | 5 | 23969 | island | 0.38865 | 0.94310 | Not queried | 4.43 | 7.94e-03 | 1.00e+00 |
|  | 22 | 38479181 | 38479332 | PDFSTATSENSEMBLUCSC | 152 | 6 | 27088 | tss | 0.31065 | 0.84218 | Not queried | 4.43 | 2.16e-03 | 1.00e+00 |
|  | 20 | 62406677 | 62406964 | PDFSTATSENSEMBLUCSC | 288 | 4 | 25473 | gene;island | 0.10595 | 0.72733 | Not queried | 4.43 | 5.71e-02 | 1.00e+00 |
|  | 17 | 74075092 | 74075195 | PDFSTATSENSEMBLUCSC | 104 | 4 | 17637 | tss | 0.19680 | 0.73880 | Not queried | 4.43 | 2.86e-02 | 1.00e+00 |
|  | X | 53350498 | 53350693 | PDFSTATSENSEMBLUCSC | 196 | 6 | 41807 | island | 0.03304 | 0.44140 | Not queried | 4.42 | 2.16e-03 | 1.00e+00 |
|  | 1 | 41444651 | 41444871 | PDFSTATSENSEMBLUCSC | 221 | 9 | 1540 | tss;island | 0.34793 | 0.05910 | Not queried | 4.42 | 4.11e-05 | 2.11e-01 |
|  | 7 | 5085252 | 5085745 | PDFSTATSENSEMBLUCSC | 494 | 9 | 38145 | island | 0.26526 | 0.02759 | Not queried | 4.42 | 4.11e-05 | 2.11e-01 |
|  | 10 | 97850047 | 97850343 | PDFSTATSENSEMBLUCSC | 297 | 5 | 5952 | island | 0.40319 | 0.93526 | Not queried | 4.42 | 1.51e-01 | 1.00e+00 |
|  | 2 | 39005329 | 39005834 | PDFSTATSENSEMBLUCSC | 506 | 5 | 22614 | gene | 0.02556 | 0.35928 | Not queried | 4.42 | 7.94e-03 | 1.00e+00 |
|  | 5 | 135415693 | 135416613 | PDFSTATSENSEMBLUCSC | 921 | 16 | 33084 | island | 0.05114 | 0.53300 | VTRNA2-1 (margin: VTRNA2-1) | 4.42 | 3.33e-09 | 1.71e-05 |
|  | 12 | 7073179 | 7073247 | PDFSTATSENSEMBLUCSC | 69 | 4 | 9515 | tss | 0.31314 | 0.86460 | Not queried | 4.42 | 2.86e-02 | 1.00e+00 |
|  | 2 | 6121909 | 6122107 | PDFSTATSENSEMBLUCSC | 199 | 4 | 23127 | tss | 0.11130 | 0.74197 | Not queried | 4.41 | 2.86e-02 | 1.00e+00 |
|  | 22 | 42896105 | 42896985 | PDFSTATSENSEMBLUCSC | 881 | 9 | 27500 | island | 0.13709 | 0.72908 | Not queried | 4.41 | 4.11e-05 | 2.11e-01 |
|  | 5 | 76925380 | 76925623 | PDFSTATSENSEMBLUCSC | 244 | 4 | 31598 | gene;island | 0.44053 | 0.05510 | Not queried | 4.41 | 2.00e-01 | 1.00e+00 |
|  | 5 | 140624869 | 140625389 | PDFSTATSENSEMBLUCSC | 521 | 10 | 33166 | island | 0.01933 | 0.35252 | Not queried | 4.41 | 1.05e-03 | 1.00e+00 |
|  | 6 | 26103952 | 26104165 | PDFSTATSENSEMBLUCSC | 214 | 5 | 34621 | tss | 0.03477 | 0.35770 | Not queried | 4.41 | 7.94e-03 | 1.00e+00 |
|  | 6 | 101328941 | 101329504 | PDFSTATSENSEMBLUCSC | 564 | 8 | 36307 | island | 0.53440 | 0.04743 | Not queried | 4.41 | 1.55e-04 | 7.99e-01 |
|  | 20 | 44258197 | 44258437 | PDFSTATSENSEMBLUCSC | 241 | 5 | 25393 | gene | 0.04004 | 0.45483 | Not queried | 4.41 | 7.94e-03 | 1.00e+00 |
|  | 8 | 97657553 | 97657833 | PDFSTATSENSEMBLUCSC | 281 | 4 | 39307 | gene | 0.06588 | 0.60095 | Not queried | 4.40 | 2.86e-02 | 1.00e+00 |
|  | 1 | 231296640 | 231297103 | PDFSTATSENSEMBLUCSC | 464 | 5 | 4310 | island | 0.85234 | 0.17515 | Not queried | 4.40 | 9.52e-02 | 1.00e+00 |
|  | 8 | 143695893 | 143696144 | PDFSTATSENSEMBLUCSC | 252 | 5 | 39867 | tss | 0.08110 | 0.64401 | Not queried | 4.40 | 7.94e-03 | 1.00e+00 |
|  | 14 | 52734156 | 52734529 | PDFSTATSENSEMBLUCSC | 374 | 8 | 12814 | island | 0.32329 | 0.90351 | Not queried | 4.40 | 1.55e-04 | 7.99e-01 |
|  | 14 | 52734156 | 52734397 | PDFSTATSENSEMBLUCSC | 242 | 6 | 12368 | tss | 0.33859 | 0.90351 | Not queried | 4.40 | 2.16e-03 | 1.00e+00 |
|  | 7 | 27291346 | 27291695 | PDFSTATSENSEMBLUCSC | 350 | 4 | 38303 | island | 0.84929 | 0.20045 | Not queried | 4.40 | 2.86e-02 | 1.00e+00 |
|  | 22 | 32599511 | 32599648 | PDFSTATSENSEMBLUCSC | 138 | 5 | 26805 | gene;tss | 0.23905 | 0.86877 | Not queried | 4.40 | 7.94e-03 | 1.00e+00 |
|  | 1 | 46958475 | 46958856 | PDFSTATSENSEMBLUCSC | 382 | 4 | 3214 | island | 0.82148 | 0.17650 | Not queried | 4.39 | 2.86e-02 | 1.00e+00 |
|  | 14 | 37125292 | 37125997 | PDFSTATSENSEMBLUCSC | 706 | 8 | 12332 | tss | 0.04101 | 0.61578 | Not queried | 4.39 | 2.95e-03 | 1.00e+00 |
|  | 14 | 37125004 | 37125997 | PDFSTATSENSEMBLUCSC | 994 | 10 | 12763 | island | 0.04101 | 0.61578 | Not queried | 4.39 | 2.88e-03 | 1.00e+00 |
|  | 1 | 16970920 | 16971585 | PDFSTATSENSEMBLUCSC | 666 | 8 | 1282 | tss;island | 0.02026 | 0.29688 | Not queried | 4.39 | 3.11e-04 | 1.00e+00 |
|  | 17 | 7284255 | 7284351 | PDFSTATSENSEMBLUCSC | 97 | 4 | 17044 | tss;island | 0.01943 | 0.33526 | Not queried | 4.39 | 2.86e-02 | 1.00e+00 |
|  | 13 | 50070436 | 50070520 | PDFSTATSENSEMBLUCSC | 85 | 5 | 11413 | tss | 0.51665 | 0.03036 | Not queried | 4.38 | 7.94e-03 | 1.00e+00 |
|  | 8 | 57358130 | 57358713 | PDFSTATSENSEMBLUCSC | 584 | 9 | 39251 | gene;island | 0.04290 | 0.51867 | Not queried | 4.38 | 4.11e-05 | 2.11e-01 |
|  | 5 | 140578896 | 140579303 | PDFSTATSENSEMBLUCSC | 408 | 7 | 32307 | tss | 0.02412 | 0.24048 | Not queried | 4.38 | 1.11e-02 | 1.00e+00 |
|  | 5 | 140578896 | 140579644 | PDFSTATSENSEMBLUCSC | 749 | 9 | 33161 | island | 0.02451 | 0.41143 | Not queried | 4.38 | 1.85e-03 | 1.00e+00 |
|  | 21 | 26934197 | 26934455 | PDFSTATSENSEMBLUCSC | 259 | 4 | 26455 | tss | 0.03410 | 0.47925 | Not queried | 4.38 | 2.86e-02 | 1.00e+00 |
|  | 6 | 13873924 | 13874251 | PDFSTATSENSEMBLUCSC | 328 | 4 | 35395 | island | 0.04969 | 0.49992 | Not queried | 4.38 | 2.86e-02 | 1.00e+00 |
|  | 1 | 50886782 | 50886969 | PDFSTATSENSEMBLUCSC | 188 | 4 | 524 | gene;island | 0.60432 | 0.08390 | Not queried | 4.38 | 2.86e-02 | 1.00e+00 |
|  | 3 | 181428046 | 181428697 | PDFSTATSENSEMBLUCSC | 652 | 15 | 27998 | gene;island | 0.43117 | 0.04126 | SOX2-OT; SOX2 (margin: SOX2-OT; SOX2) | 4.37 | 1.29e-08 | 6.63e-05 |
|  | 22 | 50523686 | 50524124 | PDFSTATSENSEMBLUCSC | 439 | 8 | 26881 | gene | 0.12856 | 0.59126 | Not queried | 4.37 | 3.11e-04 | 1.00e+00 |
|  | 1 | 144994542 | 144995101 | PDFSTATSENSEMBLUCSC | 560 | 6 | 718 | gene | 0.09677 | 0.69588 | Not queried | 4.36 | 2.16e-03 | 1.00e+00 |
|  | 16 | 80966033 | 80966491 | PDFSTATSENSEMBLUCSC | 459 | 6 | 16084 | island | 0.38661 | 0.02757 | Not queried | 4.36 | 2.16e-03 | 1.00e+00 |
|  | 1 | 217263507 | 217263847 | PDFSTATSENSEMBLUCSC | 341 | 4 | 1008 | gene;tss;island | 0.61107 | 0.13041 | Not queried | 4.36 | 1.14e-01 | 1.00e+00 |
|  | 11 | 76838498 | 76838749 | PDFSTATSENSEMBLUCSC | 252 | 5 | 7561 | tss | 0.01002 | 0.02887 | Not queried | 4.36 | 1.59e-02 | 1.00e+00 |
|  | 5 | 140529499 | 140529819 | PDFSTATSENSEMBLUCSC | 321 | 6 | 32304 | tss;island | 0.03619 | 0.37679 | Not queried | 4.36 | 2.16e-03 | 1.00e+00 |
|  | 10 | 104535792 | 104536121 | PDFSTATSENSEMBLUCSC | 330 | 9 | 4723 | gene | 0.03283 | 0.46505 | Not queried | 4.36 | 1.23e-03 | 1.00e+00 |
|  | 8 | 10586678 | 10587055 | PDFSTATSENSEMBLUCSC | 378 | 7 | 39136 | gene;island | 0.80246 | 0.14538 | Not queried | 4.36 | 5.83e-04 | 1.00e+00 |
|  | 13 | 114780772 | 114780992 | PDFSTATSENSEMBLUCSC | 221 | 4 | 11276 | gene;island | 0.19267 | 0.82753 | Not queried | 4.35 | 2.86e-02 | 1.00e+00 |
|  | 1 | 20878760 | 20878928 | PDFSTATSENSEMBLUCSC | 169 | 4 | 1311 | tss | 0.52427 | 0.05778 | Not queried | 4.35 | 5.71e-02 | 1.00e+00 |
|  | 1 | 53387430 | 53387719 | PDFSTATSENSEMBLUCSC | 290 | 4 | 3269 | island | 0.03856 | 0.49980 | Not queried | 4.35 | 2.00e-01 | 1.00e+00 |
|  | 5 | 143978290 | 143978420 | PDFSTATSENSEMBLUCSC | 131 | 4 | 33213 | island | 0.04528 | 0.49472 | Not queried | 4.35 | 2.86e-02 | 1.00e+00 |
|  | 2 | 66664535 | 66664960 | PDFSTATSENSEMBLUCSC | 426 | 5 | 22673 | gene;island | 0.69264 | 0.10669 | Not queried | 4.35 | 3.17e-02 | 1.00e+00 |
|  | 7 | 22539703 | 22540154 | PDFSTATSENSEMBLUCSC | 452 | 9 | 38236 | island | 0.03317 | 0.27603 | Not queried | 4.35 | 4.11e-05 | 2.11e-01 |
|  | 19 | 53811704 | 53812098 | PDFSTATSENSEMBLUCSC | 395 | 4 | 22338 | island | 0.11449 | 0.63778 | Not queried | 4.34 | 2.86e-02 | 1.00e+00 |
|  | 10 | 37414506 | 37414802 | PDFSTATSENSEMBLUCSC | 297 | 7 | 5650 | island | 0.14907 | 0.88112 | Not queried | 4.34 | 5.83e-04 | 1.00e+00 |
|  | 5 | 134181484 | 134181707 | PDFSTATSENSEMBLUCSC | 224 | 7 | 31695 | gene;island | 0.01110 | 0.29763 | Not queried | 4.34 | 5.83e-04 | 1.00e+00 |
|  | 10 | 118890977 | 118891355 | PDFSTATSENSEMBLUCSC | 379 | 4 | 4762 | gene;island | 0.66866 | 0.06663 | Not queried | 4.34 | 2.86e-02 | 1.00e+00 |
|  | 11 | 76839129 | 76839217 | PDFSTATSENSEMBLUCSC | 89 | 4 | 7562 | tss | 0.03751 | 0.38745 | Not queried | 4.34 | 2.86e-02 | 1.00e+00 |
|  | 11 | 58912052 | 58912240 | PDFSTATSENSEMBLUCSC | 189 | 5 | 7294 | tss | 0.73243 | 0.16012 | Not queried | 4.34 | 7.94e-03 | 1.00e+00 |
|  | 1 | 26233332 | 26233709 | PDFSTATSENSEMBLUCSC | 378 | 11 | 1376 | tss;island | 0.04786 | 0.49919 | STMN1; MIR3917 (margin: AL033528.1; STMN1; MIR3917) | 4.34 | 2.84e-06 | 1.46e-02 |
|  | X | 153707447 | 153707810 | PDFSTATSENSEMBLUCSC | 364 | 6 | 42147 | island | 0.07427 | 0.60370 | Not queried | 4.34 | 2.16e-03 | 1.00e+00 |
|  | 2 | 134326055 | 134326366 | PDFSTATSENSEMBLUCSC | 312 | 5 | 23603 | tss | 0.21414 | 0.66624 | Not queried | 4.33 | 7.94e-03 | 1.00e+00 |
|  | 11 | 113258210 | 113258455 | PDFSTATSENSEMBLUCSC | 246 | 6 | 7681 | tss;island | 0.23875 | 0.84583 | Not queried | 4.33 | 2.16e-03 | 1.00e+00 |
|  | 10 | 43818243 | 43818911 | PDFSTATSENSEMBLUCSC | 669 | 8 | 5675 | island | 0.94531 | 0.37936 | Not queried | 4.33 | 1.55e-04 | 7.99e-01 |
|  | 15 | 101587506 | 101587971 | PDFSTATSENSEMBLUCSC | 466 | 5 | 13478 | gene | 0.91970 | 0.13343 | Not queried | 4.33 | 3.17e-02 | 1.00e+00 |
|  | 5 | 80597143 | 80597395 | PDFSTATSENSEMBLUCSC | 253 | 8 | 32095 | tss | 0.44845 | 0.03230 | Not queried | 4.33 | 1.55e-04 | 7.99e-01 |
|  | X | 63615185 | 63615495 | PDFSTATSENSEMBLUCSC | 311 | 7 | 41828 | island | 0.05103 | 0.51885 | Not queried | 4.33 | 5.83e-04 | 1.00e+00 |
|  | 10 | 57390536 | 57391055 | PDFSTATSENSEMBLUCSC | 520 | 4 | 5736 | island | 0.11382 | 0.59593 | Not queried | 4.33 | 2.86e-02 | 1.00e+00 |
|  | 14 | 64855023 | 64855353 | PDFSTATSENSEMBLUCSC | 331 | 4 | 12083 | gene;island | 0.56960 | 0.04603 | Not queried | 4.32 | 2.86e-02 | 1.00e+00 |
|  | 19 | 51231557 | 51232007 | PDFSTATSENSEMBLUCSC | 451 | 5 | 22287 | island | 0.07933 | 0.63269 | Not queried | 4.32 | 1.51e-01 | 1.00e+00 |
|  | 1 | 63795636 | 63795970 | PDFSTATSENSEMBLUCSC | 335 | 5 | 3337 | island | 0.38572 | 0.04211 | Not queried | 4.32 | 7.94e-03 | 1.00e+00 |
|  | 4 | 41753439 | 41754125 | PDFSTATSENSEMBLUCSC | 687 | 8 | 30953 | island | 0.63366 | 0.10676 | Not queried | 4.32 | 1.55e-04 | 7.99e-01 |
|  | 22 | 38479099 | 38479332 | PDFSTATSENSEMBLUCSC | 234 | 7 | 27438 | island | 0.35993 | 0.85333 | Not queried | 4.31 | 5.83e-04 | 1.00e+00 |
|  | X | 54070741 | 54070878 | PDFSTATSENSEMBLUCSC | 138 | 4 | 41333 | tss;island | 0.03744 | 0.42020 | Not queried | 4.31 | 2.86e-02 | 1.00e+00 |
|  | 8 | 11560299 | 11560851 | PDFSTATSENSEMBLUCSC | 553 | 12 | 39489 | tss;island | 0.73098 | 0.16740 | GATA4 (margin: GATA4) | 4.31 | 7.40e-07 | 3.80e-03 |
|  | 3 | 53190593 | 53190763 | PDFSTATSENSEMBLUCSC | 171 | 4 | 29155 | island | 0.09276 | 0.61233 | Not queried | 4.31 | 5.71e-02 | 1.00e+00 |
|  | 3 | 181428242 | 181428697 | PDFSTATSENSEMBLUCSC | 456 | 14 | 28696 | tss | 0.41932 | 0.04117 | SOX2-OT; SOX2 (margin: SOX2-OT; SOX2) | 4.31 | 4.99e-08 | 2.56e-04 |
|  | 2 | 54342584 | 54342961 | PDFSTATSENSEMBLUCSC | 378 | 4 | 22645 | gene;island | 0.01963 | 0.28192 | Not queried | 4.31 | 2.86e-02 | 1.00e+00 |
|  | 13 | 28365587 | 28365853 | PDFSTATSENSEMBLUCSC | 267 | 4 | 11335 | tss;island | 0.03894 | 0.44491 | Not queried | 4.31 | 5.71e-02 | 1.00e+00 |
|  | 19 | 34174665 | 34175419 | PDFSTATSENSEMBLUCSC | 755 | 19 | 20652 | tss | 0.08304 | 0.73978 | CHST8 (margin: CHST8) | 4.31 | 9.13e-06 | 4.69e-02 |
|  | X | 68835489 | 68836202 | PDFSTATSENSEMBLUCSC | 714 | 11 | 41843 | island | 0.10783 | 0.01045 | Not queried | 4.30 | 3.94e-04 | 1.00e+00 |
|  | 3 | 148847007 | 148847295 | PDFSTATSENSEMBLUCSC | 289 | 6 | 28613 | tss;island | 0.03103 | 0.35905 | Not queried | 4.30 | 2.16e-03 | 1.00e+00 |
|  | 5 | 140613740 | 140614106 | PDFSTATSENSEMBLUCSC | 367 | 8 | 33164 | island | 0.02224 | 0.32781 | Not queried | 4.30 | 1.55e-04 | 7.99e-01 |
|  | 11 | 120008944 | 120009135 | PDFSTATSENSEMBLUCSC | 192 | 5 | 7736 | tss | 0.07884 | 0.62897 | Not queried | 4.30 | 9.52e-02 | 1.00e+00 |
|  | 5 | 179588440 | 179588838 | PDFSTATSENSEMBLUCSC | 399 | 4 | 31881 | gene;island | 0.24953 | 0.79882 | Not queried | 4.30 | 2.86e-02 | 1.00e+00 |
|  | 18 | 20735537 | 20735693 | PDFSTATSENSEMBLUCSC | 157 | 4 | 19038 | gene;tss | 0.13797 | 0.79952 | Not queried | 4.30 | 2.86e-02 | 1.00e+00 |
|  | X | 146992908 | 146993175 | PDFSTATSENSEMBLUCSC | 268 | 5 | 41133 | gene;tss;island | 0.04368 | 0.63945 | Not queried | 4.30 | 9.52e-02 | 1.00e+00 |
|  | 15 | 89910632 | 89910860 | PDFSTATSENSEMBLUCSC | 229 | 4 | 13858 | tss;island | 0.05553 | 0.54814 | Not queried | 4.30 | 5.71e-02 | 1.00e+00 |
|  | X | 63615339 | 63615495 | PDFSTATSENSEMBLUCSC | 157 | 6 | 41350 | tss | 0.05196 | 0.52857 | Not queried | 4.30 | 2.16e-03 | 1.00e+00 |
|  | X | 53350524 | 53350693 | PDFSTATSENSEMBLUCSC | 170 | 5 | 41329 | tss | 0.03147 | 0.42557 | Not queried | 4.29 | 7.94e-03 | 1.00e+00 |
|  | 11 | 1660843 | 1661201 | PDFSTATSENSEMBLUCSC | 359 | 4 | 6408 | gene | 0.08136 | 0.69897 | Not queried | 4.29 | 2.86e-02 | 1.00e+00 |
|  | 6 | 49755009 | 49755190 | PDFSTATSENSEMBLUCSC | 182 | 4 | 34964 | tss | 0.17584 | 0.76777 | Not queried | 4.29 | 2.86e-02 | 1.00e+00 |
|  | 10 | 130063978 | 130064296 | PDFSTATSENSEMBLUCSC | 319 | 4 | 6236 | island | 0.60455 | 0.09218 | Not queried | 4.29 | 2.86e-02 | 1.00e+00 |
|  | 6 | 138200071 | 138200387 | PDFSTATSENSEMBLUCSC | 317 | 6 | 34422 | gene | 0.10684 | 0.78070 | Not queried | 4.29 | 1.52e-02 | 1.00e+00 |
|  | 6 | 32116207 | 32116317 | PDFSTATSENSEMBLUCSC | 111 | 4 | 33994 | gene | 0.08125 | 0.58213 | Not queried | 4.28 | 2.86e-02 | 1.00e+00 |
|  | 5 | 140810051 | 140811312 | PDFSTATSENSEMBLUCSC | 1262 | 16 | 31779 | gene;island | 0.34269 | 0.89506 | PCDHGA1; PCDHGA2; PCDHGA3; PCDHGB1; PCDHGA4; PCDHGB2; PCDHGA5; PCDHGB3; PCDHGA6; PCDHGA7; PCDHGB4; PCDHGA8; PCDHGA9; PCDHGB6; PCDHGA10; PCDHGB7; PCDHGA11; PCDHGB8P; PCDHGA12 (margin: PCDHGA1; PCDHGA2; PCDHGA3; PCDHGB1; PCDHGA4; PCDHGB2; PCDHGA5; PCDHGB3; PCDHGA6; PCDHGA7; PCDHGB4; PCDHGA8; PCDHGA9; PCDHGB6; PCDHGA10; PCDHGB7; PCDHGA11; PCDHGB8P; PCDHGA12) | 4.28 | 1.33e-08 | 6.84e-05 |
|  | 11 | 65222227 | 65222786 | PDFSTATSENSEMBLUCSC | 560 | 6 | 8435 | island | 0.23480 | 0.69788 | Not queried | 4.28 | 6.49e-02 | 1.00e+00 |
|  | 6 | 122931154 | 122931702 | PDFSTATSENSEMBLUCSC | 549 | 11 | 34398 | gene;island | 0.06450 | 0.57169 | PKIB (margin: PKIB) | 4.28 | 2.84e-06 | 1.46e-02 |
|  | 3 | 44626029 | 44626860 | PDFSTATSENSEMBLUCSC | 832 | 11 | 28993 | island | 0.31663 | 0.03272 | ZKSCAN7; ZNF660; ZNF197; RP11-944L7.4 (margin: ZKSCAN7; ZNF660; ZNF197; RP11-944L7.4; MPRIPP1) | 4.27 | 5.67e-06 | 2.91e-02 |
|  | 5 | 140624869 | 140625130 | PDFSTATSENSEMBLUCSC | 262 | 7 | 32310 | tss | 0.02196 | 0.21950 | Not queried | 4.27 | 5.83e-04 | 1.00e+00 |
|  | 15 | 51385812 | 51385970 | PDFSTATSENSEMBLUCSC | 159 | 4 | 13319 | gene;island | 0.86648 | 0.21100 | Not queried | 4.27 | 5.71e-02 | 1.00e+00 |
|  | 2 | 19551750 | 19551944 | PDFSTATSENSEMBLUCSC | 195 | 4 | 22549 | gene;island | 0.81797 | 0.13045 | Not queried | 4.27 | 2.86e-02 | 1.00e+00 |
|  | 17 | 55938748 | 55939089 | PDFSTATSENSEMBLUCSC | 342 | 4 | 18553 | island | 0.06814 | 0.59346 | Not queried | 4.27 | 2.86e-02 | 1.00e+00 |
|  | 12 | 46663270 | 46663407 | PDFSTATSENSEMBLUCSC | 138 | 5 | 9632 | tss;island | 0.36027 | 0.91368 | Not queried | 4.27 | 7.94e-03 | 1.00e+00 |
|  | 17 | 45949677 | 45949878 | PDFSTATSENSEMBLUCSC | 202 | 5 | 18430 | island | 0.58980 | 0.08294 | Not queried | 4.27 | 7.94e-03 | 1.00e+00 |
|  | 8 | 70946891 | 70947218 | PDFSTATSENSEMBLUCSC | 328 | 4 | 40308 | island | 0.60007 | 0.08043 | Not queried | 4.27 | 2.86e-02 | 1.00e+00 |
|  | 2 | 198650603 | 198651076 | PDFSTATSENSEMBLUCSC | 474 | 6 | 23764 | tss;island | 0.05090 | 0.49960 | Not queried | 4.26 | 4.11e-02 | 1.00e+00 |
|  | 6 | 32133929 | 32135396 | PDFSTATSENSEMBLUCSC | 1468 | 44 | 34002 | gene;island | 0.30898 | 0.88071 | PPT2; PPT2-EGFL8; EGFL8; AGPAT1 (margin: PPT2; PPT2-EGFL8; EGFL8; RNF5; PRRT1; AGPAT1) | 4.26 | 1.57e-13 | 8.07e-10 |
|  | 11 | 61159602 | 61159837 | PDFSTATSENSEMBLUCSC | 236 | 7 | 7313 | tss;island | 0.79836 | 0.12041 | Not queried | 4.26 | 1.17e-03 | 1.00e+00 |
|  | 2 | 628881 | 629121 | PDFSTATSENSEMBLUCSC | 241 | 5 | 23971 | island | 0.02553 | 0.37299 | Not queried | 4.26 | 7.94e-03 | 1.00e+00 |
|  | 2 | 171568791 | 171569381 | PDFSTATSENSEMBLUCSC | 591 | 7 | 24807 | island | 0.69116 | 0.08981 | Not queried | 4.26 | 1.75e-02 | 1.00e+00 |
|  | 1 | 19615303 | 19615702 | PDFSTATSENSEMBLUCSC | 400 | 11 | 1298 | tss;island | 0.11750 | 0.81759 | AKR7A3 (margin: RP4-657E11.10; AKR7A3) | 4.26 | 2.84e-06 | 1.46e-02 |
|  | 2 | 9614471 | 9614578 | PDFSTATSENSEMBLUCSC | 108 | 5 | 23135 | tss | 0.44460 | 0.93695 | Not queried | 4.26 | 7.94e-03 | 1.00e+00 |
|  | X | 46404528 | 46404864 | PDFSTATSENSEMBLUCSC | 337 | 6 | 40970 | gene | 0.06298 | 0.60108 | Not queried | 4.26 | 2.16e-03 | 1.00e+00 |
|  | 19 | 31210456 | 31210714 | PDFSTATSENSEMBLUCSC | 259 | 4 | 21826 | island | 0.12683 | 0.68635 | Not queried | 4.26 | 2.86e-02 | 1.00e+00 |
|  | 6 | 292329 | 292596 | PDFSTATSENSEMBLUCSC | 268 | 4 | 33465 | gene;island | 0.05499 | 0.48873 | Not queried | 4.25 | 2.86e-02 | 1.00e+00 |
|  | 1 | 55445834 | 55446708 | PDFSTATSENSEMBLUCSC | 875 | 10 | 3297 | island | 0.10712 | 0.74370 | Not queried | 4.25 | 2.09e-03 | 1.00e+00 |
|  | 12 | 51566731 | 51567112 | PDFSTATSENSEMBLUCSC | 382 | 9 | 9687 | tss | 0.44980 | 0.03709 | Not queried | 4.25 | 4.11e-05 | 2.11e-01 |
|  | 11 | 65190135 | 65190227 | PDFSTATSENSEMBLUCSC | 93 | 7 | 7408 | tss;island | 0.10632 | 0.77132 | Not queried | 4.24 | 5.83e-04 | 1.00e+00 |
|  | 19 | 11784246 | 11785337 | PDFSTATSENSEMBLUCSC | 1092 | 15 | 21544 | island | 0.03751 | 0.51186 | ZNF833P (margin: ZNF833P; CTC-499B15.4; HNRNPA1P10) | 4.24 | 2.45e-07 | 1.26e-03 |
|  | 5 | 140220686 | 140221106 | PDFSTATSENSEMBLUCSC | 421 | 6 | 31742 | gene;island | 0.27128 | 0.86740 | Not queried | 4.24 | 2.16e-03 | 1.00e+00 |
|  | 12 | 97300410 | 97300582 | PDFSTATSENSEMBLUCSC | 173 | 4 | 9907 | tss;island | 0.67846 | 0.96519 | Not queried | 4.23 | 2.86e-02 | 1.00e+00 |
|  | 19 | 6463949 | 6464214 | PDFSTATSENSEMBLUCSC | 266 | 9 | 20354 | tss | 0.02467 | 0.46467 | Not queried | 4.23 | 8.23e-05 | 4.23e-01 |
|  | 2 | 229046406 | 229046785 | PDFSTATSENSEMBLUCSC | 380 | 4 | 23895 | tss | 0.66705 | 0.08830 | Not queried | 4.23 | 2.00e-01 | 1.00e+00 |
|  | 17 | 46659751 | 46660002 | PDFSTATSENSEMBLUCSC | 252 | 4 | 18449 | island | 0.50626 | 0.11141 | Not queried | 4.22 | 1.14e-01 | 1.00e+00 |
|  | 3 | 140813769 | 140814133 | PDFSTATSENSEMBLUCSC | 365 | 4 | 27929 | gene;island | 0.03059 | 0.39665 | Not queried | 4.22 | 1.14e-01 | 1.00e+00 |
|  | 5 | 54281198 | 54281362 | PDFSTATSENSEMBLUCSC | 165 | 4 | 31558 | gene | 0.02976 | 0.37237 | Not queried | 4.22 | 2.86e-02 | 1.00e+00 |
|  | 20 | 61847579 | 61847878 | PDFSTATSENSEMBLUCSC | 300 | 8 | 25851 | tss;island | 0.02385 | 0.23316 | Not queried | 4.22 | 1.09e-03 | 1.00e+00 |
|  | 3 | 62355319 | 62355525 | PDFSTATSENSEMBLUCSC | 207 | 4 | 29203 | island | 0.77491 | 0.13829 | Not queried | 4.21 | 2.86e-02 | 1.00e+00 |
|  | 12 | 122231614 | 122231927 | PDFSTATSENSEMBLUCSC | 314 | 6 | 10051 | tss | 0.03116 | 0.40493 | Not queried | 4.21 | 2.16e-03 | 1.00e+00 |
|  | 8 | 144852570 | 144853338 | PDFSTATSENSEMBLUCSC | 769 | 7 | 40622 | island | 0.64413 | 0.05249 | Not queried | 4.21 | 2.33e-03 | 1.00e+00 |
|  | 7 | 1329387 | 1329592 | PDFSTATSENSEMBLUCSC | 206 | 5 | 38033 | island | 0.33030 | 0.93582 | Not queried | 4.21 | 7.94e-03 | 1.00e+00 |
|  | 6 | 25992047 | 25992401 | PDFSTATSENSEMBLUCSC | 355 | 4 | 35440 | island | 0.07156 | 0.45830 | Not queried | 4.21 | 2.86e-02 | 1.00e+00 |
|  | 17 | 46805785 | 46806134 | PDFSTATSENSEMBLUCSC | 350 | 5 | 18469 | island | 0.03782 | 0.28130 | Not queried | 4.20 | 5.56e-02 | 1.00e+00 |
|  | 17 | 40274524 | 40274811 | PDFSTATSENSEMBLUCSC | 288 | 7 | 17321 | tss;island | 0.09753 | 0.66538 | Not queried | 4.20 | 5.83e-04 | 1.00e+00 |
|  | 2 | 241497412 | 241497846 | PDFSTATSENSEMBLUCSC | 435 | 8 | 23943 | tss;island | 0.57153 | 0.07070 | Not queried | 4.20 | 4.66e-03 | 1.00e+00 |
|  | 5 | 140235435 | 140235607 | PDFSTATSENSEMBLUCSC | 173 | 5 | 32296 | tss | 0.08742 | 0.62538 | Not queried | 4.20 | 7.94e-03 | 1.00e+00 |
|  | 6 | 90660913 | 90661373 | PDFSTATSENSEMBLUCSC | 461 | 5 | 34351 | gene | 0.32682 | 0.82532 | Not queried | 4.19 | 7.94e-03 | 1.00e+00 |
|  | 6 | 29717917 | 29718119 | PDFSTATSENSEMBLUCSC | 203 | 4 | 34690 | tss;island | 0.71414 | 0.10953 | Not queried | 4.19 | 2.86e-02 | 1.00e+00 |
|  | 1 | 228225533 | 228225886 | PDFSTATSENSEMBLUCSC | 354 | 5 | 1042 | gene;island | 0.06634 | 0.54695 | Not queried | 4.19 | 7.94e-03 | 1.00e+00 |
|  | 9 | 130700866 | 130701076 | PDFSTATSENSEMBLUCSC | 211 | 4 | 40776 | tss;island | 0.03151 | 0.61917 | Not queried | 4.18 | 5.71e-02 | 1.00e+00 |
|  | 18 | 52626476 | 52626848 | PDFSTATSENSEMBLUCSC | 373 | 5 | 19252 | tss;island | 0.03715 | 0.44394 | Not queried | 4.18 | 5.56e-02 | 1.00e+00 |
|  | 2 | 232260116 | 232260305 | PDFSTATSENSEMBLUCSC | 190 | 4 | 23901 | tss;island | 0.04722 | 0.46027 | Not queried | 4.18 | 2.86e-02 | 1.00e+00 |
|  | 16 | 90148388 | 90148962 | PDFSTATSENSEMBLUCSC | 575 | 5 | 16247 | island | 0.01243 | 0.39955 | Not queried | 4.17 | 3.17e-02 | 1.00e+00 |
|  | 2 | 85811292 | 85811471 | PDFSTATSENSEMBLUCSC | 180 | 4 | 23445 | tss | 0.81584 | 0.23183 | Not queried | 4.17 | 2.86e-02 | 1.00e+00 |
|  | 6 | 111580120 | 111580469 | PDFSTATSENSEMBLUCSC | 350 | 9 | 35112 | tss | 0.43511 | 0.02931 | Not queried | 4.17 | 2.76e-03 | 1.00e+00 |
|  | 17 | 46641708 | 46642104 | PDFSTATSENSEMBLUCSC | 397 | 4 | 16617 | gene;island | 0.46726 | 0.04857 | Not queried | 4.17 | 2.86e-02 | 1.00e+00 |
|  | 22 | 39378224 | 39378382 | PDFSTATSENSEMBLUCSC | 159 | 4 | 27106 | tss | 0.81294 | 0.27464 | Not queried | 4.17 | 2.86e-02 | 1.00e+00 |
|  | 5 | 140857474 | 140858107 | PDFSTATSENSEMBLUCSC | 634 | 8 | 31781 | gene;island | 0.05060 | 0.66636 | Not queried | 4.17 | 3.79e-02 | 1.00e+00 |
|  | 1 | 91191583 | 91192162 | PDFSTATSENSEMBLUCSC | 580 | 6 | 3456 | island | 0.26052 | 0.01147 | Not queried | 4.16 | 1.52e-02 | 1.00e+00 |
|  | 5 | 1295737 | 1296007 | PDFSTATSENSEMBLUCSC | 271 | 5 | 31910 | tss;island | 0.84446 | 0.35519 | Not queried | 4.16 | 3.17e-02 | 1.00e+00 |
|  | 8 | 145028093 | 145028505 | PDFSTATSENSEMBLUCSC | 413 | 9 | 39903 | tss | 0.58388 | 0.04045 | Not queried | 4.16 | 7.77e-03 | 1.00e+00 |
|  | 1 | 67217673 | 67218275 | PDFSTATSENSEMBLUCSC | 603 | 12 | 3354 | island | 0.02380 | 0.35889 | TCTEX1D1 (margin: SGIP1; TCTEX1D1) | 4.16 | 7.40e-07 | 3.80e-03 |
|  | 1 | 67217673 | 67218135 | PDFSTATSENSEMBLUCSC | 463 | 10 | 1677 | tss | 0.02380 | 0.35693 | Not queried | 4.16 | 1.08e-05 | 5.56e-02 |
|  | 19 | 56915403 | 56915879 | PDFSTATSENSEMBLUCSC | 477 | 9 | 20199 | gene;island | 0.53309 | 0.04938 | Not queried | 4.16 | 4.11e-05 | 2.11e-01 |
|  | 7 | 55000889 | 55001207 | PDFSTATSENSEMBLUCSC | 319 | 4 | 38438 | island | 0.02463 | 0.41266 | Not queried | 4.16 | 5.71e-02 | 1.00e+00 |
|  | 15 | 45421578 | 45422095 | PDFSTATSENSEMBLUCSC | 518 | 11 | 13618 | tss;island | 0.08782 | 0.58619 | Not queried | 4.16 | 8.51e-05 | 4.37e-01 |
|  | 3 | 133464463 | 133464971 | PDFSTATSENSEMBLUCSC | 509 | 6 | 28559 | tss;island | 0.41679 | 0.91096 | Not queried | 4.16 | 2.60e-02 | 1.00e+00 |
|  | 17 | 76210025 | 76210520 | PDFSTATSENSEMBLUCSC | 496 | 6 | 18784 | island | 0.03765 | 0.45910 | Not queried | 4.16 | 1.52e-02 | 1.00e+00 |
|  | 11 | 49455851 | 49456196 | PDFSTATSENSEMBLUCSC | 346 | 4 | 8254 | island | 0.21625 | 0.82617 | Not queried | 4.16 | 5.71e-02 | 1.00e+00 |
|  | 3 | 8809306 | 8809715 | PDFSTATSENSEMBLUCSC | 410 | 4 | 27604 | gene;island | 0.18181 | 0.86461 | Not queried | 4.15 | 2.86e-02 | 1.00e+00 |
|  | 16 | 56701994 | 56702158 | PDFSTATSENSEMBLUCSC | 165 | 5 | 15220 | tss | 0.13489 | 0.73306 | Not queried | 4.15 | 7.94e-03 | 1.00e+00 |
|  | 16 | 56701868 | 56702158 | PDFSTATSENSEMBLUCSC | 291 | 7 | 15921 | island | 0.13489 | 0.73306 | Not queried | 4.15 | 5.83e-04 | 1.00e+00 |
|  | 8 | 6692785 | 6693540 | PDFSTATSENSEMBLUCSC | 756 | 10 | 39990 | island | 0.10103 | 0.79271 | Not queried | 4.15 | 7.25e-04 | 1.00e+00 |
|  | 3 | 147077127 | 147077774 | PDFSTATSENSEMBLUCSC | 648 | 9 | 29522 | island | 0.66074 | 0.23629 | Not queried | 4.15 | 4.11e-05 | 2.11e-01 |
|  | 12 | 122231455 | 122231927 | PDFSTATSENSEMBLUCSC | 473 | 7 | 10890 | island | 0.03483 | 0.43080 | Not queried | 4.14 | 5.83e-04 | 1.00e+00 |
|  | 4 | 38869436 | 38869779 | PDFSTATSENSEMBLUCSC | 344 | 8 | 29934 | gene | 0.37171 | 0.02956 | Not queried | 4.14 | 2.95e-03 | 1.00e+00 |
|  | 19 | 40972228 | 40972683 | PDFSTATSENSEMBLUCSC | 456 | 4 | 20755 | tss;island | 0.08485 | 0.68587 | Not queried | 4.14 | 2.00e-01 | 1.00e+00 |
|  | 5 | 178368071 | 178368415 | PDFSTATSENSEMBLUCSC | 345 | 8 | 33412 | island | 0.39418 | 0.02452 | Not queried | 4.14 | 1.55e-04 | 7.99e-01 |
|  | 5 | 178368071 | 178368205 | PDFSTATSENSEMBLUCSC | 135 | 6 | 32485 | tss | 0.37367 | 0.02452 | Not queried | 4.14 | 2.16e-03 | 1.00e+00 |
|  | 12 | 115105220 | 115105710 | PDFSTATSENSEMBLUCSC | 491 | 5 | 10817 | island | 0.55553 | 0.04133 | Not queried | 4.14 | 1.59e-02 | 1.00e+00 |
|  | 19 | 7553700 | 7554201 | PDFSTATSENSEMBLUCSC | 502 | 9 | 21413 | island | 0.04998 | 0.47408 | Not queried | 4.14 | 1.06e-02 | 1.00e+00 |
|  | 7 | 27154262 | 27155548 | PDFSTATSENSEMBLUCSC | 1287 | 18 | 36868 | gene;island | 0.70517 | 0.08093 | HOXA-AS2; HOXA3 (margin: HOXA-AS2; HOXA2; HOXA3) | 4.14 | 9.92e-09 | 5.10e-05 |
|  | 4 | 1167230 | 1167423 | PDFSTATSENSEMBLUCSC | 194 | 5 | 29815 | gene;tss;island | 0.14766 | 0.88294 | Not queried | 4.14 | 7.94e-03 | 1.00e+00 |
|  | 8 | 8580376 | 8580960 | PDFSTATSENSEMBLUCSC | 585 | 6 | 39993 | island | 0.06475 | 0.60774 | Not queried | 4.14 | 4.11e-02 | 1.00e+00 |
|  | 10 | 135050619 | 135051351 | PDFSTATSENSEMBLUCSC | 733 | 25 | 5438 | tss | 0.32901 | 0.02832 | VENTX (margin: KNDC1; UTF1; VENTX; MIR202) | 4.14 | 1.90e-13 | 9.76e-10 |
|  | X | 152938750 | 152938907 | PDFSTATSENSEMBLUCSC | 158 | 4 | 41582 | tss | 0.25675 | 0.80746 | Not queried | 4.13 | 2.86e-02 | 1.00e+00 |
|  | 3 | 159756648 | 159757071 | PDFSTATSENSEMBLUCSC | 424 | 6 | 29582 | island | 0.44445 | 0.06295 | Not queried | 4.13 | 2.16e-03 | 1.00e+00 |
|  | 1 | 176998705 | 176999034 | PDFSTATSENSEMBLUCSC | 330 | 4 | 894 | gene;tss | 0.18178 | 0.67190 | Not queried | 4.13 | 1.14e-01 | 1.00e+00 |
|  | 4 | 190861788 | 190862268 | PDFSTATSENSEMBLUCSC | 481 | 10 | 31440 | island | 0.02123 | 0.34614 | Not queried | 4.13 | 1.30e-04 | 6.68e-01 |
|  | Y | 22737391 | 22737594 | PDFSTATSENSEMBLUCSC | 204 | 6 | 42173 | tss | 0.02924 | 0.33019 | Not queried | 4.13 | 2.16e-03 | 1.00e+00 |
|  | 8 | 97657072 | 97657833 | PDFSTATSENSEMBLUCSC | 762 | 14 | 40410 | island | 0.07953 | 0.57201 | CPQ (margin: CPQ) | 4.13 | 4.99e-08 | 2.56e-04 |
|  | 1 | 156182601 | 156183160 | PDFSTATSENSEMBLUCSC | 560 | 8 | 3836 | island | 0.44794 | 0.03817 | Not queried | 4.12 | 1.55e-04 | 7.99e-01 |
|  | 1 | 12538341 | 12538678 | PDFSTATSENSEMBLUCSC | 338 | 4 | 232 | gene | 0.17887 | 0.78850 | Not queried | 4.12 | 2.86e-02 | 1.00e+00 |
|  | 11 | 62211990 | 62212195 | PDFSTATSENSEMBLUCSC | 206 | 4 | 6618 | gene;island | 0.70585 | 0.13263 | Not queried | 4.12 | 2.86e-02 | 1.00e+00 |
|  | 6 | 32063394 | 32064258 | PDFSTATSENSEMBLUCSC | 865 | 28 | 33984 | gene;island | 0.37747 | 0.92532 | TNXB; ATF6B (margin: TNXB; ATF6B) | 4.12 | 7.11e-09 | 3.65e-05 |
|  | 6 | 24583477 | 24583848 | PDFSTATSENSEMBLUCSC | 372 | 5 | 33528 | gene | 0.27676 | 0.03913 | Not queried | 4.12 | 9.52e-02 | 1.00e+00 |
|  | 15 | 52404887 | 52405162 | PDFSTATSENSEMBLUCSC | 276 | 8 | 14119 | island | 0.15477 | 0.87139 | Not queried | 4.12 | 2.07e-02 | 1.00e+00 |
|  | 4 | 53010 | 53548 | PDFSTATSENSEMBLUCSC | 539 | 10 | 30665 | island | 0.03290 | 0.40757 | Not queried | 4.12 | 1.08e-05 | 5.56e-02 |
|  | 4 | 53010 | 53270 | PDFSTATSENSEMBLUCSC | 261 | 8 | 30174 | tss | 0.03290 | 0.40757 | Not queried | 4.12 | 1.55e-04 | 7.99e-01 |
|  | 6 | 78176473 | 78176847 | PDFSTATSENSEMBLUCSC | 375 | 6 | 36208 | island | 0.51367 | 0.05423 | Not queried | 4.11 | 2.60e-02 | 1.00e+00 |
|  | 3 | 21447391 | 21447667 | PDFSTATSENSEMBLUCSC | 277 | 4 | 27657 | gene | 0.22866 | 0.86197 | Not queried | 4.11 | 2.86e-02 | 1.00e+00 |
|  | 1 | 26856408 | 26856648 | PDFSTATSENSEMBLUCSC | 241 | 4 | 341 | gene;island | 0.03046 | 0.33597 | Not queried | 4.11 | 2.86e-02 | 1.00e+00 |
|  | 6 | 160210606 | 160211010 | PDFSTATSENSEMBLUCSC | 405 | 10 | 35258 | tss;island | 0.49134 | 0.05351 | Not queried | 4.11 | 1.08e-05 | 5.56e-02 |
|  | 14 | 24563410 | 24563578 | PDFSTATSENSEMBLUCSC | 169 | 4 | 12697 | island | 0.03033 | 0.24694 | Not queried | 4.11 | 2.86e-02 | 1.00e+00 |
|  | 7 | 22539908 | 22540154 | PDFSTATSENSEMBLUCSC | 247 | 5 | 37400 | tss | 0.03317 | 0.37217 | Not queried | 4.11 | 7.94e-03 | 1.00e+00 |
|  | 7 | 101005832 | 101006089 | PDFSTATSENSEMBLUCSC | 258 | 9 | 37737 | tss;island | 0.01202 | 0.18574 | Not queried | 4.11 | 2.88e-04 | 1.00e+00 |
|  | 7 | 2208345 | 2208908 | PDFSTATSENSEMBLUCSC | 564 | 6 | 36732 | gene | 0.46695 | 0.93835 | Not queried | 4.11 | 2.16e-03 | 1.00e+00 |
|  | 6 | 168418276 | 168418527 | PDFSTATSENSEMBLUCSC | 252 | 7 | 35282 | tss | 0.38375 | 0.87094 | Not queried | 4.10 | 3.79e-02 | 1.00e+00 |
|  | 19 | 49340489 | 49340765 | PDFSTATSENSEMBLUCSC | 277 | 4 | 20102 | gene;tss;island | 0.07746 | 0.50836 | Not queried | 4.10 | 2.86e-02 | 1.00e+00 |
|  | 4 | 206112 | 206562 | PDFSTATSENSEMBLUCSC | 451 | 8 | 30666 | island | 0.82672 | 0.18385 | Not queried | 4.10 | 1.86e-03 | 1.00e+00 |
|  | 22 | 17565455 | 17565904 | PDFSTATSENSEMBLUCSC | 450 | 9 | 27229 | island | 0.33490 | 0.03178 | Not queried | 4.10 | 2.44e-02 | 1.00e+00 |
|  | 6 | 29855110 | 29855347 | PDFSTATSENSEMBLUCSC | 238 | 7 | 34692 | tss | 0.85167 | 0.16228 | Not queried | 4.10 | 4.08e-03 | 1.00e+00 |
|  | 1 | 117112616 | 117112854 | PDFSTATSENSEMBLUCSC | 239 | 4 | 707 | gene;island | 0.60279 | 0.06211 | Not queried | 4.10 | 2.86e-02 | 1.00e+00 |
|  | 1 | 201368738 | 201369031 | PDFSTATSENSEMBLUCSC | 294 | 8 | 2162 | tss | 0.02285 | 0.29455 | Not queried | 4.10 | 1.55e-04 | 7.99e-01 |
|  | 19 | 14016603 | 14016822 | PDFSTATSENSEMBLUCSC | 220 | 7 | 19851 | gene | 0.52197 | 0.02171 | Not queried | 4.09 | 3.18e-01 | 1.00e+00 |
|  | 17 | 18011879 | 18012014 | PDFSTATSENSEMBLUCSC | 136 | 5 | 17139 | tss | 0.10198 | 0.65898 | Not queried | 4.09 | 7.94e-03 | 1.00e+00 |
|  | 10 | 135050619 | 135051581 | PDFSTATSENSEMBLUCSC | 963 | 28 | 6330 | island | 0.33540 | 0.02906 | VENTX (margin: KNDC1; UTF1; VENTX; MIR202) | 4.09 | 1.03e-09 | 5.31e-06 |
|  | 5 | 135416331 | 135416613 | PDFSTATSENSEMBLUCSC | 283 | 9 | 32251 | tss | 0.06942 | 0.55838 | Not queried | 4.09 | 4.11e-05 | 2.11e-01 |
|  | 7 | 20818657 | 20818928 | PDFSTATSENSEMBLUCSC | 272 | 6 | 38228 | island | 0.53274 | 0.06662 | Not queried | 4.09 | 2.16e-03 | 1.00e+00 |
|  | 16 | 88590325 | 88590467 | PDFSTATSENSEMBLUCSC | 143 | 5 | 14877 | gene;island | 0.32381 | 0.89093 | Not queried | 4.09 | 7.94e-03 | 1.00e+00 |
|  | 17 | 39041110 | 39041266 | PDFSTATSENSEMBLUCSC | 157 | 4 | 16534 | gene | 0.09222 | 0.61049 | Not queried | 4.09 | 2.86e-02 | 1.00e+00 |
|  | 17 | 48585858 | 48586147 | PDFSTATSENSEMBLUCSC | 290 | 4 | 16653 | gene;island | 0.41344 | 0.04342 | Not queried | 4.09 | 2.86e-02 | 1.00e+00 |
|  | 1 | 13909522 | 13909821 | PDFSTATSENSEMBLUCSC | 300 | 4 | 1257 | tss;island | 0.08018 | 0.50670 | Not queried | 4.09 | 2.86e-02 | 1.00e+00 |
|  | 20 | 44657637 | 44657812 | PDFSTATSENSEMBLUCSC | 176 | 5 | 25748 | tss | 0.22158 | 0.82960 | Not queried | 4.09 | 7.94e-03 | 1.00e+00 |
|  | 19 | 6463949 | 6464275 | PDFSTATSENSEMBLUCSC | 327 | 10 | 21394 | island | 0.02358 | 0.42233 | Not queried | 4.09 | 7.58e-05 | 3.89e-01 |
|  | 7 | 88424985 | 88425148 | PDFSTATSENSEMBLUCSC | 164 | 6 | 37016 | gene | 0.18686 | 0.71301 | Not queried | 4.08 | 2.16e-03 | 1.00e+00 |
|  | 3 | 99904510 | 99904650 | PDFSTATSENSEMBLUCSC | 141 | 4 | 28422 | tss | 0.68247 | 0.95697 | Not queried | 4.08 | 2.86e-02 | 1.00e+00 |
|  | 6 | 41528198 | 41528785 | PDFSTATSENSEMBLUCSC | 588 | 8 | 34264 | gene;island | 0.51785 | 0.07539 | Not queried | 4.08 | 6.99e-03 | 1.00e+00 |
|  | 19 | 11784955 | 11785337 | PDFSTATSENSEMBLUCSC | 383 | 6 | 19812 | gene | 0.06941 | 0.57172 | Not queried | 4.08 | 2.16e-03 | 1.00e+00 |
|  | 19 | 52035146 | 52035287 | PDFSTATSENSEMBLUCSC | 142 | 4 | 20961 | tss | 0.06897 | 0.46861 | Not queried | 4.08 | 1.14e-01 | 1.00e+00 |
|  | 3 | 46742703 | 46743199 | PDFSTATSENSEMBLUCSC | 497 | 5 | 29015 | island | 0.36378 | 0.91206 | Not queried | 4.07 | 7.94e-03 | 1.00e+00 |
|  | 7 | 102553293 | 102553457 | PDFSTATSENSEMBLUCSC | 165 | 5 | 37106 | gene | 0.30607 | 0.87535 | Not queried | 4.07 | 7.94e-03 | 1.00e+00 |
|  | 3 | 62362660 | 62363248 | PDFSTATSENSEMBLUCSC | 589 | 5 | 29207 | island | 0.86910 | 0.16967 | Not queried | 4.07 | 1.59e-02 | 1.00e+00 |
|  | 14 | 104928289 | 104928675 | PDFSTATSENSEMBLUCSC | 387 | 4 | 13176 | island | 0.03611 | 0.52953 | Not queried | 4.07 | 5.71e-02 | 1.00e+00 |
|  | 3 | 44626029 | 44626453 | PDFSTATSENSEMBLUCSC | 425 | 7 | 28215 | tss | 0.28186 | 0.03272 | Not queried | 4.07 | 1.17e-03 | 1.00e+00 |
|  | X | 75392480 | 75392755 | PDFSTATSENSEMBLUCSC | 276 | 4 | 41398 | tss | 0.47927 | 0.04004 | Not queried | 4.07 | 3.43e-01 | 1.00e+00 |
|  | 2 | 164204628 | 164205343 | PDFSTATSENSEMBLUCSC | 716 | 7 | 24789 | island | 0.04254 | 0.52157 | Not queried | 4.07 | 5.83e-04 | 1.00e+00 |
|  | 18 | 19752292 | 19752414 | PDFSTATSENSEMBLUCSC | 123 | 4 | 19035 | gene;island | 0.90414 | 0.37035 | Not queried | 4.06 | 2.86e-02 | 1.00e+00 |
|  | 22 | 35937058 | 35937319 | PDFSTATSENSEMBLUCSC | 262 | 6 | 27058 | tss | 0.59852 | 0.03261 | Not queried | 4.06 | 8.66e-03 | 1.00e+00 |
|  | 7 | 150497065 | 150497760 | PDFSTATSENSEMBLUCSC | 696 | 11 | 37214 | gene;tss;island | 0.30002 | 0.85608 | TMEM176A; TMEM176B (margin: RP5-1051J4.4; TMEM176A; TMEM176B) | 4.06 | 2.84e-06 | 1.46e-02 |
|  | 17 | 33760230 | 33760527 | PDFSTATSENSEMBLUCSC | 298 | 5 | 17237 | tss | 0.23982 | 0.77188 | Not queried | 4.06 | 7.94e-03 | 1.00e+00 |
|  | X | 49042873 | 49043355 | PDFSTATSENSEMBLUCSC | 483 | 6 | 41313 | tss;island | 0.31017 | 0.01775 | Not queried | 4.06 | 8.66e-03 | 1.00e+00 |
|  | 6 | 26103952 | 26104560 | PDFSTATSENSEMBLUCSC | 609 | 9 | 35451 | island | 0.03477 | 0.31535 | Not queried | 4.06 | 4.11e-05 | 2.11e-01 |
|  | Y | 22737391 | 22737663 | PDFSTATSENSEMBLUCSC | 273 | 7 | 42184 | island | 0.02270 | 0.30004 | Not queried | 4.05 | 5.83e-04 | 1.00e+00 |
|  | 7 | 27196296 | 27196555 | PDFSTATSENSEMBLUCSC | 260 | 7 | 37429 | tss | 0.03109 | 0.33114 | Not queried | 4.05 | 5.83e-04 | 1.00e+00 |
|  | 16 | 88832476 | 88832700 | PDFSTATSENSEMBLUCSC | 225 | 6 | 14887 | gene | 0.33568 | 0.90007 | Not queried | 4.05 | 2.16e-03 | 1.00e+00 |
|  | 11 | 1320329 | 1320634 | PDFSTATSENSEMBLUCSC | 306 | 4 | 6395 | gene;island | 0.36077 | 0.88477 | Not queried | 4.05 | 5.71e-02 | 1.00e+00 |
|  | 8 | 97657072 | 97657432 | PDFSTATSENSEMBLUCSC | 361 | 10 | 39764 | tss | 0.07953 | 0.54536 | Not queried | 4.05 | 1.08e-05 | 5.56e-02 |
|  | 11 | 124735089 | 124735227 | PDFSTATSENSEMBLUCSC | 139 | 7 | 7764 | tss | 0.06791 | 0.40594 | Not queried | 4.05 | 2.33e-03 | 1.00e+00 |
|  | 1 | 179050673 | 179051253 | PDFSTATSENSEMBLUCSC | 581 | 9 | 3980 | island | 0.04981 | 0.46360 | Not queried | 4.04 | 1.85e-03 | 1.00e+00 |
|  | 6 | 32119616 | 32119691 | PDFSTATSENSEMBLUCSC | 76 | 4 | 33997 | gene | 0.24126 | 0.83164 | Not queried | 4.04 | 2.86e-02 | 1.00e+00 |
|  | 7 | 25898206 | 25898770 | PDFSTATSENSEMBLUCSC | 565 | 7 | 38261 | island | 0.73052 | 0.12485 | Not queried | 4.04 | 5.83e-04 | 1.00e+00 |
|  | 19 | 35940425 | 35940593 | PDFSTATSENSEMBLUCSC | 169 | 4 | 20667 | tss | 0.20292 | 0.75996 | Not queried | 4.04 | 2.86e-02 | 1.00e+00 |
|  | 17 | 35300448 | 35300874 | PDFSTATSENSEMBLUCSC | 427 | 5 | 18205 | island | 0.70351 | 0.06500 | Not queried | 4.04 | 1.59e-02 | 1.00e+00 |
|  | 8 | 110987530 | 110988444 | PDFSTATSENSEMBLUCSC | 915 | 9 | 39811 | tss;island | 0.03210 | 0.27702 | Not queried | 4.04 | 3.99e-03 | 1.00e+00 |
|  | 19 | 12946231 | 12946465 | PDFSTATSENSEMBLUCSC | 235 | 6 | 20479 | tss | 0.03281 | 0.22419 | Not queried | 4.03 | 2.16e-03 | 1.00e+00 |
|  | 13 | 31506685 | 31506773 | PDFSTATSENSEMBLUCSC | 89 | 5 | 11349 | tss | 0.84188 | 0.22216 | Not queried | 4.03 | 7.94e-03 | 1.00e+00 |
|  | 12 | 99139301 | 99139867 | PDFSTATSENSEMBLUCSC | 567 | 6 | 9236 | gene;island | 0.11185 | 0.65121 | Not queried | 4.03 | 2.16e-03 | 1.00e+00 |
|  | 14 | 26674046 | 26674563 | PDFSTATSENSEMBLUCSC | 518 | 7 | 12725 | island | 0.03684 | 0.50617 | Not queried | 4.03 | 2.33e-03 | 1.00e+00 |
|  | X | 53461213 | 53461709 | PDFSTATSENSEMBLUCSC | 497 | 10 | 41809 | island | 0.03236 | 0.37159 | Not queried | 4.03 | 1.08e-05 | 5.56e-02 |
|  | 10 | 134901199 | 134901689 | PDFSTATSENSEMBLUCSC | 491 | 9 | 6313 | island | 0.02691 | 0.47961 | Not queried | 4.03 | 8.23e-05 | 4.23e-01 |
|  | 10 | 98144309 | 98144512 | PDFSTATSENSEMBLUCSC | 204 | 5 | 4676 | gene;island | 0.33779 | 0.89253 | Not queried | 4.03 | 1.59e-02 | 1.00e+00 |
|  | 7 | 33080496 | 33080615 | PDFSTATSENSEMBLUCSC | 120 | 4 | 36897 | gene | 0.31453 | 0.88060 | Not queried | 4.02 | 2.86e-02 | 1.00e+00 |
|  | 20 | 33146263 | 33146515 | PDFSTATSENSEMBLUCSC | 253 | 4 | 25654 | tss | 0.02560 | 0.29900 | Not queried | 4.02 | 2.86e-02 | 1.00e+00 |
|  | 12 | 51566379 | 51567112 | PDFSTATSENSEMBLUCSC | 734 | 11 | 10396 | island | 0.44877 | 0.04101 | TFCP2 (margin: RNU6-199P; TFCP2) | 4.02 | 2.84e-06 | 1.46e-02 |
|  | 2 | 96192273 | 96192699 | PDFSTATSENSEMBLUCSC | 427 | 4 | 24479 | island | 0.21181 | 0.74391 | Not queried | 4.02 | 2.86e-02 | 1.00e+00 |
|  | 19 | 38794554 | 38794845 | PDFSTATSENSEMBLUCSC | 292 | 6 | 19979 | gene | 0.21134 | 0.78404 | Not queried | 4.02 | 2.16e-03 | 1.00e+00 |
|  | 14 | 70316898 | 70317239 | PDFSTATSENSEMBLUCSC | 342 | 5 | 12944 | island | 0.28112 | 0.84244 | Not queried | 4.01 | 1.59e-02 | 1.00e+00 |
|  | 6 | 101329304 | 101329504 | PDFSTATSENSEMBLUCSC | 201 | 5 | 35078 | tss | 0.53212 | 0.05276 | Not queried | 4.01 | 7.94e-03 | 1.00e+00 |
|  | 17 | 48610072 | 48610255 | PDFSTATSENSEMBLUCSC | 184 | 4 | 16654 | gene | 0.03490 | 0.41122 | Not queried | 4.01 | 2.86e-02 | 1.00e+00 |
|  | 22 | 45072455 | 45072724 | PDFSTATSENSEMBLUCSC | 270 | 4 | 26851 | gene | 0.23751 | 0.78664 | Not queried | 4.00 | 2.86e-02 | 1.00e+00 |
|  | 4 | 147561669 | 147562073 | PDFSTATSENSEMBLUCSC | 405 | 4 | 30102 | gene;island | 0.22227 | 0.81826 | Not queried | 4.00 | 2.86e-02 | 1.00e+00 |
|  | 17 | 183931 | 184131 | PDFSTATSENSEMBLUCSC | 201 | 4 | 16255 | gene;island | 0.28787 | 0.85548 | Not queried | 4.00 | 2.86e-02 | 1.00e+00 |
|  | 16 | 2569911 | 2569967 | PDFSTATSENSEMBLUCSC | 57 | 4 | 14577 | gene;tss;island | 0.43139 | 0.92287 | Not queried | 4.00 | 1.14e-01 | 1.00e+00 |
|  | 22 | 24577142 | 24577438 | PDFSTATSENSEMBLUCSC | 297 | 6 | 26975 | tss | 0.04665 | 0.45386 | Not queried | 4.00 | 2.16e-03 | 1.00e+00 |
|  | 3 | 119421553 | 119421667 | PDFSTATSENSEMBLUCSC | 115 | 4 | 28483 | tss;island | 0.50545 | 0.06518 | Not queried | 4.00 | 1.14e-01 | 1.00e+00 |
|  | 7 | 75157456 | 75157765 | PDFSTATSENSEMBLUCSC | 310 | 7 | 37589 | tss | 0.32677 | 0.02228 | Not queried | 3.99 | 5.83e-04 | 1.00e+00 |
|  | 17 | 48046663 | 48046859 | PDFSTATSENSEMBLUCSC | 197 | 4 | 16641 | gene;island | 0.05096 | 0.34439 | Not queried | 3.99 | 2.86e-02 | 1.00e+00 |
|  | 6 | 151560831 | 151561236 | PDFSTATSENSEMBLUCSC | 406 | 9 | 36508 | island | 0.68217 | 0.10177 | Not queried | 3.99 | 4.11e-05 | 2.11e-01 |
|  | 1 | 29460817 | 29461070 | PDFSTATSENSEMBLUCSC | 254 | 4 | 2953 | island | 0.26976 | 0.81054 | Not queried | 3.99 | 2.86e-02 | 1.00e+00 |
|  | 3 | 52569026 | 52569169 | PDFSTATSENSEMBLUCSC | 144 | 5 | 28342 | tss;island | 0.06614 | 0.75540 | Not queried | 3.99 | 1.51e-01 | 1.00e+00 |
|  | 11 | 417540 | 417643 | PDFSTATSENSEMBLUCSC | 104 | 4 | 6991 | tss;island | 0.09664 | 0.61549 | Not queried | 3.99 | 2.86e-02 | 1.00e+00 |
|  | 9 | 95896749 | 95897015 | PDFSTATSENSEMBLUCSC | 267 | 4 | 40757 | tss;island | 0.45250 | 0.05099 | Not queried | 3.98 | 2.00e-01 | 1.00e+00 |
|  | 2 | 176956396 | 176956841 | PDFSTATSENSEMBLUCSC | 446 | 5 | 23694 | tss;island | 0.78350 | 0.24391 | Not queried | 3.98 | 7.94e-03 | 1.00e+00 |
|  | 4 | 1166755 | 1166990 | PDFSTATSENSEMBLUCSC | 236 | 8 | 29814 | gene;tss;island | 0.28190 | 0.86806 | Not queried | 3.98 | 1.55e-04 | 7.99e-01 |
|  | 11 | 63775071 | 63775612 | PDFSTATSENSEMBLUCSC | 542 | 5 | 6640 | gene;island | 0.30583 | 0.77957 | Not queried | 3.98 | 7.94e-03 | 1.00e+00 |
|  | 3 | 53529144 | 53529655 | PDFSTATSENSEMBLUCSC | 512 | 5 | 27787 | gene;island | 0.25751 | 0.03107 | Not queried | 3.98 | 1.59e-02 | 1.00e+00 |
|  | 17 | 8702698 | 8702896 | PDFSTATSENSEMBLUCSC | 199 | 6 | 17089 | tss | 0.12502 | 0.65645 | Not queried | 3.97 | 2.16e-03 | 1.00e+00 |
|  | 16 | 57125654 | 57125836 | PDFSTATSENSEMBLUCSC | 183 | 5 | 15226 | tss;island | 0.17331 | 0.02699 | Not queried | 3.97 | 1.59e-02 | 1.00e+00 |
|  | 8 | 144654887 | 144655030 | PDFSTATSENSEMBLUCSC | 144 | 4 | 40607 | island | 0.23692 | 0.73239 | Not queried | 3.97 | 2.86e-02 | 1.00e+00 |
|  | 5 | 147443067 | 147443347 | PDFSTATSENSEMBLUCSC | 281 | 4 | 32351 | tss | 0.44469 | 0.93019 | Not queried | 3.97 | 2.86e-02 | 1.00e+00 |
|  | 11 | 124735089 | 124735554 | PDFSTATSENSEMBLUCSC | 466 | 10 | 8893 | island | 0.03569 | 0.40253 | Not queried | 3.97 | 1.30e-04 | 6.68e-01 |
|  | 4 | 41880987 | 41881417 | PDFSTATSENSEMBLUCSC | 431 | 4 | 30957 | island | 0.92186 | 0.28024 | Not queried | 3.97 | 2.86e-02 | 1.00e+00 |
|  | 7 | 50861418 | 50862266 | PDFSTATSENSEMBLUCSC | 849 | 20 | 37533 | tss;island | 0.30910 | 0.04255 | Not queried | 3.97 | 2.49e-05 | 1.28e-01 |
|  | 14 | 37051863 | 37051949 | PDFSTATSENSEMBLUCSC | 87 | 4 | 12331 | tss | 0.04162 | 0.55101 | Not queried | 3.97 | 2.86e-02 | 1.00e+00 |
|  | 19 | 35531639 | 35532097 | PDFSTATSENSEMBLUCSC | 459 | 5 | 19944 | gene;island | 0.27227 | 0.73666 | Not queried | 3.97 | 1.51e-01 | 1.00e+00 |
|  | 8 | 145018010 | 145018300 | PDFSTATSENSEMBLUCSC | 291 | 5 | 39436 | gene;tss;island | 0.04283 | 0.41171 | Not queried | 3.97 | 7.94e-03 | 1.00e+00 |
|  | 5 | 140551968 | 140552163 | PDFSTATSENSEMBLUCSC | 196 | 4 | 32305 | tss | 0.04270 | 0.17673 | Not queried | 3.97 | 1.14e-01 | 1.00e+00 |
|  | 4 | 1046821 | 1047097 | PDFSTATSENSEMBLUCSC | 277 | 4 | 30716 | island | 0.23812 | 0.87410 | Not queried | 3.96 | 2.86e-02 | 1.00e+00 |
|  | 13 | 21277342 | 21277505 | PDFSTATSENSEMBLUCSC | 164 | 4 | 11551 | island | 0.36389 | 0.03464 | Not queried | 3.96 | 2.86e-02 | 1.00e+00 |
|  | 4 | 62382932 | 62383240 | PDFSTATSENSEMBLUCSC | 309 | 4 | 29967 | gene;island | 0.03297 | 0.36076 | Not queried | 3.96 | 2.86e-02 | 1.00e+00 |
|  | 10 | 37414506 | 37414768 | PDFSTATSENSEMBLUCSC | 263 | 6 | 5014 | tss | 0.25712 | 0.88236 | Not queried | 3.96 | 2.16e-03 | 1.00e+00 |
|  | X | 49126087 | 49126243 | PDFSTATSENSEMBLUCSC | 157 | 8 | 41316 | tss;island | 0.20982 | 0.75873 | Not queried | 3.96 | 1.55e-04 | 7.99e-01 |
|  | 6 | 29893926 | 29894341 | PDFSTATSENSEMBLUCSC | 416 | 16 | 34693 | tss;island | 0.80704 | 0.31106 | Not queried | 3.96 | 2.29e-04 | 1.00e+00 |
|  | 3 | 126853940 | 126854423 | PDFSTATSENSEMBLUCSC | 484 | 4 | 29398 | island | 0.25881 | 0.91258 | Not queried | 3.96 | 5.71e-02 | 1.00e+00 |
|  | 17 | 76128481 | 76128683 | PDFSTATSENSEMBLUCSC | 203 | 8 | 16779 | gene;island | 0.12202 | 0.70566 | Not queried | 3.96 | 1.55e-04 | 7.99e-01 |
|  | 10 | 6020330 | 6020585 | PDFSTATSENSEMBLUCSC | 256 | 4 | 4914 | tss;island | 0.76535 | 0.09201 | Not queried | 3.96 | 1.14e-01 | 1.00e+00 |
|  | 12 | 114846313 | 114846503 | PDFSTATSENSEMBLUCSC | 191 | 7 | 10008 | tss | 0.94564 | 0.47037 | Not queried | 3.95 | 5.83e-04 | 1.00e+00 |
|  | 2 | 220378996 | 220379260 | PDFSTATSENSEMBLUCSC | 265 | 9 | 22999 | gene;island | 0.19029 | 0.68151 | Not queried | 3.95 | 1.65e-04 | 8.46e-01 |
|  | 12 | 54378736 | 54378896 | PDFSTATSENSEMBLUCSC | 161 | 7 | 9725 | tss | 0.01530 | 0.21773 | Not queried | 3.95 | 1.17e-03 | 1.00e+00 |
|  | 3 | 192445388 | 192445594 | PDFSTATSENSEMBLUCSC | 207 | 7 | 28749 | tss | 0.01359 | 0.23222 | Not queried | 3.95 | 5.83e-04 | 1.00e+00 |
|  | 17 | 45772333 | 45772800 | PDFSTATSENSEMBLUCSC | 468 | 7 | 18423 | island | 0.34597 | 0.02909 | Not queried | 3.95 | 5.83e-04 | 1.00e+00 |
|  | 2 | 171568984 | 171569381 | PDFSTATSENSEMBLUCSC | 398 | 6 | 22880 | gene | 0.76761 | 0.15175 | Not queried | 3.95 | 4.11e-02 | 1.00e+00 |
|  | 1 | 51810337 | 51811185 | PDFSTATSENSEMBLUCSC | 849 | 8 | 3254 | island | 0.15299 | 0.65973 | Not queried | 3.95 | 6.50e-02 | 1.00e+00 |
|  | 15 | 37110774 | 37110854 | PDFSTATSENSEMBLUCSC | 81 | 4 | 13539 | tss | 0.57473 | 0.96315 | Not queried | 3.95 | 2.86e-02 | 1.00e+00 |
|  | 1 | 229567914 | 229568467 | PDFSTATSENSEMBLUCSC | 554 | 5 | 1061 | gene;island | 0.20526 | 0.86072 | Not queried | 3.94 | 3.17e-02 | 1.00e+00 |
|  | 12 | 54409207 | 54409770 | PDFSTATSENSEMBLUCSC | 564 | 12 | 9728 | tss;island | 0.91803 | 0.42467 | Not queried | 3.94 | 4.96e-05 | 2.55e-01 |
|  | 4 | 41869086 | 41869611 | PDFSTATSENSEMBLUCSC | 526 | 7 | 30955 | island | 0.28725 | 0.04277 | Not queried | 3.94 | 4.08e-03 | 1.00e+00 |
|  | 10 | 105037349 | 105037796 | PDFSTATSENSEMBLUCSC | 448 | 4 | 4725 | gene;island | 0.01495 | 0.28714 | Not queried | 3.94 | 2.86e-02 | 1.00e+00 |
|  | 7 | 24797102 | 24797884 | PDFSTATSENSEMBLUCSC | 783 | 11 | 37413 | tss | 0.51331 | 0.03719 | Not queried | 3.94 | 1.92e-02 | 1.00e+00 |
|  | 3 | 129407365 | 129407548 | PDFSTATSENSEMBLUCSC | 184 | 5 | 27901 | gene | 0.39670 | 0.04416 | Not queried | 3.93 | 7.94e-03 | 1.00e+00 |
|  | 4 | 5710319 | 5710411 | PDFSTATSENSEMBLUCSC | 93 | 6 | 29876 | gene;tss;island | 0.35200 | 0.90102 | Not queried | 3.93 | 2.16e-03 | 1.00e+00 |
|  | 16 | 58533743 | 58534157 | PDFSTATSENSEMBLUCSC | 415 | 4 | 14743 | gene;island | 0.18507 | 0.77330 | Not queried | 3.93 | 2.86e-02 | 1.00e+00 |
|  | 22 | 42896105 | 42896573 | PDFSTATSENSEMBLUCSC | 469 | 6 | 27149 | tss | 0.10396 | 0.71699 | Not queried | 3.93 | 2.16e-03 | 1.00e+00 |
|  | 12 | 114846162 | 114846503 | PDFSTATSENSEMBLUCSC | 342 | 8 | 10813 | island | 0.93782 | 0.45921 | Not queried | 3.93 | 1.55e-04 | 7.99e-01 |
|  | 8 | 22926785 | 22926911 | PDFSTATSENSEMBLUCSC | 127 | 7 | 39532 | tss | 0.43370 | 0.03110 | Not queried | 3.93 | 5.83e-04 | 1.00e+00 |
|  | 2 | 200524002 | 200524259 | PDFSTATSENSEMBLUCSC | 258 | 4 | 24956 | island | 0.14507 | 0.69823 | Not queried | 3.93 | 2.86e-02 | 1.00e+00 |
|  | 12 | 91573135 | 91573372 | PDFSTATSENSEMBLUCSC | 238 | 4 | 9217 | gene;tss | 0.15619 | 0.73476 | Not queried | 3.93 | 1.14e-01 | 1.00e+00 |
|  | 15 | 68724690 | 68725002 | PDFSTATSENSEMBLUCSC | 313 | 4 | 13731 | tss;island | 0.75425 | 0.21941 | Not queried | 3.92 | 1.14e-01 | 1.00e+00 |
|  | 6 | 26104185 | 26104560 | PDFSTATSENSEMBLUCSC | 376 | 4 | 33537 | gene | 0.03324 | 0.30436 | Not queried | 3.92 | 2.86e-02 | 1.00e+00 |
|  | 3 | 138657291 | 138657774 | PDFSTATSENSEMBLUCSC | 484 | 5 | 29485 | island | 0.61953 | 0.15497 | Not queried | 3.92 | 7.94e-03 | 1.00e+00 |
|  | 7 | 1126249 | 1126903 | PDFSTATSENSEMBLUCSC | 655 | 8 | 36686 | gene;tss;island | 0.04461 | 0.50771 | Not queried | 3.92 | 2.07e-02 | 1.00e+00 |
|  | 7 | 34118464 | 34118935 | PDFSTATSENSEMBLUCSC | 472 | 5 | 36898 | gene;island | 0.27258 | 0.85040 | Not queried | 3.92 | 7.94e-03 | 1.00e+00 |
|  | 1 | 47695138 | 47695475 | PDFSTATSENSEMBLUCSC | 338 | 4 | 3223 | island | 0.69923 | 0.11959 | Not queried | 3.92 | 2.86e-02 | 1.00e+00 |
|  | 12 | 85673200 | 85673347 | PDFSTATSENSEMBLUCSC | 148 | 4 | 9875 | tss;island | 0.49366 | 0.04198 | Not queried | 3.92 | 5.71e-02 | 1.00e+00 |
|  | 7 | 4869981 | 4870162 | PDFSTATSENSEMBLUCSC | 182 | 4 | 36796 | gene | 0.19025 | 0.84394 | Not queried | 3.92 | 5.71e-02 | 1.00e+00 |
|  | 2 | 85811292 | 85811662 | PDFSTATSENSEMBLUCSC | 371 | 5 | 24444 | island | 0.82066 | 0.23241 | Not queried | 3.92 | 7.94e-03 | 1.00e+00 |
|  | 12 | 52300911 | 52301163 | PDFSTATSENSEMBLUCSC | 253 | 4 | 9696 | tss | 0.10741 | 0.64187 | Not queried | 3.92 | 2.86e-02 | 1.00e+00 |
|  | 1 | 175568216 | 175568710 | PDFSTATSENSEMBLUCSC | 495 | 4 | 893 | gene;island | 0.38980 | 0.88981 | Not queried | 3.92 | 2.86e-02 | 1.00e+00 |
|  | 4 | 118006405 | 118006832 | PDFSTATSENSEMBLUCSC | 428 | 9 | 31210 | island | 0.02384 | 0.34489 | Not queried | 3.91 | 4.11e-05 | 2.11e-01 |
|  | X | 53461386 | 53461709 | PDFSTATSENSEMBLUCSC | 324 | 8 | 41331 | tss | 0.04037 | 0.37159 | Not queried | 3.91 | 1.55e-04 | 7.99e-01 |
|  | 11 | 27015656 | 27015991 | PDFSTATSENSEMBLUCSC | 336 | 5 | 6523 | gene | 0.13558 | 0.71504 | Not queried | 3.91 | 7.94e-03 | 1.00e+00 |
|  | 5 | 180486604 | 180486879 | PDFSTATSENSEMBLUCSC | 276 | 4 | 31890 | gene;island | 0.07288 | 0.51942 | Not queried | 3.91 | 2.86e-02 | 1.00e+00 |
|  | 5 | 139080952 | 139081136 | PDFSTATSENSEMBLUCSC | 185 | 4 | 33115 | island | 0.41472 | 0.06119 | Not queried | 3.91 | 2.86e-02 | 1.00e+00 |
|  | 19 | 52552120 | 52552461 | PDFSTATSENSEMBLUCSC | 342 | 9 | 20969 | tss | 0.09424 | 0.58127 | Not queried | 3.91 | 4.11e-05 | 2.11e-01 |
|  | 10 | 104613767 | 104613934 | PDFSTATSENSEMBLUCSC | 168 | 6 | 5314 | tss | 0.58593 | 0.03881 | Not queried | 3.90 | 1.80e-01 | 1.00e+00 |
|  | 4 | 683060 | 683240 | PDFSTATSENSEMBLUCSC | 181 | 4 | 30182 | tss;island | 0.35882 | 0.85727 | Not queried | 3.90 | 2.86e-02 | 1.00e+00 |
|  | 6 | 108492642 | 108492769 | PDFSTATSENSEMBLUCSC | 128 | 4 | 34374 | gene;island | 0.64773 | 0.18973 | Not queried | 3.90 | 2.86e-02 | 1.00e+00 |
|  | 5 | 122429966 | 122430821 | PDFSTATSENSEMBLUCSC | 856 | 10 | 31673 | gene;island | 0.50959 | 0.07284 | Not queried | 3.90 | 2.17e-05 | 1.11e-01 |
|  | 17 | 80797881 | 80798078 | PDFSTATSENSEMBLUCSC | 198 | 8 | 16926 | gene | 0.39354 | 0.91585 | Not queried | 3.90 | 1.55e-04 | 7.99e-01 |
|  | 6 | 36355343 | 36355825 | PDFSTATSENSEMBLUCSC | 483 | 7 | 36019 | island | 0.08891 | 0.72764 | Not queried | 3.90 | 5.83e-04 | 1.00e+00 |
|  | 7 | 27281216 | 27281964 | PDFSTATSENSEMBLUCSC | 749 | 14 | 37435 | tss;island | 0.58450 | 0.13816 | Not queried | 3.90 | 2.74e-02 | 1.00e+00 |
|  | 7 | 24796981 | 24797884 | PDFSTATSENSEMBLUCSC | 904 | 12 | 38254 | island | 0.38740 | 0.03597 | Not queried | 3.89 | 2.84e-02 | 1.00e+00 |
|  | 3 | 49395574 | 49395745 | PDFSTATSENSEMBLUCSC | 172 | 4 | 27739 | gene | 0.89485 | 0.34741 | Not queried | 3.89 | 2.86e-02 | 1.00e+00 |
|  | 6 | 31165567 | 31166174 | PDFSTATSENSEMBLUCSC | 608 | 20 | 33800 | gene | 0.02592 | 0.28245 | HCG27; XXbac-BPG299F13.17; XXbac-BPG299F13.14 (margin: HCG27; XXbac-BPG299F13.17; XXbac-BPG299F13.14) | 3.89 | 1.69e-07 | 8.68e-04 |
|  | 4 | 4387697 | 4387917 | PDFSTATSENSEMBLUCSC | 221 | 6 | 30220 | tss;island | 0.33197 | 0.02104 | Not queried | 3.89 | 6.49e-02 | 1.00e+00 |
|  | 7 | 1269693 | 1270134 | PDFSTATSENSEMBLUCSC | 442 | 4 | 38026 | island | 0.04620 | 0.36399 | Not queried | 3.88 | 2.86e-02 | 1.00e+00 |
|  | X | 132547142 | 132547691 | PDFSTATSENSEMBLUCSC | 550 | 5 | 41109 | gene;island | 0.39833 | 0.03016 | Not queried | 3.88 | 1.51e-01 | 1.00e+00 |
|  | 1 | 156182601 | 156182774 | PDFSTATSENSEMBLUCSC | 174 | 4 | 2006 | tss | 0.42097 | 0.04086 | Not queried | 3.88 | 2.86e-02 | 1.00e+00 |
|  | X | 82763219 | 82763266 | PDFSTATSENSEMBLUCSC | 48 | 5 | 41406 | tss | 0.34870 | 0.92921 | Not queried | 3.88 | 7.94e-03 | 1.00e+00 |
|  | 6 | 30848807 | 30848846 | PDFSTATSENSEMBLUCSC | 40 | 4 | 35668 | island | 0.79741 | 0.24249 | Not queried | 3.88 | 2.86e-02 | 1.00e+00 |
|  | 1 | 8271918 | 8272277 | PDFSTATSENSEMBLUCSC | 360 | 5 | 2629 | island | 0.20812 | 0.77899 | Not queried | 3.88 | 7.94e-03 | 1.00e+00 |
|  | 2 | 202483704 | 202484320 | PDFSTATSENSEMBLUCSC | 617 | 9 | 24972 | island | 0.27023 | 0.75519 | Not queried | 3.88 | 1.85e-03 | 1.00e+00 |
|  | 5 | 42756786 | 42756876 | PDFSTATSENSEMBLUCSC | 91 | 4 | 31982 | tss | 0.91806 | 0.37091 | Not queried | 3.87 | 1.14e-01 | 1.00e+00 |
|  | 1 | 235813839 | 235814462 | PDFSTATSENSEMBLUCSC | 624 | 11 | 2332 | tss;island | 0.46118 | 0.04185 | Not queried | 3.87 | 5.39e-05 | 2.77e-01 |
|  | 4 | 1762881 | 1763211 | PDFSTATSENSEMBLUCSC | 331 | 4 | 30749 | island | 0.07876 | 0.69043 | Not queried | 3.87 | 1.14e-01 | 1.00e+00 |
|  | 6 | 35744188 | 35744355 | PDFSTATSENSEMBLUCSC | 168 | 4 | 34867 | tss;island | 0.29183 | 0.03591 | Not queried | 3.87 | 2.00e-01 | 1.00e+00 |
|  | X | 138276904 | 138277085 | PDFSTATSENSEMBLUCSC | 182 | 4 | 41125 | gene;island | 0.01957 | 0.24732 | Not queried | 3.87 | 2.86e-02 | 1.00e+00 |
|  | 5 | 72740460 | 72740748 | PDFSTATSENSEMBLUCSC | 289 | 5 | 32810 | island | 0.74444 | 0.20036 | Not queried | 3.87 | 3.17e-02 | 1.00e+00 |
|  | 2 | 21022431 | 21023042 | PDFSTATSENSEMBLUCSC | 612 | 8 | 24079 | island | 0.92281 | 0.49336 | Not queried | 3.87 | 1.55e-04 | 7.99e-01 |
|  | 2 | 21022431 | 21022699 | PDFSTATSENSEMBLUCSC | 269 | 4 | 22555 | gene | 0.92281 | 0.49336 | Not queried | 3.87 | 2.86e-02 | 1.00e+00 |
|  | 3 | 157813327 | 157813810 | PDFSTATSENSEMBLUCSC | 484 | 6 | 29572 | island | 0.75843 | 0.17305 | Not queried | 3.87 | 4.33e-03 | 1.00e+00 |
|  | 10 | 11783794 | 11784473 | PDFSTATSENSEMBLUCSC | 680 | 10 | 5528 | island | 0.09289 | 0.63106 | Not queried | 3.87 | 1.05e-03 | 1.00e+00 |
|  | 19 | 46318633 | 46318918 | PDFSTATSENSEMBLUCSC | 286 | 4 | 20845 | tss | 0.32216 | 0.04190 | Not queried | 3.87 | 2.86e-02 | 1.00e+00 |
|  | 3 | 46742865 | 46743199 | PDFSTATSENSEMBLUCSC | 335 | 4 | 27712 | gene | 0.32310 | 0.85802 | Not queried | 3.87 | 2.86e-02 | 1.00e+00 |
|  | 10 | 129535138 | 129535509 | PDFSTATSENSEMBLUCSC | 372 | 6 | 5418 | tss | 0.05016 | 0.44190 | Not queried | 3.87 | 2.16e-03 | 1.00e+00 |
|  | 19 | 35068555 | 35068788 | PDFSTATSENSEMBLUCSC | 234 | 4 | 21853 | island | 0.23221 | 0.82786 | Not queried | 3.87 | 2.86e-02 | 1.00e+00 |
|  | 12 | 103311088 | 103311761 | PDFSTATSENSEMBLUCSC | 674 | 9 | 10699 | island | 0.02814 | 0.34054 | Not queried | 3.86 | 1.85e-03 | 1.00e+00 |
|  | 6 | 31165290 | 31166174 | PDFSTATSENSEMBLUCSC | 885 | 29 | 35694 | island | 0.02763 | 0.27578 | HCG27; XXbac-BPG299F13.17; XXbac-BPG299F13.14 (margin: HCG27; XXbac-BPG299F13.17; XXbac-BPG299F13.14) | 3.86 | 1.43e-11 | 7.33e-08 |
|  | 1 | 32741454 | 32741619 | PDFSTATSENSEMBLUCSC | 166 | 4 | 392 | gene;island | 0.34995 | 0.81320 | Not queried | 3.86 | 2.86e-02 | 1.00e+00 |
|  | 17 | 57915665 | 57915773 | PDFSTATSENSEMBLUCSC | 109 | 4 | 16680 | gene | 0.03018 | 0.38278 | Not queried | 3.86 | 2.86e-02 | 1.00e+00 |
|  | 6 | 27840957 | 27841423 | PDFSTATSENSEMBLUCSC | 467 | 11 | 34664 | tss;island | 0.45763 | 0.93338 | Not queried | 3.86 | 1.98e-05 | 1.02e-01 |
|  | X | 135578793 | 135579224 | PDFSTATSENSEMBLUCSC | 432 | 8 | 41549 | tss;island | 0.04097 | 0.52580 | Not queried | 3.85 | 1.55e-04 | 7.99e-01 |
|  | 5 | 72598865 | 72599228 | PDFSTATSENSEMBLUCSC | 364 | 4 | 32808 | island | 0.05872 | 0.48406 | Not queried | 3.85 | 2.00e-01 | 1.00e+00 |
|  | 7 | 157294324 | 157294502 | PDFSTATSENSEMBLUCSC | 179 | 4 | 39043 | island | 0.14360 | 0.76987 | Not queried | 3.85 | 2.86e-02 | 1.00e+00 |
|  | 22 | 50329860 | 50330022 | PDFSTATSENSEMBLUCSC | 163 | 4 | 27566 | island | 0.03747 | 0.38346 | Not queried | 3.84 | 2.86e-02 | 1.00e+00 |
|  | X | 2984578 | 2984799 | PDFSTATSENSEMBLUCSC | 222 | 5 | 41170 | tss | 0.04540 | 0.42273 | Not queried | 3.84 | 7.94e-03 | 1.00e+00 |
|  | 22 | 18632404 | 18632618 | PDFSTATSENSEMBLUCSC | 215 | 5 | 26908 | tss | 0.02771 | 0.33411 | Not queried | 3.84 | 5.56e-02 | 1.00e+00 |
|  | 19 | 12707809 | 12708001 | PDFSTATSENSEMBLUCSC | 193 | 4 | 19820 | gene;island | 0.94764 | 0.56483 | Not queried | 3.84 | 2.86e-02 | 1.00e+00 |
|  | 3 | 126113327 | 126114064 | PDFSTATSENSEMBLUCSC | 738 | 13 | 29389 | island | 0.29859 | 0.86290 | Not queried | 3.83 | 7.95e-04 | 1.00e+00 |
|  | 12 | 91347805 | 91348242 | PDFSTATSENSEMBLUCSC | 438 | 4 | 9215 | gene;island | 0.62744 | 0.86892 | Not queried | 3.83 | 3.43e-01 | 1.00e+00 |
|  | 19 | 17392656 | 17392923 | PDFSTATSENSEMBLUCSC | 268 | 4 | 19880 | gene;island | 0.30166 | 0.03515 | Not queried | 3.83 | 2.86e-02 | 1.00e+00 |
|  | 2 | 231712722 | 231712981 | PDFSTATSENSEMBLUCSC | 260 | 5 | 25137 | island | 0.73365 | 0.16275 | Not queried | 3.82 | 9.52e-02 | 1.00e+00 |
|  | 5 | 851101 | 851458 | PDFSTATSENSEMBLUCSC | 358 | 6 | 31903 | tss;island | 0.20964 | 0.75987 | Not queried | 3.82 | 2.16e-03 | 1.00e+00 |
|  | 16 | 3017902 | 3018262 | PDFSTATSENSEMBLUCSC | 361 | 4 | 14589 | gene;tss;island | 0.06632 | 0.56673 | Not queried | 3.82 | 2.86e-02 | 1.00e+00 |
|  | 3 | 46037408 | 46037608 | PDFSTATSENSEMBLUCSC | 201 | 6 | 28231 | tss;island | 0.39792 | 0.05926 | Not queried | 3.82 | 8.66e-03 | 1.00e+00 |
|  | 2 | 223916502 | 223916952 | PDFSTATSENSEMBLUCSC | 451 | 7 | 25119 | island | 0.45487 | 0.93262 | Not queried | 3.82 | 5.83e-04 | 1.00e+00 |
|  | X | 48815643 | 48815856 | PDFSTATSENSEMBLUCSC | 214 | 7 | 41303 | tss;island | 0.24686 | 0.01270 | Not queried | 3.82 | 4.08e-03 | 1.00e+00 |
|  | 8 | 1365049 | 1365906 | PDFSTATSENSEMBLUCSC | 858 | 7 | 39960 | island | 0.32627 | 0.87228 | Not queried | 3.82 | 6.99e-03 | 1.00e+00 |
|  | 10 | 46993502 | 46993586 | PDFSTATSENSEMBLUCSC | 85 | 4 | 5700 | island | 0.48900 | 0.06646 | Not queried | 3.82 | 2.86e-02 | 1.00e+00 |
|  | 11 | 69706831 | 69706981 | PDFSTATSENSEMBLUCSC | 151 | 4 | 8578 | island | 0.11461 | 0.64538 | Not queried | 3.81 | 2.86e-02 | 1.00e+00 |
|  | 6 | 159290838 | 159291228 | PDFSTATSENSEMBLUCSC | 391 | 4 | 36539 | island | 0.46801 | 0.92818 | Not queried | 3.81 | 2.86e-02 | 1.00e+00 |
|  | 17 | 73583839 | 73584111 | PDFSTATSENSEMBLUCSC | 273 | 8 | 17623 | tss;island | 0.52697 | 0.09306 | Not queried | 3.81 | 1.55e-04 | 7.99e-01 |
|  | 11 | 87908631 | 87908938 | PDFSTATSENSEMBLUCSC | 308 | 8 | 7591 | tss | 0.24226 | 0.03201 | Not queried | 3.81 | 4.66e-03 | 1.00e+00 |
|  | 17 | 43506728 | 43507153 | PDFSTATSENSEMBLUCSC | 426 | 4 | 16600 | gene | 0.44085 | 0.07123 | Not queried | 3.81 | 5.71e-02 | 1.00e+00 |
|  | X | 82763219 | 82763706 | PDFSTATSENSEMBLUCSC | 488 | 8 | 41892 | island | 0.36379 | 0.90835 | Not queried | 3.81 | 1.55e-04 | 7.99e-01 |
|  | 1 | 159750305 | 159750570 | PDFSTATSENSEMBLUCSC | 266 | 5 | 2026 | tss;island | 0.01292 | 0.11528 | Not queried | 3.80 | 3.17e-02 | 1.00e+00 |
|  | 11 | 87908558 | 87908938 | PDFSTATSENSEMBLUCSC | 381 | 9 | 8695 | island | 0.20440 | 0.03294 | Not queried | 3.80 | 3.99e-03 | 1.00e+00 |
|  | 5 | 170740547 | 170740937 | PDFSTATSENSEMBLUCSC | 391 | 6 | 33316 | island | 0.94813 | 0.47140 | Not queried | 3.80 | 2.16e-03 | 1.00e+00 |
|  | 2 | 27346296 | 27346593 | PDFSTATSENSEMBLUCSC | 298 | 5 | 23217 | tss | 0.07527 | 0.53128 | Not queried | 3.80 | 1.51e-01 | 1.00e+00 |
|  | 2 | 27346296 | 27346938 | PDFSTATSENSEMBLUCSC | 643 | 7 | 24131 | island | 0.07527 | 0.51047 | Not queried | 3.80 | 2.62e-02 | 1.00e+00 |
|  | X | 46404528 | 46405125 | PDFSTATSENSEMBLUCSC | 598 | 14 | 41271 | tss;island | 0.08432 | 0.59461 | ZNF674-AS1; ZNF674 (margin: ZNF674-AS1; ZNF674) | 3.80 | 4.99e-08 | 2.56e-04 |
|  | 3 | 142839837 | 142840240 | PDFSTATSENSEMBLUCSC | 404 | 5 | 27938 | gene;island | 0.05005 | 0.45878 | Not queried | 3.80 | 7.94e-03 | 1.00e+00 |
|  | 2 | 132152167 | 132152543 | PDFSTATSENSEMBLUCSC | 377 | 4 | 24692 | island | 0.06867 | 0.47574 | Not queried | 3.80 | 2.86e-02 | 1.00e+00 |
|  | 4 | 155413392 | 155413789 | PDFSTATSENSEMBLUCSC | 398 | 4 | 30596 | tss;island | 0.47909 | 0.06723 | Not queried | 3.80 | 1.14e-01 | 1.00e+00 |
|  | 10 | 93805441 | 93805870 | PDFSTATSENSEMBLUCSC | 430 | 5 | 5917 | island | 0.25707 | 0.78654 | Not queried | 3.80 | 7.94e-03 | 1.00e+00 |
|  | 11 | 63382047 | 63382095 | PDFSTATSENSEMBLUCSC | 49 | 5 | 7355 | tss;island | 0.12710 | 0.01039 | Not queried | 3.79 | 7.94e-03 | 1.00e+00 |
|  | 3 | 49066976 | 49067151 | PDFSTATSENSEMBLUCSC | 176 | 7 | 28274 | tss | 0.03096 | 0.31761 | Not queried | 3.79 | 5.83e-04 | 1.00e+00 |
|  | 19 | 40324850 | 40325100 | PDFSTATSENSEMBLUCSC | 251 | 7 | 20742 | tss | 0.04368 | 0.44299 | Not queried | 3.79 | 1.75e-02 | 1.00e+00 |
|  | 1 | 84326462 | 84326856 | PDFSTATSENSEMBLUCSC | 395 | 8 | 3399 | island | 0.03716 | 0.35114 | Not queried | 3.79 | 1.55e-04 | 7.99e-01 |
|  | 1 | 2324205 | 2324396 | PDFSTATSENSEMBLUCSC | 192 | 4 | 81 | gene;tss;island | 0.31422 | 0.81457 | Not queried | 3.79 | 2.86e-02 | 1.00e+00 |
|  | 8 | 99961768 | 99962118 | PDFSTATSENSEMBLUCSC | 351 | 4 | 39312 | gene;island | 0.87490 | 0.25212 | Not queried | 3.79 | 2.86e-02 | 1.00e+00 |
|  | 10 | 94180383 | 94180913 | PDFSTATSENSEMBLUCSC | 531 | 5 | 5921 | island | 0.45816 | 0.06552 | Not queried | 3.78 | 9.52e-02 | 1.00e+00 |
|  | 17 | 80797948 | 80798078 | PDFSTATSENSEMBLUCSC | 131 | 7 | 17748 | tss | 0.37842 | 0.90492 | Not queried | 3.78 | 5.83e-04 | 1.00e+00 |
|  | 7 | 158243369 | 158243730 | PDFSTATSENSEMBLUCSC | 362 | 4 | 37308 | gene | 0.47369 | 0.89030 | Not queried | 3.78 | 2.86e-02 | 1.00e+00 |
|  | 16 | 46918956 | 46919194 | PDFSTATSENSEMBLUCSC | 239 | 5 | 14712 | gene;island | 0.33213 | 0.04392 | Not queried | 3.78 | 5.56e-02 | 1.00e+00 |
|  | 22 | 18267969 | 18268249 | PDFSTATSENSEMBLUCSC | 281 | 4 | 27236 | island | 0.22822 | 0.82955 | Not queried | 3.78 | 2.86e-02 | 1.00e+00 |
|  | 2 | 175208588 | 175208761 | PDFSTATSENSEMBLUCSC | 174 | 4 | 24838 | island | 0.32546 | 0.03351 | Not queried | 3.78 | 2.86e-02 | 1.00e+00 |
|  | 3 | 49395574 | 49395991 | PDFSTATSENSEMBLUCSC | 418 | 8 | 29073 | island | 0.86272 | 0.34741 | Not queried | 3.78 | 1.55e-04 | 7.99e-01 |
|  | 6 | 88877064 | 88877413 | PDFSTATSENSEMBLUCSC | 350 | 4 | 36254 | island | 0.35620 | 0.04668 | Not queried | 3.78 | 2.86e-02 | 1.00e+00 |
|  | 5 | 115697214 | 115697696 | PDFSTATSENSEMBLUCSC | 483 | 5 | 32969 | island | 0.36984 | 0.76857 | Not queried | 3.78 | 7.94e-03 | 1.00e+00 |
|  | 7 | 158854447 | 158854856 | PDFSTATSENSEMBLUCSC | 410 | 4 | 37318 | gene;island | 0.48009 | 0.96095 | Not queried | 3.78 | 4.86e-01 | 1.00e+00 |
|  | 4 | 10686394 | 10686610 | PDFSTATSENSEMBLUCSC | 217 | 4 | 30254 | tss | 0.42512 | 0.91380 | Not queried | 3.77 | 2.86e-02 | 1.00e+00 |
|  | 10 | 1416546 | 1416920 | PDFSTATSENSEMBLUCSC | 375 | 6 | 4449 | gene;island | 0.26774 | 0.76757 | Not queried | 3.77 | 2.16e-03 | 1.00e+00 |
|  | 14 | 77737661 | 77737800 | PDFSTATSENSEMBLUCSC | 140 | 4 | 12501 | tss | 0.26696 | 0.84202 | Not queried | 3.77 | 2.86e-02 | 1.00e+00 |
|  | 12 | 132514266 | 132514508 | PDFSTATSENSEMBLUCSC | 243 | 6 | 9403 | gene;island | 0.42861 | 0.90694 | Not queried | 3.77 | 2.16e-03 | 1.00e+00 |
|  | 16 | 15489542 | 15489968 | PDFSTATSENSEMBLUCSC | 427 | 6 | 15694 | island | 0.03307 | 0.35429 | Not queried | 3.77 | 4.11e-02 | 1.00e+00 |
|  | 22 | 19743759 | 19744239 | PDFSTATSENSEMBLUCSC | 481 | 8 | 27262 | island | 0.24666 | 0.77682 | Not queried | 3.77 | 1.55e-04 | 7.99e-01 |
|  | 17 | 45569959 | 45570168 | PDFSTATSENSEMBLUCSC | 210 | 4 | 18421 | island | 0.01419 | 0.51074 | Not queried | 3.76 | 5.71e-02 | 1.00e+00 |
|  | 17 | 1202015 | 1202252 | PDFSTATSENSEMBLUCSC | 238 | 4 | 16276 | gene | 0.16184 | 0.59440 | Not queried | 3.76 | 2.86e-02 | 1.00e+00 |
|  | 7 | 156812806 | 156813602 | PDFSTATSENSEMBLUCSC | 797 | 8 | 39032 | island | 0.79079 | 0.20166 | Not queried | 3.76 | 3.11e-04 | 1.00e+00 |
|  | 17 | 76128522 | 76128683 | PDFSTATSENSEMBLUCSC | 162 | 7 | 17667 | tss | 0.12705 | 0.70107 | Not queried | 3.76 | 5.83e-04 | 1.00e+00 |
|  | 6 | 50813551 | 50814089 | PDFSTATSENSEMBLUCSC | 539 | 5 | 34303 | gene;island | 0.25184 | 0.05973 | Not queried | 3.76 | 5.56e-02 | 1.00e+00 |
|  | 14 | 65005951 | 65006359 | PDFSTATSENSEMBLUCSC | 409 | 10 | 12430 | tss;island | 0.54559 | 0.95729 | Not queried | 3.75 | 1.50e-03 | 1.00e+00 |
|  | 1 | 68629000 | 68629133 | PDFSTATSENSEMBLUCSC | 134 | 4 | 574 | gene;island | 0.12093 | 0.58553 | Not queried | 3.75 | 2.86e-02 | 1.00e+00 |
|  | 2 | 3383078 | 3383413 | PDFSTATSENSEMBLUCSC | 336 | 5 | 23122 | tss | 0.12765 | 0.02151 | Not queried | 3.75 | 3.10e-01 | 1.00e+00 |
|  | 1 | 174968745 | 174968980 | PDFSTATSENSEMBLUCSC | 236 | 8 | 890 | gene;island | 0.24101 | 0.02509 | Not queried | 3.75 | 1.09e-03 | 1.00e+00 |
|  | 4 | 81106240 | 81106414 | PDFSTATSENSEMBLUCSC | 175 | 6 | 30412 | tss | 0.12008 | 0.51214 | Not queried | 3.74 | 2.16e-03 | 1.00e+00 |
|  | 4 | 206112 | 206339 | PDFSTATSENSEMBLUCSC | 228 | 6 | 30175 | tss | 0.69949 | 0.23895 | Not queried | 3.74 | 1.52e-02 | 1.00e+00 |
|  | 7 | 4783964 | 4784485 | PDFSTATSENSEMBLUCSC | 522 | 6 | 36782 | gene;island | 0.10170 | 0.61541 | Not queried | 3.74 | 8.66e-03 | 1.00e+00 |
|  | 2 | 217560272 | 217560403 | PDFSTATSENSEMBLUCSC | 132 | 5 | 23833 | tss;island | 0.46452 | 0.04048 | Not queried | 3.73 | 1.59e-02 | 1.00e+00 |
|  | 6 | 147124984 | 147124996 | PDFSTATSENSEMBLUCSC | 13 | 4 | 35214 | tss | 0.53219 | 0.95440 | Not queried | 3.73 | 2.00e-01 | 1.00e+00 |
|  | 7 | 157361396 | 157361759 | PDFSTATSENSEMBLUCSC | 364 | 6 | 37264 | gene;island | 0.33668 | 0.88917 | Not queried | 3.73 | 2.16e-03 | 1.00e+00 |
|  | 11 | 61062837 | 61063144 | PDFSTATSENSEMBLUCSC | 308 | 9 | 7309 | tss | 0.85860 | 0.24345 | Not queried | 3.73 | 4.94e-04 | 1.00e+00 |
|  | 11 | 61062665 | 61063144 | PDFSTATSENSEMBLUCSC | 480 | 11 | 8297 | island | 0.85120 | 0.15562 | Not queried | 3.73 | 3.94e-04 | 1.00e+00 |
|  | X | 71458680 | 71459274 | PDFSTATSENSEMBLUCSC | 595 | 11 | 41031 | gene;island | 0.03580 | 0.42108 | Not queried | 3.73 | 3.40e-05 | 1.75e-01 |
|  | X | 71458866 | 71459274 | PDFSTATSENSEMBLUCSC | 409 | 9 | 41382 | tss | 0.03580 | 0.42108 | Not queried | 3.73 | 2.88e-04 | 1.00e+00 |
|  | 20 | 37230326 | 37230484 | PDFSTATSENSEMBLUCSC | 159 | 5 | 25698 | tss | 0.02786 | 0.29874 | Not queried | 3.73 | 7.94e-03 | 1.00e+00 |
|  | 11 | 120008836 | 120009135 | PDFSTATSENSEMBLUCSC | 300 | 6 | 8864 | island | 0.06849 | 0.61994 | Not queried | 3.73 | 6.49e-02 | 1.00e+00 |
|  | 16 | 88700622 | 88700916 | PDFSTATSENSEMBLUCSC | 295 | 4 | 16175 | island | 0.03293 | 0.31081 | Not queried | 3.72 | 2.86e-02 | 1.00e+00 |
|  | 2 | 3642098 | 3642967 | PDFSTATSENSEMBLUCSC | 870 | 11 | 23997 | island | 0.17632 | 0.79224 | Not queried | 3.72 | 1.28e-04 | 6.56e-01 |
|  | 4 | 132896266 | 132896736 | PDFSTATSENSEMBLUCSC | 471 | 5 | 31251 | island | 0.55047 | 0.79998 | Not queried | 3.72 | 7.94e-03 | 1.00e+00 |
|  | 4 | 175135048 | 175135997 | PDFSTATSENSEMBLUCSC | 950 | 9 | 31378 | island | 0.51892 | 0.06967 | Not queried | 3.72 | 4.11e-05 | 2.11e-01 |
|  | 10 | 134043618 | 134043897 | PDFSTATSENSEMBLUCSC | 280 | 5 | 4841 | gene;island | 0.34190 | 0.89247 | Not queried | 3.72 | 7.94e-03 | 1.00e+00 |
|  | 19 | 38794554 | 38794664 | PDFSTATSENSEMBLUCSC | 111 | 4 | 20713 | tss | 0.20516 | 0.76552 | Not queried | 3.72 | 2.86e-02 | 1.00e+00 |
|  | 19 | 52552025 | 52552461 | PDFSTATSENSEMBLUCSC | 437 | 10 | 22313 | island | 0.09973 | 0.55622 | Not queried | 3.72 | 1.08e-05 | 5.56e-02 |
|  | 16 | 90143751 | 90144006 | PDFSTATSENSEMBLUCSC | 256 | 5 | 16246 | island | 0.01767 | 0.30471 | Not queried | 3.72 | 7.94e-03 | 1.00e+00 |
|  | 11 | 2884118 | 2884460 | PDFSTATSENSEMBLUCSC | 343 | 5 | 7954 | island | 0.68877 | 0.20121 | Not queried | 3.72 | 7.94e-03 | 1.00e+00 |
|  | 21 | 15645649 | 15645988 | PDFSTATSENSEMBLUCSC | 340 | 4 | 26446 | tss | 0.63777 | 0.94991 | Not queried | 3.71 | 1.14e-01 | 1.00e+00 |
|  | 12 | 52300911 | 52301307 | PDFSTATSENSEMBLUCSC | 397 | 5 | 10406 | island | 0.12520 | 0.65263 | Not queried | 3.71 | 7.94e-03 | 1.00e+00 |
|  | 6 | 30883762 | 30884124 | PDFSTATSENSEMBLUCSC | 363 | 4 | 33776 | gene;island | 0.32100 | 0.89693 | Not queried | 3.71 | 2.86e-02 | 1.00e+00 |
|  | 7 | 87105022 | 87105416 | PDFSTATSENSEMBLUCSC | 395 | 5 | 37621 | tss | 0.47189 | 0.04710 | Not queried | 3.71 | 5.56e-02 | 1.00e+00 |
|  | 6 | 39016262 | 39016479 | PDFSTATSENSEMBLUCSC | 218 | 5 | 34888 | tss | 0.56546 | 0.10291 | Not queried | 3.71 | 7.94e-03 | 1.00e+00 |
|  | 1 | 201368613 | 201369031 | PDFSTATSENSEMBLUCSC | 419 | 9 | 4062 | island | 0.02824 | 0.30520 | Not queried | 3.71 | 4.11e-05 | 2.11e-01 |
|  | 11 | 17740887 | 17741070 | PDFSTATSENSEMBLUCSC | 184 | 4 | 7158 | tss | 0.05521 | 0.38981 | Not queried | 3.71 | 2.86e-02 | 1.00e+00 |
|  | 19 | 44302666 | 44303058 | PDFSTATSENSEMBLUCSC | 393 | 4 | 20043 | gene;island | 0.47159 | 0.06715 | Not queried | 3.71 | 5.71e-02 | 1.00e+00 |
|  | 2 | 21022844 | 21023042 | PDFSTATSENSEMBLUCSC | 199 | 4 | 23178 | tss | 0.92329 | 0.48250 | Not queried | 3.71 | 2.86e-02 | 1.00e+00 |
|  | 12 | 114846849 | 114847164 | PDFSTATSENSEMBLUCSC | 316 | 4 | 10009 | tss;island | 0.90333 | 0.33862 | Not queried | 3.71 | 2.86e-02 | 1.00e+00 |
|  | 2 | 45169447 | 45169681 | PDFSTATSENSEMBLUCSC | 235 | 4 | 22627 | gene;island | 0.96694 | 0.63610 | Not queried | 3.70 | 1.14e-01 | 1.00e+00 |
|  | 8 | 101315424 | 101315560 | PDFSTATSENSEMBLUCSC | 137 | 4 | 39316 | gene | 0.39488 | 0.94388 | Not queried | 3.70 | 5.71e-02 | 1.00e+00 |
|  | 3 | 64253534 | 64253818 | PDFSTATSENSEMBLUCSC | 285 | 6 | 29217 | island | 0.39519 | 0.07980 | Not queried | 3.69 | 2.16e-03 | 1.00e+00 |
|  | 17 | 79503642 | 79503877 | PDFSTATSENSEMBLUCSC | 236 | 4 | 16881 | gene;island | 0.55021 | 0.92736 | Not queried | 3.69 | 2.86e-02 | 1.00e+00 |
|  | 2 | 29337946 | 29338258 | PDFSTATSENSEMBLUCSC | 313 | 9 | 23244 | tss | 0.54966 | 0.08649 | Not queried | 3.69 | 4.11e-05 | 2.11e-01 |
|  | 1 | 22919873 | 22920169 | PDFSTATSENSEMBLUCSC | 297 | 4 | 306 | gene;island | 0.22878 | 0.77191 | Not queried | 3.69 | 2.86e-02 | 1.00e+00 |
|  | 5 | 72596646 | 72597316 | PDFSTATSENSEMBLUCSC | 671 | 9 | 32807 | island | 0.84537 | 0.25666 | Not queried | 3.69 | 4.11e-05 | 2.11e-01 |
|  | 8 | 120651190 | 120651398 | PDFSTATSENSEMBLUCSC | 209 | 4 | 39822 | tss | 0.03557 | 0.32717 | Not queried | 3.69 | 2.86e-02 | 1.00e+00 |
|  | 4 | 183721565 | 183721778 | PDFSTATSENSEMBLUCSC | 214 | 4 | 30146 | gene;island | 0.59284 | 0.92300 | Not queried | 3.69 | 2.86e-02 | 1.00e+00 |
|  | 11 | 58869663 | 58870075 | PDFSTATSENSEMBLUCSC | 413 | 5 | 8279 | island | 0.03947 | 0.42111 | Not queried | 3.69 | 7.94e-03 | 1.00e+00 |
|  | 2 | 29337946 | 29338432 | PDFSTATSENSEMBLUCSC | 487 | 10 | 24160 | island | 0.54946 | 0.06625 | Not queried | 3.69 | 7.25e-04 | 1.00e+00 |
|  | 5 | 78985425 | 78985592 | PDFSTATSENSEMBLUCSC | 168 | 9 | 32088 | tss | 0.01945 | 0.18971 | Not queried | 3.69 | 4.11e-05 | 2.11e-01 |
|  | 1 | 32708234 | 32708485 | PDFSTATSENSEMBLUCSC | 252 | 5 | 1453 | tss;island | 0.49770 | 0.04453 | Not queried | 3.69 | 1.59e-02 | 1.00e+00 |
|  | X | 134124839 | 134124960 | PDFSTATSENSEMBLUCSC | 122 | 6 | 41535 | tss | 0.09597 | 0.61884 | Not queried | 3.69 | 2.16e-03 | 1.00e+00 |
|  | 19 | 54926277 | 54926805 | PDFSTATSENSEMBLUCSC | 529 | 7 | 22370 | island | 0.03645 | 0.28555 | Not queried | 3.69 | 6.99e-03 | 1.00e+00 |
|  | 2 | 26726190 | 26726691 | PDFSTATSENSEMBLUCSC | 502 | 5 | 22577 | gene;island | 0.03542 | 0.37131 | Not queried | 3.68 | 1.59e-02 | 1.00e+00 |
|  | 8 | 1960217 | 1960466 | PDFSTATSENSEMBLUCSC | 250 | 4 | 39982 | island | 0.58200 | 0.92783 | Not queried | 3.68 | 2.86e-02 | 1.00e+00 |
|  | 22 | 24890690 | 24890833 | PDFSTATSENSEMBLUCSC | 144 | 7 | 26978 | tss;island | 0.48913 | 0.92695 | Not queried | 3.68 | 5.83e-04 | 1.00e+00 |
|  | 15 | 76004985 | 76005171 | PDFSTATSENSEMBLUCSC | 187 | 4 | 13403 | gene | 0.13489 | 0.63512 | Not queried | 3.68 | 2.86e-02 | 1.00e+00 |
|  | X | 9960096 | 9960578 | PDFSTATSENSEMBLUCSC | 483 | 4 | 41632 | island | 0.62027 | 0.95633 | Not queried | 3.68 | 2.86e-02 | 1.00e+00 |
|  | 21 | 22369802 | 22370444 | PDFSTATSENSEMBLUCSC | 643 | 6 | 26454 | tss;island | 0.04654 | 0.52412 | Not queried | 3.68 | 4.11e-02 | 1.00e+00 |
|  | 12 | 103311462 | 103311761 | PDFSTATSENSEMBLUCSC | 300 | 6 | 9926 | tss | 0.02692 | 0.31184 | Not queried | 3.68 | 4.11e-02 | 1.00e+00 |
|  | 19 | 31847893 | 31848310 | PDFSTATSENSEMBLUCSC | 418 | 6 | 21828 | island | 0.31490 | 0.03605 | Not queried | 3.68 | 8.66e-03 | 1.00e+00 |
|  | 17 | 46019006 | 46019184 | PDFSTATSENSEMBLUCSC | 179 | 5 | 16613 | gene | 0.44685 | 0.05946 | Not queried | 3.68 | 7.94e-03 | 1.00e+00 |
|  | 4 | 74719046 | 74719289 | PDFSTATSENSEMBLUCSC | 244 | 4 | 29977 | gene | 0.21612 | 0.77717 | Not queried | 3.68 | 2.86e-02 | 1.00e+00 |
|  | 17 | 21743878 | 21744126 | PDFSTATSENSEMBLUCSC | 249 | 4 | 18095 | island | 0.01761 | 0.18529 | Not queried | 3.67 | 2.86e-02 | 1.00e+00 |
|  | 21 | 42797847 | 42798386 | PDFSTATSENSEMBLUCSC | 540 | 6 | 26389 | gene;island | 0.14266 | 0.66634 | Not queried | 3.67 | 4.33e-03 | 1.00e+00 |
|  | X | 46404957 | 46405125 | PDFSTATSENSEMBLUCSC | 169 | 7 | 40971 | gene | 0.08907 | 0.57672 | Not queried | 3.67 | 5.83e-04 | 1.00e+00 |
|  | 13 | 112728799 | 112728964 | PDFSTATSENSEMBLUCSC | 166 | 4 | 11884 | island | 0.54233 | 0.21208 | Not queried | 3.67 | 5.71e-02 | 1.00e+00 |
|  | 2 | 161126295 | 161126857 | PDFSTATSENSEMBLUCSC | 563 | 7 | 24776 | island | 0.02470 | 0.30002 | Not queried | 3.67 | 5.83e-04 | 1.00e+00 |
|  | 17 | 907643 | 907886 | PDFSTATSENSEMBLUCSC | 244 | 4 | 16266 | gene;island | 0.33173 | 0.79533 | Not queried | 3.67 | 2.86e-02 | 1.00e+00 |
|  | 2 | 47499521 | 47499812 | PDFSTATSENSEMBLUCSC | 292 | 4 | 24243 | island | 0.02413 | 0.28505 | Not queried | 3.67 | 2.86e-02 | 1.00e+00 |
|  | 2 | 177053274 | 177053568 | PDFSTATSENSEMBLUCSC | 295 | 10 | 24876 | island | 0.02860 | 0.25482 | Not queried | 3.66 | 1.08e-05 | 5.56e-02 |
|  | 2 | 177053274 | 177053297 | PDFSTATSENSEMBLUCSC | 24 | 6 | 23706 | tss | 0.02740 | 0.25482 | Not queried | 3.66 | 2.16e-03 | 1.00e+00 |
|  | 2 | 27805536 | 27806117 | PDFSTATSENSEMBLUCSC | 582 | 11 | 24151 | island | 0.92048 | 0.49255 | C2orf16; ZNF512; RP11-158I13.2; AC074091.1 (margin: C2orf16; ZNF512; RP11-158I13.2; AC074091.1) | 3.66 | 5.67e-06 | 2.91e-02 |
|  | X | 152938698 | 152938907 | PDFSTATSENSEMBLUCSC | 210 | 5 | 41142 | gene;island | 0.33083 | 0.84460 | Not queried | 3.66 | 7.94e-03 | 1.00e+00 |
|  | 1 | 233462575 | 233462803 | PDFSTATSENSEMBLUCSC | 229 | 5 | 2322 | tss;island | 0.31616 | 0.06411 | Not queried | 3.66 | 5.56e-02 | 1.00e+00 |
|  | 11 | 111170578 | 111170648 | PDFSTATSENSEMBLUCSC | 71 | 4 | 7661 | tss | 0.49594 | 0.12645 | Not queried | 3.66 | 2.86e-02 | 1.00e+00 |
|  | 8 | 95653874 | 95654179 | PDFSTATSENSEMBLUCSC | 306 | 4 | 39300 | gene;island | 0.03565 | 0.32541 | Not queried | 3.66 | 2.86e-02 | 1.00e+00 |
|  | 17 | 74075092 | 74075336 | PDFSTATSENSEMBLUCSC | 245 | 5 | 18737 | island | 0.25501 | 0.83705 | Not queried | 3.66 | 7.94e-03 | 1.00e+00 |
|  | 17 | 37123669 | 37123949 | PDFSTATSENSEMBLUCSC | 281 | 8 | 17269 | tss | 0.29037 | 0.78032 | Not queried | 3.66 | 3.11e-04 | 1.00e+00 |
|  | 17 | 48609923 | 48610255 | PDFSTATSENSEMBLUCSC | 333 | 5 | 18520 | island | 0.03998 | 0.38219 | Not queried | 3.66 | 7.94e-03 | 1.00e+00 |
|  | 1 | 2918373 | 2918752 | PDFSTATSENSEMBLUCSC | 380 | 5 | 2520 | island | 0.21649 | 0.82686 | Not queried | 3.66 | 7.94e-03 | 1.00e+00 |
|  | 17 | 48071685 | 48071988 | PDFSTATSENSEMBLUCSC | 304 | 4 | 16643 | gene;island | 0.02420 | 0.20344 | Not queried | 3.66 | 2.86e-02 | 1.00e+00 |
|  | 8 | 22735111 | 22735478 | PDFSTATSENSEMBLUCSC | 368 | 5 | 39174 | gene | 0.17750 | 0.67686 | Not queried | 3.65 | 3.17e-02 | 1.00e+00 |
|  | 15 | 43531947 | 43532243 | PDFSTATSENSEMBLUCSC | 297 | 4 | 13285 | gene | 0.40745 | 0.90303 | Not queried | 3.65 | 2.86e-02 | 1.00e+00 |
|  | 17 | 64188403 | 64188644 | PDFSTATSENSEMBLUCSC | 242 | 4 | 17562 | tss;island | 0.41098 | 0.05268 | Not queried | 3.65 | 3.43e-01 | 1.00e+00 |
|  | 6 | 30923241 | 30924004 | PDFSTATSENSEMBLUCSC | 764 | 17 | 35684 | island | 0.35674 | 0.04498 | DPCR1; HCG21 (margin: DPCR1; HCG21) | 3.65 | 5.86e-07 | 3.01e-03 |
|  | 7 | 27154262 | 27155039 | PDFSTATSENSEMBLUCSC | 778 | 14 | 37425 | tss | 0.64307 | 0.07005 | HOXA-AS2; HOXA3 (margin: HOXA-AS2; HOXA2; HOXA3) | 3.65 | 1.50e-06 | 7.69e-03 |
|  | 6 | 39281421 | 39281885 | PDFSTATSENSEMBLUCSC | 465 | 5 | 34260 | gene;island | 0.03741 | 0.15877 | Not queried | 3.65 | 7.94e-03 | 1.00e+00 |
|  | 2 | 175499113 | 175499377 | PDFSTATSENSEMBLUCSC | 265 | 4 | 22893 | gene | 0.35776 | 0.87531 | Not queried | 3.64 | 5.71e-02 | 1.00e+00 |
|  | 11 | 66103823 | 66104174 | PDFSTATSENSEMBLUCSC | 352 | 7 | 8487 | island | 0.20516 | 0.76316 | Not queried | 3.64 | 1.75e-02 | 1.00e+00 |
|  | X | 153707710 | 153707810 | PDFSTATSENSEMBLUCSC | 101 | 4 | 41601 | tss | 0.10889 | 0.58485 | Not queried | 3.64 | 2.86e-02 | 1.00e+00 |
|  | 3 | 129407530 | 129407548 | PDFSTATSENSEMBLUCSC | 19 | 4 | 28545 | tss | 0.41856 | 0.04549 | Not queried | 3.64 | 2.86e-02 | 1.00e+00 |
|  | 1 | 55267006 | 55267293 | PDFSTATSENSEMBLUCSC | 288 | 7 | 1645 | tss | 0.28000 | 0.74490 | Not queried | 3.64 | 5.83e-04 | 1.00e+00 |
|  | 20 | 30639816 | 30640041 | PDFSTATSENSEMBLUCSC | 226 | 7 | 25632 | tss | 0.34397 | 0.03529 | Not queried | 3.64 | 2.33e-03 | 1.00e+00 |
|  | 12 | 119772354 | 119772577 | PDFSTATSENSEMBLUCSC | 224 | 5 | 10846 | island | 0.39044 | 0.04899 | Not queried | 3.64 | 9.52e-02 | 1.00e+00 |
|  | 15 | 96960214 | 96960725 | PDFSTATSENSEMBLUCSC | 512 | 5 | 14463 | island | 0.01679 | 0.23995 | Not queried | 3.63 | 7.94e-03 | 1.00e+00 |
|  | 16 | 2140326 | 2140438 | PDFSTATSENSEMBLUCSC | 113 | 4 | 14571 | gene;tss;island | 0.67193 | 0.94646 | Not queried | 3.63 | 5.71e-02 | 1.00e+00 |
|  | 21 | 19616939 | 19617145 | PDFSTATSENSEMBLUCSC | 207 | 4 | 26453 | tss;island | 0.09572 | 0.51830 | Not queried | 3.63 | 3.43e-01 | 1.00e+00 |
|  | 18 | 10588980 | 10589360 | PDFSTATSENSEMBLUCSC | 381 | 5 | 19358 | island | 0.69949 | 0.92002 | Not queried | 3.63 | 7.94e-03 | 1.00e+00 |
|  | 15 | 52404977 | 52405162 | PDFSTATSENSEMBLUCSC | 186 | 7 | 13649 | tss | 0.16920 | 0.89112 | Not queried | 3.62 | 3.79e-02 | 1.00e+00 |
|  | 11 | 65816463 | 65816985 | PDFSTATSENSEMBLUCSC | 523 | 10 | 8475 | island | 0.84637 | 0.22051 | Not queried | 3.62 | 1.08e-05 | 5.56e-02 |
|  | X | 153715055 | 153715609 | PDFSTATSENSEMBLUCSC | 555 | 8 | 41602 | tss;island | 0.11190 | 0.63818 | Not queried | 3.62 | 1.04e-02 | 1.00e+00 |
|  | 12 | 66582582 | 66582805 | PDFSTATSENSEMBLUCSC | 224 | 4 | 9823 | tss;island | 0.28219 | 0.84782 | Not queried | 3.62 | 2.86e-02 | 1.00e+00 |
|  | 16 | 55542668 | 55542751 | PDFSTATSENSEMBLUCSC | 84 | 4 | 15209 | tss | 0.71354 | 0.19947 | Not queried | 3.62 | 2.00e-01 | 1.00e+00 |
|  | 17 | 46692347 | 46692592 | PDFSTATSENSEMBLUCSC | 246 | 7 | 17434 | tss | 0.03055 | 0.42670 | Not queried | 3.62 | 4.08e-03 | 1.00e+00 |
|  | 20 | 19915716 | 19915874 | PDFSTATSENSEMBLUCSC | 159 | 6 | 25331 | gene | 0.10944 | 0.66903 | Not queried | 3.61 | 2.16e-03 | 1.00e+00 |
|  | 17 | 1012288 | 1012579 | PDFSTATSENSEMBLUCSC | 292 | 4 | 16272 | gene;island | 0.01922 | 0.21264 | Not queried | 3.61 | 2.86e-02 | 1.00e+00 |
|  | 3 | 183948058 | 183948313 | PDFSTATSENSEMBLUCSC | 256 | 5 | 28711 | tss | 0.02035 | 0.19749 | Not queried | 3.61 | 3.17e-02 | 1.00e+00 |
|  | 2 | 237077920 | 237078733 | PDFSTATSENSEMBLUCSC | 814 | 9 | 25187 | island | 0.31656 | 0.03653 | Not queried | 3.61 | 8.23e-05 | 4.23e-01 |
|  | 2 | 121199631 | 121199920 | PDFSTATSENSEMBLUCSC | 290 | 4 | 24628 | island | 0.05851 | 0.40190 | Not queried | 3.61 | 2.86e-02 | 1.00e+00 |
|  | 20 | 25566178 | 25566563 | PDFSTATSENSEMBLUCSC | 386 | 7 | 25613 | tss;island | 0.23822 | 0.02498 | Not queried | 3.61 | 2.09e-01 | 1.00e+00 |
|  | 16 | 55866757 | 55867072 | PDFSTATSENSEMBLUCSC | 316 | 5 | 14724 | gene;island | 0.49457 | 0.04230 | Not queried | 3.61 | 7.94e-03 | 1.00e+00 |
|  | 10 | 22623821 | 22624374 | PDFSTATSENSEMBLUCSC | 554 | 5 | 5582 | island | 0.90058 | 0.40594 | Not queried | 3.61 | 7.94e-03 | 1.00e+00 |
|  | 8 | 116660311 | 116660697 | PDFSTATSENSEMBLUCSC | 387 | 4 | 39340 | gene;island | 0.08027 | 0.48101 | Not queried | 3.61 | 5.71e-02 | 1.00e+00 |
|  | 3 | 126113327 | 126113743 | PDFSTATSENSEMBLUCSC | 417 | 10 | 28515 | tss | 0.29857 | 0.87328 | Not queried | 3.61 | 8.93e-03 | 1.00e+00 |
|  | 17 | 79881468 | 79881543 | PDFSTATSENSEMBLUCSC | 76 | 5 | 16896 | gene;tss;island | 0.69645 | 0.95331 | Not queried | 3.61 | 7.94e-03 | 1.00e+00 |
|  | 7 | 751830 | 753007 | PDFSTATSENSEMBLUCSC | 1178 | 16 | 36654 | gene;island | 0.49666 | 0.94284 | Not queried | 3.60 | 1.08e-02 | 1.00e+00 |
|  | 7 | 143106001 | 143106298 | PDFSTATSENSEMBLUCSC | 298 | 7 | 37890 | tss | 0.02210 | 0.25330 | Not queried | 3.60 | 5.83e-04 | 1.00e+00 |
|  | 22 | 32600722 | 32601274 | PDFSTATSENSEMBLUCSC | 553 | 4 | 27047 | tss | 0.04825 | 0.36422 | Not queried | 3.60 | 2.86e-02 | 1.00e+00 |
|  | X | 110513791 | 110513957 | PDFSTATSENSEMBLUCSC | 167 | 4 | 41474 | tss | 0.10728 | 0.69269 | Not queried | 3.60 | 2.86e-02 | 1.00e+00 |
|  | 17 | 46691820 | 46692592 | PDFSTATSENSEMBLUCSC | 773 | 12 | 18456 | island | 0.03498 | 0.34582 | Not queried | 3.60 | 1.41e-05 | 7.22e-02 |
|  | 7 | 75157345 | 75157765 | PDFSTATSENSEMBLUCSC | 421 | 9 | 38521 | island | 0.24227 | 0.02228 | Not queried | 3.60 | 1.85e-03 | 1.00e+00 |
|  | 22 | 42078217 | 42078723 | PDFSTATSENSEMBLUCSC | 507 | 7 | 26842 | gene;island | 0.01263 | 0.22660 | Not queried | 3.60 | 1.75e-02 | 1.00e+00 |
|  | 1 | 55266578 | 55267293 | PDFSTATSENSEMBLUCSC | 716 | 10 | 3295 | island | 0.28248 | 0.76152 | Not queried | 3.60 | 4.33e-05 | 2.23e-01 |
|  | 11 | 73020351 | 73020729 | PDFSTATSENSEMBLUCSC | 379 | 5 | 6803 | gene;island | 0.04201 | 0.34721 | Not queried | 3.60 | 7.94e-03 | 1.00e+00 |
|  | 7 | 48019205 | 48019713 | PDFSTATSENSEMBLUCSC | 509 | 5 | 38417 | island | 0.45393 | 0.94887 | Not queried | 3.60 | 1.51e-01 | 1.00e+00 |
|  | 19 | 40324746 | 40325100 | PDFSTATSENSEMBLUCSC | 355 | 10 | 21972 | island | 0.04359 | 0.39772 | Not queried | 3.60 | 1.05e-03 | 1.00e+00 |
|  | X | 54833733 | 54833994 | PDFSTATSENSEMBLUCSC | 262 | 4 | 41336 | tss;island | 0.64376 | 0.07757 | Not queried | 3.60 | 1.14e-01 | 1.00e+00 |
|  | 6 | 31165290 | 31165512 | PDFSTATSENSEMBLUCSC | 223 | 9 | 34740 | tss | 0.02763 | 0.23511 | Not queried | 3.60 | 4.11e-05 | 2.11e-01 |
|  | 4 | 46995474 | 46995821 | PDFSTATSENSEMBLUCSC | 348 | 7 | 30969 | island | 0.03167 | 0.47590 | Not queried | 3.60 | 5.83e-04 | 1.00e+00 |
|  | 2 | 26395359 | 26395556 | PDFSTATSENSEMBLUCSC | 198 | 4 | 23199 | tss;island | 0.27900 | 0.05456 | Not queried | 3.60 | 2.86e-02 | 1.00e+00 |
|  | 19 | 46318439 | 46318918 | PDFSTATSENSEMBLUCSC | 480 | 6 | 22123 | island | 0.38145 | 0.05483 | Not queried | 3.60 | 2.16e-03 | 1.00e+00 |
|  | 6 | 30882994 | 30883203 | PDFSTATSENSEMBLUCSC | 210 | 5 | 33775 | gene;island | 0.49211 | 0.93110 | Not queried | 3.60 | 9.52e-02 | 1.00e+00 |
|  | 15 | 83953690 | 83954395 | PDFSTATSENSEMBLUCSC | 706 | 18 | 13837 | tss;island | 0.49770 | 0.10274 | Not queried | 3.60 | 4.89e-05 | 2.51e-01 |
|  | 3 | 72704324 | 72704701 | PDFSTATSENSEMBLUCSC | 378 | 5 | 29238 | island | 0.43041 | 0.90118 | Not queried | 3.59 | 7.94e-03 | 1.00e+00 |
|  | 15 | 37110422 | 37110854 | PDFSTATSENSEMBLUCSC | 433 | 7 | 13980 | island | 0.49537 | 0.95698 | Not queried | 3.59 | 2.33e-03 | 1.00e+00 |
|  | X | 48931388 | 48932122 | PDFSTATSENSEMBLUCSC | 735 | 12 | 41781 | island | 0.20282 | 0.02440 | PRAF2; WDR45 (margin: CCDC120; RNU4-52P; PRAF2; WDR45) | 3.59 | 1.48e-06 | 7.60e-03 |
|  | 7 | 114561804 | 114562063 | PDFSTATSENSEMBLUCSC | 260 | 5 | 37782 | tss;island | 0.68533 | 0.06011 | Not queried | 3.59 | 5.56e-02 | 1.00e+00 |
|  | 10 | 46168377 | 46168597 | PDFSTATSENSEMBLUCSC | 221 | 6 | 5044 | tss | 0.01840 | 0.16794 | Not queried | 3.59 | 2.16e-03 | 1.00e+00 |
|  | 4 | 81106240 | 81106463 | PDFSTATSENSEMBLUCSC | 224 | 7 | 31079 | island | 0.10689 | 0.53127 | Not queried | 3.59 | 5.83e-04 | 1.00e+00 |
|  | X | 101967017 | 101967307 | PDFSTATSENSEMBLUCSC | 291 | 7 | 41433 | tss;island | 0.35719 | 0.03342 | Not queried | 3.58 | 3.79e-02 | 1.00e+00 |
|  | 1 | 44398868 | 44399069 | PDFSTATSENSEMBLUCSC | 202 | 4 | 3166 | island | 0.22333 | 0.83567 | Not queried | 3.58 | 5.71e-02 | 1.00e+00 |
|  | 7 | 27179161 | 27179432 | PDFSTATSENSEMBLUCSC | 272 | 4 | 38282 | island | 0.41837 | 0.06203 | Not queried | 3.58 | 2.86e-02 | 1.00e+00 |
|  | 10 | 61468548 | 61468757 | PDFSTATSENSEMBLUCSC | 210 | 4 | 4583 | gene;island | 0.25755 | 0.02851 | Not queried | 3.58 | 2.86e-02 | 1.00e+00 |
|  | 6 | 26273029 | 26273199 | PDFSTATSENSEMBLUCSC | 171 | 6 | 34640 | tss | 0.65967 | 0.93725 | Not queried | 3.58 | 2.16e-03 | 1.00e+00 |
|  | 3 | 52479069 | 52479108 | PDFSTATSENSEMBLUCSC | 40 | 4 | 28338 | tss | 0.05321 | 0.39636 | Not queried | 3.58 | 2.86e-02 | 1.00e+00 |
|  | X | 63425713 | 63425930 | PDFSTATSENSEMBLUCSC | 218 | 7 | 41349 | tss | 0.26076 | 0.02480 | Not queried | 3.58 | 5.83e-04 | 1.00e+00 |
|  | 12 | 119772354 | 119772421 | PDFSTATSENSEMBLUCSC | 68 | 4 | 10025 | tss | 0.35924 | 0.03701 | Not queried | 3.58 | 2.00e-01 | 1.00e+00 |
|  | 7 | 150020818 | 150020945 | PDFSTATSENSEMBLUCSC | 128 | 6 | 37911 | tss | 0.42384 | 0.06068 | Not queried | 3.58 | 8.66e-03 | 1.00e+00 |
|  | 4 | 122853693 | 122853965 | PDFSTATSENSEMBLUCSC | 273 | 4 | 30070 | gene;island | 0.32779 | 0.87871 | Not queried | 3.58 | 2.86e-02 | 1.00e+00 |
|  | X | 53468600 | 53468978 | PDFSTATSENSEMBLUCSC | 379 | 4 | 41810 | island | 0.04589 | 0.38209 | Not queried | 3.57 | 2.86e-02 | 1.00e+00 |
|  | 10 | 134791323 | 134791660 | PDFSTATSENSEMBLUCSC | 338 | 5 | 6304 | island | 0.45129 | 0.90740 | Not queried | 3.57 | 7.94e-03 | 1.00e+00 |
|  | 11 | 6341842 | 6341908 | PDFSTATSENSEMBLUCSC | 67 | 7 | 7086 | tss | 0.29122 | 0.03008 | Not queried | 3.57 | 5.83e-04 | 1.00e+00 |
|  | 1 | 228291486 | 228291705 | PDFSTATSENSEMBLUCSC | 220 | 5 | 2293 | tss;island | 0.52664 | 0.06649 | Not queried | 3.57 | 7.94e-03 | 1.00e+00 |
|  | X | 152677200 | 152677554 | PDFSTATSENSEMBLUCSC | 355 | 4 | 42108 | island | 0.49146 | 0.89301 | Not queried | 3.57 | 2.86e-02 | 1.00e+00 |
|  | 11 | 17740887 | 17741243 | PDFSTATSENSEMBLUCSC | 357 | 5 | 8082 | island | 0.06168 | 0.40117 | Not queried | 3.57 | 9.52e-02 | 1.00e+00 |
|  | 5 | 72594461 | 72594851 | PDFSTATSENSEMBLUCSC | 391 | 4 | 32805 | island | 0.53933 | 0.09249 | Not queried | 3.57 | 2.86e-02 | 1.00e+00 |
|  | 7 | 45961078 | 45961569 | PDFSTATSENSEMBLUCSC | 492 | 16 | 37522 | tss;island | 0.06329 | 0.55247 | Not queried | 3.57 | 1.15e-05 | 5.91e-02 |
|  | 18 | 74499372 | 74499584 | PDFSTATSENSEMBLUCSC | 213 | 5 | 19546 | island | 0.55125 | 0.92224 | Not queried | 3.57 | 7.94e-03 | 1.00e+00 |
|  | 4 | 6202379 | 6202609 | PDFSTATSENSEMBLUCSC | 231 | 6 | 30231 | tss;island | 0.25372 | 0.02415 | Not queried | 3.57 | 1.80e-01 | 1.00e+00 |
|  | 5 | 140561893 | 140562236 | PDFSTATSENSEMBLUCSC | 344 | 6 | 31755 | gene;island | 0.02879 | 0.22533 | Not queried | 3.57 | 9.31e-02 | 1.00e+00 |
|  | 14 | 91720227 | 91720696 | PDFSTATSENSEMBLUCSC | 470 | 5 | 12531 | tss | 0.53044 | 0.11793 | Not queried | 3.57 | 9.52e-02 | 1.00e+00 |
|  | 2 | 152830505 | 152830665 | PDFSTATSENSEMBLUCSC | 161 | 5 | 22860 | gene;tss | 0.30301 | 0.06135 | Not queried | 3.56 | 7.94e-03 | 1.00e+00 |
|  | 11 | 67183078 | 67183616 | PDFSTATSENSEMBLUCSC | 539 | 8 | 8523 | island | 0.04482 | 0.35674 | Not queried | 3.56 | 3.79e-02 | 1.00e+00 |
|  | 16 | 2765620 | 2765900 | PDFSTATSENSEMBLUCSC | 281 | 5 | 14581 | gene;island | 0.04634 | 0.41905 | Not queried | 3.56 | 3.17e-02 | 1.00e+00 |
|  | 5 | 140729429 | 140730092 | PDFSTATSENSEMBLUCSC | 664 | 9 | 31762 | gene;island | 0.01808 | 0.30962 | Not queried | 3.56 | 1.85e-03 | 1.00e+00 |
|  | 2 | 19558375 | 19558569 | PDFSTATSENSEMBLUCSC | 195 | 4 | 23167 | tss | 0.28931 | 0.04295 | Not queried | 3.56 | 2.86e-02 | 1.00e+00 |
|  | 7 | 76026853 | 76027101 | PDFSTATSENSEMBLUCSC | 249 | 4 | 36993 | gene;island | 0.04326 | 0.42570 | Not queried | 3.56 | 2.86e-02 | 1.00e+00 |
|  | 1 | 106623429 | 106623967 | PDFSTATSENSEMBLUCSC | 539 | 6 | 3528 | island | 0.21946 | 0.73596 | Not queried | 3.56 | 4.11e-02 | 1.00e+00 |
|  | 5 | 149792330 | 149792515 | PDFSTATSENSEMBLUCSC | 186 | 6 | 32370 | tss | 0.56700 | 0.11597 | Not queried | 3.56 | 2.16e-03 | 1.00e+00 |
|  | 6 | 30614081 | 30614422 | PDFSTATSENSEMBLUCSC | 342 | 8 | 34713 | tss;island | 0.11650 | 0.73252 | Not queried | 3.55 | 1.86e-03 | 1.00e+00 |
|  | 11 | 118123012 | 118123243 | PDFSTATSENSEMBLUCSC | 232 | 7 | 7706 | tss | 0.14061 | 0.77869 | Not queried | 3.55 | 6.99e-03 | 1.00e+00 |
|  | X | 84634308 | 84634593 | PDFSTATSENSEMBLUCSC | 286 | 5 | 41044 | gene | 0.43370 | 0.84161 | Not queried | 3.55 | 7.94e-03 | 1.00e+00 |
|  | 4 | 118006747 | 118006832 | PDFSTATSENSEMBLUCSC | 86 | 6 | 30511 | tss | 0.05099 | 0.33893 | Not queried | 3.55 | 2.16e-03 | 1.00e+00 |
|  | 19 | 2281919 | 2282207 | PDFSTATSENSEMBLUCSC | 289 | 5 | 21241 | island | 0.31482 | 0.07555 | Not queried | 3.55 | 1.59e-02 | 1.00e+00 |
|  | 3 | 129694392 | 129694683 | PDFSTATSENSEMBLUCSC | 292 | 4 | 27903 | gene;island | 0.73879 | 0.20467 | Not queried | 3.55 | 2.86e-02 | 1.00e+00 |
|  | 6 | 30652202 | 30652399 | PDFSTATSENSEMBLUCSC | 198 | 9 | 33739 | gene;island | 0.09131 | 0.58464 | Not queried | 3.55 | 4.11e-05 | 2.11e-01 |
|  | 5 | 26669610 | 26669882 | PDFSTATSENSEMBLUCSC | 273 | 4 | 32650 | island | 0.17999 | 0.70779 | Not queried | 3.55 | 2.86e-02 | 1.00e+00 |
|  | 4 | 74718978 | 74719289 | PDFSTATSENSEMBLUCSC | 312 | 5 | 31044 | island | 0.18770 | 0.76356 | Not queried | 3.55 | 7.94e-03 | 1.00e+00 |
|  | 4 | 123747447 | 123747750 | PDFSTATSENSEMBLUCSC | 304 | 7 | 30533 | tss;island | 0.27870 | 0.01799 | Not queried | 3.54 | 4.08e-03 | 1.00e+00 |
|  | 6 | 32119616 | 32120324 | PDFSTATSENSEMBLUCSC | 709 | 11 | 35840 | island | 0.24073 | 0.64990 | Not queried | 3.54 | 3.40e-05 | 1.75e-01 |
|  | 2 | 177053345 | 177053568 | PDFSTATSENSEMBLUCSC | 224 | 4 | 22903 | gene | 0.03280 | 0.28477 | Not queried | 3.54 | 2.86e-02 | 1.00e+00 |
|  | X | 45060311 | 45060544 | PDFSTATSENSEMBLUCSC | 234 | 4 | 41269 | tss | 0.38219 | 0.84176 | Not queried | 3.54 | 5.71e-02 | 1.00e+00 |
|  | 11 | 46940434 | 46940613 | PDFSTATSENSEMBLUCSC | 180 | 4 | 7261 | tss;island | 0.14475 | 0.02051 | Not queried | 3.54 | 1.14e-01 | 1.00e+00 |
|  | 10 | 104211296 | 104211488 | PDFSTATSENSEMBLUCSC | 193 | 6 | 6071 | island | 0.07236 | 0.45042 | Not queried | 3.54 | 2.16e-03 | 1.00e+00 |
|  | 12 | 56546403 | 56546720 | PDFSTATSENSEMBLUCSC | 318 | 4 | 9149 | gene | 0.10807 | 0.01612 | Not queried | 3.53 | 1.14e-01 | 1.00e+00 |
|  | 16 | 215410 | 215960 | PDFSTATSENSEMBLUCSC | 551 | 7 | 14931 | tss | 0.03073 | 0.26423 | Not queried | 3.53 | 5.83e-04 | 1.00e+00 |
|  | 3 | 42577680 | 42577884 | PDFSTATSENSEMBLUCSC | 205 | 5 | 27690 | gene;island | 0.25523 | 0.79853 | Not queried | 3.53 | 7.94e-03 | 1.00e+00 |
|  | 17 | 8702369 | 8702896 | PDFSTATSENSEMBLUCSC | 528 | 9 | 17975 | island | 0.15990 | 0.68355 | Not queried | 3.53 | 7.82e-04 | 1.00e+00 |
|  | 18 | 3011810 | 3012017 | PDFSTATSENSEMBLUCSC | 208 | 4 | 19323 | island | 0.11892 | 0.59414 | Not queried | 3.53 | 2.00e-01 | 1.00e+00 |
|  | 19 | 50015511 | 50015532 | PDFSTATSENSEMBLUCSC | 22 | 4 | 20917 | tss;island | 0.25738 | 0.71777 | Not queried | 3.52 | 2.86e-02 | 1.00e+00 |
|  | 13 | 100310162 | 100310405 | PDFSTATSENSEMBLUCSC | 244 | 4 | 11177 | gene;island | 0.34840 | 0.04068 | Not queried | 3.52 | 2.86e-02 | 1.00e+00 |
|  | 14 | 103655242 | 103655773 | PDFSTATSENSEMBLUCSC | 532 | 5 | 13154 | island | 0.43128 | 0.92869 | Not queried | 3.52 | 7.94e-03 | 1.00e+00 |
|  | 12 | 132990805 | 132991277 | PDFSTATSENSEMBLUCSC | 473 | 5 | 11023 | island | 0.44983 | 0.73973 | Not queried | 3.51 | 2.22e-01 | 1.00e+00 |
|  | 3 | 48632625 | 48633144 | PDFSTATSENSEMBLUCSC | 520 | 8 | 28259 | tss | 0.31949 | 0.82358 | Not queried | 3.51 | 1.55e-04 | 7.99e-01 |
|  | X | 153046480 | 153046894 | PDFSTATSENSEMBLUCSC | 415 | 5 | 41146 | gene | 0.30492 | 0.85861 | Not queried | 3.51 | 7.94e-03 | 1.00e+00 |
|  | 2 | 3452347 | 3452624 | PDFSTATSENSEMBLUCSC | 278 | 6 | 22512 | gene;island | 0.55304 | 0.95486 | Not queried | 3.51 | 4.33e-03 | 1.00e+00 |
|  | 4 | 140586921 | 140587179 | PDFSTATSENSEMBLUCSC | 259 | 6 | 30085 | gene | 0.05406 | 0.35972 | Not queried | 3.51 | 2.16e-03 | 1.00e+00 |
|  | X | 63425537 | 63425930 | PDFSTATSENSEMBLUCSC | 394 | 8 | 41827 | island | 0.26471 | 0.02676 | Not queried | 3.51 | 6.22e-04 | 1.00e+00 |
|  | 12 | 25405085 | 25405317 | PDFSTATSENSEMBLUCSC | 233 | 5 | 9587 | tss | 0.65515 | 0.18726 | Not queried | 3.51 | 1.59e-02 | 1.00e+00 |
|  | 15 | 62457456 | 62457681 | PDFSTATSENSEMBLUCSC | 226 | 4 | 14171 | island | 0.48703 | 0.09367 | Not queried | 3.51 | 2.86e-02 | 1.00e+00 |
|  | 1 | 35258435 | 35258933 | PDFSTATSENSEMBLUCSC | 499 | 4 | 3019 | island | 0.05129 | 0.45454 | Not queried | 3.50 | 2.00e-01 | 1.00e+00 |
|  | 12 | 115104274 | 115104983 | PDFSTATSENSEMBLUCSC | 710 | 7 | 10816 | island | 0.67858 | 0.09105 | Not queried | 3.50 | 2.62e-02 | 1.00e+00 |
|  | 3 | 48632484 | 48633144 | PDFSTATSENSEMBLUCSC | 661 | 11 | 29047 | island | 0.38028 | 0.84593 | COL7A1 (margin: COL7A1; UQCRC1) | 3.50 | 5.67e-06 | 2.91e-02 |
|  | 7 | 56516076 | 56516504 | PDFSTATSENSEMBLUCSC | 429 | 9 | 37546 | tss;island | 0.07804 | 0.58951 | Not queried | 3.50 | 7.77e-03 | 1.00e+00 |
|  | 11 | 64937570 | 64937697 | PDFSTATSENSEMBLUCSC | 128 | 6 | 7398 | tss | 0.22561 | 0.79159 | Not queried | 3.50 | 2.16e-03 | 1.00e+00 |
|  | 5 | 140201153 | 140201746 | PDFSTATSENSEMBLUCSC | 594 | 5 | 31739 | gene;island | 0.20369 | 0.66624 | Not queried | 3.50 | 7.94e-03 | 1.00e+00 |
|  | 11 | 9635889 | 9636089 | PDFSTATSENSEMBLUCSC | 201 | 4 | 8029 | island | 0.14056 | 0.56514 | Not queried | 3.50 | 2.86e-02 | 1.00e+00 |
|  | 11 | 393328 | 393648 | PDFSTATSENSEMBLUCSC | 321 | 5 | 6990 | tss;island | 0.30718 | 0.75022 | Not queried | 3.50 | 7.94e-03 | 1.00e+00 |
|  | 10 | 3824387 | 3824687 | PDFSTATSENSEMBLUCSC | 301 | 4 | 4463 | gene;island | 0.16658 | 0.71206 | Not queried | 3.50 | 2.86e-02 | 1.00e+00 |
|  | 19 | 51416010 | 51416213 | PDFSTATSENSEMBLUCSC | 204 | 4 | 22294 | island | 0.17141 | 0.79625 | Not queried | 3.50 | 1.14e-01 | 1.00e+00 |
|  | 3 | 96533511 | 96534005 | PDFSTATSENSEMBLUCSC | 495 | 6 | 27829 | gene;island | 0.02482 | 0.30606 | Not queried | 3.49 | 4.33e-03 | 1.00e+00 |
|  | 19 | 51520093 | 51520658 | PDFSTATSENSEMBLUCSC | 566 | 6 | 20150 | gene;island | 0.06346 | 0.47685 | Not queried | 3.49 | 2.16e-03 | 1.00e+00 |
|  | 12 | 117407879 | 117408186 | PDFSTATSENSEMBLUCSC | 308 | 5 | 9307 | gene | 0.43106 | 0.94099 | Not queried | 3.49 | 9.52e-02 | 1.00e+00 |
|  | 9 | 140473545 | 140473875 | PDFSTATSENSEMBLUCSC | 331 | 5 | 40794 | tss;island | 0.30322 | 0.01392 | Not queried | 3.49 | 2.22e-01 | 1.00e+00 |
|  | 8 | 42234724 | 42234975 | PDFSTATSENSEMBLUCSC | 252 | 5 | 39614 | tss | 0.25761 | 0.83910 | Not queried | 3.49 | 7.94e-03 | 1.00e+00 |
|  | 19 | 49198900 | 49199234 | PDFSTATSENSEMBLUCSC | 335 | 5 | 22195 | island | 0.04485 | 0.36738 | Not queried | 3.49 | 1.51e-01 | 1.00e+00 |
|  | 1 | 99469577 | 99469819 | PDFSTATSENSEMBLUCSC | 243 | 4 | 646 | gene;island | 0.46760 | 0.08941 | Not queried | 3.49 | 1.14e-01 | 1.00e+00 |
|  | 10 | 104211319 | 104211488 | PDFSTATSENSEMBLUCSC | 170 | 5 | 5308 | tss | 0.07646 | 0.45134 | Not queried | 3.49 | 7.94e-03 | 1.00e+00 |
|  | 2 | 25391656 | 25391911 | PDFSTATSENSEMBLUCSC | 256 | 10 | 23193 | tss | 0.51045 | 0.05862 | Not queried | 3.48 | 4.33e-05 | 2.23e-01 |
|  | 11 | 93583509 | 93583973 | PDFSTATSENSEMBLUCSC | 465 | 8 | 8709 | island | 0.31969 | 0.85026 | Not queried | 3.48 | 1.55e-04 | 7.99e-01 |
|  | 11 | 93583672 | 93583973 | PDFSTATSENSEMBLUCSC | 302 | 6 | 7604 | tss | 0.33996 | 0.85728 | Not queried | 3.48 | 2.16e-03 | 1.00e+00 |
|  | 10 | 135049991 | 135050355 | PDFSTATSENSEMBLUCSC | 365 | 13 | 5437 | tss;island | 0.33678 | 0.03069 | VENTX (margin: KNDC1; UTF1; VENTX; MIR202) | 3.48 | 1.35e-06 | 6.92e-03 |
|  | 3 | 48694451 | 48694673 | PDFSTATSENSEMBLUCSC | 223 | 4 | 27726 | gene;island | 0.36080 | 0.90796 | Not queried | 3.48 | 2.86e-02 | 1.00e+00 |
|  | 13 | 51417923 | 51418221 | PDFSTATSENSEMBLUCSC | 299 | 8 | 11420 | tss | 0.10784 | 0.54352 | Not queried | 3.48 | 1.04e-02 | 1.00e+00 |
|  | 12 | 4829115 | 4829690 | PDFSTATSENSEMBLUCSC | 576 | 7 | 9476 | tss | 0.10945 | 0.61648 | Not queried | 3.47 | 1.17e-03 | 1.00e+00 |
|  | 8 | 22926478 | 22926911 | PDFSTATSENSEMBLUCSC | 434 | 10 | 40080 | island | 0.35774 | 0.03085 | Not queried | 3.47 | 1.85e-02 | 1.00e+00 |
|  | 3 | 62305003 | 62305285 | PDFSTATSENSEMBLUCSC | 283 | 5 | 28382 | tss;island | 0.60053 | 0.18397 | Not queried | 3.47 | 9.52e-02 | 1.00e+00 |
|  | X | 70442524 | 70443001 | PDFSTATSENSEMBLUCSC | 478 | 6 | 41373 | tss | 0.45870 | 0.92457 | Not queried | 3.47 | 1.52e-02 | 1.00e+00 |
|  | 13 | 90015518 | 90015912 | PDFSTATSENSEMBLUCSC | 395 | 4 | 11782 | island | 0.25485 | 0.76790 | Not queried | 3.47 | 1.14e-01 | 1.00e+00 |
|  | 2 | 172945058 | 172945383 | PDFSTATSENSEMBLUCSC | 326 | 5 | 22884 | gene;island | 0.79300 | 0.20638 | Not queried | 3.47 | 3.17e-02 | 1.00e+00 |
|  | 3 | 49941376 | 49941613 | PDFSTATSENSEMBLUCSC | 238 | 7 | 28297 | tss;island | 0.05276 | 0.38100 | Not queried | 3.47 | 6.99e-03 | 1.00e+00 |
|  | 20 | 33146263 | 33146543 | PDFSTATSENSEMBLUCSC | 281 | 6 | 25362 | gene;island | 0.05474 | 0.35310 | Not queried | 3.47 | 2.16e-03 | 1.00e+00 |
|  | 2 | 80530701 | 80530948 | PDFSTATSENSEMBLUCSC | 248 | 4 | 22718 | gene;island | 0.04706 | 0.35017 | Not queried | 3.47 | 2.86e-02 | 1.00e+00 |
|  | 7 | 121956502 | 121956890 | PDFSTATSENSEMBLUCSC | 389 | 5 | 38810 | island | 0.25978 | 0.03634 | Not queried | 3.46 | 3.17e-02 | 1.00e+00 |
|  | 5 | 169532686 | 169532775 | PDFSTATSENSEMBLUCSC | 90 | 6 | 32423 | tss | 0.22349 | 0.73001 | Not queried | 3.46 | 2.16e-03 | 1.00e+00 |
|  | 1 | 68517177 | 68517273 | PDFSTATSENSEMBLUCSC | 97 | 4 | 1684 | tss;island | 0.16121 | 0.68139 | Not queried | 3.46 | 2.86e-02 | 1.00e+00 |
|  | X | 47695789 | 47696363 | PDFSTATSENSEMBLUCSC | 575 | 11 | 41757 | island | 0.03819 | 0.31500 | Not queried | 3.46 | 3.40e-05 | 1.75e-01 |
|  | X | 47695789 | 47696239 | PDFSTATSENSEMBLUCSC | 451 | 9 | 41286 | tss | 0.06467 | 0.31500 | Not queried | 3.46 | 8.23e-05 | 4.23e-01 |
|  | 11 | 6341717 | 6341908 | PDFSTATSENSEMBLUCSC | 192 | 8 | 7992 | island | 0.30693 | 0.04727 | Not queried | 3.46 | 1.55e-04 | 7.99e-01 |
|  | 1 | 155294935 | 155295267 | PDFSTATSENSEMBLUCSC | 333 | 4 | 803 | gene;tss;island | 0.25487 | 0.02885 | Not queried | 3.46 | 1.14e-01 | 1.00e+00 |
|  | 8 | 141599141 | 141599436 | PDFSTATSENSEMBLUCSC | 296 | 5 | 39380 | gene | 0.22532 | 0.82334 | Not queried | 3.46 | 7.94e-03 | 1.00e+00 |
|  | 8 | 145925708 | 145926089 | PDFSTATSENSEMBLUCSC | 382 | 5 | 40681 | island | 0.32782 | 0.04457 | Not queried | 3.46 | 7.94e-03 | 1.00e+00 |
|  | 5 | 66124395 | 66124588 | PDFSTATSENSEMBLUCSC | 194 | 5 | 32043 | tss | 0.02240 | 0.24872 | Not queried | 3.46 | 3.17e-02 | 1.00e+00 |
|  | 19 | 19383613 | 19383976 | PDFSTATSENSEMBLUCSC | 364 | 4 | 19914 | gene | 0.24905 | 0.04202 | Not queried | 3.46 | 2.86e-02 | 1.00e+00 |
|  | 1 | 26735846 | 26736154 | PDFSTATSENSEMBLUCSC | 309 | 7 | 1387 | tss;island | 0.43022 | 0.08338 | Not queried | 3.46 | 5.83e-04 | 1.00e+00 |
|  | 3 | 147128998 | 147129278 | PDFSTATSENSEMBLUCSC | 281 | 4 | 27947 | gene;island | 0.03094 | 0.33807 | Not queried | 3.46 | 2.86e-02 | 1.00e+00 |
|  | 14 | 77767644 | 77767800 | PDFSTATSENSEMBLUCSC | 157 | 4 | 12123 | gene | 0.24281 | 0.58997 | Not queried | 3.45 | 1.14e-01 | 1.00e+00 |
|  | 10 | 73136913 | 73137135 | PDFSTATSENSEMBLUCSC | 223 | 4 | 5809 | island | 0.33654 | 0.86148 | Not queried | 3.45 | 5.71e-02 | 1.00e+00 |
|  | 3 | 126007325 | 126007563 | PDFSTATSENSEMBLUCSC | 239 | 4 | 29386 | island | 0.68440 | 0.18713 | Not queried | 3.45 | 2.86e-02 | 1.00e+00 |
|  | 2 | 241389021 | 241389258 | PDFSTATSENSEMBLUCSC | 238 | 4 | 23083 | gene;island | 0.36614 | 0.80420 | Not queried | 3.45 | 3.43e-01 | 1.00e+00 |
|  | 19 | 9473565 | 9473691 | PDFSTATSENSEMBLUCSC | 127 | 6 | 20387 | tss | 0.92704 | 0.61502 | Not queried | 3.45 | 2.16e-03 | 1.00e+00 |
|  | 11 | 65816655 | 65816985 | PDFSTATSENSEMBLUCSC | 331 | 6 | 7436 | tss | 0.84637 | 0.33401 | Not queried | 3.45 | 2.16e-03 | 1.00e+00 |
|  | 11 | 2891065 | 2891118 | PDFSTATSENSEMBLUCSC | 54 | 5 | 7059 | tss;island | 0.29402 | 0.80424 | Not queried | 3.45 | 7.94e-03 | 1.00e+00 |
|  | 20 | 32856747 | 32857072 | PDFSTATSENSEMBLUCSC | 326 | 4 | 25359 | gene | 0.61666 | 0.94052 | Not queried | 3.44 | 2.86e-02 | 1.00e+00 |
|  | 8 | 24772878 | 24773268 | PDFSTATSENSEMBLUCSC | 391 | 4 | 39184 | gene;island | 0.38932 | 0.83321 | Not queried | 3.44 | 5.71e-02 | 1.00e+00 |
|  | 19 | 11529133 | 11529947 | PDFSTATSENSEMBLUCSC | 815 | 7 | 19803 | gene | 0.28811 | 0.04635 | Not queried | 3.44 | 1.28e-01 | 1.00e+00 |
|  | 14 | 106938234 | 106938621 | PDFSTATSENSEMBLUCSC | 388 | 10 | 13219 | island | 0.49415 | 0.89807 | Not queried | 3.44 | 7.58e-05 | 3.89e-01 |
|  | 1 | 32827707 | 32828191 | PDFSTATSENSEMBLUCSC | 485 | 9 | 394 | gene;tss;island | 0.04014 | 0.32549 | Not queried | 3.44 | 4.11e-05 | 2.11e-01 |
|  | 16 | 215410 | 216244 | PDFSTATSENSEMBLUCSC | 835 | 9 | 15413 | island | 0.03143 | 0.26423 | Not queried | 3.44 | 2.76e-03 | 1.00e+00 |
|  | 13 | 61989701 | 61990025 | PDFSTATSENSEMBLUCSC | 325 | 8 | 11442 | tss | 0.24253 | 0.04022 | Not queried | 3.44 | 2.95e-03 | 1.00e+00 |
|  | 2 | 25391505 | 25391911 | PDFSTATSENSEMBLUCSC | 407 | 11 | 24101 | island | 0.50087 | 0.05588 | Not queried | 3.44 | 1.98e-05 | 1.02e-01 |
|  | 13 | 113776873 | 113776964 | PDFSTATSENSEMBLUCSC | 92 | 4 | 11928 | island | 0.36943 | 0.81927 | Not queried | 3.44 | 2.86e-02 | 1.00e+00 |
|  | 22 | 19743759 | 19744078 | PDFSTATSENSEMBLUCSC | 320 | 7 | 26924 | tss | 0.25675 | 0.75550 | Not queried | 3.44 | 5.83e-04 | 1.00e+00 |
|  | 14 | 106857188 | 106857655 | PDFSTATSENSEMBLUCSC | 468 | 4 | 13218 | island | 0.49083 | 0.89650 | Not queried | 3.44 | 2.86e-02 | 1.00e+00 |
|  | 1 | 154539974 | 154540159 | PDFSTATSENSEMBLUCSC | 186 | 4 | 1963 | tss | 0.82650 | 0.34296 | Not queried | 3.44 | 2.86e-02 | 1.00e+00 |
|  | 3 | 183948058 | 183948322 | PDFSTATSENSEMBLUCSC | 265 | 6 | 29672 | island | 0.02572 | 0.15891 | Not queried | 3.43 | 1.52e-02 | 1.00e+00 |
|  | X | 153603062 | 153603268 | PDFSTATSENSEMBLUCSC | 207 | 7 | 41595 | tss | 0.05062 | 0.38745 | Not queried | 3.43 | 2.62e-02 | 1.00e+00 |
|  | 22 | 17956453 | 17956560 | PDFSTATSENSEMBLUCSC | 108 | 4 | 26902 | tss | 0.46309 | 0.90955 | Not queried | 3.43 | 2.86e-02 | 1.00e+00 |
|  | 8 | 144921531 | 144921852 | PDFSTATSENSEMBLUCSC | 322 | 4 | 39429 | gene;island | 0.37872 | 0.87115 | Not queried | 3.43 | 2.86e-02 | 1.00e+00 |
|  | 17 | 76102354 | 76102509 | PDFSTATSENSEMBLUCSC | 156 | 4 | 16778 | gene;island | 0.30377 | 0.81423 | Not queried | 3.43 | 2.00e-01 | 1.00e+00 |
|  | 1 | 17746286 | 17746597 | PDFSTATSENSEMBLUCSC | 312 | 6 | 265 | gene;island | 0.09777 | 0.65367 | Not queried | 3.43 | 9.31e-02 | 1.00e+00 |
|  | 18 | 77289084 | 77289452 | PDFSTATSENSEMBLUCSC | 369 | 4 | 19568 | island | 0.09178 | 0.52993 | Not queried | 3.43 | 2.00e-01 | 1.00e+00 |
|  | 6 | 159360705 | 159360984 | PDFSTATSENSEMBLUCSC | 280 | 4 | 36540 | island | 0.08038 | 0.58731 | Not queried | 3.43 | 1.14e-01 | 1.00e+00 |
|  | 3 | 48507354 | 48507618 | PDFSTATSENSEMBLUCSC | 265 | 6 | 27725 | gene | 0.35968 | 0.85690 | Not queried | 3.43 | 1.52e-02 | 1.00e+00 |
|  | 6 | 166666838 | 166667648 | PDFSTATSENSEMBLUCSC | 811 | 7 | 36566 | island | 0.05232 | 0.33478 | Not queried | 3.43 | 2.62e-02 | 1.00e+00 |
|  | 19 | 45720771 | 45721076 | PDFSTATSENSEMBLUCSC | 306 | 4 | 20053 | gene;island | 0.24784 | 0.76415 | Not queried | 3.42 | 5.71e-02 | 1.00e+00 |
|  | 15 | 67458370 | 67458665 | PDFSTATSENSEMBLUCSC | 296 | 4 | 13364 | gene | 0.69551 | 0.21292 | Not queried | 3.42 | 1.14e-01 | 1.00e+00 |
|  | 5 | 122434178 | 122434544 | PDFSTATSENSEMBLUCSC | 367 | 5 | 31674 | gene;island | 0.03928 | 0.26204 | Not queried | 3.42 | 3.17e-02 | 1.00e+00 |
|  | 17 | 7227499 | 7227737 | PDFSTATSENSEMBLUCSC | 239 | 4 | 16355 | gene;island | 0.36702 | 0.76421 | Not queried | 3.42 | 5.71e-02 | 1.00e+00 |
|  | 12 | 103358619 | 103358881 | PDFSTATSENSEMBLUCSC | 263 | 6 | 10702 | island | 0.03885 | 0.15042 | Not queried | 3.42 | 2.60e-02 | 1.00e+00 |
|  | 4 | 3464653 | 3465064 | PDFSTATSENSEMBLUCSC | 412 | 10 | 30790 | island | 0.44303 | 0.80568 | Not queried | 3.42 | 4.33e-02 | 1.00e+00 |
|  | 10 | 73848764 | 73849167 | PDFSTATSENSEMBLUCSC | 404 | 9 | 5817 | island | 0.27862 | 0.82957 | Not queried | 3.42 | 1.85e-03 | 1.00e+00 |
|  | 16 | 71659830 | 71660055 | PDFSTATSENSEMBLUCSC | 226 | 5 | 15317 | tss | 0.02788 | 0.20028 | Not queried | 3.42 | 9.52e-02 | 1.00e+00 |
|  | 10 | 16561202 | 16561605 | PDFSTATSENSEMBLUCSC | 404 | 5 | 4502 | gene;island | 0.83804 | 0.37014 | Not queried | 3.42 | 5.56e-02 | 1.00e+00 |
|  | 4 | 40517938 | 40518143 | PDFSTATSENSEMBLUCSC | 206 | 5 | 29942 | gene | 0.12609 | 0.57799 | Not queried | 3.41 | 7.94e-03 | 1.00e+00 |
|  | 15 | 48470606 | 48470973 | PDFSTATSENSEMBLUCSC | 368 | 6 | 13626 | tss | 0.24623 | 0.03866 | Not queried | 3.41 | 9.31e-02 | 1.00e+00 |
|  | 17 | 46695192 | 46695514 | PDFSTATSENSEMBLUCSC | 323 | 4 | 18457 | island | 0.03262 | 0.37632 | Not queried | 3.41 | 1.14e-01 | 1.00e+00 |
|  | 16 | 691444 | 691677 | PDFSTATSENSEMBLUCSC | 234 | 5 | 14940 | tss;island | 0.34668 | 0.04632 | Not queried | 3.41 | 3.17e-02 | 1.00e+00 |
|  | 6 | 73973008 | 73973128 | PDFSTATSENSEMBLUCSC | 121 | 5 | 35001 | tss | 0.24757 | 0.05621 | Not queried | 3.40 | 7.94e-03 | 1.00e+00 |
|  | 7 | 4243286 | 4243734 | PDFSTATSENSEMBLUCSC | 449 | 5 | 36771 | gene | 0.36100 | 0.89170 | Not queried | 3.40 | 7.94e-03 | 1.00e+00 |
|  | 2 | 157291759 | 157292127 | PDFSTATSENSEMBLUCSC | 369 | 6 | 23636 | tss;island | 0.37567 | 0.09479 | Not queried | 3.40 | 3.10e-01 | 1.00e+00 |
|  | 2 | 203036093 | 203036235 | PDFSTATSENSEMBLUCSC | 143 | 4 | 24978 | island | 0.29891 | 0.02516 | Not queried | 3.40 | 5.71e-02 | 1.00e+00 |
|  | 10 | 45719751 | 45720134 | PDFSTATSENSEMBLUCSC | 384 | 5 | 5693 | island | 0.31441 | 0.88327 | Not queried | 3.40 | 7.94e-03 | 1.00e+00 |
|  | 7 | 4304779 | 4305061 | PDFSTATSENSEMBLUCSC | 283 | 4 | 36775 | gene;island | 0.60874 | 0.94737 | Not queried | 3.40 | 2.86e-02 | 1.00e+00 |
|  | 6 | 134497084 | 134497755 | PDFSTATSENSEMBLUCSC | 672 | 9 | 34417 | gene;tss;island | 0.11246 | 0.05573 | Not queried | 3.40 | 6.25e-02 | 1.00e+00 |
|  | X | 70442524 | 70443093 | PDFSTATSENSEMBLUCSC | 570 | 7 | 41021 | gene;island | 0.45907 | 0.94116 | Not queried | 3.40 | 2.62e-02 | 1.00e+00 |
|  | 6 | 106433792 | 106434623 | PDFSTATSENSEMBLUCSC | 832 | 8 | 36316 | island | 0.50244 | 0.87459 | Not queried | 3.40 | 1.55e-04 | 7.99e-01 |
|  | 10 | 105212056 | 105212813 | PDFSTATSENSEMBLUCSC | 758 | 12 | 6084 | island | 0.38751 | 0.84230 | RP11-225H22.4; CALHM2; CALHM1 (margin: PDCD11; RP11-225H22.4; CALHM2; CALHM1) | 3.40 | 7.40e-07 | 3.80e-03 |
|  | 5 | 140613740 | 140613884 | PDFSTATSENSEMBLUCSC | 145 | 5 | 32309 | tss | 0.04238 | 0.29488 | Not queried | 3.39 | 7.94e-03 | 1.00e+00 |
|  | 20 | 30639816 | 30640256 | PDFSTATSENSEMBLUCSC | 441 | 10 | 26049 | island | 0.30958 | 0.03541 | Not queried | 3.39 | 3.89e-03 | 1.00e+00 |
|  | 12 | 132514292 | 132514508 | PDFSTATSENSEMBLUCSC | 217 | 5 | 10100 | tss | 0.40485 | 0.87716 | Not queried | 3.39 | 7.94e-03 | 1.00e+00 |
|  | 6 | 32123479 | 32123701 | PDFSTATSENSEMBLUCSC | 223 | 5 | 33999 | gene;island | 0.57042 | 0.95056 | Not queried | 3.39 | 9.52e-02 | 1.00e+00 |
|  | X | 68725052 | 68725088 | PDFSTATSENSEMBLUCSC | 37 | 4 | 41842 | island | 0.29844 | 0.03661 | Not queried | 3.39 | 2.86e-02 | 1.00e+00 |
|  | 21 | 34444104 | 34444382 | PDFSTATSENSEMBLUCSC | 279 | 4 | 26361 | gene;island | 0.00308 | 0.01597 | Not queried | 3.39 | 3.43e-01 | 1.00e+00 |
|  | 19 | 12946093 | 12946465 | PDFSTATSENSEMBLUCSC | 373 | 8 | 21589 | island | 0.03281 | 0.22419 | Not queried | 3.39 | 6.22e-04 | 1.00e+00 |
|  | 20 | 33413560 | 33413713 | PDFSTATSENSEMBLUCSC | 154 | 6 | 25657 | tss;island | 0.03402 | 0.29144 | Not queried | 3.39 | 2.60e-02 | 1.00e+00 |
|  | 12 | 58119915 | 58120011 | PDFSTATSENSEMBLUCSC | 97 | 5 | 9171 | gene;tss;island | 0.03468 | 0.32452 | Not queried | 3.39 | 7.94e-03 | 1.00e+00 |
|  | 1 | 220921855 | 220922217 | PDFSTATSENSEMBLUCSC | 363 | 6 | 1017 | gene | 0.33196 | 0.02550 | Not queried | 3.38 | 8.66e-03 | 1.00e+00 |
|  | 7 | 129422915 | 129423354 | PDFSTATSENSEMBLUCSC | 440 | 4 | 38849 | island | 0.86409 | 0.43330 | Not queried | 3.38 | 2.86e-02 | 1.00e+00 |
|  | 17 | 9550137 | 9550296 | PDFSTATSENSEMBLUCSC | 160 | 4 | 16401 | gene;island | 0.06320 | 0.59252 | Not queried | 3.38 | 5.71e-02 | 1.00e+00 |
|  | 17 | 75524913 | 75525151 | PDFSTATSENSEMBLUCSC | 239 | 4 | 18776 | island | 0.11068 | 0.02715 | Not queried | 3.38 | 1.14e-01 | 1.00e+00 |
|  | X | 48814914 | 48815125 | PDFSTATSENSEMBLUCSC | 212 | 6 | 41302 | tss | 0.42605 | 0.04742 | Not queried | 3.38 | 6.49e-02 | 1.00e+00 |
|  | 16 | 891260 | 891400 | PDFSTATSENSEMBLUCSC | 141 | 4 | 15462 | island | 0.44162 | 0.89696 | Not queried | 3.38 | 2.86e-02 | 1.00e+00 |
|  | 7 | 89783435 | 89783680 | PDFSTATSENSEMBLUCSC | 246 | 6 | 37630 | tss | 0.02629 | 0.23078 | Not queried | 3.37 | 1.52e-02 | 1.00e+00 |
|  | 3 | 77147070 | 77147364 | PDFSTATSENSEMBLUCSC | 295 | 5 | 27820 | gene;island | 0.50708 | 0.91024 | Not queried | 3.37 | 7.94e-03 | 1.00e+00 |
|  | 2 | 241640149 | 241640444 | PDFSTATSENSEMBLUCSC | 296 | 4 | 25240 | island | 0.48998 | 0.79592 | Not queried | 3.37 | 2.86e-02 | 1.00e+00 |
|  | 11 | 31822243 | 31822526 | PDFSTATSENSEMBLUCSC | 284 | 4 | 6539 | gene;island | 0.33649 | 0.86265 | Not queried | 3.37 | 2.00e-01 | 1.00e+00 |
|  | 10 | 129948776 | 129949058 | PDFSTATSENSEMBLUCSC | 283 | 5 | 6233 | island | 0.21173 | 0.01948 | Not queried | 3.37 | 3.17e-02 | 1.00e+00 |
|  | 4 | 186131234 | 186131285 | PDFSTATSENSEMBLUCSC | 52 | 4 | 31419 | island | 0.05387 | 0.51774 | Not queried | 3.37 | 2.86e-02 | 1.00e+00 |
|  | 5 | 114938148 | 114938459 | PDFSTATSENSEMBLUCSC | 312 | 5 | 32169 | tss | 0.37565 | 0.88478 | Not queried | 3.37 | 7.94e-03 | 1.00e+00 |
|  | 10 | 85985296 | 85985448 | PDFSTATSENSEMBLUCSC | 153 | 4 | 5176 | tss | 0.16538 | 0.63219 | Not queried | 3.37 | 2.00e-01 | 1.00e+00 |
|  | 17 | 46035183 | 46035343 | PDFSTATSENSEMBLUCSC | 161 | 4 | 17415 | tss | 0.07550 | 0.43164 | Not queried | 3.37 | 5.71e-02 | 1.00e+00 |
|  | 1 | 98515041 | 98515272 | PDFSTATSENSEMBLUCSC | 232 | 4 | 3508 | island | 0.03770 | 0.29592 | Not queried | 3.37 | 1.14e-01 | 1.00e+00 |
|  | X | 63263994 | 63264258 | PDFSTATSENSEMBLUCSC | 265 | 4 | 41826 | island | 0.17790 | 0.64416 | Not queried | 3.37 | 2.86e-02 | 1.00e+00 |
|  | 11 | 32354958 | 32355509 | PDFSTATSENSEMBLUCSC | 552 | 6 | 8153 | island | 0.19783 | 0.76284 | Not queried | 3.36 | 4.33e-03 | 1.00e+00 |
|  | 4 | 40518024 | 40518143 | PDFSTATSENSEMBLUCSC | 120 | 4 | 30311 | tss | 0.09162 | 0.56911 | Not queried | 3.36 | 2.86e-02 | 1.00e+00 |
|  | 7 | 150020751 | 150020945 | PDFSTATSENSEMBLUCSC | 195 | 7 | 37208 | gene;island | 0.37984 | 0.05624 | Not queried | 3.36 | 2.33e-03 | 1.00e+00 |
|  | 11 | 86383182 | 86383940 | PDFSTATSENSEMBLUCSC | 759 | 20 | 8688 | island | 0.10370 | 0.60214 | Not queried | 3.36 | 3.53e-03 | 1.00e+00 |
|  | 20 | 35972924 | 35973098 | PDFSTATSENSEMBLUCSC | 175 | 5 | 25687 | tss;island | 0.33009 | 0.80432 | Not queried | 3.36 | 7.94e-03 | 1.00e+00 |
|  | 2 | 133343115 | 133343373 | PDFSTATSENSEMBLUCSC | 259 | 5 | 22839 | gene;island | 0.40761 | 0.78486 | Not queried | 3.36 | 7.94e-03 | 1.00e+00 |
|  | 6 | 149772707 | 149772892 | PDFSTATSENSEMBLUCSC | 186 | 4 | 34440 | gene;island | 0.37217 | 0.82104 | Not queried | 3.36 | 2.86e-02 | 1.00e+00 |
|  | 12 | 54454163 | 54454445 | PDFSTATSENSEMBLUCSC | 283 | 5 | 9138 | gene;island | 0.91125 | 0.43001 | Not queried | 3.36 | 7.94e-03 | 1.00e+00 |
|  | 11 | 126455596 | 126456119 | PDFSTATSENSEMBLUCSC | 524 | 6 | 6943 | gene | 0.26926 | 0.68543 | Not queried | 3.35 | 1.52e-02 | 1.00e+00 |
|  | 1 | 20878760 | 20879029 | PDFSTATSENSEMBLUCSC | 270 | 5 | 2789 | island | 0.40664 | 0.05277 | Not queried | 3.35 | 1.51e-01 | 1.00e+00 |
|  | 17 | 4486934 | 4487125 | PDFSTATSENSEMBLUCSC | 192 | 5 | 16993 | tss;island | 0.35102 | 0.78991 | Not queried | 3.35 | 7.94e-03 | 1.00e+00 |
|  | 11 | 2889809 | 2890725 | PDFSTATSENSEMBLUCSC | 917 | 32 | 7058 | tss | 0.34222 | 0.86620 | KCNQ1DN (margin: KCNQ1DN; KCNQ1-AS1) | 3.35 | 5.08e-08 | 2.61e-04 |
|  | 16 | 281115 | 281424 | PDFSTATSENSEMBLUCSC | 310 | 5 | 15415 | island | 0.20633 | 0.85941 | Not queried | 3.35 | 1.51e-01 | 1.00e+00 |
|  | 3 | 65342546 | 65342971 | PDFSTATSENSEMBLUCSC | 426 | 5 | 27810 | gene;island | 0.78608 | 0.96955 | Not queried | 3.35 | 7.94e-03 | 1.00e+00 |
|  | 1 | 179050673 | 179051076 | PDFSTATSENSEMBLUCSC | 404 | 7 | 2111 | tss | 0.05343 | 0.45913 | Not queried | 3.35 | 2.62e-02 | 1.00e+00 |
|  | 2 | 45159434 | 45159663 | PDFSTATSENSEMBLUCSC | 230 | 4 | 24221 | island | 0.79454 | 0.28469 | Not queried | 3.35 | 2.86e-02 | 1.00e+00 |
|  | 1 | 901685 | 902025 | PDFSTATSENSEMBLUCSC | 341 | 6 | 2383 | island | 0.07560 | 0.43579 | Not queried | 3.35 | 2.16e-03 | 1.00e+00 |
|  | 3 | 46506404 | 46506554 | PDFSTATSENSEMBLUCSC | 151 | 6 | 28233 | tss | 0.44082 | 0.87391 | Not queried | 3.35 | 2.16e-03 | 1.00e+00 |
|  | 10 | 128193219 | 128193497 | PDFSTATSENSEMBLUCSC | 279 | 5 | 4802 | gene;island | 0.18977 | 0.70393 | Not queried | 3.34 | 1.59e-02 | 1.00e+00 |
|  | 10 | 135342218 | 135342620 | PDFSTATSENSEMBLUCSC | 403 | 4 | 4891 | gene;island | 0.75688 | 0.23343 | Not queried | 3.34 | 2.86e-02 | 1.00e+00 |
|  | 10 | 73848817 | 73849167 | PDFSTATSENSEMBLUCSC | 351 | 8 | 5129 | tss | 0.28809 | 0.80512 | Not queried | 3.34 | 4.66e-03 | 1.00e+00 |
|  | 17 | 41131966 | 41132169 | PDFSTATSENSEMBLUCSC | 204 | 5 | 16556 | gene;tss;island | 0.18491 | 0.02391 | Not queried | 3.34 | 7.94e-03 | 1.00e+00 |
|  | 6 | 17988404 | 17988960 | PDFSTATSENSEMBLUCSC | 557 | 9 | 34593 | tss;island | 0.30978 | 0.03306 | Not queried | 3.34 | 6.25e-02 | 1.00e+00 |
|  | X | 153602509 | 153603268 | PDFSTATSENSEMBLUCSC | 760 | 11 | 42136 | island | 0.05062 | 0.38745 | Not queried | 3.34 | 7.65e-04 | 1.00e+00 |
|  | 15 | 75199305 | 75199493 | PDFSTATSENSEMBLUCSC | 189 | 4 | 14292 | island | 0.04177 | 0.27109 | Not queried | 3.34 | 2.00e-01 | 1.00e+00 |
|  | 1 | 78511140 | 78511488 | PDFSTATSENSEMBLUCSC | 349 | 7 | 1707 | tss | 0.50316 | 0.07322 | Not queried | 3.34 | 5.83e-04 | 1.00e+00 |
|  | 7 | 38468944 | 38469273 | PDFSTATSENSEMBLUCSC | 330 | 5 | 36908 | gene | 0.33235 | 0.87057 | Not queried | 3.33 | 1.59e-02 | 1.00e+00 |
|  | 1 | 19600673 | 19601069 | PDFSTATSENSEMBLUCSC | 397 | 9 | 1297 | tss;island | 0.39067 | 0.86334 | Not queried | 3.33 | 4.11e-05 | 2.11e-01 |
|  | 7 | 156795767 | 156796145 | PDFSTATSENSEMBLUCSC | 379 | 5 | 39028 | island | 0.16744 | 0.04295 | Not queried | 3.33 | 1.59e-02 | 1.00e+00 |
|  | 19 | 19221316 | 19221574 | PDFSTATSENSEMBLUCSC | 259 | 4 | 19911 | gene;island | 0.24071 | 0.72848 | Not queried | 3.33 | 2.86e-02 | 1.00e+00 |
|  | 10 | 38691306 | 38691563 | PDFSTATSENSEMBLUCSC | 258 | 4 | 4548 | gene;island | 0.11157 | 0.50462 | Not queried | 3.33 | 2.86e-02 | 1.00e+00 |
|  | 1 | 214152709 | 214153036 | PDFSTATSENSEMBLUCSC | 328 | 5 | 4180 | island | 0.90043 | 0.39602 | Not queried | 3.33 | 7.94e-03 | 1.00e+00 |
|  | X | 100914892 | 100915108 | PDFSTATSENSEMBLUCSC | 217 | 4 | 41427 | tss | 0.41815 | 0.05447 | Not queried | 3.33 | 2.86e-02 | 1.00e+00 |
|  | 1 | 24239820 | 24240069 | PDFSTATSENSEMBLUCSC | 250 | 4 | 1348 | tss | 0.24570 | 0.59767 | Not queried | 3.33 | 1.14e-01 | 1.00e+00 |
|  | 14 | 91720173 | 91720696 | PDFSTATSENSEMBLUCSC | 524 | 6 | 13049 | island | 0.52555 | 0.07616 | Not queried | 3.32 | 6.49e-02 | 1.00e+00 |
|  | 16 | 29820429 | 29820592 | PDFSTATSENSEMBLUCSC | 164 | 4 | 14682 | gene;island | 0.55742 | 0.91733 | Not queried | 3.32 | 2.86e-02 | 1.00e+00 |
|  | 20 | 3713341 | 3713584 | PDFSTATSENSEMBLUCSC | 244 | 4 | 25933 | island | 0.15016 | 0.61390 | Not queried | 3.32 | 2.86e-02 | 1.00e+00 |
|  | 3 | 181444368 | 181445156 | PDFSTATSENSEMBLUCSC | 789 | 9 | 28002 | gene;island | 0.05535 | 0.33505 | Not queried | 3.32 | 7.82e-04 | 1.00e+00 |
|  | 2 | 238767990 | 238768104 | PDFSTATSENSEMBLUCSC | 115 | 4 | 23929 | tss;island | 0.10227 | 0.50778 | Not queried | 3.32 | 2.86e-02 | 1.00e+00 |
|  | 5 | 23507450 | 23507656 | PDFSTATSENSEMBLUCSC | 207 | 9 | 31942 | tss | 0.01873 | 0.13649 | Not queried | 3.31 | 7.82e-04 | 1.00e+00 |
|  | X | 68836036 | 68836202 | PDFSTATSENSEMBLUCSC | 167 | 4 | 41011 | gene | 0.24792 | 0.02590 | Not queried | 3.31 | 2.86e-02 | 1.00e+00 |
|  | 10 | 135043492 | 135043586 | PDFSTATSENSEMBLUCSC | 95 | 4 | 5436 | tss;island | 0.31810 | 0.04676 | Not queried | 3.31 | 2.86e-02 | 1.00e+00 |
|  | 2 | 71205052 | 71205564 | PDFSTATSENSEMBLUCSC | 513 | 6 | 23386 | tss | 0.41227 | 0.03503 | Not queried | 3.31 | 9.31e-02 | 1.00e+00 |
|  | X | 70273027 | 70273384 | PDFSTATSENSEMBLUCSC | 358 | 5 | 41854 | island | 0.53129 | 0.92601 | Not queried | 3.31 | 7.94e-03 | 1.00e+00 |
|  | 14 | 74185274 | 74185511 | PDFSTATSENSEMBLUCSC | 238 | 4 | 12105 | gene;island | 0.38240 | 0.85275 | Not queried | 3.31 | 5.71e-02 | 1.00e+00 |
|  | 5 | 140729429 | 140729813 | PDFSTATSENSEMBLUCSC | 385 | 7 | 32315 | tss | 0.01348 | 0.12212 | Not queried | 3.31 | 2.33e-03 | 1.00e+00 |
|  | 6 | 39016262 | 39016776 | PDFSTATSENSEMBLUCSC | 515 | 7 | 36043 | island | 0.56546 | 0.10936 | Not queried | 3.31 | 5.83e-04 | 1.00e+00 |
|  | 6 | 28601269 | 28601519 | PDFSTATSENSEMBLUCSC | 251 | 14 | 35529 | island | 0.89665 | 0.45887 | RP11-373N24.2 (margin: RP11-373N24.2) | 3.31 | 5.98e-07 | 3.07e-03 |
|  | 6 | 14211743 | 14212256 | PDFSTATSENSEMBLUCSC | 514 | 5 | 35400 | island | 0.54625 | 0.07642 | Not queried | 3.31 | 5.48e-01 | 1.00e+00 |
|  | 10 | 131323903 | 131324189 | PDFSTATSENSEMBLUCSC | 287 | 4 | 4809 | gene | 0.59463 | 0.93361 | Not queried | 3.31 | 5.71e-02 | 1.00e+00 |
|  | 15 | 96887824 | 96888024 | PDFSTATSENSEMBLUCSC | 201 | 5 | 14461 | island | 0.85707 | 0.37094 | Not queried | 3.30 | 1.51e-01 | 1.00e+00 |
|  | 3 | 147112081 | 147112605 | PDFSTATSENSEMBLUCSC | 525 | 7 | 27946 | gene;island | 0.02392 | 0.18927 | Not queried | 3.30 | 5.30e-02 | 1.00e+00 |
|  | 6 | 10881782 | 10882050 | PDFSTATSENSEMBLUCSC | 269 | 6 | 33503 | gene | 0.01316 | 0.17357 | Not queried | 3.30 | 4.11e-02 | 1.00e+00 |
|  | 21 | 45622096 | 45622493 | PDFSTATSENSEMBLUCSC | 398 | 5 | 26691 | island | 0.24483 | 0.89514 | Not queried | 3.30 | 7.94e-03 | 1.00e+00 |
|  | 5 | 140734543 | 140734758 | PDFSTATSENSEMBLUCSC | 216 | 4 | 32316 | tss | 0.25501 | 0.79603 | Not queried | 3.29 | 2.00e-01 | 1.00e+00 |
|  | 4 | 3464653 | 3465004 | PDFSTATSENSEMBLUCSC | 352 | 9 | 30215 | tss | 0.58417 | 0.76126 | Not queried | 3.29 | 9.39e-02 | 1.00e+00 |
|  | 10 | 134683711 | 134684094 | PDFSTATSENSEMBLUCSC | 384 | 5 | 6296 | island | 0.67461 | 0.95686 | Not queried | 3.29 | 7.94e-03 | 1.00e+00 |
|  | 12 | 133022423 | 133022955 | PDFSTATSENSEMBLUCSC | 533 | 6 | 11030 | island | 0.74056 | 0.95931 | Not queried | 3.29 | 2.16e-03 | 1.00e+00 |
|  | 8 | 143858490 | 143858821 | PDFSTATSENSEMBLUCSC | 332 | 9 | 39872 | tss | 0.36267 | 0.02782 | Not queried | 3.29 | 5.64e-03 | 1.00e+00 |
|  | 1 | 91190005 | 91190620 | PDFSTATSENSEMBLUCSC | 616 | 7 | 3454 | island | 0.37243 | 0.04340 | Not queried | 3.29 | 2.62e-02 | 1.00e+00 |
|  | 1 | 27286944 | 27287541 | PDFSTATSENSEMBLUCSC | 598 | 6 | 1398 | tss | 0.03070 | 0.24352 | Not queried | 3.29 | 1.32e-01 | 1.00e+00 |
|  | 17 | 45772333 | 45772523 | PDFSTATSENSEMBLUCSC | 191 | 5 | 17410 | tss | 0.29907 | 0.02320 | Not queried | 3.29 | 7.94e-03 | 1.00e+00 |
|  | 16 | 450837 | 451040 | PDFSTATSENSEMBLUCSC | 204 | 4 | 14934 | tss;island | 0.11356 | 0.55482 | Not queried | 3.29 | 2.86e-02 | 1.00e+00 |
|  | 20 | 57414884 | 57415177 | PDFSTATSENSEMBLUCSC | 294 | 4 | 25432 | gene;island | 0.59659 | 0.93141 | Not queried | 3.28 | 2.86e-02 | 1.00e+00 |
|  | 11 | 119227592 | 119227876 | PDFSTATSENSEMBLUCSC | 285 | 4 | 6919 | gene;island | 0.02974 | 0.06837 | Not queried | 3.28 | 3.43e-01 | 1.00e+00 |
|  | 1 | 45187551 | 45187946 | PDFSTATSENSEMBLUCSC | 396 | 4 | 493 | gene;island | 0.79882 | 0.34468 | Not queried | 3.28 | 2.86e-02 | 1.00e+00 |
|  | 5 | 41509849 | 41510116 | PDFSTATSENSEMBLUCSC | 268 | 5 | 31543 | gene;island | 0.11332 | 0.55366 | Not queried | 3.28 | 7.94e-03 | 1.00e+00 |
|  | 17 | 48049952 | 48049979 | PDFSTATSENSEMBLUCSC | 28 | 4 | 16642 | gene;tss;island | 0.74654 | 0.22823 | Not queried | 3.28 | 2.86e-02 | 1.00e+00 |
|  | 4 | 46392253 | 46392657 | PDFSTATSENSEMBLUCSC | 405 | 6 | 30326 | tss;island | 0.03657 | 0.22957 | Not queried | 3.28 | 1.52e-02 | 1.00e+00 |
|  | 19 | 46999055 | 46999444 | PDFSTATSENSEMBLUCSC | 390 | 10 | 22140 | island | 0.46091 | 0.07378 | Not queried | 3.28 | 1.05e-03 | 1.00e+00 |
|  | 5 | 54516779 | 54516879 | PDFSTATSENSEMBLUCSC | 101 | 4 | 32737 | island | 0.20026 | 0.03711 | Not queried | 3.28 | 2.86e-02 | 1.00e+00 |
|  | 7 | 12610355 | 12610833 | PDFSTATSENSEMBLUCSC | 479 | 6 | 36833 | gene | 0.02656 | 0.23662 | Not queried | 3.27 | 1.52e-02 | 1.00e+00 |
|  | 17 | 37784694 | 37784895 | PDFSTATSENSEMBLUCSC | 202 | 4 | 16514 | gene;island | 0.87685 | 0.29826 | Not queried | 3.27 | 2.00e-01 | 1.00e+00 |
|  | 17 | 42081810 | 42082036 | PDFSTATSENSEMBLUCSC | 227 | 8 | 17364 | tss;island | 0.42678 | 0.04131 | Not queried | 3.27 | 1.09e-03 | 1.00e+00 |
|  | 14 | 47121033 | 47121158 | PDFSTATSENSEMBLUCSC | 126 | 5 | 12347 | tss | 0.23955 | 0.85985 | Not queried | 3.27 | 7.94e-03 | 1.00e+00 |
|  | 19 | 15121204 | 15121596 | PDFSTATSENSEMBLUCSC | 393 | 9 | 21655 | island | 0.22208 | 0.63215 | Not queried | 3.27 | 1.23e-03 | 1.00e+00 |
|  | 18 | 11751343 | 11751566 | PDFSTATSENSEMBLUCSC | 224 | 6 | 19022 | gene;island | 0.37196 | 0.84830 | Not queried | 3.27 | 8.66e-03 | 1.00e+00 |
|  | 8 | 146277200 | 146277480 | PDFSTATSENSEMBLUCSC | 281 | 4 | 39935 | tss;island | 0.03021 | 0.23386 | Not queried | 3.27 | 2.86e-02 | 1.00e+00 |
|  | 16 | 850240 | 850713 | PDFSTATSENSEMBLUCSC | 474 | 6 | 14519 | gene | 0.03933 | 0.33108 | Not queried | 3.27 | 1.52e-02 | 1.00e+00 |
|  | 4 | 380157 | 380529 | PDFSTATSENSEMBLUCSC | 373 | 4 | 30669 | island | 0.64997 | 0.15105 | Not queried | 3.27 | 2.86e-02 | 1.00e+00 |
|  | 11 | 107582804 | 107582884 | PDFSTATSENSEMBLUCSC | 81 | 4 | 7642 | tss | 0.24728 | 0.79992 | Not queried | 3.27 | 3.43e-01 | 1.00e+00 |
|  | 17 | 46657274 | 46657656 | PDFSTATSENSEMBLUCSC | 383 | 5 | 18448 | island | 0.70182 | 0.24905 | Not queried | 3.26 | 9.52e-02 | 1.00e+00 |
|  | 19 | 50706387 | 50706874 | PDFSTATSENSEMBLUCSC | 488 | 7 | 20937 | tss;island | 0.03471 | 0.29067 | Not queried | 3.26 | 1.65e-01 | 1.00e+00 |
|  | 2 | 3642098 | 3642634 | PDFSTATSENSEMBLUCSC | 537 | 6 | 23125 | tss | 0.32426 | 0.85675 | Not queried | 3.26 | 1.52e-02 | 1.00e+00 |
|  | 17 | 79495268 | 79495348 | PDFSTATSENSEMBLUCSC | 81 | 5 | 17707 | tss | 0.28669 | 0.76852 | Not queried | 3.26 | 7.94e-03 | 1.00e+00 |
|  | 5 | 78281793 | 78282099 | PDFSTATSENSEMBLUCSC | 307 | 5 | 32084 | tss | 0.50758 | 0.15007 | Not queried | 3.26 | 1.59e-02 | 1.00e+00 |
|  | 11 | 2889602 | 2890725 | PDFSTATSENSEMBLUCSC | 1124 | 35 | 7955 | island | 0.33717 | 0.85799 | KCNQ1DN (margin: KCNQ1DN; KCNQ1-AS1) | 3.26 | 7.38e-08 | 3.79e-04 |
|  | 6 | 50808627 | 50808813 | PDFSTATSENSEMBLUCSC | 187 | 4 | 34301 | gene;island | 0.68594 | 0.16105 | Not queried | 3.26 | 2.86e-02 | 1.00e+00 |
|  | 1 | 150947442 | 150947589 | PDFSTATSENSEMBLUCSC | 148 | 4 | 1911 | tss | 0.23967 | 0.02871 | Not queried | 3.25 | 2.00e-01 | 1.00e+00 |
|  | 1 | 901685 | 901799 | PDFSTATSENSEMBLUCSC | 115 | 4 | 1132 | tss | 0.07798 | 0.46000 | Not queried | 3.25 | 2.86e-02 | 1.00e+00 |
|  | 1 | 6265802 | 6266099 | PDFSTATSENSEMBLUCSC | 298 | 6 | 1198 | tss;island | 0.40243 | 0.03249 | Not queried | 3.25 | 8.66e-03 | 1.00e+00 |
|  | X | 153637497 | 153637827 | PDFSTATSENSEMBLUCSC | 331 | 9 | 41157 | gene;island | 0.37282 | 0.04746 | Not queried | 3.25 | 4.11e-05 | 2.11e-01 |
|  | 4 | 190861788 | 190861959 | PDFSTATSENSEMBLUCSC | 172 | 6 | 30663 | tss | 0.02771 | 0.23560 | Not queried | 3.25 | 1.52e-02 | 1.00e+00 |
|  | 10 | 11783794 | 11784253 | PDFSTATSENSEMBLUCSC | 460 | 8 | 4929 | tss | 0.13076 | 0.63592 | Not queried | 3.25 | 6.99e-03 | 1.00e+00 |
|  | 6 | 127796989 | 127797286 | PDFSTATSENSEMBLUCSC | 298 | 4 | 34405 | gene;island | 0.39125 | 0.86454 | Not queried | 3.25 | 2.86e-02 | 1.00e+00 |
|  | 8 | 141468467 | 141468933 | PDFSTATSENSEMBLUCSC | 467 | 7 | 39861 | tss;island | 0.88016 | 0.25057 | Not queried | 3.25 | 3.79e-02 | 1.00e+00 |
|  | 6 | 31105337 | 31106302 | PDFSTATSENSEMBLUCSC | 966 | 15 | 33791 | gene | 0.29635 | 0.85954 | Not queried | 3.24 | 2.70e-03 | 1.00e+00 |
|  | 22 | 50585197 | 50585427 | PDFSTATSENSEMBLUCSC | 231 | 8 | 26883 | gene;tss | 0.13325 | 0.63293 | Not queried | 3.24 | 1.55e-04 | 7.99e-01 |
|  | 11 | 132948042 | 132948244 | PDFSTATSENSEMBLUCSC | 203 | 4 | 6964 | gene;island | 0.10641 | 0.52827 | Not queried | 3.24 | 5.71e-02 | 1.00e+00 |
|  | 3 | 42846907 | 42847116 | PDFSTATSENSEMBLUCSC | 210 | 4 | 28207 | tss;island | 0.38812 | 0.04245 | Not queried | 3.24 | 1.14e-01 | 1.00e+00 |
|  | 12 | 9800259 | 9800619 | PDFSTATSENSEMBLUCSC | 361 | 9 | 9533 | tss | 0.07606 | 0.03044 | Not queried | 3.24 | 7.77e-03 | 1.00e+00 |
|  | 4 | 113332969 | 113333269 | PDFSTATSENSEMBLUCSC | 301 | 6 | 30052 | gene;island | 0.04066 | 0.24309 | Not queried | 3.24 | 8.66e-03 | 1.00e+00 |
|  | 11 | 89224288 | 89224799 | PDFSTATSENSEMBLUCSC | 512 | 10 | 6838 | gene;island | 0.14020 | 0.02035 | Not queried | 3.24 | 1.30e-04 | 6.68e-01 |
|  | 11 | 89224684 | 89224799 | PDFSTATSENSEMBLUCSC | 116 | 6 | 7593 | tss | 0.22483 | 0.03941 | Not queried | 3.24 | 2.16e-03 | 1.00e+00 |
|  | 3 | 46734995 | 46735154 | PDFSTATSENSEMBLUCSC | 160 | 4 | 27711 | gene | 0.02312 | 0.20158 | Not queried | 3.24 | 2.86e-02 | 1.00e+00 |
|  | 10 | 127584967 | 127585144 | PDFSTATSENSEMBLUCSC | 178 | 5 | 6221 | island | 0.06693 | 0.50699 | Not queried | 3.24 | 5.56e-02 | 1.00e+00 |
|  | 10 | 97889373 | 97889448 | PDFSTATSENSEMBLUCSC | 76 | 4 | 5236 | tss | 0.82150 | 0.32621 | Not queried | 3.23 | 2.00e-01 | 1.00e+00 |
|  | 17 | 33416834 | 33417113 | PDFSTATSENSEMBLUCSC | 280 | 5 | 17231 | tss;island | 0.05203 | 0.62149 | Not queried | 3.23 | 1.51e-01 | 1.00e+00 |
|  | 16 | 28891176 | 28891926 | PDFSTATSENSEMBLUCSC | 751 | 6 | 14676 | gene;island | 0.57684 | 0.27549 | Not queried | 3.23 | 2.40e-01 | 1.00e+00 |
|  | 6 | 147124881 | 147124996 | PDFSTATSENSEMBLUCSC | 116 | 6 | 34436 | gene | 0.42393 | 0.78824 | Not queried | 3.23 | 1.32e-01 | 1.00e+00 |
|  | 5 | 7826845 | 7827133 | PDFSTATSENSEMBLUCSC | 289 | 5 | 31499 | gene | 0.29788 | 0.84269 | Not queried | 3.23 | 7.94e-03 | 1.00e+00 |
|  | X | 73513412 | 73513464 | PDFSTATSENSEMBLUCSC | 53 | 4 | 41391 | tss;island | 0.13341 | 0.37235 | Not queried | 3.23 | 5.71e-02 | 1.00e+00 |
|  | 6 | 32489553 | 32489991 | PDFSTATSENSEMBLUCSC | 439 | 6 | 34053 | gene;island | 0.15358 | 0.54148 | Not queried | 3.22 | 9.31e-02 | 1.00e+00 |
|  | 17 | 81042782 | 81043176 | PDFSTATSENSEMBLUCSC | 395 | 4 | 16942 | gene;island | 0.47242 | 0.88909 | Not queried | 3.22 | 2.86e-02 | 1.00e+00 |
|  | 3 | 142666108 | 142666476 | PDFSTATSENSEMBLUCSC | 369 | 4 | 29512 | island | 0.69044 | 0.94562 | Not queried | 3.22 | 2.86e-02 | 1.00e+00 |
|  | 11 | 63827695 | 63828346 | PDFSTATSENSEMBLUCSC | 652 | 5 | 6642 | gene | 0.81477 | 0.31593 | Not queried | 3.22 | 1.51e-01 | 1.00e+00 |
|  | 6 | 29973988 | 29975427 | PDFSTATSENSEMBLUCSC | 1440 | 47 | 33643 | gene;island | 0.16563 | 0.69279 | HLA-J; ZNRD1-AS1; HCG4P3 (margin: HLA-J; MCCD1P2; ZNRD1-AS1; HCG4P3) | 3.22 | 8.15e-23 | 4.19e-19 |
|  | 12 | 99548329 | 99548550 | PDFSTATSENSEMBLUCSC | 222 | 5 | 9237 | gene | 0.14010 | 0.58289 | Not queried | 3.22 | 7.94e-03 | 1.00e+00 |
|  | 20 | 62318426 | 62318588 | PDFSTATSENSEMBLUCSC | 163 | 4 | 25468 | gene;island | 0.43614 | 0.89482 | Not queried | 3.22 | 2.86e-02 | 1.00e+00 |
|  | 15 | 45421578 | 45422054 | PDFSTATSENSEMBLUCSC | 477 | 8 | 13298 | gene | 0.11647 | 0.52224 | Not queried | 3.22 | 4.66e-03 | 1.00e+00 |
|  | 6 | 34024220 | 34024479 | PDFSTATSENSEMBLUCSC | 260 | 4 | 34236 | gene;island | 0.13839 | 0.58637 | Not queried | 3.22 | 5.71e-02 | 1.00e+00 |
|  | 13 | 112758379 | 112758625 | PDFSTATSENSEMBLUCSC | 247 | 4 | 11885 | island | 0.24698 | 0.03652 | Not queried | 3.21 | 2.86e-02 | 1.00e+00 |
|  | 15 | 91208370 | 91208927 | PDFSTATSENSEMBLUCSC | 558 | 5 | 14428 | island | 0.63841 | 0.82838 | Not queried | 3.21 | 5.56e-02 | 1.00e+00 |
|  | 10 | 129534107 | 129534411 | PDFSTATSENSEMBLUCSC | 305 | 4 | 5417 | tss | 0.09263 | 0.47318 | Not queried | 3.21 | 1.14e-01 | 1.00e+00 |
|  | 2 | 105484357 | 105484823 | PDFSTATSENSEMBLUCSC | 467 | 4 | 24542 | island | 0.73019 | 0.25659 | Not queried | 3.21 | 2.86e-02 | 1.00e+00 |
|  | 16 | 809910 | 810292 | PDFSTATSENSEMBLUCSC | 383 | 5 | 14950 | tss | 0.36443 | 0.86269 | Not queried | 3.21 | 7.94e-03 | 1.00e+00 |
|  | 5 | 37834672 | 37835168 | PDFSTATSENSEMBLUCSC | 497 | 7 | 31534 | gene;island | 0.11056 | 0.04859 | Not queried | 3.21 | 5.83e-04 | 1.00e+00 |
|  | 5 | 37834909 | 37835168 | PDFSTATSENSEMBLUCSC | 260 | 5 | 31966 | tss | 0.19578 | 0.06467 | Not queried | 3.21 | 7.94e-03 | 1.00e+00 |
|  | X | 153637603 | 153637827 | PDFSTATSENSEMBLUCSC | 225 | 8 | 41597 | tss | 0.29747 | 0.04919 | Not queried | 3.21 | 1.55e-04 | 7.99e-01 |
|  | 2 | 176948693 | 176949017 | PDFSTATSENSEMBLUCSC | 325 | 7 | 23693 | tss | 0.04339 | 0.31878 | Not queried | 3.21 | 1.11e-02 | 1.00e+00 |
|  | 6 | 27840957 | 27841230 | PDFSTATSENSEMBLUCSC | 274 | 5 | 33560 | gene | 0.45336 | 0.90027 | Not queried | 3.21 | 7.94e-03 | 1.00e+00 |
|  | 5 | 140778342 | 140778546 | PDFSTATSENSEMBLUCSC | 205 | 4 | 31773 | gene;island | 0.63566 | 0.94164 | Not queried | 3.21 | 5.71e-02 | 1.00e+00 |
|  | 15 | 79574773 | 79575048 | PDFSTATSENSEMBLUCSC | 276 | 5 | 13815 | tss;island | 0.04042 | 0.27987 | Not queried | 3.21 | 5.56e-02 | 1.00e+00 |
|  | 15 | 42447840 | 42448259 | PDFSTATSENSEMBLUCSC | 420 | 5 | 13279 | gene | 0.47171 | 0.90671 | Not queried | 3.20 | 3.17e-02 | 1.00e+00 |
|  | 6 | 29571363 | 29571483 | PDFSTATSENSEMBLUCSC | 121 | 7 | 33601 | gene | 0.73646 | 0.94443 | Not queried | 3.20 | 4.08e-03 | 1.00e+00 |
|  | 22 | 50523790 | 50524124 | PDFSTATSENSEMBLUCSC | 335 | 4 | 27202 | tss | 0.12856 | 0.59126 | Not queried | 3.20 | 2.86e-02 | 1.00e+00 |
|  | 12 | 9217390 | 9217769 | PDFSTATSENSEMBLUCSC | 380 | 8 | 9531 | tss | 0.50364 | 0.90017 | Not queried | 3.20 | 1.55e-04 | 7.99e-01 |
|  | 19 | 58951434 | 58951885 | PDFSTATSENSEMBLUCSC | 452 | 9 | 22472 | island | 0.18437 | 0.01689 | Not queried | 3.20 | 2.88e-04 | 1.00e+00 |
|  | 11 | 134262988 | 134263349 | PDFSTATSENSEMBLUCSC | 362 | 5 | 6981 | gene | 0.43062 | 0.87427 | Not queried | 3.20 | 1.59e-02 | 1.00e+00 |
|  | Y | 21728952 | 21729314 | PDFSTATSENSEMBLUCSC | 363 | 8 | 42183 | island | 0.01285 | 0.20272 | Not queried | 3.20 | 2.34e-01 | 1.00e+00 |
|  | 4 | 1027885 | 1028067 | PDFSTATSENSEMBLUCSC | 183 | 4 | 30712 | island | 0.61013 | 0.92883 | Not queried | 3.20 | 1.14e-01 | 1.00e+00 |
|  | 2 | 223164459 | 223164925 | PDFSTATSENSEMBLUCSC | 467 | 8 | 23012 | gene;tss;island | 0.33603 | 0.04084 | Not queried | 3.19 | 1.55e-04 | 7.99e-01 |
|  | 6 | 85474028 | 85474595 | PDFSTATSENSEMBLUCSC | 568 | 10 | 35037 | tss;island | 0.01535 | 0.09425 | Not queried | 3.19 | 2.17e-05 | 1.11e-01 |
|  | 4 | 1243849 | 1244086 | PDFSTATSENSEMBLUCSC | 238 | 7 | 30193 | tss;island | 0.88673 | 0.42200 | Not queried | 3.19 | 5.83e-04 | 1.00e+00 |
|  | 6 | 30881112 | 30881842 | PDFSTATSENSEMBLUCSC | 731 | 23 | 33772 | gene | 0.07611 | 0.70181 | GTF2H4; VARS2 (margin: RN7SL175P; GTF2H4; VARS2) | 3.19 | 1.18e-06 | 6.04e-03 |
|  | 10 | 129845375 | 129846082 | PDFSTATSENSEMBLUCSC | 708 | 8 | 4806 | gene | 0.14132 | 0.61280 | Not queried | 3.19 | 2.95e-03 | 1.00e+00 |
|  | 10 | 129845375 | 129845766 | PDFSTATSENSEMBLUCSC | 392 | 6 | 5420 | tss | 0.14132 | 0.59485 | Not queried | 3.19 | 2.16e-03 | 1.00e+00 |
|  | 10 | 88718164 | 88718393 | PDFSTATSENSEMBLUCSC | 230 | 4 | 5184 | tss | 0.35672 | 0.88102 | Not queried | 3.19 | 5.71e-02 | 1.00e+00 |
|  | 2 | 220299484 | 220299900 | PDFSTATSENSEMBLUCSC | 417 | 9 | 25086 | island | 0.32963 | 0.81484 | Not queried | 3.19 | 4.11e-05 | 2.11e-01 |
|  | 2 | 220299484 | 220299672 | PDFSTATSENSEMBLUCSC | 189 | 7 | 23868 | tss | 0.32963 | 0.81484 | Not queried | 3.19 | 5.83e-04 | 1.00e+00 |
|  | 5 | 101834365 | 101834780 | PDFSTATSENSEMBLUCSC | 416 | 5 | 32924 | island | 0.22835 | 0.72909 | Not queried | 3.18 | 1.59e-02 | 1.00e+00 |
|  | 15 | 96875591 | 96875830 | PDFSTATSENSEMBLUCSC | 240 | 8 | 13464 | gene;tss;island | 0.02136 | 0.21824 | Not queried | 3.18 | 1.48e-02 | 1.00e+00 |
|  | 13 | 28503060 | 28503508 | PDFSTATSENSEMBLUCSC | 449 | 4 | 11606 | island | 0.24692 | 0.73305 | Not queried | 3.18 | 2.86e-02 | 1.00e+00 |
|  | 3 | 196366191 | 196366669 | PDFSTATSENSEMBLUCSC | 479 | 8 | 29766 | island | 0.29483 | 0.77169 | Not queried | 3.18 | 1.55e-04 | 7.99e-01 |
|  | 11 | 58672702 | 58673167 | PDFSTATSENSEMBLUCSC | 466 | 5 | 8275 | island | 0.04412 | 0.29507 | Not queried | 3.18 | 9.52e-02 | 1.00e+00 |
|  | 1 | 47915553 | 47915951 | PDFSTATSENSEMBLUCSC | 399 | 6 | 3234 | island | 0.47396 | 0.07591 | Not queried | 3.18 | 4.11e-02 | 1.00e+00 |
|  | 12 | 49726500 | 49726893 | PDFSTATSENSEMBLUCSC | 394 | 4 | 9093 | gene;island | 0.43145 | 0.84377 | Not queried | 3.18 | 2.86e-02 | 1.00e+00 |
|  | 3 | 196366191 | 196366618 | PDFSTATSENSEMBLUCSC | 428 | 7 | 28773 | tss | 0.28115 | 0.77152 | Not queried | 3.18 | 5.83e-04 | 1.00e+00 |
|  | 14 | 24801616 | 24802150 | PDFSTATSENSEMBLUCSC | 535 | 4 | 12018 | gene;island | 0.06584 | 0.47200 | Not queried | 3.18 | 2.86e-02 | 1.00e+00 |
|  | 17 | 7826155 | 7826413 | PDFSTATSENSEMBLUCSC | 259 | 5 | 16385 | gene;island | 0.02403 | 0.31929 | Not queried | 3.17 | 3.17e-02 | 1.00e+00 |
|  | 4 | 1407592 | 1407989 | PDFSTATSENSEMBLUCSC | 398 | 4 | 30737 | island | 0.36151 | 0.86053 | Not queried | 3.17 | 3.43e-01 | 1.00e+00 |
|  | 19 | 36450121 | 36450528 | PDFSTATSENSEMBLUCSC | 408 | 4 | 21894 | island | 0.04147 | 0.33172 | Not queried | 3.17 | 2.86e-02 | 1.00e+00 |
|  | 11 | 15136059 | 15136475 | PDFSTATSENSEMBLUCSC | 417 | 7 | 7149 | tss | 0.02462 | 0.25368 | Not queried | 3.17 | 1.17e-03 | 1.00e+00 |
|  | 12 | 71833017 | 71833193 | PDFSTATSENSEMBLUCSC | 177 | 4 | 9846 | tss;island | 0.02288 | 0.16491 | Not queried | 3.17 | 2.86e-02 | 1.00e+00 |
|  | 4 | 1107495 | 1107720 | PDFSTATSENSEMBLUCSC | 226 | 5 | 30719 | island | 0.32157 | 0.92019 | Not queried | 3.17 | 5.56e-02 | 1.00e+00 |
|  | 19 | 54926277 | 54926617 | PDFSTATSENSEMBLUCSC | 341 | 5 | 21025 | tss | 0.04153 | 0.28555 | Not queried | 3.17 | 5.56e-02 | 1.00e+00 |
|  | 5 | 66124395 | 66124801 | PDFSTATSENSEMBLUCSC | 407 | 7 | 31579 | gene | 0.03324 | 0.24872 | Not queried | 3.17 | 6.99e-03 | 1.00e+00 |
|  | 16 | 2198395 | 2198504 | PDFSTATSENSEMBLUCSC | 110 | 4 | 14987 | tss;island | 0.52054 | 0.03573 | Not queried | 3.16 | 5.71e-02 | 1.00e+00 |
|  | 11 | 111170390 | 111170648 | PDFSTATSENSEMBLUCSC | 259 | 5 | 6880 | gene;island | 0.44513 | 0.06316 | Not queried | 3.16 | 3.17e-02 | 1.00e+00 |
|  | 12 | 113229582 | 113230065 | PDFSTATSENSEMBLUCSC | 484 | 8 | 9291 | gene | 0.31180 | 0.69037 | Not queried | 3.16 | 6.99e-03 | 1.00e+00 |
|  | 8 | 43131260 | 43131656 | PDFSTATSENSEMBLUCSC | 397 | 5 | 40202 | island | 0.22730 | 0.76882 | Not queried | 3.16 | 1.51e-01 | 1.00e+00 |
|  | X | 70128591 | 70128670 | PDFSTATSENSEMBLUCSC | 80 | 6 | 41369 | tss | 0.58822 | 0.89065 | Not queried | 3.16 | 4.11e-02 | 1.00e+00 |
|  | 7 | 89748633 | 89748810 | PDFSTATSENSEMBLUCSC | 178 | 7 | 38580 | island | 0.06047 | 0.32246 | Not queried | 3.16 | 5.83e-04 | 1.00e+00 |
|  | 6 | 34164073 | 34164564 | PDFSTATSENSEMBLUCSC | 492 | 6 | 35990 | island | 0.07720 | 0.41967 | Not queried | 3.16 | 3.10e-01 | 1.00e+00 |
|  | 17 | 21367002 | 21367564 | PDFSTATSENSEMBLUCSC | 563 | 5 | 18089 | island | 0.33108 | 0.81519 | Not queried | 3.16 | 2.22e-01 | 1.00e+00 |
|  | 1 | 33231070 | 33231382 | PDFSTATSENSEMBLUCSC | 313 | 8 | 398 | gene | 0.68794 | 0.07372 | Not queried | 3.15 | 1.04e-02 | 1.00e+00 |
|  | 19 | 10736006 | 10736448 | PDFSTATSENSEMBLUCSC | 443 | 8 | 19782 | gene;island | 0.03415 | 0.20919 | Not queried | 3.15 | 1.55e-04 | 7.99e-01 |
|  | 5 | 140261653 | 140261810 | PDFSTATSENSEMBLUCSC | 158 | 6 | 32297 | tss | 0.05980 | 0.36902 | Not queried | 3.15 | 4.33e-03 | 1.00e+00 |
|  | 3 | 183993368 | 183993726 | PDFSTATSENSEMBLUCSC | 359 | 7 | 28714 | tss | 0.50113 | 0.85795 | Not queried | 3.15 | 9.73e-02 | 1.00e+00 |
|  | 6 | 142409417 | 142409831 | PDFSTATSENSEMBLUCSC | 415 | 5 | 34428 | gene | 0.03230 | 0.19875 | Not queried | 3.15 | 9.52e-02 | 1.00e+00 |
|  | 2 | 102090791 | 102091129 | PDFSTATSENSEMBLUCSC | 339 | 5 | 22774 | gene | 0.02832 | 0.26616 | Not queried | 3.15 | 7.94e-03 | 1.00e+00 |
|  | 3 | 69915189 | 69915449 | PDFSTATSENSEMBLUCSC | 261 | 4 | 27814 | gene | 0.49535 | 0.84383 | Not queried | 3.15 | 1.14e-01 | 1.00e+00 |
|  | 19 | 14183018 | 14183212 | PDFSTATSENSEMBLUCSC | 195 | 4 | 20500 | tss;island | 0.49613 | 0.08660 | Not queried | 3.15 | 5.71e-02 | 1.00e+00 |
|  | 4 | 90031538 | 90031969 | PDFSTATSENSEMBLUCSC | 432 | 4 | 31124 | island | 0.01608 | 0.08153 | Not queried | 3.15 | 3.43e-01 | 1.00e+00 |
|  | X | 153745603 | 153745887 | PDFSTATSENSEMBLUCSC | 285 | 4 | 41605 | tss;island | 0.31965 | 0.04226 | Not queried | 3.15 | 2.86e-02 | 1.00e+00 |
|  | 1 | 113261711 | 113261894 | PDFSTATSENSEMBLUCSC | 184 | 4 | 1837 | tss | 0.17080 | 0.55955 | Not queried | 3.14 | 1.14e-01 | 1.00e+00 |
|  | 17 | 19483367 | 19483721 | PDFSTATSENSEMBLUCSC | 355 | 5 | 18067 | island | 0.05240 | 0.30872 | Not queried | 3.14 | 7.94e-03 | 1.00e+00 |
|  | 13 | 110918331 | 110918683 | PDFSTATSENSEMBLUCSC | 353 | 4 | 11198 | gene;island | 0.59585 | 0.89665 | Not queried | 3.14 | 2.86e-02 | 1.00e+00 |
|  | 8 | 143858414 | 143858821 | PDFSTATSENSEMBLUCSC | 408 | 10 | 39405 | gene;island | 0.37182 | 0.04745 | Not queried | 3.14 | 3.89e-03 | 1.00e+00 |
|  | 2 | 65593761 | 65594021 | PDFSTATSENSEMBLUCSC | 261 | 4 | 22672 | gene | 0.78370 | 0.96659 | Not queried | 3.14 | 1.14e-01 | 1.00e+00 |
|  | 6 | 26273029 | 26273611 | PDFSTATSENSEMBLUCSC | 583 | 11 | 35471 | island | 0.57117 | 0.93176 | Not queried | 3.14 | 1.90e-04 | 9.76e-01 |
|  | 16 | 84346722 | 84346930 | PDFSTATSENSEMBLUCSC | 209 | 4 | 14837 | gene;island | 0.20125 | 0.69057 | Not queried | 3.14 | 2.86e-02 | 1.00e+00 |
|  | 7 | 150706128 | 150706355 | PDFSTATSENSEMBLUCSC | 228 | 4 | 37218 | gene;island | 0.22743 | 0.62892 | Not queried | 3.14 | 2.00e-01 | 1.00e+00 |
|  | 19 | 28296844 | 28297092 | PDFSTATSENSEMBLUCSC | 249 | 4 | 21816 | island | 0.41466 | 0.87352 | Not queried | 3.14 | 2.86e-02 | 1.00e+00 |
|  | 13 | 112895431 | 112895794 | PDFSTATSENSEMBLUCSC | 364 | 5 | 11888 | island | 0.28780 | 0.69960 | Not queried | 3.13 | 9.52e-02 | 1.00e+00 |
|  | 22 | 28198218 | 28198591 | PDFSTATSENSEMBLUCSC | 374 | 4 | 26996 | tss;island | 0.25600 | 0.03305 | Not queried | 3.13 | 2.00e-01 | 1.00e+00 |
|  | 2 | 468737 | 469214 | PDFSTATSENSEMBLUCSC | 478 | 5 | 23965 | island | 0.70802 | 0.96446 | Not queried | 3.13 | 7.94e-03 | 1.00e+00 |
|  | 8 | 496327 | 496739 | PDFSTATSENSEMBLUCSC | 413 | 5 | 39470 | tss;island | 0.53046 | 0.10830 | Not queried | 3.13 | 1.51e-01 | 1.00e+00 |
|  | 16 | 88052922 | 88053104 | PDFSTATSENSEMBLUCSC | 183 | 4 | 14867 | gene;island | 0.38900 | 0.84857 | Not queried | 3.13 | 2.86e-02 | 1.00e+00 |
|  | 16 | 4714647 | 4714815 | PDFSTATSENSEMBLUCSC | 169 | 4 | 14613 | gene | 0.48912 | 0.91510 | Not queried | 3.13 | 2.86e-02 | 1.00e+00 |
|  | 15 | 68723690 | 68724065 | PDFSTATSENSEMBLUCSC | 376 | 4 | 13367 | gene;island | 0.05050 | 0.48857 | Not queried | 3.13 | 5.71e-02 | 1.00e+00 |
|  | 16 | 71659830 | 71660113 | PDFSTATSENSEMBLUCSC | 284 | 7 | 16044 | island | 0.02788 | 0.16421 | Not queried | 3.13 | 7.28e-02 | 1.00e+00 |
|  | 19 | 46999224 | 46999444 | PDFSTATSENSEMBLUCSC | 221 | 7 | 20857 | tss | 0.44690 | 0.09184 | Not queried | 3.13 | 1.75e-02 | 1.00e+00 |
|  | X | 73834484 | 73834821 | PDFSTATSENSEMBLUCSC | 338 | 6 | 41393 | tss | 0.18983 | 0.62595 | Not queried | 3.12 | 2.16e-03 | 1.00e+00 |
|  | 2 | 200334402 | 200334584 | PDFSTATSENSEMBLUCSC | 183 | 4 | 22938 | gene;island | 0.20226 | 0.74409 | Not queried | 3.12 | 2.86e-02 | 1.00e+00 |
|  | 11 | 64108241 | 64108550 | PDFSTATSENSEMBLUCSC | 310 | 4 | 6664 | gene;island | 0.36507 | 0.06187 | Not queried | 3.12 | 2.00e-01 | 1.00e+00 |
|  | 17 | 81047627 | 81047941 | PDFSTATSENSEMBLUCSC | 315 | 4 | 16943 | gene;island | 0.38972 | 0.82782 | Not queried | 3.12 | 2.86e-02 | 1.00e+00 |
|  | 11 | 71752705 | 71752997 | PDFSTATSENSEMBLUCSC | 293 | 5 | 6790 | gene | 0.84440 | 0.31457 | Not queried | 3.12 | 5.56e-02 | 1.00e+00 |
|  | 16 | 85670135 | 85670348 | PDFSTATSENSEMBLUCSC | 214 | 4 | 14846 | gene;island | 0.67066 | 0.94372 | Not queried | 3.12 | 2.86e-02 | 1.00e+00 |
|  | 18 | 61143926 | 61143993 | PDFSTATSENSEMBLUCSC | 68 | 5 | 19278 | tss | 0.41192 | 0.85952 | Not queried | 3.12 | 7.94e-03 | 1.00e+00 |
|  | 11 | 67373625 | 67374388 | PDFSTATSENSEMBLUCSC | 764 | 9 | 8536 | island | 0.49671 | 0.07308 | Not queried | 3.12 | 9.39e-02 | 1.00e+00 |
|  | 5 | 139839 | 140289 | PDFSTATSENSEMBLUCSC | 451 | 7 | 31894 | tss | 0.69151 | 0.88364 | Not queried | 3.11 | 2.62e-02 | 1.00e+00 |
|  | 17 | 72269945 | 72270201 | PDFSTATSENSEMBLUCSC | 257 | 5 | 17589 | tss;island | 0.23247 | 0.68935 | Not queried | 3.11 | 7.94e-03 | 1.00e+00 |
|  | 19 | 49944817 | 49944964 | PDFSTATSENSEMBLUCSC | 148 | 5 | 20909 | tss | 0.03615 | 0.24260 | Not queried | 3.11 | 7.94e-03 | 1.00e+00 |
|  | 10 | 4868328 | 4868690 | PDFSTATSENSEMBLUCSC | 363 | 8 | 5498 | island | 0.09674 | 0.49439 | Not queried | 3.11 | 3.11e-04 | 1.00e+00 |
|  | 5 | 140709983 | 140710237 | PDFSTATSENSEMBLUCSC | 255 | 4 | 32313 | tss;island | 0.03449 | 0.33385 | Not queried | 3.11 | 5.71e-02 | 1.00e+00 |
|  | 3 | 50388530 | 50388670 | PDFSTATSENSEMBLUCSC | 141 | 6 | 28314 | tss | 0.14253 | 0.02454 | Not queried | 3.11 | 2.16e-03 | 1.00e+00 |
|  | 10 | 18549378 | 18549778 | PDFSTATSENSEMBLUCSC | 401 | 7 | 4508 | gene | 0.18944 | 0.70510 | Not queried | 3.11 | 1.75e-02 | 1.00e+00 |
|  | 5 | 76939333 | 76939558 | PDFSTATSENSEMBLUCSC | 226 | 4 | 32841 | island | 0.71088 | 0.13265 | Not queried | 3.11 | 2.86e-02 | 1.00e+00 |
|  | 12 | 7167584 | 7167900 | PDFSTATSENSEMBLUCSC | 317 | 4 | 9518 | tss | 0.13881 | 0.56152 | Not queried | 3.11 | 2.00e-01 | 1.00e+00 |
|  | 1 | 41350014 | 41350406 | PDFSTATSENSEMBLUCSC | 393 | 5 | 3126 | island | 0.21204 | 0.71883 | Not queried | 3.11 | 1.59e-02 | 1.00e+00 |
|  | 10 | 129533998 | 129534411 | PDFSTATSENSEMBLUCSC | 414 | 5 | 6229 | island | 0.12351 | 0.58517 | Not queried | 3.10 | 1.51e-01 | 1.00e+00 |
|  | 1 | 214153294 | 214153472 | PDFSTATSENSEMBLUCSC | 179 | 4 | 4181 | island | 0.44044 | 0.08455 | Not queried | 3.10 | 2.86e-02 | 1.00e+00 |
|  | 14 | 89028798 | 89029224 | PDFSTATSENSEMBLUCSC | 427 | 6 | 12517 | tss;island | 0.13492 | 0.13607 | Not queried | 3.10 | 5.89e-01 | 1.00e+00 |
|  | 2 | 45237689 | 45237929 | PDFSTATSENSEMBLUCSC | 241 | 4 | 23297 | tss;island | 0.41588 | 0.12915 | Not queried | 3.10 | 2.86e-02 | 1.00e+00 |
|  | X | 54556668 | 54557002 | PDFSTATSENSEMBLUCSC | 335 | 6 | 40998 | gene | 0.25452 | 0.03520 | Not queried | 3.10 | 4.33e-03 | 1.00e+00 |
|  | 12 | 91572142 | 91572276 | PDFSTATSENSEMBLUCSC | 135 | 4 | 9216 | gene | 0.10616 | 0.56251 | Not queried | 3.10 | 1.14e-01 | 1.00e+00 |
|  | 16 | 34777682 | 34778087 | PDFSTATSENSEMBLUCSC | 406 | 4 | 15861 | island | 0.19438 | 0.64787 | Not queried | 3.10 | 2.86e-02 | 1.00e+00 |
|  | 5 | 179634383 | 179634789 | PDFSTATSENSEMBLUCSC | 407 | 5 | 31882 | gene;island | 0.88779 | 0.40801 | Not queried | 3.10 | 5.56e-02 | 1.00e+00 |
|  | 2 | 176948544 | 176949017 | PDFSTATSENSEMBLUCSC | 474 | 9 | 24850 | island | 0.03922 | 0.30252 | Not queried | 3.10 | 3.99e-03 | 1.00e+00 |
|  | 19 | 11689488 | 11689726 | PDFSTATSENSEMBLUCSC | 239 | 6 | 19810 | gene | 0.61869 | 0.91956 | Not queried | 3.10 | 2.16e-03 | 1.00e+00 |
|  | 4 | 165898825 | 165898967 | PDFSTATSENSEMBLUCSC | 143 | 6 | 30618 | tss | 0.93313 | 0.59684 | Not queried | 3.09 | 2.16e-03 | 1.00e+00 |
|  | 11 | 34535085 | 34535281 | PDFSTATSENSEMBLUCSC | 197 | 4 | 6563 | gene | 0.45327 | 0.87790 | Not queried | 3.09 | 2.86e-02 | 1.00e+00 |
|  | 22 | 31198409 | 31198724 | PDFSTATSENSEMBLUCSC | 316 | 4 | 26797 | gene;island | 0.01876 | 0.11815 | Not queried | 3.09 | 5.71e-02 | 1.00e+00 |
|  | 18 | 77138860 | 77139085 | PDFSTATSENSEMBLUCSC | 226 | 4 | 19562 | island | 0.04973 | 0.34232 | Not queried | 3.09 | 1.14e-01 | 1.00e+00 |
|  | 7 | 75188876 | 75189413 | PDFSTATSENSEMBLUCSC | 538 | 7 | 36984 | gene;island | 0.44844 | 0.88188 | Not queried | 3.09 | 4.08e-03 | 1.00e+00 |
|  | 12 | 124870112 | 124870394 | PDFSTATSENSEMBLUCSC | 283 | 4 | 9367 | gene;island | 0.46321 | 0.87834 | Not queried | 3.09 | 2.86e-02 | 1.00e+00 |
|  | 1 | 153508511 | 153508875 | PDFSTATSENSEMBLUCSC | 365 | 10 | 3752 | island | 0.42961 | 0.88247 | Not queried | 3.09 | 1.08e-05 | 5.56e-02 |
|  | 19 | 49198900 | 49199217 | PDFSTATSENSEMBLUCSC | 318 | 4 | 20887 | tss | 0.04694 | 0.48801 | Not queried | 3.09 | 3.43e-01 | 1.00e+00 |
|  | 1 | 209605104 | 209605401 | PDFSTATSENSEMBLUCSC | 298 | 4 | 980 | gene;tss | 0.42451 | 0.87528 | Not queried | 3.09 | 2.86e-02 | 1.00e+00 |
|  | 14 | 106938234 | 106938451 | PDFSTATSENSEMBLUCSC | 218 | 7 | 12648 | tss | 0.50002 | 0.90370 | Not queried | 3.09 | 5.83e-04 | 1.00e+00 |
|  | 2 | 223163725 | 223163809 | PDFSTATSENSEMBLUCSC | 85 | 4 | 23878 | tss | 0.16138 | 0.02097 | Not queried | 3.09 | 2.86e-02 | 1.00e+00 |
|  | 11 | 74178684 | 74179054 | PDFSTATSENSEMBLUCSC | 371 | 7 | 7537 | tss;island | 0.39070 | 0.82375 | Not queried | 3.09 | 5.30e-02 | 1.00e+00 |
|  | 3 | 50388115 | 50388670 | PDFSTATSENSEMBLUCSC | 556 | 14 | 27766 | gene;island | 0.18697 | 0.02753 | CYB561D2; XXcos-LUCA11.5; NPRL2 (margin: ZMYND10-AS1; CYB561D2; XXcos-LUCA11.5; RASSF1; ZMYND10; NPRL2; TMEM115; CACNA2D2) | 3.08 | 4.99e-08 | 2.56e-04 |
|  | 7 | 1609341 | 1609515 | PDFSTATSENSEMBLUCSC | 175 | 4 | 36701 | gene;tss;island | 0.12882 | 0.57424 | Not queried | 3.08 | 2.86e-02 | 1.00e+00 |
|  | 7 | 19146255 | 19146555 | PDFSTATSENSEMBLUCSC | 301 | 4 | 38217 | island | 0.01725 | 0.14214 | Not queried | 3.08 | 2.86e-02 | 1.00e+00 |
|  | 1 | 25256939 | 25257191 | PDFSTATSENSEMBLUCSC | 253 | 6 | 329 | gene;tss;island | 0.29946 | 0.03834 | Not queried | 3.08 | 2.16e-03 | 1.00e+00 |
|  | 10 | 530635 | 531584 | PDFSTATSENSEMBLUCSC | 950 | 12 | 4428 | gene;island | 0.87083 | 0.50481 | Not queried | 3.08 | 2.74e-04 | 1.00e+00 |
|  | 22 | 23522307 | 23522511 | PDFSTATSENSEMBLUCSC | 205 | 7 | 26967 | tss | 0.51825 | 0.33424 | Not queried | 3.08 | 9.73e-02 | 1.00e+00 |
|  | 16 | 31227884 | 31228555 | PDFSTATSENSEMBLUCSC | 672 | 9 | 14705 | gene;island | 0.07693 | 0.33924 | Not queried | 3.08 | 4.11e-05 | 2.11e-01 |
|  | 6 | 1384491 | 1384780 | PDFSTATSENSEMBLUCSC | 290 | 4 | 35302 | island | 0.80605 | 0.43930 | Not queried | 3.08 | 2.86e-02 | 1.00e+00 |
|  | 8 | 38326358 | 38326730 | PDFSTATSENSEMBLUCSC | 373 | 6 | 39594 | tss | 0.32264 | 0.03291 | Not queried | 3.07 | 6.49e-02 | 1.00e+00 |
|  | 17 | 46657346 | 46657656 | PDFSTATSENSEMBLUCSC | 311 | 4 | 17428 | tss | 0.78548 | 0.32889 | Not queried | 3.07 | 1.14e-01 | 1.00e+00 |
|  | 7 | 5534412 | 5534721 | PDFSTATSENSEMBLUCSC | 310 | 4 | 36816 | gene;island | 0.04068 | 0.21457 | Not queried | 3.07 | 3.43e-01 | 1.00e+00 |
|  | 4 | 153601329 | 153601466 | PDFSTATSENSEMBLUCSC | 138 | 5 | 30589 | tss | 0.02481 | 0.11861 | Not queried | 3.07 | 9.52e-02 | 1.00e+00 |
|  | X | 9733868 | 9734312 | PDFSTATSENSEMBLUCSC | 445 | 5 | 41628 | island | 0.63107 | 0.93492 | Not queried | 3.07 | 2.22e-01 | 1.00e+00 |
|  | 12 | 130529058 | 130530121 | PDFSTATSENSEMBLUCSC | 1064 | 9 | 10969 | island | 0.59184 | 0.08989 | Not queried | 3.07 | 1.42e-02 | 1.00e+00 |
|  | 2 | 73298971 | 73299297 | PDFSTATSENSEMBLUCSC | 327 | 8 | 23398 | tss | 0.11563 | 0.01794 | Not queried | 3.07 | 1.95e-01 | 1.00e+00 |
|  | 5 | 37209182 | 37209440 | PDFSTATSENSEMBLUCSC | 259 | 4 | 31532 | gene;island | 0.32560 | 0.77525 | Not queried | 3.07 | 2.86e-02 | 1.00e+00 |
|  | 3 | 109128550 | 109128775 | PDFSTATSENSEMBLUCSC | 226 | 5 | 28451 | tss | 0.28107 | 0.78401 | Not queried | 3.07 | 5.56e-02 | 1.00e+00 |
|  | 8 | 108510286 | 108510343 | PDFSTATSENSEMBLUCSC | 58 | 4 | 39801 | tss | 0.20528 | 0.65131 | Not queried | 3.07 | 2.86e-02 | 1.00e+00 |
|  | X | 48754982 | 48755797 | PDFSTATSENSEMBLUCSC | 816 | 21 | 40988 | gene;tss;island | 0.24712 | 0.02750 | PQBP1; TIMM17B (margin: PQBP1; TIMM17B; SLC35A2) | 3.06 | 8.47e-08 | 4.35e-04 |
|  | 7 | 55086091 | 55086288 | PDFSTATSENSEMBLUCSC | 198 | 4 | 37538 | tss;island | 0.34282 | 0.04136 | Not queried | 3.06 | 1.14e-01 | 1.00e+00 |
|  | 12 | 130961424 | 130961986 | PDFSTATSENSEMBLUCSC | 563 | 8 | 9391 | gene | 0.41122 | 0.86601 | Not queried | 3.06 | 6.99e-03 | 1.00e+00 |
|  | X | 48534493 | 48534872 | PDFSTATSENSEMBLUCSC | 380 | 4 | 41768 | island | 0.10901 | 0.53089 | Not queried | 3.06 | 2.86e-02 | 1.00e+00 |
|  | Y | 2802900 | 2803127 | PDFSTATSENSEMBLUCSC | 228 | 7 | 42165 | tss;island | 0.08610 | 0.45100 | Not queried | 3.06 | 2.33e-03 | 1.00e+00 |
|  | 10 | 4868328 | 4868398 | PDFSTATSENSEMBLUCSC | 71 | 6 | 4904 | tss | 0.12662 | 0.51846 | Not queried | 3.06 | 2.16e-03 | 1.00e+00 |
|  | 6 | 27100831 | 27101279 | PDFSTATSENSEMBLUCSC | 449 | 5 | 33552 | gene | 0.01770 | 0.08321 | Not queried | 3.06 | 2.22e-01 | 1.00e+00 |
|  | 11 | 15136059 | 15136688 | PDFSTATSENSEMBLUCSC | 630 | 9 | 6503 | gene;island | 0.04360 | 0.25711 | Not queried | 3.06 | 3.15e-02 | 1.00e+00 |
|  | 3 | 45077649 | 45078075 | PDFSTATSENSEMBLUCSC | 427 | 6 | 29003 | island | 0.21081 | 0.73172 | Not queried | 3.05 | 1.80e-01 | 1.00e+00 |
|  | 17 | 154410 | 154671 | PDFSTATSENSEMBLUCSC | 262 | 4 | 16252 | gene;island | 0.25964 | 0.76254 | Not queried | 3.05 | 2.00e-01 | 1.00e+00 |
|  | X | 102000610 | 102001318 | PDFSTATSENSEMBLUCSC | 709 | 11 | 41056 | gene;island | 0.25503 | 0.03261 | Not queried | 3.05 | 8.33e-03 | 1.00e+00 |
|  | 12 | 114841536 | 114842031 | PDFSTATSENSEMBLUCSC | 496 | 8 | 9299 | gene;island | 0.94251 | 0.52838 | Not queried | 3.05 | 1.55e-04 | 7.99e-01 |
|  | 12 | 114841708 | 114842031 | PDFSTATSENSEMBLUCSC | 324 | 6 | 10007 | tss | 0.95191 | 0.52838 | Not queried | 3.05 | 2.16e-03 | 1.00e+00 |
|  | 4 | 1005876 | 1006278 | PDFSTATSENSEMBLUCSC | 403 | 10 | 29812 | gene;island | 0.18665 | 0.06380 | Not queried | 3.05 | 3.15e-01 | 1.00e+00 |
|  | 20 | 61147572 | 61147679 | PDFSTATSENSEMBLUCSC | 108 | 6 | 25448 | gene;island | 0.40690 | 0.85146 | Not queried | 3.05 | 4.33e-03 | 1.00e+00 |
|  | 10 | 105428385 | 105428818 | PDFSTATSENSEMBLUCSC | 434 | 5 | 4731 | gene;island | 0.51135 | 0.86897 | Not queried | 3.05 | 1.59e-02 | 1.00e+00 |
|  | 7 | 81240257 | 81240667 | PDFSTATSENSEMBLUCSC | 411 | 5 | 38559 | island | 0.03184 | 0.17875 | Not queried | 3.05 | 3.17e-02 | 1.00e+00 |
|  | 10 | 13748985 | 13749625 | PDFSTATSENSEMBLUCSC | 641 | 9 | 4491 | gene | 0.02059 | 0.19719 | Not queried | 3.05 | 2.44e-02 | 1.00e+00 |
|  | 6 | 72130539 | 72130799 | PDFSTATSENSEMBLUCSC | 261 | 9 | 34997 | tss | 0.02278 | 0.16642 | Not queried | 3.05 | 5.64e-03 | 1.00e+00 |
|  | 2 | 56412924 | 56413253 | PDFSTATSENSEMBLUCSC | 330 | 5 | 22655 | gene;island | 0.05890 | 0.34060 | Not queried | 3.04 | 2.22e-01 | 1.00e+00 |
|  | 10 | 64578739 | 64579140 | PDFSTATSENSEMBLUCSC | 402 | 5 | 5758 | island | 0.32802 | 0.04857 | Not queried | 3.04 | 7.94e-03 | 1.00e+00 |
|  | 8 | 125985352 | 125985512 | PDFSTATSENSEMBLUCSC | 161 | 4 | 39844 | tss | 0.46781 | 0.89546 | Not queried | 3.04 | 2.86e-02 | 1.00e+00 |
|  | 6 | 106546704 | 106546754 | PDFSTATSENSEMBLUCSC | 51 | 4 | 35084 | tss | 0.02474 | 0.11761 | Not queried | 3.04 | 2.86e-02 | 1.00e+00 |
|  | 4 | 9782891 | 9783398 | PDFSTATSENSEMBLUCSC | 508 | 10 | 30874 | island | 0.37467 | 0.89993 | Not queried | 3.04 | 2.88e-03 | 1.00e+00 |
|  | 19 | 12175107 | 12175719 | PDFSTATSENSEMBLUCSC | 613 | 10 | 21555 | island | 0.05102 | 0.37859 | Not queried | 3.04 | 3.55e-02 | 1.00e+00 |
|  | 5 | 140562562 | 140563400 | PDFSTATSENSEMBLUCSC | 839 | 8 | 31756 | gene;island | 0.52252 | 0.87881 | Not queried | 3.04 | 1.55e-04 | 7.99e-01 |
|  | 3 | 50359849 | 50360176 | PDFSTATSENSEMBLUCSC | 328 | 6 | 27762 | gene;tss;island | 0.56465 | 0.23813 | Not queried | 3.04 | 4.33e-03 | 1.00e+00 |
|  | 5 | 134783075 | 134783273 | PDFSTATSENSEMBLUCSC | 199 | 4 | 32245 | tss | 0.86167 | 0.46503 | Not queried | 3.04 | 2.86e-02 | 1.00e+00 |
|  | 5 | 41870746 | 41871189 | PDFSTATSENSEMBLUCSC | 444 | 10 | 32693 | island | 0.05293 | 0.36127 | Not queried | 3.04 | 2.32e-02 | 1.00e+00 |
|  | 17 | 79797038 | 79797373 | PDFSTATSENSEMBLUCSC | 336 | 5 | 18908 | island | 0.31693 | 0.84953 | Not queried | 3.04 | 3.17e-02 | 1.00e+00 |
|  | 20 | 61147572 | 61147652 | PDFSTATSENSEMBLUCSC | 81 | 5 | 25834 | tss | 0.41225 | 0.85186 | Not queried | 3.04 | 7.94e-03 | 1.00e+00 |
|  | 16 | 9058197 | 9058365 | PDFSTATSENSEMBLUCSC | 169 | 4 | 15053 | tss;island | 0.41717 | 0.06652 | Not queried | 3.03 | 2.00e-01 | 1.00e+00 |
|  | 20 | 22567724 | 22568116 | PDFSTATSENSEMBLUCSC | 393 | 4 | 26006 | island | 0.12214 | 0.56741 | Not queried | 3.03 | 3.43e-01 | 1.00e+00 |
|  | 10 | 99734416 | 99735202 | PDFSTATSENSEMBLUCSC | 787 | 8 | 4688 | gene | 0.25779 | 0.73027 | Not queried | 3.03 | 3.11e-04 | 1.00e+00 |
|  | 4 | 24796689 | 24797007 | PDFSTATSENSEMBLUCSC | 319 | 5 | 30280 | tss | 0.14151 | 0.61556 | Not queried | 3.03 | 6.90e-01 | 1.00e+00 |
|  | 12 | 12938082 | 12938425 | PDFSTATSENSEMBLUCSC | 344 | 6 | 9035 | gene;tss;island | 0.73015 | 0.31762 | Not queried | 3.03 | 2.16e-03 | 1.00e+00 |
|  | 3 | 113251061 | 113251417 | PDFSTATSENSEMBLUCSC | 357 | 5 | 29323 | island | 0.00594 | 0.03448 | Not queried | 3.03 | 4.21e-01 | 1.00e+00 |
|  | 10 | 105212236 | 105212813 | PDFSTATSENSEMBLUCSC | 578 | 11 | 5321 | tss | 0.46931 | 0.84486 | RP11-225H22.4; CALHM2; CALHM1 (margin: PDCD11; RP11-225H22.4; CALHM2; CALHM1) | 3.03 | 2.84e-06 | 1.46e-02 |
|  | X | 48931699 | 48932122 | PDFSTATSENSEMBLUCSC | 424 | 9 | 41306 | tss | 0.20012 | 0.02953 | Not queried | 3.03 | 8.23e-05 | 4.23e-01 |
|  | 17 | 38334161 | 38334186 | PDFSTATSENSEMBLUCSC | 26 | 5 | 17293 | tss;island | 0.23034 | 0.02681 | Not queried | 3.03 | 7.94e-03 | 1.00e+00 |
|  | 6 | 117802401 | 117802786 | PDFSTATSENSEMBLUCSC | 386 | 6 | 35132 | tss;island | 0.37697 | 0.08241 | Not queried | 3.03 | 8.66e-03 | 1.00e+00 |
|  | 15 | 41803428 | 41803850 | PDFSTATSENSEMBLUCSC | 423 | 4 | 13275 | gene;island | 0.58595 | 0.12018 | Not queried | 3.03 | 2.86e-02 | 1.00e+00 |
|  | 5 | 140261653 | 140261930 | PDFSTATSENSEMBLUCSC | 278 | 7 | 31746 | gene;island | 0.06114 | 0.43869 | Not queried | 3.03 | 1.75e-02 | 1.00e+00 |
|  | 18 | 11751343 | 11751556 | PDFSTATSENSEMBLUCSC | 214 | 5 | 19150 | tss | 0.37276 | 0.82868 | Not queried | 3.02 | 3.17e-02 | 1.00e+00 |
|  | 15 | 65648103 | 65648490 | PDFSTATSENSEMBLUCSC | 388 | 4 | 13350 | gene;island | 0.23337 | 0.03469 | Not queried | 3.02 | 2.00e-01 | 1.00e+00 |
|  | 2 | 26785120 | 26785460 | PDFSTATSENSEMBLUCSC | 341 | 8 | 23206 | tss | 0.48658 | 0.86936 | Not queried | 3.02 | 1.55e-04 | 7.99e-01 |
|  | 7 | 54956202 | 54956598 | PDFSTATSENSEMBLUCSC | 397 | 5 | 38437 | island | 0.61365 | 0.21752 | Not queried | 3.02 | 3.17e-02 | 1.00e+00 |
|  | 22 | 42469976 | 42470223 | PDFSTATSENSEMBLUCSC | 248 | 6 | 27145 | tss;island | 0.46554 | 0.89234 | Not queried | 3.02 | 2.16e-03 | 1.00e+00 |
|  | 2 | 242710665 | 242711046 | PDFSTATSENSEMBLUCSC | 382 | 5 | 25266 | island | 0.50661 | 0.88863 | Not queried | 3.02 | 5.56e-02 | 1.00e+00 |
|  | 19 | 58951599 | 58951885 | PDFSTATSENSEMBLUCSC | 287 | 8 | 21106 | tss | 0.22831 | 0.02388 | Not queried | 3.02 | 1.09e-03 | 1.00e+00 |
|  | 20 | 982991 | 983130 | PDFSTATSENSEMBLUCSC | 140 | 4 | 25492 | tss | 0.08009 | 0.39378 | Not queried | 3.01 | 1.14e-01 | 1.00e+00 |
|  | 5 | 169532686 | 169533120 | PDFSTATSENSEMBLUCSC | 435 | 8 | 33312 | island | 0.30183 | 0.73001 | Not queried | 3.01 | 4.66e-03 | 1.00e+00 |
|  | 17 | 43222106 | 43222258 | PDFSTATSENSEMBLUCSC | 153 | 4 | 18392 | island | 0.25781 | 0.73587 | Not queried | 3.01 | 2.86e-02 | 1.00e+00 |
|  | 15 | 57998711 | 57998919 | PDFSTATSENSEMBLUCSC | 209 | 6 | 13336 | gene;island | 0.30019 | 0.02696 | Not queried | 3.01 | 5.89e-01 | 1.00e+00 |
|  | X | 118699184 | 118699375 | PDFSTATSENSEMBLUCSC | 192 | 4 | 41091 | gene | 0.01810 | 0.12166 | Not queried | 3.01 | 3.43e-01 | 1.00e+00 |
|  | 11 | 2883529 | 2883912 | PDFSTATSENSEMBLUCSC | 384 | 4 | 7953 | island | 0.53963 | 0.21852 | Not queried | 3.01 | 1.14e-01 | 1.00e+00 |
|  | 19 | 12167119 | 12167313 | PDFSTATSENSEMBLUCSC | 195 | 4 | 21554 | island | 0.33506 | 0.75300 | Not queried | 3.01 | 2.00e-01 | 1.00e+00 |
|  | 20 | 37230326 | 37230741 | PDFSTATSENSEMBLUCSC | 416 | 7 | 26118 | island | 0.05411 | 0.29874 | Not queried | 3.00 | 5.83e-04 | 1.00e+00 |
|  | 17 | 80708279 | 80708632 | PDFSTATSENSEMBLUCSC | 354 | 4 | 16923 | gene;island | 0.93816 | 0.67332 | Not queried | 3.00 | 2.86e-02 | 1.00e+00 |
|  | 2 | 71693090 | 71694016 | PDFSTATSENSEMBLUCSC | 927 | 12 | 22694 | gene;island | 0.34590 | 0.08934 | Not queried | 3.00 | 4.96e-04 | 1.00e+00 |
|  | 4 | 139481751 | 139482017 | PDFSTATSENSEMBLUCSC | 267 | 4 | 31255 | island | 0.28044 | 0.95218 | Not queried | 3.00 | 2.00e-01 | 1.00e+00 |
|  | 9 | 38622294 | 38622748 | PDFSTATSENSEMBLUCSC | 455 | 5 | 40701 | gene;island | 0.09317 | 0.43584 | Not queried | 3.00 | 1.51e-01 | 1.00e+00 |
|  | 17 | 77903985 | 77904396 | PDFSTATSENSEMBLUCSC | 412 | 5 | 18813 | island | 0.06447 | 0.36849 | Not queried | 3.00 | 7.94e-03 | 1.00e+00 |
|  | 13 | 20735819 | 20736342 | PDFSTATSENSEMBLUCSC | 524 | 8 | 11300 | tss;island | 0.47913 | 0.06514 | Not queried | 3.00 | 1.04e-02 | 1.00e+00 |
|  | 10 | 135039948 | 135040250 | PDFSTATSENSEMBLUCSC | 303 | 4 | 6327 | island | 0.49938 | 0.88339 | Not queried | 3.00 | 2.86e-02 | 1.00e+00 |
|  | 3 | 49395923 | 49395991 | PDFSTATSENSEMBLUCSC | 69 | 4 | 28285 | tss | 0.84740 | 0.33922 | Not queried | 3.00 | 2.86e-02 | 1.00e+00 |
|  | 2 | 43327937 | 43328657 | PDFSTATSENSEMBLUCSC | 721 | 7 | 24209 | island | 0.18874 | 0.04802 | Not queried | 3.00 | 5.30e-02 | 1.00e+00 |
|  | 10 | 102976327 | 102977342 | PDFSTATSENSEMBLUCSC | 1016 | 11 | 6038 | island | 0.06850 | 0.34439 | Not queried | 3.00 | 7.65e-04 | 1.00e+00 |
|  | 17 | 33787402 | 33787739 | PDFSTATSENSEMBLUCSC | 338 | 4 | 18182 | island | 0.17826 | 0.57058 | Not queried | 2.99 | 5.71e-02 | 1.00e+00 |
|  | 2 | 223916502 | 223916820 | PDFSTATSENSEMBLUCSC | 319 | 5 | 23883 | tss | 0.51255 | 0.93262 | Not queried | 2.99 | 7.94e-03 | 1.00e+00 |
|  | 19 | 46379763 | 46380134 | PDFSTATSENSEMBLUCSC | 372 | 4 | 22125 | island | 0.26307 | 0.70536 | Not queried | 2.99 | 2.86e-02 | 1.00e+00 |
|  | 19 | 1826707 | 1826957 | PDFSTATSENSEMBLUCSC | 251 | 4 | 19630 | gene;island | 0.53280 | 0.87474 | Not queried | 2.99 | 2.86e-02 | 1.00e+00 |
|  | 19 | 46181221 | 46181546 | PDFSTATSENSEMBLUCSC | 326 | 5 | 20069 | gene;island | 0.06128 | 0.35621 | Not queried | 2.99 | 1.59e-02 | 1.00e+00 |
|  | X | 135230594 | 135230841 | PDFSTATSENSEMBLUCSC | 248 | 5 | 41118 | gene;island | 0.39518 | 0.07596 | Not queried | 2.99 | 3.17e-02 | 1.00e+00 |
|  | 10 | 135150367 | 135150517 | PDFSTATSENSEMBLUCSC | 151 | 6 | 6339 | island | 0.05108 | 0.34669 | Not queried | 2.99 | 4.33e-03 | 1.00e+00 |
|  | X | 102000610 | 102000905 | PDFSTATSENSEMBLUCSC | 296 | 7 | 41434 | tss | 0.25718 | 0.04132 | Not queried | 2.99 | 2.09e-01 | 1.00e+00 |
|  | 7 | 158936508 | 158936739 | PDFSTATSENSEMBLUCSC | 232 | 4 | 37320 | gene;island | 0.14351 | 0.01751 | Not queried | 2.99 | 3.43e-01 | 1.00e+00 |
|  | 6 | 146350041 | 146350748 | PDFSTATSENSEMBLUCSC | 708 | 8 | 34435 | gene;island | 0.26888 | 0.72036 | Not queried | 2.99 | 1.04e-02 | 1.00e+00 |
|  | 11 | 10673844 | 10674182 | PDFSTATSENSEMBLUCSC | 339 | 4 | 6491 | gene | 0.45080 | 0.84231 | Not queried | 2.99 | 2.86e-02 | 1.00e+00 |
|  | 17 | 77997663 | 77997997 | PDFSTATSENSEMBLUCSC | 335 | 5 | 16816 | gene;island | 0.60166 | 0.94371 | Not queried | 2.99 | 5.56e-02 | 1.00e+00 |
|  | 1 | 147737016 | 147737302 | PDFSTATSENSEMBLUCSC | 287 | 5 | 3690 | island | 0.21764 | 0.02807 | Not queried | 2.98 | 7.94e-03 | 1.00e+00 |
|  | 12 | 48399140 | 48399493 | PDFSTATSENSEMBLUCSC | 354 | 5 | 9641 | tss;island | 0.85467 | 0.52211 | Not queried | 2.98 | 1.59e-02 | 1.00e+00 |
|  | 17 | 56736571 | 56736918 | PDFSTATSENSEMBLUCSC | 348 | 5 | 16675 | gene;island | 0.40805 | 0.08016 | Not queried | 2.98 | 7.94e-03 | 1.00e+00 |
|  | 16 | 89114613 | 89114919 | PDFSTATSENSEMBLUCSC | 307 | 5 | 16200 | island | 0.25828 | 0.72893 | Not queried | 2.98 | 7.94e-03 | 1.00e+00 |
|  | 6 | 3752754 | 3753083 | PDFSTATSENSEMBLUCSC | 330 | 4 | 34536 | tss;island | 0.61627 | 0.18342 | Not queried | 2.98 | 2.86e-02 | 1.00e+00 |
|  | 5 | 140797172 | 140797286 | PDFSTATSENSEMBLUCSC | 115 | 4 | 31776 | gene;island | 0.18622 | 0.62754 | Not queried | 2.98 | 2.86e-02 | 1.00e+00 |
|  | 19 | 19383613 | 19384165 | PDFSTATSENSEMBLUCSC | 553 | 5 | 21775 | island | 0.22937 | 0.03830 | Not queried | 2.98 | 3.17e-02 | 1.00e+00 |
|  | 8 | 142236917 | 142237359 | PDFSTATSENSEMBLUCSC | 443 | 5 | 39388 | gene;island | 0.50159 | 0.87568 | Not queried | 2.98 | 7.94e-03 | 1.00e+00 |
|  | 16 | 52581405 | 52581746 | PDFSTATSENSEMBLUCSC | 342 | 5 | 15202 | tss;island | 0.20817 | 0.04508 | Not queried | 2.98 | 3.17e-02 | 1.00e+00 |
|  | 10 | 14014419 | 14014651 | PDFSTATSENSEMBLUCSC | 233 | 5 | 4493 | gene | 0.46718 | 0.89980 | Not queried | 2.98 | 9.52e-02 | 1.00e+00 |
|  | 16 | 88803803 | 88804051 | PDFSTATSENSEMBLUCSC | 249 | 4 | 14883 | gene;island | 0.02339 | 0.31355 | Not queried | 2.98 | 5.71e-02 | 1.00e+00 |
|  | 17 | 72889708 | 72890001 | PDFSTATSENSEMBLUCSC | 294 | 8 | 17601 | tss | 0.07101 | 0.32841 | Not queried | 2.97 | 1.55e-04 | 7.99e-01 |
|  | X | 64754701 | 64754995 | PDFSTATSENSEMBLUCSC | 295 | 10 | 41352 | tss | 0.10025 | 0.02670 | Not queried | 2.97 | 4.87e-04 | 1.00e+00 |
|  | 10 | 97889373 | 97889705 | PDFSTATSENSEMBLUCSC | 333 | 6 | 5953 | island | 0.55832 | 0.20331 | Not queried | 2.97 | 1.80e-01 | 1.00e+00 |
|  | 12 | 124941781 | 124942248 | PDFSTATSENSEMBLUCSC | 468 | 5 | 9371 | gene;island | 0.63002 | 0.91845 | Not queried | 2.97 | 7.94e-03 | 1.00e+00 |
|  | 19 | 10736006 | 10736117 | PDFSTATSENSEMBLUCSC | 112 | 5 | 20419 | tss | 0.03223 | 0.19128 | Not queried | 2.97 | 7.94e-03 | 1.00e+00 |
|  | 4 | 3486039 | 3486380 | PDFSTATSENSEMBLUCSC | 342 | 4 | 29863 | gene;island | 0.43860 | 0.79008 | Not queried | 2.97 | 2.86e-02 | 1.00e+00 |
|  | 5 | 101834365 | 101834675 | PDFSTATSENSEMBLUCSC | 311 | 4 | 31646 | gene | 0.23419 | 0.74742 | Not queried | 2.97 | 2.86e-02 | 1.00e+00 |
|  | 10 | 106399877 | 106400824 | PDFSTATSENSEMBLUCSC | 948 | 10 | 5333 | tss | 0.05861 | 0.30550 | Not queried | 2.97 | 1.08e-05 | 5.56e-02 |
|  | 10 | 1517083 | 1517340 | PDFSTATSENSEMBLUCSC | 258 | 5 | 4454 | gene;island | 0.11012 | 0.50012 | Not queried | 2.97 | 9.52e-02 | 1.00e+00 |
|  | 11 | 62559638 | 62559882 | PDFSTATSENSEMBLUCSC | 245 | 5 | 6629 | gene | 0.08880 | 0.42805 | Not queried | 2.97 | 9.52e-02 | 1.00e+00 |
|  | 17 | 75180961 | 75181373 | PDFSTATSENSEMBLUCSC | 413 | 4 | 16766 | gene | 0.34375 | 0.79088 | Not queried | 2.97 | 5.71e-02 | 1.00e+00 |
|  | 15 | 42371511 | 42371967 | PDFSTATSENSEMBLUCSC | 457 | 7 | 13278 | gene;island | 0.13195 | 0.55652 | Not queried | 2.96 | 5.83e-04 | 1.00e+00 |
|  | 10 | 100227419 | 100227782 | PDFSTATSENSEMBLUCSC | 364 | 4 | 4690 | gene;island | 0.01505 | 0.14241 | Not queried | 2.96 | 3.43e-01 | 1.00e+00 |
|  | 11 | 1902923 | 1903333 | PDFSTATSENSEMBLUCSC | 411 | 4 | 6418 | gene;island | 0.33440 | 0.71032 | Not queried | 2.96 | 2.00e-01 | 1.00e+00 |
|  | 4 | 183063397 | 183063637 | PDFSTATSENSEMBLUCSC | 241 | 4 | 30143 | gene;island | 0.60285 | 0.15083 | Not queried | 2.96 | 5.71e-02 | 1.00e+00 |
|  | 10 | 106399877 | 106400880 | PDFSTATSENSEMBLUCSC | 1004 | 11 | 6101 | island | 0.05418 | 0.26667 | SORCS3 (margin: SORCS3) | 2.96 | 5.67e-06 | 2.91e-02 |
|  | 7 | 1083072 | 1083473 | PDFSTATSENSEMBLUCSC | 402 | 4 | 36678 | gene;island | 0.61021 | 0.91816 | Not queried | 2.96 | 2.86e-02 | 1.00e+00 |
|  | 13 | 27333803 | 27334259 | PDFSTATSENSEMBLUCSC | 457 | 4 | 11085 | gene;island | 0.04702 | 0.36447 | Not queried | 2.96 | 2.86e-02 | 1.00e+00 |
|  | 22 | 27068716 | 27068761 | PDFSTATSENSEMBLUCSC | 46 | 4 | 26786 | gene | 0.05538 | 0.00722 | Not queried | 2.96 | 2.86e-02 | 1.00e+00 |
|  | 7 | 100199952 | 100200009 | PDFSTATSENSEMBLUCSC | 58 | 4 | 37081 | gene | 0.91682 | 0.63387 | Not queried | 2.95 | 2.86e-02 | 1.00e+00 |
|  | 13 | 113832355 | 113832595 | PDFSTATSENSEMBLUCSC | 241 | 4 | 11251 | gene | 0.62939 | 0.94695 | Not queried | 2.95 | 5.71e-02 | 1.00e+00 |
|  | 12 | 7033470 | 7033563 | PDFSTATSENSEMBLUCSC | 94 | 4 | 9510 | tss | 0.03532 | 0.09014 | Not queried | 2.95 | 3.43e-01 | 1.00e+00 |
|  | 11 | 65779192 | 65779343 | PDFSTATSENSEMBLUCSC | 152 | 6 | 7435 | tss | 0.25397 | 0.68469 | Not queried | 2.95 | 2.16e-03 | 1.00e+00 |
|  | 10 | 135150485 | 135150517 | PDFSTATSENSEMBLUCSC | 33 | 4 | 5443 | tss | 0.05108 | 0.30469 | Not queried | 2.95 | 5.71e-02 | 1.00e+00 |
|  | 5 | 139135602 | 139135961 | PDFSTATSENSEMBLUCSC | 360 | 4 | 33116 | island | 0.07511 | 0.38956 | Not queried | 2.95 | 5.71e-02 | 1.00e+00 |
|  | 20 | 57471654 | 57471844 | PDFSTATSENSEMBLUCSC | 191 | 5 | 25438 | gene | 0.68934 | 0.94203 | Not queried | 2.95 | 1.59e-02 | 1.00e+00 |
|  | 3 | 81810999 | 81811448 | PDFSTATSENSEMBLUCSC | 450 | 6 | 28406 | tss | 0.24867 | 0.03597 | Not queried | 2.95 | 4.11e-02 | 1.00e+00 |
|  | 6 | 31685392 | 31685575 | PDFSTATSENSEMBLUCSC | 184 | 7 | 33877 | gene | 0.11494 | 0.49746 | Not queried | 2.95 | 5.83e-04 | 1.00e+00 |
|  | X | 70288026 | 70288225 | PDFSTATSENSEMBLUCSC | 200 | 4 | 41019 | gene | 0.04226 | 0.00963 | Not queried | 2.95 | 1.14e-01 | 1.00e+00 |
|  | 19 | 15121204 | 15121531 | PDFSTATSENSEMBLUCSC | 328 | 7 | 20516 | tss | 0.25359 | 0.70801 | Not queried | 2.94 | 4.08e-03 | 1.00e+00 |
|  | 1 | 15250209 | 15250388 | PDFSTATSENSEMBLUCSC | 180 | 4 | 238 | gene;tss;island | 0.59108 | 0.18364 | Not queried | 2.94 | 2.00e-01 | 1.00e+00 |
|  | 14 | 60715218 | 60715614 | PDFSTATSENSEMBLUCSC | 397 | 4 | 12074 | gene;tss;island | 0.18187 | 0.03807 | Not queried | 2.94 | 5.71e-02 | 1.00e+00 |
|  | 2 | 176973688 | 176974060 | PDFSTATSENSEMBLUCSC | 373 | 5 | 22898 | gene;island | 0.75769 | 0.19634 | Not queried | 2.93 | 7.94e-03 | 1.00e+00 |
|  | 6 | 106546704 | 106546824 | PDFSTATSENSEMBLUCSC | 121 | 5 | 34369 | gene | 0.02896 | 0.13803 | Not queried | 2.93 | 7.94e-03 | 1.00e+00 |
|  | 1 | 155043322 | 155043967 | PDFSTATSENSEMBLUCSC | 646 | 7 | 3795 | island | 0.04838 | 0.28717 | Not queried | 2.93 | 2.09e-01 | 1.00e+00 |
|  | 22 | 37640314 | 37640514 | PDFSTATSENSEMBLUCSC | 201 | 5 | 27073 | tss | 0.06787 | 0.35746 | Not queried | 2.93 | 7.94e-03 | 1.00e+00 |
|  | 1 | 228246632 | 228247135 | PDFSTATSENSEMBLUCSC | 504 | 4 | 1043 | gene;island | 0.20196 | 0.63046 | Not queried | 2.93 | 5.71e-02 | 1.00e+00 |
|  | 10 | 81075012 | 81075241 | PDFSTATSENSEMBLUCSC | 230 | 4 | 4635 | gene | 0.59432 | 0.89943 | Not queried | 2.93 | 2.86e-02 | 1.00e+00 |
|  | 13 | 50570018 | 50570250 | PDFSTATSENSEMBLUCSC | 233 | 4 | 11139 | gene;tss;island | 0.57376 | 0.14484 | Not queried | 2.93 | 2.86e-02 | 1.00e+00 |
|  | 3 | 350503 | 351003 | PDFSTATSENSEMBLUCSC | 501 | 6 | 27595 | gene;island | 0.07001 | 0.47864 | Not queried | 2.93 | 9.31e-02 | 1.00e+00 |
|  | X | 54209594 | 54209715 | PDFSTATSENSEMBLUCSC | 122 | 4 | 41813 | island | 0.17925 | 0.02232 | Not queried | 2.93 | 1.14e-01 | 1.00e+00 |
|  | 4 | 186732837 | 186733060 | PDFSTATSENSEMBLUCSC | 224 | 7 | 30165 | gene;tss | 0.02680 | 0.35192 | Not queried | 2.93 | 4.08e-03 | 1.00e+00 |
|  | 1 | 45278971 | 45279349 | PDFSTATSENSEMBLUCSC | 379 | 4 | 497 | gene;island | 0.38139 | 0.09424 | Not queried | 2.93 | 1.14e-01 | 1.00e+00 |
|  | 11 | 32452038 | 32452839 | PDFSTATSENSEMBLUCSC | 802 | 8 | 6548 | gene;island | 0.81944 | 0.33938 | Not queried | 2.92 | 6.22e-04 | 1.00e+00 |
|  | 2 | 27851409 | 27852231 | PDFSTATSENSEMBLUCSC | 823 | 16 | 22595 | gene;tss | 0.42328 | 0.06330 | ZNF512; RP11-158I13.2; GPN1; CCDC121 (margin: ZNF512; RP11-158I13.2; GPN1; CCDC121) | 2.92 | 3.04e-06 | 1.56e-02 |
|  | 1 | 38230769 | 38230887 | PDFSTATSENSEMBLUCSC | 119 | 6 | 3067 | island | 0.09610 | 0.51455 | Not queried | 2.92 | 2.16e-03 | 1.00e+00 |
|  | 2 | 42719503 | 42719933 | PDFSTATSENSEMBLUCSC | 431 | 4 | 22618 | gene;island | 0.80495 | 0.33350 | Not queried | 2.92 | 2.86e-02 | 1.00e+00 |
|  | 17 | 46691820 | 46692248 | PDFSTATSENSEMBLUCSC | 429 | 5 | 16625 | gene | 0.03940 | 0.25985 | Not queried | 2.92 | 7.94e-03 | 1.00e+00 |
|  | 5 | 87898489 | 87898688 | PDFSTATSENSEMBLUCSC | 200 | 4 | 31617 | gene;island | 0.13180 | 0.47794 | Not queried | 2.92 | 2.00e-01 | 1.00e+00 |
|  | 1 | 29062689 | 29063076 | PDFSTATSENSEMBLUCSC | 388 | 7 | 1433 | tss;island | 0.07939 | 0.01387 | Not queried | 2.92 | 1.75e-02 | 1.00e+00 |
|  | 11 | 6291339 | 6291625 | PDFSTATSENSEMBLUCSC | 287 | 4 | 6470 | gene;island | 0.36549 | 0.79476 | Not queried | 2.92 | 5.71e-02 | 1.00e+00 |
|  | 1 | 152487844 | 152488368 | PDFSTATSENSEMBLUCSC | 525 | 8 | 756 | gene;island | 0.02565 | 0.29190 | Not queried | 2.91 | 1.48e-02 | 1.00e+00 |
|  | X | 49687028 | 49687331 | PDFSTATSENSEMBLUCSC | 304 | 10 | 41794 | island | 0.11070 | 0.01540 | Not queried | 2.91 | 1.05e-03 | 1.00e+00 |
|  | 6 | 53412803 | 53413189 | PDFSTATSENSEMBLUCSC | 387 | 5 | 36153 | island | 0.02109 | 0.13960 | Not queried | 2.91 | 5.56e-02 | 1.00e+00 |
|  | 18 | 904963 | 905611 | PDFSTATSENSEMBLUCSC | 649 | 10 | 19000 | gene | 0.07955 | 0.41910 | Not queried | 2.91 | 2.17e-05 | 1.11e-01 |
|  | 2 | 132152814 | 132152960 | PDFSTATSENSEMBLUCSC | 147 | 4 | 24693 | island | 0.09925 | 0.44826 | Not queried | 2.91 | 2.00e-01 | 1.00e+00 |
|  | 15 | 29407792 | 29408145 | PDFSTATSENSEMBLUCSC | 354 | 4 | 13239 | gene;island | 0.39598 | 0.80915 | Not queried | 2.91 | 2.86e-02 | 1.00e+00 |
|  | 18 | 54305957 | 54306437 | PDFSTATSENSEMBLUCSC | 481 | 10 | 19254 | tss | 0.02469 | 0.13424 | Not queried | 2.91 | 8.93e-03 | 1.00e+00 |
|  | 2 | 55450577 | 55450874 | PDFSTATSENSEMBLUCSC | 298 | 4 | 22649 | gene;island | 0.01502 | 0.15236 | Not queried | 2.91 | 2.86e-02 | 1.00e+00 |
|  | 2 | 109746691 | 109746754 | PDFSTATSENSEMBLUCSC | 64 | 4 | 22786 | gene;tss;island | 0.88949 | 0.47661 | Not queried | 2.91 | 2.00e-01 | 1.00e+00 |
|  | X | 48693397 | 48693665 | PDFSTATSENSEMBLUCSC | 269 | 4 | 40986 | gene;island | 0.49067 | 0.18566 | Not queried | 2.91 | 2.86e-02 | 1.00e+00 |
|  | 6 | 29795815 | 29796124 | PDFSTATSENSEMBLUCSC | 310 | 9 | 33633 | gene;island | 0.02873 | 0.18162 | Not queried | 2.91 | 9.39e-02 | 1.00e+00 |
|  | 19 | 33182526 | 33182834 | PDFSTATSENSEMBLUCSC | 309 | 7 | 20643 | tss;island | 0.02750 | 0.24477 | Not queried | 2.91 | 6.99e-03 | 1.00e+00 |
|  | 3 | 149374761 | 149375293 | PDFSTATSENSEMBLUCSC | 533 | 7 | 27950 | gene;island | 0.16515 | 0.02439 | Not queried | 2.91 | 3.79e-02 | 1.00e+00 |
|  | 1 | 174968745 | 174968881 | PDFSTATSENSEMBLUCSC | 137 | 4 | 2099 | tss | 0.20461 | 0.02912 | Not queried | 2.91 | 1.14e-01 | 1.00e+00 |
|  | 3 | 46735315 | 46735454 | PDFSTATSENSEMBLUCSC | 140 | 5 | 28236 | tss | 0.07266 | 0.38832 | Not queried | 2.90 | 9.52e-02 | 1.00e+00 |
|  | 3 | 46734995 | 46735454 | PDFSTATSENSEMBLUCSC | 460 | 9 | 29014 | island | 0.05103 | 0.27711 | Not queried | 2.90 | 3.99e-03 | 1.00e+00 |
|  | 6 | 110798012 | 110798323 | PDFSTATSENSEMBLUCSC | 312 | 5 | 35106 | tss;island | 0.55913 | 0.93601 | Not queried | 2.90 | 5.56e-02 | 1.00e+00 |
|  | 1 | 11898405 | 11898805 | PDFSTATSENSEMBLUCSC | 401 | 5 | 227 | gene;island | 0.16528 | 0.59704 | Not queried | 2.90 | 5.56e-02 | 1.00e+00 |
|  | X | 153046175 | 153046894 | PDFSTATSENSEMBLUCSC | 720 | 9 | 42120 | island | 0.34260 | 0.85861 | Not queried | 2.90 | 7.77e-03 | 1.00e+00 |
|  | 17 | 27346732 | 27347260 | PDFSTATSENSEMBLUCSC | 529 | 6 | 18131 | island | 0.46390 | 0.86009 | Not queried | 2.90 | 8.66e-03 | 1.00e+00 |
|  | 6 | 33091111 | 33092243 | PDFSTATSENSEMBLUCSC | 1133 | 20 | 34125 | gene | 0.94655 | 0.60787 | Not queried | 2.90 | 1.77e-03 | 1.00e+00 |
|  | 2 | 127963489 | 127963653 | PDFSTATSENSEMBLUCSC | 165 | 5 | 23572 | tss | 0.64478 | 0.26848 | Not queried | 2.90 | 7.94e-03 | 1.00e+00 |
|  | 2 | 202484020 | 202484320 | PDFSTATSENSEMBLUCSC | 301 | 6 | 23781 | tss | 0.19298 | 0.71730 | Not queried | 2.90 | 2.16e-03 | 1.00e+00 |
|  | 1 | 23763081 | 23763959 | PDFSTATSENSEMBLUCSC | 879 | 7 | 313 | gene;island | 0.16158 | 0.76055 | Not queried | 2.90 | 2.62e-02 | 1.00e+00 |
|  | 6 | 10881782 | 10882336 | PDFSTATSENSEMBLUCSC | 555 | 11 | 35375 | island | 0.01716 | 0.05746 | Not queried | 2.90 | 5.24e-03 | 1.00e+00 |
|  | 13 | 113540400 | 113540631 | PDFSTATSENSEMBLUCSC | 232 | 5 | 11233 | gene;island | 0.44275 | 0.85569 | Not queried | 2.90 | 7.94e-03 | 1.00e+00 |
|  | 1 | 222763258 | 222763513 | PDFSTATSENSEMBLUCSC | 256 | 8 | 2262 | tss | 0.23235 | 0.03762 | Not queried | 2.90 | 3.11e-04 | 1.00e+00 |
|  | 2 | 227700458 | 227700568 | PDFSTATSENSEMBLUCSC | 111 | 6 | 23890 | tss;island | 0.23505 | 0.05419 | Not queried | 2.90 | 2.16e-03 | 1.00e+00 |
|  | 18 | 49867117 | 49867488 | PDFSTATSENSEMBLUCSC | 372 | 5 | 19082 | gene;island | 0.09268 | 0.36575 | Not queried | 2.90 | 5.56e-02 | 1.00e+00 |
|  | 1 | 92012408 | 92012736 | PDFSTATSENSEMBLUCSC | 329 | 5 | 3468 | island | 0.39780 | 0.79074 | Not queried | 2.90 | 7.94e-03 | 1.00e+00 |
|  | 8 | 144897555 | 144897576 | PDFSTATSENSEMBLUCSC | 22 | 5 | 39897 | tss | 0.47068 | 0.10672 | Not queried | 2.90 | 7.94e-03 | 1.00e+00 |
|  | 8 | 144897297 | 144897576 | PDFSTATSENSEMBLUCSC | 280 | 7 | 40625 | island | 0.47947 | 0.10672 | Not queried | 2.90 | 5.83e-04 | 1.00e+00 |
|  | 16 | 31548640 | 31548908 | PDFSTATSENSEMBLUCSC | 269 | 4 | 15849 | island | 0.90562 | 0.50586 | Not queried | 2.90 | 5.71e-02 | 1.00e+00 |
|  | 11 | 77185354 | 77185670 | PDFSTATSENSEMBLUCSC | 317 | 4 | 7563 | tss;island | 0.04261 | 0.30557 | Not queried | 2.89 | 5.71e-02 | 1.00e+00 |
|  | 15 | 101817896 | 101818008 | PDFSTATSENSEMBLUCSC | 113 | 5 | 13905 | tss;island | 0.01232 | 0.02375 | Not queried | 2.89 | 2.22e-01 | 1.00e+00 |
|  | 15 | 79383385 | 79383980 | PDFSTATSENSEMBLUCSC | 596 | 14 | 13814 | tss;island | 0.02511 | 0.18780 | RASGRF1 (margin: RASGRF1) | 2.89 | 6.93e-06 | 3.56e-02 |
|  | 7 | 127672458 | 127672658 | PDFSTATSENSEMBLUCSC | 201 | 4 | 37148 | gene;island | 0.18099 | 0.03295 | Not queried | 2.89 | 2.86e-02 | 1.00e+00 |
|  | 12 | 20094141 | 20094385 | PDFSTATSENSEMBLUCSC | 245 | 4 | 10249 | island | 0.59124 | 0.92862 | Not queried | 2.89 | 5.71e-02 | 1.00e+00 |
|  | 6 | 29869838 | 29870062 | PDFSTATSENSEMBLUCSC | 225 | 5 | 33638 | gene | 0.80605 | 0.47011 | Not queried | 2.89 | 3.17e-02 | 1.00e+00 |
|  | 17 | 72889323 | 72890001 | PDFSTATSENSEMBLUCSC | 679 | 11 | 18693 | island | 0.08564 | 0.34967 | Not queried | 2.89 | 1.28e-04 | 6.56e-01 |
|  | 6 | 29711249 | 29711588 | PDFSTATSENSEMBLUCSC | 340 | 4 | 33627 | gene | 0.69869 | 0.26416 | Not queried | 2.89 | 2.86e-02 | 1.00e+00 |
|  | 6 | 108444791 | 108444920 | PDFSTATSENSEMBLUCSC | 130 | 4 | 36330 | island | 0.85913 | 0.58373 | Not queried | 2.89 | 2.86e-02 | 1.00e+00 |
|  | 7 | 96627602 | 96627821 | PDFSTATSENSEMBLUCSC | 220 | 4 | 37037 | gene;island | 0.35452 | 0.08058 | Not queried | 2.89 | 2.86e-02 | 1.00e+00 |
|  | 3 | 46506104 | 46506554 | PDFSTATSENSEMBLUCSC | 451 | 10 | 29011 | island | 0.55056 | 0.87391 | Not queried | 2.89 | 1.08e-05 | 5.56e-02 |
|  | 2 | 19558368 | 19558569 | PDFSTATSENSEMBLUCSC | 202 | 5 | 24064 | island | 0.27815 | 0.04258 | Not queried | 2.89 | 1.59e-02 | 1.00e+00 |
|  | 20 | 42544648 | 42544794 | PDFSTATSENSEMBLUCSC | 147 | 8 | 25384 | gene;island | 0.24481 | 0.01828 | Not queried | 2.89 | 2.07e-02 | 1.00e+00 |
|  | 1 | 67600172 | 67600835 | PDFSTATSENSEMBLUCSC | 664 | 8 | 3357 | island | 0.11250 | 0.52672 | Not queried | 2.89 | 2.07e-02 | 1.00e+00 |
|  | 12 | 54321992 | 54322396 | PDFSTATSENSEMBLUCSC | 405 | 4 | 10449 | island | 0.04327 | 0.20766 | Not queried | 2.89 | 2.00e-01 | 1.00e+00 |
|  | 4 | 57522493 | 57523055 | PDFSTATSENSEMBLUCSC | 563 | 9 | 29965 | gene;island | 0.59810 | 0.14641 | Not queried | 2.89 | 1.88e-02 | 1.00e+00 |
|  | 5 | 180542154 | 180542623 | PDFSTATSENSEMBLUCSC | 470 | 8 | 33451 | island | 0.19909 | 0.54697 | Not queried | 2.89 | 1.48e-02 | 1.00e+00 |
|  | 11 | 61519687 | 61520084 | PDFSTATSENSEMBLUCSC | 398 | 5 | 7316 | tss | 0.22110 | 0.03701 | Not queried | 2.88 | 5.48e-01 | 1.00e+00 |
|  | 6 | 30139421 | 30140417 | PDFSTATSENSEMBLUCSC | 997 | 26 | 33676 | gene;island | 0.33140 | 0.78489 | TRIM15 (margin: TRIM15; TRIM10; TRIM26) | 2.88 | 4.73e-11 | 2.43e-07 |
|  | 19 | 2543602 | 2543877 | PDFSTATSENSEMBLUCSC | 276 | 5 | 19656 | gene;island | 0.03591 | 0.11502 | Not queried | 2.88 | 5.56e-02 | 1.00e+00 |
|  | X | 134124839 | 134125259 | PDFSTATSENSEMBLUCSC | 421 | 9 | 42045 | island | 0.10769 | 0.61884 | Not queried | 2.88 | 2.88e-04 | 1.00e+00 |
|  | 15 | 101629063 | 101629375 | PDFSTATSENSEMBLUCSC | 313 | 4 | 14486 | island | 0.12414 | 0.51067 | Not queried | 2.88 | 2.00e-01 | 1.00e+00 |
|  | 22 | 21369411 | 21369777 | PDFSTATSENSEMBLUCSC | 367 | 4 | 26949 | tss;island | 0.68551 | 0.29482 | Not queried | 2.88 | 2.00e-01 | 1.00e+00 |
|  | 2 | 74875227 | 74875548 | PDFSTATSENSEMBLUCSC | 322 | 8 | 23427 | tss;island | 0.34925 | 0.78496 | Not queried | 2.88 | 1.55e-04 | 7.99e-01 |
|  | 2 | 242702553 | 242702932 | PDFSTATSENSEMBLUCSC | 380 | 4 | 23107 | gene | 0.70259 | 0.94079 | Not queried | 2.88 | 2.00e-01 | 1.00e+00 |
|  | 3 | 184972080 | 184972153 | PDFSTATSENSEMBLUCSC | 74 | 4 | 28723 | tss;island | 0.16035 | 0.60478 | Not queried | 2.88 | 2.86e-02 | 1.00e+00 |
|  | 20 | 32856747 | 32857227 | PDFSTATSENSEMBLUCSC | 481 | 6 | 26067 | island | 0.61666 | 0.93531 | Not queried | 2.88 | 2.16e-03 | 1.00e+00 |
|  | 11 | 67373625 | 67374213 | PDFSTATSENSEMBLUCSC | 589 | 8 | 7477 | tss | 0.60138 | 0.22630 | Not queried | 2.88 | 1.30e-01 | 1.00e+00 |
|  | 17 | 4545074 | 4545157 | PDFSTATSENSEMBLUCSC | 84 | 4 | 16994 | tss | 0.61187 | 0.15466 | Not queried | 2.88 | 5.71e-02 | 1.00e+00 |
|  | 1 | 78511140 | 78512129 | PDFSTATSENSEMBLUCSC | 990 | 12 | 3395 | island | 0.27574 | 0.06497 | Not queried | 2.88 | 6.81e-03 | 1.00e+00 |
|  | 8 | 143859143 | 143859410 | PDFSTATSENSEMBLUCSC | 268 | 5 | 39406 | gene;tss;island | 0.31852 | 0.05979 | Not queried | 2.88 | 3.17e-02 | 1.00e+00 |
|  | 12 | 72233240 | 72233472 | PDFSTATSENSEMBLUCSC | 233 | 8 | 9850 | tss | 0.25059 | 0.08339 | Not queried | 2.88 | 6.50e-02 | 1.00e+00 |
|  | X | 38662916 | 38663177 | PDFSTATSENSEMBLUCSC | 262 | 8 | 40964 | gene;island | 0.23678 | 0.03855 | Not queried | 2.88 | 4.99e-02 | 1.00e+00 |
|  | X | 38662916 | 38663062 | PDFSTATSENSEMBLUCSC | 147 | 6 | 41260 | tss | 0.25249 | 0.04662 | Not queried | 2.88 | 9.31e-02 | 1.00e+00 |
|  | 1 | 16345293 | 16345491 | PDFSTATSENSEMBLUCSC | 199 | 4 | 1275 | tss | 0.26782 | 0.67698 | Not queried | 2.88 | 2.86e-02 | 1.00e+00 |
|  | 2 | 118942841 | 118943184 | PDFSTATSENSEMBLUCSC | 344 | 5 | 24603 | island | 0.05412 | 0.25947 | Not queried | 2.87 | 7.94e-03 | 1.00e+00 |
|  | 12 | 56109593 | 56109799 | PDFSTATSENSEMBLUCSC | 207 | 5 | 9743 | tss | 0.72552 | 0.11976 | Not queried | 2.87 | 3.10e-01 | 1.00e+00 |
|  | 6 | 50692150 | 50692762 | PDFSTATSENSEMBLUCSC | 613 | 6 | 34299 | gene;island | 0.14594 | 0.55700 | Not queried | 2.87 | 6.49e-02 | 1.00e+00 |
|  | X | 153602509 | 153602918 | PDFSTATSENSEMBLUCSC | 410 | 4 | 41155 | gene | 0.04816 | 0.29811 | Not queried | 2.87 | 5.71e-02 | 1.00e+00 |
|  | 3 | 118663470 | 118663697 | PDFSTATSENSEMBLUCSC | 228 | 4 | 27861 | gene | 0.43276 | 0.88758 | Not queried | 2.87 | 1.14e-01 | 1.00e+00 |
|  | 6 | 108488751 | 108489064 | PDFSTATSENSEMBLUCSC | 314 | 4 | 34373 | gene;island | 0.42937 | 0.08144 | Not queried | 2.87 | 2.86e-02 | 1.00e+00 |
|  | 2 | 87568958 | 87569447 | PDFSTATSENSEMBLUCSC | 490 | 4 | 24462 | island | 0.29709 | 0.66673 | Not queried | 2.87 | 1.14e-01 | 1.00e+00 |
|  | 22 | 31317764 | 31318240 | PDFSTATSENSEMBLUCSC | 477 | 8 | 27028 | tss | 0.58726 | 0.84752 | Not queried | 2.87 | 2.07e-02 | 1.00e+00 |
|  | 5 | 180527369 | 180527697 | PDFSTATSENSEMBLUCSC | 329 | 4 | 33450 | island | 0.05877 | 0.41852 | Not queried | 2.87 | 2.86e-02 | 1.00e+00 |
|  | 15 | 25199028 | 25199270 | PDFSTATSENSEMBLUCSC | 243 | 4 | 13228 | gene;tss;island | 0.76759 | 0.20804 | Not queried | 2.87 | 1.14e-01 | 1.00e+00 |
|  | 17 | 7348316 | 7348399 | PDFSTATSENSEMBLUCSC | 84 | 6 | 17051 | tss | 0.18265 | 0.02925 | Not queried | 2.87 | 8.66e-03 | 1.00e+00 |
|  | 17 | 7348316 | 7348490 | PDFSTATSENSEMBLUCSC | 175 | 8 | 17917 | island | 0.18265 | 0.01910 | Not queried | 2.87 | 2.95e-03 | 1.00e+00 |
|  | 14 | 21769105 | 21769379 | PDFSTATSENSEMBLUCSC | 275 | 5 | 11983 | gene;island | 0.14219 | 0.61835 | Not queried | 2.87 | 3.17e-02 | 1.00e+00 |
|  | 3 | 36902435 | 36902745 | PDFSTATSENSEMBLUCSC | 311 | 4 | 27673 | gene | 0.88943 | 0.54699 | Not queried | 2.86 | 5.71e-02 | 1.00e+00 |
|  | 7 | 99067116 | 99067368 | PDFSTATSENSEMBLUCSC | 253 | 5 | 38669 | island | 0.23151 | 0.03836 | Not queried | 2.86 | 7.94e-03 | 1.00e+00 |
|  | 1 | 27286832 | 27287541 | PDFSTATSENSEMBLUCSC | 710 | 7 | 2901 | island | 0.01821 | 0.06936 | Not queried | 2.86 | 1.28e-01 | 1.00e+00 |
|  | 7 | 45927735 | 45928326 | PDFSTATSENSEMBLUCSC | 592 | 8 | 38413 | island | 0.20082 | 0.69311 | Not queried | 2.86 | 4.66e-03 | 1.00e+00 |
|  | 5 | 176543790 | 176544135 | PDFSTATSENSEMBLUCSC | 346 | 5 | 33377 | island | 0.55525 | 0.89006 | Not queried | 2.86 | 7.94e-03 | 1.00e+00 |
|  | 1 | 220921216 | 220922217 | PDFSTATSENSEMBLUCSC | 1002 | 14 | 4206 | island | 0.07899 | 0.01698 | Not queried | 2.86 | 1.18e-03 | 1.00e+00 |
|  | 11 | 108422663 | 108423325 | PDFSTATSENSEMBLUCSC | 663 | 8 | 6871 | gene | 0.41066 | 0.78887 | Not queried | 2.86 | 6.99e-03 | 1.00e+00 |
|  | 6 | 167104601 | 167104926 | PDFSTATSENSEMBLUCSC | 326 | 5 | 34488 | gene | 0.20700 | 0.65403 | Not queried | 2.86 | 3.10e-01 | 1.00e+00 |
|  | 2 | 71205052 | 71205685 | PDFSTATSENSEMBLUCSC | 634 | 7 | 24353 | island | 0.57256 | 0.04777 | Not queried | 2.85 | 5.30e-02 | 1.00e+00 |
|  | 1 | 999599 | 999888 | PDFSTATSENSEMBLUCSC | 290 | 5 | 2394 | island | 0.05527 | 0.35797 | Not queried | 2.85 | 2.22e-01 | 1.00e+00 |
|  | X | 48776465 | 48776812 | PDFSTATSENSEMBLUCSC | 348 | 7 | 41301 | tss | 0.12728 | 0.01978 | Not queried | 2.85 | 2.62e-02 | 1.00e+00 |
|  | 7 | 55224743 | 55225183 | PDFSTATSENSEMBLUCSC | 441 | 7 | 36955 | gene | 0.76632 | 0.38718 | Not queried | 2.85 | 6.99e-03 | 1.00e+00 |
|  | 2 | 28114016 | 28114462 | PDFSTATSENSEMBLUCSC | 447 | 8 | 22599 | gene;tss;island | 0.05426 | 0.35665 | Not queried | 2.85 | 4.99e-02 | 1.00e+00 |
|  | 19 | 3097565 | 3097728 | PDFSTATSENSEMBLUCSC | 164 | 4 | 19669 | gene;island | 0.65140 | 0.94154 | Not queried | 2.85 | 2.86e-02 | 1.00e+00 |
|  | 22 | 40766469 | 40766683 | PDFSTATSENSEMBLUCSC | 215 | 9 | 27469 | island | 0.02881 | 0.14278 | Not queried | 2.85 | 1.65e-04 | 8.46e-01 |
|  | 7 | 814391 | 814759 | PDFSTATSENSEMBLUCSC | 369 | 5 | 36660 | gene;island | 0.61667 | 0.93002 | Not queried | 2.85 | 2.22e-01 | 1.00e+00 |
|  | 17 | 72306008 | 72306215 | PDFSTATSENSEMBLUCSC | 208 | 4 | 16714 | gene | 0.14778 | 0.55386 | Not queried | 2.85 | 2.86e-02 | 1.00e+00 |
|  | 21 | 46706579 | 46707012 | PDFSTATSENSEMBLUCSC | 434 | 4 | 26424 | gene;tss;island | 0.40656 | 0.09029 | Not queried | 2.85 | 4.86e-01 | 1.00e+00 |
|  | 17 | 33734481 | 33734847 | PDFSTATSENSEMBLUCSC | 367 | 4 | 18180 | island | 0.87128 | 0.40149 | Not queried | 2.85 | 5.71e-02 | 1.00e+00 |
|  | 19 | 50831762 | 50832037 | PDFSTATSENSEMBLUCSC | 276 | 5 | 20136 | gene;island | 0.05679 | 0.21471 | Not queried | 2.85 | 1.51e-01 | 1.00e+00 |
|  | 19 | 12175107 | 12175540 | PDFSTATSENSEMBLUCSC | 434 | 7 | 20452 | tss | 0.04795 | 0.36617 | Not queried | 2.85 | 2.59e-01 | 1.00e+00 |
|  | 3 | 119187426 | 119187686 | PDFSTATSENSEMBLUCSC | 261 | 4 | 28478 | tss;island | 0.63805 | 0.22983 | Not queried | 2.84 | 2.00e-01 | 1.00e+00 |
|  | X | 68348465 | 68348839 | PDFSTATSENSEMBLUCSC | 375 | 6 | 41839 | island | 0.43324 | 0.85785 | Not queried | 2.84 | 2.16e-03 | 1.00e+00 |
|  | 17 | 76100911 | 76101391 | PDFSTATSENSEMBLUCSC | 481 | 5 | 16777 | gene;island | 0.62919 | 0.79580 | Not queried | 2.84 | 1.51e-01 | 1.00e+00 |
|  | 4 | 111397134 | 111397220 | PDFSTATSENSEMBLUCSC | 87 | 5 | 30500 | tss | 0.40633 | 0.89301 | Not queried | 2.84 | 3.17e-02 | 1.00e+00 |
|  | 12 | 46767665 | 46768016 | PDFSTATSENSEMBLUCSC | 352 | 6 | 9633 | tss;island | 0.31130 | 0.03452 | Not queried | 2.84 | 8.66e-03 | 1.00e+00 |
|  | 15 | 45406327 | 45406939 | PDFSTATSENSEMBLUCSC | 613 | 13 | 13617 | tss;island | 0.12429 | 0.56339 | Not queried | 2.84 | 2.97e-04 | 1.00e+00 |
|  | 19 | 46932069 | 46932308 | PDFSTATSENSEMBLUCSC | 240 | 4 | 22137 | island | 0.26495 | 0.04342 | Not queried | 2.84 | 5.71e-02 | 1.00e+00 |
|  | 20 | 20433062 | 20433328 | PDFSTATSENSEMBLUCSC | 267 | 4 | 25335 | gene;island | 0.02994 | 0.14958 | Not queried | 2.84 | 1.14e-01 | 1.00e+00 |
|  | 14 | 63568602 | 63568809 | PDFSTATSENSEMBLUCSC | 208 | 4 | 12419 | tss | 0.89675 | 0.58669 | Not queried | 2.83 | 2.86e-02 | 1.00e+00 |
|  | 10 | 127584967 | 127585090 | PDFSTATSENSEMBLUCSC | 124 | 4 | 5414 | tss | 0.05914 | 0.48903 | Not queried | 2.83 | 1.14e-01 | 1.00e+00 |
|  | 1 | 117753591 | 117753741 | PDFSTATSENSEMBLUCSC | 151 | 4 | 1866 | tss | 0.28531 | 0.72948 | Not queried | 2.83 | 2.86e-02 | 1.00e+00 |
|  | 22 | 17680477 | 17680822 | PDFSTATSENSEMBLUCSC | 346 | 9 | 26739 | gene | 0.24197 | 0.04015 | Not queried | 2.83 | 2.44e-02 | 1.00e+00 |
|  | 22 | 17680545 | 17680822 | PDFSTATSENSEMBLUCSC | 278 | 7 | 26901 | tss | 0.26968 | 0.05422 | Not queried | 2.83 | 1.75e-02 | 1.00e+00 |
|  | 6 | 32005786 | 32006079 | PDFSTATSENSEMBLUCSC | 294 | 4 | 34796 | tss | 0.56494 | 0.10336 | Not queried | 2.83 | 5.71e-02 | 1.00e+00 |
|  | 3 | 9811714 | 9811990 | PDFSTATSENSEMBLUCSC | 277 | 7 | 28087 | tss;island | 0.18225 | 0.02497 | Not queried | 2.83 | 4.08e-03 | 1.00e+00 |
|  | 19 | 49944488 | 49944964 | PDFSTATSENSEMBLUCSC | 477 | 8 | 22231 | island | 0.04232 | 0.25452 | Not queried | 2.83 | 6.22e-04 | 1.00e+00 |
|  | 8 | 22960177 | 22960427 | PDFSTATSENSEMBLUCSC | 251 | 6 | 39533 | tss | 0.25711 | 0.56908 | Not queried | 2.83 | 1.32e-01 | 1.00e+00 |
|  | 1 | 38230827 | 38230887 | PDFSTATSENSEMBLUCSC | 61 | 4 | 1504 | tss | 0.09610 | 0.48629 | Not queried | 2.83 | 2.86e-02 | 1.00e+00 |
|  | 11 | 75379432 | 75379624 | PDFSTATSENSEMBLUCSC | 193 | 7 | 8642 | island | 0.03995 | 0.21001 | Not queried | 2.83 | 1.17e-03 | 1.00e+00 |
|  | 14 | 95234658 | 95235127 | PDFSTATSENSEMBLUCSC | 470 | 7 | 12168 | gene;island | 0.09071 | 0.01682 | Not queried | 2.83 | 1.75e-02 | 1.00e+00 |
|  | 2 | 220119179 | 220119398 | PDFSTATSENSEMBLUCSC | 220 | 4 | 22994 | gene;tss;island | 0.03495 | 0.17929 | Not queried | 2.83 | 2.86e-02 | 1.00e+00 |
|  | 10 | 95517382 | 95517562 | PDFSTATSENSEMBLUCSC | 181 | 5 | 5225 | tss | 0.60550 | 0.17776 | Not queried | 2.83 | 1.59e-02 | 1.00e+00 |
|  | 16 | 66586528 | 66586745 | PDFSTATSENSEMBLUCSC | 218 | 4 | 14749 | gene | 0.21074 | 0.03403 | Not queried | 2.83 | 2.86e-02 | 1.00e+00 |
|  | 2 | 177017331 | 177017797 | PDFSTATSENSEMBLUCSC | 467 | 6 | 22902 | gene;island | 0.81117 | 0.30929 | Not queried | 2.82 | 2.16e-03 | 1.00e+00 |
|  | 5 | 92938149 | 92938402 | PDFSTATSENSEMBLUCSC | 254 | 4 | 32900 | island | 0.70119 | 0.21067 | Not queried | 2.82 | 2.86e-02 | 1.00e+00 |
|  | 2 | 242749925 | 242750102 | PDFSTATSENSEMBLUCSC | 178 | 5 | 23956 | tss | 0.55762 | 0.91132 | Not queried | 2.82 | 9.52e-02 | 1.00e+00 |
|  | X | 49687028 | 49687176 | PDFSTATSENSEMBLUCSC | 149 | 8 | 41318 | tss | 0.09051 | 0.01540 | Not queried | 2.82 | 1.48e-02 | 1.00e+00 |
|  | 6 | 33160250 | 33160604 | PDFSTATSENSEMBLUCSC | 355 | 9 | 34825 | tss | 0.09067 | 0.38213 | Not queried | 2.82 | 2.88e-04 | 1.00e+00 |
|  | 1 | 236227410 | 236227686 | PDFSTATSENSEMBLUCSC | 277 | 6 | 1093 | gene;island | 0.24889 | 0.04262 | Not queried | 2.82 | 8.66e-03 | 1.00e+00 |
|  | 6 | 72298242 | 72298704 | PDFSTATSENSEMBLUCSC | 463 | 5 | 36182 | island | 0.83102 | 0.42605 | Not queried | 2.82 | 7.94e-03 | 1.00e+00 |
|  | X | 48769307 | 48769670 | PDFSTATSENSEMBLUCSC | 364 | 7 | 41300 | tss;island | 0.14866 | 0.57461 | Not queried | 2.82 | 5.83e-04 | 1.00e+00 |
|  | 14 | 37117706 | 37117947 | PDFSTATSENSEMBLUCSC | 242 | 4 | 12761 | island | 0.01967 | 0.13218 | Not queried | 2.82 | 2.86e-02 | 1.00e+00 |
|  | 8 | 6693247 | 6693540 | PDFSTATSENSEMBLUCSC | 294 | 6 | 39476 | tss | 0.36751 | 0.79013 | Not queried | 2.82 | 6.49e-02 | 1.00e+00 |
|  | 1 | 46632363 | 46632871 | PDFSTATSENSEMBLUCSC | 509 | 7 | 3207 | island | 0.38397 | 0.81440 | Not queried | 2.82 | 2.33e-03 | 1.00e+00 |
|  | 7 | 4832112 | 4832535 | PDFSTATSENSEMBLUCSC | 424 | 5 | 38137 | island | 0.88377 | 0.53556 | Not queried | 2.82 | 7.94e-03 | 1.00e+00 |
|  | 19 | 11689488 | 11689997 | PDFSTATSENSEMBLUCSC | 510 | 12 | 20441 | tss;island | 0.63166 | 0.92566 | Not queried | 2.82 | 7.17e-05 | 3.69e-01 |
|  | 3 | 49026934 | 49027409 | PDFSTATSENSEMBLUCSC | 476 | 13 | 29059 | island | 0.53558 | 0.90155 | P4HTM; RP13-131K19.2 (margin: ARIH2; P4HTM; RP13-131K19.1; RP13-131K19.2) | 2.81 | 8.65e-06 | 4.45e-02 |
|  | 3 | 49026934 | 49027283 | PDFSTATSENSEMBLUCSC | 350 | 11 | 28270 | tss | 0.53278 | 0.90155 | Not queried | 2.81 | 8.51e-05 | 4.37e-01 |
|  | 15 | 40697419 | 40697634 | PDFSTATSENSEMBLUCSC | 216 | 4 | 13560 | tss | 0.15897 | 0.56920 | Not queried | 2.81 | 2.00e-01 | 1.00e+00 |
|  | 4 | 122685352 | 122685675 | PDFSTATSENSEMBLUCSC | 324 | 6 | 30066 | gene;tss;island | 0.46143 | 0.05760 | Not queried | 2.81 | 9.31e-02 | 1.00e+00 |
|  | 16 | 88730291 | 88730494 | PDFSTATSENSEMBLUCSC | 204 | 4 | 14879 | gene;tss;island | 0.29995 | 0.04320 | Not queried | 2.81 | 4.86e-01 | 1.00e+00 |
|  | 14 | 47120795 | 47121158 | PDFSTATSENSEMBLUCSC | 364 | 9 | 12789 | island | 0.48001 | 0.85985 | Not queried | 2.81 | 2.88e-04 | 1.00e+00 |
|  | 15 | 74537819 | 74538030 | PDFSTATSENSEMBLUCSC | 212 | 4 | 13392 | gene;island | 0.41880 | 0.79177 | Not queried | 2.81 | 2.86e-02 | 1.00e+00 |
|  | 7 | 56183885 | 56184217 | PDFSTATSENSEMBLUCSC | 333 | 4 | 38448 | island | 0.29098 | 0.75972 | Not queried | 2.81 | 2.86e-02 | 1.00e+00 |
|  | 10 | 14051636 | 14052028 | PDFSTATSENSEMBLUCSC | 393 | 6 | 4495 | gene | 0.38745 | 0.77752 | Not queried | 2.81 | 2.16e-03 | 1.00e+00 |
|  | 5 | 41870796 | 41871189 | PDFSTATSENSEMBLUCSC | 394 | 9 | 31979 | tss | 0.05395 | 0.35458 | Not queried | 2.80 | 6.25e-02 | 1.00e+00 |
|  | 7 | 89783435 | 89783976 | PDFSTATSENSEMBLUCSC | 542 | 10 | 38581 | island | 0.03613 | 0.23078 | Not queried | 2.80 | 3.89e-03 | 1.00e+00 |
|  | 10 | 23216637 | 23216847 | PDFSTATSENSEMBLUCSC | 211 | 6 | 4969 | tss | 0.04032 | 0.32995 | Not queried | 2.80 | 4.33e-03 | 1.00e+00 |
|  | 16 | 23724774 | 23724943 | PDFSTATSENSEMBLUCSC | 170 | 6 | 15736 | island | 0.76324 | 0.37646 | Not queried | 2.80 | 2.16e-03 | 1.00e+00 |
|  | 2 | 202901288 | 202901470 | PDFSTATSENSEMBLUCSC | 183 | 4 | 22955 | gene;island | 0.24029 | 0.04285 | Not queried | 2.80 | 2.86e-02 | 1.00e+00 |
|  | 22 | 32767005 | 32767162 | PDFSTATSENSEMBLUCSC | 158 | 4 | 27049 | tss | 0.88401 | 0.56476 | Not queried | 2.80 | 2.86e-02 | 1.00e+00 |
|  | 8 | 74005537 | 74005813 | PDFSTATSENSEMBLUCSC | 277 | 8 | 39703 | tss;island | 0.06077 | 0.34848 | Not queried | 2.80 | 6.22e-04 | 1.00e+00 |
|  | 6 | 41620951 | 41621322 | PDFSTATSENSEMBLUCSC | 372 | 6 | 34266 | gene;island | 0.27708 | 0.72727 | Not queried | 2.80 | 2.16e-03 | 1.00e+00 |
|  | 6 | 62996627 | 62996702 | PDFSTATSENSEMBLUCSC | 76 | 4 | 34989 | tss;island | 0.06769 | 0.35150 | Not queried | 2.80 | 2.00e-01 | 1.00e+00 |
|  | 6 | 27100370 | 27101279 | PDFSTATSENSEMBLUCSC | 910 | 13 | 34647 | tss;island | 0.01201 | 0.06692 | Not queried | 2.80 | 7.24e-03 | 1.00e+00 |
|  | 19 | 49250180 | 49250561 | PDFSTATSENSEMBLUCSC | 382 | 5 | 20890 | tss | 0.07375 | 0.02060 | Not queried | 2.79 | 7.94e-03 | 1.00e+00 |
|  | 18 | 67069959 | 67070461 | PDFSTATSENSEMBLUCSC | 503 | 6 | 19090 | gene;island | 0.11379 | 0.49385 | Not queried | 2.79 | 4.11e-02 | 1.00e+00 |
|  | 12 | 54446019 | 54446576 | PDFSTATSENSEMBLUCSC | 558 | 10 | 9136 | gene;island | 0.88256 | 0.37666 | Not queried | 2.79 | 2.09e-03 | 1.00e+00 |
|  | 16 | 419800 | 420230 | PDFSTATSENSEMBLUCSC | 431 | 5 | 14499 | gene;island | 0.75333 | 0.27275 | Not queried | 2.79 | 1.51e-01 | 1.00e+00 |
|  | 12 | 5936877 | 5937105 | PDFSTATSENSEMBLUCSC | 229 | 4 | 8996 | gene;island | 0.85374 | 0.46841 | Not queried | 2.79 | 2.86e-02 | 1.00e+00 |
|  | 3 | 44726891 | 44727195 | PDFSTATSENSEMBLUCSC | 305 | 5 | 28996 | island | 0.08802 | 0.04892 | Not queried | 2.79 | 9.52e-02 | 1.00e+00 |
|  | 11 | 133800685 | 133800913 | PDFSTATSENSEMBLUCSC | 229 | 4 | 6966 | gene;island | 0.47766 | 0.73445 | Not queried | 2.79 | 2.86e-02 | 1.00e+00 |
|  | 19 | 46094683 | 46094912 | PDFSTATSENSEMBLUCSC | 230 | 4 | 20066 | gene;island | 0.42487 | 0.70213 | Not queried | 2.79 | 3.43e-01 | 1.00e+00 |
|  | 4 | 9782891 | 9783246 | PDFSTATSENSEMBLUCSC | 356 | 8 | 30252 | tss | 0.39353 | 0.89993 | Not queried | 2.79 | 2.81e-02 | 1.00e+00 |
|  | 6 | 28501816 | 28501916 | PDFSTATSENSEMBLUCSC | 101 | 5 | 33572 | gene | 0.93978 | 0.62184 | Not queried | 2.79 | 3.10e-01 | 1.00e+00 |
|  | 11 | 119294283 | 119294441 | PDFSTATSENSEMBLUCSC | 159 | 5 | 7735 | tss;island | 0.83719 | 0.46623 | Not queried | 2.79 | 7.94e-03 | 1.00e+00 |
|  | 7 | 45197251 | 45197727 | PDFSTATSENSEMBLUCSC | 477 | 7 | 38410 | island | 0.42210 | 0.85110 | Not queried | 2.79 | 1.75e-02 | 1.00e+00 |
|  | 20 | 44098223 | 44098409 | PDFSTATSENSEMBLUCSC | 187 | 7 | 26160 | island | 0.14882 | 0.59448 | Not queried | 2.79 | 5.83e-04 | 1.00e+00 |
|  | 14 | 35099471 | 35099711 | PDFSTATSENSEMBLUCSC | 241 | 5 | 12319 | tss | 0.05040 | 0.11606 | Not queried | 2.79 | 3.10e-01 | 1.00e+00 |
|  | 19 | 8809057 | 8809360 | PDFSTATSENSEMBLUCSC | 304 | 4 | 21455 | island | 0.49170 | 0.86939 | Not queried | 2.78 | 4.86e-01 | 1.00e+00 |
|  | 12 | 56694186 | 56694667 | PDFSTATSENSEMBLUCSC | 482 | 9 | 9769 | tss | 0.02977 | 0.07638 | Not queried | 2.78 | 4.00e-02 | 1.00e+00 |
|  | 10 | 102986525 | 102986973 | PDFSTATSENSEMBLUCSC | 449 | 6 | 6041 | island | 0.77078 | 0.33298 | Not queried | 2.78 | 8.66e-03 | 1.00e+00 |
|  | 15 | 96952113 | 96952425 | PDFSTATSENSEMBLUCSC | 313 | 4 | 14462 | island | 0.95618 | 0.75520 | Not queried | 2.78 | 5.71e-02 | 1.00e+00 |
|  | 3 | 186080023 | 186080359 | PDFSTATSENSEMBLUCSC | 337 | 7 | 28729 | tss | 0.06782 | 0.34290 | Not queried | 2.78 | 5.83e-04 | 1.00e+00 |
|  | 5 | 153862183 | 153862450 | PDFSTATSENSEMBLUCSC | 268 | 4 | 33269 | island | 0.83153 | 0.49772 | Not queried | 2.78 | 2.86e-02 | 1.00e+00 |
|  | X | 117107675 | 117107923 | PDFSTATSENSEMBLUCSC | 249 | 4 | 41086 | gene | 0.30833 | 0.02316 | Not queried | 2.78 | 5.71e-02 | 1.00e+00 |
|  | 17 | 33775295 | 33776116 | PDFSTATSENSEMBLUCSC | 822 | 12 | 18181 | island | 0.45261 | 0.85822 | SLFN13 (margin: SLFN13; RP11-1094M14.7) | 2.78 | 7.40e-07 | 3.80e-03 |
|  | 3 | 10547179 | 10547228 | PDFSTATSENSEMBLUCSC | 50 | 4 | 27621 | gene | 0.19992 | 0.60576 | Not queried | 2.78 | 2.86e-02 | 1.00e+00 |
|  | 2 | 4050695 | 4050845 | PDFSTATSENSEMBLUCSC | 151 | 5 | 24002 | island | 0.21593 | 0.66962 | Not queried | 2.78 | 7.94e-03 | 1.00e+00 |
|  | 5 | 154091976 | 154092426 | PDFSTATSENSEMBLUCSC | 451 | 6 | 32391 | tss | 0.34112 | 0.71017 | Not queried | 2.78 | 2.16e-03 | 1.00e+00 |
|  | 7 | 120968877 | 120969079 | PDFSTATSENSEMBLUCSC | 203 | 7 | 37796 | tss | 0.11651 | 0.45255 | Not queried | 2.78 | 5.83e-04 | 1.00e+00 |
|  | 5 | 61601529 | 61601647 | PDFSTATSENSEMBLUCSC | 119 | 4 | 32025 | tss;island | 0.29954 | 0.05784 | Not queried | 2.78 | 2.00e-01 | 1.00e+00 |
|  | 1 | 37500195 | 37500508 | PDFSTATSENSEMBLUCSC | 314 | 5 | 1496 | tss;island | 0.12428 | 0.49202 | Not queried | 2.77 | 1.59e-02 | 1.00e+00 |
|  | 1 | 24127669 | 24128066 | PDFSTATSENSEMBLUCSC | 398 | 4 | 1345 | tss;island | 0.02092 | 0.14533 | Not queried | 2.77 | 5.71e-02 | 1.00e+00 |
|  | 5 | 19988592 | 19988944 | PDFSTATSENSEMBLUCSC | 353 | 9 | 31941 | tss | 0.02962 | 0.08484 | Not queried | 2.77 | 4.00e-02 | 1.00e+00 |
|  | 19 | 47551893 | 47551991 | PDFSTATSENSEMBLUCSC | 99 | 5 | 20865 | tss | 0.16099 | 0.04349 | Not queried | 2.77 | 9.52e-02 | 1.00e+00 |
|  | 16 | 79623618 | 79623900 | PDFSTATSENSEMBLUCSC | 283 | 5 | 16079 | island | 0.04641 | 0.24991 | Not queried | 2.77 | 2.22e-01 | 1.00e+00 |
|  | 7 | 1039875 | 1040260 | PDFSTATSENSEMBLUCSC | 386 | 4 | 36669 | gene;island | 0.39738 | 0.79243 | Not queried | 2.77 | 2.86e-02 | 1.00e+00 |
|  | 22 | 29426511 | 29426832 | PDFSTATSENSEMBLUCSC | 322 | 4 | 26788 | gene;island | 0.22854 | 0.05618 | Not queried | 2.77 | 2.86e-02 | 1.00e+00 |
|  | 6 | 156718177 | 156718546 | PDFSTATSENSEMBLUCSC | 370 | 5 | 36527 | island | 0.50384 | 0.87355 | Not queried | 2.77 | 7.94e-03 | 1.00e+00 |
|  | 11 | 68611260 | 68611596 | PDFSTATSENSEMBLUCSC | 337 | 4 | 8557 | island | 0.19774 | 0.02272 | Not queried | 2.76 | 5.71e-02 | 1.00e+00 |
|  | 6 | 134209935 | 134210257 | PDFSTATSENSEMBLUCSC | 323 | 6 | 35168 | tss | 0.33399 | 0.05527 | Not queried | 2.76 | 8.66e-03 | 1.00e+00 |
|  | 13 | 33113010 | 33113343 | PDFSTATSENSEMBLUCSC | 334 | 8 | 11356 | tss | 0.00950 | 0.07178 | Not queried | 2.76 | 1.04e-02 | 1.00e+00 |
|  | 13 | 28562798 | 28562902 | PDFSTATSENSEMBLUCSC | 105 | 7 | 11339 | tss | 0.37111 | 0.78960 | Not queried | 2.76 | 7.28e-02 | 1.00e+00 |
|  | 12 | 54133422 | 54133730 | PDFSTATSENSEMBLUCSC | 309 | 5 | 10447 | island | 0.02185 | 0.13150 | Not queried | 2.76 | 7.94e-03 | 1.00e+00 |
|  | 1 | 37499309 | 37499649 | PDFSTATSENSEMBLUCSC | 341 | 4 | 430 | gene;island | 0.14576 | 0.48953 | Not queried | 2.76 | 2.86e-02 | 1.00e+00 |
|  | 1 | 222763046 | 222763513 | PDFSTATSENSEMBLUCSC | 468 | 10 | 4216 | island | 0.20606 | 0.03510 | Not queried | 2.76 | 7.58e-05 | 3.89e-01 |
|  | 6 | 150346721 | 150347053 | PDFSTATSENSEMBLUCSC | 333 | 10 | 35230 | tss | 0.17539 | 0.67174 | Not queried | 2.76 | 1.08e-05 | 5.56e-02 |
|  | 10 | 124220359 | 124220874 | PDFSTATSENSEMBLUCSC | 516 | 9 | 5392 | tss | 0.17313 | 0.07461 | Not queried | 2.76 | 3.87e-01 | 1.00e+00 |
|  | 17 | 78161467 | 78161572 | PDFSTATSENSEMBLUCSC | 106 | 5 | 17689 | tss | 0.46288 | 0.85356 | Not queried | 2.76 | 7.94e-03 | 1.00e+00 |
|  | Y | 15016579 | 15016622 | PDFSTATSENSEMBLUCSC | 44 | 4 | 42168 | tss | 0.13275 | 0.51796 | Not queried | 2.76 | 2.86e-02 | 1.00e+00 |
|  | 1 | 24645885 | 24646392 | PDFSTATSENSEMBLUCSC | 508 | 9 | 325 | gene | 0.03056 | 0.11459 | Not queried | 2.76 | 1.85e-03 | 1.00e+00 |
|  | 1 | 1141617 | 1141991 | PDFSTATSENSEMBLUCSC | 375 | 5 | 20 | gene | 0.47915 | 0.86907 | Not queried | 2.75 | 1.59e-02 | 1.00e+00 |
|  | 17 | 37331279 | 37331768 | PDFSTATSENSEMBLUCSC | 490 | 6 | 16511 | gene;island | 0.12834 | 0.63162 | Not queried | 2.75 | 6.49e-02 | 1.00e+00 |
|  | 17 | 79283062 | 79283390 | PDFSTATSENSEMBLUCSC | 329 | 8 | 17703 | tss | 0.03492 | 0.18597 | Not queried | 2.75 | 6.99e-03 | 1.00e+00 |
|  | 17 | 79282893 | 79283390 | PDFSTATSENSEMBLUCSC | 498 | 10 | 18880 | island | 0.04171 | 0.19837 | Not queried | 2.75 | 1.05e-03 | 1.00e+00 |
|  | 2 | 11924984 | 11925375 | PDFSTATSENSEMBLUCSC | 392 | 4 | 22538 | gene;island | 0.10690 | 0.39086 | Not queried | 2.75 | 3.43e-01 | 1.00e+00 |
|  | 11 | 63258712 | 63258803 | PDFSTATSENSEMBLUCSC | 92 | 6 | 7354 | tss | 0.76639 | 0.95685 | Not queried | 2.75 | 1.52e-02 | 1.00e+00 |
|  | 11 | 86383241 | 86383940 | PDFSTATSENSEMBLUCSC | 700 | 18 | 7589 | tss | 0.17821 | 0.60214 | Not queried | 2.75 | 1.17e-02 | 1.00e+00 |
|  | 3 | 158390510 | 158390821 | PDFSTATSENSEMBLUCSC | 312 | 7 | 28643 | tss | 0.08304 | 0.54637 | Not queried | 2.75 | 2.59e-01 | 1.00e+00 |
|  | X | 69353387 | 69353974 | PDFSTATSENSEMBLUCSC | 588 | 5 | 41014 | gene;island | 0.09157 | 0.02232 | Not queried | 2.75 | 1.51e-01 | 1.00e+00 |
|  | 8 | 10928985 | 10929210 | PDFSTATSENSEMBLUCSC | 226 | 4 | 39140 | gene;island | 0.28075 | 0.75210 | Not queried | 2.75 | 5.71e-02 | 1.00e+00 |
|  | 4 | 174451954 | 174452159 | PDFSTATSENSEMBLUCSC | 206 | 4 | 30133 | gene;tss;island | 0.86681 | 0.50143 | Not queried | 2.75 | 2.00e-01 | 1.00e+00 |
|  | 6 | 32064491 | 32065211 | PDFSTATSENSEMBLUCSC | 721 | 29 | 33985 | gene;island | 0.43415 | 0.82096 | TNXB; ATF6B (margin: TNXB; ATF6B) | 2.75 | 1.79e-07 | 9.18e-04 |
|  | 14 | 73706367 | 73706676 | PDFSTATSENSEMBLUCSC | 310 | 4 | 12101 | gene;island | 0.34325 | 0.08107 | Not queried | 2.75 | 2.86e-02 | 1.00e+00 |
|  | 4 | 71337658 | 71337873 | PDFSTATSENSEMBLUCSC | 216 | 4 | 29971 | gene | 0.26423 | 0.57181 | Not queried | 2.75 | 3.43e-01 | 1.00e+00 |
|  | 22 | 23522307 | 23522748 | PDFSTATSENSEMBLUCSC | 442 | 9 | 27307 | island | 0.43579 | 0.09564 | Not queried | 2.75 | 1.90e-01 | 1.00e+00 |
|  | 5 | 1174426 | 1174666 | PDFSTATSENSEMBLUCSC | 241 | 4 | 32551 | island | 0.23050 | 0.81909 | Not queried | 2.75 | 2.00e-01 | 1.00e+00 |
|  | 19 | 47925344 | 47925618 | PDFSTATSENSEMBLUCSC | 275 | 5 | 22159 | island | 0.31710 | 0.06153 | Not queried | 2.74 | 9.52e-02 | 1.00e+00 |
|  | 8 | 71520850 | 71520989 | PDFSTATSENSEMBLUCSC | 140 | 4 | 39696 | tss;island | 0.23491 | 0.03900 | Not queried | 2.74 | 5.71e-02 | 1.00e+00 |
|  | 16 | 2908157 | 2908245 | PDFSTATSENSEMBLUCSC | 89 | 4 | 15592 | island | 0.16249 | 0.50355 | Not queried | 2.74 | 5.71e-02 | 1.00e+00 |
|  | 22 | 40766469 | 40766549 | PDFSTATSENSEMBLUCSC | 81 | 6 | 27120 | tss | 0.05124 | 0.16837 | Not queried | 2.74 | 4.33e-03 | 1.00e+00 |
|  | 16 | 1069368 | 1069760 | PDFSTATSENSEMBLUCSC | 393 | 4 | 15476 | island | 0.58550 | 0.92486 | Not queried | 2.74 | 2.86e-02 | 1.00e+00 |
|  | X | 135230594 | 135230717 | PDFSTATSENSEMBLUCSC | 124 | 4 | 41546 | tss | 0.38526 | 0.10505 | Not queried | 2.74 | 1.14e-01 | 1.00e+00 |
|  | 19 | 54466297 | 54466724 | PDFSTATSENSEMBLUCSC | 428 | 5 | 20174 | gene | 0.57421 | 0.23824 | Not queried | 2.74 | 1.59e-02 | 1.00e+00 |
|  | 11 | 72928926 | 72929394 | PDFSTATSENSEMBLUCSC | 469 | 12 | 7524 | tss | 0.21683 | 0.02920 | Not queried | 2.74 | 1.45e-02 | 1.00e+00 |
|  | 10 | 65280766 | 65280961 | PDFSTATSENSEMBLUCSC | 196 | 5 | 5089 | tss;island | 0.18096 | 0.07636 | Not queried | 2.74 | 9.52e-02 | 1.00e+00 |
|  | 19 | 51522454 | 51522921 | PDFSTATSENSEMBLUCSC | 468 | 7 | 20151 | gene;island | 0.53419 | 0.14666 | Not queried | 2.74 | 6.99e-03 | 1.00e+00 |
|  | 19 | 47551842 | 47551991 | PDFSTATSENSEMBLUCSC | 150 | 7 | 22151 | island | 0.19465 | 0.03928 | Not queried | 2.74 | 1.75e-02 | 1.00e+00 |
|  | 1 | 24307153 | 24307535 | PDFSTATSENSEMBLUCSC | 383 | 5 | 1350 | tss;island | 0.96907 | 0.72054 | Not queried | 2.74 | 3.17e-02 | 1.00e+00 |
|  | 3 | 40657083 | 40657497 | PDFSTATSENSEMBLUCSC | 415 | 6 | 28965 | island | 0.37873 | 0.09173 | Not queried | 2.74 | 6.49e-02 | 1.00e+00 |
|  | X | 70521196 | 70521525 | PDFSTATSENSEMBLUCSC | 330 | 6 | 41376 | tss | 0.75848 | 0.93634 | Not queried | 2.74 | 2.16e-03 | 1.00e+00 |
|  | 1 | 17026786 | 17027073 | PDFSTATSENSEMBLUCSC | 288 | 4 | 260 | gene;island | 0.14914 | 0.53741 | Not queried | 2.73 | 5.71e-02 | 1.00e+00 |
|  | 6 | 30653167 | 30653799 | PDFSTATSENSEMBLUCSC | 633 | 12 | 33741 | gene;island | 0.24017 | 0.64951 | Not queried | 2.73 | 2.91e-03 | 1.00e+00 |
|  | 10 | 46168220 | 46168597 | PDFSTATSENSEMBLUCSC | 378 | 8 | 5697 | island | 0.03226 | 0.19957 | Not queried | 2.73 | 1.48e-02 | 1.00e+00 |
|  | 21 | 46131601 | 46131923 | PDFSTATSENSEMBLUCSC | 323 | 5 | 26546 | tss | 0.22130 | 0.65368 | Not queried | 2.73 | 3.17e-02 | 1.00e+00 |
|  | 7 | 109600399 | 109600733 | PDFSTATSENSEMBLUCSC | 335 | 4 | 37772 | tss | 0.49612 | 0.86106 | Not queried | 2.73 | 3.43e-01 | 1.00e+00 |
|  | 12 | 106978920 | 106979229 | PDFSTATSENSEMBLUCSC | 310 | 4 | 9255 | gene;island | 0.29773 | 0.05770 | Not queried | 2.73 | 2.86e-02 | 1.00e+00 |
|  | 8 | 57359200 | 57359414 | PDFSTATSENSEMBLUCSC | 215 | 4 | 39650 | tss;island | 0.13464 | 0.53154 | Not queried | 2.73 | 2.86e-02 | 1.00e+00 |
|  | 2 | 26785120 | 26785531 | PDFSTATSENSEMBLUCSC | 412 | 10 | 24116 | island | 0.50175 | 0.85125 | Not queried | 2.73 | 1.08e-05 | 5.56e-02 |
|  | 11 | 71954982 | 71955599 | PDFSTATSENSEMBLUCSC | 618 | 11 | 8607 | island | 0.21203 | 0.01931 | Not queried | 2.73 | 1.98e-05 | 1.02e-01 |
|  | 21 | 45232531 | 45232601 | PDFSTATSENSEMBLUCSC | 71 | 4 | 26538 | tss | 0.32161 | 0.67467 | Not queried | 2.73 | 2.86e-02 | 1.00e+00 |
|  | 8 | 67782729 | 67783229 | PDFSTATSENSEMBLUCSC | 501 | 6 | 39684 | tss | 0.21495 | 0.71830 | Not queried | 2.72 | 1.52e-02 | 1.00e+00 |
|  | 3 | 46759438 | 46759698 | PDFSTATSENSEMBLUCSC | 261 | 7 | 28237 | tss | 0.27916 | 0.85980 | Not queried | 2.72 | 5.30e-02 | 1.00e+00 |
|  | 8 | 142401373 | 142401867 | PDFSTATSENSEMBLUCSC | 495 | 4 | 40557 | island | 0.21635 | 0.72677 | Not queried | 2.72 | 1.14e-01 | 1.00e+00 |
|  | 2 | 163695776 | 163696187 | PDFSTATSENSEMBLUCSC | 412 | 6 | 23652 | tss | 0.10906 | 0.45149 | Not queried | 2.72 | 4.33e-03 | 1.00e+00 |
|  | 15 | 32933661 | 32933835 | PDFSTATSENSEMBLUCSC | 175 | 4 | 13524 | tss | 0.13274 | 0.49956 | Not queried | 2.72 | 5.71e-02 | 1.00e+00 |
|  | 16 | 850240 | 850880 | PDFSTATSENSEMBLUCSC | 641 | 8 | 15459 | island | 0.03933 | 0.33108 | Not queried | 2.72 | 1.04e-02 | 1.00e+00 |
|  | 2 | 219848258 | 219848580 | PDFSTATSENSEMBLUCSC | 323 | 5 | 22985 | gene;island | 0.88134 | 0.49765 | Not queried | 2.72 | 9.52e-02 | 1.00e+00 |
|  | 5 | 140248564 | 140249080 | PDFSTATSENSEMBLUCSC | 517 | 5 | 31744 | gene;island | 0.12155 | 0.50878 | Not queried | 2.72 | 9.52e-02 | 1.00e+00 |
|  | 20 | 36151003 | 36151338 | PDFSTATSENSEMBLUCSC | 336 | 4 | 25379 | gene;island | 0.55384 | 0.82649 | Not queried | 2.72 | 5.71e-02 | 1.00e+00 |
|  | 7 | 158379132 | 158379348 | PDFSTATSENSEMBLUCSC | 217 | 4 | 37315 | gene;island | 0.61170 | 0.89876 | Not queried | 2.72 | 5.71e-02 | 1.00e+00 |
|  | 9 | 130679027 | 130679246 | PDFSTATSENSEMBLUCSC | 220 | 4 | 40720 | gene;island | 0.10496 | 0.04241 | Not queried | 2.72 | 3.43e-01 | 1.00e+00 |
|  | 12 | 12223661 | 12223989 | PDFSTATSENSEMBLUCSC | 329 | 4 | 9542 | tss | 0.41508 | 0.81850 | Not queried | 2.72 | 1.14e-01 | 1.00e+00 |
|  | X | 54556330 | 54557002 | PDFSTATSENSEMBLUCSC | 673 | 13 | 41814 | island | 0.21434 | 0.03126 | Not queried | 2.72 | 1.25e-03 | 1.00e+00 |
|  | 17 | 78161467 | 78161706 | PDFSTATSENSEMBLUCSC | 240 | 6 | 16824 | gene;island | 0.47117 | 0.85436 | Not queried | 2.72 | 4.33e-03 | 1.00e+00 |
|  | 12 | 132905611 | 132906010 | PDFSTATSENSEMBLUCSC | 400 | 4 | 11014 | island | 0.35113 | 0.77540 | Not queried | 2.72 | 2.86e-02 | 1.00e+00 |
|  | 7 | 28450687 | 28451011 | PDFSTATSENSEMBLUCSC | 325 | 5 | 36881 | gene;tss;island | 0.01784 | 0.04453 | Not queried | 2.71 | 1.51e-01 | 1.00e+00 |
|  | 17 | 45330862 | 45331082 | PDFSTATSENSEMBLUCSC | 221 | 5 | 17407 | tss;island | 0.54404 | 0.12396 | Not queried | 2.71 | 2.22e-01 | 1.00e+00 |
|  | 16 | 69958626 | 69958964 | PDFSTATSENSEMBLUCSC | 339 | 4 | 14793 | gene | 0.59447 | 0.82015 | Not queried | 2.71 | 1.14e-01 | 1.00e+00 |
|  | 20 | 13279761 | 13280201 | PDFSTATSENSEMBLUCSC | 441 | 6 | 25321 | gene;island | 0.40188 | 0.78782 | Not queried | 2.71 | 2.16e-03 | 1.00e+00 |
|  | 12 | 670520 | 671027 | PDFSTATSENSEMBLUCSC | 508 | 5 | 8971 | gene | 0.24678 | 0.69033 | Not queried | 2.71 | 7.94e-03 | 1.00e+00 |
|  | 2 | 19561327 | 19561709 | PDFSTATSENSEMBLUCSC | 383 | 4 | 24065 | island | 0.74129 | 0.31636 | Not queried | 2.71 | 2.86e-02 | 1.00e+00 |
|  | 1 | 6304199 | 6304499 | PDFSTATSENSEMBLUCSC | 301 | 7 | 2594 | island | 0.53761 | 0.09738 | Not queried | 2.71 | 1.11e-02 | 1.00e+00 |
|  | 20 | 56725695 | 56725899 | PDFSTATSENSEMBLUCSC | 205 | 8 | 25808 | tss | 0.34504 | 0.77798 | Not queried | 2.71 | 3.11e-04 | 1.00e+00 |
|  | 19 | 9473565 | 9473880 | PDFSTATSENSEMBLUCSC | 316 | 10 | 21461 | island | 0.90469 | 0.61371 | Not queried | 2.71 | 1.08e-05 | 5.56e-02 |
|  | 19 | 4173254 | 4173482 | PDFSTATSENSEMBLUCSC | 229 | 4 | 21329 | island | 0.01898 | 0.20709 | Not queried | 2.71 | 1.14e-01 | 1.00e+00 |
|  | 1 | 3182620 | 3183165 | PDFSTATSENSEMBLUCSC | 546 | 5 | 113 | gene;island | 0.63039 | 0.87668 | Not queried | 2.71 | 5.56e-02 | 1.00e+00 |
|  | 17 | 25676201 | 25676574 | PDFSTATSENSEMBLUCSC | 374 | 4 | 18101 | island | 0.14335 | 0.53619 | Not queried | 2.71 | 2.00e-01 | 1.00e+00 |
|  | 10 | 131694575 | 131694856 | PDFSTATSENSEMBLUCSC | 282 | 5 | 4820 | gene;island | 0.83842 | 0.41342 | Not queried | 2.71 | 2.22e-01 | 1.00e+00 |
|  | 13 | 49549440 | 49549665 | PDFSTATSENSEMBLUCSC | 226 | 4 | 11409 | tss;island | 0.19727 | 0.01958 | Not queried | 2.70 | 1.14e-01 | 1.00e+00 |
|  | X | 15756372 | 15756407 | PDFSTATSENSEMBLUCSC | 36 | 4 | 41202 | tss;island | 0.02384 | 0.18953 | Not queried | 2.70 | 5.71e-02 | 1.00e+00 |
|  | 12 | 106974832 | 106975246 | PDFSTATSENSEMBLUCSC | 415 | 5 | 10724 | island | 0.24463 | 0.06257 | Not queried | 2.70 | 2.22e-01 | 1.00e+00 |
|  | 7 | 766100 | 766323 | PDFSTATSENSEMBLUCSC | 224 | 6 | 37324 | tss | 0.21670 | 0.01767 | Not queried | 2.70 | 2.40e-01 | 1.00e+00 |
|  | 18 | 54305799 | 54306437 | PDFSTATSENSEMBLUCSC | 639 | 11 | 19495 | island | 0.02468 | 0.09311 | Not queried | 2.70 | 4.10e-03 | 1.00e+00 |
|  | 8 | 61591199 | 61591461 | PDFSTATSENSEMBLUCSC | 263 | 5 | 40267 | island | 0.02106 | 0.04783 | Not queried | 2.70 | 9.52e-02 | 1.00e+00 |
|  | 6 | 152702330 | 152702660 | PDFSTATSENSEMBLUCSC | 331 | 6 | 34451 | gene | 0.29975 | 0.69906 | Not queried | 2.70 | 1.52e-02 | 1.00e+00 |
|  | 11 | 60414615 | 60415111 | PDFSTATSENSEMBLUCSC | 497 | 5 | 6603 | gene;island | 0.12043 | 0.47262 | Not queried | 2.70 | 7.94e-03 | 1.00e+00 |
|  | 2 | 27072271 | 27072564 | PDFSTATSENSEMBLUCSC | 294 | 4 | 22580 | gene;island | 0.23049 | 0.03847 | Not queried | 2.70 | 2.86e-02 | 1.00e+00 |
|  | 17 | 1811181 | 1811625 | PDFSTATSENSEMBLUCSC | 445 | 5 | 17800 | island | 0.13996 | 0.30676 | Not queried | 2.70 | 3.17e-02 | 1.00e+00 |
|  | 11 | 73357019 | 73357396 | PDFSTATSENSEMBLUCSC | 378 | 8 | 7529 | tss | 0.76753 | 0.34540 | Not queried | 2.70 | 3.11e-04 | 1.00e+00 |
|  | 12 | 9800259 | 9800818 | PDFSTATSENSEMBLUCSC | 560 | 11 | 10211 | island | 0.07606 | 0.03185 | Not queried | 2.70 | 4.10e-03 | 1.00e+00 |
|  | 10 | 50816917 | 50817306 | PDFSTATSENSEMBLUCSC | 390 | 6 | 5056 | tss;island | 0.43224 | 0.11271 | Not queried | 2.70 | 4.33e-03 | 1.00e+00 |
|  | 10 | 112256517 | 112256825 | PDFSTATSENSEMBLUCSC | 309 | 4 | 5339 | tss;island | 0.36776 | 0.06404 | Not queried | 2.70 | 2.00e-01 | 1.00e+00 |
|  | 17 | 58499679 | 58499854 | PDFSTATSENSEMBLUCSC | 176 | 6 | 17519 | tss | 0.92745 | 0.70882 | Not queried | 2.70 | 8.66e-03 | 1.00e+00 |
|  | 10 | 35894012 | 35894321 | PDFSTATSENSEMBLUCSC | 310 | 7 | 5011 | tss | 0.37891 | 0.88919 | Not queried | 2.69 | 2.33e-03 | 1.00e+00 |
|  | 14 | 105104840 | 105105198 | PDFSTATSENSEMBLUCSC | 359 | 4 | 13180 | island | 0.41337 | 0.77179 | Not queried | 2.69 | 1.14e-01 | 1.00e+00 |
|  | 16 | 14403004 | 14403138 | PDFSTATSENSEMBLUCSC | 135 | 4 | 15071 | tss | 0.73621 | 0.93736 | Not queried | 2.69 | 1.14e-01 | 1.00e+00 |
|  | 3 | 113160430 | 113160821 | PDFSTATSENSEMBLUCSC | 392 | 7 | 28463 | tss | 0.67288 | 0.93617 | Not queried | 2.69 | 1.75e-02 | 1.00e+00 |
|  | 16 | 57023022 | 57023398 | PDFSTATSENSEMBLUCSC | 377 | 7 | 15225 | tss | 0.21981 | 0.06187 | Not queried | 2.69 | 2.59e-01 | 1.00e+00 |
|  | 18 | 44337664 | 44338147 | PDFSTATSENSEMBLUCSC | 484 | 6 | 19226 | tss;island | 0.03268 | 0.19073 | Not queried | 2.69 | 2.60e-02 | 1.00e+00 |
|  | 2 | 19553237 | 19553546 | PDFSTATSENSEMBLUCSC | 310 | 4 | 22550 | gene;island | 0.87775 | 0.59410 | Not queried | 2.69 | 2.00e-01 | 1.00e+00 |
|  | 12 | 93096622 | 93096819 | PDFSTATSENSEMBLUCSC | 198 | 4 | 9888 | tss | 0.72330 | 0.94575 | Not queried | 2.69 | 2.86e-02 | 1.00e+00 |
|  | X | 48937688 | 48937975 | PDFSTATSENSEMBLUCSC | 288 | 5 | 40991 | gene;tss;island | 0.09105 | 0.01938 | Not queried | 2.69 | 1.59e-02 | 1.00e+00 |
|  | 8 | 37797680 | 37797828 | PDFSTATSENSEMBLUCSC | 149 | 5 | 39585 | tss | 0.31437 | 0.74203 | Not queried | 2.69 | 7.94e-03 | 1.00e+00 |
|  | 6 | 29523592 | 29524117 | PDFSTATSENSEMBLUCSC | 526 | 8 | 33597 | gene;island | 0.49533 | 0.84315 | Not queried | 2.69 | 3.11e-04 | 1.00e+00 |
|  | 19 | 14196219 | 14196582 | PDFSTATSENSEMBLUCSC | 364 | 4 | 21636 | island | 0.67903 | 0.19558 | Not queried | 2.69 | 2.86e-02 | 1.00e+00 |
|  | 2 | 220377593 | 220377885 | PDFSTATSENSEMBLUCSC | 293 | 4 | 23870 | tss;island | 0.19978 | 0.62183 | Not queried | 2.69 | 1.14e-01 | 1.00e+00 |
|  | 14 | 105154557 | 105155327 | PDFSTATSENSEMBLUCSC | 771 | 7 | 12632 | tss;island | 0.36023 | 0.77092 | Not queried | 2.68 | 2.33e-03 | 1.00e+00 |
|  | 19 | 1155030 | 1155225 | PDFSTATSENSEMBLUCSC | 196 | 4 | 19612 | gene;island | 0.52595 | 0.86740 | Not queried | 2.68 | 2.86e-02 | 1.00e+00 |
|  | 1 | 38022359 | 38022718 | PDFSTATSENSEMBLUCSC | 360 | 8 | 3061 | island | 0.41686 | 0.05772 | Not queried | 2.68 | 1.30e-01 | 1.00e+00 |
|  | 17 | 58499679 | 58499911 | PDFSTATSENSEMBLUCSC | 233 | 7 | 18588 | island | 0.90576 | 0.70041 | Not queried | 2.68 | 2.33e-03 | 1.00e+00 |
|  | 12 | 72233240 | 72233572 | PDFSTATSENSEMBLUCSC | 333 | 11 | 10600 | island | 0.18789 | 0.06269 | Not queried | 2.68 | 7.59e-02 | 1.00e+00 |
|  | 4 | 54424476 | 54424787 | PDFSTATSENSEMBLUCSC | 312 | 4 | 30349 | tss | 0.37474 | 0.76013 | Not queried | 2.68 | 1.14e-01 | 1.00e+00 |
|  | 8 | 37824306 | 37824529 | PDFSTATSENSEMBLUCSC | 224 | 4 | 39586 | tss;island | 0.52208 | 0.24369 | Not queried | 2.67 | 2.00e-01 | 1.00e+00 |
|  | 7 | 48019388 | 48019713 | PDFSTATSENSEMBLUCSC | 326 | 4 | 37525 | tss | 0.66842 | 0.95016 | Not queried | 2.67 | 1.14e-01 | 1.00e+00 |
|  | 6 | 32920567 | 32920880 | PDFSTATSENSEMBLUCSC | 314 | 5 | 34101 | gene | 0.76126 | 0.35003 | Not queried | 2.67 | 1.51e-01 | 1.00e+00 |
|  | 12 | 68041257 | 68041428 | PDFSTATSENSEMBLUCSC | 172 | 4 | 9827 | tss;island | 0.16836 | 0.04640 | Not queried | 2.67 | 1.14e-01 | 1.00e+00 |
|  | X | 48379816 | 48380420 | PDFSTATSENSEMBLUCSC | 605 | 6 | 41763 | island | 0.14896 | 0.50173 | Not queried | 2.67 | 2.16e-03 | 1.00e+00 |
|  | 17 | 33775860 | 33776116 | PDFSTATSENSEMBLUCSC | 257 | 7 | 17238 | tss | 0.48817 | 0.82778 | Not queried | 2.67 | 5.83e-04 | 1.00e+00 |
|  | 6 | 100060917 | 100061504 | PDFSTATSENSEMBLUCSC | 588 | 6 | 34364 | gene;island | 0.07403 | 0.03170 | Not queried | 2.67 | 1.32e-01 | 1.00e+00 |
|  | 10 | 112258394 | 112258680 | PDFSTATSENSEMBLUCSC | 287 | 4 | 4739 | gene;island | 0.20073 | 0.03673 | Not queried | 2.67 | 1.14e-01 | 1.00e+00 |
|  | 6 | 99290284 | 99290737 | PDFSTATSENSEMBLUCSC | 454 | 5 | 36283 | island | 0.68832 | 0.21136 | Not queried | 2.67 | 7.94e-03 | 1.00e+00 |
|  | 11 | 65339691 | 65339797 | PDFSTATSENSEMBLUCSC | 107 | 5 | 7413 | tss;island | 0.04436 | 0.22475 | Not queried | 2.67 | 7.94e-03 | 1.00e+00 |
|  | 8 | 125741158 | 125741346 | PDFSTATSENSEMBLUCSC | 189 | 5 | 39843 | tss;island | 0.46779 | 0.14078 | Not queried | 2.67 | 5.56e-02 | 1.00e+00 |
|  | 6 | 30881112 | 30882094 | PDFSTATSENSEMBLUCSC | 983 | 28 | 34729 | tss | 0.06824 | 0.59315 | Not queried | 2.67 | 1.77e-05 | 9.08e-02 |
|  | 1 | 153283628 | 153283994 | PDFSTATSENSEMBLUCSC | 367 | 4 | 1936 | tss | 0.76095 | 0.34174 | Not queried | 2.66 | 2.00e-01 | 1.00e+00 |
|  | 12 | 52545884 | 52546408 | PDFSTATSENSEMBLUCSC | 525 | 5 | 10410 | island | 0.21143 | 0.65193 | Not queried | 2.66 | 7.94e-03 | 1.00e+00 |
|  | 11 | 121986961 | 121987203 | PDFSTATSENSEMBLUCSC | 243 | 7 | 6928 | gene;tss | 0.61543 | 0.81639 | Not queried | 2.66 | 1.75e-02 | 1.00e+00 |
|  | 6 | 29594830 | 29595661 | PDFSTATSENSEMBLUCSC | 832 | 15 | 33615 | gene;island | 0.33964 | 0.03911 | Not queried | 2.66 | 1.96e-03 | 1.00e+00 |
|  | 16 | 66412840 | 66413098 | PDFSTATSENSEMBLUCSC | 259 | 5 | 14748 | gene | 0.17734 | 0.55438 | Not queried | 2.66 | 3.17e-02 | 1.00e+00 |
|  | 2 | 71693090 | 71693793 | PDFSTATSENSEMBLUCSC | 704 | 9 | 23393 | tss | 0.30839 | 0.15488 | Not queried | 2.66 | 1.06e-02 | 1.00e+00 |
|  | 2 | 27805536 | 27805844 | PDFSTATSENSEMBLUCSC | 309 | 7 | 23235 | tss | 0.87222 | 0.51144 | Not queried | 2.66 | 5.83e-04 | 1.00e+00 |
|  | 11 | 71955299 | 71955599 | PDFSTATSENSEMBLUCSC | 301 | 8 | 7519 | tss | 0.21748 | 0.02672 | Not queried | 2.66 | 6.22e-04 | 1.00e+00 |
|  | 17 | 76967629 | 76967825 | PDFSTATSENSEMBLUCSC | 197 | 4 | 16794 | gene | 0.08835 | 0.50581 | Not queried | 2.66 | 3.43e-01 | 1.00e+00 |
|  | 5 | 38846475 | 38846743 | PDFSTATSENSEMBLUCSC | 269 | 4 | 31541 | gene;island | 0.10737 | 0.34798 | Not queried | 2.66 | 2.00e-01 | 1.00e+00 |
|  | 7 | 100964669 | 100964998 | PDFSTATSENSEMBLUCSC | 330 | 5 | 37092 | gene;island | 0.06470 | 0.12563 | Not queried | 2.66 | 9.52e-02 | 1.00e+00 |
|  | 11 | 320732 | 320956 | PDFSTATSENSEMBLUCSC | 225 | 6 | 7820 | island | 0.17467 | 0.52489 | Not queried | 2.65 | 1.52e-02 | 1.00e+00 |
|  | 1 | 1245014 | 1245184 | PDFSTATSENSEMBLUCSC | 171 | 4 | 29 | gene;island | 0.25502 | 0.63549 | Not queried | 2.65 | 1.14e-01 | 1.00e+00 |
|  | 22 | 17565455 | 17565750 | PDFSTATSENSEMBLUCSC | 296 | 6 | 26899 | tss | 0.36062 | 0.03917 | Not queried | 2.65 | 1.80e-01 | 1.00e+00 |
|  | 5 | 140571783 | 140572039 | PDFSTATSENSEMBLUCSC | 257 | 5 | 33160 | island | 0.03241 | 0.27296 | Not queried | 2.65 | 6.90e-01 | 1.00e+00 |
|  | 19 | 50931222 | 50931622 | PDFSTATSENSEMBLUCSC | 401 | 6 | 20139 | gene;island | 0.39391 | 0.77983 | Not queried | 2.65 | 2.16e-03 | 1.00e+00 |
|  | 13 | 100497453 | 100497789 | PDFSTATSENSEMBLUCSC | 337 | 4 | 11178 | gene | 0.77182 | 0.96306 | Not queried | 2.65 | 2.86e-02 | 1.00e+00 |
|  | 19 | 40732076 | 40732316 | PDFSTATSENSEMBLUCSC | 241 | 4 | 20001 | gene;island | 0.34549 | 0.69825 | Not queried | 2.65 | 2.86e-02 | 1.00e+00 |
|  | 20 | 57414039 | 57414596 | PDFSTATSENSEMBLUCSC | 558 | 11 | 25431 | gene;tss;island | 0.62223 | 0.91910 | Not queried | 2.65 | 1.28e-04 | 6.56e-01 |
|  | 6 | 25882328 | 25882590 | PDFSTATSENSEMBLUCSC | 263 | 5 | 35439 | island | 0.10470 | 0.32149 | Not queried | 2.64 | 9.52e-02 | 1.00e+00 |
|  | X | 99667001 | 99667223 | PDFSTATSENSEMBLUCSC | 223 | 4 | 41900 | island | 0.07996 | 0.40065 | Not queried | 2.64 | 2.86e-02 | 1.00e+00 |
|  | 19 | 1248287 | 1248507 | PDFSTATSENSEMBLUCSC | 221 | 5 | 20259 | tss | 0.42583 | 0.10172 | Not queried | 2.64 | 2.22e-01 | 1.00e+00 |
|  | 18 | 78004045 | 78004474 | PDFSTATSENSEMBLUCSC | 430 | 4 | 19114 | gene;island | 0.61724 | 0.20783 | Not queried | 2.64 | 3.43e-01 | 1.00e+00 |
|  | 10 | 21799047 | 21799395 | PDFSTATSENSEMBLUCSC | 349 | 4 | 5576 | island | 0.20790 | 0.06415 | Not queried | 2.64 | 3.43e-01 | 1.00e+00 |
|  | 2 | 177028606 | 177028804 | PDFSTATSENSEMBLUCSC | 199 | 5 | 24873 | island | 0.83470 | 0.47027 | Not queried | 2.64 | 7.94e-03 | 1.00e+00 |
|  | 19 | 34112111 | 34112376 | PDFSTATSENSEMBLUCSC | 266 | 9 | 20651 | tss;island | 0.36838 | 0.05798 | Not queried | 2.64 | 1.06e-02 | 1.00e+00 |
|  | 19 | 50316212 | 50316458 | PDFSTATSENSEMBLUCSC | 247 | 4 | 20128 | gene | 0.14075 | 0.04345 | Not queried | 2.64 | 2.86e-02 | 1.00e+00 |
|  | 6 | 151646540 | 151646651 | PDFSTATSENSEMBLUCSC | 112 | 5 | 35235 | tss | 0.27881 | 0.05149 | Not queried | 2.64 | 7.94e-03 | 1.00e+00 |
|  | 14 | 37666810 | 37666994 | PDFSTATSENSEMBLUCSC | 185 | 5 | 12333 | tss;island | 0.01003 | 0.09055 | Not queried | 2.64 | 1.51e-01 | 1.00e+00 |
|  | 11 | 2019079 | 2019167 | PDFSTATSENSEMBLUCSC | 89 | 5 | 7034 | tss;island | 0.06693 | 0.34400 | Not queried | 2.64 | 1.59e-02 | 1.00e+00 |
|  | 17 | 78039301 | 78039528 | PDFSTATSENSEMBLUCSC | 228 | 4 | 16819 | gene;island | 0.54473 | 0.82751 | Not queried | 2.63 | 2.86e-02 | 1.00e+00 |
|  | 19 | 15838756 | 15839006 | PDFSTATSENSEMBLUCSC | 251 | 4 | 21674 | island | 0.16805 | 0.54654 | Not queried | 2.63 | 2.86e-02 | 1.00e+00 |
|  | 1 | 6684860 | 6685162 | PDFSTATSENSEMBLUCSC | 303 | 7 | 1211 | tss;island | 0.03974 | 0.02685 | Not queried | 2.63 | 9.73e-02 | 1.00e+00 |
|  | 4 | 54965829 | 54966187 | PDFSTATSENSEMBLUCSC | 359 | 5 | 30352 | tss | 0.08165 | 0.02898 | Not queried | 2.63 | 2.22e-01 | 1.00e+00 |
|  | 6 | 43337670 | 43337791 | PDFSTATSENSEMBLUCSC | 122 | 5 | 34933 | tss;island | 0.03865 | 0.02199 | Not queried | 2.63 | 6.90e-01 | 1.00e+00 |
|  | 12 | 56075367 | 56075511 | PDFSTATSENSEMBLUCSC | 145 | 4 | 9141 | gene | 0.02870 | 0.16487 | Not queried | 2.63 | 5.71e-02 | 1.00e+00 |
|  | 6 | 170605952 | 170606506 | PDFSTATSENSEMBLUCSC | 555 | 6 | 36630 | island | 0.29396 | 0.07044 | Not queried | 2.63 | 4.33e-03 | 1.00e+00 |
|  | X | 152875453 | 152875939 | PDFSTATSENSEMBLUCSC | 487 | 4 | 42114 | island | 0.01893 | 0.23784 | Not queried | 2.63 | 4.86e-01 | 1.00e+00 |
|  | 6 | 33085470 | 33085800 | PDFSTATSENSEMBLUCSC | 331 | 8 | 34124 | gene;island | 0.22189 | 0.54470 | Not queried | 2.63 | 6.22e-04 | 1.00e+00 |
|  | 3 | 141516232 | 141516705 | PDFSTATSENSEMBLUCSC | 474 | 6 | 27934 | gene;island | 0.11430 | 0.02284 | Not queried | 2.63 | 2.60e-02 | 1.00e+00 |
|  | 6 | 26273370 | 26273611 | PDFSTATSENSEMBLUCSC | 242 | 5 | 33546 | gene | 0.56601 | 0.88965 | Not queried | 2.63 | 3.17e-02 | 1.00e+00 |
|  | 11 | 122850490 | 122850741 | PDFSTATSENSEMBLUCSC | 252 | 4 | 6929 | gene;island | 0.08080 | 0.43238 | Not queried | 2.63 | 1.14e-01 | 1.00e+00 |
|  | 12 | 133100333 | 133100714 | PDFSTATSENSEMBLUCSC | 382 | 7 | 9424 | gene;island | 0.37293 | 0.78667 | Not queried | 2.63 | 6.99e-03 | 1.00e+00 |
|  | 11 | 46366643 | 46366884 | PDFSTATSENSEMBLUCSC | 242 | 6 | 7255 | tss | 0.17852 | 0.58793 | Not queried | 2.63 | 8.66e-03 | 1.00e+00 |
|  | 11 | 1891699 | 1892392 | PDFSTATSENSEMBLUCSC | 694 | 13 | 6417 | gene;island | 0.38573 | 0.73251 | Not queried | 2.63 | 1.25e-03 | 1.00e+00 |
|  | 5 | 172359526 | 172359680 | PDFSTATSENSEMBLUCSC | 155 | 4 | 31838 | gene | 0.05714 | 0.39737 | Not queried | 2.63 | 1.14e-01 | 1.00e+00 |
|  | 11 | 31825605 | 31825969 | PDFSTATSENSEMBLUCSC | 365 | 5 | 6542 | gene;island | 0.39536 | 0.09246 | Not queried | 2.63 | 1.59e-02 | 1.00e+00 |
|  | X | 99666164 | 99666486 | PDFSTATSENSEMBLUCSC | 323 | 4 | 41415 | tss;island | 0.15456 | 0.55665 | Not queried | 2.63 | 2.86e-02 | 1.00e+00 |
|  | 16 | 3243304 | 3243552 | PDFSTATSENSEMBLUCSC | 249 | 4 | 15610 | island | 0.05447 | 0.25251 | Not queried | 2.62 | 2.86e-02 | 1.00e+00 |
|  | 1 | 229569877 | 229570288 | PDFSTATSENSEMBLUCSC | 412 | 11 | 2307 | tss | 0.06065 | 0.44192 | Not queried | 2.62 | 3.40e-05 | 1.75e-01 |
|  | 2 | 97215789 | 97216131 | PDFSTATSENSEMBLUCSC | 343 | 4 | 22745 | gene;island | 0.16671 | 0.53502 | Not queried | 2.62 | 3.43e-01 | 1.00e+00 |
|  | 1 | 248020331 | 248020436 | PDFSTATSENSEMBLUCSC | 106 | 4 | 2367 | tss | 0.02169 | 0.05460 | Not queried | 2.62 | 3.43e-01 | 1.00e+00 |
|  | 7 | 158362729 | 158362966 | PDFSTATSENSEMBLUCSC | 238 | 4 | 37314 | gene;island | 0.61639 | 0.93220 | Not queried | 2.62 | 5.71e-02 | 1.00e+00 |
|  | 17 | 40346279 | 40346486 | PDFSTATSENSEMBLUCSC | 208 | 5 | 16543 | gene | 0.07344 | 0.33293 | Not queried | 2.62 | 5.56e-02 | 1.00e+00 |
|  | 2 | 165698673 | 165698949 | PDFSTATSENSEMBLUCSC | 277 | 4 | 23655 | tss;island | 0.03940 | 0.16136 | Not queried | 2.62 | 2.00e-01 | 1.00e+00 |
|  | 19 | 58095424 | 58095595 | PDFSTATSENSEMBLUCSC | 172 | 7 | 21083 | tss | 0.01865 | 0.08423 | Not queried | 2.62 | 1.75e-02 | 1.00e+00 |
|  | 6 | 170101455 | 170101549 | PDFSTATSENSEMBLUCSC | 95 | 4 | 34509 | gene;tss;island | 0.10081 | 0.02841 | Not queried | 2.62 | 1.14e-01 | 1.00e+00 |
|  | 2 | 71134524 | 71134936 | PDFSTATSENSEMBLUCSC | 413 | 6 | 22689 | gene;island | 0.69157 | 0.32988 | Not queried | 2.62 | 2.16e-03 | 1.00e+00 |
|  | 5 | 126409198 | 126409573 | PDFSTATSENSEMBLUCSC | 376 | 9 | 32196 | tss | 0.46267 | 0.90039 | Not queried | 2.62 | 5.64e-03 | 1.00e+00 |
|  | 5 | 179499304 | 179499488 | PDFSTATSENSEMBLUCSC | 185 | 4 | 32498 | tss | 0.15702 | 0.05307 | Not queried | 2.62 | 6.86e-01 | 1.00e+00 |
|  | 17 | 46667587 | 46667812 | PDFSTATSENSEMBLUCSC | 226 | 6 | 17429 | tss;island | 0.82053 | 0.42657 | Not queried | 2.62 | 2.60e-02 | 1.00e+00 |
|  | 11 | 72928926 | 72929553 | PDFSTATSENSEMBLUCSC | 628 | 13 | 8616 | island | 0.12276 | 0.02930 | Not queried | 2.62 | 1.91e-02 | 1.00e+00 |
|  | 3 | 157821919 | 157822072 | PDFSTATSENSEMBLUCSC | 154 | 4 | 27968 | gene;island | 0.56315 | 0.16996 | Not queried | 2.62 | 2.86e-02 | 1.00e+00 |
|  | 19 | 35981371 | 35981677 | PDFSTATSENSEMBLUCSC | 307 | 5 | 20668 | tss | 0.11393 | 0.44788 | Not queried | 2.62 | 7.94e-03 | 1.00e+00 |
|  | 7 | 16505589 | 16505664 | PDFSTATSENSEMBLUCSC | 76 | 5 | 37387 | tss | 0.02978 | 0.07647 | Not queried | 2.62 | 3.17e-02 | 1.00e+00 |
|  | 1 | 1475644 | 1475742 | PDFSTATSENSEMBLUCSC | 99 | 4 | 2440 | island | 0.22353 | 0.62716 | Not queried | 2.62 | 2.86e-02 | 1.00e+00 |
|  | X | 10851838 | 10852037 | PDFSTATSENSEMBLUCSC | 200 | 4 | 41181 | tss | 0.03401 | 0.13852 | Not queried | 2.62 | 4.86e-01 | 1.00e+00 |
|  | 2 | 176988203 | 176988580 | PDFSTATSENSEMBLUCSC | 378 | 5 | 22900 | gene;island | 0.02211 | 0.10006 | Not queried | 2.61 | 7.94e-03 | 1.00e+00 |
|  | 1 | 221060753 | 221061254 | PDFSTATSENSEMBLUCSC | 502 | 6 | 4211 | island | 0.14249 | 0.44076 | Not queried | 2.61 | 2.16e-03 | 1.00e+00 |
|  | 7 | 135413023 | 135413232 | PDFSTATSENSEMBLUCSC | 210 | 5 | 37855 | tss | 0.30948 | 0.74317 | Not queried | 2.61 | 7.94e-03 | 1.00e+00 |
|  | 5 | 1386363 | 1386887 | PDFSTATSENSEMBLUCSC | 525 | 5 | 32567 | island | 0.12131 | 0.36980 | Not queried | 2.61 | 9.52e-02 | 1.00e+00 |
|  | 20 | 61638420 | 61638588 | PDFSTATSENSEMBLUCSC | 169 | 7 | 25848 | tss;island | 0.12671 | 0.53513 | Not queried | 2.61 | 5.83e-04 | 1.00e+00 |
|  | 1 | 209825672 | 209825747 | PDFSTATSENSEMBLUCSC | 76 | 4 | 982 | gene | 0.52348 | 0.88214 | Not queried | 2.61 | 5.71e-02 | 1.00e+00 |
|  | 10 | 50339928 | 50340173 | PDFSTATSENSEMBLUCSC | 246 | 4 | 4569 | gene;island | 0.39237 | 0.81822 | Not queried | 2.61 | 2.86e-02 | 1.00e+00 |
|  | 6 | 32725075 | 32725438 | PDFSTATSENSEMBLUCSC | 364 | 5 | 34065 | gene | 0.78894 | 0.44975 | Not queried | 2.61 | 7.94e-03 | 1.00e+00 |
|  | 12 | 56329615 | 56329903 | PDFSTATSENSEMBLUCSC | 289 | 4 | 9145 | gene;island | 0.81141 | 0.41811 | Not queried | 2.61 | 2.86e-02 | 1.00e+00 |
|  | 11 | 75379491 | 75379624 | PDFSTATSENSEMBLUCSC | 134 | 6 | 7549 | tss | 0.04141 | 0.21559 | Not queried | 2.60 | 4.33e-03 | 1.00e+00 |
|  | 1 | 116184219 | 116184423 | PDFSTATSENSEMBLUCSC | 205 | 4 | 1855 | tss;island | 0.65358 | 0.22357 | Not queried | 2.60 | 2.00e-01 | 1.00e+00 |
|  | 6 | 167763758 | 167764146 | PDFSTATSENSEMBLUCSC | 389 | 4 | 36581 | island | 0.20671 | 0.53472 | Not queried | 2.60 | 2.86e-02 | 1.00e+00 |
|  | 13 | 112984478 | 112984976 | PDFSTATSENSEMBLUCSC | 499 | 5 | 11889 | island | 0.50140 | 0.92124 | Not queried | 2.60 | 9.52e-02 | 1.00e+00 |
|  | 7 | 27199726 | 27199956 | PDFSTATSENSEMBLUCSC | 231 | 5 | 38291 | island | 0.59461 | 0.96071 | Not queried | 2.60 | 7.94e-03 | 1.00e+00 |
|  | X | 70151117 | 70151209 | PDFSTATSENSEMBLUCSC | 93 | 4 | 41370 | tss | 0.09005 | 0.01658 | Not queried | 2.60 | 2.86e-02 | 1.00e+00 |
|  | 7 | 128809100 | 128809442 | PDFSTATSENSEMBLUCSC | 343 | 5 | 38843 | island | 0.02275 | 0.25150 | Not queried | 2.60 | 7.94e-03 | 1.00e+00 |
|  | 11 | 74022742 | 74023050 | PDFSTATSENSEMBLUCSC | 309 | 7 | 7535 | tss | 0.52251 | 0.15324 | Not queried | 2.60 | 7.28e-02 | 1.00e+00 |
|  | 17 | 72321767 | 72322154 | PDFSTATSENSEMBLUCSC | 388 | 5 | 17590 | tss;island | 0.71782 | 0.23147 | Not queried | 2.59 | 6.90e-01 | 1.00e+00 |
|  | 12 | 7033470 | 7033628 | PDFSTATSENSEMBLUCSC | 159 | 5 | 10193 | island | 0.01721 | 0.05392 | Not queried | 2.59 | 2.22e-01 | 1.00e+00 |
|  | 3 | 150690183 | 150690585 | PDFSTATSENSEMBLUCSC | 403 | 7 | 27954 | gene | 0.12562 | 0.55476 | Not queried | 2.59 | 2.62e-02 | 1.00e+00 |
|  | X | 48334240 | 48334722 | PDFSTATSENSEMBLUCSC | 483 | 10 | 41760 | island | 0.04654 | 0.01515 | Not queried | 2.59 | 7.25e-04 | 1.00e+00 |
|  | 3 | 40518669 | 40518798 | PDFSTATSENSEMBLUCSC | 130 | 4 | 27686 | gene | 0.01446 | 0.08173 | Not queried | 2.59 | 2.00e-01 | 1.00e+00 |
|  | 6 | 32158153 | 32158513 | PDFSTATSENSEMBLUCSC | 361 | 9 | 34806 | tss | 0.06514 | 0.27846 | Not queried | 2.59 | 3.99e-03 | 1.00e+00 |
|  | 17 | 80279310 | 80279675 | PDFSTATSENSEMBLUCSC | 366 | 5 | 16909 | gene;island | 0.08994 | 0.44891 | Not queried | 2.59 | 2.22e-01 | 1.00e+00 |
|  | 3 | 114102565 | 114102863 | PDFSTATSENSEMBLUCSC | 299 | 5 | 27858 | gene;tss | 0.10949 | 0.78366 | Not queried | 2.59 | 4.21e-01 | 1.00e+00 |
|  | 18 | 904851 | 905252 | PDFSTATSENSEMBLUCSC | 402 | 12 | 19123 | tss | 0.05403 | 0.23408 | Not queried | 2.58 | 4.96e-04 | 1.00e+00 |
|  | 12 | 54376019 | 54376241 | PDFSTATSENSEMBLUCSC | 223 | 4 | 10458 | island | 0.86941 | 0.35590 | Not queried | 2.58 | 5.71e-02 | 1.00e+00 |
|  | X | 48814806 | 48815125 | PDFSTATSENSEMBLUCSC | 320 | 8 | 40990 | gene;island | 0.39768 | 0.04124 | Not queried | 2.58 | 2.81e-02 | 1.00e+00 |
|  | 11 | 68748461 | 68748584 | PDFSTATSENSEMBLUCSC | 124 | 4 | 7493 | tss | 0.33436 | 0.62509 | Not queried | 2.58 | 5.71e-02 | 1.00e+00 |
|  | 15 | 83317281 | 83317632 | PDFSTATSENSEMBLUCSC | 352 | 5 | 13830 | tss;island | 0.84999 | 0.43373 | Not queried | 2.58 | 3.17e-02 | 1.00e+00 |
|  | 17 | 33700745 | 33700817 | PDFSTATSENSEMBLUCSC | 73 | 6 | 17234 | tss;island | 0.01524 | 0.11487 | Not queried | 2.58 | 2.60e-02 | 1.00e+00 |
|  | X | 25040609 | 25041258 | PDFSTATSENSEMBLUCSC | 650 | 6 | 41695 | island | 0.19412 | 0.09328 | Not queried | 2.58 | 2.40e-01 | 1.00e+00 |
|  | 14 | 56045827 | 56046098 | PDFSTATSENSEMBLUCSC | 272 | 4 | 12061 | gene;tss;island | 0.03938 | 0.01064 | Not queried | 2.57 | 1.14e-01 | 1.00e+00 |
|  | 5 | 78281734 | 78282099 | PDFSTATSENSEMBLUCSC | 366 | 6 | 31602 | gene;island | 0.44345 | 0.11735 | Not queried | 2.57 | 4.11e-02 | 1.00e+00 |
|  | 17 | 4544925 | 4545157 | PDFSTATSENSEMBLUCSC | 233 | 5 | 17840 | island | 0.59325 | 0.15165 | Not queried | 2.57 | 1.51e-01 | 1.00e+00 |
|  | 6 | 29555799 | 29556415 | PDFSTATSENSEMBLUCSC | 617 | 8 | 33599 | gene | 0.34332 | 0.70904 | Not queried | 2.57 | 1.55e-04 | 7.99e-01 |
|  | 13 | 29394754 | 29394960 | PDFSTATSENSEMBLUCSC | 207 | 4 | 11615 | island | 0.02706 | 0.15357 | Not queried | 2.57 | 1.14e-01 | 1.00e+00 |
|  | 12 | 9217390 | 9218039 | PDFSTATSENSEMBLUCSC | 650 | 11 | 10209 | island | 0.53775 | 0.83036 | Not queried | 2.57 | 8.51e-05 | 4.37e-01 |
|  | 11 | 14280742 | 14281053 | PDFSTATSENSEMBLUCSC | 312 | 4 | 6494 | gene;island | 0.25851 | 0.59398 | Not queried | 2.56 | 2.86e-02 | 1.00e+00 |
|  | 10 | 91597207 | 91597593 | PDFSTATSENSEMBLUCSC | 387 | 4 | 5908 | island | 0.55982 | 0.88920 | Not queried | 2.56 | 2.86e-02 | 1.00e+00 |
|  | 3 | 45635931 | 45636386 | PDFSTATSENSEMBLUCSC | 456 | 7 | 29005 | island | 0.07429 | 0.32168 | Not queried | 2.56 | 1.11e-02 | 1.00e+00 |
|  | 22 | 18632404 | 18633123 | PDFSTATSENSEMBLUCSC | 720 | 10 | 27243 | island | 0.02580 | 0.19296 | Not queried | 2.56 | 5.24e-02 | 1.00e+00 |
|  | 8 | 144462007 | 144462235 | PDFSTATSENSEMBLUCSC | 229 | 4 | 39412 | gene;island | 0.28101 | 0.77243 | Not queried | 2.56 | 2.00e-01 | 1.00e+00 |
|  | 2 | 25390385 | 25390540 | PDFSTATSENSEMBLUCSC | 156 | 4 | 22565 | gene;island | 0.80663 | 0.41197 | Not queried | 2.56 | 5.71e-02 | 1.00e+00 |
|  | 19 | 50553682 | 50553907 | PDFSTATSENSEMBLUCSC | 226 | 5 | 20935 | tss | 0.02432 | 0.07327 | Not queried | 2.56 | 9.52e-02 | 1.00e+00 |
|  | 22 | 44287876 | 44288155 | PDFSTATSENSEMBLUCSC | 280 | 6 | 27164 | tss | 0.53104 | 0.21136 | Not queried | 2.56 | 4.33e-03 | 1.00e+00 |
|  | 22 | 44287772 | 44288155 | PDFSTATSENSEMBLUCSC | 384 | 8 | 27518 | island | 0.47535 | 0.20073 | Not queried | 2.56 | 1.86e-03 | 1.00e+00 |
|  | 19 | 1992866 | 1993265 | PDFSTATSENSEMBLUCSC | 400 | 5 | 19639 | gene;island | 0.37905 | 0.67047 | Not queried | 2.56 | 7.94e-03 | 1.00e+00 |
|  | 11 | 63258509 | 63258803 | PDFSTATSENSEMBLUCSC | 295 | 8 | 8347 | island | 0.71443 | 0.95024 | Not queried | 2.56 | 1.86e-03 | 1.00e+00 |
|  | 12 | 54346784 | 54347242 | PDFSTATSENSEMBLUCSC | 459 | 5 | 10454 | island | 0.02193 | 0.11659 | Not queried | 2.56 | 3.17e-02 | 1.00e+00 |
|  | 1 | 44456953 | 44457185 | PDFSTATSENSEMBLUCSC | 233 | 5 | 1566 | tss | 0.01582 | 0.02605 | Not queried | 2.56 | 3.10e-01 | 1.00e+00 |
|  | 3 | 195384522 | 195384752 | PDFSTATSENSEMBLUCSC | 231 | 6 | 28762 | tss;island | 0.26191 | 0.04549 | Not queried | 2.56 | 3.10e-01 | 1.00e+00 |
|  | 1 | 23696021 | 23696209 | PDFSTATSENSEMBLUCSC | 189 | 4 | 311 | gene;tss;island | 0.01068 | 0.06923 | Not queried | 2.56 | 2.86e-02 | 1.00e+00 |
|  | 7 | 45927735 | 45927947 | PDFSTATSENSEMBLUCSC | 213 | 5 | 37521 | tss | 0.22758 | 0.64353 | Not queried | 2.56 | 1.51e-01 | 1.00e+00 |
|  | 1 | 100111713 | 100111949 | PDFSTATSENSEMBLUCSC | 237 | 4 | 647 | gene | 0.26149 | 0.03159 | Not queried | 2.55 | 2.00e-01 | 1.00e+00 |
|  | 4 | 4864110 | 4864902 | PDFSTATSENSEMBLUCSC | 793 | 12 | 29874 | gene;island | 0.07361 | 0.57576 | Not queried | 2.55 | 3.32e-02 | 1.00e+00 |
|  | 5 | 76326040 | 76326465 | PDFSTATSENSEMBLUCSC | 426 | 6 | 32834 | island | 0.88918 | 0.46221 | Not queried | 2.55 | 2.16e-03 | 1.00e+00 |
|  | 16 | 23724821 | 23724943 | PDFSTATSENSEMBLUCSC | 123 | 5 | 15112 | tss | 0.75959 | 0.44472 | Not queried | 2.55 | 7.94e-03 | 1.00e+00 |
|  | 6 | 32076906 | 32077103 | PDFSTATSENSEMBLUCSC | 198 | 7 | 33988 | gene | 0.11972 | 0.43744 | Not queried | 2.55 | 5.83e-04 | 1.00e+00 |
|  | Y | 16636984 | 16637142 | PDFSTATSENSEMBLUCSC | 159 | 4 | 42161 | gene;island | 0.06858 | 0.32944 | Not queried | 2.55 | 2.00e-01 | 1.00e+00 |
|  | 3 | 46506104 | 46506374 | PDFSTATSENSEMBLUCSC | 271 | 4 | 27707 | gene | 0.64520 | 0.86676 | Not queried | 2.55 | 2.86e-02 | 1.00e+00 |
|  | 15 | 72476680 | 72477069 | PDFSTATSENSEMBLUCSC | 390 | 5 | 13378 | gene;island | 0.09520 | 0.46348 | Not queried | 2.55 | 3.17e-02 | 1.00e+00 |
|  | 6 | 32119874 | 32120324 | PDFSTATSENSEMBLUCSC | 451 | 7 | 34801 | tss | 0.24073 | 0.55778 | Not queried | 2.55 | 6.99e-03 | 1.00e+00 |
|  | 17 | 79495268 | 79495664 | PDFSTATSENSEMBLUCSC | 397 | 7 | 18894 | island | 0.33961 | 0.74464 | Not queried | 2.55 | 1.17e-03 | 1.00e+00 |
|  | 15 | 35087506 | 35087773 | PDFSTATSENSEMBLUCSC | 268 | 5 | 13252 | gene | 0.62360 | 0.84829 | Not queried | 2.55 | 7.94e-03 | 1.00e+00 |
|  | 2 | 74780172 | 74780617 | PDFSTATSENSEMBLUCSC | 446 | 6 | 22713 | gene;island | 0.63942 | 0.27504 | Not queried | 2.55 | 1.52e-02 | 1.00e+00 |
|  | 17 | 50236344 | 50236765 | PDFSTATSENSEMBLUCSC | 422 | 4 | 16662 | gene;tss;island | 0.19128 | 0.40675 | Not queried | 2.55 | 3.43e-01 | 1.00e+00 |
|  | 3 | 134369339 | 134369828 | PDFSTATSENSEMBLUCSC | 490 | 4 | 27915 | gene | 0.33691 | 0.08086 | Not queried | 2.55 | 4.86e-01 | 1.00e+00 |
|  | 19 | 45975694 | 45976195 | PDFSTATSENSEMBLUCSC | 502 | 5 | 20062 | gene;island | 0.21404 | 0.58513 | Not queried | 2.55 | 7.94e-03 | 1.00e+00 |
|  | 3 | 148847598 | 148847791 | PDFSTATSENSEMBLUCSC | 194 | 4 | 27949 | gene;island | 0.02256 | 0.07990 | Not queried | 2.55 | 5.71e-02 | 1.00e+00 |
|  | 1 | 26605913 | 26606110 | PDFSTATSENSEMBLUCSC | 198 | 4 | 1384 | tss;island | 0.29933 | 0.67607 | Not queried | 2.54 | 2.86e-02 | 1.00e+00 |
|  | 4 | 1983102 | 1983380 | PDFSTATSENSEMBLUCSC | 279 | 4 | 29835 | gene;island | 0.58711 | 0.93047 | Not queried | 2.54 | 5.71e-02 | 1.00e+00 |
|  | 6 | 29795350 | 29795595 | PDFSTATSENSEMBLUCSC | 246 | 9 | 33632 | gene;island | 0.35531 | 0.11712 | Not queried | 2.54 | 3.15e-02 | 1.00e+00 |
|  | 7 | 155165295 | 155165891 | PDFSTATSENSEMBLUCSC | 597 | 5 | 39000 | island | 0.03280 | 0.25412 | Not queried | 2.54 | 9.52e-02 | 1.00e+00 |
|  | 11 | 108422936 | 108423325 | PDFSTATSENSEMBLUCSC | 390 | 6 | 7652 | tss | 0.60180 | 0.75309 | Not queried | 2.54 | 4.11e-02 | 1.00e+00 |
|  | 1 | 36771714 | 36771844 | PDFSTATSENSEMBLUCSC | 131 | 4 | 1491 | tss;island | 0.05155 | 0.29117 | Not queried | 2.54 | 2.86e-02 | 1.00e+00 |
|  | 19 | 15162515 | 15162971 | PDFSTATSENSEMBLUCSC | 457 | 4 | 20517 | tss | 0.22445 | 0.44748 | Not queried | 2.54 | 1.14e-01 | 1.00e+00 |
|  | 6 | 155537901 | 155538155 | PDFSTATSENSEMBLUCSC | 255 | 7 | 34455 | gene;island | 0.15414 | 0.47430 | Not queried | 2.54 | 1.17e-03 | 1.00e+00 |
|  | 10 | 135333805 | 135334122 | PDFSTATSENSEMBLUCSC | 318 | 5 | 6348 | island | 0.72841 | 0.45169 | Not queried | 2.54 | 9.52e-02 | 1.00e+00 |
|  | 1 | 1935289 | 1935561 | PDFSTATSENSEMBLUCSC | 273 | 4 | 1162 | tss | 0.86119 | 0.48209 | Not queried | 2.53 | 2.86e-02 | 1.00e+00 |
|  | 1 | 1935048 | 1935561 | PDFSTATSENSEMBLUCSC | 514 | 6 | 2463 | island | 0.86119 | 0.43310 | Not queried | 2.53 | 2.16e-03 | 1.00e+00 |
|  | 20 | 5485245 | 5485511 | PDFSTATSENSEMBLUCSC | 267 | 7 | 25544 | tss | 0.19804 | 0.07416 | Not queried | 2.53 | 5.30e-02 | 1.00e+00 |
|  | 3 | 127316944 | 127317209 | PDFSTATSENSEMBLUCSC | 266 | 6 | 28521 | tss | 0.19389 | 0.03698 | Not queried | 2.53 | 4.33e-03 | 1.00e+00 |
|  | 12 | 34500585 | 34500813 | PDFSTATSENSEMBLUCSC | 229 | 4 | 10296 | island | 0.07727 | 0.21200 | Not queried | 2.53 | 4.86e-01 | 1.00e+00 |
|  | 5 | 140480507 | 140480967 | PDFSTATSENSEMBLUCSC | 461 | 6 | 31750 | gene;island | 0.37920 | 0.70780 | Not queried | 2.53 | 2.16e-03 | 1.00e+00 |
|  | 11 | 126872615 | 126872863 | PDFSTATSENSEMBLUCSC | 249 | 4 | 8912 | island | 0.59519 | 0.14055 | Not queried | 2.53 | 2.00e-01 | 1.00e+00 |
|  | 1 | 246952048 | 246952508 | PDFSTATSENSEMBLUCSC | 461 | 6 | 2360 | tss;island | 0.59612 | 0.87916 | Not queried | 2.53 | 6.49e-02 | 1.00e+00 |
|  | 4 | 140656749 | 140657110 | PDFSTATSENSEMBLUCSC | 362 | 4 | 30086 | gene;island | 0.09267 | 0.47270 | Not queried | 2.53 | 2.00e-01 | 1.00e+00 |
|  | 1 | 68299388 | 68299511 | PDFSTATSENSEMBLUCSC | 124 | 5 | 1682 | tss;island | 0.05163 | 0.20746 | Not queried | 2.53 | 1.59e-02 | 1.00e+00 |
|  | 3 | 120004186 | 120004483 | PDFSTATSENSEMBLUCSC | 298 | 4 | 29345 | island | 0.23726 | 0.08853 | Not queried | 2.53 | 2.00e-01 | 1.00e+00 |
|  | 1 | 150947403 | 150947589 | PDFSTATSENSEMBLUCSC | 187 | 5 | 3720 | island | 0.21176 | 0.04575 | Not queried | 2.53 | 3.10e-01 | 1.00e+00 |
|  | 2 | 202097062 | 202097173 | PDFSTATSENSEMBLUCSC | 112 | 4 | 23778 | tss | 0.01157 | 0.06313 | Not queried | 2.53 | 2.00e-01 | 1.00e+00 |
|  | X | 46432770 | 46433132 | PDFSTATSENSEMBLUCSC | 363 | 5 | 41272 | tss;island | 0.09311 | 0.02971 | Not queried | 2.53 | 3.10e-01 | 1.00e+00 |
|  | 17 | 38183370 | 38183790 | PDFSTATSENSEMBLUCSC | 421 | 5 | 16522 | gene;tss | 0.18029 | 0.66116 | Not queried | 2.53 | 9.52e-02 | 1.00e+00 |
|  | 17 | 21279561 | 21279696 | PDFSTATSENSEMBLUCSC | 136 | 5 | 17170 | tss | 0.24710 | 0.06580 | Not queried | 2.53 | 3.17e-02 | 1.00e+00 |
|  | 1 | 26606846 | 26606912 | PDFSTATSENSEMBLUCSC | 67 | 4 | 337 | gene;island | 0.04874 | 0.25626 | Not queried | 2.53 | 2.86e-02 | 1.00e+00 |
|  | 17 | 16119344 | 16119456 | PDFSTATSENSEMBLUCSC | 113 | 4 | 17121 | tss;island | 0.11223 | 0.02982 | Not queried | 2.53 | 2.86e-02 | 1.00e+00 |
|  | X | 118356756 | 118357181 | PDFSTATSENSEMBLUCSC | 426 | 5 | 41980 | island | 0.96018 | 0.60308 | Not queried | 2.53 | 3.10e-01 | 1.00e+00 |
|  | 9 | 93564028 | 93564603 | PDFSTATSENSEMBLUCSC | 576 | 5 | 40708 | gene;island | 0.02771 | 0.04389 | Not queried | 2.52 | 5.48e-01 | 1.00e+00 |
|  | 1 | 26487591 | 26488129 | PDFSTATSENSEMBLUCSC | 539 | 5 | 336 | gene;island | 0.33330 | 0.08003 | Not queried | 2.52 | 2.22e-01 | 1.00e+00 |
|  | 18 | 2913135 | 2913254 | PDFSTATSENSEMBLUCSC | 120 | 4 | 19003 | gene;island | 0.63912 | 0.92350 | Not queried | 2.52 | 2.86e-02 | 1.00e+00 |
|  | X | 48397520 | 48398048 | PDFSTATSENSEMBLUCSC | 529 | 12 | 41292 | tss | 0.19150 | 0.57702 | Not queried | 2.52 | 1.43e-03 | 1.00e+00 |
|  | 10 | 101287609 | 101287846 | PDFSTATSENSEMBLUCSC | 238 | 5 | 5991 | island | 0.42003 | 0.08319 | Not queried | 2.52 | 9.52e-02 | 1.00e+00 |
|  | 1 | 2344979 | 2345475 | PDFSTATSENSEMBLUCSC | 497 | 11 | 1170 | tss;island | 0.55131 | 0.16669 | PEX10 (margin: RER1; PLCH2; PEX10) | 2.52 | 2.84e-06 | 1.46e-02 |
|  | 20 | 30028062 | 30028292 | PDFSTATSENSEMBLUCSC | 231 | 6 | 25618 | tss | 0.32576 | 0.59444 | Not queried | 2.52 | 2.60e-02 | 1.00e+00 |
|  | 22 | 46067363 | 46067586 | PDFSTATSENSEMBLUCSC | 224 | 4 | 27182 | tss;island | 0.52613 | 0.14146 | Not queried | 2.52 | 2.00e-01 | 1.00e+00 |
|  | 17 | 72920176 | 72920385 | PDFSTATSENSEMBLUCSC | 210 | 9 | 17603 | tss;island | 0.07928 | 0.43956 | Not queried | 2.52 | 2.44e-02 | 1.00e+00 |
|  | 14 | 60977856 | 60978275 | PDFSTATSENSEMBLUCSC | 420 | 5 | 12075 | gene;island | 0.15048 | 0.02404 | Not queried | 2.52 | 1.51e-01 | 1.00e+00 |
|  | 7 | 150211761 | 150211939 | PDFSTATSENSEMBLUCSC | 179 | 5 | 37917 | tss | 0.38586 | 0.81228 | Not queried | 2.52 | 3.17e-02 | 1.00e+00 |
|  | 12 | 124864528 | 124864681 | PDFSTATSENSEMBLUCSC | 154 | 4 | 9366 | gene | 0.61106 | 0.90440 | Not queried | 2.52 | 2.86e-02 | 1.00e+00 |
|  | 3 | 48647215 | 48647426 | PDFSTATSENSEMBLUCSC | 212 | 5 | 28260 | tss | 0.08956 | 0.02244 | Not queried | 2.52 | 2.22e-01 | 1.00e+00 |
|  | 5 | 134872610 | 134872659 | PDFSTATSENSEMBLUCSC | 50 | 4 | 32247 | tss;island | 0.91552 | 0.55534 | Not queried | 2.52 | 2.86e-02 | 1.00e+00 |
|  | 15 | 77577491 | 77577655 | PDFSTATSENSEMBLUCSC | 165 | 4 | 13797 | tss | 0.74800 | 0.94791 | Not queried | 2.52 | 2.00e-01 | 1.00e+00 |
|  | 20 | 36148133 | 36149455 | PDFSTATSENSEMBLUCSC | 1323 | 41 | 25378 | gene;tss;island | 0.93272 | 0.67052 | NNAT; BLCAP (margin: NNAT; PPIAP3; RP11-425M5.5; BLCAP) | 2.52 | 3.32e-07 | 1.71e-03 |
|  | 6 | 29527870 | 29528119 | PDFSTATSENSEMBLUCSC | 250 | 4 | 34684 | tss | 0.57967 | 0.88774 | Not queried | 2.52 | 2.86e-02 | 1.00e+00 |
|  | 18 | 904851 | 905611 | PDFSTATSENSEMBLUCSC | 761 | 15 | 19317 | island | 0.06583 | 0.31619 | Not queried | 2.51 | 1.74e-04 | 8.95e-01 |
|  | 6 | 43478455 | 43478829 | PDFSTATSENSEMBLUCSC | 375 | 8 | 34935 | tss;island | 0.51589 | 0.86438 | Not queried | 2.51 | 2.07e-02 | 1.00e+00 |
|  | 1 | 155247476 | 155247785 | PDFSTATSENSEMBLUCSC | 310 | 4 | 797 | gene | 0.02598 | 0.09077 | Not queried | 2.51 | 5.71e-02 | 1.00e+00 |
|  | 8 | 72757787 | 72758095 | PDFSTATSENSEMBLUCSC | 309 | 4 | 39700 | tss;island | 0.06723 | 0.22663 | Not queried | 2.51 | 2.00e-01 | 1.00e+00 |
|  | 11 | 46366643 | 46367100 | PDFSTATSENSEMBLUCSC | 458 | 8 | 6580 | gene;island | 0.20168 | 0.60332 | Not queried | 2.51 | 6.22e-04 | 1.00e+00 |
|  | 6 | 29794503 | 29794843 | PDFSTATSENSEMBLUCSC | 341 | 4 | 35570 | island | 0.77761 | 0.38422 | Not queried | 2.51 | 1.14e-01 | 1.00e+00 |
|  | 4 | 3471816 | 3472246 | PDFSTATSENSEMBLUCSC | 431 | 4 | 29861 | gene;island | 0.68693 | 0.92626 | Not queried | 2.51 | 2.86e-02 | 1.00e+00 |
|  | 1 | 6526309 | 6526583 | PDFSTATSENSEMBLUCSC | 275 | 7 | 1203 | tss | 0.31933 | 0.75345 | Not queried | 2.51 | 1.28e-01 | 1.00e+00 |
|  | 1 | 13881331 | 13881656 | PDFSTATSENSEMBLUCSC | 326 | 5 | 2699 | island | 0.96367 | 0.82873 | Not queried | 2.51 | 9.52e-02 | 1.00e+00 |
|  | X | 48768632 | 48769107 | PDFSTATSENSEMBLUCSC | 476 | 10 | 40989 | gene;island | 0.16511 | 0.59385 | Not queried | 2.51 | 1.08e-05 | 5.56e-02 |
|  | X | 70288026 | 70288735 | PDFSTATSENSEMBLUCSC | 710 | 12 | 41855 | island | 0.17071 | 0.02416 | Not queried | 2.51 | 1.11e-03 | 1.00e+00 |
|  | 20 | 54580070 | 54580196 | PDFSTATSENSEMBLUCSC | 127 | 4 | 25793 | tss;island | 0.09448 | 0.36321 | Not queried | 2.50 | 1.14e-01 | 1.00e+00 |
|  | X | 75648802 | 75648992 | PDFSTATSENSEMBLUCSC | 191 | 4 | 41038 | gene;island | 0.36253 | 0.13080 | Not queried | 2.50 | 2.86e-02 | 1.00e+00 |
|  | 17 | 6616799 | 6617192 | PDFSTATSENSEMBLUCSC | 394 | 6 | 17018 | tss | 0.25153 | 0.05083 | Not queried | 2.50 | 1.32e-01 | 1.00e+00 |
|  | 17 | 46806445 | 46806935 | PDFSTATSENSEMBLUCSC | 491 | 6 | 17439 | tss;island | 0.06590 | 0.28194 | Not queried | 2.50 | 4.11e-02 | 1.00e+00 |
|  | 11 | 22454152 | 22454661 | PDFSTATSENSEMBLUCSC | 510 | 6 | 8113 | island | 0.32240 | 0.65678 | Not queried | 2.50 | 2.16e-03 | 1.00e+00 |
|  | 8 | 144099482 | 144099719 | PDFSTATSENSEMBLUCSC | 238 | 4 | 39408 | gene | 0.05663 | 0.42055 | Not queried | 2.50 | 2.00e-01 | 1.00e+00 |
|  | 22 | 48027641 | 48027921 | PDFSTATSENSEMBLUCSC | 281 | 4 | 27557 | island | 0.63995 | 0.90494 | Not queried | 2.50 | 2.00e-01 | 1.00e+00 |
|  | 3 | 44596178 | 44596684 | PDFSTATSENSEMBLUCSC | 507 | 8 | 28214 | tss | 0.90228 | 0.63094 | Not queried | 2.50 | 1.05e-01 | 1.00e+00 |
|  | 6 | 85473171 | 85473344 | PDFSTATSENSEMBLUCSC | 174 | 4 | 34339 | gene;island | 0.02836 | 0.14659 | Not queried | 2.50 | 2.86e-02 | 1.00e+00 |
|  | 2 | 23850319 | 23850586 | PDFSTATSENSEMBLUCSC | 268 | 4 | 22558 | gene;island | 0.61965 | 0.88600 | Not queried | 2.50 | 1.14e-01 | 1.00e+00 |
|  | 15 | 53097649 | 53097840 | PDFSTATSENSEMBLUCSC | 192 | 4 | 14126 | island | 0.56687 | 0.14359 | Not queried | 2.50 | 2.00e-01 | 1.00e+00 |
|  | 6 | 30954872 | 30955142 | PDFSTATSENSEMBLUCSC | 271 | 4 | 33782 | gene | 0.78384 | 0.42916 | Not queried | 2.50 | 2.86e-02 | 1.00e+00 |
|  | 3 | 170303540 | 170304045 | PDFSTATSENSEMBLUCSC | 506 | 7 | 29614 | island | 0.46919 | 0.15174 | Not queried | 2.50 | 7.28e-02 | 1.00e+00 |
|  | 6 | 151646540 | 151647133 | PDFSTATSENSEMBLUCSC | 594 | 9 | 34444 | gene;island | 0.24827 | 0.03246 | Not queried | 2.50 | 1.65e-04 | 8.46e-01 |
|  | 5 | 179004535 | 179004757 | PDFSTATSENSEMBLUCSC | 223 | 4 | 31875 | gene;island | 0.79713 | 0.97856 | Not queried | 2.50 | 2.00e-01 | 1.00e+00 |
|  | 3 | 48936456 | 48936584 | PDFSTATSENSEMBLUCSC | 129 | 4 | 28267 | tss | 0.03937 | 0.19524 | Not queried | 2.50 | 2.00e-01 | 1.00e+00 |
|  | 5 | 114598574 | 114599159 | PDFSTATSENSEMBLUCSC | 586 | 10 | 32165 | tss | 0.03510 | 0.03632 | Not queried | 2.50 | 2.47e-01 | 1.00e+00 |
|  | 16 | 67219152 | 67219218 | PDFSTATSENSEMBLUCSC | 67 | 4 | 14762 | gene;tss;island | 0.34531 | 0.74502 | Not queried | 2.49 | 1.14e-01 | 1.00e+00 |
|  | 3 | 186334993 | 186335202 | PDFSTATSENSEMBLUCSC | 210 | 4 | 28031 | gene | 0.03126 | 0.08836 | Not queried | 2.49 | 1.14e-01 | 1.00e+00 |
|  | X | 48694012 | 48694446 | PDFSTATSENSEMBLUCSC | 435 | 5 | 41296 | tss;island | 0.57779 | 0.05604 | Not queried | 2.49 | 2.22e-01 | 1.00e+00 |
|  | 11 | 134176598 | 134176888 | PDFSTATSENSEMBLUCSC | 291 | 5 | 6976 | gene | 0.53531 | 0.73401 | Not queried | 2.49 | 5.56e-02 | 1.00e+00 |
|  | 11 | 705692 | 706100 | PDFSTATSENSEMBLUCSC | 409 | 7 | 7004 | tss;island | 0.20775 | 0.59601 | Not queried | 2.49 | 5.30e-02 | 1.00e+00 |
|  | 3 | 186080015 | 186080359 | PDFSTATSENSEMBLUCSC | 345 | 8 | 29705 | island | 0.06807 | 0.34839 | Not queried | 2.49 | 3.11e-04 | 1.00e+00 |
|  | 12 | 132910456 | 132910801 | PDFSTATSENSEMBLUCSC | 346 | 4 | 11015 | island | 0.56082 | 0.88948 | Not queried | 2.49 | 5.71e-02 | 1.00e+00 |
|  | 14 | 38091400 | 38092175 | PDFSTATSENSEMBLUCSC | 776 | 7 | 12772 | island | 0.31726 | 0.62333 | Not queried | 2.49 | 2.33e-03 | 1.00e+00 |
|  | 12 | 120241760 | 120242513 | PDFSTATSENSEMBLUCSC | 754 | 9 | 9317 | gene;island | 0.39653 | 0.55085 | Not queried | 2.49 | 2.58e-01 | 1.00e+00 |
|  | 6 | 27782909 | 27783331 | PDFSTATSENSEMBLUCSC | 423 | 5 | 34657 | tss;island | 0.21893 | 0.62794 | Not queried | 2.49 | 5.56e-02 | 1.00e+00 |
|  | 7 | 89748633 | 89748686 | PDFSTATSENSEMBLUCSC | 54 | 6 | 37629 | tss | 0.12516 | 0.35031 | Not queried | 2.49 | 2.16e-03 | 1.00e+00 |
|  | 1 | 247694271 | 247694557 | PDFSTATSENSEMBLUCSC | 287 | 5 | 1124 | gene;tss;island | 0.67765 | 0.95265 | Not queried | 2.48 | 3.17e-02 | 1.00e+00 |
|  | 6 | 32810551 | 32810833 | PDFSTATSENSEMBLUCSC | 283 | 5 | 34085 | gene;island | 0.91451 | 0.68226 | Not queried | 2.48 | 5.56e-02 | 1.00e+00 |
|  | 5 | 134073430 | 134073579 | PDFSTATSENSEMBLUCSC | 150 | 5 | 32237 | tss;island | 0.04936 | 0.01491 | Not queried | 2.48 | 5.56e-02 | 1.00e+00 |
|  | 10 | 102731747 | 102732224 | PDFSTATSENSEMBLUCSC | 478 | 7 | 5282 | tss | 0.69153 | 0.78615 | Not queried | 2.48 | 1.28e-01 | 1.00e+00 |
|  | 6 | 32115964 | 32116317 | PDFSTATSENSEMBLUCSC | 354 | 10 | 35837 | island | 0.31274 | 0.68069 | Not queried | 2.48 | 8.93e-03 | 1.00e+00 |
|  | 17 | 79026049 | 79026354 | PDFSTATSENSEMBLUCSC | 306 | 5 | 16856 | gene;island | 0.62831 | 0.80027 | Not queried | 2.48 | 7.94e-03 | 1.00e+00 |
|  | 17 | 3438857 | 3439372 | PDFSTATSENSEMBLUCSC | 516 | 7 | 16299 | gene;island | 0.06999 | 0.33922 | Not queried | 2.48 | 7.10e-01 | 1.00e+00 |
|  | 18 | 21852313 | 21852436 | PDFSTATSENSEMBLUCSC | 124 | 5 | 19178 | tss | 0.01600 | 0.08335 | Not queried | 2.48 | 7.94e-03 | 1.00e+00 |
|  | 11 | 58673453 | 58673615 | PDFSTATSENSEMBLUCSC | 163 | 4 | 8276 | island | 0.03908 | 0.19582 | Not queried | 2.48 | 2.86e-02 | 1.00e+00 |
|  | 3 | 147131069 | 147131332 | PDFSTATSENSEMBLUCSC | 264 | 4 | 27948 | gene;island | 0.73082 | 0.28735 | Not queried | 2.48 | 2.86e-02 | 1.00e+00 |
|  | 15 | 83952722 | 83953068 | PDFSTATSENSEMBLUCSC | 347 | 5 | 13430 | gene;island | 0.02655 | 0.11179 | Not queried | 2.48 | 2.22e-01 | 1.00e+00 |
|  | 12 | 52652220 | 52652742 | PDFSTATSENSEMBLUCSC | 523 | 5 | 10413 | island | 0.58485 | 0.92847 | Not queried | 2.48 | 7.94e-03 | 1.00e+00 |
|  | 1 | 2461820 | 2461929 | PDFSTATSENSEMBLUCSC | 110 | 6 | 1172 | tss;island | 0.27252 | 0.06492 | Not queried | 2.48 | 4.33e-03 | 1.00e+00 |
|  | 10 | 18549378 | 18549536 | PDFSTATSENSEMBLUCSC | 159 | 5 | 4957 | tss | 0.30040 | 0.73956 | Not queried | 2.48 | 9.52e-02 | 1.00e+00 |
|  | 8 | 70984199 | 70984559 | PDFSTATSENSEMBLUCSC | 361 | 4 | 39694 | tss;island | 0.12126 | 0.03570 | Not queried | 2.48 | 2.86e-02 | 1.00e+00 |
|  | 2 | 187558355 | 187558598 | PDFSTATSENSEMBLUCSC | 244 | 5 | 23736 | tss | 0.28529 | 0.04401 | Not queried | 2.47 | 3.10e-01 | 1.00e+00 |
|  | 2 | 135476601 | 135477056 | PDFSTATSENSEMBLUCSC | 456 | 8 | 23604 | tss;island | 0.22850 | 0.04060 | Not queried | 2.47 | 1.61e-01 | 1.00e+00 |
|  | 17 | 29886672 | 29886890 | PDFSTATSENSEMBLUCSC | 219 | 4 | 17215 | tss | 0.35532 | 0.71197 | Not queried | 2.47 | 2.86e-02 | 1.00e+00 |
|  | X | 13587506 | 13587678 | PDFSTATSENSEMBLUCSC | 173 | 4 | 41190 | tss;island | 0.39407 | 0.11925 | Not queried | 2.47 | 2.00e-01 | 1.00e+00 |
|  | 19 | 38183253 | 38183456 | PDFSTATSENSEMBLUCSC | 204 | 6 | 20707 | tss | 0.25882 | 0.05133 | Not queried | 2.47 | 2.16e-03 | 1.00e+00 |
|  | 1 | 228464049 | 228464394 | PDFSTATSENSEMBLUCSC | 346 | 4 | 1049 | gene;island | 0.52786 | 0.88204 | Not queried | 2.47 | 1.14e-01 | 1.00e+00 |
|  | 11 | 113953462 | 113953838 | PDFSTATSENSEMBLUCSC | 377 | 4 | 6890 | gene;island | 0.73033 | 0.30028 | Not queried | 2.47 | 1.14e-01 | 1.00e+00 |
|  | 10 | 102501755 | 102502104 | PDFSTATSENSEMBLUCSC | 350 | 4 | 6014 | island | 0.30460 | 0.05519 | Not queried | 2.47 | 2.86e-02 | 1.00e+00 |
|  | 10 | 119293083 | 119293550 | PDFSTATSENSEMBLUCSC | 468 | 5 | 4766 | gene;island | 0.04352 | 0.22912 | Not queried | 2.47 | 9.52e-02 | 1.00e+00 |
|  | 1 | 1141617 | 1142405 | PDFSTATSENSEMBLUCSC | 789 | 9 | 2410 | island | 0.46816 | 0.84146 | Not queried | 2.47 | 4.94e-04 | 1.00e+00 |
|  | 16 | 34295778 | 34296306 | PDFSTATSENSEMBLUCSC | 529 | 4 | 15858 | island | 0.23195 | 0.54499 | Not queried | 2.47 | 1.14e-01 | 1.00e+00 |
|  | 6 | 27215328 | 27215397 | PDFSTATSENSEMBLUCSC | 70 | 4 | 34650 | tss | 0.50605 | 0.15647 | Not queried | 2.47 | 1.14e-01 | 1.00e+00 |
|  | 6 | 27215328 | 27215675 | PDFSTATSENSEMBLUCSC | 348 | 6 | 35487 | island | 0.54792 | 0.15647 | Not queried | 2.47 | 2.60e-02 | 1.00e+00 |
|  | 6 | 155537901 | 155538055 | PDFSTATSENSEMBLUCSC | 155 | 6 | 35247 | tss | 0.14551 | 0.44032 | Not queried | 2.47 | 4.33e-03 | 1.00e+00 |
|  | 12 | 58003774 | 58003965 | PDFSTATSENSEMBLUCSC | 192 | 5 | 9796 | tss;island | 0.19104 | 0.69107 | Not queried | 2.46 | 1.59e-02 | 1.00e+00 |
|  | 6 | 30610729 | 30610781 | PDFSTATSENSEMBLUCSC | 53 | 4 | 33727 | gene;island | 0.77510 | 0.92604 | Not queried | 2.46 | 2.86e-02 | 1.00e+00 |
|  | 6 | 29796351 | 29796770 | PDFSTATSENSEMBLUCSC | 420 | 15 | 33634 | gene;island | 0.16317 | 0.48590 | Not queried | 2.46 | 1.96e-03 | 1.00e+00 |
|  | 6 | 27841633 | 27841865 | PDFSTATSENSEMBLUCSC | 233 | 4 | 34665 | tss;island | 0.52060 | 0.87866 | Not queried | 2.46 | 2.86e-02 | 1.00e+00 |
|  | 11 | 62647979 | 62648277 | PDFSTATSENSEMBLUCSC | 299 | 6 | 7352 | tss | 0.02212 | 0.17010 | Not queried | 2.46 | 8.66e-03 | 1.00e+00 |
|  | 5 | 140734543 | 140735027 | PDFSTATSENSEMBLUCSC | 485 | 6 | 31763 | gene;island | 0.31816 | 0.75040 | Not queried | 2.46 | 4.11e-02 | 1.00e+00 |
|  | 5 | 87985809 | 87986067 | PDFSTATSENSEMBLUCSC | 259 | 4 | 32886 | island | 0.07196 | 0.00825 | Not queried | 2.46 | 2.00e-01 | 1.00e+00 |
|  | 11 | 313120 | 313624 | PDFSTATSENSEMBLUCSC | 505 | 6 | 6988 | tss;island | 0.48387 | 0.86425 | Not queried | 2.46 | 2.16e-03 | 1.00e+00 |
|  | 6 | 166722053 | 166722248 | PDFSTATSENSEMBLUCSC | 196 | 6 | 35271 | tss;island | 0.43317 | 0.09456 | Not queried | 2.46 | 4.33e-03 | 1.00e+00 |
|  | 12 | 132293329 | 132293702 | PDFSTATSENSEMBLUCSC | 374 | 5 | 10986 | island | 0.68468 | 0.94191 | Not queried | 2.46 | 3.17e-02 | 1.00e+00 |
|  | X | 37026349 | 37026473 | PDFSTATSENSEMBLUCSC | 125 | 4 | 41711 | island | 0.57138 | 0.89724 | Not queried | 2.46 | 2.86e-02 | 1.00e+00 |
|  | 19 | 50249464 | 50249927 | PDFSTATSENSEMBLUCSC | 464 | 6 | 20126 | gene;island | 0.11223 | 0.55338 | Not queried | 2.45 | 2.60e-02 | 1.00e+00 |
|  | 13 | 113776873 | 113777109 | PDFSTATSENSEMBLUCSC | 237 | 8 | 11520 | tss | 0.39161 | 0.75438 | Not queried | 2.45 | 1.55e-04 | 7.99e-01 |
|  | 16 | 15489614 | 15489968 | PDFSTATSENSEMBLUCSC | 355 | 4 | 14644 | gene | 0.03327 | 0.26478 | Not queried | 2.45 | 3.43e-01 | 1.00e+00 |
|  | 12 | 6756377 | 6756815 | PDFSTATSENSEMBLUCSC | 439 | 6 | 10176 | island | 0.10667 | 0.37829 | Not queried | 2.45 | 4.11e-02 | 1.00e+00 |
|  | 6 | 100911687 | 100911746 | PDFSTATSENSEMBLUCSC | 60 | 6 | 35075 | tss | 0.25778 | 0.06611 | Not queried | 2.45 | 2.16e-03 | 1.00e+00 |
|  | 6 | 100911526 | 100911746 | PDFSTATSENSEMBLUCSC | 221 | 8 | 36303 | island | 0.34942 | 0.09154 | Not queried | 2.45 | 1.86e-03 | 1.00e+00 |
|  | 10 | 73156377 | 73156647 | PDFSTATSENSEMBLUCSC | 271 | 4 | 5126 | tss | 0.34493 | 0.05429 | Not queried | 2.45 | 3.43e-01 | 1.00e+00 |
|  | 2 | 182545485 | 182545934 | PDFSTATSENSEMBLUCSC | 450 | 9 | 23725 | tss;island | 0.07838 | 0.35771 | Not queried | 2.45 | 2.44e-02 | 1.00e+00 |
|  | X | 48768927 | 48769107 | PDFSTATSENSEMBLUCSC | 181 | 7 | 41299 | tss | 0.18229 | 0.59281 | Not queried | 2.45 | 5.83e-04 | 1.00e+00 |
|  | 1 | 153939684 | 153939851 | PDFSTATSENSEMBLUCSC | 168 | 5 | 769 | gene;tss;island | 0.10817 | 0.02049 | Not queried | 2.45 | 7.94e-03 | 1.00e+00 |
|  | 19 | 2717293 | 2717615 | PDFSTATSENSEMBLUCSC | 323 | 4 | 19665 | gene;island | 0.51722 | 0.83531 | Not queried | 2.45 | 1.14e-01 | 1.00e+00 |
|  | 3 | 169482714 | 169483295 | PDFSTATSENSEMBLUCSC | 582 | 9 | 29603 | island | 0.32044 | 0.73358 | Not queried | 2.45 | 2.22e-01 | 1.00e+00 |
|  | 8 | 103613080 | 103613429 | PDFSTATSENSEMBLUCSC | 350 | 4 | 40444 | island | 0.69671 | 0.28697 | Not queried | 2.45 | 2.86e-02 | 1.00e+00 |
|  | 1 | 171810105 | 171810570 | PDFSTATSENSEMBLUCSC | 466 | 8 | 2089 | tss;island | 0.12996 | 0.02102 | Not queried | 2.45 | 3.79e-02 | 1.00e+00 |
|  | 19 | 17958339 | 17958825 | PDFSTATSENSEMBLUCSC | 487 | 6 | 19889 | gene | 0.50857 | 0.12623 | Not queried | 2.45 | 6.49e-02 | 1.00e+00 |
|  | 3 | 50388115 | 50388192 | PDFSTATSENSEMBLUCSC | 78 | 5 | 28313 | tss | 0.18455 | 0.04054 | Not queried | 2.45 | 7.94e-03 | 1.00e+00 |
|  | X | 48397520 | 48398133 | PDFSTATSENSEMBLUCSC | 614 | 13 | 41764 | island | 0.19996 | 0.57772 | Not queried | 2.44 | 6.28e-04 | 1.00e+00 |
|  | 2 | 223290012 | 223290515 | PDFSTATSENSEMBLUCSC | 504 | 4 | 23016 | gene;island | 0.53752 | 0.16653 | Not queried | 2.44 | 2.00e-01 | 1.00e+00 |
|  | 10 | 22725204 | 22725587 | PDFSTATSENSEMBLUCSC | 384 | 5 | 5586 | island | 0.90684 | 0.64207 | Not queried | 2.44 | 7.94e-03 | 1.00e+00 |
|  | 2 | 242212422 | 242212528 | PDFSTATSENSEMBLUCSC | 107 | 5 | 23096 | gene;tss;island | 0.13327 | 0.02844 | Not queried | 2.44 | 5.48e-01 | 1.00e+00 |
|  | 5 | 1882885 | 1883011 | PDFSTATSENSEMBLUCSC | 127 | 4 | 31915 | tss | 0.01361 | 0.13235 | Not queried | 2.44 | 2.00e-01 | 1.00e+00 |
|  | 7 | 120968877 | 120969174 | PDFSTATSENSEMBLUCSC | 298 | 8 | 37140 | gene;island | 0.13917 | 0.43901 | Not queried | 2.44 | 1.55e-04 | 7.99e-01 |
|  | X | 47930589 | 47930653 | PDFSTATSENSEMBLUCSC | 65 | 4 | 41288 | tss;island | 0.15301 | 0.43453 | Not queried | 2.44 | 2.86e-02 | 1.00e+00 |
|  | 1 | 47696212 | 47696788 | PDFSTATSENSEMBLUCSC | 577 | 6 | 1604 | tss;island | 0.20835 | 0.02615 | Not queried | 2.44 | 8.66e-03 | 1.00e+00 |
|  | 6 | 32526263 | 32526414 | PDFSTATSENSEMBLUCSC | 152 | 5 | 34054 | gene | 0.20724 | 0.58290 | Not queried | 2.44 | 4.21e-01 | 1.00e+00 |
|  | 14 | 103739856 | 103740085 | PDFSTATSENSEMBLUCSC | 230 | 4 | 13156 | island | 0.64849 | 0.92099 | Not queried | 2.43 | 2.86e-02 | 1.00e+00 |
|  | 7 | 29519297 | 29519450 | PDFSTATSENSEMBLUCSC | 154 | 5 | 37446 | tss | 0.39535 | 0.05908 | Not queried | 2.43 | 1.51e-01 | 1.00e+00 |
|  | 2 | 175205716 | 175205852 | PDFSTATSENSEMBLUCSC | 137 | 4 | 24837 | island | 0.17727 | 0.04286 | Not queried | 2.43 | 1.14e-01 | 1.00e+00 |
|  | 17 | 68128754 | 68128923 | PDFSTATSENSEMBLUCSC | 170 | 4 | 16708 | gene | 0.95642 | 0.82149 | Not queried | 2.43 | 1.14e-01 | 1.00e+00 |
|  | 4 | 53617076 | 53617448 | PDFSTATSENSEMBLUCSC | 373 | 5 | 30993 | island | 0.34254 | 0.06898 | Not queried | 2.43 | 9.52e-02 | 1.00e+00 |
|  | 12 | 132168981 | 132169130 | PDFSTATSENSEMBLUCSC | 150 | 4 | 10983 | island | 0.30045 | 0.69719 | Not queried | 2.43 | 2.86e-02 | 1.00e+00 |
|  | 17 | 1958851 | 1959132 | PDFSTATSENSEMBLUCSC | 282 | 6 | 16291 | gene;tss;island | 0.12527 | 0.50933 | Not queried | 2.43 | 2.60e-02 | 1.00e+00 |
|  | 15 | 45315362 | 45315438 | PDFSTATSENSEMBLUCSC | 77 | 4 | 13296 | gene | 0.01477 | 0.00834 | Not queried | 2.43 | 6.86e-01 | 1.00e+00 |
|  | 17 | 46802740 | 46803008 | PDFSTATSENSEMBLUCSC | 269 | 4 | 16629 | gene;island | 0.88843 | 0.52705 | Not queried | 2.43 | 2.00e-01 | 1.00e+00 |
|  | 19 | 17392161 | 17392438 | PDFSTATSENSEMBLUCSC | 278 | 7 | 20548 | tss;island | 0.67691 | 0.43216 | Not queried | 2.43 | 7.28e-02 | 1.00e+00 |
|  | 6 | 155584613 | 155584968 | PDFSTATSENSEMBLUCSC | 356 | 4 | 34456 | gene;tss | 0.81950 | 0.96476 | Not queried | 2.43 | 2.86e-02 | 1.00e+00 |
|  | 2 | 27603547 | 27604131 | PDFSTATSENSEMBLUCSC | 585 | 12 | 24143 | island | 0.57713 | 0.19000 | Not queried | 2.43 | 3.64e-03 | 1.00e+00 |
|  | 11 | 65640375 | 65640516 | PDFSTATSENSEMBLUCSC | 142 | 4 | 7426 | tss | 0.02749 | 0.13296 | Not queried | 2.43 | 5.71e-02 | 1.00e+00 |
|  | 6 | 10883834 | 10884314 | PDFSTATSENSEMBLUCSC | 481 | 6 | 35377 | island | 0.80530 | 0.42888 | Not queried | 2.43 | 2.16e-03 | 1.00e+00 |
|  | 3 | 36986513 | 36987040 | PDFSTATSENSEMBLUCSC | 528 | 10 | 28936 | island | 0.38795 | 0.10256 | Not queried | 2.43 | 8.92e-02 | 1.00e+00 |
|  | 10 | 95326033 | 95326409 | PDFSTATSENSEMBLUCSC | 377 | 9 | 5221 | tss;island | 0.20239 | 0.04677 | Not queried | 2.43 | 2.44e-02 | 1.00e+00 |
|  | 2 | 105275613 | 105275920 | PDFSTATSENSEMBLUCSC | 308 | 4 | 24538 | island | 0.03977 | 0.18499 | Not queried | 2.43 | 5.71e-02 | 1.00e+00 |
|  | 6 | 158937125 | 158937485 | PDFSTATSENSEMBLUCSC | 361 | 4 | 36534 | island | 0.46782 | 0.87171 | Not queried | 2.43 | 2.86e-02 | 1.00e+00 |
|  | 10 | 134884177 | 134884645 | PDFSTATSENSEMBLUCSC | 469 | 7 | 6311 | island | 0.55740 | 0.82290 | Not queried | 2.42 | 1.75e-02 | 1.00e+00 |
|  | 1 | 1142132 | 1142405 | PDFSTATSENSEMBLUCSC | 274 | 4 | 1141 | tss | 0.37994 | 0.76588 | Not queried | 2.42 | 2.86e-02 | 1.00e+00 |
|  | 10 | 102590168 | 102590401 | PDFSTATSENSEMBLUCSC | 234 | 4 | 6018 | island | 0.76443 | 0.39923 | Not queried | 2.42 | 2.86e-02 | 1.00e+00 |
|  | X | 34405983 | 34406351 | PDFSTATSENSEMBLUCSC | 369 | 4 | 41708 | island | 0.57552 | 0.85003 | Not queried | 2.42 | 2.86e-02 | 1.00e+00 |
|  | 1 | 229542868 | 229543254 | PDFSTATSENSEMBLUCSC | 387 | 4 | 4295 | island | 0.24480 | 0.05471 | Not queried | 2.42 | 5.71e-02 | 1.00e+00 |
|  | 13 | 21893289 | 21893662 | PDFSTATSENSEMBLUCSC | 374 | 4 | 11559 | island | 0.54714 | 0.88132 | Not queried | 2.42 | 2.86e-02 | 1.00e+00 |
|  | 22 | 46731652 | 46731750 | PDFSTATSENSEMBLUCSC | 99 | 4 | 26871 | gene;island | 0.00471 | 0.01348 | Not queried | 2.42 | 3.43e-01 | 1.00e+00 |
|  | 2 | 27603611 | 27604131 | PDFSTATSENSEMBLUCSC | 521 | 11 | 23229 | tss | 0.58975 | 0.19293 | Not queried | 2.42 | 8.33e-03 | 1.00e+00 |
|  | X | 102531822 | 102532056 | PDFSTATSENSEMBLUCSC | 235 | 5 | 41438 | tss | 0.30587 | 0.12728 | Not queried | 2.42 | 7.94e-03 | 1.00e+00 |
|  | 20 | 31870775 | 31870935 | PDFSTATSENSEMBLUCSC | 161 | 4 | 25642 | tss | 0.32538 | 0.57719 | Not queried | 2.42 | 2.00e-01 | 1.00e+00 |
|  | 1 | 6484616 | 6484980 | PDFSTATSENSEMBLUCSC | 365 | 4 | 2600 | island | 0.03587 | 0.21979 | Not queried | 2.42 | 3.43e-01 | 1.00e+00 |
|  | 1 | 2398837 | 2399054 | PDFSTATSENSEMBLUCSC | 218 | 4 | 2500 | island | 0.12548 | 0.39641 | Not queried | 2.42 | 3.43e-01 | 1.00e+00 |
|  | 15 | 90808551 | 90808880 | PDFSTATSENSEMBLUCSC | 330 | 7 | 13871 | tss | 0.06784 | 0.03032 | Not queried | 2.41 | 6.99e-03 | 1.00e+00 |
|  | 2 | 102090791 | 102091321 | PDFSTATSENSEMBLUCSC | 531 | 11 | 24533 | island | 0.24306 | 0.48398 | Not queried | 2.41 | 1.92e-02 | 1.00e+00 |
|  | 1 | 19970204 | 19970337 | PDFSTATSENSEMBLUCSC | 134 | 4 | 283 | gene;tss;island | 0.11548 | 0.38187 | Not queried | 2.41 | 1.14e-01 | 1.00e+00 |
|  | 1 | 158147446 | 158147849 | PDFSTATSENSEMBLUCSC | 404 | 4 | 3866 | island | 0.01163 | 0.07164 | Not queried | 2.41 | 1.14e-01 | 1.00e+00 |
|  | 15 | 100890907 | 100890996 | PDFSTATSENSEMBLUCSC | 90 | 4 | 13900 | tss;island | 0.16036 | 0.36518 | Not queried | 2.41 | 2.00e-01 | 1.00e+00 |
|  | 7 | 50132541 | 50133212 | PDFSTATSENSEMBLUCSC | 672 | 12 | 38425 | island | 0.46129 | 0.84820 | Not queried | 2.41 | 1.21e-02 | 1.00e+00 |
|  | 5 | 122434853 | 122435202 | PDFSTATSENSEMBLUCSC | 350 | 4 | 31675 | gene;island | 0.03421 | 0.13935 | Not queried | 2.41 | 3.43e-01 | 1.00e+00 |
|  | 3 | 48471575 | 48471901 | PDFSTATSENSEMBLUCSC | 327 | 5 | 28255 | tss;island | 0.67018 | 0.33921 | Not queried | 2.41 | 5.56e-02 | 1.00e+00 |
|  | 6 | 44310228 | 44310350 | PDFSTATSENSEMBLUCSC | 123 | 6 | 34949 | tss | 0.52642 | 0.84326 | Not queried | 2.41 | 2.16e-03 | 1.00e+00 |
|  | 4 | 4866047 | 4866192 | PDFSTATSENSEMBLUCSC | 146 | 4 | 30822 | island | 0.06710 | 0.28529 | Not queried | 2.41 | 2.00e-01 | 1.00e+00 |
|  | 16 | 54228169 | 54228582 | PDFSTATSENSEMBLUCSC | 414 | 5 | 15897 | island | 0.57211 | 0.89491 | Not queried | 2.41 | 1.59e-02 | 1.00e+00 |
|  | 11 | 68780866 | 68780984 | PDFSTATSENSEMBLUCSC | 119 | 4 | 7494 | tss;island | 0.37169 | 0.77021 | Not queried | 2.41 | 2.86e-02 | 1.00e+00 |
|  | 13 | 114786825 | 114786904 | PDFSTATSENSEMBLUCSC | 80 | 4 | 11279 | gene;island | 0.71080 | 0.92879 | Not queried | 2.41 | 2.00e-01 | 1.00e+00 |
|  | 3 | 150690183 | 150690456 | PDFSTATSENSEMBLUCSC | 274 | 6 | 28621 | tss | 0.18247 | 0.56134 | Not queried | 2.41 | 6.49e-02 | 1.00e+00 |
|  | 11 | 63997306 | 63997740 | PDFSTATSENSEMBLUCSC | 435 | 7 | 7366 | tss;island | 0.03466 | 0.03775 | Not queried | 2.40 | 6.20e-01 | 1.00e+00 |
|  | 2 | 2754829 | 2755386 | PDFSTATSENSEMBLUCSC | 558 | 5 | 23986 | island | 0.59600 | 0.90046 | Not queried | 2.40 | 3.17e-02 | 1.00e+00 |
|  | X | 70521196 | 70521650 | PDFSTATSENSEMBLUCSC | 455 | 7 | 41024 | gene | 0.74717 | 0.92732 | Not queried | 2.40 | 5.83e-04 | 1.00e+00 |
|  | X | 135862961 | 135863104 | PDFSTATSENSEMBLUCSC | 144 | 4 | 41122 | gene | 0.13582 | 0.41429 | Not queried | 2.40 | 2.86e-02 | 1.00e+00 |
|  | X | 67718299 | 67718729 | PDFSTATSENSEMBLUCSC | 431 | 8 | 41356 | tss;island | 0.27088 | 0.06721 | Not queried | 2.40 | 3.11e-04 | 1.00e+00 |
|  | 10 | 81679675 | 81679852 | PDFSTATSENSEMBLUCSC | 178 | 4 | 5167 | tss | 0.55736 | 0.89505 | Not queried | 2.40 | 1.14e-01 | 1.00e+00 |
|  | 11 | 66103386 | 66103604 | PDFSTATSENSEMBLUCSC | 219 | 4 | 6713 | gene;island | 0.25442 | 0.64317 | Not queried | 2.40 | 2.86e-02 | 1.00e+00 |
|  | 10 | 97054967 | 97055439 | PDFSTATSENSEMBLUCSC | 473 | 4 | 5945 | island | 0.22170 | 0.04597 | Not queried | 2.40 | 5.71e-02 | 1.00e+00 |
|  | 4 | 53727714 | 53728038 | PDFSTATSENSEMBLUCSC | 325 | 5 | 30346 | tss;island | 0.49603 | 0.33205 | Not queried | 2.40 | 1.00e+00 | 1.00e+00 |
|  | 11 | 69258203 | 69258477 | PDFSTATSENSEMBLUCSC | 275 | 4 | 8565 | island | 0.12114 | 0.39331 | Not queried | 2.40 | 2.86e-02 | 1.00e+00 |
|  | 7 | 100199764 | 100200009 | PDFSTATSENSEMBLUCSC | 246 | 6 | 38706 | island | 0.89798 | 0.63387 | Not queried | 2.40 | 2.16e-03 | 1.00e+00 |
|  | 17 | 6616644 | 6617192 | PDFSTATSENSEMBLUCSC | 549 | 8 | 17876 | island | 0.33212 | 0.08463 | Not queried | 2.40 | 2.81e-02 | 1.00e+00 |
|  | 6 | 28497278 | 28497370 | PDFSTATSENSEMBLUCSC | 93 | 4 | 33571 | gene | 0.92317 | 0.66312 | Not queried | 2.40 | 2.86e-02 | 1.00e+00 |
|  | 3 | 149768455 | 149768762 | PDFSTATSENSEMBLUCSC | 308 | 5 | 29543 | island | 0.81433 | 0.95798 | Not queried | 2.40 | 7.94e-03 | 1.00e+00 |
|  | 1 | 226070448 | 226070808 | PDFSTATSENSEMBLUCSC | 361 | 7 | 2279 | tss | 0.12514 | 0.03047 | Not queried | 2.40 | 2.62e-02 | 1.00e+00 |
|  | 17 | 47209660 | 47209827 | PDFSTATSENSEMBLUCSC | 168 | 4 | 17448 | tss;island | 0.88183 | 0.40609 | Not queried | 2.40 | 2.00e-01 | 1.00e+00 |
|  | 2 | 89065319 | 89065379 | PDFSTATSENSEMBLUCSC | 61 | 4 | 23466 | tss | 0.21685 | 0.53189 | Not queried | 2.40 | 2.00e-01 | 1.00e+00 |
|  | 1 | 26737005 | 26737470 | PDFSTATSENSEMBLUCSC | 466 | 8 | 2889 | island | 0.04360 | 0.20213 | Not queried | 2.40 | 1.09e-03 | 1.00e+00 |
|  | 14 | 57275166 | 57275413 | PDFSTATSENSEMBLUCSC | 248 | 4 | 12064 | gene;island | 0.29413 | 0.04513 | Not queried | 2.40 | 5.71e-02 | 1.00e+00 |
|  | 6 | 168377452 | 168377744 | PDFSTATSENSEMBLUCSC | 293 | 6 | 36587 | island | 0.83671 | 0.91231 | Not queried | 2.40 | 1.32e-01 | 1.00e+00 |
|  | 12 | 50297477 | 50297945 | PDFSTATSENSEMBLUCSC | 469 | 7 | 10380 | island | 0.37188 | 0.63470 | Not queried | 2.39 | 1.17e-03 | 1.00e+00 |
|  | 12 | 372049 | 372259 | PDFSTATSENSEMBLUCSC | 211 | 5 | 9450 | tss | 0.30903 | 0.68371 | Not queried | 2.39 | 5.56e-02 | 1.00e+00 |
|  | 2 | 171570700 | 171571151 | PDFSTATSENSEMBLUCSC | 452 | 6 | 23670 | tss;island | 0.17628 | 0.03387 | Not queried | 2.39 | 4.33e-03 | 1.00e+00 |
|  | 19 | 41129881 | 41130041 | PDFSTATSENSEMBLUCSC | 161 | 4 | 20013 | gene;island | 0.01635 | 0.04554 | Not queried | 2.39 | 4.86e-01 | 1.00e+00 |
|  | 2 | 3383078 | 3383502 | PDFSTATSENSEMBLUCSC | 425 | 6 | 23992 | island | 0.09000 | 0.03817 | Not queried | 2.39 | 3.94e-01 | 1.00e+00 |
|  | 3 | 147125712 | 147127143 | PDFSTATSENSEMBLUCSC | 1432 | 27 | 28609 | tss | 0.02342 | 0.08333 | Not queried | 2.39 | 1.59e-05 | 8.16e-02 |
|  | 8 | 9008364 | 9008681 | PDFSTATSENSEMBLUCSC | 318 | 6 | 39480 | tss | 0.08829 | 0.03577 | Not queried | 2.39 | 4.11e-02 | 1.00e+00 |
|  | 19 | 54466187 | 54466724 | PDFSTATSENSEMBLUCSC | 538 | 6 | 22356 | island | 0.56789 | 0.23312 | Not queried | 2.39 | 8.66e-03 | 1.00e+00 |
|  | 12 | 96350519 | 96350795 | PDFSTATSENSEMBLUCSC | 277 | 5 | 9230 | gene;island | 0.92581 | 0.70489 | Not queried | 2.39 | 7.94e-03 | 1.00e+00 |
|  | 5 | 142065671 | 142065819 | PDFSTATSENSEMBLUCSC | 149 | 5 | 32339 | tss | 0.47531 | 0.81882 | Not queried | 2.38 | 5.56e-02 | 1.00e+00 |
|  | 10 | 35484768 | 35484972 | PDFSTATSENSEMBLUCSC | 205 | 4 | 4545 | gene | 0.04649 | 0.01407 | Not queried | 2.38 | 4.86e-01 | 1.00e+00 |
|  | 5 | 37835506 | 37836392 | PDFSTATSENSEMBLUCSC | 887 | 11 | 31535 | gene;tss;island | 0.90825 | 0.64167 | Not queried | 2.38 | 3.36e-02 | 1.00e+00 |
|  | 7 | 89783767 | 89783976 | PDFSTATSENSEMBLUCSC | 210 | 4 | 37017 | gene | 0.05311 | 0.21225 | Not queried | 2.38 | 2.00e-01 | 1.00e+00 |
|  | 12 | 75603068 | 75603480 | PDFSTATSENSEMBLUCSC | 413 | 5 | 9195 | gene | 0.11642 | 0.29241 | Not queried | 2.38 | 1.51e-01 | 1.00e+00 |
|  | 12 | 75603068 | 75603748 | PDFSTATSENSEMBLUCSC | 681 | 7 | 10605 | island | 0.11642 | 0.29241 | Not queried | 2.38 | 2.09e-01 | 1.00e+00 |
|  | 12 | 101604027 | 101604258 | PDFSTATSENSEMBLUCSC | 232 | 4 | 9918 | tss | 0.02316 | 0.10988 | Not queried | 2.38 | 2.00e-01 | 1.00e+00 |
|  | 6 | 73972704 | 73973128 | PDFSTATSENSEMBLUCSC | 425 | 9 | 36186 | island | 0.18145 | 0.03920 | Not queried | 2.38 | 2.44e-02 | 1.00e+00 |
|  | X | 34960814 | 34960973 | PDFSTATSENSEMBLUCSC | 160 | 6 | 41709 | island | 0.58331 | 0.88537 | Not queried | 2.38 | 2.16e-03 | 1.00e+00 |
|  | 5 | 172660796 | 172661132 | PDFSTATSENSEMBLUCSC | 337 | 4 | 31844 | gene;island | 0.58351 | 0.83325 | Not queried | 2.38 | 1.14e-01 | 1.00e+00 |
|  | 13 | 113705742 | 113706204 | PDFSTATSENSEMBLUCSC | 463 | 4 | 11244 | gene;island | 0.71830 | 0.93099 | Not queried | 2.37 | 5.71e-02 | 1.00e+00 |
|  | 12 | 132689907 | 132690782 | PDFSTATSENSEMBLUCSC | 876 | 11 | 9408 | gene;island | 0.65594 | 0.85621 | Not queried | 2.37 | 1.40e-03 | 1.00e+00 |
|  | 1 | 7023495 | 7023864 | PDFSTATSENSEMBLUCSC | 370 | 4 | 183 | gene;island | 0.20284 | 0.03109 | Not queried | 2.37 | 2.00e-01 | 1.00e+00 |
|  | 5 | 141061863 | 141061943 | PDFSTATSENSEMBLUCSC | 81 | 6 | 32331 | tss | 0.03732 | 0.18017 | Not queried | 2.37 | 9.31e-02 | 1.00e+00 |
|  | 16 | 76269043 | 76269409 | PDFSTATSENSEMBLUCSC | 367 | 5 | 16072 | island | 0.51351 | 0.82452 | Not queried | 2.37 | 7.94e-03 | 1.00e+00 |
|  | 1 | 3459949 | 3460467 | PDFSTATSENSEMBLUCSC | 519 | 4 | 128 | gene;island | 0.31266 | 0.77636 | Not queried | 2.37 | 2.00e-01 | 1.00e+00 |
|  | 3 | 180319597 | 180319907 | PDFSTATSENSEMBLUCSC | 311 | 6 | 28692 | tss | 0.14953 | 0.04812 | Not queried | 2.37 | 1.80e-01 | 1.00e+00 |
|  | 11 | 65640137 | 65640516 | PDFSTATSENSEMBLUCSC | 380 | 6 | 8463 | island | 0.04869 | 0.20582 | Not queried | 2.37 | 2.60e-02 | 1.00e+00 |
|  | 11 | 31823141 | 31823282 | PDFSTATSENSEMBLUCSC | 142 | 4 | 6540 | gene;island | 0.23571 | 0.58864 | Not queried | 2.37 | 2.86e-02 | 1.00e+00 |
|  | 10 | 95326801 | 95327325 | PDFSTATSENSEMBLUCSC | 525 | 6 | 4668 | gene;island | 0.16749 | 0.41797 | Not queried | 2.37 | 9.31e-02 | 1.00e+00 |
|  | 13 | 112720171 | 112720565 | PDFSTATSENSEMBLUCSC | 395 | 6 | 11879 | island | 0.40287 | 0.09210 | Not queried | 2.37 | 6.49e-02 | 1.00e+00 |
|  | 5 | 166405485 | 166405869 | PDFSTATSENSEMBLUCSC | 385 | 4 | 33304 | island | 0.28512 | 0.04045 | Not queried | 2.37 | 2.00e-01 | 1.00e+00 |
|  | 18 | 71959244 | 71959795 | PDFSTATSENSEMBLUCSC | 552 | 8 | 19287 | tss | 0.11780 | 0.01793 | Not queried | 2.37 | 3.11e-04 | 1.00e+00 |
|  | 19 | 55791810 | 55792075 | PDFSTATSENSEMBLUCSC | 266 | 5 | 21038 | tss | 0.24925 | 0.06530 | Not queried | 2.36 | 5.48e-01 | 1.00e+00 |
|  | 5 | 140566303 | 140566767 | PDFSTATSENSEMBLUCSC | 465 | 5 | 32306 | tss;island | 0.06881 | 0.65320 | Not queried | 2.36 | 3.10e-01 | 1.00e+00 |
|  | 9 | 139948856 | 139949108 | PDFSTATSENSEMBLUCSC | 253 | 4 | 40792 | tss;island | 0.33063 | 0.69594 | Not queried | 2.36 | 3.43e-01 | 1.00e+00 |
|  | 5 | 140501321 | 140501812 | PDFSTATSENSEMBLUCSC | 492 | 10 | 33149 | island | 0.06960 | 0.58274 | Not queried | 2.36 | 2.88e-02 | 1.00e+00 |
|  | 2 | 214012 | 214353 | PDFSTATSENSEMBLUCSC | 342 | 4 | 23959 | island | 0.68005 | 0.88921 | Not queried | 2.36 | 2.86e-02 | 1.00e+00 |
|  | 19 | 47292038 | 47292253 | PDFSTATSENSEMBLUCSC | 216 | 4 | 20863 | tss;island | 0.32978 | 0.03104 | Not queried | 2.36 | 2.00e-01 | 1.00e+00 |
|  | 12 | 124941307 | 124941423 | PDFSTATSENSEMBLUCSC | 117 | 4 | 9370 | gene;island | 0.61861 | 0.88385 | Not queried | 2.36 | 2.86e-02 | 1.00e+00 |
|  | 6 | 75911716 | 75912132 | PDFSTATSENSEMBLUCSC | 417 | 4 | 34328 | gene;island | 0.45670 | 0.11416 | Not queried | 2.36 | 2.86e-02 | 1.00e+00 |
|  | 4 | 1005876 | 1006240 | PDFSTATSENSEMBLUCSC | 365 | 8 | 30189 | tss | 0.07508 | 0.04785 | Not queried | 2.36 | 5.74e-01 | 1.00e+00 |
|  | 3 | 147109426 | 147109784 | PDFSTATSENSEMBLUCSC | 359 | 4 | 27943 | gene;island | 0.17112 | 0.47719 | Not queried | 2.36 | 3.43e-01 | 1.00e+00 |
|  | 3 | 44596178 | 44596770 | PDFSTATSENSEMBLUCSC | 593 | 9 | 28992 | island | 0.86903 | 0.62754 | Not queried | 2.36 | 2.58e-01 | 1.00e+00 |
|  | 1 | 230468464 | 230468773 | PDFSTATSENSEMBLUCSC | 310 | 5 | 1068 | gene;island | 0.06871 | 0.36522 | Not queried | 2.36 | 3.17e-02 | 1.00e+00 |
|  | 2 | 223163573 | 223163809 | PDFSTATSENSEMBLUCSC | 237 | 5 | 23011 | gene;island | 0.17816 | 0.02686 | Not queried | 2.36 | 3.17e-02 | 1.00e+00 |
|  | 8 | 30892276 | 30892490 | PDFSTATSENSEMBLUCSC | 215 | 4 | 39210 | gene;tss | 0.45075 | 0.11580 | Not queried | 2.36 | 1.14e-01 | 1.00e+00 |
|  | 18 | 56296243 | 56296607 | PDFSTATSENSEMBLUCSC | 365 | 6 | 19260 | tss | 0.55902 | 0.87804 | Not queried | 2.36 | 2.60e-02 | 1.00e+00 |
|  | 1 | 32169568 | 32169868 | PDFSTATSENSEMBLUCSC | 301 | 4 | 2967 | island | 0.86191 | 0.56744 | Not queried | 2.36 | 2.86e-02 | 1.00e+00 |
|  | 12 | 112204716 | 112204829 | PDFSTATSENSEMBLUCSC | 114 | 4 | 9286 | gene;island | 0.15941 | 0.02693 | Not queried | 2.36 | 1.14e-01 | 1.00e+00 |
|  | 12 | 58011764 | 58011875 | PDFSTATSENSEMBLUCSC | 112 | 4 | 10540 | island | 0.33188 | 0.72105 | Not queried | 2.36 | 3.43e-01 | 1.00e+00 |
|  | 11 | 1404502 | 1404750 | PDFSTATSENSEMBLUCSC | 249 | 4 | 7899 | island | 0.05509 | 0.20415 | Not queried | 2.36 | 2.86e-02 | 1.00e+00 |
|  | 11 | 65425928 | 65426084 | PDFSTATSENSEMBLUCSC | 157 | 5 | 6698 | gene | 0.58713 | 0.87916 | Not queried | 2.35 | 7.94e-03 | 1.00e+00 |
|  | 5 | 16508920 | 16509123 | PDFSTATSENSEMBLUCSC | 204 | 4 | 31511 | gene | 0.13520 | 0.44216 | Not queried | 2.35 | 2.86e-02 | 1.00e+00 |
|  | 5 | 178487716 | 178488100 | PDFSTATSENSEMBLUCSC | 385 | 4 | 31869 | gene | 0.24941 | 0.50058 | Not queried | 2.35 | 4.86e-01 | 1.00e+00 |
|  | 17 | 46824727 | 46825371 | PDFSTATSENSEMBLUCSC | 645 | 5 | 18472 | island | 0.23760 | 0.61202 | Not queried | 2.35 | 1.59e-02 | 1.00e+00 |
|  | 5 | 142065539 | 142065819 | PDFSTATSENSEMBLUCSC | 281 | 6 | 31786 | gene | 0.48415 | 0.79747 | Not queried | 2.35 | 2.60e-02 | 1.00e+00 |
|  | 12 | 101603835 | 101603996 | PDFSTATSENSEMBLUCSC | 162 | 4 | 9242 | gene | 0.01696 | 0.06737 | Not queried | 2.35 | 1.14e-01 | 1.00e+00 |
|  | 12 | 101603835 | 101604258 | PDFSTATSENSEMBLUCSC | 424 | 8 | 10690 | island | 0.01914 | 0.06737 | Not queried | 2.35 | 2.07e-02 | 1.00e+00 |
|  | 1 | 936091 | 936610 | PDFSTATSENSEMBLUCSC | 520 | 5 | 1134 | tss;island | 0.03622 | 0.40415 | Not queried | 2.35 | 3.17e-02 | 1.00e+00 |
|  | 22 | 18531028 | 18531591 | PDFSTATSENSEMBLUCSC | 564 | 6 | 27240 | island | 0.49552 | 0.83869 | Not queried | 2.35 | 8.66e-03 | 1.00e+00 |
|  | 4 | 46126245 | 46126448 | PDFSTATSENSEMBLUCSC | 204 | 6 | 30324 | tss | 0.05423 | 0.32280 | Not queried | 2.35 | 6.49e-02 | 1.00e+00 |
|  | X | 74743375 | 74743636 | PDFSTATSENSEMBLUCSC | 262 | 5 | 41396 | tss;island | 0.10205 | 0.02418 | Not queried | 2.35 | 7.94e-03 | 1.00e+00 |
|  | 5 | 68710808 | 68710912 | PDFSTATSENSEMBLUCSC | 105 | 6 | 32051 | tss;island | 0.03019 | 0.13718 | Not queried | 2.35 | 8.66e-03 | 1.00e+00 |
|  | 19 | 58545001 | 58545333 | PDFSTATSENSEMBLUCSC | 333 | 8 | 21094 | tss;island | 0.03064 | 0.11710 | Not queried | 2.35 | 6.99e-03 | 1.00e+00 |
|  | 8 | 1496711 | 1496759 | PDFSTATSENSEMBLUCSC | 49 | 4 | 39104 | gene;island | 0.68456 | 0.90856 | Not queried | 2.35 | 2.86e-02 | 1.00e+00 |
|  | 10 | 102896376 | 102896640 | PDFSTATSENSEMBLUCSC | 265 | 4 | 4711 | gene;island | 0.93520 | 0.64312 | Not queried | 2.35 | 2.86e-02 | 1.00e+00 |
|  | 7 | 157982161 | 157982589 | PDFSTATSENSEMBLUCSC | 429 | 5 | 37297 | gene;island | 0.41888 | 0.79197 | Not queried | 2.35 | 2.22e-01 | 1.00e+00 |
|  | 10 | 35894012 | 35894430 | PDFSTATSENSEMBLUCSC | 419 | 8 | 5647 | island | 0.44195 | 0.86746 | Not queried | 2.35 | 1.86e-03 | 1.00e+00 |
|  | 16 | 74808678 | 74808761 | PDFSTATSENSEMBLUCSC | 84 | 5 | 16058 | island | 0.01216 | 0.01658 | Not queried | 2.35 | 2.22e-01 | 1.00e+00 |
|  | 12 | 128850432 | 128850696 | PDFSTATSENSEMBLUCSC | 265 | 5 | 9380 | gene;island | 0.25633 | 0.59923 | Not queried | 2.35 | 7.94e-03 | 1.00e+00 |
|  | 6 | 30690213 | 30690844 | PDFSTATSENSEMBLUCSC | 632 | 14 | 33753 | gene;island | 0.09183 | 0.41452 | Not queried | 2.35 | 2.49e-03 | 1.00e+00 |
|  | X | 73834295 | 73834821 | PDFSTATSENSEMBLUCSC | 527 | 9 | 41878 | island | 0.20476 | 0.62604 | Not queried | 2.34 | 4.11e-05 | 2.11e-01 |
|  | 12 | 53693054 | 53693453 | PDFSTATSENSEMBLUCSC | 400 | 8 | 9713 | tss | 0.05014 | 0.25535 | Not queried | 2.34 | 1.95e-01 | 1.00e+00 |
|  | 12 | 50349173 | 50349522 | PDFSTATSENSEMBLUCSC | 350 | 4 | 9101 | gene;island | 0.86809 | 0.61145 | Not queried | 2.34 | 2.00e-01 | 1.00e+00 |
|  | 10 | 27703329 | 27703547 | PDFSTATSENSEMBLUCSC | 219 | 6 | 4985 | tss | 0.37285 | 0.84329 | Not queried | 2.34 | 1.52e-02 | 1.00e+00 |
|  | 10 | 27703247 | 27703547 | PDFSTATSENSEMBLUCSC | 301 | 8 | 5612 | island | 0.32199 | 0.73106 | Not queried | 2.34 | 6.99e-03 | 1.00e+00 |
|  | 2 | 155554688 | 155555414 | PDFSTATSENSEMBLUCSC | 727 | 9 | 24759 | island | 0.07269 | 0.50187 | Not queried | 2.34 | 7.70e-02 | 1.00e+00 |
|  | 10 | 27547536 | 27547965 | PDFSTATSENSEMBLUCSC | 430 | 6 | 5610 | island | 0.15250 | 0.03707 | Not queried | 2.34 | 1.80e-01 | 1.00e+00 |
|  | 21 | 15352790 | 15352983 | PDFSTATSENSEMBLUCSC | 194 | 5 | 26445 | tss | 0.19327 | 0.47338 | Not queried | 2.34 | 5.56e-02 | 1.00e+00 |
|  | 21 | 15352608 | 15352983 | PDFSTATSENSEMBLUCSC | 376 | 7 | 26565 | island | 0.16295 | 0.39675 | Not queried | 2.34 | 1.11e-02 | 1.00e+00 |
|  | 7 | 73256360 | 73256820 | PDFSTATSENSEMBLUCSC | 461 | 6 | 36980 | gene | 0.36246 | 0.07547 | Not queried | 2.34 | 2.40e-01 | 1.00e+00 |
|  | 7 | 29519297 | 29519656 | PDFSTATSENSEMBLUCSC | 360 | 8 | 36886 | gene | 0.34363 | 0.05670 | Not queried | 2.34 | 2.07e-02 | 1.00e+00 |
|  | 11 | 44331905 | 44333192 | PDFSTATSENSEMBLUCSC | 1288 | 39 | 7240 | tss;island | 0.45237 | 0.12446 | ALX4 (margin: ALX4) | 2.34 | 1.44e-07 | 7.40e-04 |
|  | 10 | 101290958 | 101291397 | PDFSTATSENSEMBLUCSC | 440 | 4 | 5992 | island | 0.72418 | 0.40395 | Not queried | 2.34 | 2.86e-02 | 1.00e+00 |
|  | 15 | 90030016 | 90030410 | PDFSTATSENSEMBLUCSC | 395 | 5 | 13440 | gene;island | 0.24803 | 0.62503 | Not queried | 2.34 | 3.10e-01 | 1.00e+00 |
|  | 11 | 1891699 | 1892142 | PDFSTATSENSEMBLUCSC | 444 | 10 | 7030 | tss | 0.49217 | 0.77104 | Not queried | 2.34 | 2.32e-02 | 1.00e+00 |
|  | 16 | 66586309 | 66586745 | PDFSTATSENSEMBLUCSC | 437 | 9 | 15955 | island | 0.13606 | 0.03255 | Not queried | 2.34 | 4.11e-05 | 2.11e-01 |
|  | 6 | 27228101 | 27228330 | PDFSTATSENSEMBLUCSC | 230 | 4 | 35488 | island | 0.38608 | 0.12147 | Not queried | 2.33 | 1.14e-01 | 1.00e+00 |
|  | 14 | 99880393 | 99880641 | PDFSTATSENSEMBLUCSC | 249 | 4 | 12175 | gene | 0.94431 | 0.77023 | Not queried | 2.33 | 1.14e-01 | 1.00e+00 |
|  | 8 | 97169621 | 97170097 | PDFSTATSENSEMBLUCSC | 477 | 5 | 39303 | gene;island | 0.22381 | 0.61335 | Not queried | 2.33 | 1.59e-02 | 1.00e+00 |
|  | 20 | 22565881 | 22566289 | PDFSTATSENSEMBLUCSC | 409 | 6 | 25594 | tss;island | 0.07792 | 0.20382 | Not queried | 2.33 | 1.52e-02 | 1.00e+00 |
|  | 7 | 114055074 | 114055210 | PDFSTATSENSEMBLUCSC | 137 | 6 | 37129 | gene | 0.01058 | 0.06058 | Not queried | 2.33 | 4.11e-02 | 1.00e+00 |
|  | 17 | 61514690 | 61515010 | PDFSTATSENSEMBLUCSC | 321 | 4 | 16690 | gene;island | 0.65023 | 0.90514 | Not queried | 2.33 | 2.00e-01 | 1.00e+00 |
|  | 6 | 34499314 | 34499504 | PDFSTATSENSEMBLUCSC | 191 | 4 | 34239 | gene;island | 0.71366 | 0.93510 | Not queried | 2.33 | 2.00e-01 | 1.00e+00 |
|  | Y | 15016579 | 15016705 | PDFSTATSENSEMBLUCSC | 127 | 5 | 42160 | gene;island | 0.11017 | 0.48930 | Not queried | 2.33 | 7.94e-03 | 1.00e+00 |
|  | X | 38115344 | 38115655 | PDFSTATSENSEMBLUCSC | 312 | 4 | 41719 | island | 0.40375 | 0.84128 | Not queried | 2.33 | 5.71e-02 | 1.00e+00 |
|  | 10 | 75407372 | 75407991 | PDFSTATSENSEMBLUCSC | 620 | 6 | 4622 | gene;island | 0.26253 | 0.67922 | Not queried | 2.33 | 2.60e-02 | 1.00e+00 |
|  | 2 | 66659348 | 66659590 | PDFSTATSENSEMBLUCSC | 243 | 4 | 24317 | island | 0.17736 | 0.04171 | Not queried | 2.33 | 5.71e-02 | 1.00e+00 |
|  | 16 | 12996046 | 12996587 | PDFSTATSENSEMBLUCSC | 542 | 6 | 14642 | gene;island | 0.01672 | 0.05437 | Not queried | 2.33 | 3.10e-01 | 1.00e+00 |
|  | 1 | 205649791 | 205649956 | PDFSTATSENSEMBLUCSC | 166 | 8 | 2203 | tss;island | 0.18049 | 0.03079 | Not queried | 2.33 | 3.11e-04 | 1.00e+00 |
|  | 5 | 140501321 | 140501540 | PDFSTATSENSEMBLUCSC | 220 | 7 | 32303 | tss | 0.08670 | 0.46850 | Not queried | 2.33 | 2.09e-01 | 1.00e+00 |
|  | 12 | 49487944 | 49488311 | PDFSTATSENSEMBLUCSC | 368 | 4 | 9091 | gene;island | 0.07855 | 0.21955 | Not queried | 2.32 | 2.86e-02 | 1.00e+00 |
|  | 20 | 35169260 | 35169609 | PDFSTATSENSEMBLUCSC | 350 | 5 | 25677 | tss;island | 0.43317 | 0.87463 | Not queried | 2.32 | 3.17e-02 | 1.00e+00 |
|  | 7 | 157561593 | 157561925 | PDFSTATSENSEMBLUCSC | 333 | 5 | 37279 | gene;island | 0.83898 | 0.95684 | Not queried | 2.32 | 1.59e-02 | 1.00e+00 |
|  | 16 | 3096477 | 3096662 | PDFSTATSENSEMBLUCSC | 186 | 5 | 15018 | tss | 0.23891 | 0.02886 | Not queried | 2.32 | 1.51e-01 | 1.00e+00 |
|  | X | 72782624 | 72783164 | PDFSTATSENSEMBLUCSC | 541 | 9 | 41388 | tss;island | 0.09375 | 0.34120 | Not queried | 2.32 | 8.23e-05 | 4.23e-01 |
|  | X | 72782624 | 72782852 | PDFSTATSENSEMBLUCSC | 229 | 5 | 41035 | gene | 0.09375 | 0.34120 | Not queried | 2.32 | 7.94e-03 | 1.00e+00 |
|  | 3 | 42543933 | 42544101 | PDFSTATSENSEMBLUCSC | 169 | 6 | 28201 | tss | 0.13055 | 0.02392 | Not queried | 2.32 | 8.66e-03 | 1.00e+00 |
|  | 6 | 31733619 | 31734580 | PDFSTATSENSEMBLUCSC | 962 | 20 | 33888 | gene | 0.22033 | 0.53609 | Not queried | 2.32 | 5.62e-03 | 1.00e+00 |
|  | 13 | 28498091 | 28498544 | PDFSTATSENSEMBLUCSC | 454 | 6 | 11089 | gene;island | 0.65591 | 0.25979 | Not queried | 2.32 | 8.66e-03 | 1.00e+00 |
|  | 11 | 101000436 | 101000717 | PDFSTATSENSEMBLUCSC | 282 | 6 | 8731 | island | 0.02508 | 0.16148 | Not queried | 2.32 | 1.32e-01 | 1.00e+00 |
|  | 17 | 58218643 | 58218965 | PDFSTATSENSEMBLUCSC | 323 | 4 | 18585 | island | 0.06222 | 0.41753 | Not queried | 2.32 | 5.71e-02 | 1.00e+00 |
|  | 15 | 90808551 | 90808917 | PDFSTATSENSEMBLUCSC | 367 | 8 | 14423 | island | 0.06379 | 0.03283 | Not queried | 2.32 | 4.66e-03 | 1.00e+00 |
|  | 2 | 177028606 | 177028714 | PDFSTATSENSEMBLUCSC | 109 | 4 | 23704 | tss | 0.84796 | 0.48438 | Not queried | 2.32 | 2.86e-02 | 1.00e+00 |
|  | 3 | 45635931 | 45636225 | PDFSTATSENSEMBLUCSC | 295 | 6 | 28226 | tss | 0.08225 | 0.28812 | Not queried | 2.32 | 4.11e-02 | 1.00e+00 |
|  | 16 | 59788951 | 59789219 | PDFSTATSENSEMBLUCSC | 269 | 4 | 15951 | island | 0.64085 | 0.89846 | Not queried | 2.32 | 2.86e-02 | 1.00e+00 |
|  | 5 | 140568075 | 140568535 | PDFSTATSENSEMBLUCSC | 461 | 4 | 31757 | gene;island | 0.37680 | 0.70948 | Not queried | 2.32 | 2.86e-02 | 1.00e+00 |
|  | 5 | 1876168 | 1876397 | PDFSTATSENSEMBLUCSC | 230 | 5 | 32583 | island | 0.10880 | 0.34070 | Not queried | 2.32 | 9.52e-02 | 1.00e+00 |
|  | 8 | 2003810 | 2004050 | PDFSTATSENSEMBLUCSC | 241 | 5 | 39124 | gene;island | 0.58123 | 0.84292 | Not queried | 2.32 | 7.94e-03 | 1.00e+00 |
|  | 11 | 65779192 | 65779847 | PDFSTATSENSEMBLUCSC | 656 | 10 | 8473 | island | 0.35053 | 0.73060 | Not queried | 2.32 | 2.06e-04 | 1.00e+00 |
|  | X | 55743906 | 55744084 | PDFSTATSENSEMBLUCSC | 179 | 6 | 41343 | tss | 0.10504 | 0.37811 | Not queried | 2.32 | 4.33e-03 | 1.00e+00 |
|  | 11 | 94771024 | 94771514 | PDFSTATSENSEMBLUCSC | 491 | 4 | 8721 | island | 0.64871 | 0.87882 | Not queried | 2.31 | 1.14e-01 | 1.00e+00 |
|  | 22 | 21321253 | 21321434 | PDFSTATSENSEMBLUCSC | 182 | 4 | 26944 | tss | 0.52360 | 0.14659 | Not queried | 2.31 | 2.00e-01 | 1.00e+00 |
|  | 3 | 147125712 | 147127193 | PDFSTATSENSEMBLUCSC | 1482 | 28 | 29528 | island | 0.02220 | 0.08050 | Not queried | 2.31 | 2.10e-05 | 1.08e-01 |
|  | 3 | 193987426 | 193987757 | PDFSTATSENSEMBLUCSC | 332 | 4 | 29741 | island | 0.57208 | 0.92530 | Not queried | 2.31 | 5.71e-02 | 1.00e+00 |
|  | 7 | 95402189 | 95402528 | PDFSTATSENSEMBLUCSC | 340 | 4 | 37036 | gene;island | 0.20245 | 0.04235 | Not queried | 2.31 | 2.00e-01 | 1.00e+00 |
|  | 19 | 17322527 | 17322956 | PDFSTATSENSEMBLUCSC | 430 | 4 | 19878 | gene;island | 0.84449 | 0.95427 | Not queried | 2.31 | 1.14e-01 | 1.00e+00 |
|  | 6 | 33170692 | 33170838 | PDFSTATSENSEMBLUCSC | 147 | 6 | 34159 | gene;island | 0.83350 | 0.91065 | Not queried | 2.31 | 9.31e-02 | 1.00e+00 |
|  | 1 | 167090972 | 167091161 | PDFSTATSENSEMBLUCSC | 190 | 4 | 871 | gene;island | 0.12267 | 0.02392 | Not queried | 2.31 | 2.86e-02 | 1.00e+00 |
|  | X | 48957963 | 48958509 | PDFSTATSENSEMBLUCSC | 547 | 12 | 41783 | island | 0.12243 | 0.50297 | Not queried | 2.31 | 2.01e-04 | 1.00e+00 |
|  | X | 64754624 | 64754995 | PDFSTATSENSEMBLUCSC | 372 | 12 | 41830 | island | 0.10025 | 0.02767 | Not queried | 2.31 | 2.01e-04 | 1.00e+00 |
|  | 1 | 212688762 | 212688997 | PDFSTATSENSEMBLUCSC | 236 | 4 | 4171 | island | 0.06362 | 0.23001 | Not queried | 2.31 | 2.86e-02 | 1.00e+00 |
|  | 6 | 32710933 | 32711102 | PDFSTATSENSEMBLUCSC | 170 | 4 | 34062 | gene | 0.77061 | 0.47888 | Not queried | 2.31 | 4.86e-01 | 1.00e+00 |
|  | X | 47863193 | 47863707 | PDFSTATSENSEMBLUCSC | 515 | 10 | 41287 | tss;island | 0.10260 | 0.02159 | Not queried | 2.31 | 1.08e-05 | 5.56e-02 |
|  | 22 | 42347906 | 42348061 | PDFSTATSENSEMBLUCSC | 156 | 4 | 27142 | tss | 0.82505 | 0.47989 | Not queried | 2.31 | 5.71e-02 | 1.00e+00 |
|  | 10 | 45495677 | 45496237 | PDFSTATSENSEMBLUCSC | 561 | 9 | 5040 | tss | 0.58226 | 0.07626 | Not queried | 2.31 | 4.00e-02 | 1.00e+00 |
|  | 3 | 39851641 | 39851931 | PDFSTATSENSEMBLUCSC | 291 | 4 | 27684 | gene;island | 0.19488 | 0.04159 | Not queried | 2.31 | 3.43e-01 | 1.00e+00 |
|  | 17 | 1961109 | 1961510 | PDFSTATSENSEMBLUCSC | 402 | 4 | 16292 | gene;island | 0.68805 | 0.91518 | Not queried | 2.31 | 2.86e-02 | 1.00e+00 |
|  | X | 48958082 | 48958509 | PDFSTATSENSEMBLUCSC | 428 | 10 | 41308 | tss | 0.12243 | 0.53604 | Not queried | 2.31 | 7.25e-04 | 1.00e+00 |
|  | 5 | 218073 | 218200 | PDFSTATSENSEMBLUCSC | 128 | 4 | 31444 | gene;tss;island | 0.20286 | 0.03626 | Not queried | 2.31 | 4.86e-01 | 1.00e+00 |
|  | 8 | 135490694 | 135491240 | PDFSTATSENSEMBLUCSC | 547 | 7 | 39370 | gene;island | 0.35979 | 0.77173 | Not queried | 2.31 | 7.28e-02 | 1.00e+00 |
|  | 6 | 31730260 | 31730606 | PDFSTATSENSEMBLUCSC | 347 | 4 | 34773 | tss | 0.63422 | 0.89095 | Not queried | 2.30 | 1.14e-01 | 1.00e+00 |
|  | 19 | 58095424 | 58095659 | PDFSTATSENSEMBLUCSC | 236 | 8 | 22438 | island | 0.02483 | 0.09818 | Not queried | 2.30 | 1.04e-02 | 1.00e+00 |
|  | 14 | 95826570 | 95826997 | PDFSTATSENSEMBLUCSC | 428 | 5 | 13081 | island | 0.59440 | 0.87900 | Not queried | 2.30 | 7.94e-03 | 1.00e+00 |
|  | 4 | 4405844 | 4406200 | PDFSTATSENSEMBLUCSC | 357 | 6 | 29871 | gene | 0.60550 | 0.85608 | Not queried | 2.30 | 2.40e-01 | 1.00e+00 |
|  | X | 48687169 | 48687283 | PDFSTATSENSEMBLUCSC | 115 | 4 | 41770 | island | 0.72248 | 0.95467 | Not queried | 2.30 | 2.00e-01 | 1.00e+00 |
|  | 5 | 43040885 | 43041152 | PDFSTATSENSEMBLUCSC | 268 | 4 | 31984 | tss;island | 0.08885 | 0.28041 | Not queried | 2.30 | 5.71e-02 | 1.00e+00 |
|  | 1 | 43533796 | 43534324 | PDFSTATSENSEMBLUCSC | 529 | 6 | 3151 | island | 0.65155 | 0.88311 | Not queried | 2.30 | 4.33e-03 | 1.00e+00 |
|  | 15 | 49338748 | 49339070 | PDFSTATSENSEMBLUCSC | 323 | 5 | 13633 | tss | 0.05111 | 0.20656 | Not queried | 2.30 | 4.21e-01 | 1.00e+00 |
|  | 7 | 1588284 | 1588579 | PDFSTATSENSEMBLUCSC | 296 | 4 | 36698 | gene;island | 0.44130 | 0.80898 | Not queried | 2.30 | 2.86e-02 | 1.00e+00 |
|  | 4 | 71587640 | 71587904 | PDFSTATSENSEMBLUCSC | 265 | 4 | 29972 | gene | 0.76734 | 0.51800 | Not queried | 2.30 | 4.86e-01 | 1.00e+00 |
|  | 2 | 7171869 | 7172097 | PDFSTATSENSEMBLUCSC | 229 | 4 | 22522 | gene;island | 0.49116 | 0.83898 | Not queried | 2.30 | 2.00e-01 | 1.00e+00 |
|  | 8 | 11203882 | 11204132 | PDFSTATSENSEMBLUCSC | 251 | 4 | 39141 | gene;island | 0.64864 | 0.27446 | Not queried | 2.30 | 2.86e-02 | 1.00e+00 |
|  | 10 | 105239085 | 105239205 | PDFSTATSENSEMBLUCSC | 121 | 4 | 5322 | tss | 0.37911 | 0.70572 | Not queried | 2.30 | 2.86e-02 | 1.00e+00 |
|  | 19 | 23299649 | 23300243 | PDFSTATSENSEMBLUCSC | 595 | 5 | 21809 | island | 0.27471 | 0.06751 | Not queried | 2.29 | 9.52e-02 | 1.00e+00 |
|  | 10 | 1415977 | 1416224 | PDFSTATSENSEMBLUCSC | 248 | 4 | 4448 | gene;island | 0.27310 | 0.67515 | Not queried | 2.29 | 2.86e-02 | 1.00e+00 |
|  | 16 | 2770211 | 2770429 | PDFSTATSENSEMBLUCSC | 219 | 4 | 14582 | gene;island | 0.67877 | 0.87503 | Not queried | 2.29 | 2.00e-01 | 1.00e+00 |
|  | 15 | 45406534 | 45406939 | PDFSTATSENSEMBLUCSC | 406 | 6 | 13297 | gene | 0.22967 | 0.56426 | Not queried | 2.29 | 2.16e-03 | 1.00e+00 |
|  | 6 | 40567317 | 40567579 | PDFSTATSENSEMBLUCSC | 263 | 4 | 36052 | island | 0.03967 | 0.32589 | Not queried | 2.29 | 3.43e-01 | 1.00e+00 |
|  | 1 | 173639044 | 173639135 | PDFSTATSENSEMBLUCSC | 92 | 4 | 2093 | tss;island | 0.29149 | 0.07286 | Not queried | 2.29 | 1.14e-01 | 1.00e+00 |
|  | 13 | 103053409 | 103053761 | PDFSTATSENSEMBLUCSC | 353 | 4 | 11188 | gene;island | 0.15695 | 0.35459 | Not queried | 2.29 | 1.14e-01 | 1.00e+00 |
|  | 6 | 31590513 | 31590674 | PDFSTATSENSEMBLUCSC | 162 | 9 | 34758 | tss | 0.49484 | 0.84920 | Not queried | 2.29 | 4.00e-02 | 1.00e+00 |
|  | 17 | 46804123 | 46804414 | PDFSTATSENSEMBLUCSC | 292 | 4 | 16630 | gene;island | 0.84207 | 0.55671 | Not queried | 2.29 | 1.14e-01 | 1.00e+00 |
|  | 17 | 12568499 | 12568761 | PDFSTATSENSEMBLUCSC | 263 | 4 | 17107 | tss;island | 0.01294 | 0.08486 | Not queried | 2.29 | 5.71e-02 | 1.00e+00 |
|  | 9 | 36258825 | 36258971 | PDFSTATSENSEMBLUCSC | 147 | 4 | 40700 | gene;tss;island | 0.84993 | 0.53768 | Not queried | 2.29 | 2.86e-02 | 1.00e+00 |
|  | 19 | 34263905 | 34264111 | PDFSTATSENSEMBLUCSC | 207 | 4 | 19940 | gene;island | 0.61046 | 0.83829 | Not queried | 2.29 | 2.86e-02 | 1.00e+00 |
|  | 6 | 33169581 | 33169731 | PDFSTATSENSEMBLUCSC | 151 | 4 | 34157 | gene;tss;island | 0.48513 | 0.78851 | Not queried | 2.29 | 1.14e-01 | 1.00e+00 |
|  | 4 | 174452835 | 174453287 | PDFSTATSENSEMBLUCSC | 453 | 5 | 30134 | gene;island | 0.75815 | 0.44115 | Not queried | 2.29 | 7.94e-03 | 1.00e+00 |
|  | 16 | 28075183 | 28075295 | PDFSTATSENSEMBLUCSC | 113 | 4 | 15122 | tss;island | 0.53496 | 0.18045 | Not queried | 2.28 | 2.00e-01 | 1.00e+00 |
|  | 6 | 34984836 | 34984953 | PDFSTATSENSEMBLUCSC | 118 | 4 | 34243 | gene | 0.78454 | 0.91205 | Not queried | 2.28 | 1.14e-01 | 1.00e+00 |
|  | 10 | 1401818 | 1402293 | PDFSTATSENSEMBLUCSC | 476 | 5 | 4447 | gene;island | 0.56514 | 0.86329 | Not queried | 2.28 | 1.51e-01 | 1.00e+00 |
|  | X | 34960814 | 34960893 | PDFSTATSENSEMBLUCSC | 80 | 5 | 41250 | tss | 0.62988 | 0.91450 | Not queried | 2.28 | 7.94e-03 | 1.00e+00 |
|  | 3 | 10547179 | 10547584 | PDFSTATSENSEMBLUCSC | 406 | 8 | 28835 | island | 0.19992 | 0.60576 | Not queried | 2.28 | 1.04e-02 | 1.00e+00 |
|  | 19 | 38182773 | 38183456 | PDFSTATSENSEMBLUCSC | 684 | 11 | 21925 | island | 0.16685 | 0.04677 | ZFP30; ZNF781; CTD-2528L19.4; CTD-2528L19.3 (margin: ZFP30; ZNF781; CTD-2528L19.4; CTD-2528L19.3; ZNF607) | 2.28 | 2.84e-06 | 1.46e-02 |
|  | 19 | 38182773 | 38183210 | PDFSTATSENSEMBLUCSC | 438 | 5 | 19974 | gene | 0.13469 | 0.04240 | Not queried | 2.28 | 7.94e-03 | 1.00e+00 |
|  | 6 | 30135449 | 30135671 | PDFSTATSENSEMBLUCSC | 223 | 4 | 33674 | gene | 0.23924 | 0.59678 | Not queried | 2.28 | 2.86e-02 | 1.00e+00 |
|  | 16 | 30023400 | 30023780 | PDFSTATSENSEMBLUCSC | 381 | 4 | 15145 | tss;island | 0.85412 | 0.55303 | Not queried | 2.28 | 2.86e-02 | 1.00e+00 |
|  | 16 | 89070647 | 89070903 | PDFSTATSENSEMBLUCSC | 257 | 4 | 16199 | island | 0.55729 | 0.84316 | Not queried | 2.27 | 2.86e-02 | 1.00e+00 |
|  | 7 | 50849639 | 50849931 | PDFSTATSENSEMBLUCSC | 293 | 4 | 36952 | gene;island | 0.25460 | 0.11030 | Not queried | 2.27 | 3.43e-01 | 1.00e+00 |
|  | 2 | 201980838 | 201980910 | PDFSTATSENSEMBLUCSC | 73 | 4 | 22948 | gene | 0.08860 | 0.01798 | Not queried | 2.27 | 2.86e-02 | 1.00e+00 |
|  | 15 | 98196022 | 98196333 | PDFSTATSENSEMBLUCSC | 312 | 5 | 14464 | island | 0.04600 | 0.31301 | Not queried | 2.27 | 9.52e-02 | 1.00e+00 |
|  | 7 | 157179773 | 157180124 | PDFSTATSENSEMBLUCSC | 352 | 6 | 37257 | gene | 0.60786 | 0.88057 | Not queried | 2.27 | 3.94e-01 | 1.00e+00 |
|  | 3 | 46759335 | 46759698 | PDFSTATSENSEMBLUCSC | 364 | 8 | 29016 | island | 0.53013 | 0.87586 | Not queried | 2.27 | 4.99e-02 | 1.00e+00 |
|  | 6 | 32809960 | 32810258 | PDFSTATSENSEMBLUCSC | 299 | 5 | 34084 | gene;island | 0.91004 | 0.66207 | Not queried | 2.27 | 9.52e-02 | 1.00e+00 |
|  | X | 69509542 | 69509790 | PDFSTATSENSEMBLUCSC | 249 | 7 | 41015 | gene | 0.09616 | 0.02773 | Not queried | 2.27 | 2.62e-02 | 1.00e+00 |
|  | 19 | 54177144 | 54177261 | PDFSTATSENSEMBLUCSC | 118 | 4 | 20998 | tss | 0.62871 | 0.89993 | Not queried | 2.27 | 2.86e-02 | 1.00e+00 |
|  | 6 | 33158503 | 33158597 | PDFSTATSENSEMBLUCSC | 95 | 4 | 34149 | gene;island | 0.90333 | 0.57946 | Not queried | 2.27 | 2.00e-01 | 1.00e+00 |
|  | 19 | 15122135 | 15122224 | PDFSTATSENSEMBLUCSC | 90 | 4 | 19862 | gene;island | 0.12418 | 0.40644 | Not queried | 2.27 | 5.71e-02 | 1.00e+00 |
|  | 1 | 89664260 | 89664582 | PDFSTATSENSEMBLUCSC | 323 | 6 | 618 | gene | 0.05962 | 0.23833 | Not queried | 2.27 | 2.16e-03 | 1.00e+00 |
|  | 3 | 36986555 | 36987040 | PDFSTATSENSEMBLUCSC | 486 | 8 | 28177 | tss | 0.44102 | 0.21763 | Not queried | 2.27 | 1.61e-01 | 1.00e+00 |
|  | 10 | 102508286 | 102508633 | PDFSTATSENSEMBLUCSC | 348 | 5 | 4701 | gene;island | 0.03527 | 0.10081 | Not queried | 2.27 | 9.52e-02 | 1.00e+00 |
|  | 18 | 61144150 | 61144339 | PDFSTATSENSEMBLUCSC | 190 | 4 | 19088 | gene | 0.42986 | 0.79913 | Not queried | 2.26 | 5.71e-02 | 1.00e+00 |
|  | X | 69674800 | 69675064 | PDFSTATSENSEMBLUCSC | 265 | 4 | 41018 | gene;island | 0.06278 | 0.02370 | Not queried | 2.26 | 3.43e-01 | 1.00e+00 |
|  | 16 | 89632574 | 89632749 | PDFSTATSENSEMBLUCSC | 176 | 5 | 16228 | island | 0.61335 | 0.94758 | Not queried | 2.26 | 3.17e-02 | 1.00e+00 |
|  | 19 | 11353961 | 11354401 | PDFSTATSENSEMBLUCSC | 441 | 5 | 19796 | gene;island | 0.16813 | 0.12168 | Not queried | 2.26 | 4.21e-01 | 1.00e+00 |
|  | 16 | 51187388 | 51187807 | PDFSTATSENSEMBLUCSC | 420 | 5 | 15888 | island | 0.12005 | 0.03091 | Not queried | 2.26 | 7.94e-03 | 1.00e+00 |
|  | 15 | 75628126 | 75628370 | PDFSTATSENSEMBLUCSC | 245 | 6 | 13782 | tss | 0.05244 | 0.26017 | Not queried | 2.26 | 1.32e-01 | 1.00e+00 |
|  | 12 | 54446253 | 54446576 | PDFSTATSENSEMBLUCSC | 324 | 7 | 9731 | tss | 0.78008 | 0.41123 | Not queried | 2.26 | 5.30e-02 | 1.00e+00 |
|  | 10 | 32734661 | 32734958 | PDFSTATSENSEMBLUCSC | 298 | 7 | 5005 | tss | 0.06221 | 0.02296 | Not queried | 2.26 | 5.30e-02 | 1.00e+00 |
|  | 6 | 28972876 | 28972975 | PDFSTATSENSEMBLUCSC | 100 | 5 | 33586 | gene | 0.85848 | 0.27809 | Not queried | 2.26 | 5.56e-02 | 1.00e+00 |
|  | 10 | 23463015 | 23463377 | PDFSTATSENSEMBLUCSC | 363 | 4 | 5590 | island | 0.02473 | 0.10453 | Not queried | 2.26 | 2.86e-02 | 1.00e+00 |
|  | 16 | 1128689 | 1128865 | PDFSTATSENSEMBLUCSC | 177 | 5 | 14956 | tss;island | 0.50340 | 0.82924 | Not queried | 2.26 | 7.94e-03 | 1.00e+00 |
|  | 1 | 229569608 | 229570288 | PDFSTATSENSEMBLUCSC | 681 | 14 | 4297 | island | 0.15068 | 0.46250 | Not queried | 2.26 | 1.73e-03 | 1.00e+00 |
|  | 18 | 77203371 | 77203667 | PDFSTATSENSEMBLUCSC | 297 | 4 | 19106 | gene;island | 0.44896 | 0.80766 | Not queried | 2.26 | 2.86e-02 | 1.00e+00 |
|  | 17 | 40337534 | 40337892 | PDFSTATSENSEMBLUCSC | 359 | 6 | 17324 | tss | 0.79698 | 0.45053 | Not queried | 2.26 | 2.16e-03 | 1.00e+00 |
|  | 4 | 57547347 | 57547699 | PDFSTATSENSEMBLUCSC | 353 | 4 | 29966 | gene | 0.22367 | 0.51901 | Not queried | 2.26 | 2.86e-02 | 1.00e+00 |
|  | 4 | 46391159 | 46391929 | PDFSTATSENSEMBLUCSC | 771 | 14 | 29950 | gene;island | 0.03489 | 0.08291 | Not queried | 2.26 | 5.76e-03 | 1.00e+00 |
|  | 14 | 64319089 | 64319306 | PDFSTATSENSEMBLUCSC | 218 | 4 | 12424 | tss;island | 0.38284 | 0.14012 | Not queried | 2.25 | 4.86e-01 | 1.00e+00 |
|  | 5 | 523430 | 523794 | PDFSTATSENSEMBLUCSC | 365 | 4 | 31457 | gene;island | 0.33845 | 0.72036 | Not queried | 2.25 | 2.86e-02 | 1.00e+00 |
|  | 19 | 52872916 | 52873125 | PDFSTATSENSEMBLUCSC | 210 | 6 | 20976 | tss | 0.04466 | 0.18560 | Not queried | 2.25 | 2.60e-02 | 1.00e+00 |
|  | 16 | 64984626 | 64984977 | PDFSTATSENSEMBLUCSC | 352 | 5 | 14747 | gene;island | 0.61315 | 0.87684 | Not queried | 2.25 | 9.52e-02 | 1.00e+00 |
|  | 20 | 44098223 | 44098387 | PDFSTATSENSEMBLUCSC | 165 | 6 | 25737 | tss | 0.22702 | 0.58016 | Not queried | 2.25 | 2.16e-03 | 1.00e+00 |
|  | 11 | 62647979 | 62648405 | PDFSTATSENSEMBLUCSC | 427 | 7 | 6634 | gene;island | 0.02326 | 0.14199 | Not queried | 2.25 | 4.08e-03 | 1.00e+00 |
|  | 12 | 124990897 | 124991139 | PDFSTATSENSEMBLUCSC | 243 | 4 | 9373 | gene | 0.69675 | 0.91881 | Not queried | 2.25 | 2.86e-02 | 1.00e+00 |
|  | 7 | 143582146 | 143582630 | PDFSTATSENSEMBLUCSC | 485 | 4 | 37193 | gene;island | 0.06857 | 0.25214 | Not queried | 2.25 | 2.86e-02 | 1.00e+00 |
|  | 8 | 599963 | 600555 | PDFSTATSENSEMBLUCSC | 593 | 6 | 39939 | island | 0.62697 | 0.87572 | Not queried | 2.25 | 4.33e-03 | 1.00e+00 |
|  | 19 | 49249932 | 49250561 | PDFSTATSENSEMBLUCSC | 630 | 7 | 22198 | island | 0.15454 | 0.03706 | Not queried | 2.25 | 1.75e-02 | 1.00e+00 |
|  | 17 | 43047405 | 43047971 | PDFSTATSENSEMBLUCSC | 567 | 5 | 18384 | island | 0.43678 | 0.06712 | Not queried | 2.25 | 4.21e-01 | 1.00e+00 |
|  | 17 | 42247565 | 42248062 | PDFSTATSENSEMBLUCSC | 498 | 6 | 17370 | tss | 0.42471 | 0.75505 | Not queried | 2.25 | 1.32e-01 | 1.00e+00 |
|  | 16 | 31053788 | 31054123 | PDFSTATSENSEMBLUCSC | 336 | 4 | 15831 | island | 0.39965 | 0.73369 | Not queried | 2.25 | 2.86e-02 | 1.00e+00 |
|  | 4 | 80329388 | 80329633 | PDFSTATSENSEMBLUCSC | 246 | 8 | 30410 | tss | 0.55308 | 0.85975 | Not queried | 2.25 | 2.81e-02 | 1.00e+00 |
|  | 6 | 33039396 | 33039804 | PDFSTATSENSEMBLUCSC | 409 | 7 | 34117 | gene | 0.89351 | 0.56630 | Not queried | 2.25 | 3.79e-02 | 1.00e+00 |
|  | 17 | 37879654 | 37879858 | PDFSTATSENSEMBLUCSC | 205 | 4 | 16519 | gene;island | 0.66219 | 0.89829 | Not queried | 2.25 | 2.86e-02 | 1.00e+00 |
|  | 22 | 21321253 | 21321585 | PDFSTATSENSEMBLUCSC | 333 | 5 | 26761 | gene;island | 0.62558 | 0.17075 | Not queried | 2.24 | 1.51e-01 | 1.00e+00 |
|  | 4 | 7194841 | 7195206 | PDFSTATSENSEMBLUCSC | 366 | 5 | 29890 | gene;island | 0.11016 | 0.00867 | Not queried | 2.24 | 1.51e-01 | 1.00e+00 |
|  | 18 | 712731 | 712795 | PDFSTATSENSEMBLUCSC | 65 | 4 | 19122 | tss;island | 0.01688 | 0.06855 | Not queried | 2.24 | 1.14e-01 | 1.00e+00 |
|  | X | 51636562 | 51636681 | PDFSTATSENSEMBLUCSC | 120 | 6 | 41325 | tss | 0.10082 | 0.02292 | Not queried | 2.24 | 1.32e-01 | 1.00e+00 |
|  | 2 | 66810341 | 66810606 | PDFSTATSENSEMBLUCSC | 266 | 4 | 24321 | island | 0.21780 | 0.50103 | Not queried | 2.24 | 2.86e-02 | 1.00e+00 |
|  | 7 | 1277521 | 1277790 | PDFSTATSENSEMBLUCSC | 270 | 4 | 38028 | island | 0.12351 | 0.31936 | Not queried | 2.24 | 2.00e-01 | 1.00e+00 |
|  | 3 | 42543933 | 42544131 | PDFSTATSENSEMBLUCSC | 199 | 7 | 28970 | island | 0.14458 | 0.02720 | Not queried | 2.24 | 6.99e-03 | 1.00e+00 |
|  | 5 | 76115672 | 76116169 | PDFSTATSENSEMBLUCSC | 498 | 6 | 31595 | gene;island | 0.01159 | 0.08054 | Not queried | 2.24 | 8.66e-03 | 1.00e+00 |
|  | 10 | 100993553 | 100994143 | PDFSTATSENSEMBLUCSC | 591 | 11 | 4692 | gene;island | 0.12971 | 0.03796 | Not queried | 2.24 | 5.24e-03 | 1.00e+00 |
|  | 7 | 936081 | 936591 | PDFSTATSENSEMBLUCSC | 511 | 5 | 37994 | island | 0.83141 | 0.94868 | Not queried | 2.24 | 3.17e-02 | 1.00e+00 |
|  | 13 | 52419332 | 52419483 | PDFSTATSENSEMBLUCSC | 152 | 5 | 11426 | tss | 0.02801 | 0.13878 | Not queried | 2.24 | 7.94e-03 | 1.00e+00 |
|  | 1 | 153940090 | 153940616 | PDFSTATSENSEMBLUCSC | 527 | 7 | 1954 | tss;island | 0.15295 | 0.03474 | Not queried | 2.24 | 1.11e-02 | 1.00e+00 |
|  | 3 | 181413810 | 181414237 | PDFSTATSENSEMBLUCSC | 428 | 6 | 27997 | gene;island | 0.50439 | 0.84800 | Not queried | 2.24 | 4.33e-03 | 1.00e+00 |
|  | 8 | 9008170 | 9008681 | PDFSTATSENSEMBLUCSC | 512 | 7 | 39997 | island | 0.08818 | 0.04518 | Not queried | 2.24 | 7.28e-02 | 1.00e+00 |
|  | 1 | 203456691 | 203457150 | PDFSTATSENSEMBLUCSC | 460 | 6 | 949 | gene;island | 0.02077 | 0.08249 | Not queried | 2.24 | 4.11e-02 | 1.00e+00 |
|  | 2 | 132056834 | 132057166 | PDFSTATSENSEMBLUCSC | 333 | 4 | 22833 | gene;island | 0.50230 | 0.86142 | Not queried | 2.24 | 2.86e-02 | 1.00e+00 |
|  | 20 | 57607335 | 57607420 | PDFSTATSENSEMBLUCSC | 86 | 4 | 25442 | gene | 0.03997 | 0.17483 | Not queried | 2.24 | 2.00e-01 | 1.00e+00 |
|  | 19 | 58629828 | 58630612 | PDFSTATSENSEMBLUCSC | 785 | 9 | 21097 | tss | 0.47268 | 0.70367 | Not queried | 2.23 | 1.36e-01 | 1.00e+00 |
|  | 17 | 26732120 | 26732374 | PDFSTATSENSEMBLUCSC | 255 | 5 | 16460 | gene;island | 0.63900 | 0.48040 | Not queried | 2.23 | 1.59e-02 | 1.00e+00 |
|  | 19 | 13215041 | 13215387 | PDFSTATSENSEMBLUCSC | 347 | 5 | 21608 | island | 0.63476 | 0.86848 | Not queried | 2.23 | 2.22e-01 | 1.00e+00 |
|  | 11 | 8954570 | 8954606 | PDFSTATSENSEMBLUCSC | 37 | 4 | 7110 | tss | 0.47881 | 0.79963 | Not queried | 2.23 | 2.86e-02 | 1.00e+00 |
|  | 17 | 46507549 | 46507895 | PDFSTATSENSEMBLUCSC | 347 | 8 | 18438 | island | 0.28458 | 0.62651 | Not queried | 2.23 | 2.07e-02 | 1.00e+00 |
|  | 20 | 56725695 | 56726185 | PDFSTATSENSEMBLUCSC | 491 | 11 | 26246 | island | 0.40501 | 0.80101 | Not queried | 2.23 | 1.28e-04 | 6.56e-01 |
|  | 11 | 134126284 | 134126504 | PDFSTATSENSEMBLUCSC | 221 | 4 | 6973 | gene;island | 0.75846 | 0.88802 | Not queried | 2.23 | 2.00e-01 | 1.00e+00 |
|  | 11 | 128562003 | 128562408 | PDFSTATSENSEMBLUCSC | 406 | 7 | 7780 | tss;island | 0.20413 | 0.05251 | Not queried | 2.23 | 2.33e-03 | 1.00e+00 |
|  | 2 | 1595734 | 1596054 | PDFSTATSENSEMBLUCSC | 321 | 4 | 23978 | island | 0.25805 | 0.60592 | Not queried | 2.23 | 5.71e-02 | 1.00e+00 |
|  | X | 17879602 | 17879951 | PDFSTATSENSEMBLUCSC | 350 | 5 | 41211 | tss | 0.07303 | 0.06498 | Not queried | 2.23 | 2.22e-01 | 1.00e+00 |
|  | 1 | 20446641 | 20446899 | PDFSTATSENSEMBLUCSC | 259 | 4 | 1308 | tss | 0.94336 | 0.75716 | Not queried | 2.23 | 1.14e-01 | 1.00e+00 |
|  | 11 | 96076205 | 96076302 | PDFSTATSENSEMBLUCSC | 98 | 4 | 6854 | gene | 0.12337 | 0.13503 | Not queried | 2.23 | 6.86e-01 | 1.00e+00 |
|  | 18 | 44237002 | 44237151 | PDFSTATSENSEMBLUCSC | 150 | 6 | 19225 | tss | 0.59754 | 0.87317 | Not queried | 2.22 | 2.60e-02 | 1.00e+00 |
|  | 5 | 140588139 | 140588424 | PDFSTATSENSEMBLUCSC | 286 | 5 | 33162 | island | 0.07307 | 0.32109 | Not queried | 2.22 | 5.56e-02 | 1.00e+00 |
|  | X | 30265129 | 30265251 | PDFSTATSENSEMBLUCSC | 123 | 6 | 41243 | tss | 0.76653 | 0.93398 | Not queried | 2.22 | 8.66e-03 | 1.00e+00 |
|  | 22 | 49717382 | 49717821 | PDFSTATSENSEMBLUCSC | 440 | 5 | 27562 | island | 0.46099 | 0.60638 | Not queried | 2.22 | 5.56e-02 | 1.00e+00 |
|  | 5 | 78365647 | 78365801 | PDFSTATSENSEMBLUCSC | 155 | 5 | 31603 | gene;tss;island | 0.69363 | 0.93491 | Not queried | 2.22 | 7.94e-03 | 1.00e+00 |
|  | 5 | 140165938 | 140166213 | PDFSTATSENSEMBLUCSC | 276 | 4 | 31736 | gene;island | 0.55874 | 0.85529 | Not queried | 2.22 | 5.71e-02 | 1.00e+00 |
|  | 5 | 131892956 | 131893381 | PDFSTATSENSEMBLUCSC | 426 | 5 | 31689 | gene;island | 0.23557 | 0.64042 | Not queried | 2.22 | 3.17e-02 | 1.00e+00 |
|  | 15 | 40697419 | 40698044 | PDFSTATSENSEMBLUCSC | 626 | 8 | 14009 | island | 0.06271 | 0.11847 | Not queried | 2.22 | 1.95e-01 | 1.00e+00 |
|  | 19 | 15580310 | 15580445 | PDFSTATSENSEMBLUCSC | 136 | 4 | 19872 | gene;island | 0.48001 | 0.10240 | Not queried | 2.22 | 2.00e-01 | 1.00e+00 |
|  | 8 | 11565277 | 11565725 | PDFSTATSENSEMBLUCSC | 449 | 4 | 39142 | gene;island | 0.52202 | 0.18935 | Not queried | 2.22 | 2.86e-02 | 1.00e+00 |
|  | 17 | 80291982 | 80292252 | PDFSTATSENSEMBLUCSC | 271 | 4 | 17740 | tss;island | 0.64342 | 0.89120 | Not queried | 2.22 | 3.43e-01 | 1.00e+00 |
|  | 20 | 39321346 | 39321729 | PDFSTATSENSEMBLUCSC | 384 | 4 | 26124 | island | 0.25571 | 0.51906 | Not queried | 2.22 | 2.86e-02 | 1.00e+00 |
|  | 22 | 19137874 | 19138267 | PDFSTATSENSEMBLUCSC | 394 | 5 | 26914 | tss;island | 0.78066 | 0.35982 | Not queried | 2.22 | 3.10e-01 | 1.00e+00 |
|  | 14 | 36983404 | 36983949 | PDFSTATSENSEMBLUCSC | 546 | 7 | 12329 | tss;island | 0.89401 | 0.51136 | Not queried | 2.22 | 6.99e-03 | 1.00e+00 |
|  | 20 | 62693943 | 62694005 | PDFSTATSENSEMBLUCSC | 63 | 6 | 25478 | gene;tss;island | 0.52172 | 0.19354 | Not queried | 2.22 | 2.60e-02 | 1.00e+00 |
|  | 15 | 63570099 | 63570580 | PDFSTATSENSEMBLUCSC | 482 | 5 | 13345 | gene;island | 0.01880 | 0.21883 | Not queried | 2.21 | 1.51e-01 | 1.00e+00 |
|  | 10 | 71562314 | 71562858 | PDFSTATSENSEMBLUCSC | 545 | 4 | 4602 | gene;island | 0.23720 | 0.06755 | Not queried | 2.21 | 3.43e-01 | 1.00e+00 |
|  | 20 | 62688573 | 62688896 | PDFSTATSENSEMBLUCSC | 324 | 4 | 25477 | gene;island | 0.52800 | 0.80970 | Not queried | 2.21 | 2.86e-02 | 1.00e+00 |
|  | 19 | 35800589 | 35801014 | PDFSTATSENSEMBLUCSC | 426 | 4 | 19948 | gene;island | 0.41175 | 0.79196 | Not queried | 2.21 | 2.00e-01 | 1.00e+00 |
|  | 3 | 49203799 | 49203975 | PDFSTATSENSEMBLUCSC | 177 | 5 | 28280 | tss | 0.70863 | 0.08252 | Not queried | 2.21 | 2.22e-01 | 1.00e+00 |
|  | 6 | 13014871 | 13015221 | PDFSTATSENSEMBLUCSC | 351 | 5 | 33510 | gene;island | 0.02725 | 0.10384 | Not queried | 2.21 | 1.51e-01 | 1.00e+00 |
|  | 17 | 73840822 | 73840989 | PDFSTATSENSEMBLUCSC | 168 | 4 | 17630 | tss | 0.33729 | 0.62514 | Not queried | 2.21 | 2.86e-02 | 1.00e+00 |
|  | 19 | 496239 | 496428 | PDFSTATSENSEMBLUCSC | 190 | 5 | 20236 | tss | 0.84973 | 0.46622 | Not queried | 2.21 | 9.52e-02 | 1.00e+00 |
|  | 11 | 44972672 | 44973218 | PDFSTATSENSEMBLUCSC | 547 | 10 | 7242 | tss | 0.63934 | 0.44278 | Not queried | 2.21 | 2.32e-02 | 1.00e+00 |
|  | 5 | 501863 | 502074 | PDFSTATSENSEMBLUCSC | 212 | 4 | 31456 | gene;island | 0.53641 | 0.90052 | Not queried | 2.21 | 5.71e-02 | 1.00e+00 |
|  | 12 | 54357408 | 54357771 | PDFSTATSENSEMBLUCSC | 364 | 5 | 9130 | gene;island | 0.72235 | 0.23045 | Not queried | 2.21 | 3.17e-02 | 1.00e+00 |
|  | 6 | 28973318 | 28973524 | PDFSTATSENSEMBLUCSC | 207 | 11 | 34682 | tss | 0.02329 | 0.10097 | Not queried | 2.21 | 3.40e-05 | 1.75e-01 |
|  | X | 48432562 | 48433316 | PDFSTATSENSEMBLUCSC | 755 | 9 | 41765 | island | 0.20250 | 0.56956 | Not queried | 2.21 | 7.77e-03 | 1.00e+00 |
|  | 11 | 93822022 | 93822294 | PDFSTATSENSEMBLUCSC | 273 | 4 | 6844 | gene;island | 0.46343 | 0.82847 | Not queried | 2.21 | 2.86e-02 | 1.00e+00 |
|  | 16 | 20084851 | 20085358 | PDFSTATSENSEMBLUCSC | 508 | 5 | 15711 | island | 0.06218 | 0.04741 | Not queried | 2.20 | 3.10e-01 | 1.00e+00 |
|  | 3 | 50341009 | 50341293 | PDFSTATSENSEMBLUCSC | 285 | 4 | 27759 | gene;tss | 0.88357 | 0.61706 | Not queried | 2.20 | 3.43e-01 | 1.00e+00 |
|  | 10 | 26726877 | 26727318 | PDFSTATSENSEMBLUCSC | 442 | 8 | 5601 | island | 0.18275 | 0.53520 | Not queried | 2.20 | 1.30e-01 | 1.00e+00 |
|  | 4 | 89299733 | 89300321 | PDFSTATSENSEMBLUCSC | 589 | 7 | 31120 | island | 0.26394 | 0.04491 | Not queried | 2.20 | 3.18e-01 | 1.00e+00 |
|  | X | 74493592 | 74493847 | PDFSTATSENSEMBLUCSC | 256 | 5 | 41395 | tss | 0.05568 | 0.02484 | Not queried | 2.20 | 9.52e-02 | 1.00e+00 |
|  | 16 | 85699435 | 85699907 | PDFSTATSENSEMBLUCSC | 473 | 5 | 14848 | gene | 0.50075 | 0.92876 | Not queried | 2.20 | 9.52e-02 | 1.00e+00 |
|  | 12 | 53267981 | 53268433 | PDFSTATSENSEMBLUCSC | 453 | 5 | 10419 | island | 0.03709 | 0.20587 | Not queried | 2.20 | 4.21e-01 | 1.00e+00 |
|  | 17 | 74864041 | 74864302 | PDFSTATSENSEMBLUCSC | 262 | 4 | 17657 | tss;island | 0.41923 | 0.14832 | Not queried | 2.20 | 3.43e-01 | 1.00e+00 |
|  | 1 | 113261488 | 113261894 | PDFSTATSENSEMBLUCSC | 407 | 6 | 3600 | island | 0.06736 | 0.28706 | Not queried | 2.20 | 6.49e-02 | 1.00e+00 |
|  | 17 | 40337411 | 40337892 | PDFSTATSENSEMBLUCSC | 482 | 7 | 18299 | island | 0.79642 | 0.45971 | Not queried | 2.20 | 5.83e-04 | 1.00e+00 |
|  | 4 | 40631571 | 40632020 | PDFSTATSENSEMBLUCSC | 450 | 6 | 30944 | island | 0.00593 | 0.02814 | Not queried | 2.20 | 2.60e-02 | 1.00e+00 |
|  | 11 | 101000566 | 101000717 | PDFSTATSENSEMBLUCSC | 152 | 5 | 7620 | tss | 0.03091 | 0.12783 | Not queried | 2.20 | 3.10e-01 | 1.00e+00 |
|  | 12 | 6756671 | 6756815 | PDFSTATSENSEMBLUCSC | 145 | 4 | 9494 | tss | 0.10667 | 0.37279 | Not queried | 2.20 | 2.00e-01 | 1.00e+00 |
|  | 11 | 65360123 | 65360509 | PDFSTATSENSEMBLUCSC | 387 | 5 | 8445 | island | 0.03937 | 0.29296 | Not queried | 2.20 | 1.51e-01 | 1.00e+00 |
|  | 2 | 45155408 | 45155774 | PDFSTATSENSEMBLUCSC | 367 | 5 | 24220 | island | 0.25607 | 0.08633 | Not queried | 2.20 | 5.56e-02 | 1.00e+00 |
|  | 22 | 25758621 | 25758749 | PDFSTATSENSEMBLUCSC | 129 | 4 | 26988 | tss | 0.48143 | 0.78779 | Not queried | 2.20 | 6.86e-01 | 1.00e+00 |
|  | 3 | 169482895 | 169483295 | PDFSTATSENSEMBLUCSC | 401 | 8 | 28664 | tss | 0.32677 | 0.76452 | Not queried | 2.20 | 3.82e-01 | 1.00e+00 |
|  | 10 | 124909711 | 124910217 | PDFSTATSENSEMBLUCSC | 507 | 4 | 6199 | island | 0.55728 | 0.24503 | Not queried | 2.19 | 3.43e-01 | 1.00e+00 |
|  | 17 | 19647973 | 19648414 | PDFSTATSENSEMBLUCSC | 442 | 4 | 16447 | gene;island | 0.02990 | 0.10060 | Not queried | 2.19 | 3.43e-01 | 1.00e+00 |
|  | 3 | 169487146 | 169487465 | PDFSTATSENSEMBLUCSC | 320 | 4 | 27980 | gene;island | 0.39148 | 0.78815 | Not queried | 2.19 | 2.86e-02 | 1.00e+00 |
|  | 20 | 57428282 | 57428473 | PDFSTATSENSEMBLUCSC | 192 | 6 | 25434 | gene;island | 0.89220 | 0.40937 | Not queried | 2.19 | 4.11e-02 | 1.00e+00 |
|  | 15 | 73661642 | 73662002 | PDFSTATSENSEMBLUCSC | 361 | 6 | 13753 | tss | 0.19206 | 0.06028 | Not queried | 2.19 | 1.32e-01 | 1.00e+00 |
|  | 14 | 37135384 | 37135671 | PDFSTATSENSEMBLUCSC | 288 | 4 | 12039 | gene;island | 0.47730 | 0.15197 | Not queried | 2.19 | 2.86e-02 | 1.00e+00 |
|  | 20 | 62795341 | 62795807 | PDFSTATSENSEMBLUCSC | 467 | 5 | 25876 | tss | 0.47673 | 0.74706 | Not queried | 2.19 | 9.52e-02 | 1.00e+00 |
|  | 1 | 148000260 | 148000861 | PDFSTATSENSEMBLUCSC | 602 | 5 | 3692 | island | 0.23872 | 0.05070 | Not queried | 2.19 | 1.51e-01 | 1.00e+00 |
|  | 2 | 10712018 | 10712241 | PDFSTATSENSEMBLUCSC | 224 | 4 | 22531 | gene | 0.72983 | 0.91318 | Not queried | 2.19 | 4.86e-01 | 1.00e+00 |
|  | 17 | 79380400 | 79380706 | PDFSTATSENSEMBLUCSC | 307 | 5 | 16874 | gene;island | 0.58597 | 0.85737 | Not queried | 2.19 | 2.22e-01 | 1.00e+00 |
|  | 12 | 132865182 | 132865474 | PDFSTATSENSEMBLUCSC | 293 | 4 | 9420 | gene;island | 0.51589 | 0.90629 | Not queried | 2.19 | 1.14e-01 | 1.00e+00 |
|  | 8 | 80523191 | 80523364 | PDFSTATSENSEMBLUCSC | 174 | 4 | 39716 | tss | 0.26789 | 0.09168 | Not queried | 2.19 | 1.14e-01 | 1.00e+00 |
|  | 8 | 80523191 | 80523461 | PDFSTATSENSEMBLUCSC | 271 | 6 | 40343 | island | 0.24660 | 0.08469 | Not queried | 2.19 | 8.66e-03 | 1.00e+00 |
|  | 16 | 1226067 | 1226302 | PDFSTATSENSEMBLUCSC | 236 | 4 | 14529 | gene;island | 0.53214 | 0.82594 | Not queried | 2.19 | 1.14e-01 | 1.00e+00 |
|  | 11 | 67764948 | 67765204 | PDFSTATSENSEMBLUCSC | 257 | 4 | 6747 | gene;island | 0.61688 | 0.73782 | Not queried | 2.19 | 6.86e-01 | 1.00e+00 |
|  | 11 | 1036714 | 1036938 | PDFSTATSENSEMBLUCSC | 225 | 6 | 7016 | tss | 0.63765 | 0.87567 | Not queried | 2.19 | 2.16e-03 | 1.00e+00 |
|  | 10 | 129535138 | 129535968 | PDFSTATSENSEMBLUCSC | 831 | 11 | 6230 | island | 0.18260 | 0.47061 | Not queried | 2.18 | 2.33e-02 | 1.00e+00 |
|  | 1 | 209825672 | 209825943 | PDFSTATSENSEMBLUCSC | 272 | 7 | 2226 | tss | 0.55667 | 0.80203 | Not queried | 2.18 | 6.99e-03 | 1.00e+00 |
|  | 19 | 12948848 | 12949060 | PDFSTATSENSEMBLUCSC | 213 | 4 | 20480 | tss;island | 0.19749 | 0.02716 | Not queried | 2.18 | 6.86e-01 | 1.00e+00 |
|  | 5 | 76936361 | 76936793 | PDFSTATSENSEMBLUCSC | 433 | 4 | 32840 | island | 0.12908 | 0.03179 | Not queried | 2.18 | 5.71e-02 | 1.00e+00 |
|  | 17 | 40346279 | 40346798 | PDFSTATSENSEMBLUCSC | 520 | 8 | 17325 | tss | 0.07050 | 0.32077 | Not queried | 2.18 | 4.99e-02 | 1.00e+00 |
|  | 22 | 42062693 | 42063028 | PDFSTATSENSEMBLUCSC | 336 | 4 | 27484 | island | 0.01801 | 0.07460 | Not queried | 2.18 | 5.71e-02 | 1.00e+00 |
|  | 11 | 32449163 | 32450244 | PDFSTATSENSEMBLUCSC | 1082 | 8 | 6547 | gene;island | 0.12826 | 0.51338 | Not queried | 2.18 | 2.79e-01 | 1.00e+00 |
|  | 2 | 201980504 | 201980910 | PDFSTATSENSEMBLUCSC | 407 | 8 | 24968 | island | 0.31959 | 0.16124 | Not queried | 2.18 | 1.95e-01 | 1.00e+00 |
|  | 2 | 129494526 | 129495163 | PDFSTATSENSEMBLUCSC | 638 | 6 | 24672 | island | 0.58585 | 0.80755 | Not queried | 2.18 | 9.31e-02 | 1.00e+00 |
|  | 2 | 24397539 | 24397845 | PDFSTATSENSEMBLUCSC | 307 | 5 | 23187 | tss;island | 0.69216 | 0.94201 | Not queried | 2.18 | 9.52e-02 | 1.00e+00 |
|  | 1 | 79472532 | 79472643 | PDFSTATSENSEMBLUCSC | 112 | 4 | 1708 | tss | 0.23461 | 0.06392 | Not queried | 2.18 | 2.86e-02 | 1.00e+00 |
|  | 14 | 29254530 | 29255119 | PDFSTATSENSEMBLUCSC | 590 | 6 | 12026 | gene;island | 0.21266 | 0.04692 | Not queried | 2.18 | 6.49e-02 | 1.00e+00 |
|  | 6 | 29894619 | 29895260 | PDFSTATSENSEMBLUCSC | 642 | 26 | 35580 | island | 0.08022 | 0.31167 | Not queried | 2.18 | 2.44e-03 | 1.00e+00 |
|  | X | 70288342 | 70288735 | PDFSTATSENSEMBLUCSC | 394 | 8 | 41371 | tss | 0.17891 | 0.03602 | Not queried | 2.18 | 1.09e-03 | 1.00e+00 |
|  | 1 | 33351568 | 33351946 | PDFSTATSENSEMBLUCSC | 379 | 6 | 1462 | tss;island | 0.04009 | 0.18720 | Not queried | 2.18 | 4.11e-02 | 1.00e+00 |
|  | 5 | 1394296 | 1394633 | PDFSTATSENSEMBLUCSC | 338 | 5 | 31482 | gene | 0.58587 | 0.86139 | Not queried | 2.18 | 2.22e-01 | 1.00e+00 |
|  | 3 | 42947481 | 42947771 | PDFSTATSENSEMBLUCSC | 291 | 4 | 27694 | gene | 0.71263 | 0.91243 | Not queried | 2.18 | 2.86e-02 | 1.00e+00 |
|  | 16 | 84328104 | 84328391 | PDFSTATSENSEMBLUCSC | 288 | 6 | 15362 | tss;island | 0.29301 | 0.57490 | Not queried | 2.18 | 2.60e-02 | 1.00e+00 |
|  | 11 | 116371188 | 116371487 | PDFSTATSENSEMBLUCSC | 300 | 4 | 8808 | island | 0.92918 | 0.76960 | Not queried | 2.18 | 2.86e-02 | 1.00e+00 |
|  | 12 | 95009858 | 95010113 | PDFSTATSENSEMBLUCSC | 256 | 5 | 9225 | gene | 0.41958 | 0.80960 | Not queried | 2.17 | 1.59e-02 | 1.00e+00 |
|  | 8 | 42623686 | 42623946 | PDFSTATSENSEMBLUCSC | 261 | 6 | 39617 | tss | 0.84220 | 0.91993 | Not queried | 2.17 | 1.32e-01 | 1.00e+00 |
|  | 17 | 79259283 | 79259699 | PDFSTATSENSEMBLUCSC | 417 | 4 | 16872 | gene;island | 0.67969 | 0.89266 | Not queried | 2.17 | 2.86e-02 | 1.00e+00 |
|  | 11 | 66059064 | 66059164 | PDFSTATSENSEMBLUCSC | 101 | 4 | 7442 | tss;island | 0.14810 | 0.06115 | Not queried | 2.17 | 1.14e-01 | 1.00e+00 |
|  | 8 | 143761503 | 143761811 | PDFSTATSENSEMBLUCSC | 309 | 6 | 39868 | tss | 0.38208 | 0.72665 | Not queried | 2.17 | 4.11e-02 | 1.00e+00 |
|  | 12 | 113909932 | 113910034 | PDFSTATSENSEMBLUCSC | 103 | 6 | 10005 | tss;island | 0.31995 | 0.08490 | Not queried | 2.17 | 2.60e-02 | 1.00e+00 |
|  | 6 | 33160067 | 33160604 | PDFSTATSENSEMBLUCSC | 538 | 15 | 35906 | island | 0.12477 | 0.38724 | COL11A2; RXRB (margin: RNY4P10; SLC39A7; HSD17B8; COL11A2; RXRB) | 2.17 | 8.82e-06 | 4.53e-02 |
|  | 3 | 37033625 | 37035399 | PDFSTATSENSEMBLUCSC | 1775 | 43 | 28178 | tss;island | 0.15867 | 0.01963 | MLH1; EPM2AIP1 (margin: MLH1; RP11-129K12.4; RP11-129K12.3; EPM2AIP1) | 2.17 | 1.87e-09 | 9.60e-06 |
|  | 19 | 52872916 | 52873624 | PDFSTATSENSEMBLUCSC | 709 | 10 | 22321 | island | 0.03657 | 0.16833 | Not queried | 2.17 | 1.50e-03 | 1.00e+00 |
|  | 18 | 21852118 | 21852436 | PDFSTATSENSEMBLUCSC | 319 | 6 | 19045 | gene;island | 0.01826 | 0.08311 | Not queried | 2.17 | 4.11e-02 | 1.00e+00 |
|  | 6 | 134209935 | 134210307 | PDFSTATSENSEMBLUCSC | 373 | 9 | 36427 | island | 0.12941 | 0.05087 | Not queried | 2.17 | 1.85e-03 | 1.00e+00 |
|  | 6 | 10521142 | 10521558 | PDFSTATSENSEMBLUCSC | 417 | 4 | 34562 | tss | 0.01500 | 0.06216 | Not queried | 2.17 | 2.00e-01 | 1.00e+00 |
|  | 14 | 38065127 | 38065582 | PDFSTATSENSEMBLUCSC | 456 | 6 | 12335 | tss;island | 0.03982 | 0.17703 | Not queried | 2.17 | 6.49e-02 | 1.00e+00 |
|  | 10 | 134859687 | 134860210 | PDFSTATSENSEMBLUCSC | 524 | 7 | 6310 | island | 0.58109 | 0.87324 | Not queried | 2.17 | 9.73e-02 | 1.00e+00 |
|  | 19 | 496239 | 496528 | PDFSTATSENSEMBLUCSC | 290 | 6 | 21126 | island | 0.86811 | 0.55396 | Not queried | 2.17 | 4.11e-02 | 1.00e+00 |
|  | 7 | 1753396 | 1753659 | PDFSTATSENSEMBLUCSC | 264 | 6 | 36703 | gene;island | 0.72256 | 0.37436 | Not queried | 2.17 | 2.60e-02 | 1.00e+00 |
|  | 17 | 75451779 | 75452100 | PDFSTATSENSEMBLUCSC | 322 | 5 | 16774 | gene | 0.60470 | 0.88063 | Not queried | 2.17 | 7.94e-03 | 1.00e+00 |
|  | 6 | 31324997 | 31325455 | PDFSTATSENSEMBLUCSC | 459 | 11 | 34743 | tss | 0.14153 | 0.03061 | Not queried | 2.16 | 1.04e-02 | 1.00e+00 |
|  | 22 | 37099037 | 37099259 | PDFSTATSENSEMBLUCSC | 223 | 4 | 27069 | tss;island | 0.04690 | 0.08786 | Not queried | 2.16 | 3.43e-01 | 1.00e+00 |
|  | 18 | 77125738 | 77125858 | PDFSTATSENSEMBLUCSC | 121 | 4 | 19102 | gene;island | 0.73688 | 0.91542 | Not queried | 2.16 | 2.86e-02 | 1.00e+00 |
|  | 12 | 57619178 | 57619401 | PDFSTATSENSEMBLUCSC | 224 | 5 | 9164 | gene;island | 0.64128 | 0.38736 | Not queried | 2.16 | 1.51e-01 | 1.00e+00 |
|  | X | 48858679 | 48858921 | PDFSTATSENSEMBLUCSC | 243 | 9 | 41304 | tss | 0.05618 | 0.01117 | Not queried | 2.16 | 3.99e-03 | 1.00e+00 |
|  | 4 | 46391414 | 46391929 | PDFSTATSENSEMBLUCSC | 516 | 11 | 30325 | tss | 0.03441 | 0.08096 | Not queried | 2.16 | 1.92e-02 | 1.00e+00 |
|  | 19 | 2428350 | 2428677 | PDFSTATSENSEMBLUCSC | 328 | 4 | 19654 | gene;tss;island | 0.68198 | 0.90551 | Not queried | 2.16 | 2.86e-02 | 1.00e+00 |
|  | 7 | 12609907 | 12610833 | PDFSTATSENSEMBLUCSC | 927 | 15 | 38203 | island | 0.04063 | 0.21967 | Not queried | 2.16 | 4.94e-03 | 1.00e+00 |
|  | 22 | 25575246 | 25575559 | PDFSTATSENSEMBLUCSC | 314 | 4 | 26778 | gene;island | 0.32074 | 0.71532 | Not queried | 2.16 | 2.00e-01 | 1.00e+00 |
|  | 3 | 82857026 | 82857285 | PDFSTATSENSEMBLUCSC | 260 | 4 | 29253 | island | 0.24359 | 0.67109 | Not queried | 2.16 | 2.00e-01 | 1.00e+00 |
|  | 16 | 56554066 | 56554313 | PDFSTATSENSEMBLUCSC | 248 | 5 | 15214 | tss | 0.33792 | 0.06215 | Not queried | 2.16 | 5.48e-01 | 1.00e+00 |
|  | 8 | 1132712 | 1133105 | PDFSTATSENSEMBLUCSC | 394 | 5 | 39953 | island | 0.86773 | 0.92962 | Not queried | 2.16 | 1.51e-01 | 1.00e+00 |
|  | 4 | 55095282 | 55095529 | PDFSTATSENSEMBLUCSC | 248 | 4 | 29959 | gene | 0.18559 | 0.07300 | Not queried | 2.16 | 3.43e-01 | 1.00e+00 |
|  | 6 | 30510238 | 30510459 | PDFSTATSENSEMBLUCSC | 222 | 4 | 33710 | gene | 0.27357 | 0.53186 | Not queried | 2.16 | 2.00e-01 | 1.00e+00 |
|  | 5 | 1882775 | 1883011 | PDFSTATSENSEMBLUCSC | 237 | 6 | 32585 | island | 0.02183 | 0.14866 | Not queried | 2.16 | 9.31e-02 | 1.00e+00 |
|  | 8 | 145106246 | 145106582 | PDFSTATSENSEMBLUCSC | 337 | 4 | 39444 | gene;island | 0.44378 | 0.80014 | Not queried | 2.16 | 2.86e-02 | 1.00e+00 |
|  | 11 | 111410935 | 111411216 | PDFSTATSENSEMBLUCSC | 282 | 7 | 7666 | tss | 0.35768 | 0.04260 | Not queried | 2.15 | 4.56e-01 | 1.00e+00 |
|  | 4 | 187810446 | 187810783 | PDFSTATSENSEMBLUCSC | 338 | 5 | 31433 | island | 0.74223 | 0.93161 | Not queried | 2.15 | 1.59e-02 | 1.00e+00 |
|  | 16 | 56998544 | 56998712 | PDFSTATSENSEMBLUCSC | 169 | 4 | 14734 | gene | 0.05083 | 0.22490 | Not queried | 2.15 | 2.00e-01 | 1.00e+00 |
|  | 4 | 153859106 | 153859560 | PDFSTATSENSEMBLUCSC | 455 | 5 | 31314 | island | 0.02269 | 0.06773 | Not queried | 2.15 | 2.22e-01 | 1.00e+00 |
|  | 17 | 36734475 | 36734942 | PDFSTATSENSEMBLUCSC | 468 | 5 | 16505 | gene;island | 0.40441 | 0.15092 | Not queried | 2.15 | 1.59e-02 | 1.00e+00 |
|  | 2 | 176964404 | 176964512 | PDFSTATSENSEMBLUCSC | 109 | 5 | 23695 | tss | 0.77328 | 0.38759 | Not queried | 2.15 | 7.94e-03 | 1.00e+00 |
|  | 2 | 176964404 | 176964720 | PDFSTATSENSEMBLUCSC | 317 | 7 | 24855 | island | 0.68476 | 0.34850 | Not queried | 2.15 | 5.83e-04 | 1.00e+00 |
|  | 19 | 1675848 | 1676136 | PDFSTATSENSEMBLUCSC | 289 | 4 | 21200 | island | 0.03652 | 0.15685 | Not queried | 2.15 | 5.71e-02 | 1.00e+00 |
|  | 2 | 219857793 | 219858271 | PDFSTATSENSEMBLUCSC | 479 | 9 | 25062 | island | 0.32221 | 0.04601 | Not queried | 2.15 | 2.76e-03 | 1.00e+00 |
|  | 2 | 97173713 | 97174391 | PDFSTATSENSEMBLUCSC | 679 | 12 | 24487 | island | 0.07870 | 0.24660 | Not queried | 2.15 | 3.32e-02 | 1.00e+00 |
|  | 6 | 30431563 | 30431860 | PDFSTATSENSEMBLUCSC | 298 | 15 | 35620 | island | 0.19471 | 0.48145 | Not queried | 2.15 | 1.18e-05 | 6.05e-02 |
|  | X | 48776349 | 48776812 | PDFSTATSENSEMBLUCSC | 464 | 8 | 41777 | island | 0.10576 | 0.02019 | Not queried | 2.15 | 1.48e-02 | 1.00e+00 |
|  | 7 | 1067951 | 1068244 | PDFSTATSENSEMBLUCSC | 294 | 5 | 36675 | gene;island | 0.02603 | 0.13232 | Not queried | 2.15 | 7.94e-03 | 1.00e+00 |
|  | 10 | 57389676 | 57390047 | PDFSTATSENSEMBLUCSC | 372 | 4 | 5735 | island | 0.54572 | 0.73537 | Not queried | 2.14 | 3.43e-01 | 1.00e+00 |
|  | 6 | 13924355 | 13924708 | PDFSTATSENSEMBLUCSC | 354 | 6 | 34582 | tss;island | 0.14897 | 0.04392 | Not queried | 2.14 | 3.94e-01 | 1.00e+00 |
|  | 7 | 862427 | 862831 | PDFSTATSENSEMBLUCSC | 405 | 6 | 36661 | gene;island | 0.58200 | 0.85408 | Not queried | 2.14 | 1.80e-01 | 1.00e+00 |
|  | 17 | 46670933 | 46671635 | PDFSTATSENSEMBLUCSC | 703 | 13 | 16620 | gene;island | 0.01962 | 0.05704 | Not queried | 2.14 | 4.22e-03 | 1.00e+00 |
|  | 11 | 111154742 | 111155021 | PDFSTATSENSEMBLUCSC | 280 | 6 | 6877 | gene | 0.69602 | 0.89572 | Not queried | 2.14 | 2.40e-01 | 1.00e+00 |
|  | 6 | 31025933 | 31026287 | PDFSTATSENSEMBLUCSC | 355 | 6 | 33786 | gene | 0.16097 | 0.43584 | Not queried | 2.14 | 8.66e-03 | 1.00e+00 |
|  | 10 | 3823758 | 3824016 | PDFSTATSENSEMBLUCSC | 259 | 5 | 4462 | gene;island | 0.67361 | 0.87639 | Not queried | 2.14 | 2.22e-01 | 1.00e+00 |
|  | 11 | 69516939 | 69517471 | PDFSTATSENSEMBLUCSC | 533 | 4 | 6765 | gene;island | 0.21265 | 0.10264 | Not queried | 2.14 | 4.86e-01 | 1.00e+00 |
|  | 11 | 33680220 | 33680427 | PDFSTATSENSEMBLUCSC | 208 | 4 | 6558 | gene;island | 0.48710 | 0.82393 | Not queried | 2.14 | 2.86e-02 | 1.00e+00 |
|  | 3 | 40518435 | 40518798 | PDFSTATSENSEMBLUCSC | 364 | 6 | 28962 | island | 0.05721 | 0.11746 | Not queried | 2.14 | 2.40e-01 | 1.00e+00 |
|  | 14 | 71107931 | 71108199 | PDFSTATSENSEMBLUCSC | 269 | 4 | 12460 | tss;island | 0.03151 | 0.13433 | Not queried | 2.14 | 5.71e-02 | 1.00e+00 |
|  | 12 | 113592319 | 113592619 | PDFSTATSENSEMBLUCSC | 301 | 4 | 9295 | gene;island | 0.08009 | 0.29299 | Not queried | 2.14 | 2.86e-02 | 1.00e+00 |
|  | 11 | 74022348 | 74023050 | PDFSTATSENSEMBLUCSC | 703 | 11 | 8626 | island | 0.25903 | 0.12362 | Not queried | 2.14 | 1.93e-01 | 1.00e+00 |
|  | 11 | 43963820 | 43964150 | PDFSTATSENSEMBLUCSC | 331 | 7 | 8198 | island | 0.01406 | 0.01220 | Not queried | 2.14 | 3.18e-01 | 1.00e+00 |
|  | 2 | 71680559 | 71680761 | PDFSTATSENSEMBLUCSC | 203 | 5 | 24361 | island | 0.03406 | 0.14632 | Not queried | 2.13 | 7.94e-03 | 1.00e+00 |
|  | 11 | 67183148 | 67183616 | PDFSTATSENSEMBLUCSC | 469 | 5 | 6735 | gene | 0.13219 | 0.40079 | Not queried | 2.13 | 2.22e-01 | 1.00e+00 |
|  | 6 | 31590513 | 31590870 | PDFSTATSENSEMBLUCSC | 358 | 10 | 33847 | gene;island | 0.58408 | 0.83327 | Not queried | 2.13 | 3.55e-02 | 1.00e+00 |
|  | X | 67867130 | 67867448 | PDFSTATSENSEMBLUCSC | 319 | 8 | 41357 | tss | 0.68070 | 0.20895 | Not queried | 2.13 | 1.05e-01 | 1.00e+00 |
|  | 7 | 45961772 | 45962236 | PDFSTATSENSEMBLUCSC | 465 | 13 | 37523 | tss;island | 0.02801 | 0.16001 | Not queried | 2.13 | 2.95e-02 | 1.00e+00 |
|  | 19 | 9473696 | 9473880 | PDFSTATSENSEMBLUCSC | 185 | 4 | 19764 | gene | 0.85052 | 0.58930 | Not queried | 2.13 | 2.86e-02 | 1.00e+00 |
|  | 10 | 1256339 | 1256487 | PDFSTATSENSEMBLUCSC | 149 | 4 | 5474 | island | 0.42279 | 0.76473 | Not queried | 2.13 | 2.86e-02 | 1.00e+00 |
|  | 1 | 60392140 | 60392392 | PDFSTATSENSEMBLUCSC | 253 | 4 | 559 | gene | 0.19927 | 0.05900 | Not queried | 2.13 | 2.86e-02 | 1.00e+00 |
|  | X | 17673726 | 17674041 | PDFSTATSENSEMBLUCSC | 316 | 4 | 40944 | gene;island | 0.09540 | 0.28673 | Not queried | 2.13 | 2.86e-02 | 1.00e+00 |
|  | 13 | 113698408 | 113698688 | PDFSTATSENSEMBLUCSC | 281 | 6 | 11242 | gene;island | 0.68925 | 0.88927 | Not queried | 2.13 | 2.16e-03 | 1.00e+00 |
|  | 11 | 66104018 | 66104174 | PDFSTATSENSEMBLUCSC | 157 | 4 | 7444 | tss | 0.47368 | 0.79051 | Not queried | 2.13 | 1.14e-01 | 1.00e+00 |
|  | 22 | 19949873 | 19950166 | PDFSTATSENSEMBLUCSC | 294 | 7 | 26754 | gene | 0.04528 | 0.28433 | Not queried | 2.13 | 2.33e-03 | 1.00e+00 |
|  | 6 | 27805693 | 27806081 | PDFSTATSENSEMBLUCSC | 389 | 5 | 33557 | gene | 0.02440 | 0.13852 | Not queried | 2.13 | 5.56e-02 | 1.00e+00 |
|  | 6 | 32782617 | 32783046 | PDFSTATSENSEMBLUCSC | 430 | 5 | 34068 | gene | 0.71561 | 0.46312 | Not queried | 2.13 | 1.59e-02 | 1.00e+00 |
|  | 6 | 28493019 | 28493664 | PDFSTATSENSEMBLUCSC | 646 | 9 | 34679 | tss | 0.90006 | 0.65521 | Not queried | 2.13 | 1.85e-03 | 1.00e+00 |
|  | 17 | 74381133 | 74381280 | PDFSTATSENSEMBLUCSC | 148 | 4 | 17644 | tss | 0.05226 | 0.13317 | Not queried | 2.13 | 1.14e-01 | 1.00e+00 |
|  | 19 | 3029503 | 3029785 | PDFSTATSENSEMBLUCSC | 283 | 4 | 19667 | gene;tss;island | 0.59936 | 0.26006 | Not queried | 2.13 | 3.43e-01 | 1.00e+00 |
|  | 2 | 201980504 | 201980867 | PDFSTATSENSEMBLUCSC | 364 | 7 | 23776 | tss | 0.48635 | 0.28557 | Not queried | 2.13 | 2.59e-01 | 1.00e+00 |
|  | 17 | 41909601 | 41909716 | PDFSTATSENSEMBLUCSC | 116 | 4 | 16564 | gene;island | 0.75395 | 0.41459 | Not queried | 2.13 | 2.00e-01 | 1.00e+00 |
|  | 17 | 80373129 | 80373482 | PDFSTATSENSEMBLUCSC | 354 | 5 | 16913 | gene;island | 0.57673 | 0.88976 | Not queried | 2.13 | 2.22e-01 | 1.00e+00 |
|  | 8 | 133573739 | 133574073 | PDFSTATSENSEMBLUCSC | 335 | 5 | 39855 | tss | 0.82284 | 0.51552 | Not queried | 2.13 | 4.21e-01 | 1.00e+00 |
|  | 6 | 29635110 | 29635692 | PDFSTATSENSEMBLUCSC | 583 | 8 | 33623 | gene | 0.27595 | 0.11953 | Not queried | 2.13 | 1.95e-01 | 1.00e+00 |
|  | 19 | 52873173 | 52873624 | PDFSTATSENSEMBLUCSC | 452 | 4 | 20157 | gene | 0.02498 | 0.13231 | Not queried | 2.13 | 5.71e-02 | 1.00e+00 |
|  | 3 | 129982835 | 129983089 | PDFSTATSENSEMBLUCSC | 255 | 4 | 27904 | gene;island | 0.34332 | 0.71023 | Not queried | 2.12 | 2.86e-02 | 1.00e+00 |
|  | 11 | 67417958 | 67418113 | PDFSTATSENSEMBLUCSC | 156 | 4 | 6744 | gene | 0.45322 | 0.80418 | Not queried | 2.12 | 5.71e-02 | 1.00e+00 |
|  | 14 | 37124018 | 37124434 | PDFSTATSENSEMBLUCSC | 417 | 5 | 12762 | island | 0.47932 | 0.84939 | Not queried | 2.12 | 1.59e-02 | 1.00e+00 |
|  | 11 | 115372874 | 115373119 | PDFSTATSENSEMBLUCSC | 246 | 4 | 6892 | gene;island | 0.20192 | 0.03273 | Not queried | 2.12 | 5.71e-02 | 1.00e+00 |
|  | 4 | 1537622 | 1537799 | PDFSTATSENSEMBLUCSC | 178 | 4 | 30741 | island | 0.67096 | 0.92718 | Not queried | 2.12 | 5.71e-02 | 1.00e+00 |
|  | 6 | 29394438 | 29395425 | PDFSTATSENSEMBLUCSC | 988 | 12 | 33592 | gene | 0.50678 | 0.75441 | Not queried | 2.12 | 2.42e-02 | 1.00e+00 |
|  | 20 | 30073209 | 30073576 | PDFSTATSENSEMBLUCSC | 368 | 6 | 25621 | tss;island | 0.16373 | 0.45247 | Not queried | 2.12 | 4.11e-02 | 1.00e+00 |
|  | 8 | 101224915 | 101225361 | PDFSTATSENSEMBLUCSC | 447 | 6 | 39315 | gene;island | 0.00766 | 0.03993 | Not queried | 2.12 | 1.52e-02 | 1.00e+00 |
|  | 1 | 15271830 | 15272326 | PDFSTATSENSEMBLUCSC | 497 | 6 | 240 | gene;tss | 0.40541 | 0.83824 | Not queried | 2.12 | 2.60e-02 | 1.00e+00 |
|  | 6 | 32135715 | 32135803 | PDFSTATSENSEMBLUCSC | 89 | 5 | 34003 | gene;island | 0.45457 | 0.79714 | Not queried | 2.12 | 7.94e-03 | 1.00e+00 |
|  | 2 | 145277596 | 145277852 | PDFSTATSENSEMBLUCSC | 257 | 9 | 22854 | gene;island | 0.13230 | 0.02172 | Not queried | 2.12 | 6.25e-02 | 1.00e+00 |
|  | 6 | 169013150 | 169013766 | PDFSTATSENSEMBLUCSC | 617 | 6 | 34498 | gene | 0.96191 | 0.87470 | Not queried | 2.12 | 9.31e-02 | 1.00e+00 |
|  | 6 | 166571782 | 166572045 | PDFSTATSENSEMBLUCSC | 264 | 5 | 34478 | gene;island | 0.94011 | 0.82917 | Not queried | 2.12 | 2.22e-01 | 1.00e+00 |
|  | 6 | 31711701 | 31712195 | PDFSTATSENSEMBLUCSC | 495 | 7 | 33887 | gene;island | 0.57988 | 0.88962 | Not queried | 2.12 | 2.62e-02 | 1.00e+00 |
|  | X | 48754200 | 48754642 | PDFSTATSENSEMBLUCSC | 443 | 5 | 40987 | gene;tss;island | 0.10993 | 0.02888 | Not queried | 2.12 | 3.17e-02 | 1.00e+00 |
|  | 3 | 113160071 | 113160821 | PDFSTATSENSEMBLUCSC | 751 | 10 | 29321 | island | 0.71738 | 0.93257 | Not queried | 2.12 | 2.88e-03 | 1.00e+00 |
|  | 2 | 3699195 | 3699353 | PDFSTATSENSEMBLUCSC | 159 | 4 | 24001 | island | 0.37881 | 0.11782 | Not queried | 2.12 | 2.86e-02 | 1.00e+00 |
|  | 5 | 158531132 | 158531647 | PDFSTATSENSEMBLUCSC | 516 | 5 | 33287 | island | 0.03048 | 0.18347 | Not queried | 2.12 | 1.51e-01 | 1.00e+00 |
|  | 11 | 116706372 | 116706626 | PDFSTATSENSEMBLUCSC | 255 | 4 | 8814 | island | 0.32342 | 0.10255 | Not queried | 2.11 | 1.14e-01 | 1.00e+00 |
|  | 1 | 149871404 | 149871658 | PDFSTATSENSEMBLUCSC | 255 | 4 | 732 | gene;island | 0.25077 | 0.56804 | Not queried | 2.11 | 2.86e-02 | 1.00e+00 |
|  | 19 | 1313407 | 1313642 | PDFSTATSENSEMBLUCSC | 236 | 4 | 21177 | island | 0.43487 | 0.76752 | Not queried | 2.11 | 1.14e-01 | 1.00e+00 |
|  | 12 | 54942782 | 54943102 | PDFSTATSENSEMBLUCSC | 321 | 5 | 9740 | tss | 0.57264 | 0.23994 | Not queried | 2.11 | 5.56e-02 | 1.00e+00 |
|  | 22 | 19949873 | 19950040 | PDFSTATSENSEMBLUCSC | 168 | 6 | 26929 | tss | 0.04697 | 0.23148 | Not queried | 2.11 | 8.66e-03 | 1.00e+00 |
|  | 20 | 42143080 | 42143502 | PDFSTATSENSEMBLUCSC | 423 | 7 | 25383 | gene | 0.00746 | 0.06478 | Not queried | 2.11 | 5.30e-02 | 1.00e+00 |
|  | 17 | 6659164 | 6659562 | PDFSTATSENSEMBLUCSC | 399 | 7 | 16341 | gene | 0.37370 | 0.64329 | Not queried | 2.11 | 5.83e-04 | 1.00e+00 |
|  | 14 | 51561293 | 51561618 | PDFSTATSENSEMBLUCSC | 326 | 4 | 12051 | gene;island | 0.05342 | 0.12374 | Not queried | 2.11 | 5.71e-02 | 1.00e+00 |
|  | 14 | 61788810 | 61788914 | PDFSTATSENSEMBLUCSC | 105 | 4 | 12078 | gene;island | 0.03400 | 0.20397 | Not queried | 2.11 | 1.14e-01 | 1.00e+00 |
|  | 1 | 156338639 | 156338826 | PDFSTATSENSEMBLUCSC | 188 | 4 | 2011 | tss | 0.76806 | 0.51100 | Not queried | 2.10 | 2.86e-02 | 1.00e+00 |
|  | 19 | 2085286 | 2085483 | PDFSTATSENSEMBLUCSC | 198 | 4 | 19643 | gene | 0.85002 | 0.96121 | Not queried | 2.10 | 2.86e-02 | 1.00e+00 |
|  | 8 | 124214953 | 124215152 | PDFSTATSENSEMBLUCSC | 200 | 4 | 39355 | gene | 0.42943 | 0.64696 | Not queried | 2.10 | 4.86e-01 | 1.00e+00 |
|  | 7 | 94953956 | 94954202 | PDFSTATSENSEMBLUCSC | 247 | 4 | 37660 | tss | 0.58308 | 0.91183 | Not queried | 2.10 | 5.71e-02 | 1.00e+00 |
|  | 6 | 29855110 | 29855636 | PDFSTATSENSEMBLUCSC | 527 | 16 | 35577 | island | 0.66010 | 0.29621 | Not queried | 2.10 | 6.53e-04 | 1.00e+00 |
|  | 19 | 40724308 | 40724388 | PDFSTATSENSEMBLUCSC | 81 | 5 | 20747 | tss;island | 0.02452 | 0.14049 | Not queried | 2.10 | 5.56e-02 | 1.00e+00 |
|  | 19 | 57352269 | 57352807 | PDFSTATSENSEMBLUCSC | 539 | 12 | 20205 | gene | 0.11638 | 0.05014 | Not queried | 2.10 | 1.60e-01 | 1.00e+00 |
|  | 19 | 15530606 | 15530870 | PDFSTATSENSEMBLUCSC | 265 | 4 | 20525 | tss;island | 0.28215 | 0.51332 | Not queried | 2.10 | 2.00e-01 | 1.00e+00 |
|  | 1 | 228337181 | 228337503 | PDFSTATSENSEMBLUCSC | 323 | 5 | 2296 | tss | 0.39513 | 0.60146 | Not queried | 2.10 | 7.94e-03 | 1.00e+00 |
|  | 19 | 15311886 | 15312233 | PDFSTATSENSEMBLUCSC | 348 | 5 | 20520 | tss;island | 0.15466 | 0.06644 | Not queried | 2.10 | 1.51e-01 | 1.00e+00 |
|  | 8 | 25909171 | 25909599 | PDFSTATSENSEMBLUCSC | 429 | 6 | 40107 | island | 0.43021 | 0.10727 | Not queried | 2.10 | 8.66e-03 | 1.00e+00 |
|  | 12 | 123636480 | 123636717 | PDFSTATSENSEMBLUCSC | 238 | 4 | 10915 | island | 0.75446 | 0.94045 | Not queried | 2.10 | 2.00e-01 | 1.00e+00 |
|  | 1 | 235805403 | 235805770 | PDFSTATSENSEMBLUCSC | 368 | 4 | 1088 | gene;island | 0.43431 | 0.75094 | Not queried | 2.10 | 2.86e-02 | 1.00e+00 |
|  | 1 | 6515580 | 6515748 | PDFSTATSENSEMBLUCSC | 169 | 4 | 172 | gene;island | 0.51668 | 0.84197 | Not queried | 2.09 | 2.86e-02 | 1.00e+00 |
|  | 7 | 158250129 | 158250524 | PDFSTATSENSEMBLUCSC | 396 | 4 | 37309 | gene;island | 0.70262 | 0.89599 | Not queried | 2.09 | 2.86e-02 | 1.00e+00 |
|  | 19 | 17487776 | 17488116 | PDFSTATSENSEMBLUCSC | 341 | 5 | 19882 | gene | 0.77212 | 0.92530 | Not queried | 2.09 | 1.59e-02 | 1.00e+00 |
|  | 10 | 120355201 | 120355427 | PDFSTATSENSEMBLUCSC | 227 | 7 | 5378 | tss | 0.20446 | 0.55113 | Not queried | 2.09 | 7.28e-02 | 1.00e+00 |
|  | 8 | 144790317 | 144790410 | PDFSTATSENSEMBLUCSC | 94 | 4 | 39894 | tss | 0.68019 | 0.84893 | Not queried | 2.09 | 2.86e-02 | 1.00e+00 |
|  | 1 | 3162066 | 3162173 | PDFSTATSENSEMBLUCSC | 108 | 4 | 111 | gene;island | 0.52873 | 0.88372 | Not queried | 2.09 | 5.71e-02 | 1.00e+00 |
|  | 12 | 56694168 | 56694667 | PDFSTATSENSEMBLUCSC | 500 | 10 | 10506 | island | 0.02077 | 0.06639 | Not queried | 2.09 | 5.24e-02 | 1.00e+00 |
|  | 14 | 21270850 | 21271355 | PDFSTATSENSEMBLUCSC | 506 | 6 | 12250 | tss | 0.83126 | 0.93758 | Not queried | 2.09 | 1.52e-02 | 1.00e+00 |
|  | 17 | 46799479 | 46800028 | PDFSTATSENSEMBLUCSC | 550 | 9 | 17436 | tss;island | 0.89281 | 0.60390 | Not queried | 2.09 | 1.85e-03 | 1.00e+00 |
|  | 11 | 120080945 | 120081259 | PDFSTATSENSEMBLUCSC | 315 | 4 | 7737 | tss;island | 0.17331 | 0.04265 | Not queried | 2.09 | 1.14e-01 | 1.00e+00 |
|  | 11 | 108092087 | 108092453 | PDFSTATSENSEMBLUCSC | 367 | 10 | 6866 | gene;tss;island | 0.04325 | 0.01879 | Not queried | 2.09 | 1.90e-01 | 1.00e+00 |
|  | 5 | 140740387 | 140740607 | PDFSTATSENSEMBLUCSC | 221 | 4 | 31765 | gene;island | 0.56006 | 0.87681 | Not queried | 2.09 | 2.00e-01 | 1.00e+00 |
|  | 4 | 141264393 | 141264602 | PDFSTATSENSEMBLUCSC | 210 | 4 | 30556 | tss | 0.79424 | 0.94131 | Not queried | 2.09 | 2.86e-02 | 1.00e+00 |
|  | 14 | 102680187 | 102680518 | PDFSTATSENSEMBLUCSC | 332 | 4 | 12200 | gene;island | 0.56488 | 0.82494 | Not queried | 2.09 | 2.86e-02 | 1.00e+00 |
|  | 8 | 41167660 | 41168481 | PDFSTATSENSEMBLUCSC | 822 | 12 | 39604 | tss;island | 0.78803 | 0.42533 | Not queried | 2.09 | 2.74e-04 | 1.00e+00 |
|  | 1 | 41156730 | 41157266 | PDFSTATSENSEMBLUCSC | 537 | 8 | 456 | gene;tss;island | 0.14018 | 0.02704 | Not queried | 2.09 | 1.04e-02 | 1.00e+00 |
|  | 7 | 96643454 | 96643821 | PDFSTATSENSEMBLUCSC | 368 | 5 | 37669 | tss;island | 0.07947 | 0.40436 | Not queried | 2.09 | 2.22e-01 | 1.00e+00 |
|  | 14 | 75330608 | 75330798 | PDFSTATSENSEMBLUCSC | 191 | 4 | 12483 | tss | 0.76670 | 0.93318 | Not queried | 2.09 | 2.86e-02 | 1.00e+00 |
|  | 11 | 3177622 | 3178322 | PDFSTATSENSEMBLUCSC | 701 | 6 | 6458 | gene;island | 0.79037 | 0.93937 | Not queried | 2.08 | 8.66e-03 | 1.00e+00 |
|  | 10 | 63213339 | 63213510 | PDFSTATSENSEMBLUCSC | 172 | 5 | 5080 | tss | 0.04116 | 0.22307 | Not queried | 2.08 | 9.52e-02 | 1.00e+00 |
|  | 17 | 74381133 | 74381380 | PDFSTATSENSEMBLUCSC | 248 | 5 | 16750 | gene;island | 0.06221 | 0.18253 | Not queried | 2.08 | 9.52e-02 | 1.00e+00 |
|  | 13 | 25338113 | 25338289 | PDFSTATSENSEMBLUCSC | 177 | 6 | 11319 | tss | 0.78420 | 0.95567 | Not queried | 2.08 | 6.49e-02 | 1.00e+00 |
|  | 3 | 9258385 | 9258573 | PDFSTATSENSEMBLUCSC | 189 | 5 | 27609 | gene | 0.32527 | 0.90139 | Not queried | 2.08 | 3.17e-02 | 1.00e+00 |
|  | X | 56258900 | 56259373 | PDFSTATSENSEMBLUCSC | 474 | 6 | 41003 | gene | 0.08842 | 0.02197 | Not queried | 2.08 | 9.31e-02 | 1.00e+00 |
|  | 7 | 5111488 | 5111669 | PDFSTATSENSEMBLUCSC | 182 | 8 | 37355 | tss;island | 0.05383 | 0.15506 | Not queried | 2.08 | 2.07e-02 | 1.00e+00 |
|  | 22 | 39640959 | 39641154 | PDFSTATSENSEMBLUCSC | 196 | 6 | 27108 | tss | 0.05001 | 0.20745 | Not queried | 2.08 | 8.66e-03 | 1.00e+00 |
|  | 18 | 71958904 | 71959795 | PDFSTATSENSEMBLUCSC | 892 | 12 | 19534 | island | 0.10561 | 0.01793 | Not queried | 2.08 | 4.51e-03 | 1.00e+00 |
|  | 15 | 75470777 | 75471218 | PDFSTATSENSEMBLUCSC | 442 | 6 | 14297 | island | 0.62156 | 0.29435 | Not queried | 2.08 | 4.33e-03 | 1.00e+00 |
|  | 2 | 37571677 | 37571901 | PDFSTATSENSEMBLUCSC | 225 | 6 | 24185 | island | 0.03016 | 0.07086 | Not queried | 2.08 | 2.60e-02 | 1.00e+00 |
|  | 15 | 95869918 | 95870475 | PDFSTATSENSEMBLUCSC | 558 | 9 | 14456 | island | 0.04171 | 0.18051 | Not queried | 2.08 | 2.76e-03 | 1.00e+00 |
|  | 7 | 157366933 | 157367159 | PDFSTATSENSEMBLUCSC | 227 | 5 | 37265 | gene;island | 0.80369 | 0.94367 | Not queried | 2.08 | 1.59e-02 | 1.00e+00 |
|  | 19 | 47287778 | 47288263 | PDFSTATSENSEMBLUCSC | 486 | 7 | 20078 | gene;island | 0.28005 | 0.03852 | Not queried | 2.08 | 1.75e-02 | 1.00e+00 |
|  | Y | 22917782 | 22917942 | PDFSTATSENSEMBLUCSC | 161 | 5 | 42174 | tss | 0.81070 | 0.54487 | Not queried | 2.08 | 7.94e-03 | 1.00e+00 |
|  | 15 | 74421739 | 74421935 | PDFSTATSENSEMBLUCSC | 197 | 7 | 13387 | gene | 0.08008 | 0.02633 | Not queried | 2.08 | 3.79e-02 | 1.00e+00 |
|  | 7 | 6655711 | 6655973 | PDFSTATSENSEMBLUCSC | 263 | 4 | 36824 | gene;island | 0.38195 | 0.13045 | Not queried | 2.08 | 3.43e-01 | 1.00e+00 |
|  | X | 34961467 | 34961658 | PDFSTATSENSEMBLUCSC | 192 | 4 | 40961 | gene;island | 0.52421 | 0.78837 | Not queried | 2.08 | 2.00e-01 | 1.00e+00 |
|  | 8 | 145162300 | 145162592 | PDFSTATSENSEMBLUCSC | 293 | 4 | 39910 | tss;island | 0.64257 | 0.88754 | Not queried | 2.08 | 2.00e-01 | 1.00e+00 |
|  | 11 | 130298544 | 130298880 | PDFSTATSENSEMBLUCSC | 337 | 8 | 7797 | tss | 0.24375 | 0.04972 | Not queried | 2.08 | 6.50e-02 | 1.00e+00 |
|  | 4 | 41880280 | 41880747 | PDFSTATSENSEMBLUCSC | 468 | 6 | 30956 | island | 0.85279 | 0.56694 | Not queried | 2.08 | 4.33e-03 | 1.00e+00 |
|  | 17 | 14206774 | 14207036 | PDFSTATSENSEMBLUCSC | 263 | 7 | 16414 | gene;tss;island | 0.01389 | 0.05333 | Not queried | 2.07 | 1.75e-02 | 1.00e+00 |
|  | 8 | 1051510 | 1051814 | PDFSTATSENSEMBLUCSC | 305 | 5 | 39947 | island | 0.74865 | 0.89037 | Not queried | 2.07 | 1.51e-01 | 1.00e+00 |
|  | 15 | 96868857 | 96869132 | PDFSTATSENSEMBLUCSC | 276 | 7 | 13889 | tss | 0.02623 | 0.13997 | Not queried | 2.07 | 1.75e-02 | 1.00e+00 |
|  | 8 | 110552297 | 110552896 | PDFSTATSENSEMBLUCSC | 600 | 6 | 39335 | gene;island | 0.12590 | 0.01550 | Not queried | 2.07 | 1.32e-01 | 1.00e+00 |
|  | 11 | 130416857 | 130417168 | PDFSTATSENSEMBLUCSC | 312 | 5 | 8939 | island | 0.92275 | 0.73954 | Not queried | 2.07 | 3.10e-01 | 1.00e+00 |
|  | 12 | 81110706 | 81111012 | PDFSTATSENSEMBLUCSC | 307 | 5 | 10621 | island | 0.52696 | 0.76735 | Not queried | 2.07 | 7.94e-03 | 1.00e+00 |
|  | 10 | 99080594 | 99081016 | PDFSTATSENSEMBLUCSC | 423 | 4 | 4679 | gene;island | 0.27400 | 0.08659 | Not queried | 2.07 | 3.43e-01 | 1.00e+00 |
|  | 12 | 132859441 | 132859951 | PDFSTATSENSEMBLUCSC | 511 | 5 | 9417 | gene;island | 0.67001 | 0.83940 | Not queried | 2.07 | 9.52e-02 | 1.00e+00 |
|  | X | 27825843 | 27826007 | PDFSTATSENSEMBLUCSC | 165 | 5 | 41240 | tss | 0.75923 | 0.89996 | Not queried | 2.07 | 5.56e-02 | 1.00e+00 |
|  | 5 | 180646608 | 180646976 | PDFSTATSENSEMBLUCSC | 369 | 4 | 33457 | island | 0.73248 | 0.39482 | Not queried | 2.07 | 2.86e-02 | 1.00e+00 |
|  | 12 | 48577720 | 48578223 | PDFSTATSENSEMBLUCSC | 504 | 4 | 9086 | gene;island | 0.03707 | 0.13868 | Not queried | 2.07 | 2.00e-01 | 1.00e+00 |
|  | 17 | 7121848 | 7122261 | PDFSTATSENSEMBLUCSC | 414 | 4 | 16349 | gene;tss;island | 0.23995 | 0.62371 | Not queried | 2.07 | 5.71e-02 | 1.00e+00 |
|  | 6 | 55039232 | 55039382 | PDFSTATSENSEMBLUCSC | 151 | 4 | 34309 | gene;island | 0.07159 | 0.26967 | Not queried | 2.07 | 2.00e-01 | 1.00e+00 |
|  | 8 | 41504078 | 41504331 | PDFSTATSENSEMBLUCSC | 254 | 4 | 39225 | gene;island | 0.01497 | 0.05728 | Not queried | 2.07 | 2.86e-02 | 1.00e+00 |
|  | 17 | 21279561 | 21279874 | PDFSTATSENSEMBLUCSC | 314 | 6 | 18085 | island | 0.21345 | 0.05985 | Not queried | 2.07 | 1.52e-02 | 1.00e+00 |
|  | 4 | 54965829 | 54966247 | PDFSTATSENSEMBLUCSC | 419 | 6 | 30999 | island | 0.07978 | 0.05230 | Not queried | 2.07 | 2.40e-01 | 1.00e+00 |
|  | 6 | 72130209 | 72130799 | PDFSTATSENSEMBLUCSC | 591 | 12 | 36181 | island | 0.02322 | 0.07069 | Not queried | 2.07 | 1.00e-02 | 1.00e+00 |
|  | 17 | 74868561 | 74868725 | PDFSTATSENSEMBLUCSC | 165 | 4 | 16762 | gene;tss;island | 0.33492 | 0.64542 | Not queried | 2.07 | 2.86e-02 | 1.00e+00 |
|  | 16 | 47007740 | 47007832 | PDFSTATSENSEMBLUCSC | 93 | 4 | 15191 | tss;island | 0.14005 | 0.02469 | Not queried | 2.07 | 5.71e-02 | 1.00e+00 |
|  | 17 | 46656543 | 46656690 | PDFSTATSENSEMBLUCSC | 148 | 4 | 17427 | tss;island | 0.14780 | 0.43123 | Not queried | 2.07 | 2.00e-01 | 1.00e+00 |
|  | 3 | 126006749 | 126006903 | PDFSTATSENSEMBLUCSC | 155 | 4 | 29385 | island | 0.12654 | 0.04358 | Not queried | 2.07 | 4.86e-01 | 1.00e+00 |
|  | 17 | 46827458 | 46827626 | PDFSTATSENSEMBLUCSC | 169 | 4 | 18473 | island | 0.75688 | 0.36427 | Not queried | 2.07 | 5.71e-02 | 1.00e+00 |
|  | 12 | 113013048 | 113013494 | PDFSTATSENSEMBLUCSC | 447 | 5 | 10794 | island | 0.53562 | 0.78704 | Not queried | 2.07 | 7.94e-03 | 1.00e+00 |
|  | 7 | 55516724 | 55517132 | PDFSTATSENSEMBLUCSC | 409 | 5 | 38441 | island | 0.77221 | 0.93416 | Not queried | 2.07 | 7.94e-03 | 1.00e+00 |
|  | 19 | 17877419 | 17877846 | PDFSTATSENSEMBLUCSC | 428 | 6 | 19886 | gene;island | 0.02083 | 0.13358 | Not queried | 2.07 | 1.80e-01 | 1.00e+00 |
|  | 10 | 134853084 | 134853344 | PDFSTATSENSEMBLUCSC | 261 | 4 | 6309 | island | 0.56059 | 0.84588 | Not queried | 2.06 | 2.00e-01 | 1.00e+00 |
|  | 10 | 79793353 | 79793501 | PDFSTATSENSEMBLUCSC | 149 | 7 | 5162 | tss | 0.02320 | 0.06280 | Not queried | 2.06 | 1.65e-01 | 1.00e+00 |
|  | 6 | 29631227 | 29631447 | PDFSTATSENSEMBLUCSC | 221 | 4 | 33622 | gene | 0.53673 | 0.67756 | Not queried | 2.06 | 3.43e-01 | 1.00e+00 |
|  | 8 | 9791214 | 9791557 | PDFSTATSENSEMBLUCSC | 344 | 4 | 40003 | island | 0.03902 | 0.16937 | Not queried | 2.06 | 2.86e-02 | 1.00e+00 |
|  | 3 | 130368058 | 130368395 | PDFSTATSENSEMBLUCSC | 338 | 6 | 27905 | gene | 0.50772 | 0.78500 | Not queried | 2.06 | 1.32e-01 | 1.00e+00 |
|  | 3 | 10547373 | 10547584 | PDFSTATSENSEMBLUCSC | 212 | 4 | 28108 | tss | 0.22541 | 0.54632 | Not queried | 2.06 | 3.43e-01 | 1.00e+00 |
|  | 12 | 117319785 | 117320316 | PDFSTATSENSEMBLUCSC | 532 | 7 | 10016 | tss;island | 0.16325 | 0.03926 | Not queried | 2.06 | 9.73e-02 | 1.00e+00 |
|  | 8 | 39171866 | 39172120 | PDFSTATSENSEMBLUCSC | 255 | 7 | 39599 | tss | 0.06592 | 0.25567 | Not queried | 2.06 | 1.75e-02 | 1.00e+00 |
|  | 2 | 219858132 | 219858271 | PDFSTATSENSEMBLUCSC | 140 | 6 | 23849 | tss | 0.32158 | 0.07222 | Not queried | 2.06 | 2.16e-03 | 1.00e+00 |
|  | 5 | 180288429 | 180288995 | PDFSTATSENSEMBLUCSC | 567 | 5 | 32504 | tss;island | 0.24169 | 0.02355 | Not queried | 2.06 | 8.41e-01 | 1.00e+00 |
|  | 1 | 167682516 | 167683013 | PDFSTATSENSEMBLUCSC | 498 | 6 | 3936 | island | 0.05915 | 0.01577 | Not queried | 2.05 | 8.66e-03 | 1.00e+00 |
|  | 3 | 134083080 | 134083286 | PDFSTATSENSEMBLUCSC | 207 | 4 | 27912 | gene;island | 0.04617 | 0.17794 | Not queried | 2.05 | 5.71e-02 | 1.00e+00 |
|  | 2 | 37571677 | 37571732 | PDFSTATSENSEMBLUCSC | 56 | 5 | 23268 | tss | 0.02092 | 0.06464 | Not queried | 2.05 | 1.59e-02 | 1.00e+00 |
|  | 1 | 25257505 | 25258264 | PDFSTATSENSEMBLUCSC | 760 | 20 | 1361 | tss | 0.35193 | 0.08869 | RUNX3 (margin: RUNX3) | 2.05 | 8.34e-08 | 4.29e-04 |
|  | 3 | 11178593 | 11178758 | PDFSTATSENSEMBLUCSC | 166 | 5 | 28112 | tss | 0.36088 | 0.62963 | Not queried | 2.05 | 3.17e-02 | 1.00e+00 |
|  | 1 | 33648648 | 33648819 | PDFSTATSENSEMBLUCSC | 172 | 4 | 1467 | tss;island | 0.44738 | 0.73981 | Not queried | 2.05 | 1.14e-01 | 1.00e+00 |
|  | 6 | 32179862 | 32180305 | PDFSTATSENSEMBLUCSC | 444 | 6 | 34030 | gene | 0.82438 | 0.54202 | Not queried | 2.05 | 1.32e-01 | 1.00e+00 |
|  | 11 | 537558 | 537737 | PDFSTATSENSEMBLUCSC | 180 | 4 | 6360 | gene | 0.66775 | 0.24946 | Not queried | 2.05 | 1.14e-01 | 1.00e+00 |
|  | 1 | 45792557 | 45792733 | PDFSTATSENSEMBLUCSC | 177 | 5 | 500 | gene | 0.62127 | 0.85807 | Not queried | 2.05 | 1.59e-02 | 1.00e+00 |
|  | 6 | 30881112 | 30882384 | PDFSTATSENSEMBLUCSC | 1273 | 39 | 35678 | island | 0.06502 | 0.46325 | Not queried | 2.05 | 1.01e-03 | 1.00e+00 |
|  | X | 139590116 | 139590479 | PDFSTATSENSEMBLUCSC | 364 | 4 | 42078 | island | 0.09268 | 0.03357 | Not queried | 2.05 | 4.86e-01 | 1.00e+00 |
|  | 2 | 242927418 | 242927773 | PDFSTATSENSEMBLUCSC | 356 | 4 | 25280 | island | 0.35641 | 0.69402 | Not queried | 2.05 | 2.86e-02 | 1.00e+00 |
|  | 3 | 27766009 | 27766382 | PDFSTATSENSEMBLUCSC | 374 | 6 | 28910 | island | 0.49765 | 0.19825 | Not queried | 2.05 | 9.31e-02 | 1.00e+00 |
|  | 11 | 69590282 | 69590580 | PDFSTATSENSEMBLUCSC | 299 | 7 | 7501 | tss | 0.09447 | 0.02927 | Not queried | 2.05 | 9.73e-02 | 1.00e+00 |
|  | 8 | 142517341 | 142517553 | PDFSTATSENSEMBLUCSC | 213 | 5 | 39866 | tss | 0.46696 | 0.68113 | Not queried | 2.05 | 5.56e-02 | 1.00e+00 |
|  | 12 | 40019768 | 40019919 | PDFSTATSENSEMBLUCSC | 152 | 4 | 9613 | tss | 0.63599 | 0.89483 | Not queried | 2.05 | 2.86e-02 | 1.00e+00 |
|  | 22 | 50708924 | 50709221 | PDFSTATSENSEMBLUCSC | 298 | 5 | 27211 | tss;island | 0.02030 | 0.04798 | Not queried | 2.05 | 7.94e-03 | 1.00e+00 |
|  | 11 | 27743258 | 27743580 | PDFSTATSENSEMBLUCSC | 323 | 4 | 6530 | gene | 0.54421 | 0.18694 | Not queried | 2.04 | 3.43e-01 | 1.00e+00 |
|  | 1 | 223888869 | 223889141 | PDFSTATSENSEMBLUCSC | 273 | 4 | 2269 | tss | 0.36176 | 0.70167 | Not queried | 2.04 | 2.86e-02 | 1.00e+00 |
|  | 17 | 10560609 | 10560928 | PDFSTATSENSEMBLUCSC | 320 | 5 | 17101 | tss | 0.60541 | 0.88081 | Not queried | 2.04 | 7.94e-03 | 1.00e+00 |
|  | 17 | 66508512 | 66508605 | PDFSTATSENSEMBLUCSC | 94 | 4 | 16707 | gene;island | 0.00569 | 0.02126 | Not queried | 2.04 | 2.00e-01 | 1.00e+00 |
|  | 17 | 7758397 | 7758854 | PDFSTATSENSEMBLUCSC | 458 | 7 | 16382 | gene | 0.57199 | 0.84617 | Not queried | 2.04 | 5.83e-04 | 1.00e+00 |
|  | X | 43515213 | 43515575 | PDFSTATSENSEMBLUCSC | 363 | 7 | 41730 | island | 0.15943 | 0.04716 | Not queried | 2.04 | 7.28e-02 | 1.00e+00 |
|  | 9 | 139096738 | 139097029 | PDFSTATSENSEMBLUCSC | 292 | 4 | 40909 | island | 0.13621 | 0.41213 | Not queried | 2.04 | 3.43e-01 | 1.00e+00 |
|  | 12 | 7341580 | 7341666 | PDFSTATSENSEMBLUCSC | 87 | 4 | 9522 | tss;island | 0.21787 | 0.03573 | Not queried | 2.04 | 2.00e-01 | 1.00e+00 |
|  | 11 | 64532839 | 64533113 | PDFSTATSENSEMBLUCSC | 275 | 4 | 6676 | gene;island | 0.80212 | 0.93980 | Not queried | 2.04 | 1.14e-01 | 1.00e+00 |
|  | 4 | 3516534 | 3516758 | PDFSTATSENSEMBLUCSC | 225 | 4 | 29865 | gene;island | 0.72943 | 0.90909 | Not queried | 2.04 | 1.14e-01 | 1.00e+00 |
|  | 1 | 34632573 | 34632658 | PDFSTATSENSEMBLUCSC | 86 | 4 | 1473 | tss;island | 0.53407 | 0.22874 | Not queried | 2.04 | 3.43e-01 | 1.00e+00 |
|  | 17 | 40332731 | 40333009 | PDFSTATSENSEMBLUCSC | 279 | 4 | 16542 | gene;island | 0.05328 | 0.24997 | Not queried | 2.04 | 1.14e-01 | 1.00e+00 |
|  | 3 | 148804381 | 148805294 | PDFSTATSENSEMBLUCSC | 914 | 21 | 28612 | tss | 0.94020 | 0.56346 | Not queried | 2.04 | 1.46e-02 | 1.00e+00 |
|  | 4 | 995849 | 996280 | PDFSTATSENSEMBLUCSC | 432 | 5 | 29811 | gene;island | 0.73027 | 0.93271 | Not queried | 2.04 | 1.59e-02 | 1.00e+00 |
|  | 17 | 47113407 | 47113596 | PDFSTATSENSEMBLUCSC | 190 | 4 | 16636 | gene;island | 0.52601 | 0.83991 | Not queried | 2.04 | 1.14e-01 | 1.00e+00 |
|  | 8 | 62200380 | 62200518 | PDFSTATSENSEMBLUCSC | 139 | 4 | 39663 | tss | 0.01809 | 0.06500 | Not queried | 2.03 | 2.86e-02 | 1.00e+00 |
|  | 7 | 37955508 | 37956018 | PDFSTATSENSEMBLUCSC | 511 | 6 | 36906 | gene;island | 0.12457 | 0.39195 | Not queried | 2.03 | 4.11e-02 | 1.00e+00 |
|  | 15 | 69744390 | 69745085 | PDFSTATSENSEMBLUCSC | 696 | 12 | 13736 | tss | 0.03814 | 0.01280 | Not queried | 2.03 | 1.43e-03 | 1.00e+00 |
|  | 19 | 19050971 | 19051482 | PDFSTATSENSEMBLUCSC | 512 | 8 | 19909 | gene;tss;island | 0.27290 | 0.08326 | Not queried | 2.03 | 1.04e-02 | 1.00e+00 |
|  | 7 | 77669697 | 77670191 | PDFSTATSENSEMBLUCSC | 495 | 5 | 37001 | gene;island | 0.07148 | 0.38215 | Not queried | 2.03 | 5.48e-01 | 1.00e+00 |
|  | 12 | 129337674 | 129337910 | PDFSTATSENSEMBLUCSC | 237 | 5 | 10090 | tss;island | 0.79656 | 0.48938 | Not queried | 2.03 | 1.59e-02 | 1.00e+00 |
|  | 7 | 73245439 | 73245752 | PDFSTATSENSEMBLUCSC | 314 | 4 | 36979 | gene;island | 0.54728 | 0.82555 | Not queried | 2.03 | 2.86e-02 | 1.00e+00 |
|  | 16 | 56622951 | 56623215 | PDFSTATSENSEMBLUCSC | 265 | 6 | 15215 | tss | 0.50048 | 0.23544 | Not queried | 2.03 | 3.10e-01 | 1.00e+00 |
|  | 16 | 72042693 | 72042925 | PDFSTATSENSEMBLUCSC | 233 | 4 | 14804 | gene | 0.01438 | 0.02784 | Not queried | 2.03 | 3.43e-01 | 1.00e+00 |
|  | 1 | 6304272 | 6304499 | PDFSTATSENSEMBLUCSC | 228 | 4 | 164 | gene | 0.61521 | 0.20598 | Not queried | 2.03 | 1.14e-01 | 1.00e+00 |
|  | 7 | 149411256 | 149411652 | PDFSTATSENSEMBLUCSC | 397 | 4 | 37906 | tss;island | 0.37216 | 0.13393 | Not queried | 2.03 | 3.43e-01 | 1.00e+00 |
|  | 6 | 32147440 | 32147894 | PDFSTATSENSEMBLUCSC | 455 | 8 | 34012 | gene | 0.63146 | 0.87486 | Not queried | 2.03 | 4.66e-03 | 1.00e+00 |
|  | 15 | 37782885 | 37783172 | PDFSTATSENSEMBLUCSC | 288 | 6 | 13988 | island | 0.38523 | 0.83156 | Not queried | 2.03 | 2.16e-03 | 1.00e+00 |
|  | 14 | 38068553 | 38069206 | PDFSTATSENSEMBLUCSC | 654 | 9 | 12770 | island | 0.03982 | 0.25210 | Not queried | 2.02 | 1.06e-02 | 1.00e+00 |
|  | 3 | 49449349 | 49449964 | PDFSTATSENSEMBLUCSC | 616 | 14 | 28286 | tss;island | 0.01913 | 0.05034 | Not queried | 2.02 | 2.49e-03 | 1.00e+00 |
|  | 4 | 7941764 | 7942031 | PDFSTATSENSEMBLUCSC | 268 | 8 | 30247 | tss;island | 0.10739 | 0.03424 | Not queried | 2.02 | 3.28e-01 | 1.00e+00 |
|  | 6 | 108479070 | 108479584 | PDFSTATSENSEMBLUCSC | 515 | 7 | 36332 | island | 0.01349 | 0.06402 | Not queried | 2.02 | 2.62e-02 | 1.00e+00 |
|  | 12 | 56843287 | 56843596 | PDFSTATSENSEMBLUCSC | 310 | 7 | 9773 | tss | 0.08834 | 0.02913 | Not queried | 2.02 | 3.79e-02 | 1.00e+00 |
|  | 19 | 23941207 | 23941575 | PDFSTATSENSEMBLUCSC | 369 | 5 | 19931 | gene | 0.18208 | 0.04376 | Not queried | 2.02 | 7.94e-03 | 1.00e+00 |
|  | 17 | 72772726 | 72772745 | PDFSTATSENSEMBLUCSC | 20 | 4 | 16725 | gene | 0.01120 | 0.02521 | Not queried | 2.02 | 4.86e-01 | 1.00e+00 |
|  | 1 | 9129487 | 9129791 | PDFSTATSENSEMBLUCSC | 305 | 4 | 198 | gene;island | 0.77757 | 0.64820 | Not queried | 2.02 | 4.86e-01 | 1.00e+00 |
|  | 19 | 55417361 | 55417500 | PDFSTATSENSEMBLUCSC | 140 | 5 | 21030 | tss | 0.54505 | 0.84329 | Not queried | 2.02 | 9.52e-02 | 1.00e+00 |
|  | 16 | 3096477 | 3096711 | PDFSTATSENSEMBLUCSC | 235 | 6 | 15604 | island | 0.21683 | 0.03632 | Not queried | 2.02 | 9.31e-02 | 1.00e+00 |
|  | 19 | 59066570 | 59066700 | PDFSTATSENSEMBLUCSC | 131 | 7 | 21113 | tss;island | 0.25833 | 0.63635 | Not queried | 2.02 | 5.83e-04 | 1.00e+00 |
|  | 11 | 9335544 | 9335706 | PDFSTATSENSEMBLUCSC | 163 | 4 | 6485 | gene;island | 0.01663 | 0.04044 | Not queried | 2.02 | 5.71e-02 | 1.00e+00 |
|  | 4 | 7026788 | 7027124 | PDFSTATSENSEMBLUCSC | 337 | 4 | 29886 | gene;island | 0.82404 | 0.93675 | Not queried | 2.02 | 2.00e-01 | 1.00e+00 |
|  | 21 | 42880448 | 42880714 | PDFSTATSENSEMBLUCSC | 267 | 5 | 26518 | tss;island | 0.26500 | 0.14132 | Not queried | 2.02 | 7.94e-03 | 1.00e+00 |
|  | 7 | 152456579 | 152456815 | PDFSTATSENSEMBLUCSC | 237 | 5 | 37943 | tss;island | 0.02321 | 0.01698 | Not queried | 2.02 | 4.21e-01 | 1.00e+00 |
|  | 17 | 41466953 | 41467212 | PDFSTATSENSEMBLUCSC | 260 | 4 | 17355 | tss | 0.01306 | 0.05795 | Not queried | 2.01 | 1.14e-01 | 1.00e+00 |
|  | 2 | 37572102 | 37572423 | PDFSTATSENSEMBLUCSC | 322 | 4 | 22612 | gene;island | 0.04122 | 0.15703 | Not queried | 2.01 | 1.14e-01 | 1.00e+00 |
|  | 2 | 11796786 | 11797017 | PDFSTATSENSEMBLUCSC | 232 | 4 | 24043 | island | 0.43716 | 0.75793 | Not queried | 2.01 | 2.86e-02 | 1.00e+00 |
|  | 8 | 144790187 | 144790410 | PDFSTATSENSEMBLUCSC | 224 | 5 | 40616 | island | 0.69550 | 0.90693 | Not queried | 2.01 | 7.94e-03 | 1.00e+00 |
|  | 8 | 99076526 | 99076734 | PDFSTATSENSEMBLUCSC | 209 | 4 | 39770 | tss | 0.43349 | 0.09346 | Not queried | 2.01 | 3.43e-01 | 1.00e+00 |
|  | X | 55187543 | 55188047 | PDFSTATSENSEMBLUCSC | 505 | 9 | 41341 | tss | 0.19007 | 0.02851 | Not queried | 2.01 | 4.00e-02 | 1.00e+00 |
|  | 20 | 61885236 | 61885291 | PDFSTATSENSEMBLUCSC | 56 | 6 | 25853 | tss | 0.00731 | 0.01221 | Not queried | 2.01 | 3.94e-01 | 1.00e+00 |
|  | 7 | 102087552 | 102087746 | PDFSTATSENSEMBLUCSC | 195 | 4 | 37104 | gene | 0.62762 | 0.92454 | Not queried | 2.01 | 1.14e-01 | 1.00e+00 |
|  | 1 | 14027378 | 14027581 | PDFSTATSENSEMBLUCSC | 204 | 4 | 235 | gene;island | 0.27298 | 0.04418 | Not queried | 2.01 | 1.14e-01 | 1.00e+00 |
|  | 17 | 7757933 | 7758854 | PDFSTATSENSEMBLUCSC | 922 | 17 | 17941 | island | 0.57199 | 0.87623 | Not queried | 2.01 | 7.44e-05 | 3.82e-01 |
|  | 19 | 18496984 | 18497201 | PDFSTATSENSEMBLUCSC | 218 | 4 | 19899 | gene | 0.69161 | 0.82073 | Not queried | 2.01 | 3.43e-01 | 1.00e+00 |
|  | 1 | 166039444 | 166039918 | PDFSTATSENSEMBLUCSC | 475 | 5 | 865 | gene | 0.20031 | 0.39340 | Not queried | 2.01 | 9.52e-02 | 1.00e+00 |
|  | 14 | 77647955 | 77648040 | PDFSTATSENSEMBLUCSC | 86 | 5 | 12500 | tss | 0.05473 | 0.08149 | Not queried | 2.01 | 5.56e-02 | 1.00e+00 |
|  | 12 | 6166874 | 6167147 | PDFSTATSENSEMBLUCSC | 274 | 4 | 8997 | gene;island | 0.58630 | 0.25804 | Not queried | 2.01 | 2.86e-02 | 1.00e+00 |
|  | 13 | 25874859 | 25875646 | PDFSTATSENSEMBLUCSC | 788 | 10 | 11323 | tss | 0.03092 | 0.39757 | Not queried | 2.01 | 1.90e-01 | 1.00e+00 |
|  | 11 | 518649 | 519271 | PDFSTATSENSEMBLUCSC | 623 | 5 | 7836 | island | 0.26875 | 0.60034 | Not queried | 2.01 | 4.21e-01 | 1.00e+00 |
|  | 17 | 42385942 | 42386419 | PDFSTATSENSEMBLUCSC | 478 | 7 | 16578 | gene | 0.49570 | 0.15191 | Not queried | 2.01 | 1.28e-01 | 1.00e+00 |
|  | 15 | 90455635 | 90455799 | PDFSTATSENSEMBLUCSC | 165 | 4 | 13446 | gene;island | 0.01347 | 0.03667 | Not queried | 2.01 | 5.71e-02 | 1.00e+00 |
|  | 10 | 102731747 | 102732323 | PDFSTATSENSEMBLUCSC | 577 | 8 | 6021 | island | 0.65137 | 0.80292 | Not queried | 2.01 | 8.30e-02 | 1.00e+00 |
|  | 22 | 27053195 | 27053470 | PDFSTATSENSEMBLUCSC | 276 | 10 | 26995 | tss | 0.08837 | 0.03023 | Not queried | 2.01 | 6.84e-03 | 1.00e+00 |
|  | 8 | 144798309 | 144798477 | PDFSTATSENSEMBLUCSC | 169 | 7 | 39895 | tss | 0.01875 | 0.06145 | Not queried | 2.01 | 5.30e-02 | 1.00e+00 |
|  | 17 | 20687681 | 20687912 | PDFSTATSENSEMBLUCSC | 232 | 4 | 18077 | island | 0.31823 | 0.57819 | Not queried | 2.00 | 2.00e-01 | 1.00e+00 |
|  | X | 114423840 | 114424082 | PDFSTATSENSEMBLUCSC | 243 | 4 | 41084 | gene;island | 0.55783 | 0.87836 | Not queried | 2.00 | 2.00e-01 | 1.00e+00 |
|  | 11 | 2153991 | 2154432 | PDFSTATSENSEMBLUCSC | 442 | 6 | 6427 | gene;island | 0.23433 | 0.56969 | Not queried | 2.00 | 2.60e-02 | 1.00e+00 |
|  | 5 | 141488016 | 141488153 | PDFSTATSENSEMBLUCSC | 138 | 5 | 32336 | tss;island | 0.20461 | 0.06038 | Not queried | 2.00 | 5.48e-01 | 1.00e+00 |
|  | 18 | 5196738 | 5197071 | PDFSTATSENSEMBLUCSC | 334 | 4 | 19013 | gene;island | 0.03632 | 0.05779 | Not queried | 2.00 | 4.86e-01 | 1.00e+00 |
|  | 7 | 150026554 | 150026909 | PDFSTATSENSEMBLUCSC | 356 | 4 | 37912 | tss | 0.72456 | 0.75619 | Not queried | 2.00 | 6.86e-01 | 1.00e+00 |
|  | 1 | 174968089 | 174968509 | PDFSTATSENSEMBLUCSC | 421 | 9 | 2098 | tss;island | 0.07763 | 0.02559 | Not queried | 2.00 | 1.42e-02 | 1.00e+00 |
|  | 13 | 108922032 | 108922504 | PDFSTATSENSEMBLUCSC | 473 | 7 | 11195 | gene | 0.22226 | 0.58397 | Not queried | 2.00 | 1.28e-01 | 1.00e+00 |
|  | 2 | 74010290 | 74010621 | PDFSTATSENSEMBLUCSC | 332 | 4 | 23407 | tss;island | 0.51303 | 0.80804 | Not queried | 2.00 | 2.86e-02 | 1.00e+00 |
|  | 11 | 10472001 | 10472161 | PDFSTATSENSEMBLUCSC | 161 | 7 | 7124 | tss;island | 0.14060 | 0.05369 | Not queried | 2.00 | 1.75e-02 | 1.00e+00 |
|  | 2 | 23840537 | 23840763 | PDFSTATSENSEMBLUCSC | 227 | 4 | 22557 | gene | 0.65274 | 0.89873 | Not queried | 2.00 | 1.14e-01 | 1.00e+00 |
|  | 6 | 33043072 | 33043220 | PDFSTATSENSEMBLUCSC | 149 | 4 | 34824 | tss | 0.82728 | 0.63522 | Not queried | 2.00 | 6.86e-01 | 1.00e+00 |
|  | 4 | 7395388 | 7395612 | PDFSTATSENSEMBLUCSC | 225 | 4 | 29891 | gene | 0.72963 | 0.89499 | Not queried | 2.00 | 2.00e-01 | 1.00e+00 |
|  | 21 | 34398085 | 34398265 | PDFSTATSENSEMBLUCSC | 181 | 8 | 26601 | island | 0.01371 | 0.07794 | Not queried | 2.00 | 1.86e-03 | 1.00e+00 |
|  | 8 | 63776508 | 63776762 | PDFSTATSENSEMBLUCSC | 255 | 4 | 39256 | gene | 0.46741 | 0.81984 | Not queried | 2.00 | 2.00e-01 | 1.00e+00 |
|  | 2 | 144994885 | 144995206 | PDFSTATSENSEMBLUCSC | 322 | 4 | 22851 | gene | 0.82114 | 0.94898 | Not queried | 2.00 | 2.86e-02 | 1.00e+00 |
|  | 7 | 31726494 | 31726704 | PDFSTATSENSEMBLUCSC | 211 | 5 | 37458 | tss | 0.92095 | 0.96451 | Not queried | 2.00 | 9.52e-02 | 1.00e+00 |
|  | 11 | 94276895 | 94276960 | PDFSTATSENSEMBLUCSC | 66 | 4 | 7609 | tss | 0.18942 | 0.05438 | Not queried | 2.00 | 2.86e-02 | 1.00e+00 |
|  | 19 | 17905589 | 17905835 | PDFSTATSENSEMBLUCSC | 247 | 5 | 20564 | tss;island | 0.55068 | 0.09054 | Not queried | 2.00 | 3.17e-02 | 1.00e+00 |
|  | 12 | 106533840 | 106533903 | PDFSTATSENSEMBLUCSC | 64 | 5 | 9941 | tss | 0.72111 | 0.39330 | Not queried | 2.00 | 3.17e-02 | 1.00e+00 |
|  | 7 | 149629 | 150084 | PDFSTATSENSEMBLUCSC | 456 | 5 | 37965 | island | 0.54094 | 0.82452 | Not queried | 2.00 | 5.48e-01 | 1.00e+00 |
|  | 15 | 22646692 | 22647092 | PDFSTATSENSEMBLUCSC | 401 | 4 | 13910 | island | 0.27936 | 0.60039 | Not queried | 2.00 | 2.86e-02 | 1.00e+00 |
|  | 3 | 123167507 | 123167770 | PDFSTATSENSEMBLUCSC | 264 | 4 | 28502 | tss;island | 0.23021 | 0.05209 | Not queried | 1.99 | 3.43e-01 | 1.00e+00 |
|  | 12 | 54385275 | 54385625 | PDFSTATSENSEMBLUCSC | 351 | 5 | 10460 | island | 0.68784 | 0.31672 | Not queried | 1.99 | 7.94e-03 | 1.00e+00 |
|  | 18 | 19997912 | 19998049 | PDFSTATSENSEMBLUCSC | 138 | 7 | 19167 | tss | 0.93075 | 0.75124 | Not queried | 1.99 | 5.30e-02 | 1.00e+00 |
|  | 17 | 61615504 | 61615913 | PDFSTATSENSEMBLUCSC | 410 | 4 | 16693 | gene;island | 0.10035 | 0.30355 | Not queried | 1.99 | 2.86e-02 | 1.00e+00 |
|  | 3 | 170136159 | 170136539 | PDFSTATSENSEMBLUCSC | 381 | 9 | 28671 | tss;island | 0.09736 | 0.12113 | Not queried | 1.99 | 1.61e-01 | 1.00e+00 |
|  | X | 9734033 | 9734312 | PDFSTATSENSEMBLUCSC | 280 | 4 | 41179 | tss | 0.76937 | 0.93448 | Not queried | 1.99 | 4.86e-01 | 1.00e+00 |
|  | 8 | 144120335 | 144120648 | PDFSTATSENSEMBLUCSC | 314 | 5 | 39879 | tss | 0.58223 | 0.25967 | Not queried | 1.99 | 3.17e-02 | 1.00e+00 |
|  | 1 | 19181040 | 19181459 | PDFSTATSENSEMBLUCSC | 420 | 6 | 275 | gene;island | 0.46056 | 0.72383 | Not queried | 1.99 | 2.16e-03 | 1.00e+00 |
|  | 1 | 67772896 | 67773044 | PDFSTATSENSEMBLUCSC | 149 | 5 | 1680 | tss | 0.29897 | 0.07589 | Not queried | 1.99 | 2.22e-01 | 1.00e+00 |
|  | 10 | 134893401 | 134894048 | PDFSTATSENSEMBLUCSC | 648 | 8 | 6312 | island | 0.56840 | 0.79816 | Not queried | 1.99 | 1.05e-01 | 1.00e+00 |
|  | 19 | 51141227 | 51141413 | PDFSTATSENSEMBLUCSC | 187 | 4 | 20143 | gene;island | 0.48268 | 0.19120 | Not queried | 1.99 | 2.00e-01 | 1.00e+00 |
|  | 16 | 1384626 | 1384908 | PDFSTATSENSEMBLUCSC | 283 | 5 | 15498 | island | 0.33397 | 0.11221 | Not queried | 1.99 | 5.48e-01 | 1.00e+00 |
|  | 3 | 11034279 | 11034345 | PDFSTATSENSEMBLUCSC | 67 | 4 | 28111 | tss | 0.06797 | 0.38356 | Not queried | 1.99 | 5.71e-02 | 1.00e+00 |
|  | 7 | 63774078 | 63774504 | PDFSTATSENSEMBLUCSC | 427 | 5 | 36960 | gene | 0.08073 | 0.02338 | Not queried | 1.99 | 1.51e-01 | 1.00e+00 |
|  | 6 | 29079939 | 29080437 | PDFSTATSENSEMBLUCSC | 499 | 5 | 33587 | gene | 0.21207 | 0.56391 | Not queried | 1.99 | 1.59e-02 | 1.00e+00 |
|  | 14 | 102554826 | 102554977 | PDFSTATSENSEMBLUCSC | 152 | 4 | 12198 | gene;tss;island | 0.51401 | 0.19835 | Not queried | 1.99 | 2.86e-02 | 1.00e+00 |
|  | 7 | 28220498 | 28220645 | PDFSTATSENSEMBLUCSC | 148 | 8 | 37438 | tss;island | 0.06718 | 0.02077 | Not queried | 1.99 | 2.07e-02 | 1.00e+00 |
|  | 17 | 40489513 | 40489785 | PDFSTATSENSEMBLUCSC | 273 | 5 | 16544 | gene | 0.87118 | 0.96402 | Not queried | 1.99 | 5.56e-02 | 1.00e+00 |
|  | 22 | 39640835 | 39641154 | PDFSTATSENSEMBLUCSC | 320 | 7 | 27458 | island | 0.05689 | 0.21533 | Not queried | 1.99 | 2.33e-03 | 1.00e+00 |
|  | 18 | 12253749 | 12254566 | PDFSTATSENSEMBLUCSC | 818 | 18 | 19153 | tss | 0.32709 | 0.64075 | Not queried | 1.99 | 9.59e-05 | 4.93e-01 |
|  | 19 | 16830613 | 16830749 | PDFSTATSENSEMBLUCSC | 137 | 4 | 20539 | tss | 0.72030 | 0.91113 | Not queried | 1.99 | 1.14e-01 | 1.00e+00 |
|  | 17 | 33700180 | 33700295 | PDFSTATSENSEMBLUCSC | 116 | 4 | 16484 | gene;island | 0.04125 | 0.14508 | Not queried | 1.99 | 1.14e-01 | 1.00e+00 |
|  | 6 | 117584596 | 117584815 | PDFSTATSENSEMBLUCSC | 220 | 4 | 36373 | island | 0.28111 | 0.66262 | Not queried | 1.98 | 2.86e-02 | 1.00e+00 |
|  | 16 | 88569083 | 88569490 | PDFSTATSENSEMBLUCSC | 408 | 5 | 14875 | gene;island | 0.63132 | 0.79750 | Not queried | 1.98 | 5.56e-02 | 1.00e+00 |
|  | X | 117629034 | 117629344 | PDFSTATSENSEMBLUCSC | 311 | 5 | 41481 | tss;island | 0.26691 | 0.06216 | Not queried | 1.98 | 2.22e-01 | 1.00e+00 |
|  | X | 54556330 | 54556538 | PDFSTATSENSEMBLUCSC | 209 | 7 | 41335 | tss | 0.09541 | 0.03030 | Not queried | 1.98 | 9.73e-02 | 1.00e+00 |
|  | 2 | 176968584 | 176968920 | PDFSTATSENSEMBLUCSC | 337 | 4 | 24856 | island | 0.27907 | 0.08451 | Not queried | 1.98 | 2.00e-01 | 1.00e+00 |
|  | 1 | 60392140 | 60392567 | PDFSTATSENSEMBLUCSC | 428 | 6 | 3319 | island | 0.17384 | 0.04793 | Not queried | 1.98 | 4.33e-03 | 1.00e+00 |
|  | 16 | 3085571 | 3085982 | PDFSTATSENSEMBLUCSC | 412 | 4 | 15017 | tss | 0.64462 | 0.50122 | Not queried | 1.98 | 6.86e-01 | 1.00e+00 |
|  | 1 | 25566331 | 25566753 | PDFSTATSENSEMBLUCSC | 423 | 5 | 2862 | island | 0.02082 | 0.07743 | Not queried | 1.98 | 2.22e-01 | 1.00e+00 |
|  | X | 105969622 | 105969840 | PDFSTATSENSEMBLUCSC | 219 | 7 | 41456 | tss | 0.01976 | 0.04789 | Not queried | 1.98 | 2.62e-02 | 1.00e+00 |
|  | 16 | 66586309 | 66586415 | PDFSTATSENSEMBLUCSC | 107 | 5 | 15249 | tss | 0.11458 | 0.02559 | Not queried | 1.98 | 7.94e-03 | 1.00e+00 |
|  | 16 | 433439 | 433825 | PDFSTATSENSEMBLUCSC | 387 | 5 | 14501 | gene;island | 0.02255 | 0.04813 | Not queried | 1.98 | 9.52e-02 | 1.00e+00 |
|  | 1 | 2125049 | 2125243 | PDFSTATSENSEMBLUCSC | 195 | 7 | 69 | gene;tss;island | 0.84648 | 0.58093 | Not queried | 1.98 | 5.83e-04 | 1.00e+00 |
|  | 16 | 85060822 | 85061228 | PDFSTATSENSEMBLUCSC | 407 | 5 | 15365 | tss;island | 0.04410 | 0.05776 | Not queried | 1.98 | 5.48e-01 | 1.00e+00 |
|  | 1 | 25257505 | 25258332 | PDFSTATSENSEMBLUCSC | 828 | 21 | 330 | gene;island | 0.33984 | 0.09938 | RUNX3 (margin: RUNX3) | 1.98 | 3.44e-08 | 1.77e-04 |
|  | 1 | 19665070 | 19665240 | PDFSTATSENSEMBLUCSC | 171 | 4 | 2776 | island | 0.65706 | 0.38805 | Not queried | 1.98 | 5.71e-02 | 1.00e+00 |
|  | 19 | 49685846 | 49686254 | PDFSTATSENSEMBLUCSC | 409 | 5 | 20109 | gene;island | 0.93601 | 0.72926 | Not queried | 1.98 | 9.52e-02 | 1.00e+00 |
|  | 4 | 102712010 | 102712397 | PDFSTATSENSEMBLUCSC | 388 | 4 | 30025 | gene;island | 0.18771 | 0.05804 | Not queried | 1.98 | 1.14e-01 | 1.00e+00 |
|  | 7 | 100612725 | 100612891 | PDFSTATSENSEMBLUCSC | 167 | 4 | 37731 | tss | 0.31408 | 0.61765 | Not queried | 1.97 | 2.86e-02 | 1.00e+00 |
|  | 14 | 29247148 | 29247705 | PDFSTATSENSEMBLUCSC | 558 | 6 | 12025 | gene;island | 0.13173 | 0.38734 | Not queried | 1.97 | 9.31e-02 | 1.00e+00 |
|  | 19 | 12997997 | 12998135 | PDFSTATSENSEMBLUCSC | 139 | 4 | 21596 | island | 0.55527 | 0.24207 | Not queried | 1.97 | 2.00e-01 | 1.00e+00 |
|  | 11 | 1873757 | 1874093 | PDFSTATSENSEMBLUCSC | 337 | 5 | 7029 | tss | 0.50598 | 0.85365 | Not queried | 1.97 | 1.51e-01 | 1.00e+00 |
|  | 1 | 201252200 | 201252510 | PDFSTATSENSEMBLUCSC | 311 | 6 | 2161 | tss | 0.41867 | 0.76729 | Not queried | 1.97 | 2.16e-03 | 1.00e+00 |
|  | 1 | 201252200 | 201252734 | PDFSTATSENSEMBLUCSC | 535 | 8 | 4061 | island | 0.42575 | 0.76729 | Not queried | 1.97 | 1.86e-03 | 1.00e+00 |
|  | 7 | 44104860 | 44105166 | PDFSTATSENSEMBLUCSC | 307 | 4 | 36926 | gene | 0.15016 | 0.40278 | Not queried | 1.97 | 5.71e-02 | 1.00e+00 |
|  | 2 | 219925065 | 219925330 | PDFSTATSENSEMBLUCSC | 266 | 7 | 25067 | island | 0.03174 | 0.11764 | Not queried | 1.97 | 7.28e-02 | 1.00e+00 |
|  | 18 | 12253749 | 12254690 | PDFSTATSENSEMBLUCSC | 942 | 19 | 19367 | island | 0.34224 | 0.64101 | Not queried | 1.97 | 2.83e-04 | 1.00e+00 |
|  | 2 | 27665017 | 27665150 | PDFSTATSENSEMBLUCSC | 134 | 6 | 23232 | tss | 0.28833 | 0.64586 | Not queried | 1.97 | 2.16e-03 | 1.00e+00 |
|  | 19 | 708469 | 708709 | PDFSTATSENSEMBLUCSC | 241 | 5 | 20241 | tss;island | 0.28174 | 0.11088 | Not queried | 1.97 | 3.10e-01 | 1.00e+00 |
|  | 11 | 31839521 | 31839947 | PDFSTATSENSEMBLUCSC | 427 | 7 | 7204 | tss | 0.44793 | 0.09224 | Not queried | 1.97 | 7.28e-02 | 1.00e+00 |
|  | 11 | 31839314 | 31839947 | PDFSTATSENSEMBLUCSC | 634 | 9 | 8146 | island | 0.22810 | 0.07325 | Not queried | 1.97 | 5.03e-02 | 1.00e+00 |
|  | 16 | 31227272 | 31227515 | PDFSTATSENSEMBLUCSC | 244 | 4 | 14704 | gene;island | 0.02986 | 0.11451 | Not queried | 1.97 | 1.14e-01 | 1.00e+00 |
|  | 2 | 97193006 | 97193289 | PDFSTATSENSEMBLUCSC | 284 | 4 | 24489 | island | 0.46890 | 0.19486 | Not queried | 1.97 | 2.86e-02 | 1.00e+00 |
|  | 20 | 23030343 | 23030533 | PDFSTATSENSEMBLUCSC | 191 | 5 | 25597 | tss | 0.02140 | 0.08844 | Not queried | 1.97 | 7.94e-03 | 1.00e+00 |
|  | 20 | 23030102 | 23030533 | PDFSTATSENSEMBLUCSC | 432 | 7 | 26008 | island | 0.02140 | 0.11655 | Not queried | 1.97 | 1.17e-03 | 1.00e+00 |
|  | 3 | 54121761 | 54122146 | PDFSTATSENSEMBLUCSC | 386 | 6 | 29166 | island | 0.54103 | 0.14454 | Not queried | 1.97 | 2.60e-02 | 1.00e+00 |
|  | X | 70150722 | 70151209 | PDFSTATSENSEMBLUCSC | 488 | 7 | 41853 | island | 0.07502 | 0.02074 | Not queried | 1.97 | 2.33e-03 | 1.00e+00 |
|  | 16 | 1080388 | 1080808 | PDFSTATSENSEMBLUCSC | 421 | 4 | 15478 | island | 0.57451 | 0.82243 | Not queried | 1.97 | 2.86e-02 | 1.00e+00 |
|  | 5 | 45695467 | 45695922 | PDFSTATSENSEMBLUCSC | 456 | 4 | 31551 | gene;island | 0.05346 | 0.30343 | Not queried | 1.97 | 5.71e-02 | 1.00e+00 |
|  | 17 | 6796745 | 6797119 | PDFSTATSENSEMBLUCSC | 375 | 5 | 16342 | gene;island | 0.74166 | 0.42363 | Not queried | 1.97 | 1.59e-02 | 1.00e+00 |
|  | 16 | 967929 | 968265 | PDFSTATSENSEMBLUCSC | 337 | 6 | 14525 | gene;island | 0.63832 | 0.89034 | Not queried | 1.97 | 9.31e-02 | 1.00e+00 |
|  | X | 153665624 | 153665799 | PDFSTATSENSEMBLUCSC | 176 | 4 | 41160 | gene;island | 0.00477 | 0.01324 | Not queried | 1.96 | 2.86e-02 | 1.00e+00 |
|  | 14 | 60973773 | 60974039 | PDFSTATSENSEMBLUCSC | 267 | 4 | 12875 | island | 0.17533 | 0.04190 | Not queried | 1.96 | 2.86e-02 | 1.00e+00 |
|  | 19 | 5822318 | 5822630 | PDFSTATSENSEMBLUCSC | 313 | 4 | 20347 | tss;island | 0.30783 | 0.69944 | Not queried | 1.96 | 1.14e-01 | 1.00e+00 |
|  | 6 | 29623646 | 29623992 | PDFSTATSENSEMBLUCSC | 347 | 4 | 34687 | tss | 0.57157 | 0.83217 | Not queried | 1.96 | 2.00e-01 | 1.00e+00 |
|  | 16 | 29801602 | 29801967 | PDFSTATSENSEMBLUCSC | 366 | 7 | 15137 | tss;island | 0.02615 | 0.01691 | Not queried | 1.96 | 6.20e-01 | 1.00e+00 |
|  | 4 | 987303 | 987672 | PDFSTATSENSEMBLUCSC | 370 | 5 | 30187 | tss | 0.72886 | 0.84815 | Not queried | 1.96 | 1.59e-02 | 1.00e+00 |
|  | 6 | 32814921 | 32815091 | PDFSTATSENSEMBLUCSC | 171 | 6 | 34088 | gene;island | 0.70706 | 0.28341 | Not queried | 1.96 | 6.49e-02 | 1.00e+00 |
|  | 1 | 46599002 | 46599128 | PDFSTATSENSEMBLUCSC | 127 | 4 | 1591 | tss;island | 0.00500 | 0.01602 | Not queried | 1.96 | 4.86e-01 | 1.00e+00 |
|  | 1 | 202858691 | 202858826 | PDFSTATSENSEMBLUCSC | 136 | 4 | 2180 | tss;island | 0.52456 | 0.88745 | Not queried | 1.96 | 2.00e-01 | 1.00e+00 |
|  | 2 | 119609429 | 119609839 | PDFSTATSENSEMBLUCSC | 411 | 6 | 24612 | island | 0.69443 | 0.53167 | Not queried | 1.96 | 2.16e-03 | 1.00e+00 |
|  | 17 | 72667005 | 72667394 | PDFSTATSENSEMBLUCSC | 390 | 5 | 18686 | island | 0.08222 | 0.26595 | Not queried | 1.96 | 1.59e-02 | 1.00e+00 |
|  | X | 102318831 | 102319120 | PDFSTATSENSEMBLUCSC | 290 | 6 | 41057 | gene;island | 0.23835 | 0.09403 | Not queried | 1.95 | 4.33e-03 | 1.00e+00 |
|  | 20 | 23015899 | 23015936 | PDFSTATSENSEMBLUCSC | 38 | 5 | 25596 | tss;island | 0.53241 | 0.80482 | Not queried | 1.95 | 1.59e-02 | 1.00e+00 |
|  | 12 | 14518655 | 14518974 | PDFSTATSENSEMBLUCSC | 320 | 4 | 9039 | gene | 0.07614 | 0.20898 | Not queried | 1.95 | 5.71e-02 | 1.00e+00 |
|  | 10 | 131682251 | 131682509 | PDFSTATSENSEMBLUCSC | 259 | 5 | 4818 | gene | 0.70944 | 0.92314 | Not queried | 1.95 | 3.17e-02 | 1.00e+00 |
|  | 8 | 20107455 | 20107758 | PDFSTATSENSEMBLUCSC | 304 | 4 | 39159 | gene;island | 0.66733 | 0.88570 | Not queried | 1.95 | 2.00e-01 | 1.00e+00 |
|  | 10 | 98480463 | 98480474 | PDFSTATSENSEMBLUCSC | 12 | 4 | 5238 | tss;island | 0.06841 | 0.02792 | Not queried | 1.95 | 2.00e-01 | 1.00e+00 |
|  | 12 | 54867429 | 54867653 | PDFSTATSENSEMBLUCSC | 225 | 4 | 9739 | tss | 0.85885 | 0.61852 | Not queried | 1.95 | 3.43e-01 | 1.00e+00 |
|  | 1 | 24645802 | 24646392 | PDFSTATSENSEMBLUCSC | 591 | 13 | 2852 | island | 0.02458 | 0.09434 | Not queried | 1.95 | 2.97e-04 | 1.00e+00 |
|  | 17 | 38521067 | 38521482 | PDFSTATSENSEMBLUCSC | 416 | 4 | 17299 | tss;island | 0.92156 | 0.87123 | Not queried | 1.95 | 2.00e-01 | 1.00e+00 |
|  | 11 | 62457265 | 62457345 | PDFSTATSENSEMBLUCSC | 81 | 5 | 7337 | tss;island | 0.61760 | 0.88085 | Not queried | 1.95 | 7.94e-03 | 1.00e+00 |
|  | 9 | 1042605 | 1042970 | PDFSTATSENSEMBLUCSC | 366 | 4 | 40796 | island | 0.13201 | 0.22750 | Not queried | 1.95 | 2.00e-01 | 1.00e+00 |
|  | 17 | 54230419 | 54230793 | PDFSTATSENSEMBLUCSC | 375 | 5 | 17487 | tss | 0.15208 | 0.40894 | Not queried | 1.95 | 3.17e-02 | 1.00e+00 |
|  | 8 | 1109986 | 1110263 | PDFSTATSENSEMBLUCSC | 278 | 4 | 39951 | island | 0.60887 | 0.86960 | Not queried | 1.95 | 5.71e-02 | 1.00e+00 |
|  | 16 | 84681507 | 84681729 | PDFSTATSENSEMBLUCSC | 223 | 4 | 15363 | tss;island | 0.32908 | 0.08259 | Not queried | 1.95 | 3.43e-01 | 1.00e+00 |
|  | 10 | 120355139 | 120355427 | PDFSTATSENSEMBLUCSC | 289 | 8 | 6169 | island | 0.22134 | 0.51548 | Not queried | 1.95 | 3.79e-02 | 1.00e+00 |
|  | 17 | 6899522 | 6899888 | PDFSTATSENSEMBLUCSC | 367 | 5 | 16344 | gene | 0.57090 | 0.69158 | Not queried | 1.95 | 1.00e+00 | 1.00e+00 |
|  | 6 | 169951905 | 169952299 | PDFSTATSENSEMBLUCSC | 395 | 4 | 34504 | gene | 0.87827 | 0.95475 | Not queried | 1.94 | 2.86e-02 | 1.00e+00 |
|  | 15 | 89954560 | 89955171 | PDFSTATSENSEMBLUCSC | 612 | 6 | 14406 | island | 0.16737 | 0.33559 | Not queried | 1.94 | 3.10e-01 | 1.00e+00 |
|  | 10 | 119292062 | 119292587 | PDFSTATSENSEMBLUCSC | 526 | 8 | 4765 | gene;island | 0.24397 | 0.56896 | Not queried | 1.94 | 2.07e-02 | 1.00e+00 |
|  | 17 | 1617291 | 1617465 | PDFSTATSENSEMBLUCSC | 175 | 5 | 16960 | tss | 0.06030 | 0.14728 | Not queried | 1.94 | 2.22e-01 | 1.00e+00 |
|  | 6 | 170584106 | 170584501 | PDFSTATSENSEMBLUCSC | 396 | 4 | 36625 | island | 0.92654 | 0.78651 | Not queried | 1.94 | 2.86e-02 | 1.00e+00 |
|  | 1 | 34090664 | 34090941 | PDFSTATSENSEMBLUCSC | 278 | 5 | 404 | gene;island | 0.13364 | 0.32374 | Not queried | 1.94 | 2.22e-01 | 1.00e+00 |
|  | 6 | 32371488 | 32371538 | PDFSTATSENSEMBLUCSC | 51 | 4 | 34047 | gene | 0.17467 | 0.43151 | Not queried | 1.94 | 2.00e-01 | 1.00e+00 |
|  | 19 | 21541756 | 21541917 | PDFSTATSENSEMBLUCSC | 162 | 5 | 19924 | gene | 0.39902 | 0.12793 | Not queried | 1.94 | 5.56e-02 | 1.00e+00 |
|  | 1 | 26737005 | 26737262 | PDFSTATSENSEMBLUCSC | 258 | 5 | 1388 | tss | 0.08495 | 0.17777 | Not queried | 1.94 | 3.17e-02 | 1.00e+00 |
|  | 19 | 55458886 | 55459282 | PDFSTATSENSEMBLUCSC | 397 | 6 | 21031 | tss | 0.79199 | 0.92569 | Not queried | 1.94 | 6.49e-02 | 1.00e+00 |
|  | 13 | 40746370 | 40746648 | PDFSTATSENSEMBLUCSC | 279 | 4 | 11656 | island | 0.32533 | 0.60832 | Not queried | 1.94 | 3.43e-01 | 1.00e+00 |
|  | 3 | 49947639 | 49947934 | PDFSTATSENSEMBLUCSC | 296 | 4 | 27750 | gene;island | 0.96277 | 0.74025 | Not queried | 1.94 | 1.14e-01 | 1.00e+00 |
|  | 5 | 3326343 | 3326731 | PDFSTATSENSEMBLUCSC | 389 | 5 | 32602 | island | 0.63473 | 0.87099 | Not queried | 1.94 | 9.52e-02 | 1.00e+00 |
|  | 14 | 96967798 | 96968050 | PDFSTATSENSEMBLUCSC | 253 | 4 | 12567 | tss;island | 0.04243 | 0.01027 | Not queried | 1.94 | 2.00e-01 | 1.00e+00 |
|  | 6 | 32084298 | 32084658 | PDFSTATSENSEMBLUCSC | 361 | 6 | 33989 | gene | 0.71158 | 0.89119 | Not queried | 1.94 | 2.60e-02 | 1.00e+00 |
|  | 22 | 19712623 | 19712750 | PDFSTATSENSEMBLUCSC | 128 | 4 | 27260 | island | 0.41419 | 0.75831 | Not queried | 1.94 | 3.43e-01 | 1.00e+00 |
|  | 8 | 102504447 | 102504859 | PDFSTATSENSEMBLUCSC | 413 | 9 | 40441 | island | 0.02110 | 0.13104 | Not queried | 1.94 | 1.06e-02 | 1.00e+00 |
|  | 12 | 81110749 | 81111012 | PDFSTATSENSEMBLUCSC | 264 | 4 | 9201 | gene | 0.56909 | 0.77323 | Not queried | 1.94 | 2.86e-02 | 1.00e+00 |
|  | 10 | 133796164 | 133796559 | PDFSTATSENSEMBLUCSC | 396 | 6 | 5426 | tss;island | 0.67394 | 0.35091 | Not queried | 1.94 | 3.94e-01 | 1.00e+00 |
|  | 14 | 24457893 | 24458014 | PDFSTATSENSEMBLUCSC | 122 | 6 | 12280 | tss | 0.03227 | 0.11988 | Not queried | 1.94 | 1.80e-01 | 1.00e+00 |
|  | 13 | 36050788 | 36051073 | PDFSTATSENSEMBLUCSC | 286 | 8 | 11099 | gene;island | 0.03085 | 0.13153 | Not queried | 1.93 | 1.30e-01 | 1.00e+00 |
|  | 17 | 30669342 | 30669494 | PDFSTATSENSEMBLUCSC | 153 | 4 | 17221 | tss | 0.24416 | 0.07646 | Not queried | 1.93 | 4.86e-01 | 1.00e+00 |
|  | 13 | 50702675 | 50703026 | PDFSTATSENSEMBLUCSC | 352 | 6 | 11713 | island | 0.32262 | 0.15615 | Not queried | 1.93 | 2.60e-02 | 1.00e+00 |
|  | 8 | 41583018 | 41583523 | PDFSTATSENSEMBLUCSC | 506 | 5 | 39229 | gene;island | 0.44761 | 0.76805 | Not queried | 1.93 | 5.56e-02 | 1.00e+00 |
|  | 13 | 21900392 | 21900591 | PDFSTATSENSEMBLUCSC | 200 | 4 | 11560 | island | 0.20943 | 0.06353 | Not queried | 1.93 | 1.14e-01 | 1.00e+00 |
|  | 4 | 57522689 | 57523055 | PDFSTATSENSEMBLUCSC | 367 | 7 | 30364 | tss | 0.61849 | 0.16764 | Not queried | 1.93 | 7.28e-02 | 1.00e+00 |
|  | 11 | 64058878 | 64059057 | PDFSTATSENSEMBLUCSC | 180 | 4 | 6659 | gene | 0.35318 | 0.19562 | Not queried | 1.93 | 3.43e-01 | 1.00e+00 |
|  | 11 | 64058550 | 64059057 | PDFSTATSENSEMBLUCSC | 508 | 6 | 8379 | island | 0.55960 | 0.27755 | Not queried | 1.93 | 1.80e-01 | 1.00e+00 |
|  | 5 | 112849156 | 112849285 | PDFSTATSENSEMBLUCSC | 130 | 4 | 32161 | tss | 0.19271 | 0.02691 | Not queried | 1.93 | 4.86e-01 | 1.00e+00 |
|  | 1 | 18972919 | 18973303 | PDFSTATSENSEMBLUCSC | 385 | 5 | 274 | gene;island | 0.35787 | 0.05496 | Not queried | 1.93 | 3.17e-02 | 1.00e+00 |
|  | 8 | 28748202 | 28748679 | PDFSTATSENSEMBLUCSC | 478 | 5 | 39203 | gene;tss;island | 0.04385 | 0.12405 | Not queried | 1.93 | 3.10e-01 | 1.00e+00 |
|  | 4 | 16362340 | 16362666 | PDFSTATSENSEMBLUCSC | 327 | 5 | 30892 | island | 0.64433 | 0.87343 | Not queried | 1.93 | 9.52e-02 | 1.00e+00 |
|  | 11 | 7041752 | 7041987 | PDFSTATSENSEMBLUCSC | 236 | 6 | 6477 | gene | 0.04852 | 0.02087 | Not queried | 1.93 | 4.11e-02 | 1.00e+00 |
|  | 17 | 80189962 | 80190136 | PDFSTATSENSEMBLUCSC | 175 | 6 | 17737 | tss | 0.27368 | 0.55145 | Not queried | 1.93 | 2.16e-03 | 1.00e+00 |
|  | 3 | 49727025 | 49727474 | PDFSTATSENSEMBLUCSC | 450 | 5 | 27745 | gene | 0.02374 | 0.10392 | Not queried | 1.93 | 1.51e-01 | 1.00e+00 |
|  | X | 147133598 | 147133830 | PDFSTATSENSEMBLUCSC | 233 | 4 | 42087 | island | 0.37411 | 0.67283 | Not queried | 1.93 | 2.86e-02 | 1.00e+00 |
|  | 8 | 102504447 | 102504666 | PDFSTATSENSEMBLUCSC | 220 | 7 | 39786 | tss | 0.02110 | 0.13104 | Not queried | 1.93 | 7.28e-02 | 1.00e+00 |
|  | 8 | 97345492 | 97346037 | PDFSTATSENSEMBLUCSC | 546 | 6 | 39305 | gene | 0.51341 | 0.73744 | Not queried | 1.93 | 2.40e-01 | 1.00e+00 |
|  | 12 | 133656595 | 133656953 | PDFSTATSENSEMBLUCSC | 359 | 7 | 10112 | tss;island | 0.02469 | 0.02974 | Not queried | 1.93 | 1.28e-01 | 1.00e+00 |
|  | X | 38080219 | 38080608 | PDFSTATSENSEMBLUCSC | 390 | 7 | 41256 | tss | 0.03769 | 0.04460 | Not queried | 1.93 | 6.20e-01 | 1.00e+00 |
|  | 5 | 1201359 | 1201713 | PDFSTATSENSEMBLUCSC | 355 | 10 | 32553 | island | 0.47858 | 0.79186 | Not queried | 1.93 | 1.08e-05 | 5.56e-02 |
|  | 17 | 80189962 | 80190154 | PDFSTATSENSEMBLUCSC | 193 | 7 | 16907 | gene;island | 0.27570 | 0.58624 | Not queried | 1.93 | 5.83e-04 | 1.00e+00 |
|  | 3 | 141087187 | 141087363 | PDFSTATSENSEMBLUCSC | 177 | 5 | 27931 | gene | 0.57704 | 0.85231 | Not queried | 1.93 | 5.56e-02 | 1.00e+00 |
|  | 11 | 68609518 | 68609747 | PDFSTATSENSEMBLUCSC | 230 | 6 | 7491 | tss;island | 0.08997 | 0.02159 | Not queried | 1.92 | 2.40e-01 | 1.00e+00 |
|  | 6 | 111279493 | 111279703 | PDFSTATSENSEMBLUCSC | 211 | 9 | 35109 | tss | 0.14355 | 0.04043 | Not queried | 1.92 | 1.88e-02 | 1.00e+00 |
|  | 20 | 45313109 | 45313390 | PDFSTATSENSEMBLUCSC | 282 | 6 | 25402 | gene;tss | 0.51339 | 0.80125 | Not queried | 1.92 | 2.60e-02 | 1.00e+00 |
|  | 4 | 88343439 | 88343678 | PDFSTATSENSEMBLUCSC | 240 | 4 | 30441 | tss | 0.12825 | 0.04558 | Not queried | 1.92 | 4.86e-01 | 1.00e+00 |
|  | 17 | 73055792 | 73056187 | PDFSTATSENSEMBLUCSC | 396 | 6 | 16727 | gene | 0.72301 | 0.92590 | Not queried | 1.92 | 9.31e-02 | 1.00e+00 |
|  | 10 | 131989161 | 131989849 | PDFSTATSENSEMBLUCSC | 689 | 8 | 6255 | island | 0.89697 | 0.63009 | Not queried | 1.92 | 4.99e-02 | 1.00e+00 |
|  | 6 | 150039400 | 150039767 | PDFSTATSENSEMBLUCSC | 368 | 8 | 35222 | tss | 0.01368 | 0.11429 | Not queried | 1.92 | 1.86e-03 | 1.00e+00 |
|  | 4 | 987108 | 987672 | PDFSTATSENSEMBLUCSC | 565 | 6 | 29810 | gene;island | 0.75424 | 0.88043 | Not queried | 1.92 | 4.33e-03 | 1.00e+00 |
|  | 3 | 170303540 | 170303830 | PDFSTATSENSEMBLUCSC | 291 | 4 | 27987 | gene | 0.25966 | 0.09127 | Not queried | 1.92 | 1.14e-01 | 1.00e+00 |
|  | 19 | 49939549 | 49940062 | PDFSTATSENSEMBLUCSC | 514 | 6 | 20113 | gene;island | 0.72171 | 0.23482 | Not queried | 1.92 | 4.11e-02 | 1.00e+00 |
|  | 19 | 57337294 | 57337648 | PDFSTATSENSEMBLUCSC | 355 | 4 | 20202 | gene;tss | 0.86116 | 0.94385 | Not queried | 1.92 | 2.86e-02 | 1.00e+00 |
|  | 1 | 156390102 | 156390240 | PDFSTATSENSEMBLUCSC | 139 | 5 | 821 | gene;island | 0.20470 | 0.50573 | Not queried | 1.92 | 5.56e-02 | 1.00e+00 |
|  | 19 | 43969650 | 43970034 | PDFSTATSENSEMBLUCSC | 385 | 6 | 22048 | island | 0.14736 | 0.07838 | Not queried | 1.92 | 6.49e-02 | 1.00e+00 |
|  | X | 130192035 | 130192160 | PDFSTATSENSEMBLUCSC | 126 | 5 | 41521 | tss | 0.24381 | 0.08078 | Not queried | 1.92 | 5.56e-02 | 1.00e+00 |
|  | 17 | 7555132 | 7555411 | PDFSTATSENSEMBLUCSC | 280 | 4 | 16372 | gene;island | 0.24101 | 0.07128 | Not queried | 1.92 | 2.00e-01 | 1.00e+00 |
|  | 17 | 1665119 | 1665220 | PDFSTATSENSEMBLUCSC | 102 | 5 | 16963 | tss | 0.33131 | 0.66283 | Not queried | 1.92 | 7.94e-03 | 1.00e+00 |
|  | 1 | 2776972 | 2777244 | PDFSTATSENSEMBLUCSC | 273 | 4 | 2514 | island | 0.49265 | 0.74555 | Not queried | 1.92 | 2.86e-02 | 1.00e+00 |
|  | 2 | 85839322 | 85839722 | PDFSTATSENSEMBLUCSC | 401 | 5 | 23448 | tss | 0.33280 | 0.11288 | Not queried | 1.92 | 3.10e-01 | 1.00e+00 |
|  | 12 | 23998921 | 23999045 | PDFSTATSENSEMBLUCSC | 125 | 4 | 9057 | gene | 0.59642 | 0.86156 | Not queried | 1.92 | 2.00e-01 | 1.00e+00 |
|  | 20 | 31261784 | 31261994 | PDFSTATSENSEMBLUCSC | 211 | 4 | 25636 | tss | 0.20742 | 0.49632 | Not queried | 1.92 | 1.14e-01 | 1.00e+00 |
|  | 2 | 158453600 | 158454401 | PDFSTATSENSEMBLUCSC | 802 | 9 | 22866 | gene;island | 0.03077 | 0.16180 | Not queried | 1.92 | 2.44e-02 | 1.00e+00 |
|  | 7 | 645309 | 645796 | PDFSTATSENSEMBLUCSC | 488 | 6 | 36653 | gene;island | 0.88136 | 0.94743 | Not queried | 1.91 | 4.11e-02 | 1.00e+00 |
|  | 17 | 35290307 | 35291580 | PDFSTATSENSEMBLUCSC | 1274 | 12 | 18202 | island | 0.54870 | 0.29256 | Not queried | 1.91 | 3.64e-03 | 1.00e+00 |
|  | 6 | 74160917 | 74161222 | PDFSTATSENSEMBLUCSC | 306 | 4 | 34324 | gene;island | 0.17926 | 0.39586 | Not queried | 1.91 | 2.00e-01 | 1.00e+00 |
|  | 7 | 151330008 | 151330230 | PDFSTATSENSEMBLUCSC | 223 | 4 | 37232 | gene;tss | 0.38233 | 0.16719 | Not queried | 1.91 | 2.86e-02 | 1.00e+00 |
|  | 5 | 78407418 | 78407566 | PDFSTATSENSEMBLUCSC | 149 | 6 | 32086 | tss | 0.11702 | 0.28443 | Not queried | 1.91 | 1.32e-01 | 1.00e+00 |
|  | 1 | 26394201 | 26394593 | PDFSTATSENSEMBLUCSC | 393 | 5 | 1378 | tss | 0.92953 | 0.66679 | Not queried | 1.91 | 9.52e-02 | 1.00e+00 |
|  | 1 | 6341140 | 6341327 | PDFSTATSENSEMBLUCSC | 188 | 4 | 166 | gene;island | 0.76179 | 0.89144 | Not queried | 1.91 | 1.14e-01 | 1.00e+00 |
|  | 11 | 69285633 | 69286352 | PDFSTATSENSEMBLUCSC | 720 | 8 | 8567 | island | 0.54408 | 0.82148 | Not queried | 1.91 | 1.04e-02 | 1.00e+00 |
|  | 5 | 161494274 | 161494645 | PDFSTATSENSEMBLUCSC | 372 | 6 | 32414 | tss | 0.24908 | 0.50389 | Not queried | 1.91 | 2.16e-03 | 1.00e+00 |
|  | 12 | 14996508 | 14996776 | PDFSTATSENSEMBLUCSC | 269 | 8 | 9563 | tss | 0.63979 | 0.87597 | Not queried | 1.91 | 6.99e-03 | 1.00e+00 |
|  | 7 | 155174991 | 155175340 | PDFSTATSENSEMBLUCSC | 350 | 4 | 39003 | island | 0.95622 | 0.86157 | Not queried | 1.91 | 2.00e-01 | 1.00e+00 |
|  | 17 | 77924180 | 77924371 | PDFSTATSENSEMBLUCSC | 192 | 4 | 16811 | gene;island | 0.39059 | 0.16480 | Not queried | 1.91 | 2.00e-01 | 1.00e+00 |
|  | 17 | 46651722 | 46651952 | PDFSTATSENSEMBLUCSC | 231 | 7 | 18445 | island | 0.38312 | 0.14196 | Not queried | 1.91 | 7.28e-02 | 1.00e+00 |
|  | 10 | 119786 | 120151 | PDFSTATSENSEMBLUCSC | 366 | 4 | 5449 | island | 0.01911 | 0.10737 | Not queried | 1.91 | 1.14e-01 | 1.00e+00 |
|  | 10 | 125853676 | 125853893 | PDFSTATSENSEMBLUCSC | 218 | 4 | 6206 | island | 0.09608 | 0.20169 | Not queried | 1.91 | 3.43e-01 | 1.00e+00 |
|  | 1 | 47082037 | 47082211 | PDFSTATSENSEMBLUCSC | 175 | 4 | 1599 | tss | 0.21611 | 0.06250 | Not queried | 1.91 | 2.00e-01 | 1.00e+00 |
|  | 21 | 43299789 | 43300024 | PDFSTATSENSEMBLUCSC | 236 | 4 | 26520 | tss | 0.09661 | 0.03074 | Not queried | 1.91 | 3.43e-01 | 1.00e+00 |
|  | 7 | 45808644 | 45808808 | PDFSTATSENSEMBLUCSC | 165 | 5 | 37520 | tss | 0.03806 | 0.06695 | Not queried | 1.91 | 2.22e-01 | 1.00e+00 |
|  | 6 | 30231639 | 30232015 | PDFSTATSENSEMBLUCSC | 377 | 5 | 33692 | gene;island | 0.29366 | 0.64839 | Not queried | 1.91 | 3.17e-02 | 1.00e+00 |
|  | X | 129245188 | 129245245 | PDFSTATSENSEMBLUCSC | 58 | 4 | 41515 | tss;island | 0.34628 | 0.07547 | Not queried | 1.90 | 1.14e-01 | 1.00e+00 |
|  | 12 | 75601711 | 75601928 | PDFSTATSENSEMBLUCSC | 218 | 4 | 9194 | gene;island | 0.10415 | 0.33561 | Not queried | 1.90 | 2.00e-01 | 1.00e+00 |
|  | 18 | 24130267 | 24130633 | PDFSTATSENSEMBLUCSC | 367 | 4 | 19048 | gene;tss;island | 0.22822 | 0.08642 | Not queried | 1.90 | 2.00e-01 | 1.00e+00 |
|  | 17 | 78078605 | 78079086 | PDFSTATSENSEMBLUCSC | 482 | 5 | 16820 | gene;island | 0.61216 | 0.90649 | Not queried | 1.90 | 1.59e-02 | 1.00e+00 |
|  | 3 | 158390329 | 158390821 | PDFSTATSENSEMBLUCSC | 493 | 10 | 27969 | gene | 0.06823 | 0.28286 | Not queried | 1.90 | 2.80e-01 | 1.00e+00 |
|  | 2 | 162283434 | 162284206 | PDFSTATSENSEMBLUCSC | 773 | 8 | 24785 | island | 0.66951 | 0.90033 | Not queried | 1.90 | 6.50e-02 | 1.00e+00 |
|  | 2 | 124782117 | 124782263 | PDFSTATSENSEMBLUCSC | 147 | 4 | 23568 | tss;island | 0.05178 | 0.18672 | Not queried | 1.90 | 4.86e-01 | 1.00e+00 |
|  | 17 | 7757933 | 7758291 | PDFSTATSENSEMBLUCSC | 359 | 10 | 17066 | tss | 0.55735 | 0.89390 | Not queried | 1.90 | 8.93e-03 | 1.00e+00 |
|  | 3 | 111717534 | 111717969 | PDFSTATSENSEMBLUCSC | 436 | 7 | 28455 | tss | 0.04922 | 0.02380 | Not queried | 1.90 | 7.28e-02 | 1.00e+00 |
|  | 17 | 66194137 | 66194797 | PDFSTATSENSEMBLUCSC | 661 | 9 | 17570 | tss | 0.07669 | 0.24742 | Not queried | 1.90 | 7.70e-02 | 1.00e+00 |
|  | 16 | 3142645 | 3143250 | PDFSTATSENSEMBLUCSC | 606 | 8 | 15605 | island | 0.77878 | 0.87833 | Not queried | 1.90 | 1.30e-01 | 1.00e+00 |
|  | 6 | 32407289 | 32407570 | PDFSTATSENSEMBLUCSC | 282 | 4 | 34808 | tss | 0.63825 | 0.22816 | Not queried | 1.90 | 4.86e-01 | 1.00e+00 |
|  | X | 152759883 | 152760248 | PDFSTATSENSEMBLUCSC | 366 | 8 | 41579 | tss | 0.20379 | 0.53908 | Not queried | 1.90 | 1.48e-02 | 1.00e+00 |
|  | 19 | 13134771 | 13134830 | PDFSTATSENSEMBLUCSC | 60 | 4 | 19840 | gene;island | 0.11707 | 0.27571 | Not queried | 1.90 | 2.00e-01 | 1.00e+00 |
|  | 17 | 46671131 | 46671635 | PDFSTATSENSEMBLUCSC | 505 | 9 | 17431 | tss | 0.01666 | 0.05419 | Not queried | 1.90 | 9.39e-02 | 1.00e+00 |
|  | 10 | 3827595 | 3827878 | PDFSTATSENSEMBLUCSC | 284 | 9 | 4903 | tss;island | 0.01964 | 0.07357 | Not queried | 1.90 | 2.88e-04 | 1.00e+00 |
|  | 7 | 141250684 | 141251045 | PDFSTATSENSEMBLUCSC | 362 | 6 | 37880 | tss | 0.11674 | 0.03477 | Not queried | 1.90 | 1.32e-01 | 1.00e+00 |
|  | 16 | 21170927 | 21171239 | PDFSTATSENSEMBLUCSC | 313 | 5 | 14658 | gene;tss;island | 0.08808 | 0.02523 | Not queried | 1.90 | 3.10e-01 | 1.00e+00 |
|  | 1 | 161059429 | 161059654 | PDFSTATSENSEMBLUCSC | 226 | 4 | 2040 | tss | 0.37066 | 0.64466 | Not queried | 1.90 | 2.86e-02 | 1.00e+00 |
|  | 7 | 120577 | 120801 | PDFSTATSENSEMBLUCSC | 225 | 5 | 37964 | island | 0.72452 | 0.78826 | Not queried | 1.90 | 3.10e-01 | 1.00e+00 |
|  | 17 | 7165831 | 7165909 | PDFSTATSENSEMBLUCSC | 79 | 6 | 17036 | tss;island | 0.01251 | 0.04905 | Not queried | 1.89 | 6.49e-02 | 1.00e+00 |
|  | 2 | 71017846 | 71018354 | PDFSTATSENSEMBLUCSC | 509 | 5 | 23383 | tss;island | 0.09107 | 0.22974 | Not queried | 1.89 | 6.90e-01 | 1.00e+00 |
|  | 6 | 33374613 | 33374667 | PDFSTATSENSEMBLUCSC | 55 | 5 | 34211 | gene;island | 0.78280 | 0.94096 | Not queried | 1.89 | 3.17e-02 | 1.00e+00 |
|  | 16 | 66599843 | 66600311 | PDFSTATSENSEMBLUCSC | 469 | 9 | 15250 | tss | 0.83775 | 0.96467 | Not queried | 1.89 | 3.99e-03 | 1.00e+00 |
|  | 16 | 66599843 | 66600700 | PDFSTATSENSEMBLUCSC | 858 | 13 | 15956 | island | 0.83775 | 0.96467 | Not queried | 1.89 | 2.87e-03 | 1.00e+00 |
|  | 6 | 30652647 | 30652907 | PDFSTATSENSEMBLUCSC | 261 | 5 | 33740 | gene;island | 0.33186 | 0.61607 | Not queried | 1.89 | 7.94e-03 | 1.00e+00 |
|  | 10 | 56561096 | 56561213 | PDFSTATSENSEMBLUCSC | 118 | 4 | 5070 | tss | 0.04589 | 0.14218 | Not queried | 1.89 | 2.86e-02 | 1.00e+00 |
|  | X | 51636562 | 51636726 | PDFSTATSENSEMBLUCSC | 165 | 7 | 40996 | gene;island | 0.11935 | 0.02358 | Not queried | 1.89 | 9.73e-02 | 1.00e+00 |
|  | X | 57022079 | 57022192 | PDFSTATSENSEMBLUCSC | 114 | 4 | 41346 | tss;island | 0.20708 | 0.40264 | Not queried | 1.89 | 2.86e-02 | 1.00e+00 |
|  | 16 | 29625133 | 29625259 | PDFSTATSENSEMBLUCSC | 127 | 5 | 15135 | tss;island | 0.09745 | 0.04028 | Not queried | 1.89 | 4.21e-01 | 1.00e+00 |
|  | 17 | 72848048 | 72848669 | PDFSTATSENSEMBLUCSC | 622 | 5 | 16726 | gene;island | 0.12185 | 0.31066 | Not queried | 1.89 | 3.10e-01 | 1.00e+00 |
|  | 4 | 571371 | 571737 | PDFSTATSENSEMBLUCSC | 367 | 4 | 30673 | island | 0.24344 | 0.09221 | Not queried | 1.89 | 5.71e-02 | 1.00e+00 |
|  | 6 | 136172553 | 136172818 | PDFSTATSENSEMBLUCSC | 266 | 5 | 35175 | tss | 0.05485 | 0.14298 | Not queried | 1.89 | 2.22e-01 | 1.00e+00 |
|  | 20 | 31331814 | 31332353 | PDFSTATSENSEMBLUCSC | 540 | 6 | 25637 | tss;island | 0.29117 | 0.08752 | Not queried | 1.89 | 1.80e-01 | 1.00e+00 |
|  | 17 | 61042718 | 61043072 | PDFSTATSENSEMBLUCSC | 355 | 4 | 18605 | island | 0.41115 | 0.29119 | Not queried | 1.89 | 6.86e-01 | 1.00e+00 |
|  | X | 55187242 | 55188047 | PDFSTATSENSEMBLUCSC | 806 | 14 | 41819 | island | 0.07562 | 0.02426 | Not queried | 1.89 | 1.86e-02 | 1.00e+00 |
|  | 11 | 44972574 | 44973218 | PDFSTATSENSEMBLUCSC | 645 | 11 | 8207 | island | 0.63126 | 0.40880 | Not queried | 1.89 | 3.36e-02 | 1.00e+00 |
|  | 12 | 12871752 | 12871924 | PDFSTATSENSEMBLUCSC | 173 | 4 | 9034 | gene;island | 0.03739 | 0.14791 | Not queried | 1.89 | 5.71e-02 | 1.00e+00 |
|  | X | 43515213 | 43515377 | PDFSTATSENSEMBLUCSC | 165 | 4 | 41266 | tss | 0.17814 | 0.05037 | Not queried | 1.89 | 2.00e-01 | 1.00e+00 |
|  | 3 | 49726775 | 49727474 | PDFSTATSENSEMBLUCSC | 700 | 12 | 28290 | tss;island | 0.02333 | 0.08562 | Not queried | 1.89 | 1.45e-02 | 1.00e+00 |
|  | 12 | 127212034 | 127212528 | PDFSTATSENSEMBLUCSC | 495 | 4 | 10956 | island | 0.15273 | 0.47122 | Not queried | 1.89 | 2.86e-02 | 1.00e+00 |
|  | X | 69501934 | 69502066 | PDFSTATSENSEMBLUCSC | 133 | 6 | 41366 | tss | 0.62500 | 0.83715 | Not queried | 1.89 | 9.31e-02 | 1.00e+00 |
|  | 6 | 33160067 | 33160234 | PDFSTATSENSEMBLUCSC | 168 | 6 | 34151 | gene | 0.15514 | 0.38908 | Not queried | 1.89 | 2.60e-02 | 1.00e+00 |
|  | 12 | 132851783 | 132852053 | PDFSTATSENSEMBLUCSC | 271 | 4 | 9415 | gene;island | 0.38295 | 0.72109 | Not queried | 1.89 | 2.86e-02 | 1.00e+00 |
|  | 10 | 97515222 | 97515398 | PDFSTATSENSEMBLUCSC | 177 | 4 | 4675 | gene;tss | 0.19453 | 0.06424 | Not queried | 1.89 | 5.71e-02 | 1.00e+00 |
|  | 17 | 3558202 | 3558426 | PDFSTATSENSEMBLUCSC | 225 | 4 | 16306 | gene | 0.83737 | 0.93726 | Not queried | 1.89 | 3.43e-01 | 1.00e+00 |
|  | 16 | 56965376 | 56965658 | PDFSTATSENSEMBLUCSC | 283 | 4 | 15224 | tss | 0.25205 | 0.52558 | Not queried | 1.88 | 2.00e-01 | 1.00e+00 |
|  | 2 | 24398427 | 24398859 | PDFSTATSENSEMBLUCSC | 433 | 5 | 22561 | gene;island | 0.62745 | 0.87479 | Not queried | 1.88 | 9.52e-02 | 1.00e+00 |
|  | 3 | 183993368 | 183994242 | PDFSTATSENSEMBLUCSC | 875 | 11 | 28017 | gene;island | 0.50113 | 0.76901 | Not queried | 1.88 | 1.92e-02 | 1.00e+00 |
|  | 8 | 81397601 | 81397876 | PDFSTATSENSEMBLUCSC | 276 | 5 | 39718 | tss;island | 0.01026 | 0.02188 | Not queried | 1.88 | 9.52e-02 | 1.00e+00 |
|  | 1 | 45792540 | 45792733 | PDFSTATSENSEMBLUCSC | 194 | 7 | 3192 | island | 0.52352 | 0.83761 | Not queried | 1.88 | 6.99e-03 | 1.00e+00 |
|  | 5 | 153852554 | 153853283 | PDFSTATSENSEMBLUCSC | 730 | 8 | 33265 | island | 0.81352 | 0.51010 | Not queried | 1.88 | 6.99e-03 | 1.00e+00 |
|  | 8 | 25905478 | 25905811 | PDFSTATSENSEMBLUCSC | 334 | 4 | 40106 | island | 0.07209 | 0.17252 | Not queried | 1.88 | 6.86e-01 | 1.00e+00 |
|  | 7 | 136553088 | 136553327 | PDFSTATSENSEMBLUCSC | 240 | 7 | 37857 | tss;island | 0.23269 | 0.28023 | Not queried | 1.88 | 3.18e-01 | 1.00e+00 |
|  | 12 | 53183773 | 53184086 | PDFSTATSENSEMBLUCSC | 314 | 5 | 9115 | gene;island | 0.73324 | 0.90604 | Not queried | 1.88 | 9.52e-02 | 1.00e+00 |
|  | 6 | 76059450 | 76059773 | PDFSTATSENSEMBLUCSC | 324 | 6 | 34331 | gene | 0.07737 | 0.02934 | Not queried | 1.88 | 4.33e-03 | 1.00e+00 |
|  | 22 | 31002757 | 31003147 | PDFSTATSENSEMBLUCSC | 391 | 8 | 27024 | tss | 0.04259 | 0.15118 | Not queried | 1.88 | 1.09e-03 | 1.00e+00 |
|  | 13 | 114880761 | 114881194 | PDFSTATSENSEMBLUCSC | 434 | 6 | 11288 | gene;island | 0.61564 | 0.91299 | Not queried | 1.88 | 8.66e-03 | 1.00e+00 |
|  | 22 | 31536476 | 31536784 | PDFSTATSENSEMBLUCSC | 309 | 5 | 27033 | tss | 0.39771 | 0.15234 | Not queried | 1.88 | 2.22e-01 | 1.00e+00 |
|  | 2 | 1748872 | 1749076 | PDFSTATSENSEMBLUCSC | 205 | 4 | 23120 | tss;island | 0.56957 | 0.30847 | Not queried | 1.88 | 2.86e-02 | 1.00e+00 |
|  | X | 15755989 | 15756062 | PDFSTATSENSEMBLUCSC | 74 | 4 | 41201 | tss;island | 0.02360 | 0.06996 | Not queried | 1.88 | 5.71e-02 | 1.00e+00 |
|  | 15 | 29499226 | 29499526 | PDFSTATSENSEMBLUCSC | 301 | 5 | 13241 | gene | 0.68870 | 0.91403 | Not queried | 1.88 | 3.17e-02 | 1.00e+00 |
|  | 12 | 113917395 | 113917737 | PDFSTATSENSEMBLUCSC | 343 | 5 | 10807 | island | 0.03597 | 0.09153 | Not queried | 1.87 | 9.52e-02 | 1.00e+00 |
|  | 1 | 3184434 | 3184704 | PDFSTATSENSEMBLUCSC | 271 | 4 | 114 | gene;island | 0.61311 | 0.86318 | Not queried | 1.87 | 2.86e-02 | 1.00e+00 |
|  | 6 | 31276088 | 31276212 | PDFSTATSENSEMBLUCSC | 125 | 5 | 35701 | island | 0.03262 | 0.13569 | Not queried | 1.87 | 1.59e-02 | 1.00e+00 |
|  | 2 | 242808427 | 242808907 | PDFSTATSENSEMBLUCSC | 481 | 5 | 25274 | island | 0.48093 | 0.81286 | Not queried | 1.87 | 7.94e-03 | 1.00e+00 |
|  | 1 | 230777685 | 230777875 | PDFSTATSENSEMBLUCSC | 191 | 4 | 2312 | tss;island | 0.04079 | 0.02136 | Not queried | 1.87 | 2.86e-02 | 1.00e+00 |
|  | 19 | 10426722 | 10426933 | PDFSTATSENSEMBLUCSC | 212 | 4 | 20409 | tss;island | 0.13407 | 0.31237 | Not queried | 1.87 | 4.86e-01 | 1.00e+00 |
|  | 10 | 729479 | 729956 | PDFSTATSENSEMBLUCSC | 478 | 6 | 4438 | gene | 0.93566 | 0.81644 | Not queried | 1.87 | 8.66e-03 | 1.00e+00 |
|  | 3 | 27764473 | 27764816 | PDFSTATSENSEMBLUCSC | 344 | 4 | 28154 | tss;island | 0.45840 | 0.18801 | Not queried | 1.87 | 5.71e-02 | 1.00e+00 |
|  | 17 | 44896748 | 44897024 | PDFSTATSENSEMBLUCSC | 277 | 4 | 17403 | tss;island | 0.11554 | 0.02840 | Not queried | 1.87 | 2.86e-02 | 1.00e+00 |
|  | 4 | 1595294 | 1595726 | PDFSTATSENSEMBLUCSC | 433 | 5 | 30745 | island | 0.81425 | 0.96282 | Not queried | 1.87 | 3.17e-02 | 1.00e+00 |
|  | 11 | 1036677 | 1036938 | PDFSTATSENSEMBLUCSC | 262 | 8 | 7876 | island | 0.65043 | 0.85649 | Not queried | 1.87 | 1.55e-04 | 7.99e-01 |
|  | 14 | 88788343 | 88788672 | PDFSTATSENSEMBLUCSC | 330 | 6 | 12137 | gene;island | 0.16806 | 0.26250 | Not queried | 1.87 | 2.40e-01 | 1.00e+00 |
|  | 6 | 32809263 | 32809657 | PDFSTATSENSEMBLUCSC | 395 | 10 | 34083 | gene;island | 0.84434 | 0.66111 | Not queried | 1.87 | 5.20e-03 | 1.00e+00 |
|  | 3 | 148804272 | 148805294 | PDFSTATSENSEMBLUCSC | 1023 | 23 | 29536 | island | 0.93849 | 0.49347 | Not queried | 1.87 | 3.22e-02 | 1.00e+00 |
|  | 6 | 1515524 | 1515797 | PDFSTATSENSEMBLUCSC | 274 | 4 | 35304 | island | 0.48561 | 0.79770 | Not queried | 1.87 | 2.86e-02 | 1.00e+00 |
|  | 20 | 1165065 | 1165435 | PDFSTATSENSEMBLUCSC | 371 | 5 | 25893 | island | 0.79794 | 0.51992 | Not queried | 1.87 | 9.52e-02 | 1.00e+00 |
|  | 5 | 1201359 | 1201698 | PDFSTATSENSEMBLUCSC | 340 | 9 | 31908 | tss | 0.46545 | 0.77147 | Not queried | 1.87 | 4.11e-05 | 2.11e-01 |
|  | 2 | 240302585 | 240302855 | PDFSTATSENSEMBLUCSC | 271 | 4 | 23081 | gene | 0.61202 | 0.88583 | Not queried | 1.87 | 5.71e-02 | 1.00e+00 |
|  | 5 | 122971759 | 122971951 | PDFSTATSENSEMBLUCSC | 193 | 4 | 32996 | island | 0.64553 | 0.92035 | Not queried | 1.87 | 1.14e-01 | 1.00e+00 |
|  | 19 | 17487776 | 17488445 | PDFSTATSENSEMBLUCSC | 670 | 8 | 21704 | island | 0.69180 | 0.90016 | Not queried | 1.86 | 1.04e-02 | 1.00e+00 |
|  | 11 | 1769152 | 1769645 | PDFSTATSENSEMBLUCSC | 494 | 9 | 6412 | gene;island | 0.71395 | 0.92663 | Not queried | 1.86 | 1.85e-03 | 1.00e+00 |
|  | 11 | 1769382 | 1769645 | PDFSTATSENSEMBLUCSC | 264 | 7 | 7024 | tss | 0.71395 | 0.92663 | Not queried | 1.86 | 6.99e-03 | 1.00e+00 |
|  | 1 | 51810823 | 51811185 | PDFSTATSENSEMBLUCSC | 363 | 5 | 1619 | tss | 0.32184 | 0.63338 | Not queried | 1.86 | 4.21e-01 | 1.00e+00 |
|  | 5 | 172664995 | 172665149 | PDFSTATSENSEMBLUCSC | 155 | 4 | 33344 | island | 0.48385 | 0.79606 | Not queried | 1.86 | 2.86e-02 | 1.00e+00 |
|  | X | 22290866 | 22290942 | PDFSTATSENSEMBLUCSC | 77 | 5 | 41224 | tss | 0.79643 | 0.95185 | Not queried | 1.86 | 7.94e-03 | 1.00e+00 |
|  | X | 55187242 | 55187570 | PDFSTATSENSEMBLUCSC | 329 | 8 | 41001 | gene | 0.05755 | 0.01438 | Not queried | 1.86 | 1.05e-01 | 1.00e+00 |
|  | 13 | 113655468 | 113655622 | PDFSTATSENSEMBLUCSC | 155 | 4 | 11238 | gene;island | 0.51222 | 0.79176 | Not queried | 1.86 | 2.00e-01 | 1.00e+00 |
|  | 9 | 2717146 | 2717621 | PDFSTATSENSEMBLUCSC | 476 | 5 | 40797 | island | 0.80136 | 0.95176 | Not queried | 1.86 | 3.17e-02 | 1.00e+00 |
|  | 1 | 2063799 | 2064216 | PDFSTATSENSEMBLUCSC | 418 | 5 | 63 | gene;island | 0.25811 | 0.56897 | Not queried | 1.86 | 9.52e-02 | 1.00e+00 |
|  | 2 | 171784945 | 171785124 | PDFSTATSENSEMBLUCSC | 180 | 4 | 23672 | tss;island | 0.75592 | 0.92667 | Not queried | 1.86 | 3.43e-01 | 1.00e+00 |
|  | 10 | 94459183 | 94459665 | PDFSTATSENSEMBLUCSC | 483 | 6 | 5928 | island | 0.06858 | 0.03556 | Not queried | 1.86 | 9.31e-02 | 1.00e+00 |
|  | 16 | 898803 | 899108 | PDFSTATSENSEMBLUCSC | 306 | 4 | 15463 | island | 0.43605 | 0.77409 | Not queried | 1.86 | 2.00e-01 | 1.00e+00 |
|  | 11 | 103908255 | 103908576 | PDFSTATSENSEMBLUCSC | 322 | 4 | 6860 | gene;island | 0.64277 | 0.83830 | Not queried | 1.86 | 1.14e-01 | 1.00e+00 |
|  | 12 | 10183167 | 10183267 | PDFSTATSENSEMBLUCSC | 101 | 6 | 9534 | tss | 0.84528 | 0.94870 | Not queried | 1.86 | 2.60e-02 | 1.00e+00 |
|  | 19 | 1248287 | 1248689 | PDFSTATSENSEMBLUCSC | 403 | 6 | 21169 | island | 0.26325 | 0.07483 | Not queried | 1.86 | 3.10e-01 | 1.00e+00 |
|  | 1 | 3059517 | 3059820 | PDFSTATSENSEMBLUCSC | 304 | 4 | 103 | gene;island | 0.77205 | 0.92519 | Not queried | 1.86 | 1.14e-01 | 1.00e+00 |
|  | 1 | 184005360 | 184005990 | PDFSTATSENSEMBLUCSC | 631 | 5 | 917 | gene;island | 0.34637 | 0.11877 | Not queried | 1.85 | 2.22e-01 | 1.00e+00 |
|  | 3 | 64670459 | 64670640 | PDFSTATSENSEMBLUCSC | 182 | 4 | 27808 | gene;island | 0.40162 | 0.16182 | Not queried | 1.85 | 4.86e-01 | 1.00e+00 |
|  | 17 | 37754160 | 37754424 | PDFSTATSENSEMBLUCSC | 265 | 4 | 18244 | island | 0.14055 | 0.44284 | Not queried | 1.85 | 3.43e-01 | 1.00e+00 |
|  | 4 | 1008425 | 1008738 | PDFSTATSENSEMBLUCSC | 314 | 4 | 29813 | gene;island | 0.44055 | 0.75819 | Not queried | 1.85 | 5.71e-02 | 1.00e+00 |
|  | 13 | 114760892 | 114761326 | PDFSTATSENSEMBLUCSC | 435 | 4 | 11274 | gene;island | 0.32964 | 0.74971 | Not queried | 1.85 | 5.71e-02 | 1.00e+00 |
|  | 2 | 242930039 | 242930747 | PDFSTATSENSEMBLUCSC | 709 | 7 | 25281 | island | 0.66742 | 0.88194 | Not queried | 1.85 | 2.62e-02 | 1.00e+00 |
|  | 11 | 130298172 | 130298880 | PDFSTATSENSEMBLUCSC | 709 | 11 | 8937 | island | 0.15684 | 0.05040 | Not queried | 1.85 | 1.33e-01 | 1.00e+00 |
|  | 19 | 39989234 | 39989542 | PDFSTATSENSEMBLUCSC | 309 | 5 | 20738 | tss | 0.02656 | 0.04115 | Not queried | 1.85 | 2.22e-01 | 1.00e+00 |
|  | 19 | 39989234 | 39989592 | PDFSTATSENSEMBLUCSC | 359 | 7 | 21965 | island | 0.01644 | 0.03840 | Not queried | 1.85 | 3.18e-01 | 1.00e+00 |
|  | 4 | 141264393 | 141264721 | PDFSTATSENSEMBLUCSC | 329 | 5 | 30089 | gene | 0.81546 | 0.94093 | Not queried | 1.85 | 1.59e-02 | 1.00e+00 |
|  | 7 | 36022276 | 36022841 | PDFSTATSENSEMBLUCSC | 566 | 5 | 38349 | island | 0.64653 | 0.89256 | Not queried | 1.85 | 1.59e-02 | 1.00e+00 |
|  | 1 | 31158026 | 31158299 | PDFSTATSENSEMBLUCSC | 274 | 5 | 2958 | island | 0.03952 | 0.08399 | Not queried | 1.85 | 4.21e-01 | 1.00e+00 |
|  | 3 | 44063315 | 44063832 | PDFSTATSENSEMBLUCSC | 518 | 6 | 28988 | island | 0.17182 | 0.06457 | Not queried | 1.85 | 3.10e-01 | 1.00e+00 |
|  | 5 | 43020161 | 43020716 | PDFSTATSENSEMBLUCSC | 556 | 7 | 32703 | island | 0.02256 | 0.08442 | Not queried | 1.85 | 7.28e-02 | 1.00e+00 |
|  | 17 | 38256932 | 38257323 | PDFSTATSENSEMBLUCSC | 392 | 11 | 18262 | island | 0.05626 | 0.02047 | Not queried | 1.85 | 1.58e-02 | 1.00e+00 |
|  | 12 | 14927294 | 14927542 | PDFSTATSENSEMBLUCSC | 249 | 4 | 9040 | gene | 0.01377 | 0.03186 | Not queried | 1.84 | 2.86e-02 | 1.00e+00 |
|  | 7 | 73241729 | 73242028 | PDFSTATSENSEMBLUCSC | 300 | 4 | 38506 | island | 0.42606 | 0.70545 | Not queried | 1.84 | 5.71e-02 | 1.00e+00 |
|  | 17 | 10541401 | 10541764 | PDFSTATSENSEMBLUCSC | 364 | 4 | 16406 | gene;island | 0.46522 | 0.79982 | Not queried | 1.84 | 3.43e-01 | 1.00e+00 |
|  | 7 | 154863176 | 154863381 | PDFSTATSENSEMBLUCSC | 206 | 4 | 37242 | gene;island | 0.21130 | 0.47021 | Not queried | 1.84 | 2.00e-01 | 1.00e+00 |
|  | 6 | 32825040 | 32825148 | PDFSTATSENSEMBLUCSC | 109 | 6 | 34093 | gene;island | 0.64898 | 0.42807 | Not queried | 1.84 | 4.11e-02 | 1.00e+00 |
|  | 19 | 18496756 | 18496953 | PDFSTATSENSEMBLUCSC | 198 | 4 | 20582 | tss | 0.40226 | 0.70810 | Not queried | 1.84 | 2.86e-02 | 1.00e+00 |
|  | 19 | 18496756 | 18497201 | PDFSTATSENSEMBLUCSC | 446 | 8 | 21743 | island | 0.49199 | 0.73314 | Not queried | 1.84 | 8.30e-02 | 1.00e+00 |
|  | 12 | 2944286 | 2944493 | PDFSTATSENSEMBLUCSC | 208 | 6 | 9466 | tss | 0.45497 | 0.78275 | Not queried | 1.84 | 4.11e-02 | 1.00e+00 |
|  | 3 | 183959465 | 183959853 | PDFSTATSENSEMBLUCSC | 389 | 4 | 28015 | gene;island | 0.40304 | 0.70544 | Not queried | 1.84 | 2.86e-02 | 1.00e+00 |
|  | 12 | 46319990 | 46320110 | PDFSTATSENSEMBLUCSC | 121 | 4 | 9079 | gene | 0.93892 | 0.97722 | Not queried | 1.84 | 2.86e-02 | 1.00e+00 |
|  | 10 | 101282572 | 101282883 | PDFSTATSENSEMBLUCSC | 312 | 5 | 5990 | island | 0.23348 | 0.04274 | Not queried | 1.84 | 1.51e-01 | 1.00e+00 |
|  | 12 | 50297777 | 50297945 | PDFSTATSENSEMBLUCSC | 169 | 4 | 9672 | tss | 0.33715 | 0.62926 | Not queried | 1.84 | 2.86e-02 | 1.00e+00 |
|  | 1 | 22470218 | 22470343 | PDFSTATSENSEMBLUCSC | 126 | 4 | 1327 | tss;island | 0.34103 | 0.11028 | Not queried | 1.84 | 2.00e-01 | 1.00e+00 |
|  | 12 | 22094963 | 22095477 | PDFSTATSENSEMBLUCSC | 515 | 5 | 10256 | island | 0.05735 | 0.05921 | Not queried | 1.83 | 5.48e-01 | 1.00e+00 |
|  | 15 | 84047906 | 84048308 | PDFSTATSENSEMBLUCSC | 403 | 5 | 14371 | island | 0.85506 | 0.59895 | Not queried | 1.83 | 7.94e-03 | 1.00e+00 |
|  | 4 | 80246713 | 80247088 | PDFSTATSENSEMBLUCSC | 376 | 4 | 29987 | gene | 0.52810 | 0.75261 | Not queried | 1.83 | 2.86e-02 | 1.00e+00 |
|  | 19 | 19322527 | 19322778 | PDFSTATSENSEMBLUCSC | 252 | 6 | 20603 | tss | 0.02377 | 0.06554 | Not queried | 1.83 | 3.94e-01 | 1.00e+00 |
|  | 17 | 74524852 | 74525125 | PDFSTATSENSEMBLUCSC | 274 | 4 | 16756 | gene | 0.65697 | 0.90335 | Not queried | 1.83 | 3.43e-01 | 1.00e+00 |
|  | 10 | 26931731 | 26931991 | PDFSTATSENSEMBLUCSC | 261 | 8 | 4978 | tss | 0.52727 | 0.81606 | Not queried | 1.83 | 8.30e-02 | 1.00e+00 |
|  | 17 | 57408935 | 57409010 | PDFSTATSENSEMBLUCSC | 76 | 5 | 17512 | tss | 0.01037 | 0.01578 | Not queried | 1.83 | 9.52e-02 | 1.00e+00 |
|  | 3 | 11034279 | 11034501 | PDFSTATSENSEMBLUCSC | 223 | 5 | 28837 | island | 0.06132 | 0.22320 | Not queried | 1.83 | 9.52e-02 | 1.00e+00 |
|  | 19 | 48202226 | 48202596 | PDFSTATSENSEMBLUCSC | 371 | 4 | 20082 | gene;island | 0.52776 | 0.81643 | Not queried | 1.83 | 2.00e-01 | 1.00e+00 |
|  | 1 | 1293868 | 1294140 | PDFSTATSENSEMBLUCSC | 273 | 4 | 2425 | island | 0.36390 | 0.69770 | Not queried | 1.83 | 2.86e-02 | 1.00e+00 |
|  | 17 | 38375119 | 38375510 | PDFSTATSENSEMBLUCSC | 392 | 7 | 17294 | tss;island | 0.02142 | 0.01226 | Not queried | 1.83 | 2.09e-01 | 1.00e+00 |
|  | 3 | 42947263 | 42947771 | PDFSTATSENSEMBLUCSC | 509 | 7 | 28980 | island | 0.79867 | 0.96023 | Not queried | 1.83 | 1.11e-02 | 1.00e+00 |
|  | 11 | 31846414 | 31846849 | PDFSTATSENSEMBLUCSC | 436 | 6 | 8148 | island | 0.18208 | 0.09122 | Not queried | 1.83 | 3.10e-01 | 1.00e+00 |
|  | 1 | 155232925 | 155233109 | PDFSTATSENSEMBLUCSC | 185 | 4 | 795 | gene;tss;island | 0.72435 | 0.92758 | Not queried | 1.83 | 1.14e-01 | 1.00e+00 |
|  | 1 | 153508755 | 153508875 | PDFSTATSENSEMBLUCSC | 121 | 4 | 1938 | tss | 0.44514 | 0.78364 | Not queried | 1.83 | 2.86e-02 | 1.00e+00 |
|  | 4 | 155662217 | 155662515 | PDFSTATSENSEMBLUCSC | 299 | 4 | 31331 | island | 0.25429 | 0.03574 | Not queried | 1.83 | 3.43e-01 | 1.00e+00 |
|  | 1 | 1190026 | 1190238 | PDFSTATSENSEMBLUCSC | 213 | 4 | 24 | gene;island | 0.83740 | 0.95102 | Not queried | 1.83 | 4.86e-01 | 1.00e+00 |
|  | 10 | 44788627 | 44789077 | PDFSTATSENSEMBLUCSC | 451 | 5 | 5688 | island | 0.57411 | 0.83114 | Not queried | 1.83 | 9.52e-02 | 1.00e+00 |
|  | 6 | 27782909 | 27783226 | PDFSTATSENSEMBLUCSC | 318 | 4 | 33556 | gene | 0.19687 | 0.59028 | Not queried | 1.82 | 1.14e-01 | 1.00e+00 |
|  | 6 | 33386967 | 33387205 | PDFSTATSENSEMBLUCSC | 239 | 6 | 34844 | tss;island | 0.66938 | 0.90471 | Not queried | 1.82 | 4.11e-02 | 1.00e+00 |
|  | 11 | 10562070 | 10562473 | PDFSTATSENSEMBLUCSC | 404 | 4 | 6489 | gene;island | 0.01496 | 0.02984 | Not queried | 1.82 | 2.00e-01 | 1.00e+00 |
|  | 17 | 6735063 | 6735316 | PDFSTATSENSEMBLUCSC | 254 | 8 | 17021 | tss | 0.01875 | 0.06396 | Not queried | 1.82 | 4.66e-03 | 1.00e+00 |
|  | 7 | 94536541 | 94536787 | PDFSTATSENSEMBLUCSC | 247 | 4 | 37657 | tss | 0.84506 | 0.61348 | Not queried | 1.82 | 2.00e-01 | 1.00e+00 |
|  | 7 | 158045980 | 158046358 | PDFSTATSENSEMBLUCSC | 379 | 6 | 37298 | gene | 0.51641 | 0.80731 | Not queried | 1.82 | 9.31e-02 | 1.00e+00 |
|  | 7 | 23530449 | 23530810 | PDFSTATSENSEMBLUCSC | 362 | 4 | 36853 | gene;island | 0.29266 | 0.64858 | Not queried | 1.82 | 5.71e-02 | 1.00e+00 |
|  | 6 | 32798417 | 32798510 | PDFSTATSENSEMBLUCSC | 94 | 4 | 34075 | gene | 0.53421 | 0.23777 | Not queried | 1.82 | 4.86e-01 | 1.00e+00 |
|  | 17 | 80084554 | 80084751 | PDFSTATSENSEMBLUCSC | 198 | 4 | 16906 | gene;island | 0.50780 | 0.85138 | Not queried | 1.82 | 3.43e-01 | 1.00e+00 |
|  | 2 | 85553759 | 85554135 | PDFSTATSENSEMBLUCSC | 377 | 5 | 22724 | gene;island | 0.03647 | 0.15227 | Not queried | 1.82 | 9.52e-02 | 1.00e+00 |
|  | 6 | 33084420 | 33085063 | PDFSTATSENSEMBLUCSC | 644 | 20 | 34123 | gene;island | 0.20694 | 0.42774 | Not queried | 1.82 | 1.77e-04 | 9.07e-01 |
|  | 14 | 29234890 | 29235469 | PDFSTATSENSEMBLUCSC | 580 | 11 | 12307 | tss;island | 0.15844 | 0.05424 | Not queried | 1.82 | 2.75e-04 | 1.00e+00 |
|  | 5 | 42924215 | 42924694 | PDFSTATSENSEMBLUCSC | 480 | 4 | 32698 | island | 0.59849 | 0.85513 | Not queried | 1.82 | 2.00e-01 | 1.00e+00 |
|  | 17 | 7757933 | 7758102 | PDFSTATSENSEMBLUCSC | 170 | 5 | 16381 | gene | 0.62072 | 0.86521 | Not queried | 1.82 | 3.10e-01 | 1.00e+00 |
|  | 13 | 47470793 | 47471090 | PDFSTATSENSEMBLUCSC | 298 | 5 | 11130 | gene | 0.87869 | 0.66106 | Not queried | 1.82 | 2.22e-01 | 1.00e+00 |
|  | 19 | 863054 | 863244 | PDFSTATSENSEMBLUCSC | 191 | 4 | 19600 | gene;island | 0.60935 | 0.32380 | Not queried | 1.81 | 5.71e-02 | 1.00e+00 |
|  | Y | 21728952 | 21729144 | PDFSTATSENSEMBLUCSC | 193 | 6 | 42171 | tss | 0.01799 | 0.11642 | Not queried | 1.81 | 5.89e-01 | 1.00e+00 |
|  | 5 | 140719008 | 140719302 | PDFSTATSENSEMBLUCSC | 295 | 4 | 31760 | gene;island | 0.69207 | 0.91039 | Not queried | 1.81 | 5.71e-02 | 1.00e+00 |
|  | 1 | 16163479 | 16164122 | PDFSTATSENSEMBLUCSC | 644 | 7 | 251 | gene;island | 0.56000 | 0.82138 | Not queried | 1.81 | 1.75e-02 | 1.00e+00 |
|  | 2 | 220361916 | 220362242 | PDFSTATSENSEMBLUCSC | 327 | 6 | 25088 | island | 0.41586 | 0.67391 | Not queried | 1.81 | 2.16e-03 | 1.00e+00 |
|  | 17 | 146404 | 146926 | PDFSTATSENSEMBLUCSC | 523 | 5 | 16250 | gene | 0.58406 | 0.83128 | Not queried | 1.81 | 4.21e-01 | 1.00e+00 |
|  | X | 152954816 | 152954921 | PDFSTATSENSEMBLUCSC | 106 | 6 | 41585 | tss | 0.76112 | 0.58387 | Not queried | 1.81 | 4.33e-03 | 1.00e+00 |
|  | 6 | 31516015 | 31516059 | PDFSTATSENSEMBLUCSC | 45 | 4 | 33836 | gene;tss | 0.79861 | 0.53610 | Not queried | 1.81 | 2.00e-01 | 1.00e+00 |
|  | 16 | 66600294 | 66600700 | PDFSTATSENSEMBLUCSC | 407 | 6 | 14751 | gene | 0.83143 | 0.95158 | Not queried | 1.81 | 9.31e-02 | 1.00e+00 |
|  | 8 | 70983567 | 70983760 | PDFSTATSENSEMBLUCSC | 194 | 4 | 39693 | tss | 0.11723 | 0.04826 | Not queried | 1.81 | 1.14e-01 | 1.00e+00 |
|  | 2 | 176987161 | 176987411 | PDFSTATSENSEMBLUCSC | 251 | 5 | 23699 | tss | 0.00535 | 0.01971 | Not queried | 1.81 | 9.52e-02 | 1.00e+00 |
|  | 2 | 219232590 | 219232875 | PDFSTATSENSEMBLUCSC | 286 | 4 | 25046 | island | 0.49928 | 0.75700 | Not queried | 1.81 | 2.86e-02 | 1.00e+00 |
|  | 7 | 136849018 | 136849317 | PDFSTATSENSEMBLUCSC | 300 | 5 | 38880 | island | 0.50388 | 0.89569 | Not queried | 1.81 | 9.52e-02 | 1.00e+00 |
|  | 1 | 15256054 | 15256222 | PDFSTATSENSEMBLUCSC | 169 | 5 | 239 | gene;tss | 0.54608 | 0.82389 | Not queried | 1.81 | 3.17e-02 | 1.00e+00 |
|  | 10 | 63213104 | 63213510 | PDFSTATSENSEMBLUCSC | 407 | 7 | 5749 | island | 0.04116 | 0.22307 | Not queried | 1.81 | 5.30e-02 | 1.00e+00 |
|  | 12 | 91348999 | 91349273 | PDFSTATSENSEMBLUCSC | 275 | 7 | 9886 | tss;island | 0.77283 | 0.91122 | Not queried | 1.81 | 9.73e-02 | 1.00e+00 |
|  | 5 | 161494711 | 161494810 | PDFSTATSENSEMBLUCSC | 100 | 4 | 31822 | gene | 0.22073 | 0.41766 | Not queried | 1.81 | 2.86e-02 | 1.00e+00 |
|  | 4 | 119948360 | 119948542 | PDFSTATSENSEMBLUCSC | 183 | 4 | 30061 | gene | 0.56808 | 0.82753 | Not queried | 1.81 | 2.00e-01 | 1.00e+00 |
|  | 2 | 40739661 | 40740063 | PDFSTATSENSEMBLUCSC | 403 | 4 | 23280 | tss | 0.20379 | 0.45576 | Not queried | 1.81 | 1.14e-01 | 1.00e+00 |
|  | 5 | 101632286 | 101632559 | PDFSTATSENSEMBLUCSC | 274 | 9 | 32139 | tss | 0.14716 | 0.06779 | Not queried | 1.81 | 6.25e-02 | 1.00e+00 |
|  | 14 | 105041382 | 105041708 | PDFSTATSENSEMBLUCSC | 327 | 5 | 13178 | island | 0.70349 | 0.87454 | Not queried | 1.81 | 1.51e-01 | 1.00e+00 |
|  | 19 | 21950196 | 21950478 | PDFSTATSENSEMBLUCSC | 283 | 6 | 21804 | island | 0.39810 | 0.10549 | Not queried | 1.81 | 6.49e-02 | 1.00e+00 |
|  | 6 | 31847922 | 31848099 | PDFSTATSENSEMBLUCSC | 178 | 4 | 33917 | gene;tss | 0.64971 | 0.89993 | Not queried | 1.81 | 2.86e-02 | 1.00e+00 |
|  | 11 | 22214333 | 22214683 | PDFSTATSENSEMBLUCSC | 351 | 5 | 7181 | tss | 0.05996 | 0.10986 | Not queried | 1.80 | 3.17e-02 | 1.00e+00 |
|  | 3 | 53880717 | 53880872 | PDFSTATSENSEMBLUCSC | 156 | 4 | 27790 | gene;tss;island | 0.20992 | 0.06722 | Not queried | 1.80 | 2.86e-02 | 1.00e+00 |
|  | 13 | 92051400 | 92051786 | PDFSTATSENSEMBLUCSC | 387 | 4 | 11161 | gene;island | 0.14622 | 0.28864 | Not queried | 1.80 | 4.86e-01 | 1.00e+00 |
|  | 1 | 75593804 | 75594261 | PDFSTATSENSEMBLUCSC | 458 | 6 | 3378 | island | 0.85057 | 0.61325 | Not queried | 1.80 | 2.60e-02 | 1.00e+00 |
|  | 5 | 171616253 | 171616574 | PDFSTATSENSEMBLUCSC | 322 | 4 | 32430 | tss;island | 0.14522 | 0.04427 | Not queried | 1.80 | 5.71e-02 | 1.00e+00 |
|  | 3 | 127316944 | 127317259 | PDFSTATSENSEMBLUCSC | 316 | 7 | 29402 | island | 0.11302 | 0.03607 | Not queried | 1.80 | 3.79e-02 | 1.00e+00 |
|  | 15 | 79298209 | 79298396 | PDFSTATSENSEMBLUCSC | 188 | 4 | 13416 | gene;tss | 0.89541 | 0.73135 | Not queried | 1.80 | 2.86e-02 | 1.00e+00 |
|  | 11 | 65625941 | 65626208 | PDFSTATSENSEMBLUCSC | 268 | 7 | 7423 | tss;island | 0.00783 | 0.02116 | Not queried | 1.80 | 4.56e-01 | 1.00e+00 |
|  | 8 | 114447055 | 114447871 | PDFSTATSENSEMBLUCSC | 817 | 6 | 39339 | gene;island | 0.00653 | 0.01925 | Not queried | 1.80 | 6.49e-02 | 1.00e+00 |
|  | 20 | 42544648 | 42544728 | PDFSTATSENSEMBLUCSC | 81 | 6 | 25714 | tss | 0.19976 | 0.04120 | Not queried | 1.80 | 1.32e-01 | 1.00e+00 |
|  | 22 | 42475450 | 42475749 | PDFSTATSENSEMBLUCSC | 300 | 7 | 27498 | island | 0.01198 | 0.03734 | Not queried | 1.80 | 5.30e-02 | 1.00e+00 |
|  | 11 | 46354005 | 46354296 | PDFSTATSENSEMBLUCSC | 292 | 4 | 7253 | tss;island | 0.25281 | 0.06710 | Not queried | 1.80 | 5.71e-02 | 1.00e+00 |
|  | 5 | 87975798 | 87976294 | PDFSTATSENSEMBLUCSC | 497 | 5 | 31624 | gene;island | 0.09482 | 0.02278 | Not queried | 1.80 | 2.22e-01 | 1.00e+00 |
|  | 1 | 75593804 | 75594066 | PDFSTATSENSEMBLUCSC | 263 | 5 | 1695 | tss | 0.84160 | 0.59040 | Not queried | 1.80 | 5.56e-02 | 1.00e+00 |
|  | 5 | 95768997 | 95769079 | PDFSTATSENSEMBLUCSC | 83 | 6 | 32130 | tss | 0.13400 | 0.01917 | Not queried | 1.80 | 1.80e-01 | 1.00e+00 |
|  | 8 | 144798535 | 144799022 | PDFSTATSENSEMBLUCSC | 488 | 6 | 39426 | gene | 0.12354 | 0.04159 | Not queried | 1.80 | 3.94e-01 | 1.00e+00 |
|  | 2 | 232791732 | 232792015 | PDFSTATSENSEMBLUCSC | 284 | 5 | 23909 | tss;island | 0.77605 | 0.46773 | Not queried | 1.80 | 7.94e-03 | 1.00e+00 |
|  | 2 | 27665017 | 27665306 | PDFSTATSENSEMBLUCSC | 290 | 7 | 24147 | island | 0.33683 | 0.63831 | Not queried | 1.80 | 5.83e-04 | 1.00e+00 |
|  | 1 | 20692812 | 20693027 | PDFSTATSENSEMBLUCSC | 216 | 4 | 2785 | island | 0.09655 | 0.24304 | Not queried | 1.80 | 2.86e-02 | 1.00e+00 |
|  | 19 | 8274853 | 8275229 | PDFSTATSENSEMBLUCSC | 377 | 4 | 19756 | gene;island | 0.28685 | 0.11050 | Not queried | 1.80 | 2.00e-01 | 1.00e+00 |
|  | 18 | 12254365 | 12254690 | PDFSTATSENSEMBLUCSC | 326 | 9 | 19027 | gene | 0.57487 | 0.81215 | Not queried | 1.80 | 3.15e-02 | 1.00e+00 |
|  | 6 | 32797476 | 32797830 | PDFSTATSENSEMBLUCSC | 355 | 5 | 34074 | gene | 0.49179 | 0.71973 | Not queried | 1.79 | 1.00e+00 | 1.00e+00 |
|  | 3 | 186490656 | 186490915 | PDFSTATSENSEMBLUCSC | 260 | 5 | 29708 | island | 0.37222 | 0.70058 | Not queried | 1.79 | 7.94e-03 | 1.00e+00 |
|  | 7 | 44152996 | 44153500 | PDFSTATSENSEMBLUCSC | 505 | 6 | 36927 | gene;island | 0.69841 | 0.92168 | Not queried | 1.79 | 6.49e-02 | 1.00e+00 |
|  | 10 | 94455543 | 94455895 | PDFSTATSENSEMBLUCSC | 353 | 4 | 5927 | island | 0.56951 | 0.79034 | Not queried | 1.79 | 2.86e-02 | 1.00e+00 |
|  | 8 | 1364427 | 1364689 | PDFSTATSENSEMBLUCSC | 263 | 5 | 39959 | island | 0.83269 | 0.93345 | Not queried | 1.79 | 2.22e-01 | 1.00e+00 |
|  | 15 | 79382548 | 79383167 | PDFSTATSENSEMBLUCSC | 620 | 7 | 13418 | gene;island | 0.01747 | 0.03627 | Not queried | 1.79 | 1.65e-01 | 1.00e+00 |
|  | 11 | 8832111 | 8832283 | PDFSTATSENSEMBLUCSC | 173 | 4 | 6481 | gene | 0.14729 | 0.06550 | Not queried | 1.79 | 2.86e-02 | 1.00e+00 |
|  | 16 | 81254010 | 81254209 | PDFSTATSENSEMBLUCSC | 200 | 4 | 15350 | tss | 0.79701 | 0.45544 | Not queried | 1.79 | 5.71e-02 | 1.00e+00 |
|  | 6 | 101846650 | 101846805 | PDFSTATSENSEMBLUCSC | 156 | 7 | 35079 | tss | 0.09792 | 0.03167 | Not queried | 1.79 | 9.73e-02 | 1.00e+00 |
|  | 8 | 53852030 | 53852446 | PDFSTATSENSEMBLUCSC | 417 | 7 | 39636 | tss;island | 0.02873 | 0.08346 | Not queried | 1.79 | 2.62e-02 | 1.00e+00 |
|  | 6 | 137241923 | 137242316 | PDFSTATSENSEMBLUCSC | 394 | 5 | 35181 | tss;island | 0.23498 | 0.07598 | Not queried | 1.79 | 9.52e-02 | 1.00e+00 |
|  | 4 | 90757351 | 90757814 | PDFSTATSENSEMBLUCSC | 464 | 7 | 30011 | gene;island | 0.04168 | 0.19052 | Not queried | 1.79 | 1.11e-02 | 1.00e+00 |
|  | X | 69665036 | 69665233 | PDFSTATSENSEMBLUCSC | 198 | 4 | 41017 | gene;island | 0.10931 | 0.33266 | Not queried | 1.79 | 5.71e-02 | 1.00e+00 |
|  | 3 | 13974286 | 13974800 | PDFSTATSENSEMBLUCSC | 515 | 4 | 28866 | island | 0.96320 | 0.87359 | Not queried | 1.79 | 2.00e-01 | 1.00e+00 |
|  | 8 | 142183507 | 142183860 | PDFSTATSENSEMBLUCSC | 354 | 4 | 39383 | gene;island | 0.43779 | 0.76590 | Not queried | 1.79 | 2.00e-01 | 1.00e+00 |
|  | 3 | 178865550 | 178866004 | PDFSTATSENSEMBLUCSC | 455 | 5 | 28684 | tss;island | 0.02271 | 0.06012 | Not queried | 1.79 | 1.51e-01 | 1.00e+00 |
|  | 7 | 49815117 | 49815938 | PDFSTATSENSEMBLUCSC | 822 | 7 | 36946 | gene;island | 0.58653 | 0.25561 | Not queried | 1.78 | 1.28e-01 | 1.00e+00 |
|  | 12 | 66123127 | 66123496 | PDFSTATSENSEMBLUCSC | 370 | 4 | 10573 | island | 0.15719 | 0.03737 | Not queried | 1.78 | 2.00e-01 | 1.00e+00 |
|  | 8 | 8859686 | 8860266 | PDFSTATSENSEMBLUCSC | 581 | 7 | 39479 | tss | 0.05894 | 0.19934 | Not queried | 1.78 | 3.83e-01 | 1.00e+00 |
|  | 7 | 157368881 | 157369158 | PDFSTATSENSEMBLUCSC | 278 | 6 | 37266 | gene;island | 0.52492 | 0.89147 | Not queried | 1.78 | 3.94e-01 | 1.00e+00 |
|  | 6 | 28834254 | 28834520 | PDFSTATSENSEMBLUCSC | 267 | 11 | 35537 | island | 0.32012 | 0.61235 | Not queried | 1.78 | 5.53e-04 | 1.00e+00 |
|  | 13 | 50422122 | 50422495 | PDFSTATSENSEMBLUCSC | 374 | 4 | 11708 | island | 0.01684 | 0.02693 | Not queried | 1.78 | 8.86e-01 | 1.00e+00 |
|  | 16 | 12183835 | 12184133 | PDFSTATSENSEMBLUCSC | 299 | 5 | 14639 | gene | 0.57953 | 0.19287 | Not queried | 1.78 | 3.17e-02 | 1.00e+00 |
|  | 1 | 197744757 | 197745238 | PDFSTATSENSEMBLUCSC | 482 | 8 | 2150 | tss;island | 0.13803 | 0.08061 | Not queried | 1.78 | 1.61e-01 | 1.00e+00 |
|  | 19 | 46056723 | 46057029 | PDFSTATSENSEMBLUCSC | 307 | 4 | 20065 | gene;island | 0.73459 | 0.86422 | Not queried | 1.78 | 2.86e-02 | 1.00e+00 |
|  | 6 | 33943520 | 33943929 | PDFSTATSENSEMBLUCSC | 410 | 5 | 35986 | island | 0.66591 | 0.88894 | Not queried | 1.78 | 1.59e-02 | 1.00e+00 |
|  | 13 | 100624207 | 100624373 | PDFSTATSENSEMBLUCSC | 167 | 7 | 11487 | tss | 0.11964 | 0.03117 | Not queried | 1.78 | 1.28e-01 | 1.00e+00 |
|  | 13 | 100623942 | 100624373 | PDFSTATSENSEMBLUCSC | 432 | 9 | 11816 | island | 0.11964 | 0.02298 | Not queried | 1.78 | 4.00e-02 | 1.00e+00 |
|  | 11 | 14664598 | 14665070 | PDFSTATSENSEMBLUCSC | 473 | 7 | 6498 | gene;tss;island | 0.28752 | 0.10405 | Not queried | 1.78 | 3.18e-01 | 1.00e+00 |
|  | X | 17879424 | 17879951 | PDFSTATSENSEMBLUCSC | 528 | 6 | 41670 | island | 0.07282 | 0.07928 | Not queried | 1.78 | 5.89e-01 | 1.00e+00 |
|  | 12 | 132923339 | 132923603 | PDFSTATSENSEMBLUCSC | 265 | 4 | 11017 | island | 0.60952 | 0.83299 | Not queried | 1.78 | 2.00e-01 | 1.00e+00 |
|  | 3 | 71180032 | 71180071 | PDFSTATSENSEMBLUCSC | 40 | 4 | 27816 | gene | 0.66689 | 0.77177 | Not queried | 1.78 | 2.00e-01 | 1.00e+00 |
|  | 17 | 73893198 | 73893209 | PDFSTATSENSEMBLUCSC | 12 | 4 | 17632 | tss | 0.05004 | 0.14644 | Not queried | 1.78 | 1.14e-01 | 1.00e+00 |
|  | 17 | 4872326 | 4872603 | PDFSTATSENSEMBLUCSC | 278 | 4 | 16325 | gene;tss;island | 0.76637 | 0.60068 | Not queried | 1.78 | 1.14e-01 | 1.00e+00 |
|  | 1 | 156696747 | 156697041 | PDFSTATSENSEMBLUCSC | 295 | 4 | 826 | gene;island | 0.29583 | 0.51429 | Not queried | 1.78 | 1.14e-01 | 1.00e+00 |
|  | 17 | 1810459 | 1810853 | PDFSTATSENSEMBLUCSC | 395 | 5 | 17799 | island | 0.58423 | 0.79278 | Not queried | 1.78 | 3.17e-02 | 1.00e+00 |
|  | 1 | 6260181 | 6260373 | PDFSTATSENSEMBLUCSC | 193 | 4 | 1196 | tss;island | 0.48522 | 0.14986 | Not queried | 1.78 | 3.43e-01 | 1.00e+00 |
|  | 1 | 245753302 | 245753635 | PDFSTATSENSEMBLUCSC | 334 | 4 | 1115 | gene;island | 0.58223 | 0.82672 | Not queried | 1.78 | 2.00e-01 | 1.00e+00 |
|  | 3 | 147140754 | 147140930 | PDFSTATSENSEMBLUCSC | 177 | 4 | 29532 | island | 0.08855 | 0.03780 | Not queried | 1.78 | 5.71e-02 | 1.00e+00 |
|  | 19 | 58629719 | 58630612 | PDFSTATSENSEMBLUCSC | 894 | 10 | 22457 | island | 0.46031 | 0.66838 | Not queried | 1.78 | 7.53e-02 | 1.00e+00 |
|  | 1 | 47082037 | 47082372 | PDFSTATSENSEMBLUCSC | 336 | 5 | 511 | gene;island | 0.18420 | 0.09447 | Not queried | 1.78 | 1.51e-01 | 1.00e+00 |
|  | 11 | 20184128 | 20184779 | PDFSTATSENSEMBLUCSC | 652 | 7 | 8104 | island | 0.12325 | 0.04808 | Not queried | 1.77 | 7.28e-02 | 1.00e+00 |
|  | 15 | 79298604 | 79298769 | PDFSTATSENSEMBLUCSC | 166 | 8 | 13417 | gene;tss | 0.91532 | 0.76530 | Not queried | 1.77 | 6.22e-04 | 1.00e+00 |
|  | 14 | 88792913 | 88793252 | PDFSTATSENSEMBLUCSC | 340 | 6 | 12138 | gene | 0.04774 | 0.20183 | Not queried | 1.77 | 2.60e-02 | 1.00e+00 |
|  | 4 | 4250464 | 4250746 | PDFSTATSENSEMBLUCSC | 283 | 4 | 30218 | tss;island | 0.10215 | 0.02785 | Not queried | 1.77 | 8.86e-01 | 1.00e+00 |
|  | 2 | 119603383 | 119603969 | PDFSTATSENSEMBLUCSC | 587 | 5 | 22804 | gene;island | 0.38442 | 0.15438 | Not queried | 1.77 | 6.90e-01 | 1.00e+00 |
|  | 3 | 164914996 | 164915196 | PDFSTATSENSEMBLUCSC | 201 | 5 | 28657 | tss | 0.13182 | 0.22455 | Not queried | 1.77 | 1.51e-01 | 1.00e+00 |
|  | 4 | 38869118 | 38869779 | PDFSTATSENSEMBLUCSC | 662 | 14 | 30929 | island | 0.12378 | 0.02719 | Not queried | 1.77 | 4.44e-02 | 1.00e+00 |
|  | 17 | 79412583 | 79412788 | PDFSTATSENSEMBLUCSC | 206 | 4 | 16877 | gene;island | 0.83290 | 0.94315 | Not queried | 1.77 | 2.86e-02 | 1.00e+00 |
|  | 5 | 32709071 | 32709396 | PDFSTATSENSEMBLUCSC | 326 | 4 | 32657 | island | 0.79269 | 0.46239 | Not queried | 1.77 | 5.71e-02 | 1.00e+00 |
|  | 16 | 67232180 | 67232557 | PDFSTATSENSEMBLUCSC | 378 | 4 | 14763 | gene;tss;island | 0.67488 | 0.86604 | Not queried | 1.77 | 5.71e-02 | 1.00e+00 |
|  | 17 | 7486551 | 7486961 | PDFSTATSENSEMBLUCSC | 411 | 9 | 17057 | tss | 0.40301 | 0.31169 | Not queried | 1.77 | 2.58e-01 | 1.00e+00 |
|  | 12 | 106533667 | 106533903 | PDFSTATSENSEMBLUCSC | 237 | 6 | 10720 | island | 0.66989 | 0.38914 | Not queried | 1.77 | 8.66e-03 | 1.00e+00 |
|  | 4 | 6695203 | 6695475 | PDFSTATSENSEMBLUCSC | 273 | 6 | 30236 | tss | 0.70120 | 0.88911 | Not queried | 1.77 | 2.16e-03 | 1.00e+00 |
|  | 6 | 30226874 | 30227326 | PDFSTATSENSEMBLUCSC | 453 | 10 | 34702 | tss | 0.15323 | 0.04412 | Not queried | 1.77 | 1.05e-01 | 1.00e+00 |
|  | 2 | 63272214 | 63272334 | PDFSTATSENSEMBLUCSC | 121 | 4 | 22665 | gene;island | 0.36736 | 0.64386 | Not queried | 1.77 | 2.86e-02 | 1.00e+00 |
|  | 2 | 132233433 | 132233607 | PDFSTATSENSEMBLUCSC | 175 | 4 | 23596 | tss | 0.96146 | 0.88058 | Not queried | 1.77 | 3.43e-01 | 1.00e+00 |
|  | 11 | 47587211 | 47587672 | PDFSTATSENSEMBLUCSC | 462 | 4 | 6591 | gene;island | 0.01508 | 0.05334 | Not queried | 1.77 | 1.14e-01 | 1.00e+00 |
|  | 12 | 110888206 | 110888378 | PDFSTATSENSEMBLUCSC | 173 | 4 | 9978 | tss | 0.02088 | 0.04335 | Not queried | 1.77 | 4.86e-01 | 1.00e+00 |
|  | X | 49087234 | 49087477 | PDFSTATSENSEMBLUCSC | 244 | 4 | 40994 | gene;island | 0.05276 | 0.02586 | Not queried | 1.77 | 1.14e-01 | 1.00e+00 |
|  | 19 | 11494980 | 11495295 | PDFSTATSENSEMBLUCSC | 316 | 4 | 21530 | island | 0.33550 | 0.10042 | Not queried | 1.77 | 4.86e-01 | 1.00e+00 |
|  | 4 | 144258809 | 144259119 | PDFSTATSENSEMBLUCSC | 311 | 4 | 30094 | gene;island | 0.02772 | 0.03037 | Not queried | 1.77 | 6.86e-01 | 1.00e+00 |
|  | 15 | 96868857 | 96869220 | PDFSTATSENSEMBLUCSC | 364 | 8 | 14457 | island | 0.02803 | 0.21854 | Not queried | 1.77 | 2.07e-02 | 1.00e+00 |
|  | 6 | 29973221 | 29973669 | PDFSTATSENSEMBLUCSC | 449 | 5 | 33642 | gene;tss;island | 0.77833 | 0.93528 | Not queried | 1.77 | 9.52e-02 | 1.00e+00 |
|  | 5 | 112824497 | 112824920 | PDFSTATSENSEMBLUCSC | 424 | 7 | 32951 | island | 0.44245 | 0.62444 | Not queried | 1.77 | 3.83e-01 | 1.00e+00 |
|  | 5 | 1813772 | 1814201 | PDFSTATSENSEMBLUCSC | 430 | 5 | 31492 | gene | 0.64523 | 0.88810 | Not queried | 1.77 | 1.59e-02 | 1.00e+00 |
|  | 19 | 21579787 | 21579911 | PDFSTATSENSEMBLUCSC | 125 | 6 | 20624 | tss | 0.03751 | 0.02298 | Not queried | 1.77 | 1.80e-01 | 1.00e+00 |
|  | 11 | 442018 | 442380 | PDFSTATSENSEMBLUCSC | 363 | 6 | 6992 | tss | 0.52191 | 0.74533 | Not queried | 1.76 | 2.16e-03 | 1.00e+00 |
|  | 18 | 72201716 | 72201785 | PDFSTATSENSEMBLUCSC | 70 | 4 | 19094 | gene | 0.75111 | 0.92419 | Not queried | 1.76 | 1.14e-01 | 1.00e+00 |
|  | 17 | 7832764 | 7832943 | PDFSTATSENSEMBLUCSC | 180 | 6 | 17070 | tss | 0.08595 | 0.02647 | Not queried | 1.76 | 2.60e-02 | 1.00e+00 |
|  | 10 | 134994186 | 134994455 | PDFSTATSENSEMBLUCSC | 270 | 5 | 4873 | gene;island | 0.93225 | 0.90889 | Not queried | 1.76 | 6.90e-01 | 1.00e+00 |
|  | 6 | 74072242 | 74072376 | PDFSTATSENSEMBLUCSC | 135 | 6 | 35003 | tss | 0.64418 | 0.86504 | Not queried | 1.76 | 1.52e-02 | 1.00e+00 |
|  | 12 | 130645423 | 130645645 | PDFSTATSENSEMBLUCSC | 223 | 4 | 10970 | island | 0.06471 | 0.02170 | Not queried | 1.76 | 1.14e-01 | 1.00e+00 |
|  | 7 | 87936225 | 87936414 | PDFSTATSENSEMBLUCSC | 190 | 5 | 37626 | tss | 0.03525 | 0.11144 | Not queried | 1.76 | 2.22e-01 | 1.00e+00 |
|  | 3 | 122399230 | 122400006 | PDFSTATSENSEMBLUCSC | 777 | 10 | 29360 | island | 0.31332 | 0.62739 | Not queried | 1.76 | 7.53e-02 | 1.00e+00 |
|  | 19 | 49568000 | 49568214 | PDFSTATSENSEMBLUCSC | 215 | 4 | 20902 | tss | 0.06945 | 0.16191 | Not queried | 1.76 | 5.71e-02 | 1.00e+00 |
|  | 17 | 48042605 | 48043063 | PDFSTATSENSEMBLUCSC | 459 | 4 | 18499 | island | 0.57636 | 0.26799 | Not queried | 1.76 | 2.86e-02 | 1.00e+00 |
|  | 1 | 3230250 | 3230647 | PDFSTATSENSEMBLUCSC | 398 | 5 | 118 | gene | 0.45684 | 0.75256 | Not queried | 1.76 | 9.52e-02 | 1.00e+00 |
|  | 1 | 236511281 | 236511627 | PDFSTATSENSEMBLUCSC | 347 | 4 | 4347 | island | 0.58392 | 0.85729 | Not queried | 1.76 | 8.86e-01 | 1.00e+00 |
|  | X | 48432846 | 48433316 | PDFSTATSENSEMBLUCSC | 471 | 6 | 40984 | gene | 0.21269 | 0.57303 | Not queried | 1.76 | 8.66e-03 | 1.00e+00 |
|  | X | 133370784 | 133371052 | PDFSTATSENSEMBLUCSC | 269 | 6 | 41527 | tss | 0.31034 | 0.07082 | Not queried | 1.76 | 9.31e-02 | 1.00e+00 |
|  | 11 | 1221398 | 1221812 | PDFSTATSENSEMBLUCSC | 415 | 4 | 7885 | island | 0.53706 | 0.77373 | Not queried | 1.76 | 2.00e-01 | 1.00e+00 |
|  | 21 | 34638126 | 34638662 | PDFSTATSENSEMBLUCSC | 537 | 7 | 26478 | tss;island | 0.02173 | 0.05300 | Not queried | 1.76 | 9.73e-02 | 1.00e+00 |
|  | 11 | 47586666 | 47586994 | PDFSTATSENSEMBLUCSC | 329 | 4 | 8245 | island | 0.26358 | 0.03758 | Not queried | 1.76 | 2.00e-01 | 1.00e+00 |
|  | 2 | 97173852 | 97174391 | PDFSTATSENSEMBLUCSC | 540 | 10 | 23479 | tss | 0.05044 | 0.17838 | Not queried | 1.76 | 3.55e-02 | 1.00e+00 |
|  | 17 | 48171802 | 48172203 | PDFSTATSENSEMBLUCSC | 402 | 7 | 17458 | tss;island | 0.02532 | 0.07939 | Not queried | 1.76 | 7.28e-02 | 1.00e+00 |
|  | 1 | 713921 | 714177 | PDFSTATSENSEMBLUCSC | 257 | 6 | 2372 | island | 0.03709 | 0.15287 | Not queried | 1.76 | 4.11e-02 | 1.00e+00 |
|  | 17 | 79010831 | 79011542 | PDFSTATSENSEMBLUCSC | 712 | 6 | 16855 | gene;island | 0.62874 | 0.94100 | Not queried | 1.76 | 2.60e-02 | 1.00e+00 |
|  | 11 | 537287 | 537737 | PDFSTATSENSEMBLUCSC | 451 | 11 | 7837 | island | 0.26244 | 0.03578 | Not queried | 1.76 | 3.36e-02 | 1.00e+00 |
|  | 13 | 112630296 | 112630795 | PDFSTATSENSEMBLUCSC | 500 | 7 | 11875 | island | 0.82572 | 0.93603 | Not queried | 1.76 | 1.28e-01 | 1.00e+00 |
|  | 19 | 48897863 | 48898160 | PDFSTATSENSEMBLUCSC | 298 | 4 | 22179 | island | 0.07439 | 0.36681 | Not queried | 1.76 | 1.14e-01 | 1.00e+00 |
|  | 11 | 64703415 | 64703818 | PDFSTATSENSEMBLUCSC | 404 | 5 | 7388 | tss | 0.38197 | 0.67586 | Not queried | 1.75 | 5.48e-01 | 1.00e+00 |
|  | 6 | 108436768 | 108437116 | PDFSTATSENSEMBLUCSC | 349 | 5 | 36329 | island | 0.02812 | 0.08165 | Not queried | 1.75 | 7.94e-03 | 1.00e+00 |
|  | 2 | 228028601 | 228028776 | PDFSTATSENSEMBLUCSC | 176 | 4 | 23020 | gene;tss;island | 0.03761 | 0.03335 | Not queried | 1.75 | 8.86e-01 | 1.00e+00 |
|  | 8 | 23567310 | 23567941 | PDFSTATSENSEMBLUCSC | 632 | 7 | 40092 | island | 0.08131 | 0.05976 | Not queried | 1.75 | 3.83e-01 | 1.00e+00 |
|  | 3 | 197183271 | 197183662 | PDFSTATSENSEMBLUCSC | 392 | 6 | 29773 | island | 0.18106 | 0.45247 | Not queried | 1.75 | 1.52e-02 | 1.00e+00 |
|  | 19 | 44009019 | 44009073 | PDFSTATSENSEMBLUCSC | 55 | 5 | 20793 | tss;island | 0.45241 | 0.18037 | Not queried | 1.75 | 9.52e-02 | 1.00e+00 |
|  | 1 | 47082691 | 47082869 | PDFSTATSENSEMBLUCSC | 179 | 5 | 1600 | tss;island | 0.65430 | 0.30510 | Not queried | 1.75 | 1.51e-01 | 1.00e+00 |
|  | 7 | 94536541 | 94537033 | PDFSTATSENSEMBLUCSC | 493 | 6 | 38610 | island | 0.72453 | 0.35064 | Not queried | 1.75 | 3.10e-01 | 1.00e+00 |
|  | X | 68723670 | 68723812 | PDFSTATSENSEMBLUCSC | 143 | 4 | 41361 | tss;island | 0.07791 | 0.02425 | Not queried | 1.75 | 2.86e-02 | 1.00e+00 |
|  | 4 | 106816089 | 106816564 | PDFSTATSENSEMBLUCSC | 476 | 5 | 30484 | tss;island | 0.21926 | 0.07704 | Not queried | 1.75 | 2.22e-01 | 1.00e+00 |
|  | 5 | 131892242 | 131892523 | PDFSTATSENSEMBLUCSC | 282 | 5 | 32213 | tss | 0.46355 | 0.79183 | Not queried | 1.75 | 1.51e-01 | 1.00e+00 |
|  | 17 | 48585470 | 48585645 | PDFSTATSENSEMBLUCSC | 176 | 4 | 17468 | tss;island | 0.06125 | 0.02973 | Not queried | 1.75 | 2.86e-02 | 1.00e+00 |
|  | 6 | 13274151 | 13274354 | PDFSTATSENSEMBLUCSC | 204 | 5 | 33511 | gene | 0.63836 | 0.84078 | Not queried | 1.75 | 3.17e-02 | 1.00e+00 |
|  | 17 | 77194008 | 77194259 | PDFSTATSENSEMBLUCSC | 252 | 4 | 16802 | gene;island | 0.63159 | 0.85171 | Not queried | 1.75 | 2.86e-02 | 1.00e+00 |
|  | X | 68385381 | 68385467 | PDFSTATSENSEMBLUCSC | 87 | 4 | 41360 | tss | 0.08072 | 0.02004 | Not queried | 1.75 | 3.43e-01 | 1.00e+00 |
|  | 18 | 76160108 | 76160443 | PDFSTATSENSEMBLUCSC | 336 | 5 | 19555 | island | 0.54030 | 0.79804 | Not queried | 1.75 | 1.59e-02 | 1.00e+00 |
|  | 8 | 2242024 | 2242304 | PDFSTATSENSEMBLUCSC | 281 | 4 | 39986 | island | 0.48577 | 0.75479 | Not queried | 1.75 | 2.00e-01 | 1.00e+00 |
|  | 13 | 113698931 | 113699016 | PDFSTATSENSEMBLUCSC | 86 | 7 | 11243 | gene;island | 0.67088 | 0.88203 | Not queried | 1.75 | 4.08e-03 | 1.00e+00 |
|  | 14 | 24803679 | 24804342 | PDFSTATSENSEMBLUCSC | 664 | 10 | 12718 | island | 0.03237 | 0.14360 | Not queried | 1.75 | 7.58e-05 | 3.89e-01 |
|  | 14 | 24803873 | 24804342 | PDFSTATSENSEMBLUCSC | 470 | 8 | 12300 | tss | 0.03237 | 0.14257 | Not queried | 1.75 | 1.09e-03 | 1.00e+00 |
|  | 6 | 32157454 | 32157596 | PDFSTATSENSEMBLUCSC | 143 | 6 | 34019 | gene | 0.23542 | 0.48648 | Not queried | 1.75 | 1.80e-01 | 1.00e+00 |
|  | 14 | 32546448 | 32546633 | PDFSTATSENSEMBLUCSC | 186 | 4 | 12316 | tss;island | 0.02330 | 0.08330 | Not queried | 1.75 | 1.14e-01 | 1.00e+00 |
|  | 1 | 205745431 | 205745761 | PDFSTATSENSEMBLUCSC | 331 | 5 | 2204 | tss;island | 0.97077 | 0.88542 | Not queried | 1.75 | 5.56e-02 | 1.00e+00 |
|  | 7 | 150019955 | 150020269 | PDFSTATSENSEMBLUCSC | 315 | 9 | 37910 | tss | 0.33682 | 0.15736 | Not queried | 1.75 | 4.94e-04 | 1.00e+00 |
|  | 17 | 38118571 | 38118787 | PDFSTATSENSEMBLUCSC | 217 | 4 | 17285 | tss | 0.76359 | 0.89466 | Not queried | 1.74 | 4.86e-01 | 1.00e+00 |
|  | 1 | 35395541 | 35395837 | PDFSTATSENSEMBLUCSC | 297 | 4 | 3024 | island | 0.08527 | 0.02567 | Not queried | 1.74 | 4.86e-01 | 1.00e+00 |
|  | 1 | 207277018 | 207277581 | PDFSTATSENSEMBLUCSC | 564 | 8 | 2215 | tss | 0.68472 | 0.84385 | Not queried | 1.74 | 2.79e-01 | 1.00e+00 |
|  | 3 | 136751342 | 136751795 | PDFSTATSENSEMBLUCSC | 454 | 5 | 29468 | island | 0.04408 | 0.16245 | Not queried | 1.74 | 9.52e-02 | 1.00e+00 |
|  | X | 16888414 | 16888622 | PDFSTATSENSEMBLUCSC | 209 | 4 | 41664 | island | 0.01454 | 0.03743 | Not queried | 1.74 | 5.71e-02 | 1.00e+00 |
|  | X | 13587976 | 13588301 | PDFSTATSENSEMBLUCSC | 326 | 4 | 40939 | gene;island | 0.03131 | 0.05188 | Not queried | 1.74 | 4.86e-01 | 1.00e+00 |
|  | 10 | 12110577 | 12110759 | PDFSTATSENSEMBLUCSC | 183 | 6 | 4933 | tss | 0.01665 | 0.03564 | Not queried | 1.74 | 3.10e-01 | 1.00e+00 |
|  | 10 | 81892109 | 81892161 | PDFSTATSENSEMBLUCSC | 53 | 4 | 5169 | tss;island | 0.53282 | 0.25852 | Not queried | 1.74 | 2.86e-02 | 1.00e+00 |
|  | 12 | 54329854 | 54330354 | PDFSTATSENSEMBLUCSC | 501 | 5 | 10450 | island | 0.83055 | 0.59328 | Not queried | 1.74 | 1.51e-01 | 1.00e+00 |
|  | 14 | 65239327 | 65239653 | PDFSTATSENSEMBLUCSC | 327 | 4 | 12087 | gene;island | 0.57395 | 0.88451 | Not queried | 1.74 | 5.71e-02 | 1.00e+00 |
|  | 16 | 31228427 | 31228555 | PDFSTATSENSEMBLUCSC | 129 | 4 | 15179 | tss | 0.14497 | 0.28756 | Not queried | 1.74 | 2.86e-02 | 1.00e+00 |
|  | 11 | 598325 | 598696 | PDFSTATSENSEMBLUCSC | 372 | 4 | 6364 | gene;island | 0.70420 | 0.91248 | Not queried | 1.74 | 2.86e-02 | 1.00e+00 |
|  | 15 | 100533202 | 100533336 | PDFSTATSENSEMBLUCSC | 135 | 4 | 13475 | gene;island | 0.88860 | 0.66543 | Not queried | 1.74 | 2.00e-01 | 1.00e+00 |
|  | 11 | 134147143 | 134147634 | PDFSTATSENSEMBLUCSC | 492 | 4 | 6974 | gene;island | 0.15991 | 0.03485 | Not queried | 1.74 | 5.71e-02 | 1.00e+00 |
|  | 10 | 27609163 | 27609333 | PDFSTATSENSEMBLUCSC | 171 | 4 | 5611 | island | 0.39201 | 0.55679 | Not queried | 1.74 | 6.86e-01 | 1.00e+00 |
|  | 6 | 31922863 | 31923212 | PDFSTATSENSEMBLUCSC | 350 | 4 | 33936 | gene;island | 0.69860 | 0.91201 | Not queried | 1.74 | 5.71e-02 | 1.00e+00 |
|  | 19 | 3062808 | 3062986 | PDFSTATSENSEMBLUCSC | 179 | 4 | 20302 | tss;island | 0.48747 | 0.16063 | Not queried | 1.74 | 1.14e-01 | 1.00e+00 |
|  | 17 | 5402883 | 5403516 | PDFSTATSENSEMBLUCSC | 634 | 6 | 16332 | gene;island | 0.41635 | 0.11760 | Not queried | 1.74 | 2.40e-01 | 1.00e+00 |
|  | 1 | 35220224 | 35220710 | PDFSTATSENSEMBLUCSC | 487 | 6 | 1475 | tss | 0.81450 | 0.52442 | Not queried | 1.73 | 1.52e-02 | 1.00e+00 |
|  | 10 | 11344086 | 11344395 | PDFSTATSENSEMBLUCSC | 310 | 4 | 4481 | gene | 0.96845 | 0.89516 | Not queried | 1.73 | 2.86e-02 | 1.00e+00 |
|  | 1 | 85512957 | 85513315 | PDFSTATSENSEMBLUCSC | 359 | 4 | 603 | gene;island | 0.53259 | 0.26760 | Not queried | 1.73 | 1.14e-01 | 1.00e+00 |
|  | 1 | 29587088 | 29587490 | PDFSTATSENSEMBLUCSC | 403 | 5 | 376 | gene;island | 0.96602 | 0.89426 | Not queried | 1.73 | 5.56e-02 | 1.00e+00 |
|  | 14 | 96180576 | 96181044 | PDFSTATSENSEMBLUCSC | 469 | 9 | 12562 | tss | 0.71469 | 0.90315 | Not queried | 1.73 | 4.11e-05 | 2.11e-01 |
|  | 14 | 96180319 | 96181044 | PDFSTATSENSEMBLUCSC | 726 | 11 | 13084 | island | 0.68556 | 0.88042 | Not queried | 1.73 | 8.51e-05 | 4.37e-01 |
|  | 17 | 78388520 | 78388593 | PDFSTATSENSEMBLUCSC | 74 | 4 | 16832 | gene;tss;island | 0.04052 | 0.11816 | Not queried | 1.73 | 5.71e-02 | 1.00e+00 |
|  | 7 | 75932840 | 75932977 | PDFSTATSENSEMBLUCSC | 138 | 6 | 36990 | gene;island | 0.02140 | 0.09785 | Not queried | 1.73 | 8.66e-03 | 1.00e+00 |
|  | 13 | 33780205 | 33780362 | PDFSTATSENSEMBLUCSC | 158 | 4 | 11358 | tss | 0.80551 | 0.55617 | Not queried | 1.73 | 2.86e-02 | 1.00e+00 |
|  | 6 | 33169975 | 33170452 | PDFSTATSENSEMBLUCSC | 478 | 6 | 34158 | gene;island | 0.46920 | 0.75947 | Not queried | 1.73 | 6.49e-02 | 1.00e+00 |
|  | 7 | 1315546 | 1315866 | PDFSTATSENSEMBLUCSC | 321 | 4 | 38032 | island | 0.60132 | 0.92973 | Not queried | 1.73 | 2.00e-01 | 1.00e+00 |
|  | 4 | 2930073 | 2930361 | PDFSTATSENSEMBLUCSC | 289 | 4 | 29850 | gene;island | 0.89904 | 0.94246 | Not queried | 1.73 | 2.00e-01 | 1.00e+00 |
|  | X | 108778793 | 108779030 | PDFSTATSENSEMBLUCSC | 238 | 4 | 41955 | island | 0.71408 | 0.89273 | Not queried | 1.73 | 2.00e-01 | 1.00e+00 |
|  | 2 | 21266947 | 21267334 | PDFSTATSENSEMBLUCSC | 388 | 9 | 23179 | tss;island | 0.30916 | 0.58342 | Not queried | 1.73 | 2.76e-03 | 1.00e+00 |
|  | 8 | 144824625 | 144824658 | PDFSTATSENSEMBLUCSC | 34 | 4 | 40621 | island | 0.73561 | 0.89884 | Not queried | 1.73 | 2.86e-02 | 1.00e+00 |
|  | 7 | 45808481 | 45808808 | PDFSTATSENSEMBLUCSC | 328 | 8 | 38412 | island | 0.03652 | 0.06244 | Not queried | 1.73 | 8.30e-02 | 1.00e+00 |
|  | 10 | 135122746 | 135123421 | PDFSTATSENSEMBLUCSC | 676 | 12 | 5442 | tss | 0.04225 | 0.06689 | Not queried | 1.73 | 1.98e-01 | 1.00e+00 |
|  | 10 | 100991959 | 100992222 | PDFSTATSENSEMBLUCSC | 264 | 4 | 4691 | gene;island | 0.30988 | 0.10848 | Not queried | 1.73 | 1.14e-01 | 1.00e+00 |
|  | 12 | 117593259 | 117593728 | PDFSTATSENSEMBLUCSC | 470 | 7 | 9310 | gene;island | 0.55117 | 0.79206 | Not queried | 1.72 | 1.65e-01 | 1.00e+00 |
|  | 7 | 121950924 | 121951055 | PDFSTATSENSEMBLUCSC | 132 | 4 | 38808 | island | 0.09864 | 0.03199 | Not queried | 1.72 | 5.71e-02 | 1.00e+00 |
|  | 13 | 33780078 | 33780362 | PDFSTATSENSEMBLUCSC | 285 | 5 | 11097 | gene | 0.78745 | 0.58381 | Not queried | 1.72 | 1.59e-02 | 1.00e+00 |
|  | 1 | 29213106 | 29213367 | PDFSTATSENSEMBLUCSC | 262 | 4 | 1434 | tss;island | 0.07992 | 0.04584 | Not queried | 1.72 | 3.43e-01 | 1.00e+00 |
|  | 17 | 33759929 | 33759986 | PDFSTATSENSEMBLUCSC | 58 | 4 | 17236 | tss | 0.92677 | 0.97188 | Not queried | 1.72 | 5.71e-02 | 1.00e+00 |
|  | 15 | 78632670 | 78632861 | PDFSTATSENSEMBLUCSC | 192 | 5 | 13409 | gene | 0.23164 | 0.49688 | Not queried | 1.72 | 7.94e-03 | 1.00e+00 |
|  | 8 | 86350278 | 86350592 | PDFSTATSENSEMBLUCSC | 315 | 6 | 39730 | tss;island | 0.15460 | 0.36232 | Not queried | 1.72 | 2.60e-02 | 1.00e+00 |
|  | 3 | 35785735 | 35786030 | PDFSTATSENSEMBLUCSC | 296 | 5 | 27671 | gene | 0.57758 | 0.22165 | Not queried | 1.72 | 3.10e-01 | 1.00e+00 |
|  | 7 | 157644160 | 157644665 | PDFSTATSENSEMBLUCSC | 506 | 5 | 37282 | gene;island | 0.38756 | 0.16092 | Not queried | 1.72 | 9.52e-02 | 1.00e+00 |
|  | 1 | 26503623 | 26503979 | PDFSTATSENSEMBLUCSC | 357 | 8 | 1381 | tss | 0.06364 | 0.18370 | Not queried | 1.72 | 6.50e-02 | 1.00e+00 |
|  | 14 | 105953088 | 105953244 | PDFSTATSENSEMBLUCSC | 157 | 4 | 12645 | tss | 0.03199 | 0.10276 | Not queried | 1.72 | 2.00e-01 | 1.00e+00 |
|  | 14 | 105953088 | 105953462 | PDFSTATSENSEMBLUCSC | 375 | 6 | 13212 | island | 0.03199 | 0.11340 | Not queried | 1.72 | 2.40e-01 | 1.00e+00 |
|  | 12 | 51818350 | 51818768 | PDFSTATSENSEMBLUCSC | 419 | 7 | 10401 | island | 0.11000 | 0.02150 | Not queried | 1.72 | 5.30e-02 | 1.00e+00 |
|  | 15 | 89312795 | 89313222 | PDFSTATSENSEMBLUCSC | 428 | 7 | 14393 | island | 0.09094 | 0.32699 | Not queried | 1.72 | 1.11e-02 | 1.00e+00 |
|  | 15 | 49338251 | 49339070 | PDFSTATSENSEMBLUCSC | 820 | 8 | 14098 | island | 0.03943 | 0.05633 | Not queried | 1.72 | 3.28e-01 | 1.00e+00 |
|  | 8 | 145501817 | 145502297 | PDFSTATSENSEMBLUCSC | 481 | 5 | 39450 | gene;island | 0.72540 | 0.89377 | Not queried | 1.72 | 7.94e-03 | 1.00e+00 |
|  | 19 | 38210837 | 38210932 | PDFSTATSENSEMBLUCSC | 96 | 4 | 20708 | tss | 0.04440 | 0.16420 | Not queried | 1.72 | 2.00e-01 | 1.00e+00 |
|  | 5 | 110074217 | 110074725 | PDFSTATSENSEMBLUCSC | 509 | 10 | 32146 | tss | 0.07598 | 0.01868 | Not queried | 1.72 | 6.84e-03 | 1.00e+00 |
|  | 1 | 113216774 | 113217043 | PDFSTATSENSEMBLUCSC | 270 | 5 | 1834 | tss | 0.06211 | 0.25522 | Not queried | 1.72 | 5.56e-02 | 1.00e+00 |
|  | 12 | 52257893 | 52258264 | PDFSTATSENSEMBLUCSC | 372 | 4 | 10405 | island | 0.05056 | 0.01850 | Not queried | 1.72 | 2.86e-02 | 1.00e+00 |
|  | X | 56258113 | 56259373 | PDFSTATSENSEMBLUCSC | 1261 | 14 | 41821 | island | 0.05722 | 0.02863 | Not queried | 1.72 | 3.52e-03 | 1.00e+00 |
|  | 1 | 1549615 | 1549818 | PDFSTATSENSEMBLUCSC | 204 | 4 | 1154 | tss;island | 0.02406 | 0.12641 | Not queried | 1.72 | 5.71e-02 | 1.00e+00 |
|  | 5 | 54281437 | 54281733 | PDFSTATSENSEMBLUCSC | 297 | 4 | 32003 | tss | 0.19647 | 0.43121 | Not queried | 1.71 | 2.00e-01 | 1.00e+00 |
|  | 4 | 7593370 | 7593585 | PDFSTATSENSEMBLUCSC | 216 | 4 | 29894 | gene;island | 0.80740 | 0.91963 | Not queried | 1.71 | 2.00e-01 | 1.00e+00 |
|  | 17 | 66097276 | 66097529 | PDFSTATSENSEMBLUCSC | 254 | 5 | 17569 | tss;island | 0.12116 | 0.06868 | Not queried | 1.71 | 5.56e-02 | 1.00e+00 |
|  | 6 | 31237664 | 31238036 | PDFSTATSENSEMBLUCSC | 373 | 5 | 33802 | gene;island | 0.69291 | 0.68953 | Not queried | 1.71 | 8.41e-01 | 1.00e+00 |
|  | X | 128812233 | 128812557 | PDFSTATSENSEMBLUCSC | 325 | 5 | 42018 | island | 0.56317 | 0.80400 | Not queried | 1.71 | 1.59e-02 | 1.00e+00 |
|  | 6 | 31324916 | 31325455 | PDFSTATSENSEMBLUCSC | 540 | 16 | 35706 | island | 0.15277 | 0.02903 | Not queried | 1.71 | 1.52e-02 | 1.00e+00 |
|  | 2 | 10567526 | 10567843 | PDFSTATSENSEMBLUCSC | 318 | 4 | 24030 | island | 0.85297 | 0.96574 | Not queried | 1.71 | 3.43e-01 | 1.00e+00 |
|  | 17 | 39216457 | 39216687 | PDFSTATSENSEMBLUCSC | 231 | 4 | 17304 | tss | 0.57260 | 0.87381 | Not queried | 1.71 | 5.71e-02 | 1.00e+00 |
|  | 6 | 152957995 | 152958664 | PDFSTATSENSEMBLUCSC | 670 | 14 | 35242 | tss | 0.11476 | 0.05590 | Not queried | 1.71 | 4.01e-01 | 1.00e+00 |
|  | 2 | 239046684 | 239047336 | PDFSTATSENSEMBLUCSC | 653 | 8 | 23932 | tss;island | 0.45409 | 0.71173 | Not queried | 1.71 | 2.07e-02 | 1.00e+00 |
|  | 2 | 98351769 | 98352002 | PDFSTATSENSEMBLUCSC | 234 | 4 | 22759 | gene;island | 0.56908 | 0.81602 | Not queried | 1.71 | 2.86e-02 | 1.00e+00 |
|  | 17 | 7832680 | 7832943 | PDFSTATSENSEMBLUCSC | 264 | 7 | 17945 | island | 0.09614 | 0.03021 | Not queried | 1.71 | 7.28e-02 | 1.00e+00 |
|  | 16 | 69141250 | 69141478 | PDFSTATSENSEMBLUCSC | 229 | 7 | 14787 | gene;island | 0.10962 | 0.03527 | Not queried | 1.71 | 1.11e-02 | 1.00e+00 |
|  | 7 | 1596103 | 1596260 | PDFSTATSENSEMBLUCSC | 158 | 5 | 37337 | tss | 0.65702 | 0.89842 | Not queried | 1.71 | 5.56e-02 | 1.00e+00 |
|  | 6 | 30122947 | 30123226 | PDFSTATSENSEMBLUCSC | 280 | 5 | 33667 | gene | 0.21741 | 0.31255 | Not queried | 1.71 | 1.51e-01 | 1.00e+00 |
|  | 14 | 31343146 | 31343427 | PDFSTATSENSEMBLUCSC | 282 | 4 | 12310 | tss;island | 0.07037 | 0.02239 | Not queried | 1.71 | 5.71e-02 | 1.00e+00 |
|  | 1 | 151812421 | 151812710 | PDFSTATSENSEMBLUCSC | 290 | 4 | 754 | gene;island | 0.16693 | 0.04870 | Not queried | 1.71 | 2.86e-02 | 1.00e+00 |
|  | 22 | 51221675 | 51221773 | PDFSTATSENSEMBLUCSC | 99 | 4 | 26894 | gene;tss;island | 0.01507 | 0.03607 | Not queried | 1.71 | 3.43e-01 | 1.00e+00 |
|  | 6 | 33136859 | 33137336 | PDFSTATSENSEMBLUCSC | 478 | 7 | 34134 | gene | 0.66933 | 0.29825 | Not queried | 1.71 | 1.75e-02 | 1.00e+00 |
|  | 6 | 33383065 | 33383412 | PDFSTATSENSEMBLUCSC | 348 | 4 | 34215 | gene;island | 0.81650 | 0.92636 | Not queried | 1.71 | 3.43e-01 | 1.00e+00 |
|  | 17 | 73629082 | 73629463 | PDFSTATSENSEMBLUCSC | 382 | 6 | 16736 | gene;tss;island | 0.37892 | 0.68529 | Not queried | 1.70 | 4.11e-02 | 1.00e+00 |
|  | 7 | 2563478 | 2563909 | PDFSTATSENSEMBLUCSC | 432 | 5 | 36742 | gene;island | 0.62786 | 0.31378 | Not queried | 1.70 | 7.94e-03 | 1.00e+00 |
|  | 5 | 1298511 | 1298965 | PDFSTATSENSEMBLUCSC | 455 | 6 | 32565 | island | 0.58348 | 0.76761 | Not queried | 1.70 | 1.32e-01 | 1.00e+00 |
|  | 13 | 47371412 | 47371644 | PDFSTATSENSEMBLUCSC | 233 | 8 | 11400 | tss | 0.05109 | 0.11549 | Not queried | 1.70 | 1.30e-01 | 1.00e+00 |
|  | 6 | 28553927 | 28555079 | PDFSTATSENSEMBLUCSC | 1153 | 20 | 33575 | gene | 0.02775 | 0.06856 | Not queried | 1.70 | 1.03e-02 | 1.00e+00 |
|  | 17 | 35078659 | 35079061 | PDFSTATSENSEMBLUCSC | 403 | 4 | 18200 | island | 0.54478 | 0.79964 | Not queried | 1.70 | 4.86e-01 | 1.00e+00 |
|  | 5 | 172175604 | 172175855 | PDFSTATSENSEMBLUCSC | 252 | 4 | 33331 | island | 0.47423 | 0.79001 | Not queried | 1.70 | 1.14e-01 | 1.00e+00 |
|  | 6 | 31913759 | 31914305 | PDFSTATSENSEMBLUCSC | 547 | 9 | 33932 | gene | 0.69661 | 0.37580 | Not queried | 1.70 | 1.23e-03 | 1.00e+00 |
|  | 1 | 226111974 | 226112534 | PDFSTATSENSEMBLUCSC | 561 | 6 | 2280 | tss | 0.05228 | 0.30579 | Not queried | 1.70 | 1.80e-01 | 1.00e+00 |
|  | 15 | 85113863 | 85114479 | PDFSTATSENSEMBLUCSC | 617 | 8 | 14373 | island | 0.02556 | 0.19881 | Not queried | 1.70 | 1.05e-01 | 1.00e+00 |
|  | 4 | 699224 | 699435 | PDFSTATSENSEMBLUCSC | 212 | 6 | 30183 | tss;island | 0.04400 | 0.12027 | Not queried | 1.70 | 1.52e-02 | 1.00e+00 |
|  | 1 | 46951291 | 46951683 | PDFSTATSENSEMBLUCSC | 393 | 6 | 3213 | island | 0.17412 | 0.04558 | Not queried | 1.70 | 1.32e-01 | 1.00e+00 |
|  | 17 | 46703854 | 46704004 | PDFSTATSENSEMBLUCSC | 151 | 6 | 17435 | tss;island | 0.05271 | 0.15411 | Not queried | 1.70 | 1.32e-01 | 1.00e+00 |
|  | 2 | 51254021 | 51254170 | PDFSTATSENSEMBLUCSC | 150 | 4 | 22640 | gene;island | 0.59827 | 0.82682 | Not queried | 1.70 | 2.00e-01 | 1.00e+00 |
|  | 2 | 33359198 | 33359550 | PDFSTATSENSEMBLUCSC | 353 | 5 | 23260 | tss | 0.68991 | 0.83075 | Not queried | 1.70 | 5.56e-02 | 1.00e+00 |
|  | 8 | 10872604 | 10872819 | PDFSTATSENSEMBLUCSC | 216 | 4 | 39139 | gene;island | 0.12194 | 0.03515 | Not queried | 1.70 | 5.71e-02 | 1.00e+00 |
|  | 13 | 102104976 | 102105129 | PDFSTATSENSEMBLUCSC | 154 | 5 | 11185 | gene | 0.48251 | 0.74912 | Not queried | 1.70 | 3.17e-02 | 1.00e+00 |
|  | 13 | 102104573 | 102105129 | PDFSTATSENSEMBLUCSC | 557 | 9 | 11827 | island | 0.48251 | 0.74912 | Not queried | 1.70 | 1.85e-03 | 1.00e+00 |
|  | 10 | 121484846 | 121485184 | PDFSTATSENSEMBLUCSC | 339 | 6 | 5384 | tss;island | 0.02025 | 0.05412 | Not queried | 1.70 | 4.33e-03 | 1.00e+00 |
|  | 10 | 131665838 | 131666314 | PDFSTATSENSEMBLUCSC | 477 | 6 | 4816 | gene;island | 0.80608 | 0.94559 | Not queried | 1.70 | 9.31e-02 | 1.00e+00 |
|  | 17 | 8868541 | 8868834 | PDFSTATSENSEMBLUCSC | 294 | 4 | 16399 | gene;island | 0.23955 | 0.20248 | Not queried | 1.70 | 6.86e-01 | 1.00e+00 |
|  | 14 | 103986227 | 103986467 | PDFSTATSENSEMBLUCSC | 241 | 4 | 12208 | gene;island | 0.79480 | 0.94904 | Not queried | 1.70 | 5.71e-02 | 1.00e+00 |
|  | 15 | 85114131 | 85114479 | PDFSTATSENSEMBLUCSC | 349 | 6 | 13839 | tss | 0.02028 | 0.03164 | Not queried | 1.69 | 3.10e-01 | 1.00e+00 |
|  | 10 | 71087834 | 71088038 | PDFSTATSENSEMBLUCSC | 205 | 4 | 4599 | gene | 0.66866 | 0.86299 | Not queried | 1.69 | 2.86e-02 | 1.00e+00 |
|  | 2 | 128422113 | 128422717 | PDFSTATSENSEMBLUCSC | 605 | 10 | 22825 | gene;island | 0.51720 | 0.80837 | Not queried | 1.69 | 2.80e-01 | 1.00e+00 |
|  | 12 | 69753119 | 69753455 | PDFSTATSENSEMBLUCSC | 337 | 5 | 9835 | tss | 0.02406 | 0.02830 | Not queried | 1.69 | 6.90e-01 | 1.00e+00 |
|  | 11 | 1968266 | 1968980 | PDFSTATSENSEMBLUCSC | 715 | 8 | 7918 | island | 0.03857 | 0.12590 | Not queried | 1.69 | 1.48e-02 | 1.00e+00 |
|  | 1 | 27961563 | 27962037 | PDFSTATSENSEMBLUCSC | 475 | 7 | 2919 | island | 0.80453 | 0.56015 | Not queried | 1.69 | 1.28e-01 | 1.00e+00 |
|  | 13 | 33859993 | 33860083 | PDFSTATSENSEMBLUCSC | 91 | 6 | 11359 | tss | 0.03569 | 0.05509 | Not queried | 1.69 | 2.40e-01 | 1.00e+00 |
|  | 10 | 134928055 | 134928245 | PDFSTATSENSEMBLUCSC | 191 | 4 | 4869 | gene;island | 0.79622 | 0.92188 | Not queried | 1.69 | 3.43e-01 | 1.00e+00 |
|  | 17 | 8661887 | 8662011 | PDFSTATSENSEMBLUCSC | 125 | 5 | 17088 | tss | 0.88770 | 0.71462 | Not queried | 1.69 | 6.90e-01 | 1.00e+00 |
|  | 14 | 27066404 | 27066771 | PDFSTATSENSEMBLUCSC | 368 | 4 | 12023 | gene;island | 0.01576 | 0.02443 | Not queried | 1.69 | 4.86e-01 | 1.00e+00 |
|  | 19 | 35791192 | 35791409 | PDFSTATSENSEMBLUCSC | 218 | 4 | 19947 | gene;island | 0.35174 | 0.60009 | Not queried | 1.69 | 3.43e-01 | 1.00e+00 |
|  | 13 | 114914537 | 114914682 | PDFSTATSENSEMBLUCSC | 146 | 4 | 11968 | island | 0.62082 | 0.84592 | Not queried | 1.69 | 3.43e-01 | 1.00e+00 |
|  | 14 | 24685281 | 24685421 | PDFSTATSENSEMBLUCSC | 141 | 4 | 12293 | tss | 0.01457 | 0.03606 | Not queried | 1.69 | 2.00e-01 | 1.00e+00 |
|  | 2 | 133426129 | 133426418 | PDFSTATSENSEMBLUCSC | 290 | 4 | 22841 | gene;island | 0.03184 | 0.07861 | Not queried | 1.69 | 3.43e-01 | 1.00e+00 |
|  | 18 | 74175071 | 74175208 | PDFSTATSENSEMBLUCSC | 138 | 4 | 19293 | tss | 0.91258 | 0.93877 | Not queried | 1.69 | 3.43e-01 | 1.00e+00 |
|  | 13 | 61988802 | 61989203 | PDFSTATSENSEMBLUCSC | 402 | 4 | 11146 | gene | 0.02709 | 0.03970 | Not queried | 1.69 | 4.86e-01 | 1.00e+00 |
|  | 17 | 50237205 | 50237844 | PDFSTATSENSEMBLUCSC | 640 | 9 | 17482 | tss;island | 0.38541 | 0.62457 | Not queried | 1.69 | 7.70e-02 | 1.00e+00 |
|  | 1 | 109655203 | 109655543 | PDFSTATSENSEMBLUCSC | 341 | 4 | 662 | gene;tss;island | 0.04901 | 0.10725 | Not queried | 1.69 | 4.86e-01 | 1.00e+00 |
|  | 3 | 98376390 | 98376574 | PDFSTATSENSEMBLUCSC | 185 | 4 | 29273 | island | 0.03341 | 0.10117 | Not queried | 1.69 | 2.86e-02 | 1.00e+00 |
|  | 5 | 497397 | 497639 | PDFSTATSENSEMBLUCSC | 243 | 4 | 31454 | gene;island | 0.43932 | 0.73661 | Not queried | 1.69 | 2.86e-02 | 1.00e+00 |
|  | 16 | 2907517 | 2907859 | PDFSTATSENSEMBLUCSC | 343 | 4 | 14587 | gene;island | 0.21120 | 0.45206 | Not queried | 1.69 | 2.00e-01 | 1.00e+00 |
|  | 20 | 45179226 | 45179413 | PDFSTATSENSEMBLUCSC | 188 | 5 | 25754 | tss | 0.55196 | 0.83267 | Not queried | 1.69 | 5.56e-02 | 1.00e+00 |
|  | 22 | 41956810 | 41956987 | PDFSTATSENSEMBLUCSC | 178 | 4 | 27133 | tss | 0.32381 | 0.58251 | Not queried | 1.69 | 2.86e-02 | 1.00e+00 |
|  | 5 | 93077290 | 93077405 | PDFSTATSENSEMBLUCSC | 116 | 5 | 31635 | gene | 0.66064 | 0.79325 | Not queried | 1.69 | 1.51e-01 | 1.00e+00 |
|  | 6 | 152957910 | 152958664 | PDFSTATSENSEMBLUCSC | 755 | 15 | 36518 | island | 0.07246 | 0.05526 | Not queried | 1.69 | 5.12e-01 | 1.00e+00 |
|  | 12 | 14518391 | 14518974 | PDFSTATSENSEMBLUCSC | 584 | 9 | 10237 | island | 0.10071 | 0.26498 | Not queried | 1.69 | 1.61e-01 | 1.00e+00 |
|  | 6 | 126071099 | 126071538 | PDFSTATSENSEMBLUCSC | 440 | 5 | 34401 | gene;island | 0.06528 | 0.03151 | Not queried | 1.69 | 1.59e-02 | 1.00e+00 |
|  | 17 | 31254544 | 31254878 | PDFSTATSENSEMBLUCSC | 335 | 8 | 17224 | tss;island | 0.26206 | 0.05604 | Not queried | 1.69 | 2.81e-02 | 1.00e+00 |
|  | 13 | 53423940 | 53424127 | PDFSTATSENSEMBLUCSC | 188 | 4 | 11438 | tss;island | 0.23823 | 0.10877 | Not queried | 1.69 | 2.00e-01 | 1.00e+00 |
|  | 13 | 70682525 | 70682771 | PDFSTATSENSEMBLUCSC | 247 | 5 | 11148 | gene;island | 0.16034 | 0.38059 | Not queried | 1.69 | 1.59e-02 | 1.00e+00 |
|  | 6 | 33040535 | 33040914 | PDFSTATSENSEMBLUCSC | 380 | 5 | 34118 | gene | 0.74502 | 0.47593 | Not queried | 1.69 | 3.10e-01 | 1.00e+00 |
|  | 8 | 91803657 | 91803805 | PDFSTATSENSEMBLUCSC | 149 | 5 | 39743 | tss;island | 0.06896 | 0.19241 | Not queried | 1.69 | 9.52e-02 | 1.00e+00 |
|  | 14 | 105936300 | 105936715 | PDFSTATSENSEMBLUCSC | 416 | 4 | 12237 | gene;island | 0.71582 | 0.92242 | Not queried | 1.68 | 2.00e-01 | 1.00e+00 |
|  | 19 | 34918900 | 34919411 | PDFSTATSENSEMBLUCSC | 512 | 8 | 21851 | island | 0.01162 | 0.02231 | Not queried | 1.68 | 1.61e-01 | 1.00e+00 |
|  | 11 | 60681193 | 60681305 | PDFSTATSENSEMBLUCSC | 113 | 6 | 7304 | tss;island | 0.00619 | 0.01960 | Not queried | 1.68 | 4.11e-02 | 1.00e+00 |
|  | 7 | 2559692 | 2560104 | PDFSTATSENSEMBLUCSC | 413 | 4 | 36741 | gene;island | 0.11149 | 0.03004 | Not queried | 1.68 | 4.86e-01 | 1.00e+00 |
|  | 6 | 43252858 | 43253153 | PDFSTATSENSEMBLUCSC | 296 | 4 | 34282 | gene;island | 0.72392 | 0.88535 | Not queried | 1.68 | 2.86e-02 | 1.00e+00 |
|  | 12 | 133424221 | 133424709 | PDFSTATSENSEMBLUCSC | 489 | 7 | 9441 | gene | 0.80122 | 0.85982 | Not queried | 1.68 | 1.28e-01 | 1.00e+00 |
|  | 2 | 662602 | 663024 | PDFSTATSENSEMBLUCSC | 423 | 4 | 23972 | island | 0.59699 | 0.79035 | Not queried | 1.68 | 3.43e-01 | 1.00e+00 |
|  | 1 | 153747551 | 153747728 | PDFSTATSENSEMBLUCSC | 178 | 6 | 1949 | tss | 0.04188 | 0.13057 | Not queried | 1.68 | 2.60e-02 | 1.00e+00 |
|  | 11 | 72353049 | 72353604 | PDFSTATSENSEMBLUCSC | 556 | 10 | 6795 | gene;island | 0.16906 | 0.08271 | Not queried | 1.68 | 2.88e-02 | 1.00e+00 |
|  | 1 | 26516478 | 26516623 | PDFSTATSENSEMBLUCSC | 146 | 4 | 1382 | tss | 0.39835 | 0.70687 | Not queried | 1.68 | 2.86e-02 | 1.00e+00 |
|  | 22 | 46659317 | 46659595 | PDFSTATSENSEMBLUCSC | 279 | 4 | 27190 | tss;island | 0.95545 | 0.86992 | Not queried | 1.68 | 2.00e-01 | 1.00e+00 |
|  | 6 | 32909282 | 32909523 | PDFSTATSENSEMBLUCSC | 242 | 5 | 34817 | tss | 0.93240 | 0.72923 | Not queried | 1.68 | 2.22e-01 | 1.00e+00 |
|  | 1 | 164545553 | 164545843 | PDFSTATSENSEMBLUCSC | 291 | 4 | 860 | gene;island | 0.69559 | 0.37196 | Not queried | 1.68 | 2.86e-02 | 1.00e+00 |
|  | 15 | 47476133 | 47476482 | PDFSTATSENSEMBLUCSC | 350 | 6 | 14090 | island | 0.32161 | 0.10382 | Not queried | 1.68 | 1.80e-01 | 1.00e+00 |
|  | 8 | 38326209 | 38326730 | PDFSTATSENSEMBLUCSC | 522 | 8 | 40169 | island | 0.15173 | 0.03107 | Not queried | 1.68 | 1.61e-01 | 1.00e+00 |
|  | 6 | 28863395 | 28863544 | PDFSTATSENSEMBLUCSC | 150 | 4 | 35538 | island | 0.01612 | 0.03562 | Not queried | 1.68 | 5.71e-02 | 1.00e+00 |
|  | 17 | 46669455 | 46669674 | PDFSTATSENSEMBLUCSC | 220 | 7 | 17430 | tss | 0.86838 | 0.65297 | Not queried | 1.68 | 2.62e-02 | 1.00e+00 |
|  | 13 | 100636183 | 100636299 | PDFSTATSENSEMBLUCSC | 117 | 4 | 11181 | gene;island | 0.04788 | 0.00863 | Not queried | 1.68 | 1.14e-01 | 1.00e+00 |
|  | 7 | 25989524 | 25989763 | PDFSTATSENSEMBLUCSC | 240 | 4 | 38263 | island | 0.01020 | 0.04392 | Not queried | 1.68 | 3.43e-01 | 1.00e+00 |
|  | 19 | 7735239 | 7735510 | PDFSTATSENSEMBLUCSC | 272 | 4 | 21425 | island | 0.11620 | 0.20474 | Not queried | 1.68 | 1.14e-01 | 1.00e+00 |
|  | 6 | 42858647 | 42859023 | PDFSTATSENSEMBLUCSC | 377 | 8 | 34919 | tss | 0.01470 | 0.02697 | Not queried | 1.68 | 2.79e-01 | 1.00e+00 |
|  | 17 | 54675001 | 54675231 | PDFSTATSENSEMBLUCSC | 231 | 4 | 18544 | island | 0.10528 | 0.06205 | Not queried | 1.68 | 2.00e-01 | 1.00e+00 |
|  | 12 | 130821453 | 130821607 | PDFSTATSENSEMBLUCSC | 155 | 4 | 10093 | tss;island | 0.94388 | 0.81099 | Not queried | 1.68 | 2.00e-01 | 1.00e+00 |
|  | 15 | 52311035 | 52311380 | PDFSTATSENSEMBLUCSC | 346 | 4 | 13648 | tss | 0.09185 | 0.04459 | Not queried | 1.68 | 4.86e-01 | 1.00e+00 |
|  | 3 | 128336483 | 128337044 | PDFSTATSENSEMBLUCSC | 562 | 8 | 29413 | island | 0.06838 | 0.20911 | Not queried | 1.68 | 6.99e-03 | 1.00e+00 |
|  | 18 | 74729064 | 74729251 | PDFSTATSENSEMBLUCSC | 188 | 6 | 19295 | tss | 0.81820 | 0.93965 | Not queried | 1.67 | 4.11e-02 | 1.00e+00 |
|  | 5 | 172386517 | 172387101 | PDFSTATSENSEMBLUCSC | 585 | 8 | 31840 | gene | 0.05888 | 0.01613 | Not queried | 1.67 | 8.30e-02 | 1.00e+00 |
|  | 3 | 45066580 | 45066971 | PDFSTATSENSEMBLUCSC | 392 | 4 | 28224 | tss | 0.71658 | 0.87039 | Not queried | 1.67 | 5.71e-02 | 1.00e+00 |
|  | 16 | 474271 | 474528 | PDFSTATSENSEMBLUCSC | 258 | 4 | 14936 | tss;island | 0.93481 | 0.77527 | Not queried | 1.67 | 2.00e-01 | 1.00e+00 |
|  | 13 | 51483748 | 51484248 | PDFSTATSENSEMBLUCSC | 501 | 9 | 11717 | island | 0.01780 | 0.05135 | Not queried | 1.67 | 9.39e-02 | 1.00e+00 |
|  | 6 | 41470812 | 41471064 | PDFSTATSENSEMBLUCSC | 253 | 4 | 36060 | island | 0.14705 | 0.07428 | Not queried | 1.67 | 3.43e-01 | 1.00e+00 |
|  | 1 | 3775078 | 3775334 | PDFSTATSENSEMBLUCSC | 257 | 4 | 148 | gene;island | 0.80816 | 0.50140 | Not queried | 1.67 | 3.43e-01 | 1.00e+00 |
|  | 11 | 43701985 | 43702128 | PDFSTATSENSEMBLUCSC | 144 | 5 | 7234 | tss | 0.02225 | 0.06562 | Not queried | 1.67 | 9.52e-02 | 1.00e+00 |
|  | 20 | 22566877 | 22567170 | PDFSTATSENSEMBLUCSC | 294 | 5 | 25595 | tss;island | 0.39191 | 0.68573 | Not queried | 1.67 | 1.51e-01 | 1.00e+00 |
|  | 10 | 18629497 | 18629677 | PDFSTATSENSEMBLUCSC | 181 | 5 | 4509 | gene | 0.38176 | 0.08063 | Not queried | 1.67 | 5.56e-02 | 1.00e+00 |
|  | 5 | 1666974 | 1667258 | PDFSTATSENSEMBLUCSC | 285 | 4 | 32579 | island | 0.21313 | 0.06265 | Not queried | 1.67 | 4.86e-01 | 1.00e+00 |
|  | 3 | 120170383 | 120170646 | PDFSTATSENSEMBLUCSC | 264 | 4 | 28485 | tss;island | 0.74197 | 0.46482 | Not queried | 1.67 | 2.00e-01 | 1.00e+00 |
|  | 10 | 134944446 | 134945099 | PDFSTATSENSEMBLUCSC | 654 | 6 | 4870 | gene;island | 0.56757 | 0.80163 | Not queried | 1.67 | 2.60e-02 | 1.00e+00 |
|  | 7 | 94953653 | 94954202 | PDFSTATSENSEMBLUCSC | 550 | 9 | 38613 | island | 0.61852 | 0.81183 | Not queried | 1.67 | 3.15e-02 | 1.00e+00 |
|  | 19 | 13616871 | 13617094 | PDFSTATSENSEMBLUCSC | 224 | 4 | 19846 | gene;island | 0.06174 | 0.05747 | Not queried | 1.67 | 6.86e-01 | 1.00e+00 |
|  | 5 | 42949542 | 42950152 | PDFSTATSENSEMBLUCSC | 611 | 6 | 32699 | island | 0.95288 | 0.88476 | Not queried | 1.67 | 1.52e-02 | 1.00e+00 |
|  | 16 | 84163803 | 84163961 | PDFSTATSENSEMBLUCSC | 159 | 4 | 14832 | gene | 0.62779 | 0.83123 | Not queried | 1.67 | 4.86e-01 | 1.00e+00 |
|  | 7 | 54609587 | 54609968 | PDFSTATSENSEMBLUCSC | 382 | 8 | 37536 | tss | 0.01547 | 0.05231 | Not queried | 1.67 | 6.50e-02 | 1.00e+00 |
|  | 7 | 156811252 | 156811655 | PDFSTATSENSEMBLUCSC | 404 | 6 | 39031 | island | 0.12019 | 0.03898 | Not queried | 1.67 | 1.32e-01 | 1.00e+00 |
|  | 11 | 2189562 | 2189856 | PDFSTATSENSEMBLUCSC | 295 | 6 | 6434 | gene;island | 0.64841 | 0.80993 | Not queried | 1.67 | 6.49e-02 | 1.00e+00 |
|  | 2 | 240868184 | 240868631 | PDFSTATSENSEMBLUCSC | 448 | 5 | 25226 | island | 0.54333 | 0.86372 | Not queried | 1.67 | 3.17e-02 | 1.00e+00 |
|  | 22 | 21986976 | 21987048 | PDFSTATSENSEMBLUCSC | 73 | 5 | 26955 | tss | 0.49850 | 0.67596 | Not queried | 1.67 | 5.56e-02 | 1.00e+00 |
|  | 7 | 2701419 | 2701802 | PDFSTATSENSEMBLUCSC | 384 | 5 | 36749 | gene;island | 0.92674 | 0.92920 | Not queried | 1.67 | 4.21e-01 | 1.00e+00 |
|  | 5 | 172655841 | 172656166 | PDFSTATSENSEMBLUCSC | 326 | 6 | 33341 | island | 0.63037 | 0.84884 | Not queried | 1.67 | 8.66e-03 | 1.00e+00 |
|  | 12 | 122356316 | 122356852 | PDFSTATSENSEMBLUCSC | 537 | 6 | 10896 | island | 0.78892 | 0.88224 | Not queried | 1.66 | 1.32e-01 | 1.00e+00 |
|  | 12 | 49782966 | 49783222 | PDFSTATSENSEMBLUCSC | 257 | 5 | 9094 | gene;island | 0.46469 | 0.77572 | Not queried | 1.66 | 9.52e-02 | 1.00e+00 |
|  | 7 | 150778201 | 150778340 | PDFSTATSENSEMBLUCSC | 140 | 5 | 37223 | gene | 0.10499 | 0.18599 | Not queried | 1.66 | 1.51e-01 | 1.00e+00 |
|  | 1 | 198828157 | 198828249 | PDFSTATSENSEMBLUCSC | 93 | 4 | 2155 | tss | 0.95347 | 0.86300 | Not queried | 1.66 | 3.43e-01 | 1.00e+00 |
|  | 7 | 27191914 | 27192656 | PDFSTATSENSEMBLUCSC | 743 | 8 | 38287 | island | 0.26971 | 0.62053 | Not queried | 1.66 | 1.48e-02 | 1.00e+00 |
|  | 12 | 79198115 | 79198467 | PDFSTATSENSEMBLUCSC | 353 | 4 | 10618 | island | 0.54958 | 0.75817 | Not queried | 1.66 | 3.43e-01 | 1.00e+00 |
|  | 2 | 177014467 | 177015125 | PDFSTATSENSEMBLUCSC | 659 | 13 | 23702 | tss;island | 0.82583 | 0.61181 | Not queried | 1.66 | 7.23e-02 | 1.00e+00 |
|  | 13 | 102104573 | 102104848 | PDFSTATSENSEMBLUCSC | 276 | 4 | 11492 | tss | 0.48742 | 0.74106 | Not queried | 1.66 | 5.71e-02 | 1.00e+00 |
|  | X | 30233227 | 30233603 | PDFSTATSENSEMBLUCSC | 377 | 8 | 41242 | tss | 0.82248 | 0.94188 | Not queried | 1.66 | 6.99e-03 | 1.00e+00 |
|  | 12 | 8332640 | 8332802 | PDFSTATSENSEMBLUCSC | 163 | 4 | 9528 | tss | 0.03397 | 0.08107 | Not queried | 1.66 | 3.43e-01 | 1.00e+00 |
|  | 17 | 65471303 | 65471507 | PDFSTATSENSEMBLUCSC | 205 | 5 | 16702 | gene | 0.94611 | 0.91908 | Not queried | 1.66 | 3.10e-01 | 1.00e+00 |
|  | 12 | 133019854 | 133020030 | PDFSTATSENSEMBLUCSC | 177 | 4 | 11029 | island | 0.73459 | 0.89460 | Not queried | 1.66 | 1.14e-01 | 1.00e+00 |
|  | 2 | 177027291 | 177027931 | PDFSTATSENSEMBLUCSC | 641 | 7 | 24872 | island | 0.80171 | 0.50501 | Not queried | 1.66 | 5.30e-02 | 1.00e+00 |
|  | 6 | 11111894 | 11112237 | PDFSTATSENSEMBLUCSC | 344 | 5 | 33505 | gene | 0.53552 | 0.82242 | Not queried | 1.66 | 3.17e-02 | 1.00e+00 |
|  | 7 | 6191780 | 6192094 | PDFSTATSENSEMBLUCSC | 315 | 6 | 36822 | gene;island | 0.79791 | 0.91170 | Not queried | 1.66 | 4.11e-02 | 1.00e+00 |
|  | 5 | 139060476 | 139060922 | PDFSTATSENSEMBLUCSC | 447 | 6 | 31723 | gene;island | 0.62958 | 0.85324 | Not queried | 1.66 | 2.16e-03 | 1.00e+00 |
|  | 7 | 32996733 | 32996881 | PDFSTATSENSEMBLUCSC | 149 | 6 | 37464 | tss;island | 0.11337 | 0.03322 | Not queried | 1.66 | 4.11e-02 | 1.00e+00 |
|  | 13 | 114108433 | 114108713 | PDFSTATSENSEMBLUCSC | 281 | 6 | 11524 | tss | 0.79494 | 0.90931 | Not queried | 1.66 | 2.60e-02 | 1.00e+00 |
|  | 1 | 154539974 | 154540535 | PDFSTATSENSEMBLUCSC | 562 | 8 | 3783 | island | 0.44003 | 0.18350 | Not queried | 1.66 | 4.42e-01 | 1.00e+00 |
|  | 7 | 150644459 | 150644866 | PDFSTATSENSEMBLUCSC | 408 | 5 | 37215 | gene;island | 0.44487 | 0.71641 | Not queried | 1.66 | 3.10e-01 | 1.00e+00 |
|  | 10 | 134411295 | 134411480 | PDFSTATSENSEMBLUCSC | 186 | 4 | 4851 | gene;island | 0.96179 | 0.91730 | Not queried | 1.66 | 1.14e-01 | 1.00e+00 |
|  | 21 | 37432620 | 37432782 | PDFSTATSENSEMBLUCSC | 163 | 4 | 26492 | tss | 0.01878 | 0.02891 | Not queried | 1.66 | 4.86e-01 | 1.00e+00 |
|  | 6 | 144385771 | 144386528 | PDFSTATSENSEMBLUCSC | 758 | 20 | 35206 | tss | 0.86331 | 0.51765 | Not queried | 1.66 | 3.50e-02 | 1.00e+00 |
|  | 7 | 75956931 | 75957376 | PDFSTATSENSEMBLUCSC | 446 | 7 | 36991 | gene;island | 0.86586 | 0.94673 | Not queried | 1.66 | 7.28e-02 | 1.00e+00 |
|  | X | 75005076 | 75005131 | PDFSTATSENSEMBLUCSC | 56 | 5 | 41397 | tss | 0.09152 | 0.37602 | Not queried | 1.65 | 3.17e-02 | 1.00e+00 |
|  | 19 | 52004992 | 52005188 | PDFSTATSENSEMBLUCSC | 197 | 6 | 20960 | tss | 0.24828 | 0.50251 | Not queried | 1.65 | 2.60e-02 | 1.00e+00 |
|  | 5 | 167719478 | 167719742 | PDFSTATSENSEMBLUCSC | 265 | 4 | 31825 | gene;island | 0.03042 | 0.09558 | Not queried | 1.65 | 2.86e-02 | 1.00e+00 |
|  | 8 | 143333074 | 143333834 | PDFSTATSENSEMBLUCSC | 761 | 7 | 39392 | gene;island | 0.56210 | 0.81708 | Not queried | 1.65 | 1.75e-02 | 1.00e+00 |
|  | 3 | 138153127 | 138153381 | PDFSTATSENSEMBLUCSC | 255 | 8 | 28580 | tss | 0.19519 | 0.07584 | Not queried | 1.65 | 2.34e-01 | 1.00e+00 |
|  | 16 | 58497230 | 58497395 | PDFSTATSENSEMBLUCSC | 166 | 4 | 15241 | tss;island | 0.33681 | 0.06219 | Not queried | 1.65 | 3.43e-01 | 1.00e+00 |
|  | 12 | 48398320 | 48398730 | PDFSTATSENSEMBLUCSC | 411 | 7 | 9640 | tss;island | 0.12749 | 0.04136 | Not queried | 1.65 | 3.79e-02 | 1.00e+00 |
|  | 19 | 47778045 | 47778150 | PDFSTATSENSEMBLUCSC | 106 | 4 | 22157 | island | 0.01744 | 0.08359 | Not queried | 1.65 | 1.14e-01 | 1.00e+00 |
|  | 20 | 62716332 | 62716606 | PDFSTATSENSEMBLUCSC | 275 | 4 | 25481 | gene;tss;island | 0.79923 | 0.53472 | Not queried | 1.65 | 5.71e-02 | 1.00e+00 |
|  | 16 | 66878426 | 66879016 | PDFSTATSENSEMBLUCSC | 591 | 11 | 14753 | gene;island | 0.09547 | 0.03127 | Not queried | 1.65 | 2.75e-04 | 1.00e+00 |
|  | 16 | 66878426 | 66878810 | PDFSTATSENSEMBLUCSC | 385 | 9 | 15255 | tss | 0.09002 | 0.02797 | Not queried | 1.65 | 2.88e-04 | 1.00e+00 |
|  | 1 | 153600762 | 153600972 | PDFSTATSENSEMBLUCSC | 211 | 4 | 761 | gene;tss | 0.21510 | 0.47392 | Not queried | 1.65 | 3.43e-01 | 1.00e+00 |
|  | 18 | 56529784 | 56529878 | PDFSTATSENSEMBLUCSC | 95 | 5 | 19262 | tss;island | 0.12915 | 0.02393 | Not queried | 1.65 | 5.56e-02 | 1.00e+00 |
|  | 2 | 213400697 | 213401333 | PDFSTATSENSEMBLUCSC | 637 | 5 | 22968 | gene;island | 0.01893 | 0.02902 | Not queried | 1.65 | 5.48e-01 | 1.00e+00 |
|  | 19 | 10602587 | 10602960 | PDFSTATSENSEMBLUCSC | 374 | 4 | 19777 | gene;island | 0.74709 | 0.88150 | Not queried | 1.65 | 8.86e-01 | 1.00e+00 |
|  | 18 | 55862577 | 55862872 | PDFSTATSENSEMBLUCSC | 296 | 7 | 19087 | gene;island | 0.66868 | 0.84658 | Not queried | 1.65 | 1.11e-02 | 1.00e+00 |
|  | 6 | 29923913 | 29924475 | PDFSTATSENSEMBLUCSC | 563 | 13 | 35583 | island | 0.32372 | 0.64307 | Not queried | 1.65 | 1.55e-03 | 1.00e+00 |
|  | 11 | 1463541 | 1463662 | PDFSTATSENSEMBLUCSC | 122 | 4 | 6401 | gene;island | 0.41047 | 0.69190 | Not queried | 1.65 | 1.14e-01 | 1.00e+00 |
|  | 4 | 26321136 | 26321356 | PDFSTATSENSEMBLUCSC | 221 | 8 | 30288 | tss;island | 0.04119 | 0.01498 | Not queried | 1.65 | 1.09e-03 | 1.00e+00 |
|  | 18 | 51750950 | 51751391 | PDFSTATSENSEMBLUCSC | 442 | 7 | 19488 | island | 0.01555 | 0.02761 | Not queried | 1.65 | 2.59e-01 | 1.00e+00 |
|  | 8 | 38126881 | 38127091 | PDFSTATSENSEMBLUCSC | 211 | 8 | 39592 | tss;island | 0.01210 | 0.03926 | Not queried | 1.65 | 1.48e-02 | 1.00e+00 |
|  | 4 | 5021203 | 5021328 | PDFSTATSENSEMBLUCSC | 126 | 5 | 30225 | tss | 0.62971 | 0.42537 | Not queried | 1.65 | 7.94e-03 | 1.00e+00 |
|  | 5 | 145722132 | 145722517 | PDFSTATSENSEMBLUCSC | 386 | 5 | 33220 | island | 0.30270 | 0.49337 | Not queried | 1.65 | 7.94e-03 | 1.00e+00 |
|  | 17 | 22051978 | 22052191 | PDFSTATSENSEMBLUCSC | 214 | 4 | 18097 | island | 0.22300 | 0.48162 | Not queried | 1.65 | 5.71e-02 | 1.00e+00 |
|  | 17 | 35732737 | 35732945 | PDFSTATSENSEMBLUCSC | 209 | 4 | 16496 | gene;tss | 0.87643 | 0.95805 | Not queried | 1.65 | 2.00e-01 | 1.00e+00 |
|  | 12 | 54422127 | 54422488 | PDFSTATSENSEMBLUCSC | 362 | 5 | 9134 | gene;island | 0.01847 | 0.03376 | Not queried | 1.64 | 5.48e-01 | 1.00e+00 |
|  | 19 | 34918900 | 34919248 | PDFSTATSENSEMBLUCSC | 349 | 7 | 20656 | tss | 0.01664 | 0.02143 | Not queried | 1.64 | 2.59e-01 | 1.00e+00 |
|  | 17 | 53342593 | 53342740 | PDFSTATSENSEMBLUCSC | 148 | 4 | 16665 | gene;island | 0.01701 | 0.05427 | Not queried | 1.64 | 1.14e-01 | 1.00e+00 |
|  | 2 | 27505396 | 27505593 | PDFSTATSENSEMBLUCSC | 198 | 5 | 23224 | tss | 0.56761 | 0.25219 | Not queried | 1.64 | 7.94e-03 | 1.00e+00 |
|  | 5 | 172260464 | 172260815 | PDFSTATSENSEMBLUCSC | 352 | 4 | 32436 | tss;island | 0.28482 | 0.07410 | Not queried | 1.64 | 4.86e-01 | 1.00e+00 |
|  | 6 | 33384179 | 33384537 | PDFSTATSENSEMBLUCSC | 359 | 7 | 34216 | gene;island | 0.68112 | 0.91011 | Not queried | 1.64 | 5.30e-02 | 1.00e+00 |
|  | 7 | 8483249 | 8483710 | PDFSTATSENSEMBLUCSC | 462 | 4 | 36830 | gene;island | 0.02370 | 0.06640 | Not queried | 1.64 | 2.00e-01 | 1.00e+00 |
|  | 11 | 58830191 | 58830610 | PDFSTATSENSEMBLUCSC | 420 | 4 | 8278 | island | 0.45531 | 0.73054 | Not queried | 1.64 | 2.86e-02 | 1.00e+00 |
|  | 3 | 118892068 | 118892330 | PDFSTATSENSEMBLUCSC | 263 | 4 | 28474 | tss | 0.30237 | 0.11505 | Not queried | 1.64 | 2.00e-01 | 1.00e+00 |
|  | 12 | 48152204 | 48152585 | PDFSTATSENSEMBLUCSC | 382 | 6 | 9080 | gene;tss;island | 0.42587 | 0.18385 | Not queried | 1.64 | 1.80e-01 | 1.00e+00 |
|  | 12 | 1940380 | 1940618 | PDFSTATSENSEMBLUCSC | 239 | 4 | 8976 | gene | 0.09243 | 0.23111 | Not queried | 1.64 | 2.86e-02 | 1.00e+00 |
|  | 20 | 43729808 | 43730241 | PDFSTATSENSEMBLUCSC | 434 | 9 | 25729 | tss;island | 0.33253 | 0.07822 | Not queried | 1.64 | 6.25e-02 | 1.00e+00 |
|  | 1 | 236228598 | 236228837 | PDFSTATSENSEMBLUCSC | 240 | 6 | 2335 | tss;island | 0.11111 | 0.02810 | Not queried | 1.64 | 2.40e-01 | 1.00e+00 |
|  | 19 | 6534760 | 6535078 | PDFSTATSENSEMBLUCSC | 319 | 5 | 19731 | gene;island | 0.92130 | 0.84986 | Not queried | 1.64 | 1.59e-02 | 1.00e+00 |
|  | 10 | 118084109 | 118084587 | PDFSTATSENSEMBLUCSC | 479 | 6 | 4760 | gene | 0.50910 | 0.74227 | Not queried | 1.64 | 1.52e-02 | 1.00e+00 |
|  | 5 | 87968528 | 87968749 | PDFSTATSENSEMBLUCSC | 222 | 4 | 31620 | gene;island | 0.14687 | 0.04107 | Not queried | 1.64 | 2.00e-01 | 1.00e+00 |
|  | 13 | 114220436 | 114220724 | PDFSTATSENSEMBLUCSC | 289 | 4 | 11943 | island | 0.68403 | 0.87137 | Not queried | 1.64 | 5.71e-02 | 1.00e+00 |
|  | 11 | 8832817 | 8833095 | PDFSTATSENSEMBLUCSC | 279 | 6 | 6482 | gene;tss | 0.58346 | 0.30504 | Not queried | 1.64 | 2.16e-03 | 1.00e+00 |
|  | 1 | 3331987 | 3332264 | PDFSTATSENSEMBLUCSC | 278 | 6 | 124 | gene;island | 0.58588 | 0.74273 | Not queried | 1.64 | 2.16e-03 | 1.00e+00 |
|  | 1 | 155290236 | 155290614 | PDFSTATSENSEMBLUCSC | 379 | 7 | 1992 | tss | 0.10960 | 0.25611 | Not queried | 1.64 | 3.18e-01 | 1.00e+00 |
|  | 14 | 89259551 | 89259748 | PDFSTATSENSEMBLUCSC | 198 | 4 | 12519 | tss;island | 0.04002 | 0.18986 | Not queried | 1.64 | 3.43e-01 | 1.00e+00 |
|  | 2 | 11809681 | 11810183 | PDFSTATSENSEMBLUCSC | 503 | 5 | 22537 | gene | 0.11813 | 0.27273 | Not queried | 1.64 | 4.21e-01 | 1.00e+00 |
|  | 6 | 31549480 | 31549631 | PDFSTATSENSEMBLUCSC | 152 | 4 | 33843 | gene;island | 0.66688 | 0.42118 | Not queried | 1.64 | 3.43e-01 | 1.00e+00 |
|  | 4 | 99064459 | 99064904 | PDFSTATSENSEMBLUCSC | 446 | 7 | 30460 | tss | 0.01127 | 0.03424 | Not queried | 1.64 | 1.65e-01 | 1.00e+00 |
|  | 16 | 53407421 | 53407808 | PDFSTATSENSEMBLUCSC | 388 | 7 | 15892 | island | 0.34412 | 0.64560 | Not queried | 1.64 | 2.33e-03 | 1.00e+00 |
|  | 17 | 29886672 | 29887049 | PDFSTATSENSEMBLUCSC | 378 | 5 | 18155 | island | 0.44708 | 0.68640 | Not queried | 1.64 | 7.94e-03 | 1.00e+00 |
|  | 15 | 78632572 | 78632861 | PDFSTATSENSEMBLUCSC | 290 | 8 | 14324 | island | 0.28015 | 0.50222 | Not queried | 1.64 | 4.66e-03 | 1.00e+00 |
|  | 2 | 87088796 | 87088843 | PDFSTATSENSEMBLUCSC | 48 | 4 | 22736 | gene;island | 0.57758 | 0.80659 | Not queried | 1.64 | 2.86e-02 | 1.00e+00 |
|  | 7 | 127670993 | 127671195 | PDFSTATSENSEMBLUCSC | 203 | 6 | 37147 | gene;island | 0.08400 | 0.03329 | Not queried | 1.64 | 4.11e-02 | 1.00e+00 |
|  | 5 | 5139334 | 5140029 | PDFSTATSENSEMBLUCSC | 696 | 17 | 31920 | tss;island | 0.70943 | 0.41940 | Not queried | 1.64 | 1.40e-01 | 1.00e+00 |
|  | 6 | 31866508 | 31866825 | PDFSTATSENSEMBLUCSC | 318 | 4 | 34786 | tss;island | 0.25109 | 0.46706 | Not queried | 1.63 | 8.86e-01 | 1.00e+00 |
|  | 7 | 100823700 | 100823790 | PDFSTATSENSEMBLUCSC | 91 | 4 | 37735 | tss;island | 0.36607 | 0.15509 | Not queried | 1.63 | 1.14e-01 | 1.00e+00 |
|  | 11 | 32459428 | 32459954 | PDFSTATSENSEMBLUCSC | 527 | 4 | 6551 | gene;island | 0.16873 | 0.08582 | Not queried | 1.63 | 3.43e-01 | 1.00e+00 |
|  | 1 | 3028813 | 3029070 | PDFSTATSENSEMBLUCSC | 258 | 6 | 99 | gene;island | 0.70999 | 0.87204 | Not queried | 1.63 | 2.60e-02 | 1.00e+00 |
|  | 11 | 19367501 | 19368277 | PDFSTATSENSEMBLUCSC | 777 | 8 | 8099 | island | 0.10777 | 0.27030 | Not queried | 1.63 | 6.50e-02 | 1.00e+00 |
|  | 13 | 112712424 | 112712795 | PDFSTATSENSEMBLUCSC | 372 | 4 | 11878 | island | 0.42603 | 0.26657 | Not queried | 1.63 | 4.86e-01 | 1.00e+00 |
|  | 10 | 134977981 | 134978147 | PDFSTATSENSEMBLUCSC | 167 | 5 | 4871 | gene;island | 0.66303 | 0.87365 | Not queried | 1.63 | 9.52e-02 | 1.00e+00 |
|  | 17 | 39019545 | 39020053 | PDFSTATSENSEMBLUCSC | 509 | 6 | 16533 | gene;island | 0.45903 | 0.70880 | Not queried | 1.63 | 1.80e-01 | 1.00e+00 |
|  | 6 | 32086755 | 32087190 | PDFSTATSENSEMBLUCSC | 436 | 12 | 33991 | gene | 0.27251 | 0.46857 | Not queried | 1.63 | 5.97e-02 | 1.00e+00 |
|  | 19 | 58446312 | 58446669 | PDFSTATSENSEMBLUCSC | 358 | 4 | 20217 | gene | 0.04161 | 0.11891 | Not queried | 1.63 | 2.00e-01 | 1.00e+00 |
|  | 6 | 33400477 | 33400543 | PDFSTATSENSEMBLUCSC | 67 | 4 | 34226 | gene | 0.72720 | 0.89327 | Not queried | 1.63 | 5.71e-02 | 1.00e+00 |
|  | 6 | 29364418 | 29365287 | PDFSTATSENSEMBLUCSC | 870 | 11 | 33591 | gene | 0.46583 | 0.79273 | Not queried | 1.63 | 4.73e-02 | 1.00e+00 |
|  | 17 | 38599271 | 38599508 | PDFSTATSENSEMBLUCSC | 238 | 4 | 17301 | tss | 0.10602 | 0.05807 | Not queried | 1.63 | 4.86e-01 | 1.00e+00 |
|  | 11 | 117668035 | 117668336 | PDFSTATSENSEMBLUCSC | 302 | 8 | 7700 | tss | 0.44787 | 0.23213 | Not queried | 1.63 | 1.30e-01 | 1.00e+00 |
|  | 12 | 132976722 | 132977140 | PDFSTATSENSEMBLUCSC | 419 | 4 | 11021 | island | 0.42008 | 0.70950 | Not queried | 1.63 | 5.71e-02 | 1.00e+00 |
|  | 17 | 38249266 | 38249729 | PDFSTATSENSEMBLUCSC | 464 | 6 | 16524 | gene;island | 0.66051 | 0.88565 | Not queried | 1.63 | 1.32e-01 | 1.00e+00 |
|  | 18 | 21452730 | 21452895 | PDFSTATSENSEMBLUCSC | 166 | 6 | 19042 | gene;tss | 0.57022 | 0.84036 | Not queried | 1.63 | 2.60e-02 | 1.00e+00 |
|  | 22 | 38071001 | 38071188 | PDFSTATSENSEMBLUCSC | 188 | 4 | 27078 | tss;island | 0.13773 | 0.32499 | Not queried | 1.63 | 2.86e-02 | 1.00e+00 |
|  | 3 | 193587490 | 193587939 | PDFSTATSENSEMBLUCSC | 450 | 4 | 29734 | island | 0.58926 | 0.80841 | Not queried | 1.63 | 2.00e-01 | 1.00e+00 |
|  | 10 | 77165618 | 77165905 | PDFSTATSENSEMBLUCSC | 288 | 4 | 4631 | gene;island | 0.71300 | 0.53851 | Not queried | 1.63 | 3.43e-01 | 1.00e+00 |
|  | 1 | 203242409 | 203242638 | PDFSTATSENSEMBLUCSC | 230 | 4 | 4088 | island | 0.05677 | 0.20576 | Not queried | 1.63 | 3.43e-01 | 1.00e+00 |
|  | 10 | 102893925 | 102894639 | PDFSTATSENSEMBLUCSC | 715 | 8 | 4710 | gene;island | 0.55757 | 0.79745 | Not queried | 1.63 | 1.55e-04 | 7.99e-01 |
|  | 1 | 86888585 | 86888728 | PDFSTATSENSEMBLUCSC | 144 | 4 | 1724 | tss | 0.87667 | 0.66412 | Not queried | 1.63 | 1.14e-01 | 1.00e+00 |
|  | 10 | 71892593 | 71892787 | PDFSTATSENSEMBLUCSC | 195 | 7 | 5793 | island | 0.07312 | 0.19906 | Not queried | 1.63 | 2.62e-02 | 1.00e+00 |
|  | 6 | 74072242 | 74072447 | PDFSTATSENSEMBLUCSC | 206 | 7 | 36189 | island | 0.74862 | 0.87599 | Not queried | 1.63 | 5.30e-02 | 1.00e+00 |
|  | 16 | 29757344 | 29757461 | PDFSTATSENSEMBLUCSC | 118 | 4 | 15136 | tss | 0.72582 | 0.87920 | Not queried | 1.63 | 2.00e-01 | 1.00e+00 |
|  | 17 | 28563054 | 28563300 | PDFSTATSENSEMBLUCSC | 247 | 5 | 17205 | tss;island | 0.03719 | 0.10731 | Not queried | 1.63 | 2.22e-01 | 1.00e+00 |
|  | 5 | 133451237 | 133451474 | PDFSTATSENSEMBLUCSC | 238 | 7 | 32228 | tss | 0.17086 | 0.03041 | Not queried | 1.63 | 5.30e-02 | 1.00e+00 |
|  | 8 | 23082721 | 23082961 | PDFSTATSENSEMBLUCSC | 241 | 8 | 39535 | tss | 0.11069 | 0.04791 | Not queried | 1.63 | 2.81e-02 | 1.00e+00 |
|  | 19 | 52408259 | 52408520 | PDFSTATSENSEMBLUCSC | 262 | 8 | 20964 | tss | 0.06886 | 0.02730 | Not queried | 1.63 | 4.99e-02 | 1.00e+00 |
|  | 8 | 142238691 | 142239055 | PDFSTATSENSEMBLUCSC | 365 | 6 | 39864 | tss | 0.88603 | 0.94511 | Not queried | 1.63 | 6.49e-02 | 1.00e+00 |
|  | 4 | 48988015 | 48988321 | PDFSTATSENSEMBLUCSC | 307 | 7 | 30985 | island | 0.30016 | 0.14263 | Not queried | 1.63 | 3.79e-02 | 1.00e+00 |
|  | 7 | 94953653 | 94953877 | PDFSTATSENSEMBLUCSC | 225 | 5 | 37033 | gene | 0.61852 | 0.71747 | Not queried | 1.63 | 4.21e-01 | 1.00e+00 |
|  | 17 | 59668571 | 59668616 | PDFSTATSENSEMBLUCSC | 46 | 6 | 17525 | tss | 0.63448 | 0.85894 | Not queried | 1.63 | 6.49e-02 | 1.00e+00 |
|  | 6 | 160783785 | 160784044 | PDFSTATSENSEMBLUCSC | 260 | 4 | 34470 | gene;island | 0.85808 | 0.70585 | Not queried | 1.63 | 2.86e-02 | 1.00e+00 |
|  | 1 | 223254706 | 223255040 | PDFSTATSENSEMBLUCSC | 335 | 5 | 4220 | island | 0.08357 | 0.02991 | Not queried | 1.63 | 1.51e-01 | 1.00e+00 |
|  | 16 | 69141250 | 69141437 | PDFSTATSENSEMBLUCSC | 188 | 5 | 15295 | tss | 0.10138 | 0.03527 | Not queried | 1.63 | 9.52e-02 | 1.00e+00 |
|  | 19 | 11531294 | 11531519 | PDFSTATSENSEMBLUCSC | 226 | 5 | 19804 | gene;island | 0.03673 | 0.07334 | Not queried | 1.63 | 2.22e-01 | 1.00e+00 |
|  | 6 | 30074595 | 30074764 | PDFSTATSENSEMBLUCSC | 170 | 4 | 33659 | gene;island | 0.72782 | 0.55527 | Not queried | 1.63 | 3.43e-01 | 1.00e+00 |
|  | 7 | 27207996 | 27208285 | PDFSTATSENSEMBLUCSC | 290 | 4 | 38293 | island | 0.90751 | 0.72591 | Not queried | 1.63 | 5.71e-02 | 1.00e+00 |
|  | 2 | 97523237 | 97523478 | PDFSTATSENSEMBLUCSC | 242 | 4 | 22751 | gene;island | 0.02814 | 0.02552 | Not queried | 1.63 | 6.86e-01 | 1.00e+00 |
|  | 2 | 132233726 | 132234134 | PDFSTATSENSEMBLUCSC | 409 | 5 | 22834 | gene | 0.96181 | 0.88415 | Not queried | 1.63 | 3.17e-02 | 1.00e+00 |
|  | 2 | 132233433 | 132234134 | PDFSTATSENSEMBLUCSC | 702 | 9 | 24695 | island | 0.96181 | 0.88099 | Not queried | 1.63 | 7.77e-03 | 1.00e+00 |
|  | 6 | 28192892 | 28193049 | PDFSTATSENSEMBLUCSC | 158 | 5 | 34672 | tss | 0.02296 | 0.05346 | Not queried | 1.62 | 3.10e-01 | 1.00e+00 |
|  | 21 | 46131464 | 46131923 | PDFSTATSENSEMBLUCSC | 460 | 6 | 26699 | island | 0.36765 | 0.63322 | Not queried | 1.62 | 1.52e-02 | 1.00e+00 |
|  | 11 | 2193041 | 2193293 | PDFSTATSENSEMBLUCSC | 253 | 4 | 7043 | tss | 0.47503 | 0.82592 | Not queried | 1.62 | 1.14e-01 | 1.00e+00 |
|  | 5 | 92929319 | 92929679 | PDFSTATSENSEMBLUCSC | 361 | 5 | 31633 | gene;island | 0.77202 | 0.42354 | Not queried | 1.62 | 9.52e-02 | 1.00e+00 |
|  | 13 | 114214248 | 114214740 | PDFSTATSENSEMBLUCSC | 493 | 5 | 11942 | island | 0.90237 | 0.96759 | Not queried | 1.62 | 5.56e-02 | 1.00e+00 |
|  | 14 | 60974384 | 60974525 | PDFSTATSENSEMBLUCSC | 142 | 5 | 12876 | island | 0.66508 | 0.40504 | Not queried | 1.62 | 3.10e-01 | 1.00e+00 |
|  | 1 | 5932351 | 5932678 | PDFSTATSENSEMBLUCSC | 328 | 5 | 154 | gene | 0.85515 | 0.97393 | Not queried | 1.62 | 4.21e-01 | 1.00e+00 |
|  | 21 | 34398085 | 34398226 | PDFSTATSENSEMBLUCSC | 142 | 6 | 26475 | tss | 0.01371 | 0.04953 | Not queried | 1.62 | 1.52e-02 | 1.00e+00 |
|  | 6 | 32781171 | 32781384 | PDFSTATSENSEMBLUCSC | 214 | 5 | 34067 | gene | 0.83424 | 0.50996 | Not queried | 1.62 | 2.22e-01 | 1.00e+00 |
|  | 2 | 85637673 | 85637851 | PDFSTATSENSEMBLUCSC | 179 | 4 | 24439 | island | 0.45597 | 0.68240 | Not queried | 1.62 | 2.86e-02 | 1.00e+00 |
|  | 1 | 101360652 | 101360775 | PDFSTATSENSEMBLUCSC | 124 | 4 | 1777 | tss;island | 0.04061 | 0.01607 | Not queried | 1.62 | 4.86e-01 | 1.00e+00 |
|  | X | 152954816 | 152954965 | PDFSTATSENSEMBLUCSC | 150 | 7 | 41144 | gene | 0.76859 | 0.58614 | Not queried | 1.62 | 1.17e-03 | 1.00e+00 |
|  | 6 | 30131001 | 30132226 | PDFSTATSENSEMBLUCSC | 1226 | 24 | 33673 | gene | 0.36161 | 0.63172 | TRIM15 (margin: TRIM15; TRIM10) | 1.62 | 4.20e-06 | 2.16e-02 |
|  | 2 | 242811830 | 242811962 | PDFSTATSENSEMBLUCSC | 133 | 4 | 25275 | island | 0.73400 | 0.88735 | Not queried | 1.62 | 3.43e-01 | 1.00e+00 |
|  | 8 | 144814911 | 144815275 | PDFSTATSENSEMBLUCSC | 365 | 4 | 39427 | gene;island | 0.28904 | 0.54113 | Not queried | 1.62 | 2.00e-01 | 1.00e+00 |
|  | 11 | 50257496 | 50257979 | PDFSTATSENSEMBLUCSC | 484 | 7 | 8257 | island | 0.49823 | 0.71297 | Not queried | 1.62 | 1.75e-02 | 1.00e+00 |
|  | 8 | 11471395 | 11471881 | PDFSTATSENSEMBLUCSC | 487 | 5 | 40018 | island | 0.60925 | 0.84758 | Not queried | 1.62 | 1.59e-02 | 1.00e+00 |
|  | 5 | 148521450 | 148521623 | PDFSTATSENSEMBLUCSC | 174 | 4 | 31799 | gene;island | 0.11348 | 0.27511 | Not queried | 1.62 | 2.86e-02 | 1.00e+00 |
|  | 17 | 72931569 | 72932048 | PDFSTATSENSEMBLUCSC | 480 | 12 | 18696 | island | 0.20095 | 0.45905 | Not queried | 1.62 | 6.56e-04 | 1.00e+00 |
|  | 2 | 177027440 | 177027931 | PDFSTATSENSEMBLUCSC | 492 | 6 | 23703 | tss | 0.83681 | 0.51987 | Not queried | 1.62 | 1.32e-01 | 1.00e+00 |
|  | 2 | 87304015 | 87304216 | PDFSTATSENSEMBLUCSC | 202 | 4 | 23459 | tss;island | 0.02320 | 0.05820 | Not queried | 1.62 | 2.86e-02 | 1.00e+00 |
|  | 1 | 7740243 | 7740286 | PDFSTATSENSEMBLUCSC | 44 | 4 | 186 | gene;island | 0.02128 | 0.07774 | Not queried | 1.62 | 2.00e-01 | 1.00e+00 |
|  | 19 | 19007311 | 19007647 | PDFSTATSENSEMBLUCSC | 337 | 5 | 20594 | tss;island | 0.87591 | 0.43218 | Not queried | 1.62 | 5.56e-02 | 1.00e+00 |
|  | 2 | 220159595 | 220159976 | PDFSTATSENSEMBLUCSC | 382 | 4 | 22995 | gene;tss;island | 0.03256 | 0.16515 | Not queried | 1.62 | 1.14e-01 | 1.00e+00 |
|  | 8 | 26047780 | 26048065 | PDFSTATSENSEMBLUCSC | 286 | 5 | 40108 | island | 0.19258 | 0.36168 | Not queried | 1.62 | 1.51e-01 | 1.00e+00 |
|  | 2 | 27439738 | 27439915 | PDFSTATSENSEMBLUCSC | 178 | 4 | 22587 | gene;tss;island | 0.85895 | 0.94569 | Not queried | 1.62 | 4.86e-01 | 1.00e+00 |
|  | 4 | 1004110 | 1005405 | PDFSTATSENSEMBLUCSC | 1296 | 28 | 30188 | tss;island | 0.03233 | 0.16594 | Not queried | 1.62 | 3.91e-03 | 1.00e+00 |
|  | 3 | 52351355 | 52351963 | PDFSTATSENSEMBLUCSC | 609 | 5 | 27775 | gene | 0.01781 | 0.05266 | Not queried | 1.62 | 2.22e-01 | 1.00e+00 |
|  | 10 | 77871958 | 77872458 | PDFSTATSENSEMBLUCSC | 501 | 5 | 4632 | gene;island | 0.61922 | 0.84003 | Not queried | 1.62 | 9.52e-02 | 1.00e+00 |
|  | 10 | 112289853 | 112290331 | PDFSTATSENSEMBLUCSC | 479 | 7 | 6112 | island | 0.03702 | 0.09031 | Not queried | 1.62 | 1.11e-02 | 1.00e+00 |
|  | 11 | 72982929 | 72983097 | PDFSTATSENSEMBLUCSC | 169 | 4 | 6802 | gene;tss | 0.54670 | 0.83990 | Not queried | 1.62 | 5.71e-02 | 1.00e+00 |
|  | 5 | 7851280 | 7851681 | PDFSTATSENSEMBLUCSC | 402 | 5 | 31924 | tss | 0.45809 | 0.16073 | Not queried | 1.62 | 6.90e-01 | 1.00e+00 |
|  | 11 | 85338771 | 85338971 | PDFSTATSENSEMBLUCSC | 201 | 4 | 7581 | tss;island | 0.03189 | 0.01180 | Not queried | 1.61 | 2.00e-01 | 1.00e+00 |
|  | 5 | 140602851 | 140603034 | PDFSTATSENSEMBLUCSC | 184 | 4 | 32308 | tss;island | 0.02472 | 0.03437 | Not queried | 1.61 | 6.86e-01 | 1.00e+00 |
|  | 3 | 42947263 | 42947565 | PDFSTATSENSEMBLUCSC | 303 | 5 | 28208 | tss | 0.89356 | 0.96023 | Not queried | 1.61 | 5.56e-02 | 1.00e+00 |
|  | 4 | 135865398 | 135865584 | PDFSTATSENSEMBLUCSC | 187 | 4 | 31254 | island | 0.86824 | 0.92961 | Not queried | 1.61 | 3.43e-01 | 1.00e+00 |
|  | 2 | 218898491 | 218898788 | PDFSTATSENSEMBLUCSC | 298 | 4 | 25040 | island | 0.65695 | 0.38695 | Not queried | 1.61 | 5.71e-02 | 1.00e+00 |
|  | 6 | 138745349 | 138745739 | PDFSTATSENSEMBLUCSC | 391 | 5 | 34423 | gene;island | 0.62649 | 0.89031 | Not queried | 1.61 | 2.22e-01 | 1.00e+00 |
|  | 1 | 152733025 | 152733395 | PDFSTATSENSEMBLUCSC | 371 | 6 | 757 | gene;island | 0.24895 | 0.54743 | Not queried | 1.61 | 2.16e-03 | 1.00e+00 |
|  | 2 | 9514739 | 9514994 | PDFSTATSENSEMBLUCSC | 256 | 4 | 22525 | gene;island | 0.58726 | 0.81324 | Not queried | 1.61 | 2.00e-01 | 1.00e+00 |
|  | 19 | 19322527 | 19322902 | PDFSTATSENSEMBLUCSC | 376 | 8 | 21773 | island | 0.02018 | 0.05208 | Not queried | 1.61 | 1.95e-01 | 1.00e+00 |
|  | 17 | 693137 | 693388 | PDFSTATSENSEMBLUCSC | 252 | 4 | 16259 | gene | 0.67559 | 0.84226 | Not queried | 1.61 | 5.71e-02 | 1.00e+00 |
|  | 7 | 1054075 | 1054449 | PDFSTATSENSEMBLUCSC | 375 | 6 | 36672 | gene;island | 0.69939 | 0.92463 | Not queried | 1.61 | 6.49e-02 | 1.00e+00 |
|  | X | 30265129 | 30265447 | PDFSTATSENSEMBLUCSC | 319 | 8 | 40954 | gene;island | 0.82007 | 0.92055 | Not queried | 1.61 | 6.99e-03 | 1.00e+00 |
|  | 7 | 56160717 | 56161020 | PDFSTATSENSEMBLUCSC | 304 | 7 | 37544 | tss | 0.25045 | 0.52937 | Not queried | 1.61 | 1.75e-02 | 1.00e+00 |
|  | 17 | 2240014 | 2240362 | PDFSTATSENSEMBLUCSC | 349 | 4 | 16294 | gene;tss;island | 0.03335 | 0.01117 | Not queried | 1.61 | 3.43e-01 | 1.00e+00 |
|  | 16 | 46603015 | 46603321 | PDFSTATSENSEMBLUCSC | 307 | 6 | 15187 | tss | 0.77050 | 0.93244 | Not queried | 1.61 | 9.31e-02 | 1.00e+00 |
|  | 14 | 36993831 | 36994456 | PDFSTATSENSEMBLUCSC | 626 | 5 | 12758 | island | 0.03056 | 0.09807 | Not queried | 1.61 | 1.51e-01 | 1.00e+00 |
|  | 12 | 8332808 | 8333192 | PDFSTATSENSEMBLUCSC | 385 | 5 | 9024 | gene | 0.04595 | 0.18114 | Not queried | 1.61 | 2.22e-01 | 1.00e+00 |
|  | 12 | 8332640 | 8333192 | PDFSTATSENSEMBLUCSC | 553 | 9 | 10207 | island | 0.03556 | 0.12003 | Not queried | 1.61 | 1.13e-01 | 1.00e+00 |
|  | 22 | 51001330 | 51001387 | PDFSTATSENSEMBLUCSC | 58 | 5 | 27217 | tss | 0.00397 | 0.01630 | Not queried | 1.61 | 3.10e-01 | 1.00e+00 |
|  | 19 | 7968429 | 7968594 | PDFSTATSENSEMBLUCSC | 166 | 6 | 20374 | tss;island | 0.00624 | 0.01850 | Not queried | 1.61 | 1.32e-01 | 1.00e+00 |
|  | 12 | 131512480 | 131512597 | PDFSTATSENSEMBLUCSC | 118 | 4 | 9396 | gene | 0.78096 | 0.90493 | Not queried | 1.61 | 3.43e-01 | 1.00e+00 |
|  | 1 | 147782116 | 147782558 | PDFSTATSENSEMBLUCSC | 443 | 5 | 3691 | island | 0.84004 | 0.70931 | Not queried | 1.61 | 5.56e-02 | 1.00e+00 |
|  | 8 | 144820683 | 144820908 | PDFSTATSENSEMBLUCSC | 226 | 4 | 40620 | island | 0.53025 | 0.77865 | Not queried | 1.61 | 1.14e-01 | 1.00e+00 |
|  | 10 | 135340445 | 135340850 | PDFSTATSENSEMBLUCSC | 406 | 7 | 5447 | tss | 0.94705 | 0.84129 | Not queried | 1.61 | 2.62e-02 | 1.00e+00 |
|  | 10 | 135340445 | 135341025 | PDFSTATSENSEMBLUCSC | 581 | 9 | 6349 | island | 0.94213 | 0.76704 | Not queried | 1.61 | 1.42e-02 | 1.00e+00 |
|  | X | 103810908 | 103810984 | PDFSTATSENSEMBLUCSC | 77 | 4 | 41452 | tss | 0.01732 | 0.03914 | Not queried | 1.61 | 2.00e-01 | 1.00e+00 |
|  | 5 | 133304474 | 133304652 | PDFSTATSENSEMBLUCSC | 179 | 5 | 32226 | tss | 0.01050 | 0.02276 | Not queried | 1.60 | 3.17e-02 | 1.00e+00 |
|  | 1 | 26372718 | 26373407 | PDFSTATSENSEMBLUCSC | 690 | 7 | 1377 | tss;island | 0.68227 | 0.34487 | Not queried | 1.60 | 5.35e-01 | 1.00e+00 |
|  | 19 | 22193293 | 22193724 | PDFSTATSENSEMBLUCSC | 432 | 5 | 19928 | gene | 0.88941 | 0.72538 | Not queried | 1.60 | 2.22e-01 | 1.00e+00 |
|  | 19 | 22193293 | 22193911 | PDFSTATSENSEMBLUCSC | 619 | 7 | 21806 | island | 0.88941 | 0.72538 | Not queried | 1.60 | 1.65e-01 | 1.00e+00 |
|  | 11 | 15962841 | 15963167 | PDFSTATSENSEMBLUCSC | 327 | 5 | 8065 | island | 0.19455 | 0.44953 | Not queried | 1.60 | 3.10e-01 | 1.00e+00 |
|  | 1 | 248903300 | 248903467 | PDFSTATSENSEMBLUCSC | 168 | 4 | 2369 | tss | 0.67637 | 0.43946 | Not queried | 1.60 | 2.86e-02 | 1.00e+00 |
|  | 3 | 50540137 | 50540261 | PDFSTATSENSEMBLUCSC | 125 | 4 | 27769 | gene;island | 0.02432 | 0.08289 | Not queried | 1.60 | 3.43e-01 | 1.00e+00 |
|  | X | 37209004 | 37209388 | PDFSTATSENSEMBLUCSC | 385 | 4 | 40962 | gene;island | 0.02917 | 0.09203 | Not queried | 1.60 | 3.43e-01 | 1.00e+00 |
|  | 6 | 168388195 | 168388388 | PDFSTATSENSEMBLUCSC | 194 | 4 | 36589 | island | 0.90716 | 0.76407 | Not queried | 1.60 | 2.00e-01 | 1.00e+00 |
|  | 7 | 612458 | 612876 | PDFSTATSENSEMBLUCSC | 419 | 4 | 36650 | gene;island | 0.56382 | 0.74125 | Not queried | 1.60 | 4.86e-01 | 1.00e+00 |
|  | 18 | 59560091 | 59560495 | PDFSTATSENSEMBLUCSC | 405 | 8 | 19512 | island | 0.00968 | 0.03486 | Not queried | 1.60 | 1.05e-01 | 1.00e+00 |
|  | 2 | 139660149 | 139660341 | PDFSTATSENSEMBLUCSC | 193 | 4 | 24727 | island | 0.71598 | 0.83329 | Not queried | 1.60 | 2.00e-01 | 1.00e+00 |
|  | 7 | 157849615 | 157849794 | PDFSTATSENSEMBLUCSC | 180 | 5 | 37294 | gene | 0.48955 | 0.93100 | Not queried | 1.60 | 5.56e-02 | 1.00e+00 |
|  | 2 | 219861012 | 219861150 | PDFSTATSENSEMBLUCSC | 139 | 5 | 25063 | island | 0.02203 | 0.05908 | Not queried | 1.60 | 3.10e-01 | 1.00e+00 |
|  | 20 | 61885236 | 61885448 | PDFSTATSENSEMBLUCSC | 213 | 7 | 25460 | gene;island | 0.00675 | 0.01241 | Not queried | 1.60 | 2.59e-01 | 1.00e+00 |
|  | 11 | 94883242 | 94883516 | PDFSTATSENSEMBLUCSC | 275 | 4 | 8722 | island | 0.18670 | 0.10568 | Not queried | 1.60 | 1.14e-01 | 1.00e+00 |
|  | 7 | 134250141 | 134250223 | PDFSTATSENSEMBLUCSC | 83 | 4 | 37847 | tss | 0.78022 | 0.59435 | Not queried | 1.60 | 2.00e-01 | 1.00e+00 |
|  | 7 | 142583555 | 142583836 | PDFSTATSENSEMBLUCSC | 282 | 4 | 37884 | tss | 0.04340 | 0.13478 | Not queried | 1.60 | 3.43e-01 | 1.00e+00 |
|  | 1 | 2984218 | 2984869 | PDFSTATSENSEMBLUCSC | 652 | 10 | 2523 | island | 0.59984 | 0.80396 | Not queried | 1.60 | 8.93e-03 | 1.00e+00 |
|  | 7 | 75831232 | 75831678 | PDFSTATSENSEMBLUCSC | 447 | 5 | 36988 | gene | 0.03890 | 0.04948 | Not queried | 1.60 | 4.21e-01 | 1.00e+00 |
|  | 3 | 127634188 | 127634587 | PDFSTATSENSEMBLUCSC | 400 | 5 | 29405 | island | 0.64751 | 0.90824 | Not queried | 1.60 | 1.59e-02 | 1.00e+00 |
|  | 11 | 64405764 | 64406051 | PDFSTATSENSEMBLUCSC | 288 | 4 | 6673 | gene;island | 0.46273 | 0.14141 | Not queried | 1.60 | 2.00e-01 | 1.00e+00 |
|  | 6 | 32796516 | 32796739 | PDFSTATSENSEMBLUCSC | 224 | 4 | 34072 | gene | 0.71908 | 0.51268 | Not queried | 1.60 | 5.71e-02 | 1.00e+00 |
|  | 18 | 12777645 | 12778029 | PDFSTATSENSEMBLUCSC | 385 | 4 | 19373 | island | 0.69899 | 0.88318 | Not queried | 1.60 | 2.00e-01 | 1.00e+00 |
|  | X | 117250736 | 117251123 | PDFSTATSENSEMBLUCSC | 388 | 8 | 41087 | gene;island | 0.03135 | 0.05380 | Not queried | 1.59 | 1.04e-02 | 1.00e+00 |
|  | 2 | 133428422 | 133428993 | PDFSTATSENSEMBLUCSC | 572 | 7 | 22842 | gene | 0.01157 | 0.11009 | Not queried | 1.59 | 5.30e-02 | 1.00e+00 |
|  | 2 | 133428502 | 133429299 | PDFSTATSENSEMBLUCSC | 798 | 9 | 23602 | tss | 0.01105 | 0.06882 | Not queried | 1.59 | 4.00e-02 | 1.00e+00 |
|  | 19 | 39574483 | 39574911 | PDFSTATSENSEMBLUCSC | 429 | 9 | 20729 | tss | 0.13295 | 0.26877 | Not queried | 1.59 | 7.82e-04 | 1.00e+00 |
|  | 2 | 176977220 | 176977458 | PDFSTATSENSEMBLUCSC | 239 | 4 | 24859 | island | 0.80604 | 0.62117 | Not queried | 1.59 | 5.71e-02 | 1.00e+00 |
|  | 21 | 45713510 | 45713719 | PDFSTATSENSEMBLUCSC | 210 | 4 | 26413 | gene;island | 0.98010 | 0.90731 | Not queried | 1.59 | 4.86e-01 | 1.00e+00 |
|  | 7 | 155249398 | 155249941 | PDFSTATSENSEMBLUCSC | 544 | 6 | 37946 | tss;island | 0.03737 | 0.03816 | Not queried | 1.59 | 4.85e-01 | 1.00e+00 |
|  | 11 | 16632311 | 16632724 | PDFSTATSENSEMBLUCSC | 414 | 4 | 8070 | island | 0.31638 | 0.58541 | Not queried | 1.59 | 1.14e-01 | 1.00e+00 |
|  | 6 | 32920962 | 32921233 | PDFSTATSENSEMBLUCSC | 272 | 5 | 34818 | tss | 0.32577 | 0.03247 | Not queried | 1.59 | 2.22e-01 | 1.00e+00 |
|  | 12 | 132896199 | 132896939 | PDFSTATSENSEMBLUCSC | 741 | 9 | 9422 | gene;island | 0.72182 | 0.88657 | Not queried | 1.59 | 4.00e-02 | 1.00e+00 |
|  | X | 19533456 | 19533577 | PDFSTATSENSEMBLUCSC | 122 | 6 | 41216 | tss | 0.05483 | 0.01807 | Not queried | 1.59 | 6.49e-02 | 1.00e+00 |
|  | 12 | 70083090 | 70083213 | PDFSTATSENSEMBLUCSC | 124 | 5 | 9187 | gene;tss | 0.08946 | 0.19574 | Not queried | 1.59 | 1.51e-01 | 1.00e+00 |
|  | 20 | 61924240 | 61924423 | PDFSTATSENSEMBLUCSC | 184 | 4 | 25856 | tss;island | 0.34032 | 0.65608 | Not queried | 1.59 | 1.14e-01 | 1.00e+00 |
|  | 2 | 55746547 | 55746709 | PDFSTATSENSEMBLUCSC | 163 | 5 | 23325 | tss | 0.01506 | 0.02248 | Not queried | 1.59 | 1.51e-01 | 1.00e+00 |
|  | 16 | 3142911 | 3143250 | PDFSTATSENSEMBLUCSC | 340 | 6 | 15020 | tss | 0.83977 | 0.90788 | Not queried | 1.59 | 1.32e-01 | 1.00e+00 |
|  | 1 | 110672769 | 110673319 | PDFSTATSENSEMBLUCSC | 551 | 6 | 3566 | island | 0.66727 | 0.38855 | Not queried | 1.59 | 1.52e-02 | 1.00e+00 |
|  | 19 | 16013630 | 16013895 | PDFSTATSENSEMBLUCSC | 266 | 4 | 21675 | island | 0.35259 | 0.63158 | Not queried | 1.59 | 1.14e-01 | 1.00e+00 |
|  | 11 | 2828214 | 2828778 | PDFSTATSENSEMBLUCSC | 565 | 7 | 6449 | gene;island | 0.47152 | 0.76004 | Not queried | 1.59 | 1.75e-02 | 1.00e+00 |
|  | 11 | 65779526 | 65779847 | PDFSTATSENSEMBLUCSC | 322 | 4 | 6703 | gene | 0.50387 | 0.77265 | Not queried | 1.59 | 5.71e-02 | 1.00e+00 |
|  | 6 | 32800412 | 32800591 | PDFSTATSENSEMBLUCSC | 180 | 11 | 34076 | gene | 0.85093 | 0.56282 | Not queried | 1.59 | 2.33e-02 | 1.00e+00 |
|  | 4 | 42154840 | 42154983 | PDFSTATSENSEMBLUCSC | 144 | 7 | 30961 | island | 0.06702 | 0.01547 | Not queried | 1.59 | 1.65e-01 | 1.00e+00 |
|  | 3 | 127174705 | 127175109 | PDFSTATSENSEMBLUCSC | 405 | 5 | 29400 | island | 0.19521 | 0.05929 | Not queried | 1.59 | 1.51e-01 | 1.00e+00 |
|  | 22 | 17517062 | 17517418 | PDFSTATSENSEMBLUCSC | 357 | 5 | 26898 | tss;island | 0.20278 | 0.35079 | Not queried | 1.59 | 9.52e-02 | 1.00e+00 |
|  | 3 | 138679347 | 138679578 | PDFSTATSENSEMBLUCSC | 232 | 4 | 29488 | island | 0.88068 | 0.71289 | Not queried | 1.59 | 2.86e-02 | 1.00e+00 |
|  | 19 | 7505029 | 7505344 | PDFSTATSENSEMBLUCSC | 316 | 4 | 19742 | gene;island | 0.74287 | 0.89798 | Not queried | 1.59 | 5.71e-02 | 1.00e+00 |
|  | 10 | 112431719 | 112431906 | PDFSTATSENSEMBLUCSC | 188 | 4 | 4740 | gene;island | 0.15714 | 0.04551 | Not queried | 1.59 | 3.43e-01 | 1.00e+00 |
|  | 1 | 63782980 | 63783167 | PDFSTATSENSEMBLUCSC | 188 | 4 | 3332 | island | 0.01430 | 0.03063 | Not queried | 1.58 | 3.43e-01 | 1.00e+00 |
|  | 7 | 155603792 | 155604091 | PDFSTATSENSEMBLUCSC | 300 | 4 | 37250 | gene;island | 0.06276 | 0.13860 | Not queried | 1.58 | 6.86e-01 | 1.00e+00 |
|  | 5 | 172672684 | 172673349 | PDFSTATSENSEMBLUCSC | 666 | 8 | 33346 | island | 0.66224 | 0.88826 | Not queried | 1.58 | 1.04e-02 | 1.00e+00 |
|  | 17 | 79374181 | 79374741 | PDFSTATSENSEMBLUCSC | 561 | 6 | 16873 | gene;island | 0.36493 | 0.16516 | Not queried | 1.58 | 4.85e-01 | 1.00e+00 |
|  | X | 134156624 | 134156809 | PDFSTATSENSEMBLUCSC | 186 | 5 | 41536 | tss | 0.03316 | 0.02326 | Not queried | 1.58 | 8.41e-01 | 1.00e+00 |
|  | 20 | 31755609 | 31755943 | PDFSTATSENSEMBLUCSC | 335 | 4 | 25640 | tss | 0.85270 | 0.67267 | Not queried | 1.58 | 3.43e-01 | 1.00e+00 |
|  | 6 | 31138925 | 31139359 | PDFSTATSENSEMBLUCSC | 435 | 7 | 34737 | tss | 0.34730 | 0.61451 | Not queried | 1.58 | 3.83e-01 | 1.00e+00 |
|  | 10 | 47083283 | 47083504 | PDFSTATSENSEMBLUCSC | 222 | 8 | 5046 | tss | 0.40917 | 0.62622 | Not queried | 1.58 | 2.95e-03 | 1.00e+00 |
|  | 1 | 3143682 | 3144209 | PDFSTATSENSEMBLUCSC | 528 | 5 | 108 | gene | 0.75468 | 0.81645 | Not queried | 1.58 | 9.52e-02 | 1.00e+00 |
|  | 10 | 133938604 | 133938970 | PDFSTATSENSEMBLUCSC | 367 | 6 | 4832 | gene;island | 0.74481 | 0.89469 | Not queried | 1.58 | 3.10e-01 | 1.00e+00 |
|  | 13 | 41496623 | 41496979 | PDFSTATSENSEMBLUCSC | 357 | 4 | 11379 | tss;island | 0.60057 | 0.33541 | Not queried | 1.58 | 2.86e-02 | 1.00e+00 |
|  | 20 | 865780 | 866087 | PDFSTATSENSEMBLUCSC | 308 | 5 | 25287 | gene;island | 0.04857 | 0.16100 | Not queried | 1.58 | 1.51e-01 | 1.00e+00 |
|  | 16 | 67979437 | 67979792 | PDFSTATSENSEMBLUCSC | 356 | 4 | 14779 | gene | 0.80670 | 0.93528 | Not queried | 1.58 | 1.14e-01 | 1.00e+00 |
|  | 9 | 131486520 | 131486918 | PDFSTATSENSEMBLUCSC | 399 | 6 | 40778 | tss | 0.01905 | 0.03397 | Not queried | 1.58 | 2.40e-01 | 1.00e+00 |
|  | 11 | 2176877 | 2177018 | PDFSTATSENSEMBLUCSC | 142 | 4 | 6433 | gene;island | 0.70843 | 0.88251 | Not queried | 1.58 | 2.00e-01 | 1.00e+00 |
|  | 7 | 117513825 | 117514017 | PDFSTATSENSEMBLUCSC | 193 | 4 | 37792 | tss;island | 0.06916 | 0.03317 | Not queried | 1.58 | 2.00e-01 | 1.00e+00 |
|  | 2 | 71680559 | 71680721 | PDFSTATSENSEMBLUCSC | 163 | 4 | 23392 | tss | 0.03526 | 0.10666 | Not queried | 1.58 | 2.86e-02 | 1.00e+00 |
|  | 17 | 5972281 | 5972448 | PDFSTATSENSEMBLUCSC | 168 | 4 | 17868 | island | 0.02594 | 0.07394 | Not queried | 1.58 | 2.00e-01 | 1.00e+00 |
|  | 18 | 59560365 | 59560495 | PDFSTATSENSEMBLUCSC | 131 | 5 | 19267 | tss | 0.00595 | 0.03394 | Not queried | 1.58 | 2.22e-01 | 1.00e+00 |
|  | 1 | 59280290 | 59280619 | PDFSTATSENSEMBLUCSC | 330 | 4 | 3311 | island | 0.66555 | 0.85684 | Not queried | 1.58 | 2.00e-01 | 1.00e+00 |
|  | 5 | 92914036 | 92914319 | PDFSTATSENSEMBLUCSC | 284 | 5 | 31632 | gene;island | 0.86831 | 0.58279 | Not queried | 1.58 | 1.51e-01 | 1.00e+00 |
|  | 11 | 8710368 | 8710910 | PDFSTATSENSEMBLUCSC | 543 | 5 | 6480 | gene;island | 0.03122 | 0.18232 | Not queried | 1.58 | 1.51e-01 | 1.00e+00 |
|  | 12 | 49371987 | 49372180 | PDFSTATSENSEMBLUCSC | 194 | 7 | 9654 | tss | 0.06550 | 0.22484 | Not queried | 1.58 | 1.28e-01 | 1.00e+00 |
|  | 12 | 49371987 | 49372474 | PDFSTATSENSEMBLUCSC | 488 | 9 | 10354 | island | 0.13167 | 0.32669 | Not queried | 1.58 | 1.13e-01 | 1.00e+00 |
|  | 17 | 61613061 | 61613356 | PDFSTATSENSEMBLUCSC | 296 | 5 | 16692 | gene;island | 0.91337 | 0.77673 | Not queried | 1.58 | 9.52e-02 | 1.00e+00 |
|  | 1 | 1097027 | 1097270 | PDFSTATSENSEMBLUCSC | 244 | 4 | 2400 | island | 0.56526 | 0.75550 | Not queried | 1.58 | 2.86e-02 | 1.00e+00 |
|  | 17 | 26645200 | 26645523 | PDFSTATSENSEMBLUCSC | 324 | 4 | 17174 | tss;island | 0.05885 | 0.01906 | Not queried | 1.58 | 2.86e-02 | 1.00e+00 |
|  | 11 | 36422377 | 36422615 | PDFSTATSENSEMBLUCSC | 239 | 5 | 6567 | gene | 0.72037 | 0.90568 | Not queried | 1.58 | 9.52e-02 | 1.00e+00 |
|  | 7 | 35494345 | 35494808 | PDFSTATSENSEMBLUCSC | 464 | 4 | 38345 | island | 0.54453 | 0.78562 | Not queried | 1.58 | 5.71e-02 | 1.00e+00 |
|  | 14 | 88792913 | 88793311 | PDFSTATSENSEMBLUCSC | 399 | 8 | 13028 | island | 0.07605 | 0.22816 | Not queried | 1.58 | 2.81e-02 | 1.00e+00 |
|  | 12 | 16757954 | 16758267 | PDFSTATSENSEMBLUCSC | 314 | 6 | 9046 | gene | 0.23483 | 0.08319 | Not queried | 1.58 | 6.49e-02 | 1.00e+00 |
|  | 4 | 828098 | 828281 | PDFSTATSENSEMBLUCSC | 184 | 4 | 30690 | island | 0.70821 | 0.87845 | Not queried | 1.58 | 5.71e-02 | 1.00e+00 |
|  | 21 | 45705175 | 45705742 | PDFSTATSENSEMBLUCSC | 568 | 10 | 26541 | tss;island | 0.77832 | 0.92520 | Not queried | 1.58 | 1.47e-02 | 1.00e+00 |
|  | 7 | 158235891 | 158236160 | PDFSTATSENSEMBLUCSC | 270 | 6 | 37307 | gene | 0.78021 | 0.88926 | Not queried | 1.58 | 3.10e-01 | 1.00e+00 |
|  | 11 | 71724287 | 71724677 | PDFSTATSENSEMBLUCSC | 391 | 6 | 6789 | gene | 0.64993 | 0.87327 | Not queried | 1.57 | 6.49e-02 | 1.00e+00 |
|  | 5 | 14405632 | 14406021 | PDFSTATSENSEMBLUCSC | 390 | 5 | 31507 | gene | 0.70299 | 0.83618 | Not queried | 1.57 | 3.10e-01 | 1.00e+00 |
|  | 22 | 29875578 | 29875991 | PDFSTATSENSEMBLUCSC | 414 | 5 | 27007 | tss;island | 0.03745 | 0.24265 | Not queried | 1.57 | 2.22e-01 | 1.00e+00 |
|  | 17 | 6679254 | 6679781 | PDFSTATSENSEMBLUCSC | 528 | 12 | 17877 | island | 0.53709 | 0.23442 | Not queried | 1.57 | 2.22e-05 | 1.14e-01 |
|  | 5 | 140473857 | 140474184 | PDFSTATSENSEMBLUCSC | 328 | 6 | 32302 | tss | 0.03252 | 0.10263 | Not queried | 1.57 | 3.10e-01 | 1.00e+00 |
|  | 6 | 150378738 | 150379047 | PDFSTATSENSEMBLUCSC | 310 | 5 | 36504 | island | 0.23992 | 0.49910 | Not queried | 1.57 | 5.56e-02 | 1.00e+00 |
|  | 13 | 111281037 | 111281479 | PDFSTATSENSEMBLUCSC | 443 | 6 | 11205 | gene;island | 0.69596 | 0.93073 | Not queried | 1.57 | 2.60e-02 | 1.00e+00 |
|  | 17 | 7452289 | 7452644 | PDFSTATSENSEMBLUCSC | 356 | 5 | 17922 | island | 0.34027 | 0.19631 | Not queried | 1.57 | 2.22e-01 | 1.00e+00 |
|  | 3 | 27765232 | 27765546 | PDFSTATSENSEMBLUCSC | 315 | 5 | 28909 | island | 0.26825 | 0.21156 | Not queried | 1.57 | 6.90e-01 | 1.00e+00 |
|  | 17 | 46698881 | 46699155 | PDFSTATSENSEMBLUCSC | 275 | 4 | 16626 | gene;island | 0.10499 | 0.37369 | Not queried | 1.57 | 5.71e-02 | 1.00e+00 |
|  | 7 | 150065170 | 150065255 | PDFSTATSENSEMBLUCSC | 86 | 6 | 37913 | tss;island | 0.29587 | 0.10565 | Not queried | 1.57 | 6.49e-02 | 1.00e+00 |
|  | 10 | 131697669 | 131697980 | PDFSTATSENSEMBLUCSC | 312 | 4 | 4821 | gene;island | 0.88693 | 0.73047 | Not queried | 1.57 | 1.14e-01 | 1.00e+00 |
|  | 6 | 30853948 | 30854233 | PDFSTATSENSEMBLUCSC | 286 | 8 | 33764 | gene;island | 0.19983 | 0.08726 | Not queried | 1.57 | 1.30e-01 | 1.00e+00 |
|  | 2 | 220196530 | 220196755 | PDFSTATSENSEMBLUCSC | 226 | 4 | 22996 | gene;island | 0.16790 | 0.44006 | Not queried | 1.57 | 4.86e-01 | 1.00e+00 |
|  | 22 | 50451054 | 50451414 | PDFSTATSENSEMBLUCSC | 361 | 5 | 27568 | island | 0.40866 | 0.66550 | Not queried | 1.57 | 5.56e-02 | 1.00e+00 |
|  | 3 | 13520685 | 13521744 | PDFSTATSENSEMBLUCSC | 1060 | 25 | 28122 | tss | 0.08715 | 0.03231 | Not queried | 1.57 | 1.61e-02 | 1.00e+00 |
|  | 4 | 46911291 | 46911550 | PDFSTATSENSEMBLUCSC | 260 | 5 | 30327 | tss | 0.64410 | 0.84252 | Not queried | 1.57 | 2.22e-01 | 1.00e+00 |
|  | 6 | 86302808 | 86302950 | PDFSTATSENSEMBLUCSC | 143 | 4 | 34340 | gene;island | 0.01083 | 0.02983 | Not queried | 1.57 | 3.43e-01 | 1.00e+00 |
|  | 10 | 71336773 | 71337104 | PDFSTATSENSEMBLUCSC | 332 | 4 | 5788 | island | 0.22090 | 0.06720 | Not queried | 1.57 | 4.86e-01 | 1.00e+00 |
|  | 7 | 150019955 | 150020401 | PDFSTATSENSEMBLUCSC | 447 | 10 | 37207 | gene;island | 0.34360 | 0.16759 | Not queried | 1.57 | 1.30e-04 | 6.68e-01 |
|  | 11 | 1474386 | 1474841 | PDFSTATSENSEMBLUCSC | 456 | 5 | 6403 | gene;island | 0.45015 | 0.76784 | Not queried | 1.57 | 5.56e-02 | 1.00e+00 |
|  | 3 | 62861071 | 62861311 | PDFSTATSENSEMBLUCSC | 241 | 6 | 28385 | tss | 0.02317 | 0.09306 | Not queried | 1.57 | 2.40e-01 | 1.00e+00 |
|  | 17 | 37074902 | 37075068 | PDFSTATSENSEMBLUCSC | 167 | 4 | 16510 | gene;island | 0.67588 | 0.84191 | Not queried | 1.57 | 3.43e-01 | 1.00e+00 |
|  | 16 | 1480790 | 1480992 | PDFSTATSENSEMBLUCSC | 203 | 4 | 15511 | island | 0.59308 | 0.82769 | Not queried | 1.56 | 3.43e-01 | 1.00e+00 |
|  | 17 | 80185943 | 80186336 | PDFSTATSENSEMBLUCSC | 394 | 6 | 17736 | tss;island | 0.78103 | 0.50696 | Not queried | 1.56 | 4.11e-02 | 1.00e+00 |
|  | 10 | 126331180 | 126331425 | PDFSTATSENSEMBLUCSC | 246 | 4 | 4793 | gene | 0.84934 | 0.93302 | Not queried | 1.56 | 2.00e-01 | 1.00e+00 |
|  | 19 | 2474993 | 2475189 | PDFSTATSENSEMBLUCSC | 197 | 4 | 20291 | tss;island | 0.04672 | 0.09118 | Not queried | 1.56 | 5.71e-02 | 1.00e+00 |
|  | 18 | 55019621 | 55019711 | PDFSTATSENSEMBLUCSC | 91 | 4 | 19256 | tss | 0.15434 | 0.07835 | Not queried | 1.56 | 1.14e-01 | 1.00e+00 |
|  | 1 | 27216732 | 27216796 | PDFSTATSENSEMBLUCSC | 65 | 6 | 345 | gene | 0.02023 | 0.06030 | Not queried | 1.56 | 1.32e-01 | 1.00e+00 |
|  | 11 | 1154976 | 1155278 | PDFSTATSENSEMBLUCSC | 303 | 4 | 7883 | island | 0.78034 | 0.90025 | Not queried | 1.56 | 2.00e-01 | 1.00e+00 |
|  | 16 | 2762569 | 2762819 | PDFSTATSENSEMBLUCSC | 251 | 4 | 14580 | gene;island | 0.63877 | 0.74082 | Not queried | 1.56 | 3.43e-01 | 1.00e+00 |
|  | 3 | 156544070 | 156544233 | PDFSTATSENSEMBLUCSC | 164 | 5 | 29567 | island | 0.23353 | 0.54596 | Not queried | 1.56 | 7.94e-03 | 1.00e+00 |
|  | 12 | 109162390 | 109162748 | PDFSTATSENSEMBLUCSC | 359 | 6 | 10745 | island | 0.42542 | 0.18343 | Not queried | 1.56 | 4.33e-03 | 1.00e+00 |
|  | 2 | 74725040 | 74725369 | PDFSTATSENSEMBLUCSC | 330 | 4 | 22706 | gene;island | 0.12223 | 0.10328 | Not queried | 1.56 | 6.86e-01 | 1.00e+00 |
|  | X | 43741814 | 43741945 | PDFSTATSENSEMBLUCSC | 132 | 5 | 41267 | tss;island | 0.05694 | 0.02410 | Not queried | 1.56 | 5.48e-01 | 1.00e+00 |
|  | 18 | 77230484 | 77230795 | PDFSTATSENSEMBLUCSC | 312 | 4 | 19107 | gene;island | 0.74049 | 0.89526 | Not queried | 1.56 | 2.86e-02 | 1.00e+00 |
|  | 19 | 18111623 | 18111880 | PDFSTATSENSEMBLUCSC | 258 | 5 | 20571 | tss | 0.10798 | 0.04671 | Not queried | 1.56 | 2.22e-01 | 1.00e+00 |
|  | 7 | 157352978 | 157353191 | PDFSTATSENSEMBLUCSC | 214 | 5 | 37263 | gene;island | 0.88637 | 0.94305 | Not queried | 1.56 | 3.10e-01 | 1.00e+00 |
|  | 19 | 13885098 | 13885429 | PDFSTATSENSEMBLUCSC | 332 | 5 | 21619 | island | 0.01990 | 0.03837 | Not queried | 1.56 | 4.21e-01 | 1.00e+00 |
|  | 15 | 65068326 | 65068594 | PDFSTATSENSEMBLUCSC | 269 | 4 | 13704 | tss;island | 0.04174 | 0.02773 | Not queried | 1.56 | 4.86e-01 | 1.00e+00 |
|  | 6 | 31275551 | 31275881 | PDFSTATSENSEMBLUCSC | 331 | 11 | 35700 | island | 0.41452 | 0.64915 | Not queried | 1.56 | 1.13e-05 | 5.83e-02 |
|  | 1 | 1148973 | 1149211 | PDFSTATSENSEMBLUCSC | 239 | 4 | 21 | gene;island | 0.50013 | 0.72952 | Not queried | 1.56 | 2.86e-02 | 1.00e+00 |
|  | 17 | 20946875 | 20947103 | PDFSTATSENSEMBLUCSC | 229 | 4 | 17166 | tss;island | 0.00455 | 0.01224 | Not queried | 1.56 | 2.00e-01 | 1.00e+00 |
|  | 6 | 43141823 | 43142093 | PDFSTATSENSEMBLUCSC | 271 | 4 | 34280 | gene;island | 0.39233 | 0.48923 | Not queried | 1.56 | 8.86e-01 | 1.00e+00 |
|  | 12 | 51818636 | 51818768 | PDFSTATSENSEMBLUCSC | 133 | 4 | 9107 | gene | 0.03651 | 0.02251 | Not queried | 1.56 | 4.86e-01 | 1.00e+00 |
|  | X | 37706736 | 37707161 | PDFSTATSENSEMBLUCSC | 426 | 7 | 41716 | island | 0.13258 | 0.02737 | Not queried | 1.56 | 1.28e-01 | 1.00e+00 |
|  | 5 | 131346985 | 131347278 | PDFSTATSENSEMBLUCSC | 294 | 4 | 31687 | gene | 0.10407 | 0.06955 | Not queried | 1.55 | 8.86e-01 | 1.00e+00 |
|  | 7 | 45613676 | 45613825 | PDFSTATSENSEMBLUCSC | 150 | 4 | 37519 | tss;island | 0.16556 | 0.04804 | Not queried | 1.55 | 8.86e-01 | 1.00e+00 |
|  | 3 | 75707805 | 75708197 | PDFSTATSENSEMBLUCSC | 393 | 4 | 29246 | island | 0.45101 | 0.25516 | Not queried | 1.55 | 3.43e-01 | 1.00e+00 |
|  | 13 | 88328251 | 88328471 | PDFSTATSENSEMBLUCSC | 221 | 4 | 11159 | gene;island | 0.33452 | 0.60247 | Not queried | 1.55 | 2.00e-01 | 1.00e+00 |
|  | 10 | 180188 | 180411 | PDFSTATSENSEMBLUCSC | 224 | 7 | 4893 | tss | 0.01593 | 0.05383 | Not queried | 1.55 | 1.65e-01 | 1.00e+00 |
|  | 13 | 103532159 | 103532435 | PDFSTATSENSEMBLUCSC | 277 | 7 | 11499 | tss | 0.54290 | 0.76720 | Not queried | 1.55 | 9.73e-02 | 1.00e+00 |
|  | 12 | 50475035 | 50475372 | PDFSTATSENSEMBLUCSC | 338 | 4 | 9102 | gene;island | 0.90620 | 0.66279 | Not queried | 1.55 | 1.14e-01 | 1.00e+00 |
|  | 7 | 4848683 | 4848939 | PDFSTATSENSEMBLUCSC | 257 | 4 | 36794 | gene;island | 0.87641 | 0.93361 | Not queried | 1.55 | 3.43e-01 | 1.00e+00 |
|  | 18 | 5891948 | 5892245 | PDFSTATSENSEMBLUCSC | 298 | 4 | 19342 | island | 0.67693 | 0.41619 | Not queried | 1.55 | 2.86e-02 | 1.00e+00 |
|  | 7 | 80548695 | 80548752 | PDFSTATSENSEMBLUCSC | 58 | 5 | 37610 | tss;island | 0.01554 | 0.04424 | Not queried | 1.55 | 4.21e-01 | 1.00e+00 |
|  | 5 | 176937350 | 176937527 | PDFSTATSENSEMBLUCSC | 178 | 4 | 32471 | tss | 0.84449 | 0.92242 | Not queried | 1.55 | 1.14e-01 | 1.00e+00 |
|  | 6 | 32126315 | 32126616 | PDFSTATSENSEMBLUCSC | 302 | 4 | 34000 | gene | 0.83323 | 0.92359 | Not queried | 1.55 | 2.00e-01 | 1.00e+00 |
|  | 6 | 168197625 | 168198163 | PDFSTATSENSEMBLUCSC | 539 | 7 | 35279 | tss | 0.53069 | 0.76149 | Not queried | 1.55 | 1.11e-02 | 1.00e+00 |
|  | 12 | 132939657 | 132939992 | PDFSTATSENSEMBLUCSC | 336 | 4 | 11019 | island | 0.86024 | 0.94651 | Not queried | 1.55 | 1.14e-01 | 1.00e+00 |
|  | 1 | 181451906 | 181452137 | PDFSTATSENSEMBLUCSC | 232 | 6 | 2125 | tss;island | 0.04078 | 0.09543 | Not queried | 1.55 | 4.33e-03 | 1.00e+00 |
|  | 20 | 1294311 | 1294376 | PDFSTATSENSEMBLUCSC | 66 | 4 | 25498 | tss | 0.17519 | 0.04332 | Not queried | 1.55 | 4.86e-01 | 1.00e+00 |
|  | 1 | 24194974 | 24195659 | PDFSTATSENSEMBLUCSC | 686 | 12 | 1347 | tss;island | 0.06355 | 0.18173 | Not queried | 1.55 | 4.49e-02 | 1.00e+00 |
|  | 6 | 30540557 | 30540762 | PDFSTATSENSEMBLUCSC | 206 | 4 | 33715 | gene;island | 0.74621 | 0.89084 | Not queried | 1.55 | 3.43e-01 | 1.00e+00 |
|  | 8 | 33369905 | 33370172 | PDFSTATSENSEMBLUCSC | 268 | 5 | 39213 | gene;island | 0.02012 | 0.01177 | Not queried | 1.55 | 1.51e-01 | 1.00e+00 |
|  | 22 | 46402185 | 46402486 | PDFSTATSENSEMBLUCSC | 302 | 4 | 27183 | tss | 0.07568 | 0.32368 | Not queried | 1.55 | 4.86e-01 | 1.00e+00 |
|  | 2 | 130955979 | 130956271 | PDFSTATSENSEMBLUCSC | 293 | 8 | 24678 | island | 0.96948 | 0.87074 | Not queried | 1.55 | 1.30e-01 | 1.00e+00 |
|  | 1 | 6240267 | 6240508 | PDFSTATSENSEMBLUCSC | 242 | 5 | 1194 | tss;island | 0.02794 | 0.09876 | Not queried | 1.55 | 3.17e-02 | 1.00e+00 |
|  | 21 | 45773189 | 45773400 | PDFSTATSENSEMBLUCSC | 212 | 4 | 26543 | tss | 0.58115 | 0.81662 | Not queried | 1.55 | 6.86e-01 | 1.00e+00 |
|  | 1 | 1309161 | 1309641 | PDFSTATSENSEMBLUCSC | 481 | 4 | 33 | gene;island | 0.25564 | 0.51340 | Not queried | 1.55 | 2.00e-01 | 1.00e+00 |
|  | 15 | 45926780 | 45927218 | PDFSTATSENSEMBLUCSC | 439 | 8 | 13625 | tss | 0.07224 | 0.04596 | Not queried | 1.55 | 6.50e-02 | 1.00e+00 |
|  | 22 | 50451108 | 50451414 | PDFSTATSENSEMBLUCSC | 307 | 4 | 27200 | tss | 0.35610 | 0.61464 | Not queried | 1.55 | 2.00e-01 | 1.00e+00 |
|  | 3 | 47620963 | 47621127 | PDFSTATSENSEMBLUCSC | 165 | 4 | 28247 | tss;island | 0.55979 | 0.37835 | Not queried | 1.55 | 2.86e-02 | 1.00e+00 |
|  | 17 | 1617102 | 1617465 | PDFSTATSENSEMBLUCSC | 364 | 6 | 16282 | gene;island | 0.06332 | 0.18089 | Not queried | 1.55 | 1.80e-01 | 1.00e+00 |
|  | 1 | 25868969 | 25869196 | PDFSTATSENSEMBLUCSC | 228 | 4 | 1366 | tss;island | 0.82786 | 0.62248 | Not queried | 1.55 | 4.86e-01 | 1.00e+00 |
|  | 20 | 1165206 | 1165435 | PDFSTATSENSEMBLUCSC | 230 | 4 | 25495 | tss | 0.78586 | 0.46306 | Not queried | 1.55 | 2.00e-01 | 1.00e+00 |
|  | 17 | 800084 | 800512 | PDFSTATSENSEMBLUCSC | 429 | 5 | 16264 | gene | 0.89400 | 0.81934 | Not queried | 1.55 | 9.52e-02 | 1.00e+00 |
|  | 11 | 1331124 | 1331497 | PDFSTATSENSEMBLUCSC | 374 | 4 | 6399 | gene;tss;island | 0.20994 | 0.07403 | Not queried | 1.55 | 2.00e-01 | 1.00e+00 |
|  | 5 | 37838290 | 37838871 | PDFSTATSENSEMBLUCSC | 582 | 6 | 31536 | gene;island | 0.05326 | 0.12229 | Not queried | 1.55 | 3.94e-01 | 1.00e+00 |
|  | 7 | 149416674 | 149416973 | PDFSTATSENSEMBLUCSC | 300 | 5 | 37203 | gene;island | 0.78043 | 0.94994 | Not queried | 1.55 | 1.51e-01 | 1.00e+00 |
|  | 19 | 50303041 | 50303389 | PDFSTATSENSEMBLUCSC | 349 | 5 | 20127 | gene;island | 0.82492 | 0.92556 | Not queried | 1.54 | 3.10e-01 | 1.00e+00 |
|  | 6 | 29723301 | 29723407 | PDFSTATSENSEMBLUCSC | 107 | 4 | 35566 | island | 0.68126 | 0.47632 | Not queried | 1.54 | 2.00e-01 | 1.00e+00 |
|  | 18 | 44557635 | 44557819 | PDFSTATSENSEMBLUCSC | 185 | 5 | 19072 | gene;tss;island | 0.64057 | 0.83868 | Not queried | 1.54 | 2.22e-01 | 1.00e+00 |
|  | 11 | 46383031 | 46383209 | PDFSTATSENSEMBLUCSC | 179 | 4 | 6581 | gene | 0.48513 | 0.73982 | Not queried | 1.54 | 2.86e-02 | 1.00e+00 |
|  | 10 | 131265059 | 131265435 | PDFSTATSENSEMBLUCSC | 377 | 11 | 5422 | tss | 0.04378 | 0.09774 | Not queried | 1.54 | 2.81e-02 | 1.00e+00 |
|  | X | 103810908 | 103811025 | PDFSTATSENSEMBLUCSC | 118 | 5 | 41937 | island | 0.01011 | 0.02840 | Not queried | 1.54 | 1.51e-01 | 1.00e+00 |
|  | 7 | 2078650 | 2079008 | PDFSTATSENSEMBLUCSC | 359 | 5 | 36725 | gene | 0.80407 | 0.91467 | Not queried | 1.54 | 5.56e-02 | 1.00e+00 |
|  | 8 | 18666531 | 18666598 | PDFSTATSENSEMBLUCSC | 68 | 4 | 39157 | gene;tss | 0.76047 | 0.90040 | Not queried | 1.54 | 2.86e-02 | 1.00e+00 |
|  | 3 | 48342880 | 48343235 | PDFSTATSENSEMBLUCSC | 356 | 7 | 28253 | tss | 0.04477 | 0.02130 | Not queried | 1.54 | 5.30e-02 | 1.00e+00 |
|  | 13 | 113759771 | 113760286 | PDFSTATSENSEMBLUCSC | 516 | 6 | 11927 | island | 0.68886 | 0.87142 | Not queried | 1.54 | 1.32e-01 | 1.00e+00 |
|  | 5 | 7869257 | 7869428 | PDFSTATSENSEMBLUCSC | 172 | 5 | 31502 | gene | 0.00408 | 0.01178 | Not queried | 1.54 | 3.10e-01 | 1.00e+00 |
|  | 19 | 40732518 | 40732902 | PDFSTATSENSEMBLUCSC | 385 | 9 | 21983 | island | 0.47302 | 0.75926 | Not queried | 1.54 | 1.85e-03 | 1.00e+00 |
|  | 5 | 158689930 | 158690029 | PDFSTATSENSEMBLUCSC | 100 | 4 | 32403 | tss | 0.01097 | 0.02698 | Not queried | 1.54 | 2.00e-01 | 1.00e+00 |
|  | 16 | 84401756 | 84402160 | PDFSTATSENSEMBLUCSC | 405 | 6 | 16108 | island | 0.03015 | 0.09268 | Not queried | 1.54 | 3.10e-01 | 1.00e+00 |
|  | 12 | 75905429 | 75905681 | PDFSTATSENSEMBLUCSC | 253 | 7 | 9857 | tss | 0.01607 | 0.03051 | Not queried | 1.54 | 9.73e-02 | 1.00e+00 |
|  | 3 | 147124363 | 147124827 | PDFSTATSENSEMBLUCSC | 465 | 6 | 28608 | tss;island | 0.53100 | 0.39020 | Not queried | 1.54 | 5.89e-01 | 1.00e+00 |
|  | 19 | 11531294 | 11531469 | PDFSTATSENSEMBLUCSC | 176 | 4 | 20436 | tss | 0.02071 | 0.04402 | Not queried | 1.54 | 3.43e-01 | 1.00e+00 |
|  | 6 | 32732585 | 32732806 | PDFSTATSENSEMBLUCSC | 222 | 4 | 34809 | tss;island | 0.73960 | 0.49877 | Not queried | 1.54 | 6.86e-01 | 1.00e+00 |
|  | 12 | 113574066 | 113574368 | PDFSTATSENSEMBLUCSC | 303 | 6 | 10000 | tss | 0.43058 | 0.20455 | Not queried | 1.54 | 1.32e-01 | 1.00e+00 |
|  | 1 | 228558987 | 228559321 | PDFSTATSENSEMBLUCSC | 335 | 5 | 1054 | gene;island | 0.60893 | 0.82862 | Not queried | 1.54 | 7.94e-03 | 1.00e+00 |
|  | 11 | 61355119 | 61355505 | PDFSTATSENSEMBLUCSC | 387 | 4 | 8307 | island | 0.02543 | 0.07128 | Not queried | 1.54 | 1.14e-01 | 1.00e+00 |
|  | 6 | 42110740 | 42111005 | PDFSTATSENSEMBLUCSC | 266 | 5 | 34914 | tss;island | 0.53627 | 0.63831 | Not queried | 1.54 | 4.21e-01 | 1.00e+00 |
|  | 19 | 16528764 | 16529096 | PDFSTATSENSEMBLUCSC | 333 | 4 | 19875 | gene;island | 0.34024 | 0.77644 | Not queried | 1.54 | 1.14e-01 | 1.00e+00 |
|  | 17 | 77216389 | 77216699 | PDFSTATSENSEMBLUCSC | 311 | 4 | 16803 | gene | 0.53032 | 0.76605 | Not queried | 1.54 | 5.71e-02 | 1.00e+00 |
|  | 22 | 42949526 | 42949829 | PDFSTATSENSEMBLUCSC | 304 | 8 | 27151 | tss | 0.04861 | 0.02314 | Not queried | 1.54 | 1.95e-01 | 1.00e+00 |
|  | 3 | 52008487 | 52009002 | PDFSTATSENSEMBLUCSC | 516 | 11 | 28327 | tss;island | 0.11509 | 0.25149 | Not queried | 1.53 | 1.16e-01 | 1.00e+00 |
|  | 5 | 138713750 | 138714339 | PDFSTATSENSEMBLUCSC | 590 | 7 | 31718 | gene;island | 0.56256 | 0.78992 | Not queried | 1.53 | 5.83e-04 | 1.00e+00 |
|  | 17 | 41277694 | 41279022 | PDFSTATSENSEMBLUCSC | 1329 | 28 | 16560 | gene | 0.62368 | 0.38919 | Not queried | 1.53 | 8.22e-03 | 1.00e+00 |
|  | 7 | 100612725 | 100612937 | PDFSTATSENSEMBLUCSC | 213 | 5 | 38722 | island | 0.34236 | 0.62597 | Not queried | 1.53 | 5.56e-02 | 1.00e+00 |
|  | 13 | 111522755 | 111522985 | PDFSTATSENSEMBLUCSC | 231 | 5 | 11512 | tss | 0.67726 | 0.42200 | Not queried | 1.53 | 7.94e-03 | 1.00e+00 |
|  | 12 | 11151430 | 11151622 | PDFSTATSENSEMBLUCSC | 193 | 4 | 9029 | gene;tss | 0.75115 | 0.89248 | Not queried | 1.53 | 2.86e-02 | 1.00e+00 |
|  | 6 | 33244976 | 33246488 | PDFSTATSENSEMBLUCSC | 1513 | 45 | 34181 | gene | 0.68953 | 0.45706 | Not queried | 1.53 | 5.13e-03 | 1.00e+00 |
|  | X | 12601534 | 12601783 | PDFSTATSENSEMBLUCSC | 250 | 4 | 40938 | gene;island | 0.84250 | 0.95389 | Not queried | 1.53 | 1.14e-01 | 1.00e+00 |
|  | 8 | 99076526 | 99076837 | PDFSTATSENSEMBLUCSC | 312 | 6 | 40418 | island | 0.15597 | 0.08004 | Not queried | 1.53 | 2.40e-01 | 1.00e+00 |
|  | 11 | 405846 | 406019 | PDFSTATSENSEMBLUCSC | 174 | 4 | 6352 | gene;island | 0.80728 | 0.67248 | Not queried | 1.53 | 1.14e-01 | 1.00e+00 |
|  | 7 | 1407330 | 1407571 | PDFSTATSENSEMBLUCSC | 242 | 6 | 38036 | island | 0.61742 | 0.84817 | Not queried | 1.53 | 1.52e-02 | 1.00e+00 |
|  | 6 | 1523751 | 1524190 | PDFSTATSENSEMBLUCSC | 440 | 5 | 35305 | island | 0.85632 | 0.55137 | Not queried | 1.53 | 1.51e-01 | 1.00e+00 |
|  | 6 | 33282313 | 33283328 | PDFSTATSENSEMBLUCSC | 1016 | 34 | 34199 | gene;tss;island | 0.14363 | 0.46921 | Not queried | 1.53 | 6.80e-03 | 1.00e+00 |
|  | 7 | 73275265 | 73275436 | PDFSTATSENSEMBLUCSC | 172 | 6 | 37579 | tss | 0.83687 | 0.64973 | Not queried | 1.53 | 9.31e-02 | 1.00e+00 |
|  | 18 | 77748485 | 77748761 | PDFSTATSENSEMBLUCSC | 277 | 5 | 19580 | island | 0.01578 | 0.03615 | Not queried | 1.53 | 9.52e-02 | 1.00e+00 |
|  | 13 | 114927579 | 114928233 | PDFSTATSENSEMBLUCSC | 655 | 8 | 11970 | island | 0.40977 | 0.64333 | Not queried | 1.53 | 2.07e-02 | 1.00e+00 |
|  | X | 46696351 | 46696474 | PDFSTATSENSEMBLUCSC | 124 | 4 | 40972 | gene | 0.03267 | 0.02389 | Not queried | 1.53 | 4.86e-01 | 1.00e+00 |
|  | 3 | 49756301 | 49757476 | PDFSTATSENSEMBLUCSC | 1176 | 12 | 27746 | gene;island | 0.17725 | 0.36417 | Not queried | 1.53 | 1.78e-01 | 1.00e+00 |
|  | 19 | 45316493 | 45316789 | PDFSTATSENSEMBLUCSC | 297 | 4 | 20049 | gene;island | 0.97328 | 0.90048 | Not queried | 1.53 | 3.43e-01 | 1.00e+00 |
|  | 16 | 89293993 | 89294245 | PDFSTATSENSEMBLUCSC | 253 | 5 | 14903 | gene | 0.97316 | 0.93890 | Not queried | 1.53 | 5.56e-02 | 1.00e+00 |
|  | 14 | 101335129 | 101335386 | PDFSTATSENSEMBLUCSC | 258 | 5 | 12581 | tss | 0.48114 | 0.69143 | Not queried | 1.53 | 7.94e-03 | 1.00e+00 |
|  | 11 | 117651217 | 117651424 | PDFSTATSENSEMBLUCSC | 208 | 4 | 6902 | gene;island | 0.65093 | 0.78748 | Not queried | 1.53 | 5.71e-02 | 1.00e+00 |
|  | 2 | 242844601 | 242844943 | PDFSTATSENSEMBLUCSC | 343 | 4 | 25277 | island | 0.67226 | 0.85953 | Not queried | 1.53 | 1.14e-01 | 1.00e+00 |
|  | 3 | 33260732 | 33261204 | PDFSTATSENSEMBLUCSC | 473 | 7 | 28170 | tss;island | 0.30926 | 0.06613 | Not queried | 1.53 | 6.20e-01 | 1.00e+00 |
|  | 1 | 166134182 | 166134699 | PDFSTATSENSEMBLUCSC | 518 | 6 | 866 | gene;island | 0.02586 | 0.07101 | Not queried | 1.53 | 1.80e-01 | 1.00e+00 |
|  | 4 | 42154912 | 42154983 | PDFSTATSENSEMBLUCSC | 72 | 6 | 30319 | tss | 0.08810 | 0.03241 | Not queried | 1.53 | 3.94e-01 | 1.00e+00 |
|  | 2 | 133428422 | 133429299 | PDFSTATSENSEMBLUCSC | 878 | 10 | 24708 | island | 0.01131 | 0.08896 | Not queried | 1.53 | 3.55e-02 | 1.00e+00 |
|  | 12 | 132832250 | 132832440 | PDFSTATSENSEMBLUCSC | 191 | 4 | 9413 | gene;island | 0.67394 | 0.82599 | Not queried | 1.53 | 2.86e-02 | 1.00e+00 |
|  | 4 | 156298166 | 156298427 | PDFSTATSENSEMBLUCSC | 262 | 7 | 30602 | tss | 0.08218 | 0.24152 | Not queried | 1.53 | 1.28e-01 | 1.00e+00 |
|  | 1 | 207494213 | 207494733 | PDFSTATSENSEMBLUCSC | 521 | 6 | 2216 | tss;island | 0.02028 | 0.01273 | Not queried | 1.53 | 2.60e-02 | 1.00e+00 |
|  | 3 | 119041204 | 119041529 | PDFSTATSENSEMBLUCSC | 326 | 4 | 27865 | gene;island | 0.08323 | 0.02764 | Not queried | 1.52 | 4.86e-01 | 1.00e+00 |
|  | 8 | 1327367 | 1327621 | PDFSTATSENSEMBLUCSC | 255 | 6 | 39958 | island | 0.68994 | 0.90529 | Not queried | 1.52 | 4.11e-02 | 1.00e+00 |
|  | 16 | 67428155 | 67428497 | PDFSTATSENSEMBLUCSC | 343 | 4 | 15270 | tss;island | 0.42352 | 0.66076 | Not queried | 1.52 | 2.86e-02 | 1.00e+00 |
|  | 17 | 66254884 | 66255444 | PDFSTATSENSEMBLUCSC | 561 | 8 | 18656 | island | 0.02660 | 0.10243 | Not queried | 1.52 | 3.79e-02 | 1.00e+00 |
|  | 12 | 107078410 | 107078680 | PDFSTATSENSEMBLUCSC | 271 | 5 | 9256 | gene | 0.82311 | 0.93191 | Not queried | 1.52 | 3.10e-01 | 1.00e+00 |
|  | 10 | 86300295 | 86300605 | PDFSTATSENSEMBLUCSC | 311 | 4 | 5879 | island | 0.17245 | 0.05176 | Not queried | 1.52 | 2.00e-01 | 1.00e+00 |
|  | 11 | 910094 | 910475 | PDFSTATSENSEMBLUCSC | 382 | 4 | 6377 | gene;island | 0.03185 | 0.04637 | Not queried | 1.52 | 4.86e-01 | 1.00e+00 |
|  | 7 | 5527927 | 5528266 | PDFSTATSENSEMBLUCSC | 340 | 4 | 36815 | gene;island | 0.50083 | 0.84299 | Not queried | 1.52 | 5.71e-02 | 1.00e+00 |
|  | 19 | 49004523 | 49004834 | PDFSTATSENSEMBLUCSC | 312 | 4 | 20092 | gene;island | 0.11975 | 0.07747 | Not queried | 1.52 | 2.00e-01 | 1.00e+00 |
|  | 7 | 44614305 | 44614519 | PDFSTATSENSEMBLUCSC | 215 | 5 | 37504 | tss;island | 0.32965 | 0.58543 | Not queried | 1.52 | 3.10e-01 | 1.00e+00 |
|  | 2 | 74153086 | 74153317 | PDFSTATSENSEMBLUCSC | 232 | 4 | 23409 | tss;island | 0.73942 | 0.88173 | Not queried | 1.52 | 1.14e-01 | 1.00e+00 |
|  | 11 | 67210033 | 67210282 | PDFSTATSENSEMBLUCSC | 250 | 5 | 6739 | gene | 0.41761 | 0.66917 | Not queried | 1.52 | 1.59e-02 | 1.00e+00 |
|  | 10 | 37413610 | 37413799 | PDFSTATSENSEMBLUCSC | 190 | 4 | 5013 | tss;island | 0.54181 | 0.77471 | Not queried | 1.52 | 2.86e-02 | 1.00e+00 |
|  | 17 | 72350354 | 72350710 | PDFSTATSENSEMBLUCSC | 357 | 4 | 16716 | gene;island | 0.18101 | 0.42592 | Not queried | 1.52 | 2.00e-01 | 1.00e+00 |
|  | 5 | 65222276 | 65222522 | PDFSTATSENSEMBLUCSC | 247 | 5 | 32777 | island | 0.00328 | 0.03134 | Not queried | 1.52 | 4.21e-01 | 1.00e+00 |
|  | 19 | 55866087 | 55866412 | PDFSTATSENSEMBLUCSC | 326 | 9 | 22383 | island | 0.03774 | 0.08280 | Not queried | 1.52 | 1.61e-01 | 1.00e+00 |
|  | 5 | 93447204 | 93447700 | PDFSTATSENSEMBLUCSC | 497 | 10 | 32902 | island | 0.02459 | 0.10154 | Not queried | 1.52 | 1.23e-01 | 1.00e+00 |
|  | X | 54466428 | 54466849 | PDFSTATSENSEMBLUCSC | 422 | 9 | 41334 | tss | 0.05076 | 0.01917 | Not queried | 1.52 | 1.06e-02 | 1.00e+00 |
|  | 17 | 7909776 | 7910040 | PDFSTATSENSEMBLUCSC | 265 | 4 | 16387 | gene;island | 0.90035 | 0.76153 | Not queried | 1.52 | 2.00e-01 | 1.00e+00 |
|  | 6 | 19804711 | 19805404 | PDFSTATSENSEMBLUCSC | 694 | 7 | 35419 | island | 0.01461 | 0.05984 | Not queried | 1.52 | 2.59e-01 | 1.00e+00 |
|  | 4 | 3512250 | 3512607 | PDFSTATSENSEMBLUCSC | 358 | 4 | 30794 | island | 0.98041 | 0.92448 | Not queried | 1.52 | 2.86e-02 | 1.00e+00 |
|  | 13 | 21140606 | 21140776 | PDFSTATSENSEMBLUCSC | 171 | 4 | 11305 | tss;island | 0.01865 | 0.00871 | Not queried | 1.52 | 3.43e-01 | 1.00e+00 |
|  | 8 | 1106401 | 1106743 | PDFSTATSENSEMBLUCSC | 343 | 5 | 39950 | island | 0.89569 | 0.90187 | Not queried | 1.52 | 5.48e-01 | 1.00e+00 |
|  | 11 | 44324759 | 44325029 | PDFSTATSENSEMBLUCSC | 271 | 5 | 6571 | gene;island | 0.89146 | 0.70007 | Not queried | 1.52 | 1.51e-01 | 1.00e+00 |
|  | 10 | 95848649 | 95848841 | PDFSTATSENSEMBLUCSC | 193 | 4 | 4671 | gene | 0.60929 | 0.35723 | Not queried | 1.51 | 2.00e-01 | 1.00e+00 |
|  | 8 | 56791576 | 56791882 | PDFSTATSENSEMBLUCSC | 307 | 5 | 39645 | tss;island | 0.05029 | 0.00964 | Not queried | 1.51 | 3.10e-01 | 1.00e+00 |
|  | 1 | 35226707 | 35227272 | PDFSTATSENSEMBLUCSC | 566 | 6 | 411 | gene;island | 0.85364 | 0.64478 | Not queried | 1.51 | 4.11e-02 | 1.00e+00 |
|  | 15 | 68113328 | 68113580 | PDFSTATSENSEMBLUCSC | 253 | 4 | 14223 | island | 0.02101 | 0.04019 | Not queried | 1.51 | 2.00e-01 | 1.00e+00 |
|  | 14 | 94393497 | 94393816 | PDFSTATSENSEMBLUCSC | 320 | 5 | 12165 | gene;tss | 0.81382 | 0.92035 | Not queried | 1.51 | 9.52e-02 | 1.00e+00 |
|  | 9 | 74764263 | 74764504 | PDFSTATSENSEMBLUCSC | 242 | 4 | 40828 | island | 0.14324 | 0.31828 | Not queried | 1.51 | 5.71e-02 | 1.00e+00 |
|  | 17 | 47840643 | 47841033 | PDFSTATSENSEMBLUCSC | 391 | 5 | 16640 | gene;island | 0.03247 | 0.01406 | Not queried | 1.51 | 8.41e-01 | 1.00e+00 |
|  | 19 | 44455140 | 44455383 | PDFSTATSENSEMBLUCSC | 244 | 8 | 20801 | tss | 0.00689 | 0.03457 | Not queried | 1.51 | 1.95e-01 | 1.00e+00 |
|  | 6 | 3849688 | 3849818 | PDFSTATSENSEMBLUCSC | 131 | 5 | 33474 | gene | 0.02970 | 0.07356 | Not queried | 1.51 | 2.22e-01 | 1.00e+00 |
|  | 12 | 123319216 | 123319366 | PDFSTATSENSEMBLUCSC | 151 | 4 | 10063 | tss;island | 0.06510 | 0.02244 | Not queried | 1.51 | 3.43e-01 | 1.00e+00 |
|  | 6 | 166868856 | 166869078 | PDFSTATSENSEMBLUCSC | 223 | 4 | 34479 | gene;island | 0.96738 | 0.90917 | Not queried | 1.51 | 2.86e-02 | 1.00e+00 |
|  | 15 | 23932620 | 23933079 | PDFSTATSENSEMBLUCSC | 460 | 5 | 13487 | tss;island | 0.21595 | 0.47438 | Not queried | 1.51 | 4.21e-01 | 1.00e+00 |
|  | 2 | 102091195 | 102091321 | PDFSTATSENSEMBLUCSC | 127 | 6 | 23510 | tss | 0.34811 | 0.57651 | Not queried | 1.51 | 4.33e-03 | 1.00e+00 |
|  | X | 14048147 | 14048281 | PDFSTATSENSEMBLUCSC | 135 | 8 | 41195 | tss | 0.02309 | 0.03965 | Not queried | 1.51 | 1.30e-01 | 1.00e+00 |
|  | 8 | 57233269 | 57233391 | PDFSTATSENSEMBLUCSC | 123 | 4 | 39649 | tss;island | 0.47794 | 0.78842 | Not queried | 1.51 | 3.43e-01 | 1.00e+00 |
|  | 5 | 141392652 | 141392853 | PDFSTATSENSEMBLUCSC | 202 | 6 | 32335 | tss | 0.03047 | 0.08252 | Not queried | 1.51 | 4.11e-02 | 1.00e+00 |
|  | 16 | 30075788 | 30076044 | PDFSTATSENSEMBLUCSC | 257 | 5 | 14687 | gene;tss;island | 0.07452 | 0.01965 | Not queried | 1.51 | 1.51e-01 | 1.00e+00 |
|  | 11 | 89463427 | 89463689 | PDFSTATSENSEMBLUCSC | 263 | 4 | 8698 | island | 0.88017 | 0.70501 | Not queried | 1.51 | 3.43e-01 | 1.00e+00 |
|  | 16 | 86588418 | 86588622 | PDFSTATSENSEMBLUCSC | 205 | 4 | 14855 | gene;tss;island | 0.02835 | 0.04165 | Not queried | 1.51 | 4.86e-01 | 1.00e+00 |
|  | 6 | 28753834 | 28754198 | PDFSTATSENSEMBLUCSC | 365 | 5 | 35531 | island | 0.58983 | 0.33576 | Not queried | 1.51 | 7.94e-03 | 1.00e+00 |
|  | 22 | 27053195 | 27053934 | PDFSTATSENSEMBLUCSC | 740 | 15 | 27336 | island | 0.08329 | 0.02876 | Not queried | 1.51 | 4.27e-03 | 1.00e+00 |
|  | 10 | 7670683 | 7670827 | PDFSTATSENSEMBLUCSC | 145 | 4 | 4472 | gene | 0.71842 | 0.89981 | Not queried | 1.51 | 2.00e-01 | 1.00e+00 |
|  | 8 | 143359879 | 143360290 | PDFSTATSENSEMBLUCSC | 412 | 4 | 39393 | gene;island | 0.63444 | 0.81723 | Not queried | 1.51 | 1.14e-01 | 1.00e+00 |
|  | 16 | 3085468 | 3085982 | PDFSTATSENSEMBLUCSC | 515 | 6 | 15603 | island | 0.75331 | 0.51759 | Not queried | 1.51 | 3.94e-01 | 1.00e+00 |
|  | 12 | 57634549 | 57634855 | PDFSTATSENSEMBLUCSC | 307 | 7 | 9789 | tss | 0.04778 | 0.05778 | Not queried | 1.51 | 9.73e-02 | 1.00e+00 |
|  | 2 | 118616155 | 118616576 | PDFSTATSENSEMBLUCSC | 422 | 5 | 24600 | island | 0.65514 | 0.41826 | Not queried | 1.51 | 6.90e-01 | 1.00e+00 |
|  | 10 | 90712684 | 90712948 | PDFSTATSENSEMBLUCSC | 265 | 6 | 4654 | gene;tss | 0.61657 | 0.82262 | Not queried | 1.51 | 8.66e-03 | 1.00e+00 |
|  | 4 | 3671067 | 3671697 | PDFSTATSENSEMBLUCSC | 631 | 5 | 30801 | island | 0.89038 | 0.94388 | Not queried | 1.51 | 4.21e-01 | 1.00e+00 |
|  | 10 | 18940606 | 18940907 | PDFSTATSENSEMBLUCSC | 302 | 10 | 4959 | tss;island | 0.05168 | 0.02214 | Not queried | 1.51 | 2.32e-02 | 1.00e+00 |
|  | 10 | 31892464 | 31892769 | PDFSTATSENSEMBLUCSC | 306 | 4 | 5634 | island | 0.06681 | 0.19590 | Not queried | 1.51 | 2.00e-01 | 1.00e+00 |
|  | 16 | 66599843 | 66600105 | PDFSTATSENSEMBLUCSC | 263 | 4 | 14750 | gene | 0.85681 | 0.95256 | Not queried | 1.51 | 2.00e-01 | 1.00e+00 |
|  | 1 | 39281613 | 39282003 | PDFSTATSENSEMBLUCSC | 391 | 4 | 3085 | island | 0.91232 | 0.77572 | Not queried | 1.51 | 3.43e-01 | 1.00e+00 |
|  | 6 | 74161911 | 74162142 | PDFSTATSENSEMBLUCSC | 232 | 7 | 36194 | island | 0.68659 | 0.50973 | Not queried | 1.51 | 5.83e-04 | 1.00e+00 |
|  | 6 | 5996893 | 5997375 | PDFSTATSENSEMBLUCSC | 483 | 6 | 35340 | island | 0.01355 | 0.02736 | Not queried | 1.51 | 1.80e-01 | 1.00e+00 |
|  | 19 | 4851963 | 4852205 | PDFSTATSENSEMBLUCSC | 243 | 4 | 19705 | gene;island | 0.79732 | 0.87731 | Not queried | 1.51 | 6.86e-01 | 1.00e+00 |
|  | 10 | 44070149 | 44070200 | PDFSTATSENSEMBLUCSC | 52 | 4 | 5035 | tss | 0.12255 | 0.05124 | Not queried | 1.51 | 2.00e-01 | 1.00e+00 |
|  | 22 | 32555255 | 32555310 | PDFSTATSENSEMBLUCSC | 56 | 4 | 27045 | tss | 0.93282 | 0.73576 | Not queried | 1.51 | 2.00e-01 | 1.00e+00 |
|  | 1 | 170630397 | 170630734 | PDFSTATSENSEMBLUCSC | 338 | 5 | 3949 | island | 0.24127 | 0.02754 | Not queried | 1.51 | 2.22e-01 | 1.00e+00 |
|  | 12 | 132982231 | 132982483 | PDFSTATSENSEMBLUCSC | 253 | 4 | 11022 | island | 0.70476 | 0.83926 | Not queried | 1.50 | 1.14e-01 | 1.00e+00 |
|  | 2 | 176993017 | 176993320 | PDFSTATSENSEMBLUCSC | 304 | 6 | 23700 | tss;island | 0.28561 | 0.10149 | Not queried | 1.50 | 4.33e-03 | 1.00e+00 |
|  | 1 | 75139470 | 75139746 | PDFSTATSENSEMBLUCSC | 277 | 6 | 1693 | tss | 0.40846 | 0.70910 | Not queried | 1.50 | 2.16e-03 | 1.00e+00 |
|  | 10 | 120863603 | 120863861 | PDFSTATSENSEMBLUCSC | 259 | 5 | 6172 | island | 0.01725 | 0.04594 | Not queried | 1.50 | 5.56e-02 | 1.00e+00 |
|  | 6 | 32362551 | 32362744 | PDFSTATSENSEMBLUCSC | 194 | 8 | 34042 | gene | 0.72018 | 0.60131 | Not queried | 1.50 | 1.05e-01 | 1.00e+00 |
|  | 11 | 1326508 | 1326879 | PDFSTATSENSEMBLUCSC | 372 | 4 | 6396 | gene;island | 0.82564 | 0.90218 | Not queried | 1.50 | 5.71e-02 | 1.00e+00 |
|  | 10 | 54074743 | 54075082 | PDFSTATSENSEMBLUCSC | 340 | 4 | 4580 | gene;island | 0.03386 | 0.08448 | Not queried | 1.50 | 2.86e-02 | 1.00e+00 |
|  | 6 | 28778091 | 28778676 | PDFSTATSENSEMBLUCSC | 586 | 9 | 35532 | island | 0.01893 | 0.04019 | Not queried | 1.50 | 3.99e-03 | 1.00e+00 |
|  | 15 | 52030519 | 52030746 | PDFSTATSENSEMBLUCSC | 228 | 4 | 13323 | gene;tss;island | 0.83675 | 0.66266 | Not queried | 1.50 | 5.71e-02 | 1.00e+00 |
|  | 11 | 22214333 | 22214726 | PDFSTATSENSEMBLUCSC | 394 | 6 | 8111 | island | 0.06288 | 0.10492 | Not queried | 1.50 | 6.49e-02 | 1.00e+00 |
|  | 7 | 157467883 | 157468443 | PDFSTATSENSEMBLUCSC | 561 | 7 | 37276 | gene | 0.73586 | 0.90107 | Not queried | 1.50 | 1.28e-01 | 1.00e+00 |
|  | 8 | 27950625 | 27950972 | PDFSTATSENSEMBLUCSC | 348 | 5 | 39197 | gene | 0.00718 | 0.03082 | Not queried | 1.50 | 4.21e-01 | 1.00e+00 |
|  | 12 | 52845930 | 52846088 | PDFSTATSENSEMBLUCSC | 159 | 4 | 9703 | tss | 0.56918 | 0.80331 | Not queried | 1.50 | 2.86e-02 | 1.00e+00 |
|  | 20 | 982623 | 983130 | PDFSTATSENSEMBLUCSC | 508 | 8 | 25891 | island | 0.19342 | 0.39378 | Not queried | 1.50 | 5.74e-01 | 1.00e+00 |
|  | 7 | 27194614 | 27195036 | PDFSTATSENSEMBLUCSC | 423 | 5 | 36873 | gene;island | 0.54801 | 0.75202 | Not queried | 1.50 | 1.51e-01 | 1.00e+00 |
|  | 2 | 73964587 | 73964834 | PDFSTATSENSEMBLUCSC | 248 | 6 | 23405 | tss | 0.13399 | 0.42365 | Not queried | 1.50 | 2.40e-01 | 1.00e+00 |
|  | 19 | 13124604 | 13125281 | PDFSTATSENSEMBLUCSC | 678 | 7 | 19839 | gene;island | 0.24263 | 0.08341 | Not queried | 1.50 | 1.28e-01 | 1.00e+00 |
|  | X | 37404389 | 37404647 | PDFSTATSENSEMBLUCSC | 259 | 4 | 41714 | island | 0.75103 | 0.89506 | Not queried | 1.50 | 1.14e-01 | 1.00e+00 |
|  | X | 69509542 | 69510172 | PDFSTATSENSEMBLUCSC | 631 | 12 | 41367 | tss;island | 0.08638 | 0.03004 | Not queried | 1.50 | 2.32e-03 | 1.00e+00 |
|  | 17 | 77375461 | 77375842 | PDFSTATSENSEMBLUCSC | 382 | 5 | 16805 | gene | 0.55021 | 0.75687 | Not queried | 1.50 | 3.17e-02 | 1.00e+00 |
|  | 11 | 74442547 | 74442900 | PDFSTATSENSEMBLUCSC | 354 | 4 | 7540 | tss;island | 0.46462 | 0.16013 | Not queried | 1.50 | 4.86e-01 | 1.00e+00 |
|  | 3 | 49203769 | 49203975 | PDFSTATSENSEMBLUCSC | 207 | 7 | 29067 | island | 0.67909 | 0.07934 | Not queried | 1.50 | 8.05e-01 | 1.00e+00 |
|  | 1 | 40420503 | 40420692 | PDFSTATSENSEMBLUCSC | 190 | 9 | 1528 | tss | 0.10349 | 0.03926 | Not queried | 1.50 | 1.90e-01 | 1.00e+00 |
|  | 19 | 1816188 | 1816405 | PDFSTATSENSEMBLUCSC | 218 | 8 | 19627 | gene;island | 0.85177 | 0.91640 | Not queried | 1.50 | 2.81e-02 | 1.00e+00 |
|  | 22 | 41763093 | 41763370 | PDFSTATSENSEMBLUCSC | 278 | 7 | 27128 | tss | 0.39376 | 0.59252 | Not queried | 1.50 | 2.62e-02 | 1.00e+00 |
|  | 10 | 101088533 | 101088828 | PDFSTATSENSEMBLUCSC | 296 | 4 | 5263 | tss | 0.26660 | 0.58511 | Not queried | 1.50 | 3.43e-01 | 1.00e+00 |
|  | 8 | 38585881 | 38586183 | PDFSTATSENSEMBLUCSC | 303 | 4 | 39222 | gene | 0.13812 | 0.35075 | Not queried | 1.50 | 2.00e-01 | 1.00e+00 |
|  | 17 | 7137923 | 7138078 | PDFSTATSENSEMBLUCSC | 156 | 5 | 17031 | tss | 0.02083 | 0.03932 | Not queried | 1.50 | 1.51e-01 | 1.00e+00 |
|  | 10 | 79793353 | 79794011 | PDFSTATSENSEMBLUCSC | 659 | 11 | 5857 | island | 0.01944 | 0.03017 | Not queried | 1.50 | 1.16e-01 | 1.00e+00 |
|  | 5 | 87969157 | 87969330 | PDFSTATSENSEMBLUCSC | 174 | 4 | 31621 | gene;tss;island | 0.08003 | 0.02315 | Not queried | 1.50 | 1.14e-01 | 1.00e+00 |
|  | X | 153561128 | 153561349 | PDFSTATSENSEMBLUCSC | 222 | 4 | 42135 | island | 0.93750 | 0.82855 | Not queried | 1.50 | 1.14e-01 | 1.00e+00 |
|  | 10 | 102748541 | 102749041 | PDFSTATSENSEMBLUCSC | 501 | 5 | 4703 | gene;island | 0.72465 | 0.89852 | Not queried | 1.50 | 3.17e-02 | 1.00e+00 |
|  | X | 28605581 | 28605646 | PDFSTATSENSEMBLUCSC | 66 | 4 | 41241 | tss | 0.13562 | 0.05895 | Not queried | 1.50 | 4.86e-01 | 1.00e+00 |
|  | 18 | 61034556 | 61034655 | PDFSTATSENSEMBLUCSC | 100 | 4 | 19275 | tss | 0.00532 | 0.02071 | Not queried | 1.50 | 2.00e-01 | 1.00e+00 |
|  | 19 | 40732691 | 40732902 | PDFSTATSENSEMBLUCSC | 212 | 8 | 20748 | tss | 0.47656 | 0.75226 | Not queried | 1.49 | 6.99e-03 | 1.00e+00 |
|  | 16 | 734504 | 734844 | PDFSTATSENSEMBLUCSC | 341 | 7 | 14945 | tss | 0.07410 | 0.18486 | Not queried | 1.49 | 3.83e-01 | 1.00e+00 |
|  | 17 | 46669455 | 46669781 | PDFSTATSENSEMBLUCSC | 327 | 8 | 16619 | gene;island | 0.86298 | 0.62479 | Not queried | 1.49 | 6.99e-03 | 1.00e+00 |
|  | 7 | 150715634 | 150716072 | PDFSTATSENSEMBLUCSC | 439 | 4 | 37219 | gene;island | 0.55919 | 0.73457 | Not queried | 1.49 | 1.14e-01 | 1.00e+00 |
|  | 1 | 3163341 | 3163710 | PDFSTATSENSEMBLUCSC | 370 | 4 | 112 | gene;island | 0.76133 | 0.86702 | Not queried | 1.49 | 3.43e-01 | 1.00e+00 |
|  | 8 | 145625497 | 145625814 | PDFSTATSENSEMBLUCSC | 318 | 5 | 39457 | gene;island | 0.85885 | 0.93836 | Not queried | 1.49 | 5.56e-02 | 1.00e+00 |
|  | 4 | 13530094 | 13530470 | PDFSTATSENSEMBLUCSC | 377 | 4 | 30879 | island | 0.91552 | 0.94511 | Not queried | 1.49 | 4.86e-01 | 1.00e+00 |
|  | 2 | 136498791 | 136499146 | PDFSTATSENSEMBLUCSC | 356 | 4 | 23608 | tss;island | 0.10606 | 0.26209 | Not queried | 1.49 | 8.86e-01 | 1.00e+00 |
|  | 17 | 7137795 | 7138078 | PDFSTATSENSEMBLUCSC | 284 | 7 | 17891 | island | 0.02083 | 0.03932 | Not queried | 1.49 | 1.28e-01 | 1.00e+00 |
|  | 13 | 113622539 | 113622750 | PDFSTATSENSEMBLUCSC | 212 | 7 | 11517 | tss;island | 0.67044 | 0.78131 | Not queried | 1.49 | 5.30e-02 | 1.00e+00 |
|  | 19 | 1816254 | 1816405 | PDFSTATSENSEMBLUCSC | 152 | 7 | 20276 | tss | 0.86582 | 0.91797 | Not queried | 1.49 | 3.79e-02 | 1.00e+00 |
|  | 2 | 10444432 | 10445016 | PDFSTATSENSEMBLUCSC | 585 | 6 | 22529 | gene;island | 0.34234 | 0.26170 | Not queried | 1.49 | 2.40e-01 | 1.00e+00 |
|  | X | 68047520 | 68049085 | PDFSTATSENSEMBLUCSC | 1566 | 26 | 41838 | island | 0.09770 | 0.03950 | Not queried | 1.49 | 1.24e-03 | 1.00e+00 |
|  | 17 | 39728362 | 39728628 | PDFSTATSENSEMBLUCSC | 267 | 5 | 17307 | tss | 0.17510 | 0.36579 | Not queried | 1.49 | 7.94e-03 | 1.00e+00 |
|  | 12 | 54366760 | 54366875 | PDFSTATSENSEMBLUCSC | 116 | 4 | 9724 | tss | 0.21030 | 0.44002 | Not queried | 1.49 | 3.43e-01 | 1.00e+00 |
|  | 6 | 41168941 | 41169171 | PDFSTATSENSEMBLUCSC | 231 | 6 | 34903 | tss | 0.71544 | 0.52950 | Not queried | 1.49 | 3.10e-01 | 1.00e+00 |
|  | 8 | 23423734 | 23423960 | PDFSTATSENSEMBLUCSC | 227 | 4 | 39180 | gene | 0.82321 | 0.93139 | Not queried | 1.49 | 5.71e-02 | 1.00e+00 |
|  | 11 | 10772408 | 10772781 | PDFSTATSENSEMBLUCSC | 374 | 9 | 7128 | tss | 0.01187 | 0.02904 | Not queried | 1.49 | 1.42e-02 | 1.00e+00 |
|  | 11 | 10772408 | 10773025 | PDFSTATSENSEMBLUCSC | 618 | 11 | 8039 | island | 0.01187 | 0.03222 | Not queried | 1.49 | 1.28e-02 | 1.00e+00 |
|  | 5 | 140743780 | 140744155 | PDFSTATSENSEMBLUCSC | 376 | 6 | 31766 | gene;island | 0.70241 | 0.87744 | Not queried | 1.49 | 2.16e-03 | 1.00e+00 |
|  | 10 | 93334974 | 93335196 | PDFSTATSENSEMBLUCSC | 223 | 4 | 4658 | gene | 0.79990 | 0.64396 | Not queried | 1.49 | 3.43e-01 | 1.00e+00 |
|  | 1 | 50513451 | 50513661 | PDFSTATSENSEMBLUCSC | 211 | 4 | 1611 | tss | 0.02372 | 0.07321 | Not queried | 1.48 | 6.86e-01 | 1.00e+00 |
|  | 12 | 56109593 | 56110162 | PDFSTATSENSEMBLUCSC | 570 | 8 | 10482 | island | 0.06742 | 0.04995 | Not queried | 1.48 | 4.42e-01 | 1.00e+00 |
|  | 10 | 52834498 | 52835078 | PDFSTATSENSEMBLUCSC | 581 | 5 | 4577 | gene;island | 0.02909 | 0.07735 | Not queried | 1.48 | 5.56e-02 | 1.00e+00 |
|  | 11 | 108464380 | 108464498 | PDFSTATSENSEMBLUCSC | 119 | 4 | 7653 | tss | 0.01065 | 0.01975 | Not queried | 1.48 | 1.14e-01 | 1.00e+00 |
|  | 7 | 99595095 | 99595458 | PDFSTATSENSEMBLUCSC | 364 | 5 | 38678 | island | 0.30660 | 0.57695 | Not queried | 1.48 | 1.51e-01 | 1.00e+00 |
|  | 3 | 9932180 | 9932217 | PDFSTATSENSEMBLUCSC | 38 | 5 | 28092 | tss | 0.04255 | 0.06170 | Not queried | 1.48 | 4.21e-01 | 1.00e+00 |
|  | 4 | 155253864 | 155254602 | PDFSTATSENSEMBLUCSC | 739 | 6 | 30109 | gene;island | 0.02544 | 0.06710 | Not queried | 1.48 | 1.80e-01 | 1.00e+00 |
|  | 12 | 127765195 | 127765627 | PDFSTATSENSEMBLUCSC | 433 | 4 | 10957 | island | 0.06240 | 0.27624 | Not queried | 1.48 | 1.14e-01 | 1.00e+00 |
|  | 3 | 131080235 | 131080927 | PDFSTATSENSEMBLUCSC | 693 | 9 | 29436 | island | 0.55269 | 0.70881 | Not queried | 1.48 | 1.06e-02 | 1.00e+00 |
|  | 7 | 26191458 | 26191726 | PDFSTATSENSEMBLUCSC | 269 | 6 | 37416 | tss;island | 0.71484 | 0.21802 | Not queried | 1.48 | 3.10e-01 | 1.00e+00 |
|  | 11 | 117667737 | 117668336 | PDFSTATSENSEMBLUCSC | 600 | 10 | 8827 | island | 0.25335 | 0.08279 | Not queried | 1.48 | 1.23e-01 | 1.00e+00 |
|  | 2 | 217236783 | 217237043 | PDFSTATSENSEMBLUCSC | 261 | 10 | 23829 | tss | 0.02502 | 0.05491 | Not queried | 1.48 | 5.24e-02 | 1.00e+00 |
|  | 6 | 74103959 | 74104137 | PDFSTATSENSEMBLUCSC | 179 | 4 | 35005 | tss;island | 0.65682 | 0.91000 | Not queried | 1.48 | 3.43e-01 | 1.00e+00 |
|  | 14 | 102391388 | 102391830 | PDFSTATSENSEMBLUCSC | 443 | 5 | 12190 | gene;island | 0.83123 | 0.89910 | Not queried | 1.48 | 1.51e-01 | 1.00e+00 |
|  | 6 | 31623180 | 31623318 | PDFSTATSENSEMBLUCSC | 139 | 4 | 34761 | tss;island | 0.40775 | 0.64988 | Not queried | 1.48 | 6.86e-01 | 1.00e+00 |
|  | 10 | 180188 | 180755 | PDFSTATSENSEMBLUCSC | 568 | 12 | 5450 | island | 0.00815 | 0.02090 | Not queried | 1.48 | 5.97e-02 | 1.00e+00 |
|  | 7 | 98739496 | 98739782 | PDFSTATSENSEMBLUCSC | 287 | 4 | 37060 | gene;island | 0.05332 | 0.11475 | Not queried | 1.48 | 1.14e-01 | 1.00e+00 |
|  | 10 | 115613834 | 115614022 | PDFSTATSENSEMBLUCSC | 189 | 6 | 5354 | tss;island | 0.00634 | 0.02426 | Not queried | 1.48 | 4.11e-02 | 1.00e+00 |
|  | 2 | 237086398 | 237086782 | PDFSTATSENSEMBLUCSC | 385 | 4 | 25189 | island | 0.16640 | 0.42355 | Not queried | 1.48 | 1.14e-01 | 1.00e+00 |
|  | 3 | 79817125 | 79817333 | PDFSTATSENSEMBLUCSC | 209 | 4 | 28405 | tss | 0.03224 | 0.05854 | Not queried | 1.48 | 4.86e-01 | 1.00e+00 |
|  | 17 | 22191704 | 22191979 | PDFSTATSENSEMBLUCSC | 276 | 4 | 18098 | island | 0.72159 | 0.56653 | Not queried | 1.48 | 2.00e-01 | 1.00e+00 |
|  | 17 | 46831306 | 46831742 | PDFSTATSENSEMBLUCSC | 437 | 4 | 18474 | island | 0.79327 | 0.58039 | Not queried | 1.48 | 2.00e-01 | 1.00e+00 |
|  | 17 | 45894369 | 45894729 | PDFSTATSENSEMBLUCSC | 361 | 4 | 16611 | gene;island | 0.60479 | 0.80725 | Not queried | 1.48 | 2.00e-01 | 1.00e+00 |
|  | 1 | 235811811 | 235812280 | PDFSTATSENSEMBLUCSC | 470 | 6 | 1089 | gene;island | 0.50698 | 0.27198 | Not queried | 1.48 | 3.94e-01 | 1.00e+00 |
|  | 5 | 93077316 | 93077405 | PDFSTATSENSEMBLUCSC | 90 | 4 | 32119 | tss | 0.52417 | 0.75441 | Not queried | 1.48 | 3.43e-01 | 1.00e+00 |
|  | 7 | 2316876 | 2317124 | PDFSTATSENSEMBLUCSC | 249 | 6 | 36736 | gene;island | 0.85175 | 0.94528 | Not queried | 1.48 | 9.31e-02 | 1.00e+00 |
|  | 5 | 139944046 | 139944115 | PDFSTATSENSEMBLUCSC | 70 | 4 | 31730 | gene | 0.00676 | 0.01920 | Not queried | 1.48 | 4.86e-01 | 1.00e+00 |
|  | 5 | 140473857 | 140474578 | PDFSTATSENSEMBLUCSC | 722 | 9 | 33147 | island | 0.02479 | 0.08210 | Not queried | 1.48 | 7.70e-02 | 1.00e+00 |
|  | 22 | 19718172 | 19718480 | PDFSTATSENSEMBLUCSC | 309 | 4 | 27261 | island | 0.02998 | 0.11792 | Not queried | 1.48 | 2.00e-01 | 1.00e+00 |
|  | 4 | 737930 | 738199 | PDFSTATSENSEMBLUCSC | 270 | 4 | 29790 | gene;island | 0.67034 | 0.84838 | Not queried | 1.48 | 2.00e-01 | 1.00e+00 |
|  | 11 | 6518108 | 6518485 | PDFSTATSENSEMBLUCSC | 378 | 7 | 7093 | tss | 0.77989 | 0.88882 | Not queried | 1.48 | 2.59e-01 | 1.00e+00 |
|  | 7 | 75115567 | 75115859 | PDFSTATSENSEMBLUCSC | 293 | 9 | 38520 | island | 0.03495 | 0.01947 | Not queried | 1.48 | 3.87e-01 | 1.00e+00 |
|  | 4 | 185926063 | 185926620 | PDFSTATSENSEMBLUCSC | 558 | 7 | 31411 | island | 0.16625 | 0.06042 | Not queried | 1.48 | 2.59e-01 | 1.00e+00 |
|  | 2 | 232395269 | 232395463 | PDFSTATSENSEMBLUCSC | 195 | 6 | 23904 | tss;island | 0.03501 | 0.14462 | Not queried | 1.48 | 9.31e-02 | 1.00e+00 |
|  | 5 | 178593785 | 178594649 | PDFSTATSENSEMBLUCSC | 865 | 9 | 31870 | gene;island | 0.14410 | 0.51823 | Not queried | 1.48 | 4.89e-01 | 1.00e+00 |
|  | 20 | 62715756 | 62715942 | PDFSTATSENSEMBLUCSC | 187 | 5 | 25873 | tss | 0.64301 | 0.39439 | Not queried | 1.48 | 4.21e-01 | 1.00e+00 |
|  | 11 | 32421514 | 32421845 | PDFSTATSENSEMBLUCSC | 332 | 6 | 6546 | gene | 0.41148 | 0.71859 | Not queried | 1.48 | 4.11e-02 | 1.00e+00 |
|  | 6 | 33037413 | 33037690 | PDFSTATSENSEMBLUCSC | 278 | 6 | 34116 | gene | 0.76172 | 0.56026 | Not queried | 1.48 | 1.32e-01 | 1.00e+00 |
|  | X | 67653315 | 67653925 | PDFSTATSENSEMBLUCSC | 611 | 8 | 41355 | tss | 0.08138 | 0.02132 | Not queried | 1.48 | 1.04e-02 | 1.00e+00 |
|  | 5 | 43037123 | 43037666 | PDFSTATSENSEMBLUCSC | 544 | 6 | 32704 | island | 0.95584 | 0.85952 | Not queried | 1.48 | 9.31e-02 | 1.00e+00 |
|  | 1 | 63791922 | 63792239 | PDFSTATSENSEMBLUCSC | 318 | 6 | 3336 | island | 0.03683 | 0.19314 | Not queried | 1.48 | 4.11e-02 | 1.00e+00 |
|  | 1 | 4221331 | 4221616 | PDFSTATSENSEMBLUCSC | 286 | 5 | 2577 | island | 0.66450 | 0.75010 | Not queried | 1.48 | 2.22e-01 | 1.00e+00 |
|  | 19 | 571107 | 571416 | PDFSTATSENSEMBLUCSC | 310 | 5 | 20237 | tss;island | 0.03831 | 0.13624 | Not queried | 1.48 | 5.56e-02 | 1.00e+00 |
|  | 6 | 31657467 | 31657855 | PDFSTATSENSEMBLUCSC | 389 | 4 | 33875 | gene | 0.65566 | 0.86092 | Not queried | 1.48 | 4.86e-01 | 1.00e+00 |
|  | 6 | 28550343 | 28550437 | PDFSTATSENSEMBLUCSC | 95 | 5 | 33574 | gene | 0.96037 | 0.90654 | Not queried | 1.48 | 9.52e-02 | 1.00e+00 |
|  | 6 | 31930566 | 31930875 | PDFSTATSENSEMBLUCSC | 310 | 6 | 33940 | gene;island | 0.81820 | 0.91941 | Not queried | 1.48 | 9.31e-02 | 1.00e+00 |
|  | 16 | 127772 | 128016 | PDFSTATSENSEMBLUCSC | 245 | 4 | 14493 | gene;tss;island | 0.02121 | 0.07946 | Not queried | 1.48 | 2.00e-01 | 1.00e+00 |
|  | 1 | 154934627 | 154934734 | PDFSTATSENSEMBLUCSC | 108 | 4 | 1967 | tss;island | 0.03134 | 0.02388 | Not queried | 1.47 | 4.86e-01 | 1.00e+00 |
|  | 16 | 88807658 | 88807933 | PDFSTATSENSEMBLUCSC | 276 | 5 | 14884 | gene;island | 0.71263 | 0.87329 | Not queried | 1.47 | 1.59e-02 | 1.00e+00 |
|  | 12 | 48396630 | 48397225 | PDFSTATSENSEMBLUCSC | 596 | 5 | 9082 | gene;island | 0.07683 | 0.25383 | Not queried | 1.47 | 5.56e-02 | 1.00e+00 |
|  | 5 | 172710685 | 172711094 | PDFSTATSENSEMBLUCSC | 410 | 6 | 33347 | island | 0.33636 | 0.06344 | Not queried | 1.47 | 6.49e-02 | 1.00e+00 |
|  | 13 | 113104883 | 113105341 | PDFSTATSENSEMBLUCSC | 459 | 5 | 11890 | island | 0.67066 | 0.81308 | Not queried | 1.47 | 4.21e-01 | 1.00e+00 |
|  | 6 | 32018906 | 32019556 | PDFSTATSENSEMBLUCSC | 651 | 5 | 33955 | gene | 0.59577 | 0.47723 | Not queried | 1.47 | 2.22e-01 | 1.00e+00 |
|  | 15 | 83240578 | 83240791 | PDFSTATSENSEMBLUCSC | 214 | 6 | 13829 | tss | 0.16050 | 0.38083 | Not queried | 1.47 | 9.31e-02 | 1.00e+00 |
|  | 19 | 47217696 | 47218031 | PDFSTATSENSEMBLUCSC | 336 | 5 | 20860 | tss | 0.08970 | 0.27193 | Not queried | 1.47 | 9.52e-02 | 1.00e+00 |
|  | 14 | 100752144 | 100752463 | PDFSTATSENSEMBLUCSC | 320 | 4 | 13107 | island | 0.21061 | 0.40667 | Not queried | 1.47 | 3.43e-01 | 1.00e+00 |
|  | 3 | 131245815 | 131246097 | PDFSTATSENSEMBLUCSC | 283 | 4 | 29439 | island | 0.44508 | 0.74515 | Not queried | 1.47 | 3.43e-01 | 1.00e+00 |
|  | 14 | 37641134 | 37641726 | PDFSTATSENSEMBLUCSC | 593 | 8 | 12040 | gene;island | 0.01816 | 0.04305 | Not queried | 1.47 | 1.05e-01 | 1.00e+00 |
|  | 17 | 46795860 | 46796298 | PDFSTATSENSEMBLUCSC | 439 | 4 | 18463 | island | 0.03838 | 0.13642 | Not queried | 1.47 | 1.14e-01 | 1.00e+00 |
|  | 8 | 145008909 | 145009406 | PDFSTATSENSEMBLUCSC | 498 | 5 | 39434 | gene;island | 0.80373 | 0.89447 | Not queried | 1.47 | 5.56e-02 | 1.00e+00 |
|  | 20 | 26188908 | 26188997 | PDFSTATSENSEMBLUCSC | 90 | 7 | 26027 | island | 0.06960 | 0.20427 | Not queried | 1.47 | 9.73e-02 | 1.00e+00 |
|  | 16 | 1133074 | 1133378 | PDFSTATSENSEMBLUCSC | 305 | 5 | 15484 | island | 0.84361 | 0.93768 | Not queried | 1.47 | 1.51e-01 | 1.00e+00 |
|  | 2 | 109335627 | 109335769 | PDFSTATSENSEMBLUCSC | 143 | 5 | 23527 | tss;island | 0.01139 | 0.05736 | Not queried | 1.47 | 2.22e-01 | 1.00e+00 |
|  | 6 | 37225949 | 37226214 | PDFSTATSENSEMBLUCSC | 266 | 5 | 34256 | gene;tss;island | 0.00808 | 0.01278 | Not queried | 1.47 | 9.52e-02 | 1.00e+00 |
|  | 2 | 130956098 | 130956271 | PDFSTATSENSEMBLUCSC | 174 | 7 | 23588 | tss | 0.96692 | 0.86452 | Not queried | 1.47 | 2.09e-01 | 1.00e+00 |
|  | 1 | 181454975 | 181455181 | PDFSTATSENSEMBLUCSC | 207 | 4 | 906 | gene;island | 0.03980 | 0.08227 | Not queried | 1.47 | 2.86e-02 | 1.00e+00 |
|  | 13 | 112200641 | 112201036 | PDFSTATSENSEMBLUCSC | 396 | 6 | 11868 | island | 0.69463 | 0.84585 | Not queried | 1.47 | 2.40e-01 | 1.00e+00 |
|  | 5 | 128300967 | 128301185 | PDFSTATSENSEMBLUCSC | 219 | 6 | 32201 | tss | 0.02201 | 0.04869 | Not queried | 1.47 | 4.85e-01 | 1.00e+00 |
|  | 3 | 52279403 | 52279700 | PDFSTATSENSEMBLUCSC | 298 | 5 | 28333 | tss;island | 0.31840 | 0.28724 | Not queried | 1.47 | 5.48e-01 | 1.00e+00 |
|  | 20 | 30062970 | 30063249 | PDFSTATSENSEMBLUCSC | 280 | 8 | 26031 | island | 0.05603 | 0.03085 | Not queried | 1.47 | 4.99e-02 | 1.00e+00 |
|  | 19 | 18721531 | 18721840 | PDFSTATSENSEMBLUCSC | 310 | 4 | 21753 | island | 0.17423 | 0.06686 | Not queried | 1.47 | 6.86e-01 | 1.00e+00 |
|  | 4 | 2470698 | 2470786 | PDFSTATSENSEMBLUCSC | 89 | 4 | 30203 | tss | 0.01010 | 0.08980 | Not queried | 1.47 | 1.14e-01 | 1.00e+00 |
|  | 4 | 2470698 | 2471025 | PDFSTATSENSEMBLUCSC | 328 | 6 | 30768 | island | 0.01010 | 0.05884 | Not queried | 1.47 | 6.49e-02 | 1.00e+00 |
|  | 19 | 55691730 | 55692003 | PDFSTATSENSEMBLUCSC | 274 | 7 | 21036 | tss;island | 0.02298 | 0.08796 | Not queried | 1.47 | 2.09e-01 | 1.00e+00 |
|  | X | 69352975 | 69353185 | PDFSTATSENSEMBLUCSC | 211 | 6 | 41364 | tss;island | 0.18620 | 0.05937 | Not queried | 1.46 | 6.49e-02 | 1.00e+00 |
|  | 8 | 49293379 | 49293600 | PDFSTATSENSEMBLUCSC | 222 | 4 | 40217 | island | 0.16457 | 0.07109 | Not queried | 1.46 | 4.86e-01 | 1.00e+00 |
|  | 17 | 40936498 | 40936820 | PDFSTATSENSEMBLUCSC | 323 | 5 | 16553 | gene;island | 0.91945 | 0.80125 | Not queried | 1.46 | 7.94e-03 | 1.00e+00 |
|  | X | 51139025 | 51139247 | PDFSTATSENSEMBLUCSC | 223 | 4 | 41800 | island | 0.16569 | 0.03925 | Not queried | 1.46 | 1.14e-01 | 1.00e+00 |
|  | 19 | 3207921 | 3208130 | PDFSTATSENSEMBLUCSC | 210 | 4 | 19676 | gene;island | 0.85729 | 0.94101 | Not queried | 1.46 | 3.43e-01 | 1.00e+00 |
|  | 18 | 70211774 | 70211974 | PDFSTATSENSEMBLUCSC | 201 | 6 | 19284 | tss;island | 0.18678 | 0.36116 | Not queried | 1.46 | 5.89e-01 | 1.00e+00 |
|  | 6 | 31473264 | 31473581 | PDFSTATSENSEMBLUCSC | 318 | 4 | 33819 | gene | 0.90828 | 0.66132 | Not queried | 1.46 | 3.43e-01 | 1.00e+00 |
|  | 6 | 101846650 | 101847058 | PDFSTATSENSEMBLUCSC | 409 | 12 | 36308 | island | 0.09973 | 0.03972 | Not queried | 1.46 | 3.64e-03 | 1.00e+00 |
|  | 17 | 34058433 | 34058623 | PDFSTATSENSEMBLUCSC | 191 | 5 | 17241 | tss;island | 0.12613 | 0.05868 | Not queried | 1.46 | 5.56e-02 | 1.00e+00 |
|  | 2 | 97524767 | 97525009 | PDFSTATSENSEMBLUCSC | 243 | 4 | 23487 | tss;island | 0.56419 | 0.46934 | Not queried | 1.46 | 6.86e-01 | 1.00e+00 |
|  | 6 | 30955358 | 30955425 | PDFSTATSENSEMBLUCSC | 68 | 5 | 33783 | gene | 0.88821 | 0.69389 | Not queried | 1.46 | 9.52e-02 | 1.00e+00 |
|  | 2 | 128453108 | 128453484 | PDFSTATSENSEMBLUCSC | 377 | 5 | 24661 | island | 0.93265 | 0.82171 | Not queried | 1.46 | 1.51e-01 | 1.00e+00 |
|  | 22 | 19710880 | 19711051 | PDFSTATSENSEMBLUCSC | 172 | 6 | 26748 | gene;tss;island | 0.31022 | 0.57929 | Not queried | 1.46 | 2.16e-03 | 1.00e+00 |
|  | 17 | 40120250 | 40120496 | PDFSTATSENSEMBLUCSC | 247 | 4 | 16538 | gene;island | 0.85850 | 0.94829 | Not queried | 1.46 | 3.43e-01 | 1.00e+00 |
|  | 6 | 74063982 | 74064594 | PDFSTATSENSEMBLUCSC | 613 | 8 | 35002 | tss;island | 0.62920 | 0.85902 | Not queried | 1.46 | 3.79e-02 | 1.00e+00 |
|  | 2 | 182549353 | 182549604 | PDFSTATSENSEMBLUCSC | 252 | 5 | 24900 | island | 0.38064 | 0.61664 | Not queried | 1.46 | 2.22e-01 | 1.00e+00 |
|  | 16 | 1404834 | 1405223 | PDFSTATSENSEMBLUCSC | 390 | 4 | 14537 | gene;island | 0.81693 | 0.93094 | Not queried | 1.46 | 2.00e-01 | 1.00e+00 |
|  | 1 | 3772978 | 3773316 | PDFSTATSENSEMBLUCSC | 339 | 4 | 147 | gene;tss;island | 0.01562 | 0.00808 | Not queried | 1.46 | 4.86e-01 | 1.00e+00 |
|  | 7 | 1130967 | 1131133 | PDFSTATSENSEMBLUCSC | 167 | 4 | 36687 | gene;island | 0.73642 | 0.83751 | Not queried | 1.46 | 6.86e-01 | 1.00e+00 |
|  | 19 | 49220102 | 49220485 | PDFSTATSENSEMBLUCSC | 384 | 6 | 20099 | gene;island | 0.69093 | 0.38514 | Not queried | 1.46 | 2.16e-03 | 1.00e+00 |
|  | 2 | 66809780 | 66809985 | PDFSTATSENSEMBLUCSC | 206 | 4 | 24320 | island | 0.29771 | 0.49229 | Not queried | 1.46 | 5.71e-02 | 1.00e+00 |
|  | 17 | 6023675 | 6024017 | PDFSTATSENSEMBLUCSC | 343 | 4 | 16335 | gene;island | 0.63249 | 0.79106 | Not queried | 1.46 | 2.86e-02 | 1.00e+00 |
|  | 15 | 72977851 | 72978371 | PDFSTATSENSEMBLUCSC | 521 | 5 | 13381 | gene;tss;island | 0.02801 | 0.02687 | Not queried | 1.46 | 4.21e-01 | 1.00e+00 |
|  | 1 | 159825552 | 159825761 | PDFSTATSENSEMBLUCSC | 210 | 4 | 836 | gene;tss;island | 0.82617 | 0.69825 | Not queried | 1.46 | 1.14e-01 | 1.00e+00 |
|  | 2 | 169658881 | 169659047 | PDFSTATSENSEMBLUCSC | 167 | 4 | 23661 | tss | 0.72508 | 0.86393 | Not queried | 1.46 | 6.86e-01 | 1.00e+00 |
|  | 19 | 54485321 | 54485509 | PDFSTATSENSEMBLUCSC | 189 | 6 | 20175 | gene;tss;island | 0.17492 | 0.33863 | Not queried | 1.46 | 3.94e-01 | 1.00e+00 |
|  | 1 | 113216774 | 113217181 | PDFSTATSENSEMBLUCSC | 408 | 6 | 3597 | island | 0.04899 | 0.16481 | Not queried | 1.46 | 1.80e-01 | 1.00e+00 |
|  | 17 | 21454528 | 21455185 | PDFSTATSENSEMBLUCSC | 658 | 7 | 18092 | island | 0.02385 | 0.07588 | Not queried | 1.46 | 2.59e-01 | 1.00e+00 |
|  | 15 | 78914083 | 78914439 | PDFSTATSENSEMBLUCSC | 357 | 4 | 13809 | tss;island | 0.85091 | 0.62488 | Not queried | 1.46 | 1.14e-01 | 1.00e+00 |
|  | 14 | 45553114 | 45553636 | PDFSTATSENSEMBLUCSC | 523 | 5 | 12785 | island | 0.01808 | 0.03878 | Not queried | 1.46 | 5.56e-02 | 1.00e+00 |
|  | 18 | 19284796 | 19285051 | PDFSTATSENSEMBLUCSC | 256 | 9 | 19166 | tss | 0.03099 | 0.10698 | Not queried | 1.46 | 1.13e-01 | 1.00e+00 |
|  | 16 | 1365584 | 1365915 | PDFSTATSENSEMBLUCSC | 332 | 4 | 14533 | gene;island | 0.73439 | 0.89039 | Not queried | 1.46 | 4.86e-01 | 1.00e+00 |
|  | 20 | 62037503 | 62037758 | PDFSTATSENSEMBLUCSC | 256 | 4 | 26311 | island | 0.81749 | 0.92678 | Not queried | 1.46 | 1.14e-01 | 1.00e+00 |
|  | 12 | 114847438 | 114847641 | PDFSTATSENSEMBLUCSC | 204 | 4 | 10010 | tss;island | 0.65839 | 0.34937 | Not queried | 1.46 | 1.14e-01 | 1.00e+00 |
|  | 3 | 62355884 | 62356323 | PDFSTATSENSEMBLUCSC | 440 | 4 | 27803 | gene;island | 0.15572 | 0.13585 | Not queried | 1.46 | 8.86e-01 | 1.00e+00 |
|  | 17 | 13505433 | 13505799 | PDFSTATSENSEMBLUCSC | 367 | 5 | 17111 | tss;island | 0.02710 | 0.02579 | Not queried | 1.46 | 5.48e-01 | 1.00e+00 |
|  | 17 | 185102 | 185263 | PDFSTATSENSEMBLUCSC | 162 | 4 | 16256 | gene;island | 0.93567 | 0.85698 | Not queried | 1.46 | 3.43e-01 | 1.00e+00 |
|  | 17 | 62084205 | 62084359 | PDFSTATSENSEMBLUCSC | 155 | 5 | 16696 | gene | 0.43233 | 0.62125 | Not queried | 1.45 | 2.22e-01 | 1.00e+00 |
|  | 15 | 64387625 | 64388259 | PDFSTATSENSEMBLUCSC | 635 | 9 | 14183 | island | 0.00997 | 0.02142 | Not queried | 1.45 | 2.97e-01 | 1.00e+00 |
|  | 14 | 21737751 | 21738093 | PDFSTATSENSEMBLUCSC | 343 | 6 | 12254 | tss;island | 0.00618 | 0.02059 | Not queried | 1.45 | 1.52e-02 | 1.00e+00 |
|  | 8 | 122654023 | 122654328 | PDFSTATSENSEMBLUCSC | 306 | 4 | 39352 | gene;tss;island | 0.01526 | 0.04143 | Not queried | 1.45 | 3.43e-01 | 1.00e+00 |
|  | 16 | 12997467 | 12997684 | PDFSTATSENSEMBLUCSC | 218 | 4 | 14643 | gene;island | 0.04632 | 0.14085 | Not queried | 1.45 | 3.43e-01 | 1.00e+00 |
|  | 16 | 50875140 | 50875496 | PDFSTATSENSEMBLUCSC | 357 | 4 | 15883 | island | 0.25802 | 0.56406 | Not queried | 1.45 | 2.00e-01 | 1.00e+00 |
|  | 6 | 32826171 | 32826281 | PDFSTATSENSEMBLUCSC | 111 | 4 | 34094 | gene;island | 0.74542 | 0.55004 | Not queried | 1.45 | 4.86e-01 | 1.00e+00 |
|  | 15 | 69323876 | 69324156 | PDFSTATSENSEMBLUCSC | 281 | 5 | 13368 | gene;island | 0.02556 | 0.06696 | Not queried | 1.45 | 3.10e-01 | 1.00e+00 |
|  | 19 | 14224992 | 14225172 | PDFSTATSENSEMBLUCSC | 181 | 5 | 19855 | gene;tss;island | 0.56310 | 0.78905 | Not queried | 1.45 | 9.52e-02 | 1.00e+00 |
|  | 17 | 53315783 | 53316308 | PDFSTATSENSEMBLUCSC | 526 | 5 | 18540 | island | 0.00488 | 0.01716 | Not queried | 1.45 | 4.21e-01 | 1.00e+00 |
|  | 2 | 216177085 | 216177303 | PDFSTATSENSEMBLUCSC | 219 | 4 | 22970 | gene;island | 0.08090 | 0.03906 | Not queried | 1.45 | 5.71e-02 | 1.00e+00 |
|  | 4 | 3517779 | 3518121 | PDFSTATSENSEMBLUCSC | 343 | 4 | 29866 | gene;island | 0.84293 | 0.93912 | Not queried | 1.45 | 3.43e-01 | 1.00e+00 |
|  | 15 | 41805868 | 41806234 | PDFSTATSENSEMBLUCSC | 367 | 5 | 14035 | island | 0.51567 | 0.29162 | Not queried | 1.45 | 1.51e-01 | 1.00e+00 |
|  | 15 | 58357922 | 58357989 | PDFSTATSENSEMBLUCSC | 68 | 4 | 13669 | tss | 0.01209 | 0.03123 | Not queried | 1.45 | 2.00e-01 | 1.00e+00 |
|  | 11 | 2292361 | 2293309 | PDFSTATSENSEMBLUCSC | 949 | 32 | 7044 | tss;island | 0.71152 | 0.84319 | Not queried | 1.45 | 9.20e-05 | 4.73e-01 |
|  | 15 | 53086630 | 53087599 | PDFSTATSENSEMBLUCSC | 970 | 9 | 14125 | island | 0.90753 | 0.76673 | Not queried | 1.45 | 1.06e-02 | 1.00e+00 |
|  | 1 | 247578859 | 247579312 | PDFSTATSENSEMBLUCSC | 454 | 4 | 2364 | tss | 0.53125 | 0.75511 | Not queried | 1.45 | 1.14e-01 | 1.00e+00 |
|  | 22 | 37252282 | 37252652 | PDFSTATSENSEMBLUCSC | 371 | 4 | 27417 | island | 0.08612 | 0.18275 | Not queried | 1.45 | 6.86e-01 | 1.00e+00 |
|  | 1 | 206730120 | 206730453 | PDFSTATSENSEMBLUCSC | 334 | 4 | 2210 | tss | 0.08558 | 0.24781 | Not queried | 1.45 | 4.86e-01 | 1.00e+00 |
|  | 17 | 73892982 | 73893209 | PDFSTATSENSEMBLUCSC | 228 | 6 | 18732 | island | 0.05004 | 0.09266 | Not queried | 1.45 | 9.31e-02 | 1.00e+00 |
|  | 1 | 33937968 | 33938226 | PDFSTATSENSEMBLUCSC | 259 | 7 | 1471 | tss | 0.00948 | 0.02547 | Not queried | 1.45 | 1.75e-02 | 1.00e+00 |
|  | 8 | 786658 | 787298 | PDFSTATSENSEMBLUCSC | 641 | 6 | 39942 | island | 0.88397 | 0.95430 | Not queried | 1.45 | 1.80e-01 | 1.00e+00 |
|  | 10 | 47083283 | 47083632 | PDFSTATSENSEMBLUCSC | 350 | 9 | 5701 | island | 0.44558 | 0.64180 | Not queried | 1.45 | 1.23e-03 | 1.00e+00 |
|  | X | 51149400 | 51149742 | PDFSTATSENSEMBLUCSC | 343 | 5 | 41324 | tss;island | 0.76410 | 0.91653 | Not queried | 1.45 | 5.48e-01 | 1.00e+00 |
|  | 6 | 30121774 | 30122593 | PDFSTATSENSEMBLUCSC | 820 | 18 | 33666 | gene | 0.35700 | 0.57667 | Not queried | 1.45 | 9.86e-04 | 1.00e+00 |
|  | 3 | 13520685 | 13521915 | PDFSTATSENSEMBLUCSC | 1231 | 27 | 28859 | island | 0.07325 | 0.03231 | Not queried | 1.45 | 2.37e-02 | 1.00e+00 |
|  | 2 | 219925246 | 219925330 | PDFSTATSENSEMBLUCSC | 85 | 6 | 23852 | tss | 0.02873 | 0.09665 | Not queried | 1.45 | 4.11e-02 | 1.00e+00 |
|  | 4 | 185654506 | 185654737 | PDFSTATSENSEMBLUCSC | 232 | 5 | 30158 | gene;island | 0.01558 | 0.03568 | Not queried | 1.45 | 2.22e-01 | 1.00e+00 |
|  | 1 | 207669003 | 207669334 | PDFSTATSENSEMBLUCSC | 332 | 5 | 2219 | tss;island | 0.39020 | 0.35611 | Not queried | 1.45 | 6.90e-01 | 1.00e+00 |
|  | 3 | 30936239 | 30936531 | PDFSTATSENSEMBLUCSC | 293 | 8 | 28159 | tss | 0.02652 | 0.08838 | Not queried | 1.45 | 2.81e-02 | 1.00e+00 |
|  | 19 | 36236496 | 36236615 | PDFSTATSENSEMBLUCSC | 120 | 4 | 19956 | gene | 0.00821 | 0.00843 | Not queried | 1.45 | 6.86e-01 | 1.00e+00 |
|  | 6 | 31543169 | 31543300 | PDFSTATSENSEMBLUCSC | 132 | 4 | 34755 | tss | 0.37548 | 0.61634 | Not queried | 1.45 | 5.71e-02 | 1.00e+00 |
|  | 16 | 30457273 | 30457524 | PDFSTATSENSEMBLUCSC | 252 | 9 | 15154 | tss | 0.02242 | 0.03028 | Not queried | 1.45 | 9.31e-01 | 1.00e+00 |
|  | 19 | 40562144 | 40562354 | PDFSTATSENSEMBLUCSC | 211 | 5 | 20744 | tss | 0.02776 | 0.07446 | Not queried | 1.45 | 1.51e-01 | 1.00e+00 |
|  | 16 | 22824877 | 22825282 | PDFSTATSENSEMBLUCSC | 406 | 5 | 15102 | tss;island | 0.04120 | 0.08208 | Not queried | 1.45 | 9.52e-02 | 1.00e+00 |
|  | 11 | 62621178 | 62621406 | PDFSTATSENSEMBLUCSC | 229 | 4 | 6631 | gene;tss;island | 0.22696 | 0.15661 | Not queried | 1.45 | 3.43e-01 | 1.00e+00 |
|  | 1 | 184006765 | 184007101 | PDFSTATSENSEMBLUCSC | 337 | 5 | 4024 | island | 0.13134 | 0.05260 | Not queried | 1.45 | 2.22e-01 | 1.00e+00 |
|  | 2 | 101889574 | 101889754 | PDFSTATSENSEMBLUCSC | 181 | 5 | 23508 | tss | 0.70244 | 0.86533 | Not queried | 1.44 | 7.94e-03 | 1.00e+00 |
|  | 7 | 44104860 | 44105434 | PDFSTATSENSEMBLUCSC | 575 | 6 | 38390 | island | 0.23793 | 0.36151 | Not queried | 1.44 | 6.49e-02 | 1.00e+00 |
|  | 6 | 29573014 | 29573377 | PDFSTATSENSEMBLUCSC | 364 | 5 | 33603 | gene | 0.68494 | 0.91528 | Not queried | 1.44 | 3.17e-02 | 1.00e+00 |
|  | 5 | 141293785 | 141294040 | PDFSTATSENSEMBLUCSC | 256 | 4 | 33200 | island | 0.15691 | 0.05869 | Not queried | 1.44 | 1.14e-01 | 1.00e+00 |
|  | 10 | 99257444 | 99257699 | PDFSTATSENSEMBLUCSC | 256 | 4 | 4682 | gene;tss;island | 0.01580 | 0.04163 | Not queried | 1.44 | 2.86e-02 | 1.00e+00 |
|  | 18 | 34340585 | 34340941 | PDFSTATSENSEMBLUCSC | 357 | 4 | 19059 | gene;island | 0.79698 | 0.90858 | Not queried | 1.44 | 2.00e-01 | 1.00e+00 |
|  | 7 | 5390491 | 5390564 | PDFSTATSENSEMBLUCSC | 74 | 4 | 36808 | gene;island | 0.85203 | 0.93806 | Not queried | 1.44 | 2.86e-02 | 1.00e+00 |
|  | 5 | 72746710 | 72746990 | PDFSTATSENSEMBLUCSC | 281 | 4 | 32811 | island | 0.03722 | 0.06047 | Not queried | 1.44 | 6.86e-01 | 1.00e+00 |
|  | 4 | 104641033 | 104641319 | PDFSTATSENSEMBLUCSC | 287 | 6 | 30479 | tss;island | 0.09318 | 0.03873 | Not queried | 1.44 | 1.52e-02 | 1.00e+00 |
|  | 12 | 133007675 | 133008035 | PDFSTATSENSEMBLUCSC | 361 | 5 | 11025 | island | 0.67626 | 0.83635 | Not queried | 1.44 | 4.21e-01 | 1.00e+00 |
|  | 17 | 37843793 | 37844289 | PDFSTATSENSEMBLUCSC | 497 | 8 | 16516 | gene | 0.04468 | 0.01900 | Not queried | 1.44 | 4.99e-02 | 1.00e+00 |
|  | 13 | 47371267 | 47371644 | PDFSTATSENSEMBLUCSC | 378 | 11 | 11689 | island | 0.04706 | 0.09779 | Not queried | 1.44 | 5.57e-02 | 1.00e+00 |
|  | 17 | 46871708 | 46871905 | PDFSTATSENSEMBLUCSC | 198 | 5 | 17440 | tss | 0.78524 | 0.57375 | Not queried | 1.44 | 3.10e-01 | 1.00e+00 |
|  | 16 | 31538556 | 31538946 | PDFSTATSENSEMBLUCSC | 391 | 4 | 15183 | tss | 0.71673 | 0.68431 | Not queried | 1.44 | 8.86e-01 | 1.00e+00 |
|  | X | 107334429 | 107334815 | PDFSTATSENSEMBLUCSC | 387 | 5 | 41073 | gene | 0.00773 | 0.03342 | Not queried | 1.44 | 3.10e-01 | 1.00e+00 |
|  | 4 | 74702421 | 74702728 | PDFSTATSENSEMBLUCSC | 308 | 5 | 29976 | gene;island | 0.11425 | 0.07065 | Not queried | 1.44 | 1.51e-01 | 1.00e+00 |
|  | 10 | 102988831 | 102988889 | PDFSTATSENSEMBLUCSC | 59 | 4 | 5288 | tss;island | 0.04166 | 0.13753 | Not queried | 1.44 | 4.86e-01 | 1.00e+00 |
|  | 19 | 49375485 | 49375640 | PDFSTATSENSEMBLUCSC | 156 | 4 | 20893 | tss | 0.01833 | 0.03922 | Not queried | 1.44 | 4.86e-01 | 1.00e+00 |
|  | 1 | 162838649 | 162838907 | PDFSTATSENSEMBLUCSC | 259 | 4 | 2056 | tss | 0.48754 | 0.71734 | Not queried | 1.44 | 1.14e-01 | 1.00e+00 |
|  | 6 | 26247238 | 26247270 | PDFSTATSENSEMBLUCSC | 33 | 4 | 34636 | tss | 0.88934 | 0.94416 | Not queried | 1.44 | 3.43e-01 | 1.00e+00 |
|  | 2 | 223160102 | 223160569 | PDFSTATSENSEMBLUCSC | 468 | 5 | 23008 | gene;island | 0.12099 | 0.32928 | Not queried | 1.44 | 7.94e-03 | 1.00e+00 |
|  | 4 | 48486087 | 48486472 | PDFSTATSENSEMBLUCSC | 386 | 4 | 29954 | gene;island | 0.45160 | 0.21723 | Not queried | 1.44 | 2.86e-02 | 1.00e+00 |
|  | 12 | 89919186 | 89919388 | PDFSTATSENSEMBLUCSC | 203 | 4 | 9214 | gene;tss;island | 0.01469 | 0.04208 | Not queried | 1.44 | 6.86e-01 | 1.00e+00 |
|  | 19 | 5250376 | 5250655 | PDFSTATSENSEMBLUCSC | 280 | 4 | 19714 | gene;island | 0.70464 | 0.86294 | Not queried | 1.44 | 2.00e-01 | 1.00e+00 |
|  | 5 | 140792511 | 140792992 | PDFSTATSENSEMBLUCSC | 482 | 8 | 31775 | gene;island | 0.01845 | 0.12484 | Not queried | 1.44 | 6.50e-02 | 1.00e+00 |
|  | X | 48366824 | 48367230 | PDFSTATSENSEMBLUCSC | 407 | 9 | 41290 | tss;island | 0.09070 | 0.01885 | Not queried | 1.44 | 3.15e-02 | 1.00e+00 |
|  | 19 | 859330 | 859680 | PDFSTATSENSEMBLUCSC | 351 | 6 | 21144 | island | 0.78725 | 0.62644 | Not queried | 1.44 | 2.16e-03 | 1.00e+00 |
|  | 16 | 1451278 | 1451656 | PDFSTATSENSEMBLUCSC | 379 | 4 | 14541 | gene;island | 0.80194 | 0.88407 | Not queried | 1.44 | 4.86e-01 | 1.00e+00 |
|  | 8 | 914335 | 914572 | PDFSTATSENSEMBLUCSC | 238 | 5 | 39945 | island | 0.82414 | 0.95893 | Not queried | 1.44 | 5.56e-02 | 1.00e+00 |
|  | 8 | 103666796 | 103667415 | PDFSTATSENSEMBLUCSC | 620 | 5 | 39324 | gene;tss;island | 0.02520 | 0.05453 | Not queried | 1.44 | 1.51e-01 | 1.00e+00 |
|  | 1 | 156549118 | 156549431 | PDFSTATSENSEMBLUCSC | 314 | 4 | 2013 | tss | 0.61888 | 0.40737 | Not queried | 1.44 | 1.14e-01 | 1.00e+00 |
|  | X | 2835913 | 2836300 | PDFSTATSENSEMBLUCSC | 388 | 6 | 40924 | gene;island | 0.82366 | 0.90706 | Not queried | 1.43 | 1.32e-01 | 1.00e+00 |
|  | 18 | 19284713 | 19285051 | PDFSTATSENSEMBLUCSC | 339 | 10 | 19385 | island | 0.02713 | 0.09384 | Not queried | 1.43 | 8.92e-02 | 1.00e+00 |
|  | 16 | 88933068 | 88933359 | PDFSTATSENSEMBLUCSC | 292 | 4 | 15387 | tss;island | 0.46784 | 0.67263 | Not queried | 1.43 | 2.00e-01 | 1.00e+00 |
|  | 8 | 145848921 | 145849607 | PDFSTATSENSEMBLUCSC | 687 | 8 | 40679 | island | 0.81355 | 0.93647 | Not queried | 1.43 | 2.34e-01 | 1.00e+00 |
|  | 13 | 113759771 | 113760101 | PDFSTATSENSEMBLUCSC | 331 | 5 | 11519 | tss | 0.73379 | 0.87368 | Not queried | 1.43 | 2.22e-01 | 1.00e+00 |
|  | 13 | 84455525 | 84455742 | PDFSTATSENSEMBLUCSC | 218 | 4 | 11157 | gene;island | 0.04822 | 0.13382 | Not queried | 1.43 | 4.86e-01 | 1.00e+00 |
|  | 2 | 81694296 | 81694608 | PDFSTATSENSEMBLUCSC | 313 | 5 | 24428 | island | 0.94249 | 0.86369 | Not queried | 1.43 | 9.52e-02 | 1.00e+00 |
|  | 6 | 32808504 | 32808918 | PDFSTATSENSEMBLUCSC | 415 | 7 | 34082 | gene;island | 0.75633 | 0.50647 | Not queried | 1.43 | 1.28e-01 | 1.00e+00 |
|  | 12 | 42681744 | 42682194 | PDFSTATSENSEMBLUCSC | 451 | 5 | 10304 | island | 0.86049 | 0.90788 | Not queried | 1.43 | 3.10e-01 | 1.00e+00 |
|  | 14 | 24457893 | 24458345 | PDFSTATSENSEMBLUCSC | 453 | 10 | 12695 | island | 0.07751 | 0.17684 | Not queried | 1.43 | 2.80e-01 | 1.00e+00 |
|  | 4 | 186741788 | 186742139 | PDFSTATSENSEMBLUCSC | 352 | 5 | 30166 | gene | 0.73729 | 0.88006 | Not queried | 1.43 | 1.59e-02 | 1.00e+00 |
|  | X | 69396942 | 69397273 | PDFSTATSENSEMBLUCSC | 332 | 5 | 41365 | tss | 0.25984 | 0.73113 | Not queried | 1.43 | 1.51e-01 | 1.00e+00 |
|  | X | 130192035 | 130192365 | PDFSTATSENSEMBLUCSC | 331 | 7 | 42030 | island | 0.21880 | 0.08451 | Not queried | 1.43 | 2.62e-02 | 1.00e+00 |
|  | 7 | 193229 | 193732 | PDFSTATSENSEMBLUCSC | 504 | 5 | 36639 | gene;island | 0.04116 | 0.04583 | Not queried | 1.43 | 3.10e-01 | 1.00e+00 |
|  | 11 | 67188491 | 67188871 | PDFSTATSENSEMBLUCSC | 381 | 5 | 6736 | gene;island | 0.01090 | 0.01170 | Not queried | 1.43 | 5.48e-01 | 1.00e+00 |
|  | 1 | 27718858 | 27719045 | PDFSTATSENSEMBLUCSC | 188 | 4 | 1408 | tss;island | 0.01632 | 0.01032 | Not queried | 1.43 | 3.43e-01 | 1.00e+00 |
|  | 10 | 96305598 | 96305691 | PDFSTATSENSEMBLUCSC | 94 | 4 | 4672 | gene | 0.01680 | 0.03120 | Not queried | 1.43 | 3.43e-01 | 1.00e+00 |
|  | 10 | 120354627 | 120354870 | PDFSTATSENSEMBLUCSC | 244 | 4 | 4775 | gene;island | 0.35675 | 0.68530 | Not queried | 1.43 | 2.00e-01 | 1.00e+00 |
|  | 14 | 105616988 | 105617308 | PDFSTATSENSEMBLUCSC | 321 | 4 | 12228 | gene;island | 0.67109 | 0.84789 | Not queried | 1.43 | 4.86e-01 | 1.00e+00 |
|  | 6 | 11044877 | 11044974 | PDFSTATSENSEMBLUCSC | 98 | 4 | 34569 | tss;island | 0.01431 | 0.03383 | Not queried | 1.43 | 3.43e-01 | 1.00e+00 |
|  | 3 | 55522611 | 55522835 | PDFSTATSENSEMBLUCSC | 225 | 5 | 29173 | island | 0.00784 | 0.02085 | Not queried | 1.43 | 4.21e-01 | 1.00e+00 |
|  | 2 | 73053217 | 73053592 | PDFSTATSENSEMBLUCSC | 376 | 8 | 23395 | tss;island | 0.06281 | 0.02694 | Not queried | 1.43 | 5.74e-01 | 1.00e+00 |
|  | 13 | 36050844 | 36051073 | PDFSTATSENSEMBLUCSC | 230 | 7 | 11362 | tss | 0.03226 | 0.14117 | Not queried | 1.43 | 2.59e-01 | 1.00e+00 |
|  | 22 | 18507401 | 18507747 | PDFSTATSENSEMBLUCSC | 347 | 5 | 26905 | tss;island | 0.02724 | 0.19864 | Not queried | 1.43 | 2.22e-01 | 1.00e+00 |
|  | 1 | 244213358 | 244213618 | PDFSTATSENSEMBLUCSC | 261 | 5 | 2350 | tss;island | 0.07411 | 0.04213 | Not queried | 1.43 | 3.17e-02 | 1.00e+00 |
|  | 7 | 100484435 | 100484748 | PDFSTATSENSEMBLUCSC | 314 | 4 | 37087 | gene;island | 0.76498 | 0.89397 | Not queried | 1.43 | 4.86e-01 | 1.00e+00 |
|  | 15 | 25307169 | 25307422 | PDFSTATSENSEMBLUCSC | 254 | 4 | 13493 | tss | 0.92013 | 0.97126 | Not queried | 1.43 | 3.43e-01 | 1.00e+00 |
|  | 6 | 41339246 | 41340058 | PDFSTATSENSEMBLUCSC | 813 | 7 | 36057 | island | 0.04578 | 0.09848 | Not queried | 1.43 | 5.30e-02 | 1.00e+00 |
|  | 18 | 35145353 | 35145650 | PDFSTATSENSEMBLUCSC | 298 | 4 | 19062 | gene;island | 0.02860 | 0.03097 | Not queried | 1.43 | 4.86e-01 | 1.00e+00 |
|  | 4 | 7436693 | 7437156 | PDFSTATSENSEMBLUCSC | 464 | 6 | 29892 | gene | 0.42404 | 0.65598 | Not queried | 1.43 | 1.52e-02 | 1.00e+00 |
|  | 4 | 40751717 | 40751845 | PDFSTATSENSEMBLUCSC | 129 | 4 | 30313 | tss | 0.07757 | 0.18812 | Not queried | 1.43 | 4.86e-01 | 1.00e+00 |
|  | 4 | 26492153 | 26492488 | PDFSTATSENSEMBLUCSC | 336 | 6 | 30290 | tss | 0.60177 | 0.69053 | Not queried | 1.43 | 3.94e-01 | 1.00e+00 |
|  | 20 | 30060822 | 30061044 | PDFSTATSENSEMBLUCSC | 223 | 5 | 25619 | tss;island | 0.70818 | 0.50025 | Not queried | 1.43 | 7.94e-03 | 1.00e+00 |
|  | 7 | 157414294 | 157414580 | PDFSTATSENSEMBLUCSC | 287 | 4 | 37270 | gene;island | 0.89141 | 0.96828 | Not queried | 1.43 | 4.86e-01 | 1.00e+00 |
|  | 12 | 54388841 | 54389264 | PDFSTATSENSEMBLUCSC | 424 | 5 | 10461 | island | 0.02932 | 0.06992 | Not queried | 1.43 | 1.51e-01 | 1.00e+00 |
|  | 4 | 145568182 | 145568399 | PDFSTATSENSEMBLUCSC | 218 | 4 | 30096 | gene;island | 0.06739 | 0.20368 | Not queried | 1.43 | 3.43e-01 | 1.00e+00 |
|  | 3 | 123699145 | 123699389 | PDFSTATSENSEMBLUCSC | 245 | 4 | 27878 | gene | 0.07541 | 0.18640 | Not queried | 1.43 | 4.86e-01 | 1.00e+00 |
|  | 19 | 58446312 | 58446988 | PDFSTATSENSEMBLUCSC | 677 | 12 | 22450 | island | 0.03024 | 0.06135 | Not queried | 1.43 | 1.73e-02 | 1.00e+00 |
|  | 14 | 47120795 | 47121002 | PDFSTATSENSEMBLUCSC | 208 | 4 | 12047 | gene | 0.67174 | 0.84560 | Not queried | 1.43 | 1.14e-01 | 1.00e+00 |
|  | 7 | 4218743 | 4219174 | PDFSTATSENSEMBLUCSC | 432 | 4 | 36770 | gene | 0.75999 | 0.87313 | Not queried | 1.42 | 5.71e-02 | 1.00e+00 |
|  | 10 | 102989254 | 102989376 | PDFSTATSENSEMBLUCSC | 123 | 8 | 5289 | tss;island | 0.03783 | 0.06339 | Not queried | 1.42 | 1.95e-01 | 1.00e+00 |
|  | 19 | 33571484 | 33571768 | PDFSTATSENSEMBLUCSC | 285 | 4 | 20645 | tss | 0.29024 | 0.11429 | Not queried | 1.42 | 3.43e-01 | 1.00e+00 |
|  | 1 | 228403127 | 228403515 | PDFSTATSENSEMBLUCSC | 389 | 5 | 1047 | gene;island | 0.79424 | 0.91925 | Not queried | 1.42 | 9.52e-02 | 1.00e+00 |
|  | 2 | 97171137 | 97171449 | PDFSTATSENSEMBLUCSC | 313 | 5 | 22744 | gene;island | 0.52458 | 0.77508 | Not queried | 1.42 | 5.56e-02 | 1.00e+00 |
|  | 19 | 44716462 | 44716642 | PDFSTATSENSEMBLUCSC | 181 | 5 | 20810 | tss | 0.01543 | 0.03443 | Not queried | 1.42 | 7.94e-03 | 1.00e+00 |
|  | 4 | 949314 | 949689 | PDFSTATSENSEMBLUCSC | 376 | 4 | 29805 | gene;island | 0.85461 | 0.95628 | Not queried | 1.42 | 2.00e-01 | 1.00e+00 |
|  | 11 | 65600739 | 65600888 | PDFSTATSENSEMBLUCSC | 150 | 4 | 7421 | tss;island | 0.31783 | 0.12872 | Not queried | 1.42 | 2.00e-01 | 1.00e+00 |
|  | 18 | 21017489 | 21017905 | PDFSTATSENSEMBLUCSC | 417 | 5 | 19039 | gene | 0.00835 | 0.01373 | Not queried | 1.42 | 4.21e-01 | 1.00e+00 |
|  | 14 | 105964848 | 105965379 | PDFSTATSENSEMBLUCSC | 532 | 5 | 12239 | gene;island | 0.76394 | 0.92434 | Not queried | 1.42 | 3.17e-02 | 1.00e+00 |
|  | 15 | 75019070 | 75019376 | PDFSTATSENSEMBLUCSC | 307 | 10 | 13772 | tss;island | 0.81426 | 0.65842 | Not queried | 1.42 | 1.43e-01 | 1.00e+00 |
|  | 6 | 29576818 | 29577348 | PDFSTATSENSEMBLUCSC | 531 | 6 | 33605 | gene | 0.72884 | 0.81977 | Not queried | 1.42 | 2.40e-01 | 1.00e+00 |
|  | 4 | 2935692 | 2936284 | PDFSTATSENSEMBLUCSC | 593 | 9 | 29851 | gene;island | 0.04493 | 0.07081 | Not queried | 1.42 | 1.36e-01 | 1.00e+00 |
|  | 8 | 145162905 | 145163136 | PDFSTATSENSEMBLUCSC | 232 | 4 | 39448 | gene;island | 0.75614 | 0.92478 | Not queried | 1.42 | 1.14e-01 | 1.00e+00 |
|  | X | 54666063 | 54666407 | PDFSTATSENSEMBLUCSC | 345 | 4 | 41815 | island | 0.13481 | 0.02773 | Not queried | 1.42 | 1.14e-01 | 1.00e+00 |
|  | 17 | 79520615 | 79521089 | PDFSTATSENSEMBLUCSC | 475 | 5 | 18897 | island | 0.55868 | 0.83902 | Not queried | 1.42 | 2.22e-01 | 1.00e+00 |
|  | X | 34150470 | 34150583 | PDFSTATSENSEMBLUCSC | 114 | 4 | 41249 | tss | 0.74881 | 0.87615 | Not queried | 1.42 | 2.86e-02 | 1.00e+00 |
|  | 1 | 22140769 | 22141400 | PDFSTATSENSEMBLUCSC | 632 | 7 | 300 | gene;island | 0.23423 | 0.25952 | Not queried | 1.42 | 5.35e-01 | 1.00e+00 |
|  | 1 | 111889241 | 111889533 | PDFSTATSENSEMBLUCSC | 293 | 5 | 681 | gene | 0.04068 | 0.07902 | Not queried | 1.42 | 5.56e-02 | 1.00e+00 |
|  | 22 | 38902403 | 38902925 | PDFSTATSENSEMBLUCSC | 523 | 8 | 27099 | tss | 0.00946 | 0.02896 | Not queried | 1.42 | 1.30e-01 | 1.00e+00 |
|  | 22 | 38902252 | 38902925 | PDFSTATSENSEMBLUCSC | 674 | 10 | 27449 | island | 0.00946 | 0.02808 | Not queried | 1.42 | 8.92e-02 | 1.00e+00 |
|  | 19 | 16435203 | 16435660 | PDFSTATSENSEMBLUCSC | 458 | 5 | 21681 | island | 0.02674 | 0.14748 | Not queried | 1.42 | 6.90e-01 | 1.00e+00 |
|  | 3 | 46607965 | 46608179 | PDFSTATSENSEMBLUCSC | 215 | 6 | 27708 | gene;island | 0.17890 | 0.09009 | Not queried | 1.42 | 1.32e-01 | 1.00e+00 |
|  | 8 | 61590588 | 61590787 | PDFSTATSENSEMBLUCSC | 200 | 4 | 39662 | tss;island | 0.01601 | 0.06325 | Not queried | 1.42 | 3.43e-01 | 1.00e+00 |
|  | 4 | 189580331 | 189580798 | PDFSTATSENSEMBLUCSC | 468 | 5 | 31438 | island | 0.75347 | 0.88658 | Not queried | 1.42 | 2.22e-01 | 1.00e+00 |
|  | X | 73523856 | 73523965 | PDFSTATSENSEMBLUCSC | 110 | 4 | 41392 | tss | 0.77336 | 0.91533 | Not queried | 1.42 | 1.14e-01 | 1.00e+00 |
|  | 14 | 37116121 | 37116408 | PDFSTATSENSEMBLUCSC | 288 | 4 | 12760 | island | 0.22969 | 0.07001 | Not queried | 1.42 | 3.43e-01 | 1.00e+00 |
|  | 11 | 67206263 | 67206434 | PDFSTATSENSEMBLUCSC | 172 | 4 | 6738 | gene;tss;island | 0.76277 | 0.90612 | Not queried | 1.42 | 3.43e-01 | 1.00e+00 |
|  | 2 | 224904626 | 224904837 | PDFSTATSENSEMBLUCSC | 212 | 5 | 23886 | tss;island | 0.13770 | 0.05613 | Not queried | 1.42 | 1.51e-01 | 1.00e+00 |
|  | 11 | 64108790 | 64109158 | PDFSTATSENSEMBLUCSC | 369 | 4 | 6665 | gene;island | 0.70825 | 0.56071 | Not queried | 1.42 | 6.86e-01 | 1.00e+00 |
|  | 18 | 12656824 | 12657166 | PDFSTATSENSEMBLUCSC | 343 | 4 | 19028 | gene;tss;island | 0.03605 | 0.03298 | Not queried | 1.42 | 8.86e-01 | 1.00e+00 |
|  | 22 | 25758455 | 25758749 | PDFSTATSENSEMBLUCSC | 295 | 5 | 26780 | gene;island | 0.15022 | 0.70438 | Not queried | 1.41 | 5.48e-01 | 1.00e+00 |
|  | 17 | 40687822 | 40687943 | PDFSTATSENSEMBLUCSC | 122 | 7 | 17331 | tss | 0.04684 | 0.25767 | Not queried | 1.41 | 2.59e-01 | 1.00e+00 |
|  | 1 | 3566701 | 3568245 | PDFSTATSENSEMBLUCSC | 1545 | 34 | 1179 | tss | 0.58263 | 0.40863 | Not queried | 1.41 | 1.23e-02 | 1.00e+00 |
|  | 7 | 72349822 | 72349862 | PDFSTATSENSEMBLUCSC | 41 | 4 | 37565 | tss | 0.02883 | 0.01055 | Not queried | 1.41 | 1.14e-01 | 1.00e+00 |
|  | 6 | 30175792 | 30176107 | PDFSTATSENSEMBLUCSC | 316 | 5 | 33688 | gene | 0.83726 | 0.91149 | Not queried | 1.41 | 3.10e-01 | 1.00e+00 |
|  | 16 | 55512806 | 55513017 | PDFSTATSENSEMBLUCSC | 212 | 5 | 15208 | tss | 0.01506 | 0.02773 | Not queried | 1.41 | 5.48e-01 | 1.00e+00 |
|  | 17 | 46651822 | 46651952 | PDFSTATSENSEMBLUCSC | 131 | 5 | 17425 | tss | 0.50860 | 0.27984 | Not queried | 1.41 | 2.22e-01 | 1.00e+00 |
|  | 6 | 29760856 | 29761044 | PDFSTATSENSEMBLUCSC | 189 | 6 | 34691 | tss | 0.13051 | 0.26243 | Not queried | 1.41 | 2.40e-01 | 1.00e+00 |
|  | 12 | 105501526 | 105501569 | PDFSTATSENSEMBLUCSC | 44 | 4 | 9250 | gene | 0.00549 | 0.01352 | Not queried | 1.41 | 2.86e-02 | 1.00e+00 |
|  | 12 | 110271439 | 110271625 | PDFSTATSENSEMBLUCSC | 187 | 4 | 9971 | tss;island | 0.04868 | 0.10333 | Not queried | 1.41 | 2.00e-01 | 1.00e+00 |
|  | 16 | 56622951 | 56623549 | PDFSTATSENSEMBLUCSC | 599 | 10 | 15914 | island | 0.21636 | 0.06870 | Not queried | 1.41 | 4.36e-01 | 1.00e+00 |
|  | 1 | 44456953 | 44457406 | PDFSTATSENSEMBLUCSC | 454 | 8 | 3171 | island | 0.02709 | 0.04496 | Not queried | 1.41 | 2.79e-01 | 1.00e+00 |
|  | 6 | 64281439 | 64281886 | PDFSTATSENSEMBLUCSC | 448 | 8 | 34990 | tss | 0.02459 | 0.01907 | Not queried | 1.41 | 5.74e-01 | 1.00e+00 |
|  | 16 | 1221825 | 1222188 | PDFSTATSENSEMBLUCSC | 364 | 4 | 14528 | gene;island | 0.70571 | 0.85786 | Not queried | 1.41 | 3.43e-01 | 1.00e+00 |
|  | 17 | 80351032 | 80351457 | PDFSTATSENSEMBLUCSC | 426 | 5 | 16912 | gene;island | 0.44913 | 0.69016 | Not queried | 1.41 | 9.52e-02 | 1.00e+00 |
|  | 16 | 81348309 | 81348534 | PDFSTATSENSEMBLUCSC | 226 | 5 | 15351 | tss;island | 0.00722 | 0.01710 | Not queried | 1.41 | 4.21e-01 | 1.00e+00 |
|  | 8 | 145641991 | 145642456 | PDFSTATSENSEMBLUCSC | 466 | 10 | 39918 | tss | 0.67243 | 0.85475 | Not queried | 1.41 | 1.50e-03 | 1.00e+00 |
|  | 8 | 28560467 | 28560723 | PDFSTATSENSEMBLUCSC | 257 | 5 | 39199 | gene;island | 0.30149 | 0.13347 | Not queried | 1.41 | 1.51e-01 | 1.00e+00 |
|  | 17 | 53800225 | 53800554 | PDFSTATSENSEMBLUCSC | 330 | 4 | 17486 | tss | 0.04651 | 0.11638 | Not queried | 1.41 | 3.43e-01 | 1.00e+00 |
|  | 5 | 63985282 | 63985489 | PDFSTATSENSEMBLUCSC | 208 | 4 | 32031 | tss;island | 0.63241 | 0.50622 | Not queried | 1.41 | 4.86e-01 | 1.00e+00 |
|  | 11 | 1096283 | 1096455 | PDFSTATSENSEMBLUCSC | 173 | 4 | 6386 | gene;island | 0.74738 | 0.88416 | Not queried | 1.41 | 1.14e-01 | 1.00e+00 |
|  | 10 | 102766322 | 102766610 | PDFSTATSENSEMBLUCSC | 289 | 4 | 4705 | gene;island | 0.74686 | 0.89770 | Not queried | 1.41 | 1.14e-01 | 1.00e+00 |
|  | 1 | 3664522 | 3664680 | PDFSTATSENSEMBLUCSC | 159 | 4 | 1185 | tss;island | 0.40335 | 0.48088 | Not queried | 1.41 | 6.86e-01 | 1.00e+00 |
|  | 3 | 156544070 | 156544081 | PDFSTATSENSEMBLUCSC | 12 | 4 | 28634 | tss | 0.29215 | 0.50981 | Not queried | 1.41 | 2.86e-02 | 1.00e+00 |
|  | 15 | 27111388 | 27112197 | PDFSTATSENSEMBLUCSC | 810 | 21 | 13513 | tss | 0.68389 | 0.42611 | Not queried | 1.41 | 1.82e-02 | 1.00e+00 |
|  | 1 | 66258022 | 66258134 | PDFSTATSENSEMBLUCSC | 113 | 4 | 1675 | tss;island | 0.04013 | 0.10120 | Not queried | 1.41 | 2.86e-02 | 1.00e+00 |
|  | 2 | 242497960 | 242498098 | PDFSTATSENSEMBLUCSC | 139 | 4 | 23953 | tss;island | 0.18637 | 0.05883 | Not queried | 1.41 | 4.86e-01 | 1.00e+00 |
|  | 17 | 19771360 | 19771813 | PDFSTATSENSEMBLUCSC | 454 | 7 | 17163 | tss;island | 0.31615 | 0.09823 | Not queried | 1.41 | 3.18e-01 | 1.00e+00 |
|  | 2 | 127414802 | 127415063 | PDFSTATSENSEMBLUCSC | 262 | 4 | 22817 | gene;island | 0.34547 | 0.15461 | Not queried | 1.41 | 2.00e-01 | 1.00e+00 |
|  | 16 | 29153044 | 29153407 | PDFSTATSENSEMBLUCSC | 364 | 5 | 15767 | island | 0.43668 | 0.60692 | Not queried | 1.41 | 5.56e-02 | 1.00e+00 |
|  | 2 | 129076335 | 129076394 | PDFSTATSENSEMBLUCSC | 60 | 5 | 23585 | tss;island | 0.39514 | 0.63362 | Not queried | 1.41 | 5.56e-02 | 1.00e+00 |
|  | 5 | 177581310 | 177581771 | PDFSTATSENSEMBLUCSC | 462 | 6 | 32477 | tss;island | 0.43111 | 0.67991 | Not queried | 1.41 | 1.32e-01 | 1.00e+00 |
|  | 11 | 2554198 | 2554583 | PDFSTATSENSEMBLUCSC | 386 | 6 | 6444 | gene;island | 0.27450 | 0.44230 | Not queried | 1.41 | 3.94e-01 | 1.00e+00 |
|  | X | 154842689 | 154842856 | PDFSTATSENSEMBLUCSC | 168 | 5 | 41614 | tss | 0.05008 | 0.05370 | Not queried | 1.41 | 1.00e+00 | 1.00e+00 |
|  | 1 | 153329781 | 153330257 | PDFSTATSENSEMBLUCSC | 477 | 6 | 1937 | tss | 0.66426 | 0.35543 | Not queried | 1.40 | 8.66e-03 | 1.00e+00 |
|  | 10 | 118956639 | 118956998 | PDFSTATSENSEMBLUCSC | 360 | 7 | 5372 | tss | 0.81330 | 0.63496 | Not queried | 1.40 | 3.79e-02 | 1.00e+00 |
|  | 6 | 30124214 | 30124299 | PDFSTATSENSEMBLUCSC | 86 | 4 | 33668 | gene | 0.61899 | 0.76132 | Not queried | 1.40 | 2.00e-01 | 1.00e+00 |
|  | 16 | 58718767 | 58718971 | PDFSTATSENSEMBLUCSC | 205 | 4 | 15245 | tss | 0.64089 | 0.42357 | Not queried | 1.40 | 3.43e-01 | 1.00e+00 |
|  | 6 | 142409417 | 142410235 | PDFSTATSENSEMBLUCSC | 819 | 13 | 36461 | island | 0.03230 | 0.10883 | Not queried | 1.40 | 1.40e-02 | 1.00e+00 |
|  | 13 | 24734712 | 24734795 | PDFSTATSENSEMBLUCSC | 84 | 4 | 11317 | tss | 0.03471 | 0.09154 | Not queried | 1.40 | 1.14e-01 | 1.00e+00 |
|  | 11 | 2151676 | 2151725 | PDFSTATSENSEMBLUCSC | 50 | 4 | 6426 | gene;island | 0.55941 | 0.85173 | Not queried | 1.40 | 2.00e-01 | 1.00e+00 |
|  | 8 | 144521374 | 144521607 | PDFSTATSENSEMBLUCSC | 234 | 7 | 39414 | gene;island | 0.85133 | 0.91565 | Not queried | 1.40 | 5.30e-02 | 1.00e+00 |
|  | X | 9912520 | 9912880 | PDFSTATSENSEMBLUCSC | 361 | 5 | 40932 | gene;island | 0.86385 | 0.94214 | Not queried | 1.40 | 2.22e-01 | 1.00e+00 |
|  | 3 | 128444572 | 128444915 | PDFSTATSENSEMBLUCSC | 344 | 6 | 28529 | tss | 0.02198 | 0.03313 | Not queried | 1.40 | 5.89e-01 | 1.00e+00 |
|  | 5 | 95768915 | 95769079 | PDFSTATSENSEMBLUCSC | 165 | 7 | 32911 | island | 0.15122 | 0.02543 | Not queried | 1.40 | 1.28e-01 | 1.00e+00 |
|  | 1 | 2984245 | 2984869 | PDFSTATSENSEMBLUCSC | 625 | 9 | 1175 | tss | 0.61376 | 0.81123 | Not queried | 1.40 | 2.44e-02 | 1.00e+00 |
|  | 19 | 4090224 | 4090587 | PDFSTATSENSEMBLUCSC | 364 | 4 | 21328 | island | 0.71488 | 0.85581 | Not queried | 1.40 | 2.86e-02 | 1.00e+00 |
|  | 6 | 168197410 | 168198163 | PDFSTATSENSEMBLUCSC | 754 | 9 | 36586 | island | 0.55468 | 0.76149 | Not queried | 1.40 | 1.88e-02 | 1.00e+00 |
|  | 8 | 1094484 | 1094904 | PDFSTATSENSEMBLUCSC | 421 | 6 | 39948 | island | 0.83891 | 0.91239 | Not queried | 1.40 | 9.31e-02 | 1.00e+00 |
|  | 1 | 28414998 | 28415118 | PDFSTATSENSEMBLUCSC | 121 | 5 | 362 | gene | 0.01483 | 0.03493 | Not queried | 1.40 | 9.52e-02 | 1.00e+00 |
|  | 11 | 19736150 | 19736333 | PDFSTATSENSEMBLUCSC | 184 | 5 | 6517 | gene;tss;island | 0.37189 | 0.44978 | Not queried | 1.40 | 1.59e-02 | 1.00e+00 |
